# Photocatalytic Controlled Halodefluorination of Perfluoroalkyl Compounds Using *N*‑Arylphenothiazines

**DOI:** 10.1021/jacs.6c07703

**Published:** 2026-07-02

**Authors:** Michael G. J. Doyle, Maria Elgaard Jespersen, Shana Noureen, Zijun Chen, Theodore L. Jefferson, Caitlin A. Baptista, Marie Houot, Job J. C. Struijs, Christopher A. Goult, Artem A. Bakulin, Robert S. Paton, Alyssa-Jennifer Avestro, Véronique Gouverneur

**Affiliations:** 1 Department of Chemistry, Chemistry Research Laboratory, 6396 University of Oxford, Oxford OX1 3TA, United Kingdom; 2 Department of Chemistry and Centre for Processable Electronics, 4615 Imperial College London, London W12 0BZ, United Kingdom; 3 Department of Chemistry, 224023 Colorado State University, Fort Collins, Colorado 80528, United States

## Abstract

Recent advances in C–F bond activation of per- and polyfluoroalkyl substances (PFAS) have enabled complete degradation of fluorochemical waste, yet achieving precise, site-selective monodefluorination continues to present a significant synthetic challenge. Here, we demonstrate that *N*-arylphenothiazine photocatalysts enable controlled halodefluorination of perfluoroalkyl compounds using simple halide salts. Mechanistic studies support a consecutive photoinduced electron transfer (conPET) manifold, where the photocatalyst operates as a potent excited-state reductant and oxidant in succession under single- or multiwavelength irradiation. Single-electron halide oxidation is mediated by a transient radical cation superoxidant *­[PC]^•+^, which facilitates net halogen metathesis with high fidelity. This study further exemplifies the redox versatility of *N*-arylphenothiazine photosensitizers and contributes to the development of mechanistically diverse methods for functionalization of strong C–F bonds in polyfluorinated molecules.

## Graphical abstract

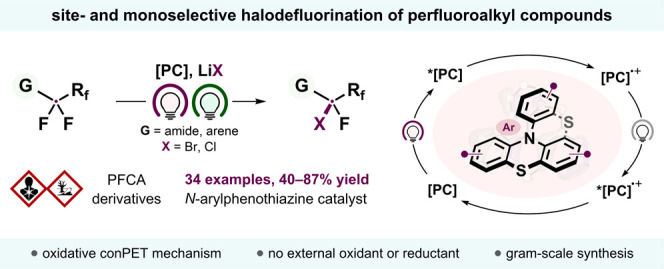

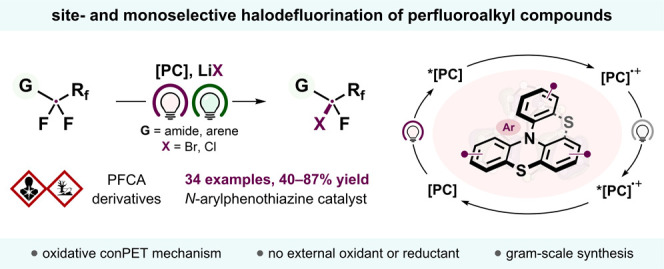

## Introduction

Photoredox catalysis remains at the forefront of small molecule activation methods in organic chemistry.^1–4^ Light-induced processes offer distinct advantages over traditional synthetic strategies such as mild reaction conditions, unique reactivity pathways, and the ability for photosensitizers to act simultaneously as both oxidants and reductants.^5–8^ The recent advent of consecutive photoinduced electron transfer (conPET), whereby multiple photons can be harnessed in a single catalytic turnover, has enabled access to excited-state radicals with redox potentials beyond what is typically achievable in conventional single-photon photoredox catalysis (Figure 1a).^9–17^ Reductive transformations driven by multiphoton absorption are well-established using a myriad of catalytic systems,^18,19^ while analogous oxidative approaches commonly employ *N*-phenylphenothiazine (PTH) or electron-poor triarylamines as photocatalysts, which have been used for the oxidation of arenes,^20–23^ styrenes,^24–27^ and chloride ions^28^ (Figure 1a). Although PTH functions as a potent excited-state oxidant in these contexts (*E* ^red^ 2.1–2.5 V vs SCE for *PTH^•+^), *N*-arylphenothiazines have been more widely applied as excited-state photoreductants (*E* ^ox^ −2.5 V vs SCE for *PTH) in a variety of organic transformations, underscoring their redox versatility (Figure 1b).^29–32^ Protocols that simultaneously exploit both the strongly oxidizing and reducing properties of *N*-arylphenothiazines under oxidative conPET conditions in the absence of terminal oxidants or reductants remain less explored.^24–27,33^ Expanding the repertoire of transformations that capitalize on the sizable excited-state redox window of these photosensitizers (Δ*E* up to ∼5.0 V for PTH, Figure 1b) would be highly desirable, especially to address unmet synthetic needs.

**Figure 1:**
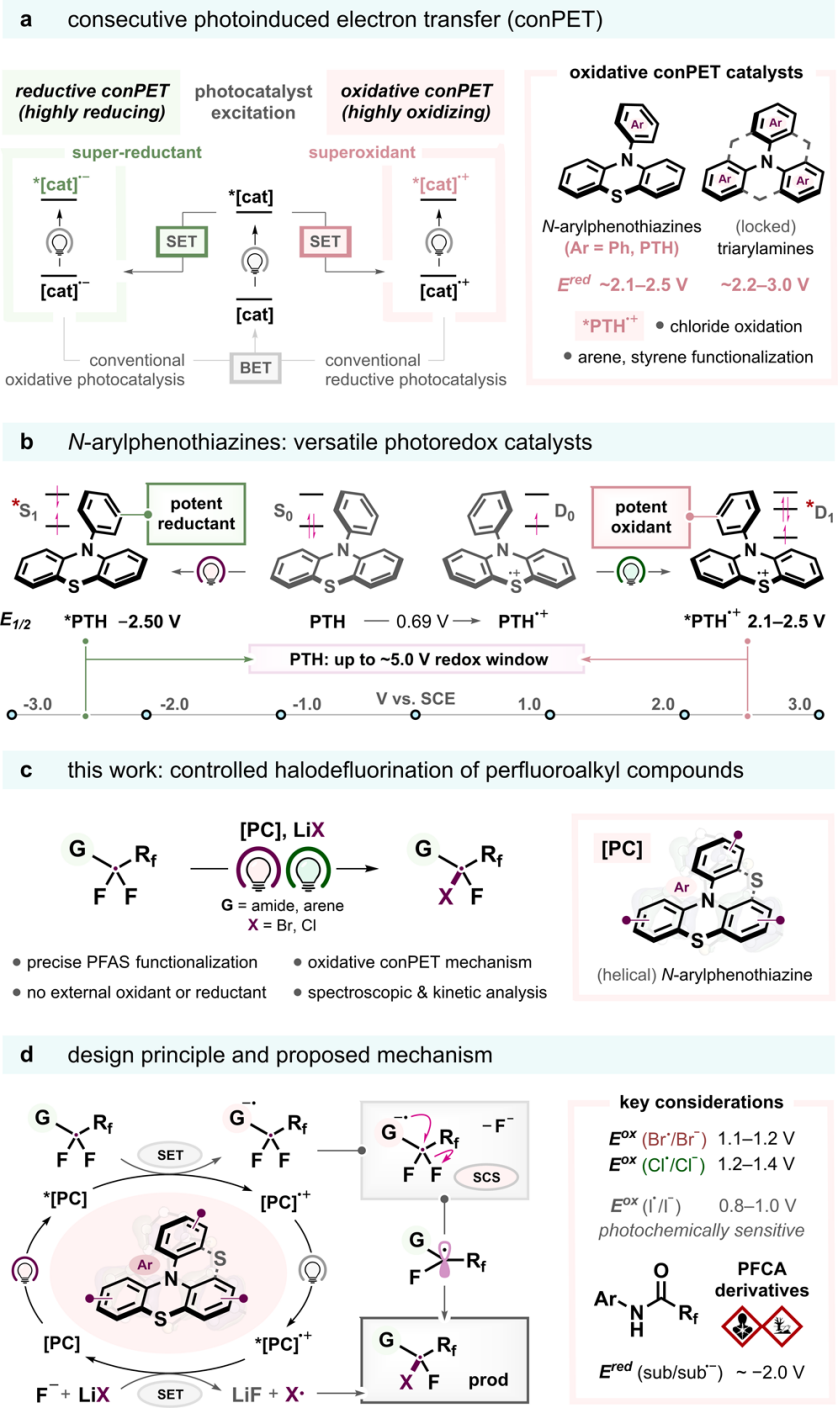
*N*-Arylphenothiazines in multiphoton photoredox catalysis. (a) Overview of consecutive photoinduced electron transfer (conPET). (b) *N*-Arylphenothiazines can act as potent excited-state photoreductants or photooxidants. (c) This work describes controlled halodefluorination of perfluoroalkyl compounds catalyzed by *N*-arylphenothiazines through an oxidative conPET mechanism. (d) Reaction design principle and mechanistic proposal. All redox potentials are listed vs SCE. SET = single-electron transfer. BET = back-electron transfer. SCS = spin-center shift. PFCA = perfluorocarboxylic acid. SCE = saturated calomel electrode.

The development of methods for the mineralization of per- and polyfluoroalkyl substances (PFAS) triggered by C–F bond cleavage has been a longstanding synthetic challenge, drawing significant interest due to their environmental persistence and adverse effects on human health.^34–37^ Recently, our group disclosed mechanochemical strategies for PFAS destruction coupled with fluorine valorization, with a circular fluorine economy in mind.^38,39^ Independent groups have expanded the scope of methods for PFAS destruction and fluoride recovery via mechanochemical activation,^40–44^ as well as complementary approaches including transfer fluorination^45^ and electrochemical defluorination,^46^ among others.^47,48^ Despite recent advances leveraging light-induced C–F bond activation, achieving site-controlled functionalization of PFAS remains a formidable challenge.^49–53^ Moreover, precise replacement of a fluorine atom with other halogen congeners remains nontrivial because the resulting Cl-, Br-, or I-containing products are typically more reactive, rendering such transformations rare despite their synthetic utility.^54–56^ Here, we demonstrate that *N*-arylphenothiazines enable site-selective monohalodefluorination of perfluoroalkylamides and -arenes with simple halide salts through an oxidative conPET catalytic pathway (Figure 1c). The photocatalyst exhibits exceptional fidelity for monodefluorination, thereby offering new opportunities for the synthetic manipulation of fluorochemical waste.

Our design principle is centered around the ability of an *N*-arylphenothiazine to act as both a powerful excited-state oxidant and reductant (Figure 1d). We hypothesized that upon UV photoexcitation, the photocatalyst *­[PC] would undergo oxidative quenching by the perfluorinated substrate.^57^ The resulting radical anion could then rearrange into a perfluoroalkyl radical through a spin-center shift (SCS) mechanism with concomitant fluoride extrusion.^58,59^ In parallel, the *N*-arylphenothiazine radical cation **[PC]** ^**•+**^ could absorb a second photon to generate a short-lived superoxidant *­[PC] ^**•+**^.^60^ Subsequent reductive quenching by a halide salt would then furnish a halogen radical (X = I, Br, or Cl) to trap the perfluoroalkyl spin intermediate while regenerating the ground-state catalyst. We anticipated the second photon absorption being crucial for bromide or chloride oxidation, as this process would be at least 0.4–0.5 V uphill transpiring from the ground-state catalyst radical cation (i.e., for PTH^•+^) and therefore energetically disfavored (Figure 1d).^61^ Coupling between the perfluoroalkyl- and halogen radicals would lead to a net halodefluorinated product, as dictated by their matched polarities.^62^ We viewed perfluoroalkylamides as appropriate model substrates for halodefluorination on the basis of their reduction potentials (*E* ^red^ ∼ −2.0 V vs SCE), which fall within the accessible range of *N*-arylphenothiazine photocatalysts.^63^

## Results and Discussion

We first examined the halodefluorination of perfluoroalkylamides using bromide sources. It was found that electron-rich *N*-arylphenothiazine **PC1** ^29^ (*E ^ox^ −2.45 V, ^•+^ *E* ^red^ 0.73 V vs SCE in MeCN) catalyzes defluorinative bromination of **1a** (*E* ^red^ −1.75 V vs SCE in MeCN) under 370 nm UV light irradiation to form **2a** in 86% ^19^F NMR yield (*n* = 15, σ = 1.9) when using LiBr (*E* ^ox^ 1.11 V vs SCE in MeCN)^64^ as the coupling partner in MeCN (Figure 2). Bisphenothiazine catalyst **PC2** ^65^ (*E ^ox^ −2.40 V, ^•+^ *E* ^red^ 0.82 V vs SCE in MeCN) delivered product in comparably high yield (83%), while *N*-aryl derivatives **PC3**–**5** and PTH (**PC6**) were generally less efficient in promoting bromodefluorination (Figure 2a). The use of donor–acceptor cyanoarenes **PC7** and **PC8**, which are known reductive conPET photosensitizers,^13^ led to no conversion of **1a** under the standard conditions. *N*-Arylphenoxazine **PC9** was able to mediate some product formation (17%) despite being a weak ground-state oxidant (^•+^ *E* ^red^ 0.43 V vs SCE in MeCN),^66^ underscoring a potential conPET mechanism for bromide oxidation. Highly reducing Ir complex **PC10** (*E ^ox^ −1.73 V vs SCE in MeCN) was unable to initiate reactivity of **1a**, similar to other precious metal sensitizers which rarely function as multiphoton absorption catalysts (see the Supporting Information (SI) for the full catalyst survey).^67^

**Figure 2:**
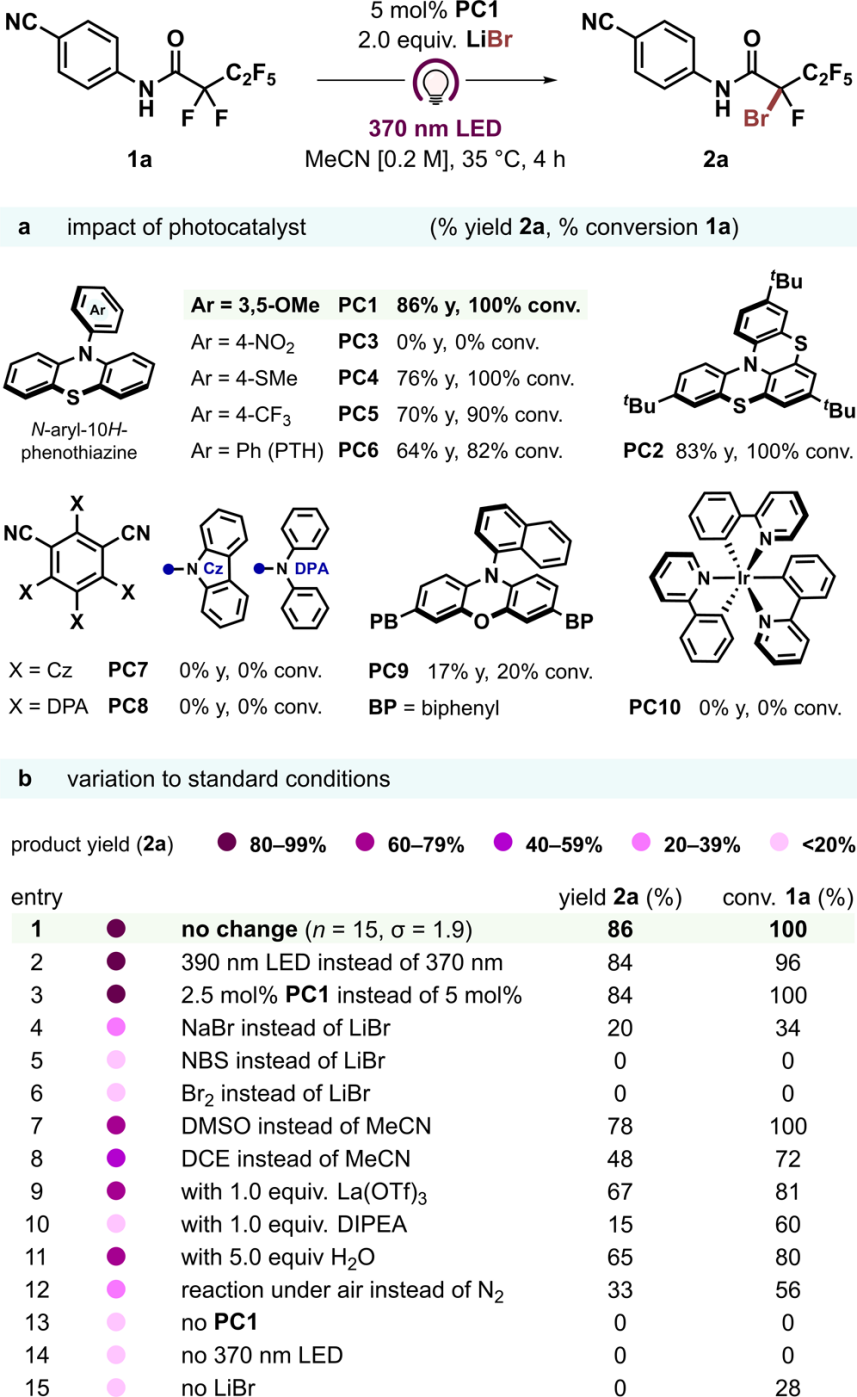
Reaction optimization for bromodefluorination of perfluoroalkylamide **1a**. (a) Photocatalyst impact on conversion of substrate **1a** and yield of product **2a**. (b) Conditions screening, additive testing, and control reactions. Reaction conversion and yields were determined by quantitative ^19^F NMR using 1-fluoronaphthalene as an internal standard.

Deviations to the standard conditions revealed that switching to a higher wavelength UV light source (Figure 2b, entry 2) or halving the catalyst loading (entry 3) had little effect on the reaction outcome. Conversely, swapping LiBr for NaBr (entry 4) significantly dampens product formation, suggesting that precipitation of LiF facilitates net halogen exchange (Δ*U*_POT(LiF–NaF)_ = +28.4 kcal/mol, see the SI for a LiF recovery experiment).^68^ Electrophilic bromine oxidants as coupling partners instead of bromide salts resulted in no consumption of **1a**, further implying the catalyst functions as a dual photoreductant/oxidant (entries 5 and 6). Reduced yields of **2a** were observed when using alternative solvents (entries 7 and 8). The presence of Lewis acidic or basic additives also resulted in diminished product yield and substrate conversion; the latter is possibly due to reductive quenching of *PC1 ^**•+**^ (entries 9 and 10).^69^ Bromodefluorination of **1a** remains operational in the presence of water or air (entries 11 and 12), although full conversion of starting material is not achieved under these conditions. Moreover, control experiments (entries 13–15) reinforced that each reaction component is required for defluorinative bromination, as no product was detected upon omission of **PC1**, light, or LiBr (see the SI for additional optimization studies).

With an efficient bromodefluorination protocol established, we investigated the photophysical and kinetic factors governing this transformation. Given that direct bromide oxidation by ground-state radical cation **PC1** ^**•+**^ is thermodynamically disfavored (Δ*E* = −0.38 V), we aimed to validate whether this process is mediated by the proposed excited-state superoxidant *PC1 ^**•+**^. UV–vis absorption studies in MeCN revealed the characteristic *N*-arylphenothiazine UV absorption profile of **PC1** (300–380 nm, ε = 3880 M^–1^ cm^–1^), whereas electrochemically generated **PC1** ^**•+**^ displayed an intense bathochromically shifted band in the 400–550 nm region (ε = 10450 M^–1^ cm^–1^, Figure 3a). Radical cation **PC1** ^**•+**^ shows moderate absorption (compared to **PC1**) within the emission range of our UV LED source (Figure 3a), supporting the feasibility of **PC1** ^**•+**^ excitation to generate *PC1 ^**•+**^ (*E* ^red^ 2.09 V vs SCE in MeCN) that is capable of bromide oxidation (see the SI for excited-state redox potential calculations). We considered that **PC1** ^**•+**^ could undergo disproportionation to generate a dicationic oxidant (**PC1** ^**2+**^, *E* ^red^ 1.42 V vs SCE in MeCN), although this process is typically favored under strongly acidic conditions^70^ and is reported to have an exceptionally small equilibrium constant in MeCN (10^–8^ M^–1^ for PTH^•+^).^28^

**Figure 3:**
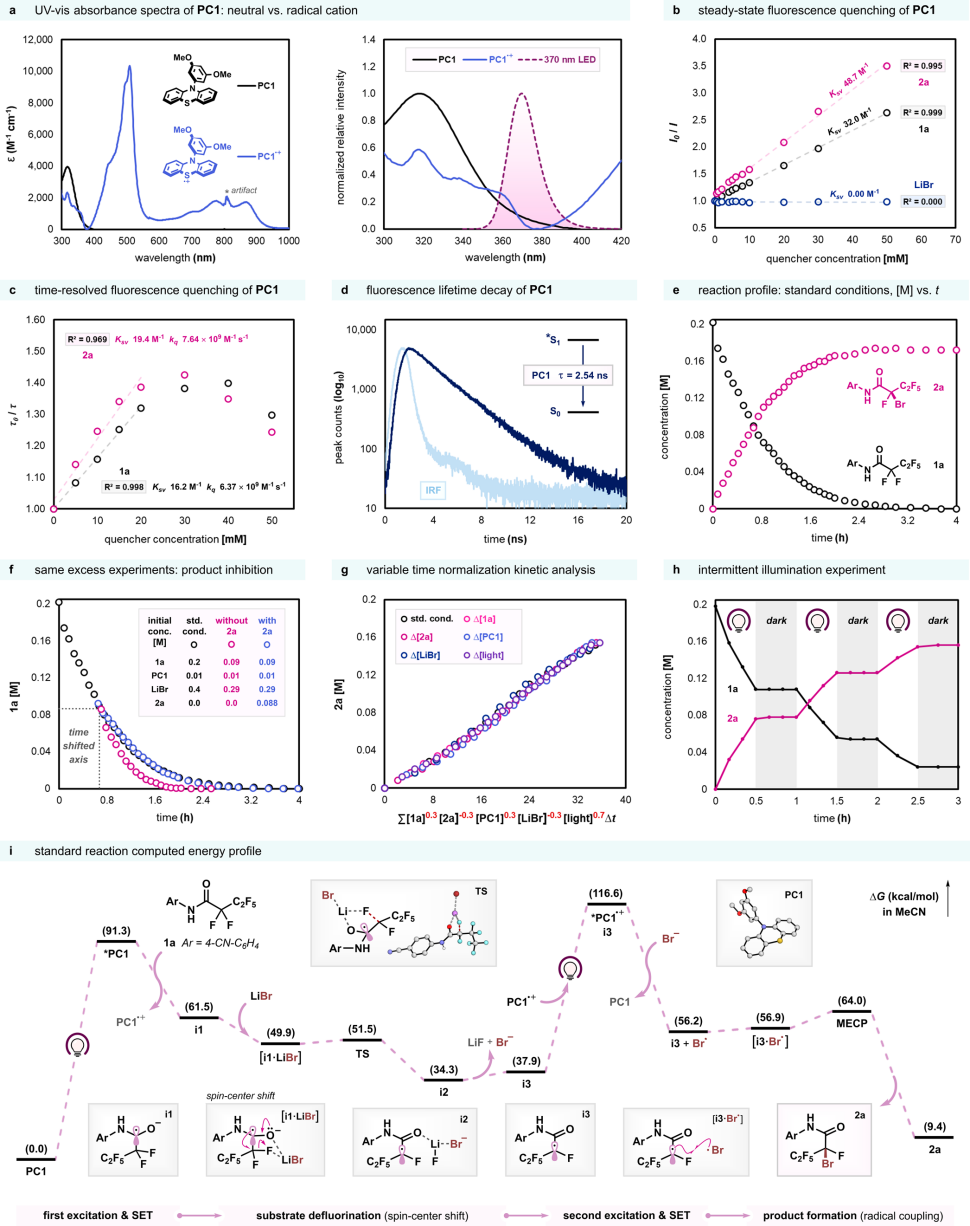
Spectroscopic, kinetic, and computational studies of the site-selective monobromodefluorination of perfluoroalkyl compounds. (a) UV–vis absorption profiles of **PC1** and the corresponding radical cation **PC1** ^**•+**^ against the 370 nm light source emission profile. (b) Steady-state fluorescence quenching studies of **PC1** with **1a**, **2a**, and LiBr. (c) Time-resolved fluorescence quenching studies of **PC1** with **1a** and **2a**. (d) Excited-state lifetime decay of **PC1** recorded by time-correlated single photon counting (TCSPC) upon excitation with a 365 nm laser (λ_exc_ = 365 nm, λ_em_ = 440 nm, 850 ps pulse width). Lifetime values were calculated using the tail-fit method (χ^2^ = 1.07). (e) Reaction profile of bromodefluorination under the standard conditions. (f) Product inhibition/catalyst degradation studies using “same excess” experiments. (g) “Different excess” experiments performed by variable time normalization kinetic analysis. (h) Intermittent illumination time-course study. (i) DFT-computed energy profile for the proposed defluorinative bromination reaction. Calculations were performed at the (U)­ωB97X-D3/def2-TZVPPD/SMD//(U)­M06-2X-D3/def2-TZVP/SMD level of theory in MeCN. Ar = 4-CN-C_6_H_4_. MECP = minimum energy crossing point.

To probe the origins of **PC1** ^**•+**^ formation under the standard conditions, Stern–Volmer fluorescence quenching studies were performed. Excited-state reductant *PC1 undergoes linearly dependent oxidative quenching by substrate **1a** with a Stern–Volmer constant (*K*_*SV*_) of 32.0 M^–1^ under steady-state conditions (Figure 3b). Although a linear relationship was likewise observed in time-resolved measurements up to concentrations of 20 mM **1a**, ostensibly indicating collisional quenching (apparent *k*_q_ = 6.37 × 10^9^ M^–1^ s^–1^), the apparent *K*_SV_ value differs from what is obtained in our steady-state studies (*K*_SV_ = 16.2 M^–1^) which indicates an additional static quenching interaction is likely present (Figure 3c).^71^ Nondynamic contributions become more pronounced at quencher concentrations >20 mM, which is reflected by the observed delineation (see the SI for a discussion regarding static quenching and inner filter effects). Brominated product **2a** (*E* ^red^ −2.20 V vs SCE in MeCN) also exhibits a similar mixed quenching mechanism (steady-state *K*_*SV*_ = 48.7 M^–1^, time-resolved apparent *K*_SV_ = 19.4 M^–1^, apparent *k*_*q*_ = 7.64 × 10^9^ M^–1^ s^–1^), suggesting it may behave as a reaction inhibitor through reversible reduction by the photocatalyst. Steady-state fluorescence bleaching of *PC1 was not observed in the sole presence of LiBr, further suggesting that the catalytic cycle likely begins with photoexcitation of **PC1** and subsequent single-electron reduction of **1a**. Triplet energy transfer represents another possible route for activation of **1a** by the photocatalyst,^72^ although it is established that *N*-arylphenothiazine photocatalysis is mainly driven by singlet excited-state reactivity.^33^ The large dynamic vertical triplet energy of **1a** (*E*_T,DvTE_ = 66.1 kcal/mol), determined from DFT calculations at the (U)­M06-2X/6-31G­(d) level of theory, in addition to the short photoluminescence lifetime of *PC1 (τ = 2.54 ns, Figure 3d) suggests triplet energy transfer is a noncontributing pathway toward oxidative quenching of *PC1 (see the SI for details regarding *E*_T,DvTE_ calculations).^73,74^

Despite behaving as a photoluminescence quencher, brominated product **2a** remains chemically stable under the optimized reaction conditions (Figure 3e, see the SI for the distribution of side products). To confirm our product inhibition hypothesis and simultaneously test for catalyst deactivation, we conducted “same excess” experiments that were designed to mimic the standard reaction after 40 min (55% conversion of **1a**).^75^ In the absence of product **2a**, the reaction profile deviates from that of the standard conditions, which is consistent with product inhibition and/or catalyst degradation (Figure 3f, pink trace). In contrast, when performing the same experiment in the presence of the amount of **2a** that would be formed after 40 min (44%, 0.088 M), excellent overlap with the standard reaction profile is observed (Figure 3f, blue trace). This result corroborates that **2a** is an inhibitor for the bromodefluorination of **1a** and demonstrates that no detectable photocatalyst deactivation is present. To determine the order of each reaction constituent, we performed “different excess” experiments by variable time normalization analysis (VTNA), where the concentrations of the individual components were systematically varied in a series of kinetic trials (Figure 3g).^75,76^ A positive order in light source power (0.7, assumed to be proportional to photon flux) in combination with lower orders (≤0.3) for all other reaction constituents suggests photon absorption is rate-determining (see the SI for a detailed discussion on individual component orders and pseudo-monophotonic vs biphotonic dependence).^77–79^ An intermittent illumination experiment further exemplified the critical role of UV light in activating the defluorinative bromination of **1a**, as neither substrate conversion nor product formation was observed during the dark reaction intervals (Figure 3h).

Building upon our experimental understanding of the individual mechanistic events driving bromodefluorination, we next sought to evaluate the energetic viability of this transformation by computing the reaction free energy profile using density functional theory (DFT), employing **1a** as the model substrate (Figure 3i). UV photoexcitation of **PC1** first generates singlet reductant *PC1, which facilitates single-electron transfer (SET) to **1a**, forming radical anion **i1** and catalyst radical cation **PC1** ^**•+**^ in a highly exergonic step (Δ*G* = −29.8 kcal/mol). Serving as a Lewis acid, LiBr then coordinates reduced substrate **i1** to produce an adduct that is predisposed to undergo a spin-center shift. This rearrangement leads to radical translocation with parallel fluoride elimination through a low-energy transition state (Δ*G* ^‡^ = +1.6 kcal/mol) to yield radical complex **i2**, which ultimately delivers perfluoroalkyl radical **i3** upon extrusion of LiF. Compelling evidence of perfluoroalkyl radical formation was corroborated experimentally by conducting radical trapping experiments, where **i3** was successfully intercepted with various radical scavengers to give isolable C–C and C–O adducts (see the SI for radical trap experiments). Shifting focus toward bromide oxidation, the previously generated **PC1** ^**•+**^ is able to absorb another UV photon to furnish excited ion radical *PC1 ^**•+**^.^33^ Downhill single-electron oxidation of bromide by *PC1 ^**•+**^ then occurs to introduce a bromine radical while regenerating the neutral, ground-state photocatalyst **PC1** (Δ*G* = −60.4 kcal/mol). Given the very short excited-state lifetime of *PC1 ^**•+**^ (τ = 22.2 ps), it is likely that bromide oxidation occurs from a preassociated photocatalyst radical ion pair (see the SI for transient absorption spectroscopy experiments).^80^ Conversely, pathways leading to the oxidation of bromide by ground-state **PC1** ^**•+**^ (Δ*G* = +18.3 kcal/mol) or **PC1** ^**2+**^ (Δ*G* = +26.7 kcal/mol) are thermodynamically demanding and hence less likely contributors for productive bromodefluorination (see the SI for a detailed discussion of all possible oxidation routes). The open-shell intermediate **i3** and Br radical can combine barrierlessly in the singlet state to afford the net halogen exchange product **2a**, in which case we expect diffusion-controlled kinetics. If the complex is formed in the triplet state, the minimum energy crossing point (MECP), required for crossing to the singlet state, has a barrier of +7.8 kcal/mol. This is the upper limit for radical recombination, and is still extremely facile. Alternatively, bromine radicals are known to be stabilized by excess bromide in solution to produce Br_2_^•–^, which is also a capable radical brominating agent.^81^ Taken altogether, our experimental and theoretical observations support our mechanistic hypothesis of bromide oxidation occurring through conPET, where the photocatalyst can behave as a strong excited-state oxidant and reductant in succession.

A variety of perfluoroalkyl compounds underwent bromodefluorination when subjected to the optimized photocatalytic reaction conditions (Figure 4). Fluorinated alkylamides containing perfluoroalkyl­(ether) chains of length C_2_–C_8_, including derivatives of known perfluorocarboxylic acid contaminants, were efficiently transformed into their brominated analogues typically isolated in high yields (71–87%, **2a**–**i**). Brominated products bearing ester, amide, sulfonamide, and sulfonyl *N*-aryl moieties were isolated in moderate to good yields when using **PC1** as the photosensitizer (41–84%, **2j**–**m**). In addition to perfluoroalkylamide substrates possessing fluorinated isosteres, heterocyclic and disubstituted *N*-aryl groups were tolerated, giving rise to halogen metathesis products **2n**–**t** (48–72%; 59% and 60% ^19^F NMR yields for **2q** and **2s** respectively).

**Figure 4:**
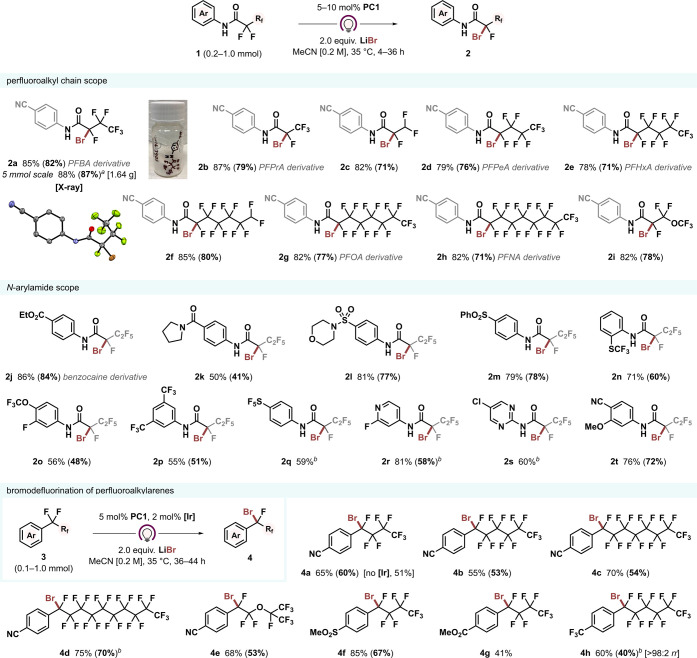
Scope of the controlled bromodefluorination of perfluoroalkyl compounds. See the Supporting Information for details on each individual example. Reaction yields were determined by quantitative ^19^F NMR using 1-fluoronapthalene as an internal standard and are given in brackets for isolated materials. **[Ir]** = [Ir­(dFCF_3_ppy)_2_(dtbpy)]­PF_6_. ^*a*^[0.4 M] MeCN instead of [0.2 M]. ^*b*^ **PC2** instead of **PC1**.

Minor adjustments to the reaction conditions enabled adaptation of the bromodefluorination protocol to include perfluoroalkylarene substrates. Coupled with longer reaction times, the addition of Ir­(dFCF_3_ppy)_2_(dtbpy) as a cocatalyst gave **4a** isolated in 60% yield. *p*-Cyanoarenes featuring longer perfluoroalkyl­(ether) chains were successfully brominated in 53–70% yield (**4b**–**e**). Other electron-poor arene derivatives incorporating sulfonyl and ester groups underwent smooth bromination (**4f**, 67%, **4g**, 41% ^19^F NMR yield), while trifluoromethyl arene product **4h** was furnished in 40% yield with high selectivity for perfluoroalkyl chain functionalization (>98:2 *rr*). Electron-rich perfluoroalkylamides and -arenes exhibited diminished reactivity under the standard conditions (typically <30% ^19^F NMR yield, see the SI for less successful examples **2u**–**ab**, **4i**–**l**), consistent with a lower propensity for photoreduction as a result of their decreased potentials (*E* ^red^ < −2.0 V vs SCE in MeCN, see the SI for a comparison of substrate reduction potentials). Bromodefluorination of electron-poor amide **1aa** bearing a trifluoromethyl (CF_3_) group resulted in no substrate conversion, which may result from the reduced stability of the primary radical that would be generated after defluorination (see the SI for a computed reaction energy profile).^82,83^ Nevertheless, this defluorinative bromination strategy provides access to perfluoroalkyl derivatives that cannot be obtained through previously reported synthetic methodologies.

In an effort to adapt our bromodefluorination methodology to encompass other halide sources, we next investigated defluorinative chlorination of **1a** using LiCl (*E* ^ox^ 1.22 V vs SCE in MeCN).^64^ Full substrate conversion was not observed under the standard conditions from Figure 2, where chlorinated product **5a** was delivered only in moderate ^19^F NMR yield (37%) with **PC1** in employ (Figure 5a, entry 1). In screening photocatalysts with improved optical absorption, the combination of bisphenothiazine catalyst **PC2** with UV (370 nm) and green (525 nm) light, alongside the addition of 3Å molecular sieves, led to nearly full consumption of **1a** while furnishing **5a** in good yield (70%, entry 2). Notably, the amount of product generated is nearly halved when **PC1** is used in place of **PC2** (entry 3) or when the green LED is removed (entry 4), demonstrating the direct response of the bisphenothiazine photocatalyst to green light. With only a slight decrease in yield, the reaction remains efficient when 3Å molecular sieves are omitted (entry 5) but performs poorly when water is added (entry 6). Given the hygroscopicity of LiCl, we suspect that the sieves primarily act as water scavengers.^84,85^ Similarly to defluorinative bromination, lithium remains the optimal halide counterion for chlorodefluorination, as using chloride salts other than LiCl or electrophilic chlorine oxidants promoted little to no formation of **5a** (entries 7–9, see the SI for additional optimization studies).

**Figure 5:**
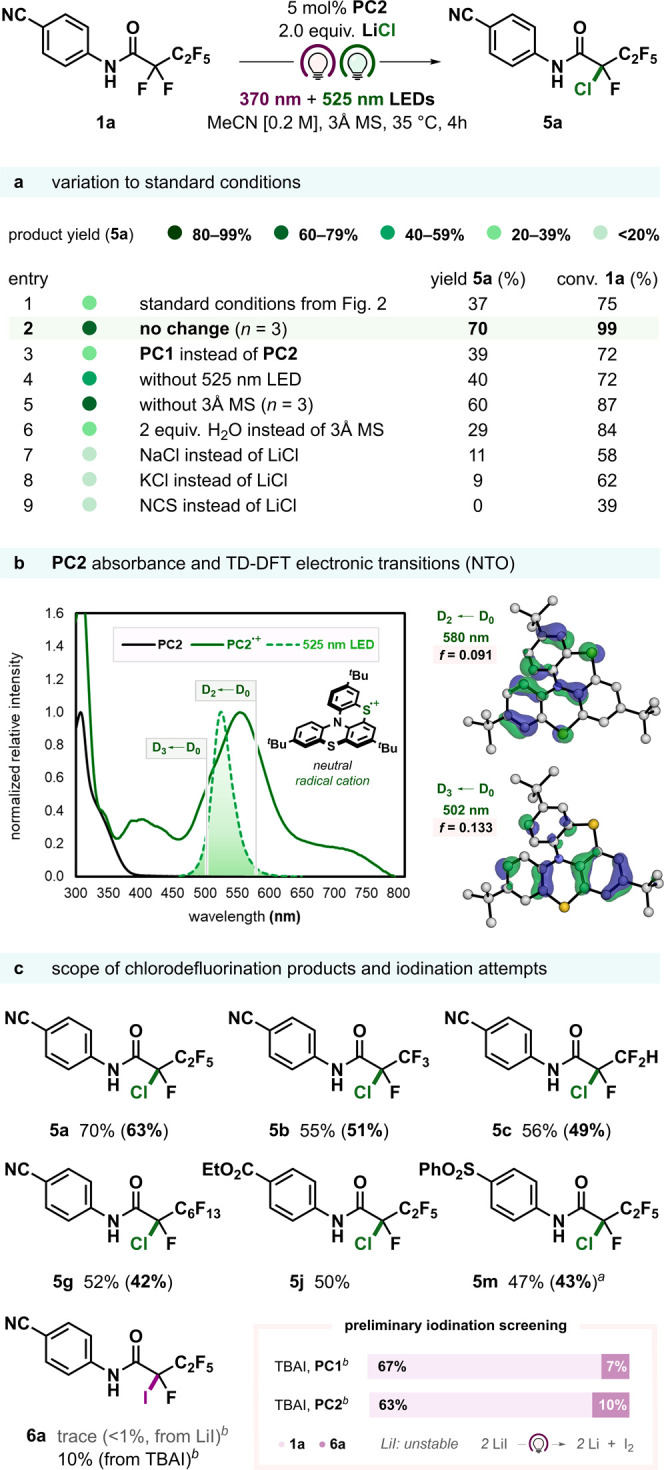
Defluorinative halogenation of perfluoroalkylamides using other halide sources. (a) Reaction optimization for chlorodefluorination of **1a** and variations to the standard conditions. (b) UV–vis absorption profile of **PC2** and **PC2** ^**•+**^ against the 525 nm LED emission profile, alongside natural transition orbitals (NTO) dictating optical transitions for **PC2** ^**•+**^. (c) Defluorinative chlorination scope and investigation of iododefluorination. See the Supporting Information for details on each individual example. Reaction yields were determined by quantitative ^19^F NMR using 1-fluoronapthalene as an internal standard and are given in brackets for isolated materials. ^*a*^10% **PC2** instead of 5%. ^*b*^Without 3Å molecular sieves.

Upon probing the absorption properties of **PC2** in MeCN, we observed the anticipated UV absorption bands from its neutral state (300–390 nm), while electrochemically generated radical cation **PC2** ^**•+**^ displayed a large band in the green region (450–650 nm, Figure 5b). TD-DFT calculations of natural transition orbitals (NTO) revealed two primary transitions at 580 nm (D_2_ ← D_0_, *f* = 0.091) and 502 nm (D_3_ ← D_0_, *f* = 0.133) which both overlap with the emission profile of our green LED source, indicating that excitation of **PC2** ^**•+**^ is feasible. Although decent product yields are still obtained with UV light alone, the incorporation of a green LED substantially improves both yield and conversion which not only enables facile product purification but also suggests that chloride oxidation can proceed through a multiphoton absorption mechanism.^28^

Photocatalytic chlorodefluorination was conducted on a representative group of perfluoroalkylamides to establish proof of concept. Substrates bearing perfluoroalkyl chains of varying lengths (C_2_–C_7_) were efficiently chlorinated under multiwavelength irradiation using **PC2**, affording the corresponding products **5a**–**c** and **5g** isolated in 42–63% yield (Figure 5c). Diversifying the *N*-aryl moiety of the perfluoroalkylamide facilitated chlorination of substrates bearing ester and sulfonyl groups (**5j**, 50% ^19^F NMR yield; **5m**, 43% isolated yield). Although the chlorinated products eventually degrade under the optimized conditions, reaction yields remain comparable to bromodefluorination (see the SI for side product distribution). We finally tested the iodination of perfluoroalkylamides (Figure 5c). Considering LiI decomposes into elemental I_2_ under UV irradiation,^86,87^ iodinated product **6a** was only detected in trace amounts under our standard single- or dual-wavelength photoredox conditions, as expected. Encouragingly, **6a** can be obtained when using the more photostable tetra-*n*-butylammonium iodide (TBAI)^88,89^ instead of LiI, although starting material conversion and product yield remains low (10%, see the SI for preliminary iodination screening).

## Conclusions

In summary, we have demonstrated that *N*-arylphenothiazines catalyze highly site-selective monohalodefluorination of perfluoroalkyl compounds using halide salts. The redox flexibility of these photocatalysts allows them to act as potent reductants and oxidants in sequence under oxidative conPET conditions, offering a modular platform for precise C–F functionalization. Systematic tuning of the reaction parameters and photocatalyst structure enabled net halogen exchange through single- or multiwavelength irradiation, where perfluorinated substrates could be converted into their brominated or chlorinated analogues in good yields. Spectroscopic, kinetic, and computational studies support formation of a transient *N*-arylphenothiazine radical cation superoxidant, which mediates single-electron halide oxidation. These findings are anticipated to open new avenues for targeted conversion of fluorochemical contaminants, and the further development of mechanistically guided, multiphoton-driven defluorinative transformations.

## Supplementary Material



## Notes

Experimental procedures, analytical and physical data, and computational details supporting this manuscript (PDF)
