# Supplementary material for: Photocatalytic Controlled Halodefluorination of Perfluoroalkyl Compounds Using N‑Arylphenothiazines
Source: J Am Chem Soc. 2026 Jul 2;148(27):29084–93. doi: 10.1021/jacs.6c07703 (PMC13383711; doi:10.1021/jacs.6c07703)
Supplement: Supplementary file 1 [file ja6c07703_si_001.pdf]

Supporting Information for

Photocatalytic Controlled Halodefluorination of Perfluoroalkyl Compounds Using *N*-Arylphenothiazines

Michael G. J. Doyle,[1]* Maria Elgaard Jespersen,[1] Shana Noureen,[1] Zijun Chen,[1] Theodore L. Jefferson,[1] Caitlin A. Baptista,[1] Marie Houot,[2] Job J. C. Struijs,[1] Christopher A. Goult,[1] Artem A. Bakulin,[2] Robert S. Paton,[3] Alyssa-Jennifer Avestro,[1]* and Véronique Gouverneur[1]*

[1]Department of Chemistry, Chemistry Research Laboratory, University of Oxford, Oxford OX1 3TA, United Kingdom
[2]Department of Chemistry and Centre for Processable Electronics, Imperial College London, London W12 0BZ, United Kingdom
[3]Department of Chemistry, Colorado State University, Fort Collins, Colorado 80528, United States

*Corresponding Author. Email:
veronique.gouverneur@chem.ox.ac.uk
alyssa-jennifer.avestro@chem.ox.ac.uk
michael.doyle@chem.ox.ac.uk

Contents

**1**	**General Considerations**	**S–1**
**2**	**Photocatalyst Synthesis**	**S–3**
**3**	**General Procedures for Halodefluorination**	**S–4**
**4**	**Product Characterization Data & Experimental Details**	**S–6**
**5**	**Starting Material Synthesis & Characterization Data**	**S–20**
**6**	**Additional Reaction Optimization Data**	**S–33**
**7**	**Less Successful Scope Examples**	**S–37**
**8**	**Reaction Kinetics & Mechanistic Experiments**	**S–38**
**9**	**Absorption Spectroscopy (UV-Vis, Spectroelectrochemistry)**	**S–50**
**10**	**Emission Spectroscopy (Steady-State, Time-Resolved)**	**S–54**
**11**	**Electrochemistry**	**S–62**
**12**	**Excited-State Redox Potential Calculations**	**S–70**
**13**	**Femtosecond Transient Absorption Spectroscopy**	**S–72**
**14**	**Computational Analysis**	**S–74**
**15**	**X-Ray Crystallography**	**S–94**
**16**	**References**	**S–95**
**17**	**NMR Spectra**	**S–98**

1 General Considerations

Reactions, Reagents & Supplies: unless noted, all reactions were conducted under an inert atmosphere employing standard Schlenk techniques using anhydrous solvents. All glassware was flame-dried and subsequently cooled under vacuum prior to use. Flash column chromatography was performed using Millipore Geduran® Si 60 silica gel (40–63 µm particle size, 60Å pore size, Merck). Analytical thin-layer chromatography (TLC) was performed using aluminum plates pre-coated with silica (Supelco TLC Silica gel 60 F_{254} – aluminum-backed, Merck). TLC plates were visualized under UV light (short λ = 254 nm, or long λ = 365 nm) or by staining with potassium permanganate or phosphomolybdic acid (PMA) stains. Unless otherwise noted, all reagents were obtained from commercial vendors (Fluorochem, Apollo Scientific, Manchester Organics, BLDpharm, Merck, Sigma-Aldrich, Fisher Scientific) and used as supplied. LiBr (≥99.995% trace metals basis) was purchased from Merck and dried under vacuum (<300 mTorr) at 150 °C overnight prior to each use. LiCl (99.0%) was purchased from Fluorochem and dried under vacuum (<300 mTorr) at 150 °C overnight prior to each use. Anhydrous MeCN (99.9%, extra dry over molecular sieves) was purchased from Thermo Fisher Scientific and used as supplied. Other solvents were obtained from commercial vendors and dried/deoxygenated using a solvent purification system (MBraun SPS 5). $Ir[dFCF_3ppy]_2(dtbpy)PF_6$ was purchased from BLDpharm and used as supplied. LED lamps (PR160L-370 nm Gen 2 44W, PR160L-390 nm 52W, PR160L-456 nm 50W, PR160L-525 nm 44W) were purchased from Kessil®. A high velocity cooling fan (Schallen, 14 in.) was purchased from Amazon.

NMR Spectroscopy: NMR spectra (^1H, ^{19}F, ^{13}C) were obtained on a Bruker AVIII HD 600 MHz, Bruker NEO 600 MHz, Bruker AVIII HD 500 MHz, or Bruker AVIII HD 400 MHz spectrometer. The chemical shifts are given as parts per million (ppm) and were referenced to the residual solvent signal [($CDCl_3$: δH = 7.26 ppm, δC = 77.16 ppm), (MeCN-d_3: δH = 1.94 ppm, δC = 1.32 ppm), (DMSO-d_6: δH = 2.50 ppm, δC = 39.50 ppm), (Acetone-d_6: δH = 2.05 ppm, δC = 29.84 ppm)].[1] ^{19}F NMR spectra were referenced externally relative to $CFCl_3$. For $^{13}C\{^1H,^{19}F\}$ experiments, only partial $^{13}C\{^{19}F\}$ decoupling was observed in most cases; this is stated for the relevant compounds. NMR spectra were processed with MestReNova v15.0.1. Peak multiplicities are described as follows: s (singlet), d (doublet), t (triplet), q (quartet), m (multiplet), or br (broad). Unless otherwise noted, quantitative ^{19}F NMR yields were determined from crude reaction mixtures using 1-fluoronaphthalene (FNP) as an internal standard. FNP is not stable under the photochemical reaction conditions and was therefore added to the crude reaction samples directly. ^1H NMR yields were determined from crude reaction mixtures using 1,3,5-trimethoxybenzene (TMB) as an internal standard. TMB is not stable under the photochemical reaction conditions and was therefore added to the crude reaction samples directly.

Mass Spectrometry: HRMS analyses of the products were performed on a Waters BioAccord LC-MS system (ESI) in positive or negative ionization mode or on an Agilent 7200 Accurate Mass Q-TOF GC-MS connected to an Agilent 7890 GC system (EI, CI) in positive or negative ionization mode. Some compounds did not ionize under a variety of MS ionization methods (ESI, EI, CI, APCI, MALDI) using the aforementioned instruments, and therefore no HRMS spectra could be obtained for them. In some of these cases, low resolution data (LRMS) were collected using an Agilent 5977B GC/MSD (EI) in positive ionization mode. Compounds **3d**, **4d**, and **4g** did not ionize when measured by HRMS or LRMS, and therefore no mass spectra could be obtained.

Absorption Spectroscopy: steady-state absorption spectra were recorded on an Agilent Technologies Cary 5000 UV-Vis-NIR spectrophotometer using a standard quartz cuvette (l = 10 mm) at room temperature. Spectroelectrochemistry (SEC) experiments were performed using a Gamry Reference 3000 potentiostat interfaced to a PC. Measurements were done at room temperature using an optically transparent thin-layer electrochemical cell (OTTLE, l ≈ 0.2 mm, with two CaF_2 windows separated by PTFE spacers) fitted with a Pt wire mesh working electrode, Pt wire counter electrode, and an Ag wire pseudo-reference electrode. All SEC samples were prepared as N_2-purged solutions in anhydrous MeCN containing [0.1 M] tetra-*n*-butylammonium hexafluorophosphate ($TBAPF_6$) as the supporting electrolyte and analyzed under a constant applied voltage.

Emission Spectroscopy: LED emission data (PR160L-370 nm Gen 2 44W, PR160L-525 nm 44W) were graciously provided by Kessil. Steady-state fluorescence spectra were recorded on an Agilent Technologies Cary Eclipse Fluorescence Spectrophotometer (Cary WinFLR software) equipped with a xenon lamp, using a standard quartz cuvette (l = 10 mm) at room temperature. Time-resolved emission spectra were recorded on an Edinburgh Instruments FS5 Spectrofluorometer (time-correlated single photon counting, TCSPC) at room temperature using a 365 nm picosecond pulsed diode laser (EPLED-365, λ_{exc} = 365 nm, 850 ps pulse width). Fluorescence lifetime values were calculated using the tail fit method. The instrument response function (IRF) was recorded independently for a colloidal suspension of alumina (0.5 μM) in H_2O. Unless otherwise noted, all samples were prepared in anhydrous, degassed MeCN and sparged with N_2 before measurement.

Electrochemistry: cyclic voltammetry was performed using a Gamry Reference 3000 potentiostat interfaced to a PC. Samples were prepared in anhydrous MeCN, purged with N_2, and recorded at room temperature using [1.0 M] tetrabutylammonium hexafluorophosphate ($TBAPF_6$) as the supporting electrolyte. All solution-state electrochemical experiments were performed using a glassy carbon working electrode (BASi; 0.071 cm^2). The electrode surface was polished with a 0.05 μM alumina-water slurry on a felt surface immediately before each use. The reference electrode was an Ag/AgCl (sat. KCl) aqueous electrode, with Pt wire used as the counter electrode. Redox potentials were converted to the saturated calomel electrode (SCE) by subtracting 0.044 V from the values obtained vs. Ag/AgCl.[2]

Powder X-Ray Diffraction: powder x-ray diffraction (PXRD) data were collected using a Malvern Panalytical X'pert Alpha diffractometer; the radiations Cu Kα1,2 were used (λ = 1.54056, 1.54439 Å, Kα1:Kα2 2:1). The diffractometer was operated with an accelerating voltage of 45 kV and a current of 40 mA. Powder samples were adhered onto glass microscope slides using Molykote silicon grease. Diffractograms were analyzed in Malvern Panalytical Xpert Highscore Plus (version: 5.0.0.28712).

X-Ray Crystallography: solid-state data were collected using an Oxford Diffraction/Agilent SuperNova. Crystals were selected under Fomblin perfluoro polyether oil, mounted on MiTeGen micromount loops, and quench-cooled using an Oxford Cryosystems open flow N_2 cooling device.[3] Data were processed using the CrysAlisPro package,[4] including unit cell parameter refinement and inter-frame scaling. Equivalent reflections were merged and corrected; crystal faces were indexed and used to apply an absorption and illuminated volume correction with the CrysAlisPro suite. Structures were solved *ab initio* from the integrated intensities using SuperFlip[5] and refined using full-matrix least-squares on F2 using Crystals.[6] Hydrogen atoms were included in the refinement with soft restraints.[7]

2 Photocatalyst Synthesis

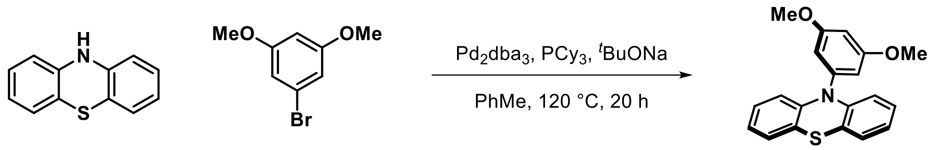

PC1 Adapted from a literature procedure.[8] To a 50 mL round bottom flask charged with a stir bar was sequentially added phenothiazine (598 mg, 3.0 mmol, 1.0 equiv.), 1-bromo-3,5-dimethoxybenzene (977 mg, 4.5 mmol, 1.5 equiv.), NaO^{t}Bu (721 mg, 7.5 mmol, 2.5 equiv.), Pd_2dba_3 (137 mg, 0.15 mmol, 0.05 equiv.), and PCy_3 (59 mg, 0.21 mmol, 0.07 equiv.). The flask was fit with a reflux condenser and subsequently put under an N_2 atmosphere. This was followed by the addition of anhydrous toluene (12 mL). The reaction mixture was then heated to 120 °C and stirred for 20 h. Upon completion of the reaction as monitored by TLC or ^{1}H NMR, the reaction mixture was cooled to rt and quenched with H_2O (30 mL). The layers were separated, and the aqueous layer was extracted with EtOAc (3 × 50 mL). The organics were combined and subsequently washed with brine (1 × 50 mL). The combined organics were then dried over Na_2SO_4, filtered, and concentrated *in vacuo*. Isolated in 95% yield (958 mg) as a white solid after purification by silica gel chromatography (5% EtOAc in Hexanes, dry load).

^{1}H NMR ($CDCl_3$, 500 MHz) δ 7.05 – 7.01 (m, 2H), 6.92 – 6.87 (m, 2H), 6.85 – 6.80 (m, 2H), 6.59 – 6.54 (m, 3H), 6.42 – 6.38 (m, 2H), 3.82 (s, 6H);
^{13}C NMR ($CDCl_3$, 126 MHz) δ 162.6, 143.9, 142.8, 127.0, 126.8, 122.7, 120.4, 116.4, 108.0, 100.5, 55.7;
HRMS (ESI): calcd. for $C_{20}H_{18}NO_2S$ $[M+H]^+$: 336.1053. Found 336.1061.

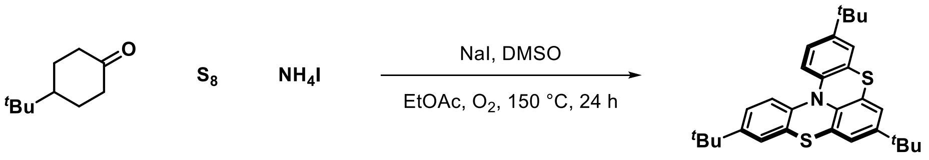

PC2 Adapted from a literature procedure.[9–10] To a 60 mL pressure tube charged with a stir bar was sequentially added S_8 (769 mg, 3.0 mmol, 1.0 equiv.), NH_4I (1.09 g, 7.5 mmol, 2.5 equiv.), NaI (135 mg, 0.9 mmol, 0.3 equiv.), 4-*tert*-butylcyclohexanone (1.39 g, 9.0 mmol, 3.0 equiv.), DMSO (469 mg, 6.0 mmol, 2.0 equiv.), and EtOAc (9.0 mL). After sparging the suspension with O_2 for 5 min, the tube was sealed and subsequently stirred at 150 °C for 24 h. Upon completion of the reaction as monitored by TLC or ^{1}H NMR, the reaction mixture was cooled to rt, filtered through a celite pad, and concentrated *in vacuo*. Isolated in 32% yield (448 mg) as a yellow solid after purification by silica gel chromatography (0 → 20% EtOAc in Hexanes, dry load). The NMR data is in accordance with the literature.[10]

Note: *this photocatalyst is mildly light-sensitive. New batches were synthesized monthly, and the material was stored in the dark under argon.*

^{1}H NMR ($CDCl_3$, 500 MHz) δ 7.20 (s, 2H), 7.14–7.12 (m, 4H), 6.98 (s, 2H), 1.29 (s, 18H), 1.24 (s, 9H);
^{13}C NMR ($CDCl_3$, 126 MHz) δ 148.1, 147.7, 140.1, 137.1, 126.0, 125.1, 124.7, 124.5, 122.7, 119.9, 34.5, 34.4, 31.3, 31.2.

3 General Procedures for Halodefluorination

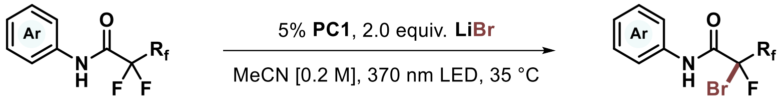

General Procedure A (Bromodefluorination, Perfluoroalkylamides): perfluoroalkylamide (0.40 mmol, 1.0 equiv.), **PC1** (6.7 mg, 0.02 mmol, 0.05 equiv.), LiBr (69.5 mg, 0.80 mmol, 2.0 equiv.), and anhydrous MeCN (2.0 mL) were added sequentially to a dried 1-dram vial charged with a stir bar. The vial was sealed with a PTFE-lined cap, and the solution was subsequently frozen in an acetone/CO_2 cooling bath (−78 °C). The reaction headspace was then evacuated and backfilled with N_2 on a Schlenk line while maintaining the cooling bath temperature at −78 °C. After the final purging cycle, the reaction mixture was allowed to warm to room temperature. The vial cap was then sealed with parafilm and electrical tape. The reaction was placed ~3 cm away from an LED lamp (Kessil PR160L-370 nm Gen 2 44W) and irradiated (intensity setting: 100) under fan cooling with vigorous stirring. Upon completion of the reaction (4–36 h) as determined by ^{19}F NMR, the reaction mixture was diluted with EtOAc (30 mL) and filtered through a celite pad. The crude organics were dried over Na_2SO_4, filtered, concentrated *in vacuo*, and purified by silica gel chromatography.

Notes: *LiBr is very hygroscopic, thus it is ideal to weigh it quickly. For the two light setup, the vial was placed in between two LED lamps which measured 6.5 cm apart from each other. Vial temperatures reached ~35 °C using these photochemical setups. See the following page for a schematic of the different photochemical setups. For reaction scales >0.40 mmol, use a 4- or 8-dram vial under the two light setup.*

Reaction Monitoring by ^{19}F NMR: before quenching the reaction, substrate conversion and product yield were determined by removing a small aliquot (40 μL) of the reaction and placing it into 0.60 mL $CDCl_3$ for calibrated ^{19}F NMR analysis using FNP as the reference signal. This was done by preparing a stock solution of FNP (27.1 mg) in $CDCl_3$ (1.0 mL) and adding a 40 μL aliquot of this solution to the $CDCl_3$ solution containing the reaction aliquot (corresponding to a 1:1 integration between starting material or product (1 fluorine) and the FNP internal standard signal at −123.5 ppm). FNP must be added directly to the reaction aliquot, as it is not stable under the optimized reaction conditions.

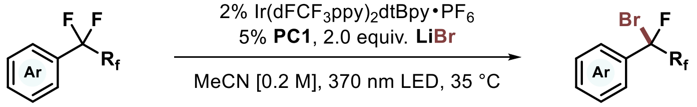

General Procedure B (Bromodefluorination, Perfluoroalkylarenes): perfluoroalkyl arene (0.40 mmol, 1.0 equiv.), Ir[dFCF_3ppy]$_2$(dtbpy)PF_6 (9.0 mg, 0.008 mmol, 0.02 equiv.), **PC1** (6.7 mg, 0.02 mmol, 0.05 equiv.), LiBr (69.5 mg, 0.80 mmol, 2.0 equiv.), and anhydrous MeCN (2.0 mL) were added sequentially to a dried 1-dram vial charged with a stir bar. The remainder of the procedure is identical to General Procedure A.

Note: *upon completion of the reaction as determined by ^{19}F NMR (36–44 h), the reaction mixture was diluted with $CHCl_3$ (30 mL) instead of EtOAc due to improved solubility.*

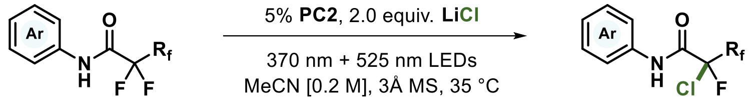

General Procedure C (Chlorodefluorination, Perfluoroalkylamides): perfluoroalkylamide (0.10 mmol, 1.0 equiv.), **PC2** (2.4 mg, 0.005 mmol, 0.05 equiv.), activated 3Å molecular sieves (×2), LiCl (8.5 mg, 0.20 mmol, 2.0 equiv.), and anhydrous MeCN (0.5 mL) were added sequentially to a dried ½-dram vial charged with a stir bar. The vial was sealed with a PTFE-lined cap, and the solution was subsequently frozen in an acetone/CO_2 cooling bath (−78 °C). The reaction headspace was then evacuated and backfilled with N_2 on a Schlenk line while maintaining the cooling bath temperature at −78 °C. After the final purging cycle, the reaction mixture was allowed to warm to room temperature. The vial cap was then sealed with parafilm and electrical tape. The reaction was placed ~3 cm away from each of ***two*** LED lamps (Kessil PR160L-370 nm Gen 2 44W, PR160L-525 nm 44W) and irradiated (intensity setting: 100) under fan cooling with vigorous stirring. The remainder of the procedure is identical to General Procedure A.

Note: *the performance of **PC2** declines after 3–4 weeks due to mild photosensitivity, thus it was synthesized monthly and subsequently stored in the dark under argon.*

Photochemical Reaction Setup

Note: *reaction temperatures reach ~°35 C using these photochemical setups. Without fan cooling, the temperature rises to ~°40 C.*

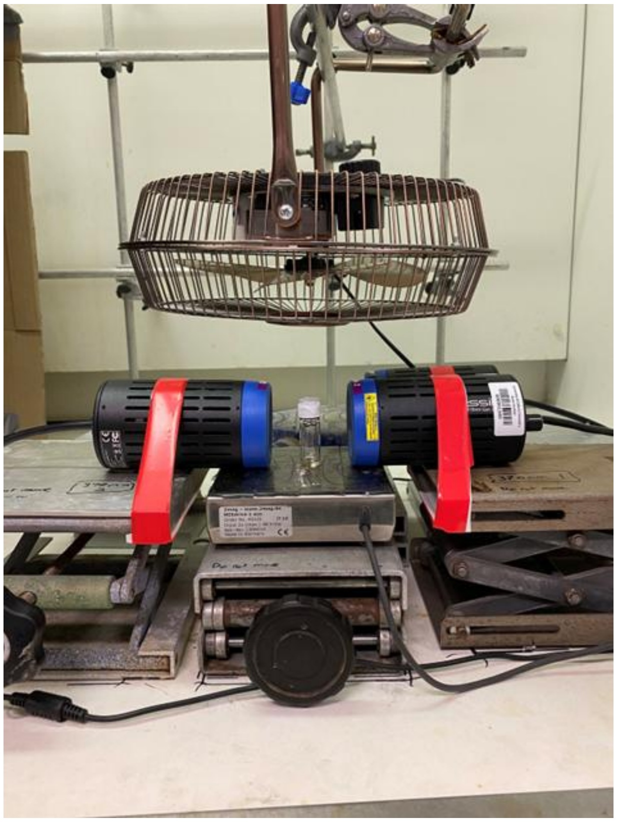

Two Light Setup (either 2 × 370 nm, or 1 × 370 nm + 1 × 525 nm)

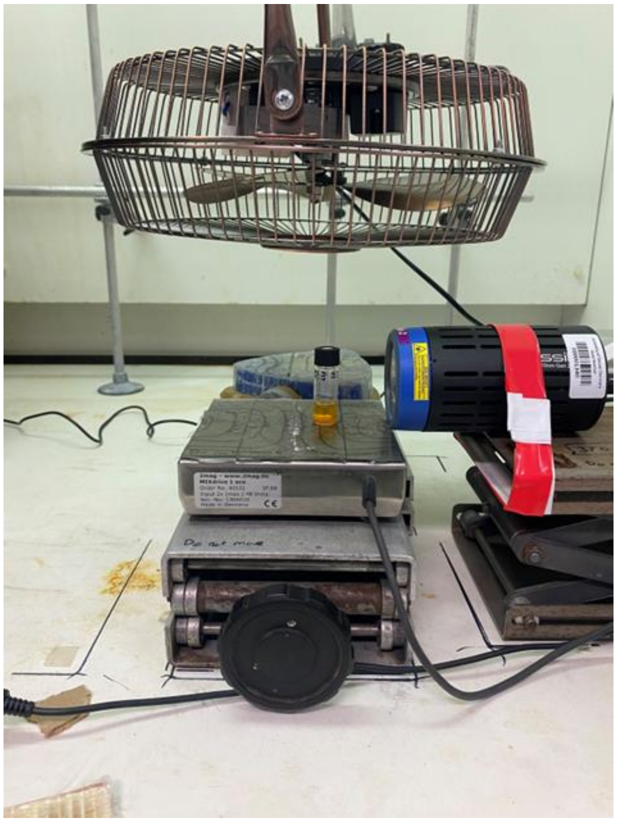

One Light Setup

4 Product Characterization Data & Experimental Details

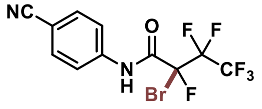

2a Prepared according to General Procedure A in a 1-dram vial from substrate **1a** (125.7 mg, 0.40 mmol, 1.0 equiv.), photocatalyst **PC1** (6.7 mg, 0.02 mmol, 0.05 equiv.), and LiBr (69.5 mg, 0.80 mmol, 2.0 equiv.) in anhydrous MeCN (2.0 mL). The reaction mixture was allowed to stir for 4 h under UV irradiation (370 nm) using the one light setup. ^{19}F NMR yield: 85%. Isolated in 82% yield (122.4 mg) as a white solid after purification by silica gel chromatography (15% EtOAc in hexanes, dry load).

Through 15 independent experiments conducted over the course of the project, the average crude ^{19}F NMR yield was 86% (σ = 1.9).

2a was also prepared on a larger scale. Prepared according to General Procedure A in a 4-dram vial from substrate **1a** (1.57 g, 5.0 mmol, 1.0 equiv.), photocatalyst **PC1** (84.0 mg, 0.25 mmol, 0.05 equiv.), and LiBr (869 mg, 10.0 mmol, 2.0 equiv.) in anhydrous MeCN (12.5 mL). The reaction mixture was allowed to stir for 12 h under UV irradiation (370 nm) using the two light setup. ^{19}F NMR yield: 88%. Isolated in 87% yield (1.64 g) as a white solid after purification by silica gel chromatography (15% EtOAc in hexanes, dry load).

Crystals were grown for x-ray crystallography using the solvent layering method. In a 2-dram vial, 100 mg **2a** was dissolved in ~1 mL $CHCl_3$ to make a concentrated solution. Pentane (5 mL) was then layered gently on top of the $CHCl_3$ solution, and the vial cap was closed. The vial was allowed to sit undisturbed at room temperature for 24 h, after which thick, block-like crystals were present in the $CHCl_3$ layer.

^{1}H NMR ($CDCl_3$, 500 MHz) δ 8.24 (br s, 1H), 7.77 – 7.67 (m, 4H);
^{19}F NMR ($CDCl_3$, 470 MHz) δ −78.5 (d, *J* = 11.5 Hz, 3F), −115.4 (dd, *J* = 281.9, 8.9 Hz, 1F), −117.3 (dd, *J* = 282.1, 12.0 Hz, 1F), −132.5 – −132.7 (m, 1F);
^{13}C{^{1}H,^{19}F} NMR ($CDCl_3$, 126 MHz, partial ^{13}C{^{19}F} decoupling observed) δ 158.5, 139.5, 133.7, 120.8, 118.3, 116.6 – 116.3 (m), 109.8, 109.5 – 109.2 (m), 93.3;
HRMS (ESI): calcd. for $C_{11}H_4[^{79}Br]F_6N_2O$ $[M-H]^-$: 372.9417. Found 372.9410.

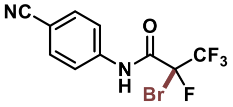

2b Prepared according to General Procedure A in a 4-dram vial from substrate **1b** (264.2 mg, 1.0 mmol, 1.0 equiv.), photocatalyst **PC1** (16.8 mg, 0.05 mmol, 0.05 equiv.), and LiBr (173.7 mg, 2.0 mmol, 2.0 equiv.) in anhydrous MeCN (5.0 mL). The reaction mixture was allowed to stir for 16 h under UV irradiation (370 nm) using the two light setup. ^{19}F NMR yield: 87%. Isolated in 79% yield (257.0 mg) as a white solid after purification by silica gel chromatography (15% EtOAc in hexanes, dry load).

^{1}H NMR ($CDCl_3$, 500 MHz) δ 8.31 (br s, 1H), 7.78 – 7.73 (m, 2H), 7.72 – 7.67 (m, 2H);
^{19}F NMR ($CDCl_3$, 470 MHz) δ −77.3 (d, *J* = 8.7 Hz, 3F), −134.4 (q, *J* = 8.7 Hz, 1F);
^{13}C{^{1}H,^{19}F} NMR ($CDCl_3$, 126 MHz, partial ^{13}C{^{19}F} decoupling observed) δ 158.5, 139.6, 133.7, 120.7, 119.0 – 118.6 (m), 118.3, 109.6, 92.5 – 90.6 (m);
HRMS (ESI): calcd. for $C_{10}H_4[^{79}Br]F_4N_2O$ $[M-H]^-$: 322.9449. Found 322.9446.

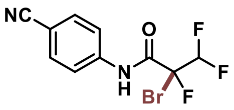

2c Prepared according to General Procedure A in a 1-dram vial from substrate **1c** (98.5 mg, 0.40 mmol, 1.0 equiv.), photocatalyst **PC1** (6.7 mg, 0.02 mmol, 0.05 equiv.), and LiBr (69.5 mg, 0.80 mmol, 2.0 equiv.) in anhydrous MeCN (2.0 mL). The reaction mixture was allowed to stir for 5 h under UV irradiation (370 nm) using the two light setup. ^{19}F NMR yield: 82%. Isolated in 71% yield (86.9 mg) as an off-white solid after purification by silica gel chromatography (15 → 20% EtOAc in hexanes, dry load).

^{1}H NMR (MeCN-d_3, 500 MHz) δ 9.41 (br s, 1H), 7.84 – 7.78 (m, 2H), 7.77 – 7.71 (m, 2H), 6.49 (td, *J* = 52.7, 11.0 Hz, 1H);
^{19}F NMR (MeCN-d_3, 470 MHz) δ −127.9 (ddd, *J* = 279.2, 49.9, 18.3 Hz, 1F), −137.0 (ddd, *J* = 279.1, 49.9, 14.1 Hz, 1F), −145.0 (ddd, *J* = 24.5, 13.8, 10.7 Hz, 1F);
^{13}C{^{1}H,^{19}F} NMR (MeCN-d_3, 126 MHz, partial ^{13}C{^{19}F} decoupling observed) δ 162.4, 141.5, 134.3, 122.0, 119.4, 113.6 – 111.9 (m), 109.6, 95.4 – 92.7 (m);
HRMS (ESI): calcd. for $C_{10}H_5[^{79}Br]F_3N_2O$ [M−H]$^-$: 304.9543. Found 304.9538.

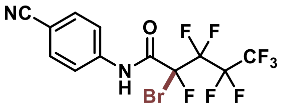

2d Prepared according to General Procedure A in a 1-dram vial from substrate **1d** (145.7 mg, 0.40 mmol, 1.0 equiv.), photocatalyst **PC1** (6.7 mg, 0.02 mmol, 0.05 equiv.), and LiBr (69.5 mg, 0.80 mmol, 2.0 equiv.) in anhydrous MeCN (2.0 mL). The reaction mixture was allowed to stir for 14 h under UV irradiation (370 nm) using the one light setup. ^{19}F NMR yield: 79%. Isolated in 76% yield (137.0 mg) as a beige solid after purification by silica gel chromatography (15% EtOAc in hexanes, dry load).

^{1}H NMR (Acetone-d_6, 500 MHz) δ 10.53 (br s, 1H), 8.00 – 7.95 (m, 2H), 7.86 – 7.82 (m, 2H);
^{19}F NMR (Acetone-d_6, 470 MHz) δ −81.8 – −81.9 (m, 3F), −110.4 – −111.2 (m, 1F), −113.8 – −114.6 (m, 1F), −123.2 (dddd, *J* = 289.7, 11.6, 10.9, 2.2 Hz, 1F), −124.0 (ddd, *J* = 290.2, 19.5, 9.4 Hz, 1F), −133.3 – −133.6 (m, 1F);
^{13}C{^{1}H,^{19}F} NMR (Acetone-d_6, 126 MHz, partial ^{13}C{^{19}F} decoupling observed) δ 160.2, 141.6, 134.2, 122.2, 119.0, 112.2 – 111.9 (m), 110.1 – 109.9 (m), 109.8, 94.3;
HRMS (ESI): calcd. for $C_{12}H_4[^{79}Br]F_8N_2O$ [M−H]$^-$: 422.9385. Found 422.9386.

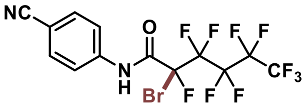

2e Prepared according to General Procedure A in a 1-dram vial from substrate **1e** (124.3 mg, 0.30 mmol, 1.0 equiv.), photocatalyst **PC1** (5.1 mg, 0.015 mmol, 0.05 equiv.), and LiBr (52.1 mg, 0.60 mmol, 2.0 equiv.) in anhydrous MeCN (1.5 mL). The reaction mixture was allowed to stir for 6 h under UV irradiation (370 nm) using the one light setup. ^{19}F NMR yield: 78%. Isolated in 71% yield (100.9 mg) as a white solid after purification by silica gel chromatography (15% EtOAc in hexanes, dry load).

^{1}H NMR (Acetone-d_6, 500 MHz) δ 10.53 (br s, 1H), 8.00 – 7.94 (m, 2H), 7.87 – 7.81 (m, 2H);

^{19}F NMR (Acetone-d_6, 470 MHz) δ −81.6 (tt, J = 10.2, 2.6 Hz, 3F), −109.9 – −110.8 (m, 1F), −113.1 – −114.0 (m, 1F), −119.3 – −120.2 (m, 1F), −120.2 – −121.1 (m, 1F), −126.0 – −127.5 (m, 2F), −133.0 – −133.3 (m, 1F);
^{13}C{^{1}H,^{19}F} NMR (Acetone-d_6, 126 MHz, partial ^{13}C{^{19}F} decoupling observed) δ 160.2, 141.6, 134.1, 122.2, 118.9, 113.0 – 112.2 (m), 111.7, 109.7 – 109.0 (m), 109.8, 94.5;
HRMS (ESI): calcd. for $C_{13}H_4[^{79}Br]F_{10}N_2O$ [M−H]$^-$: 472.9353. Found 472.9357.

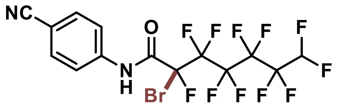

2f Prepared according to General Procedure A in a 1-dram vial from substrate **1f** (178.5 mg, 0.40 mmol, 1.0 equiv.), photocatalyst **PC1** (6.7 mg, 0.02 mmol, 0.05 equiv.), and LiBr (69.5 mg, 0.80 mmol, 2.0 equiv.) in anhydrous MeCN (2.0 mL). The reaction mixture was allowed to stir for 13 h under UV irradiation (370 nm) using the one light setup. ^{19}F NMR yield: 85%. Isolated in 80% yield (162.2 mg) as a white solid after purification by silica gel chromatography (20% EtOAc in hexanes, dry load).

^{1}H NMR ($CDCl_3$, 500 MHz) δ 8.22 (br s, 1H), 7.76 – 7.67 (m, 4H), 6.04 (tt, J = 51.9, 5.0 Hz, 1H);
^{19}F NMR ($CDCl_3$, 470 MHz) δ −109.8 – −110.6 (m, 1F), −112.4 – −113.2 (m, 1F), −118.1 – −118.9 (m, 1F), −119.0 – −119.9 (m, 1F), −122.3 – −123.9 (m, 2F), −129.0 – −129.3 (m, 2F), −131.7 – −131.9 (m, 1F), −136.2 – −137.7 (m, 2F);
^{13}C{^{1}H,^{19}F} NMR ($CDCl_3$, 126 MHz, partial ^{13}C{^{19}F} decoupling observed) δ 158.7, 139.5, 133.7, 120.7, 118.3, 111.8 – 111.3 (m), 110.8, 110.3 – 110.0 (m), 109.7, 107.9 – 107.4 (m), 94.3;
HRMS (ESI): calcd. for $C_{14}H_5[^{79}Br]F_{11}N_2O$ [M−H]$^-$: 504.9415. Found 504.9412.

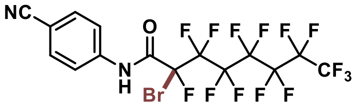

2g Prepared according to General Procedure A in a 1-dram vial from substrate **1g** (154.3 mg, 0.30 mmol, 1.0 equiv.), photocatalyst **PC1** (5.1 mg, 0.015 mmol, 0.05 equiv.), and LiBr (52.1 mg, 0.60 mmol, 2.0 equiv.) in anhydrous MeCN (1.5 mL). The reaction mixture was allowed to stir for 12 h under UV irradiation (370 nm) using the one light setup. ^{19}F NMR yield: 82%. Isolated in 77% yield (132.1 mg) as a white solid after purification by silica gel chromatography (15% EtOAc in hexanes, dry load).

^{1}H NMR (MeCN-d_3, 500 MHz) δ 9.46 (br s, 1H), 7.83 – 7.78 (m, 2H), 7.78 – 7.73 (m, 2H);
^{19}F NMR (MeCN-d_3, 470 MHz) δ −81.5 (tt, J = 10.2, 2.3 Hz, 3F), −109.9 – −110.8 (m, 1F), −113.0 – −113.8 (m, 1F), −118.4 – −119.2 (m, 1F), −119.3 – −120.2 (m, 1F), −122.2 – −122.5 (m, 2F), −122.9 – −123.2 (m, 2F), −126.5 – −126.7 (m, 2F), −132.6 – −132.8 (m, 1F);
^{13}C{^{1}H,^{19}F} NMR (MeCN-d_3, 126 MHz, partial ^{13}C{^{19}F} decoupling observed) δ 160.2, 141.2, 134.4, 122.3, 119.3, 112.9 – 112.4 (m), 112.3, 111.7, 111.2, 109.9, 94.9;
HRMS (ESI): calcd. for $C_{15}H_4[^{79}Br]F_{14}N_2O$ [M−H]$^-$: 572.9289. Found 572.9281.

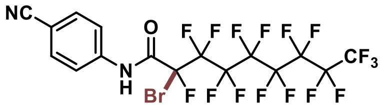

2h Prepared according to General Procedure A in a 1-dram vial from substrate **1h** (112.8 mg, 0.20 mmol, 1.0 equiv.), photocatalyst **PC1** (3.4 mg, 0.01 mmol, 0.05 equiv.), and LiBr (34.8 mg, 0.40 mmol,

2.0 equiv.) in anhydrous MeCN (1.0 mL). The reaction mixture was allowed to stir for 4 h under UV irradiation (370 nm) using the one light setup. ^{19}F NMR yield: 82%. Isolated in 71% yield (89.1 mg) as a white solid after purification by silica gel chromatography (15% EtOAc in hexanes, dry load).

^{1}H NMR (MeCN-d$_3$, 500 MHz) δ 9.47 (br s, 1H), 7.83 – 7.72 (m, 4H);
^{19}F NMR (MeCN-d$_3$, 470 MHz) δ −81.5 (tt, *J* = 10.3, 2.2 Hz, 3F), −109.8 – −110.8 (m, 1F), −113.0 – −113.8 (m, 1F), −118.3 – −119.1 (m, 1F), −119.2 – −120.1 (m, 1F), −122.0 – −122.4 (m, 4F), −123.0 – −123.3 (m, 2F), −126.5 – −126.7 (m, 2F), −132.6 – −132.8 (m, 1F);
^{13}C{^{1}H,^{19}F} NMR (MeCN-d$_3$, 126 MHz, partial ^{13}C{^{19}F} decoupling observed) δ 160.3, 141.3, 134.4, 122.4, 119.3, 112.7 – 112.5 (m), 112.4 – 112.1 (m), 111.9 – 111.7 (m), 111.7, 111.3 – 111.0 (m), 110.0, 94.9;
HRMS (ESI): calcd. for $C_{16}H_4[^{79}Br]F_{16}N_2O$ [M−H]$^-$: 622.9257. Found 622.9258.

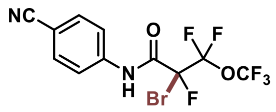

2i Prepared according to General Procedure A in a 1-dram vial from substrate **1i** (66.0 mg, 0.20 mmol, 1.0 equiv.), photocatalyst **PC1** (3.4 mg, 0.01 mmol, 0.05 equiv.), and LiBr (34.8 mg, 0.40 mmol, 2.0 equiv.) in anhydrous MeCN (1.0 mL). The reaction mixture was allowed to stir for 13 h under UV irradiation (370 nm) using the one light setup. ^{19}F NMR yield: 82%. Isolated in 78% yield (61.2 mg) as a white solid after purification by silica gel chromatography (15% EtOAc in hexanes, dry load).

^{1}H NMR (CDCl$_3$, 500 MHz) δ 8.21 (br s, 1H), 7.77 – 7.73 (m, 2H), 7.72 – 7.68 (m, 2H);
^{19}F NMR (CDCl$_3$, 470 MHz) δ −55.1 (t, *J* = 9.3 Hz, 3F), −79.4 – −79.8 (m, 1F), −81.9 – −82.3 (m, 1F), −133.2 – −133.3 (m, 1F);
^{13}C{^{1}H,^{19}F} NMR (CDCl$_3$, 126 MHz, partial ^{13}C{^{19}F} decoupling observed) δ 158.4, 139.6, 133.7, 120.7, 120.6, 118.3, 118.2 – 118.0 (m), 109.7, 92.8 – 92.6 (m);
HRMS (ESI): calcd. for $C_{11}H_4[^{79}Br]F_6N_2O_2$ [M−H]$^-$: 388.9366. Found 388.9359.

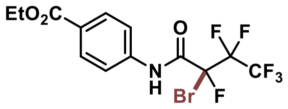

2j Prepared according to General Procedure A in a 1-dram vial from substrate **1j** (144.5 mg, 0.40 mmol, 1.0 equiv.), photocatalyst **PC1** (6.7 mg, 0.02 mmol, 0.05 equiv.), and LiBr (69.5 mg, 0.80 mmol, 2.0 equiv.) in anhydrous MeCN (2.0 mL). The reaction mixture was allowed to stir for 13 h under UV irradiation (370 nm) using the two light setup. ^{19}F NMR yield: 86%. Isolated in 84% yield (142.1 mg) as a colourless oil after purification by silica gel chromatography (15% EtOAc in hexanes, dry load).

^{1}H NMR (CDCl$_3$, 500 MHz) δ 8.18 (br s, 1H), 8.11 – 8.05 (m, 2H), 7.69 – 7.63 (m, 2H), 4.38 (q, *J* = 7.2 Hz, 2H), 1.40 (t, *J* = 7.2 Hz, 3H);
^{19}F NMR (CDCl$_3$, 470 MHz) δ −78.5 (d, *J* = 11.6 Hz, 3F), −115.4 (dd, *J* = 281.6, 9.2 Hz, 1F), −117.4 (dd, *J* = 281.6, 11.8 Hz, 1F), −132.2 – −132.4 (m, 1F);
^{13}C{^{1}H,^{19}F} NMR (CDCl$_3$, 126 MHz, partial ^{13}C{^{19}F} decoupling observed) δ 165.8, 158.3, 139.4, 131.1, 128.3, 120.0, 119.0 – 116.4 (m), 109.7 – 109.2 (m), 93.5, 61.4, 14.5;
HRMS (ESI): calcd. for $C_{13}H_9[^{79}Br]F_6N_2O_3$ [M−H]$^-$: 419.9675. Found 419.9673.

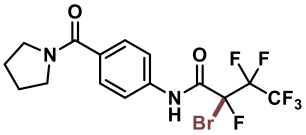

2k Prepared according to General Procedure A in a 1-dram vial from substrate **1k** (115.9 mg, 0.30 mmol, 1.0 equiv.), photocatalyst **PC1** (10.1 mg, 0.03 mmol, 0.1 equiv.), and LiBr (52.1 mg, 0.60 mmol, 2.0 equiv.) in anhydrous MeCN (1.5 mL). The reaction mixture was allowed to stir for 22 h under UV irradiation (370 nm) using the two light setup. ^{19}F NMR yield: 50%. Isolated in 41% yield (54.6 mg) as a colourless oil after purification by silica gel chromatography (30 → 50% EtOAc in $CHCl_3$, dry load).

^{1}H NMR ($CDCl_3$, 500 MHz) δ 8.74 (br s, 1H), 7.58 – 7.53 (m, 2H), 7.51 – 7.45 (m, 2H), 3.63 (dd, J = 6.9, 6.9 Hz, 2H), 3.42 (dd, J = 6.7, 6.7 Hz, 2H), 2.00 – 1.93 (m, 2H), 1.92 – 1.84 (m, 2H);
^{19}F NMR ($CDCl_3$, 470 MHz) δ −78.5 (d, J = 11.3 Hz, 3F), −115.3 (dd, J = 281.2, 9.1 Hz, 1F), −117.4 (dd, J = 281.3, 11.9 Hz, 1F), −132.0 – −132.2 (m, 1F);
^{13}C{^{1}H,^{19}F} NMR ($CDCl_3$, 126 MHz, partial ^{13}C{^{19}F} decoupling observed) δ 169.0, 158.6, 137.0, 134.8, 128.4, 120.7, 109.7 – 109.2 (m), 93.6, 49.8, 46.5, 26.5, 24.5;
HRMS (ESI): calcd. for $C_{15}H_{12}[^{79}Br]F_6N_2O_2$ $[M-H]^-$: 444.9992. Found 444.9993.

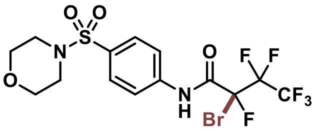

2l Prepared according to General Procedure A in a 1-dram vial from substrate **1l** (131.5 mg, 0.30 mmol, 1.0 equiv.), photocatalyst **PC1** (5.1 mg, 0.015 mmol, 0.05 equiv.), and LiBr (52.1 mg, 0.60 mmol, 2.0 equiv.) in anhydrous MeCN (1.5 mL). The reaction mixture was allowed to stir for 12 h under UV irradiation (370 nm) using the one light setup. ^{19}F NMR yield: 81%. Isolated in 77% yield (115.3 mg) as a white solid after purification by silica gel chromatography (33% EtOAc in hexanes, dry load).

^{1}H NMR (MeCN-d_3, 500 MHz) δ 9.50 (br s, 1H), 7.91 – 7.85 (m, 2H), 7.80 – 7.74 (m, 2H), 3.70 – 3.63 (m, 4H), 2.97 – 2.88 (m, 4H);
^{19}F NMR (MeCN-d_3, 470 MHz) δ −79.4 (d, J = 11.3 Hz, 3F), −115.8 (dd, J = 280.9, 8.9 Hz, 1F), −118.0 (dd, J = 280.9, 12.0 Hz, 1F), −133.2 – −133.4 (m, 1F);
^{13}C{^{1}H,^{19}F} NMR (MeCN-d_3, 126 MHz, partial ^{13}C{^{19}F} decoupling observed) δ 160.1, 141.5, 133.1, 130.0, 122.2, 110.7 – 110.4 (m), 94.0, 66.7, 47.1;
HRMS (ESI): calcd. for $C_{14}H_{12}[^{79}Br]F_6N_2O_4S$ $[M-H]^-$: 496.9611. Found 496.9615.

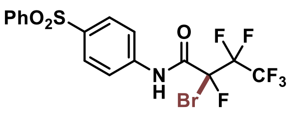

2m Prepared according to General Procedure A in a 1-dram vial from substrate **1m** (171.7 mg, 0.40 mmol, 1.0 equiv.), photocatalyst **PC1** (6.7 mg, 0.02 mmol, 0.05 equiv.), and LiBr (69.5 mg, 0.80 mmol, 2.0 equiv.) in anhydrous MeCN (2.0 mL). The reaction mixture was allowed to stir for 14 h under UV irradiation (370 nm) using the one light setup. ^{19}F NMR yield: 79%. Isolated in 78% yield (153.0 mg) as an off-white solid after purification by silica gel chromatography (15% EtOAc in hexanes, dry load).

^{1}H NMR ($CDCl_3$, 500 MHz) δ 8.24 (br s, 1H), 7.98 – 7.90 (m, 4H), 7.76 – 7.71 (m, 2H), 7.60 – 7.55 (m, 1H), 7.54 – 7.48 (m, 2H);
^{19}F NMR ($CDCl_3$, 470 MHz) δ −78.5 (d, *J* = 11.3 Hz, 3F), −115.4 (dd, *J* = 281.9, 9.0 Hz, 1F), −117.4 (dd, *J* = 282.0, 11.9 Hz, 1F), −132.5 – −132.7 (m, 1F);
^{13}C{^{1}H,^{19}F} NMR ($CDCl_3$, 126 MHz, partial ^{13}C{^{19}F} decoupling observed) δ 158.5, 141.4, 139.8, 139.0, 133.6, 129.6, 129.4, 127.7, 120.9, 119.1 – 116.3 (m), 109.5 – 109.1 (m), 93.3;
HRMS (ESI): calcd. for $C_{16}H_9[^{79}Br]F_6NO_3S$ $[M-H]^-$: 487.9396. Found 487.9405.

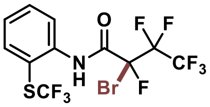

2n Prepared according to General Procedure A in a 1-dram vial from substrate **1n** (116.7 mg, 0.30 mmol, 1.0 equiv.), photocatalyst **PC1** (10.1 mg, 0.03 mmol, 0.1 equiv.), and LiBr (52.1 mg, 0.60 mmol, 2.0 equiv.) in anhydrous MeCN (1.5 mL). The reaction mixture was allowed to stir for 30 h under UV irradiation (370 nm) using the two light setup. ^{19}F NMR yield: 71%. Isolated in 60% yield (54.6 mg) as a colourless oil after purification by silica gel chromatography (9% $CHCl_3$ in hexanes, dry load).

^{1}H NMR ($CDCl_3$, 500 MHz) δ 9.20 (br s, 1H), 8.46 (dd, *J* = 8.3, 0.7 Hz, 1H), 7.75 (dd, *J* = 7.7, 0.7 Hz, 1H), 7.62 (ddd, *J* = 8.1, 8.1, 1.4 Hz, 1H), 7.30 (ddd, *J* = 7.7, 7.7, 1.1 Hz, 1H);
^{19}F NMR ($CDCl_3$, 470 MHz) δ −42.5 (s, 3F), −78.7 (d, *J* = 11.3 Hz, 3F), −115.4 (dd, *J* = 282.1, 8.9 Hz, 1F), −117.7 (dd, *J* = 282.2, 12.6 Hz, 1F), −132.2 – −132.4 (m, 1F);
^{13}C{^{1}H,^{19}F} NMR ($CDCl_3$, 126 MHz, partial ^{13}C{^{19}F} decoupling observed) δ 158.0, 139.23, 139.20, 133.9, 130.1 – 129.6 (m), 127.8 – 127.2 (m), 126.6, 121.4, 116.6, 113.4, 109.7 – 109.1 (m), 93.6;
HRMS (ESI): calcd. for $C_{11}H_4[^{79}Br]F_9NOS$ $[M-H]^-$: 447.9059. Found 447.9063.

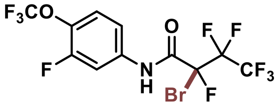

2o Prepared according to General Procedure A in a 1-dram vial from substrate **1o** (156.5 mg, 0.40 mmol, 1.0 equiv.), photocatalyst **PC1** (13.4 mg, 0.04 mmol, 0.1 equiv.), and LiBr (69.5 mg, 0.80 mmol, 2.0 equiv.) in anhydrous MeCN (2.0 mL). The reaction mixture was allowed to stir for 21 h under UV irradiation (370 nm) using the two light setup. ^{19}F NMR yield: 56%. Isolated in 48% yield (142.1 mg) as a white solid after purification by silica gel chromatography (17 → 33% $CHCl_3$ in hexanes, dry load).

^{1}H NMR ($CDCl_3$, 500 MHz) δ 8.08 (br s, 1H), 7.69 (dd, *J* = 11.3, 2.6 Hz, 1H), 7.34 (dd, *J* = 8.6, 8.6 Hz, 1H), 7.29 – 7.24 (m, 1H);
^{19}F NMR ($CDCl_3$, 470 MHz) δ −59.0 (d, *J* = 4.9 Hz, 3F), −78.6 (d, *J* = 11.4 Hz, 3F), −115.4 (dd, *J* = 282.2, 9.0 Hz, 1F), −117.5 (dd, *J* = 281.9, 12.0 Hz, 1F), −124.8 – −124.9 (m, 1F), −132.7 – −132.9 (m, 1F);
^{13}C{^{1}H,^{19}F} NMR ($CDCl_3$, 126 MHz, partial ^{13}C{^{19}F} decoupling observed) δ 158.4, 154.8, 135.2, 134.3, 124.6, 121.8 – 119.3 (m), 116.4, 116.3, 110.2, 109.5 – 109.2 (m), 93.4;
HRMS (ESI): calcd. for $C_{11}H_3[^{79}Br]F_{10}NO_2$ $[M-H]^-$: 449.9193. Found 449.9195.

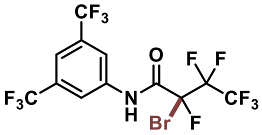

2p Prepared according to General Procedure A in a 1-dram vial from substrate **1p** (85.0 mg, 0.20 mmol, 1.0 equiv.), photocatalyst **PC1** (6.7 mg, 0.02 mmol, 0.1 equiv.), and LiBr (34.8 mg, 0.40 mmol, 2.0 equiv.) in anhydrous MeCN (1.0 mL). The reaction mixture was allowed to stir for 36 h under UV irradiation (370 nm) using the two light setup. ^{19}F NMR yield: 55%. Isolated in 51% yield (49.4 mg) as a white solid after purification by silica gel chromatography (17% $CHCl_3$ in hexanes, dry load).

^{1}H NMR ($CDCl_3$, 500 MHz) δ 8.27 (br s, 1H), 8.10 (s, 2H), 7.76 (s, 1H);
^{19}F NMR ($CDCl_3$, 470 MHz) δ −63.1 (s, 6F), −78.4 (d, *J* = 11.8 Hz, 3F), −115.5 (dd, *J* = 282.4, 9.3 Hz, 1F), −117.3 (dd, *J* = 282.4, 11.7 Hz, 1F), −133.0 – −133.2 (m, 1F);
^{13}C{^{1}H,^{19}F} NMR ($CDCl_3$, 126 MHz, partial ^{13}C{^{19}F} decoupling observed) δ 158.8, 137.0, 133.8 – 132.7 (m), 121.7, 120.5, 120.1 – 119.9 (m), 116.4, 109.6 – 109.2 (m), 93.2;
HRMS (ESI): calcd. for $C_{12}H_3[^{79}Br]F_{12}NO$ $[M-H]^-$: 483.9212. Found 483.9211.

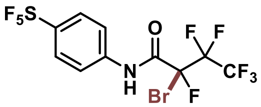

2q The experiment followed General Procedure A in a 1-dram vial from substrate **1q** (85.0 mg, 0.20 mmol, 1.0 equiv.), photocatalyst **PC2** (4.7 mg, 0.01 mmol, 0.05 equiv.), and LiBr (34.8 mg, 0.40 mmol, 2.0 equiv.) in anhydrous MeCN (1.0 mL). The reaction mixture was allowed to stir for 36 h under UV irradiation (370 nm) using the two light setup. ^{19}F NMR yield: 59%.

^{1}H NMR ($CDCl_3$, 500 MHz) δ 8.90 (br s, 1H), 7.77 – 7.74 (m, 2H), 7.73 – 7.70 (m, 2H);
^{19}F NMR ($CDCl_3$, 470 MHz) δ 84.9 – 83.5 (m, 1F), 63.4 – 62.9 (m, 4F), −78.6 (d, *J* = 11.5 Hz, 3F), −115.4 (dd, *J* = 281.6, 9.2 Hz, 1F), −117.5 (dd, *J* = 281.4, 11.9 Hz, 1F), −132.3 – −132.5 (m, 1F);
HRMS (ESI): calcd. for $C_{10}H_4[^{79}Br]F_{11}NOS$ $[M-H]^-$: 473.9027. Found 473.9021.

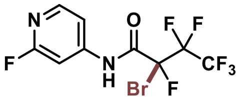

2r Prepared according to General Procedure A in a 1-dram vial from substrate **1r** (61.6 mg, 0.20 mmol, 1.0 equiv.), photocatalyst **PC2** (9.5 mg, 0.02 mmol, 0.10 equiv.), and LiBr (34.7 mg, 0.40 mmol, 2.0 equiv.) in anhydrous MeCN (1.0 mL). The reaction mixture was allowed to stir for 20 h under UV irradiation (370 nm) using the two light setup. ^{19}F NMR yield: 81%. Isolated in 58% yield (77.3 mg) as a beige solid after purification by silica gel chromatography (20% EtOAc in hexanes, dry load).

^{1}H NMR (Acetone-d_6, 500 MHz) δ 10.70 (br s, 1H), 8.25 – 8.21 (m, 1H), 7.66 – 7.62 (m, 1H), 7.51 – 7.49 (m, 1H);
^{19}F NMR (Acetone-d_6, 470 MHz) δ −67.8 – −68.0 (m, 1F), −79.4 (d, *J* = 11.3 Hz, 3F), −115.6 – −116.3 (m, 1F), −117.6 – −118.3 (m, 1F), −134.5 – −134.8 (m, 1F);
^{13}C{^{1}H,^{19}F} NMR (Acetone-d_6, 126 MHz, partial ^{13}C{^{19}F} decoupling observed) δ 166.9 – 163.9 (m), 160.6, 149.8 – 149.4 (m), 149.0, 120.3 – 116.9 (m), 113.9, 110.7 – 110.2 (m), 101.1 – 100.4 (m), 94.3 – 92.4 (m);
HRMS (ESI): calcd. for $C_9H_3[^{79}Br]F_7N_2O$ $[M-H]^-$: 366.9322. Found 366.9321.

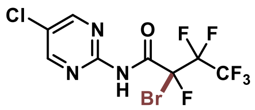

2s The experiment followed General Procedure A in a 1-dram vial from substrate **1s** (65.1 mg, 0.20 mmol, 1.0 equiv.), photocatalyst **PC2** (4.7 mg, 0.01 mmol, 0.05 equiv.), and LiBr (34.7 mg, 0.40 mmol, 2.0 equiv.) in anhydrous MeCN (1.0 mL). The reaction mixture was allowed to stir for 20 h under UV irradiation (370 nm) using the two light setup. ^{19}F NMR yield: 60%.

^{1}H NMR ($CDCl_3$, 500 MHz) δ 8.86 (br s, 1H), 8.66 (s, 2H);
^{19}F NMR ($CDCl_3$, 470 MHz) δ −78.4 (d, *J* = 11.3 Hz, 3F), −115.5 (dd, *J* = 281.7, 9.0 Hz, 1F), −117.2 (dd, *J* = 281.8, 11.7 Hz, 1F), −132.8 – −133.0 (m, 1F);
HRMS (ESI): calcd. for $C_8H_2[^{79}Br]ClF_6N_3O$ $[M-H]^-$: 383.8979. Found 383.8972.

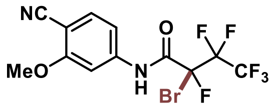

2t Prepared according to General Procedure A in a 1-dram vial from substrate **1t** (103.3 mg, 0.30 mmol, 1.0 equiv.), photocatalyst **PC1** (7.5 mg, 0.023 mmol, 0.075 equiv.), and LiBr (52.1 mg, 0.60 mmol, 2.0 equiv.) in anhydrous MeCN (1.5 mL). The reaction mixture was allowed to stir for 7 h under UV irradiation (370 nm) using the two light setup. ^{19}F NMR yield: 76%. Isolated in 72% yield (87.2 mg) as a white solid after purification by silica gel chromatography (20% EtOAc in hexanes, dry load).

^{1}H NMR ($CDCl_3$, 500 MHz) δ 8.38 (br s, 1H), 7.63 (d, *J* = 1.8 Hz, 1H), 7.55 (d, *J* = 8.4 Hz, 1H), 7.05 (dd, *J* = 8.4, 1.8 Hz, 1H), 3.95 (s, 3H);
^{19}F NMR ($CDCl_3$, 470 MHz) δ −78.5 (d, *J* = 11.5 Hz, 3F), −115.5 (dd, *J* = 281.9, 9.3 Hz, 1F), −117.3 (dd, *J* = 282.0, 11.6 Hz, 1F), −132.6 – −132.7 (m, 1F);
^{13}C{^{1}H,^{19}F} NMR ($CDCl_3$, 126 MHz, partial ^{13}C{^{19}F} decoupling observed) δ 162.5, 158.7, 141.2, 134.5, 118.9 – 116.4 (m), 116.0, 112.3, 109.6 – 109.2 (m), 103.5, 99.0, 93.3, 56.4;
HRMS (ESI): calcd. for $C_{12}H_6[^{79}Br]F_6N_2O_2$ $[M-H]^-$: 402.9522. Found 402.9515.

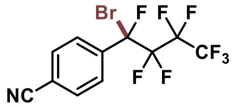

4a Prepared according to General Procedure B in a 4-dram vial from substrate **3a** (321.2 mg, 1.0 mmol, 1.0 equiv.), $Ir[dFCF_3ppy]_2(dtbpy)PF_6$ (22.4 mg, 0.02 mmol, 0.02 equiv.), photocatalyst **PC1** (16.8 mg, 0.05 mmol, 0.05 equiv.), and LiBr (173.7 mg, 2.0 mmol, 2.0 equiv.) in anhydrous MeCN (5.0 mL). The reaction mixture was allowed to stir for 40 h under UV irradiation (370 nm) using the two light setup. ^{19}F NMR yield: 65%. Isolated in 60% yield (229.0 mg) as a white solid after purification by silica gel chromatography (17% DCM in hexanes, wet load).

^{1}H NMR ($CDCl_3$, 500 MHz) δ 7.79 – 7.72 (m, 4H);
^{19}F NMR ($CDCl_3$, 470 MHz) δ −80.9 (td, *J* = 11.0, 2.0 Hz, 3F), −112.1 – −112.9 (m, 1F), −113.3 – −114.1 (m, 1F), −121.3 (dddd, *J* = 289.2, 16.8, 8.7, 7.2 Hz, 1F), −124.4 (dddd, *J* = 289.2, 25.2, 12.6, 4.3 Hz, 1F), −127.8 – −128.0 (m, 1F);
^{19}F{^{19}F} NMR ($CDCl_3$, 565 MHz) δ −80.9, −112.5, −113.7, −121.3, −124.4, −127.9;
^{13}C{^{1}H,^{19}F} NMR ($CDCl_3$, 126 MHz, partial ^{13}C{^{19}F} decoupling observed) δ 139.1, 132.5, 127.1, 117.6, 117.4, 115.3, 111.4 – 111.3 (m), 109.1, 100.2;

HRMS (EI): calcd. for $C_{11}H_4[^{79}Br]F_8N$ [M•]$^+$: 380.9394. Found 380.9378.

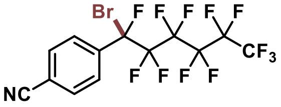

4b Prepared according to General Procedure B in a 1-dram vial from substrate **3b** (168.5 mg, 0.40 mmol, 1.0 equiv.), Ir[dFCF$_3$ppy]$_2$(dtbpy)PF$_6$ (9.0 mg, 0.008 mmol, 0.02 equiv.), photocatalyst **PC1** (6.7 mg, 0.02 mmol, 0.05 equiv.), and LiBr (69.5 mg, 0.80 mmol, 2.0 equiv.) in anhydrous MeCN (2.0 mL). The reaction mixture was allowed to stir for 36 h under UV irradiation (370 nm) using the one light setup. ^{19}F NMR yield: 55%. Isolated in 53% yield (101.3 mg) as a white solid after purification by silica gel chromatography (17% $CHCl_3$ in hexanes, wet load).

^{1}H NMR ($CDCl_3$, 500 MHz) δ 7.79 – 7.72 (m, 4H);
^{19}F NMR ($CDCl_3$, 470 MHz) δ −80.8 (tt, *J* = 10.0, 2.1 Hz, 3F), −111.1 – −111.9 (m, 1F), −112.8 – −113.6 (m, 1F), −117.0 – −117.9 (m, 1F), −119.5 – −120.4 (m, 1F), −121.7 – −122.5 (m, 1F), −122.6 – −123.5 (m, 1F), −125.2 – −126.0 (m, 1F), −126.2 – −127.0 (m, 1F), −125.5 – −127.7 (m, 1F);
^{13}C{^{1}H,^{19}F} NMR ($CDCl_3$, 126 MHz, partial ^{13}C{^{19}F} decoupling observed) δ 139.2, 132.5, 127.1, 117.6, 115.3, 112.2 – 111.6 (m), 111.4, 110.3, 109.0 – 108.2 (m), 100.5;
LRMS (EI): calcd. for $C_{13}H_4[^{79}Br]F_{12}N$ [M•]$^+$: 480.93. Found 480.96. calcd. for $C_{13}H_4[^{81}Br]F_{12}N$ [M•]$^+$: 482.93. Found 482.96.

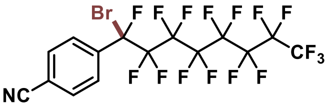

4c Prepared according to General Procedure B in a 1-dram vial from substrate **3c** (104.2 mg, 0.20 mmol, 1.0 equiv.), Ir[dFCF$_3$ppy]$_2$(dtbpy)PF$_6$ (4.5 mg, 0.004 mmol, 0.02 equiv.), photocatalyst **PC1** (3.4 mg, 0.01 mmol, 0.05 equiv.), and LiBr (34.8 mg, 0.40 mmol, 2.0 equiv.) in anhydrous MeCN (1.0 mL). The reaction mixture was allowed to stir for 40 h under UV irradiation (370 nm) using the one light setup. ^{19}F NMR yield: 70%. Isolated in 54% yield (62.6 mg) as a white solid after purification by silica gel chromatography (17 → 25% $CHCl_3$ in hexanes, dry load).

^{1}H NMR ($CDCl_3$, 500 MHz) δ 7.80 – 7.72 (m, 4H);
^{19}F NMR ($CDCl_3$, 470 MHz) δ −80.8 (tt, *J* = 9.9, 2.1 Hz, 3F), −111.0 – −111.8 (m, 1F), −112.8 – −113.6 (m, 1F), −116.8 – −117.7 (m, 1F), −119.3 – −120.2 (m, 1F), −121.0 – −123.5 (m, 6F), −125.4 – −126.1 (m, 1F), −126.1 – −126.9 (m, 1F), −127.4 – −127.6 (m, 1F);
^{13}C{^{1}H,^{19}F} NMR ($CDCl_3$, 126 MHz, partial ^{13}C{^{19}F} decoupling observed) δ 139.2, 132.5, 127.1, 117.6, 115.3, 112.2 – 111.7 (m), 111.5, 110.9, 110.8, 110.3, 108.9 – 108.2 (m), 100.5;
LRMS (EI): calcd. for $C_{15}H_4[^{79}Br]F_{16}N$ [M•]$^+$: 580.93. Found 581.01. calcd. for $C_{15}H_4[^{81}Br]F_{16}N$ [M•]$^+$: 582.92. Found 582.89.

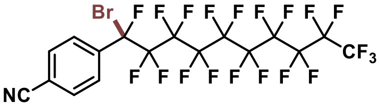

4d Prepared according to General Procedure B in a 1-dram vial from substrate **3d** (62.1 mg, 0.10 mmol, 1.0 equiv.), Ir[dFCF$_3$ppy]$_2$(dtbpy)PF$_6$ (2.2 mg, 0.002 mmol, 0.02 equiv.), photocatalyst **PC2** (2.4 mg, 0.005 mmol, 0.05 equiv.), and LiBr (17.4 mg, 0.20 mmol, 2.0 equiv.) in anhydrous MeCN (0.5 mL). The reaction mixture was allowed to stir for 40 h under UV irradiation (370 nm) using the two light setup. ^{19}F NMR yield: 75%. Isolated in 70% yield (47.5 mg) as a white solid after purification by silica gel chromatography (17 → 25% CHCl$_3$ in hexanes, dry load).

^{1}H NMR (CDCl$_3$, 600 MHz) δ 7.79 – 7.74 (m, 4H);
^{19}F NMR (CDCl$_3$, 565 MHz) δ −80.7 (tt, *J* = 10.1, 2.1 Hz, 3F), −111.0 – −111.7 (m, 1F), −112.8 – −113.5 (m, 1F), −116.8 – −117.6 (m, 1F), −119.3 – −120.1 (m, 1F), −121.0 – −122.4 (m, 8F), −122.5 – −123.3 (m, 2F), −125.5 – −126.7 (m, 2F), −127.4 – −127.6 (m, 1F);
^{13}C{^{1}H,^{19}F} NMR (CDCl$_3$, 126 MHz, partial ^{13}C{^{19}F} decoupling observed) δ 139.2, 132.5, 127.1, 117.6, 115.3, 112.5 – 111.6 (m), 111.5 – 111.2 (m), 110.89, 110.86, 110.83, 110.7, 110.3, 100.5;
HRMS/LRMS: not found under a variety of ionization techniques (ESI, EI, CI, APCI, MALDI).

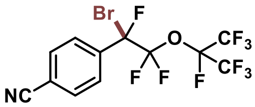

4e Prepared according to General Procedure B in a 1-dram vial from substrate **3e** (154.9 mg, 0.40 mmol, 1.0 equiv.), Ir[dFCF$_3$ppy]$_2$(dtbpy)PF$_6$ (9.0 mg, 0.008 mmol, 0.02 equiv.), photocatalyst **PC1** (6.7 mg, 0.02 mmol, 0.05 equiv.), and LiBr (69.5 mg, 0.80 mmol, 2.0 equiv.) in anhydrous MeCN (2.0 mL). The reaction mixture was allowed to stir for 40 h under UV irradiation (370 nm) using the two light setup. ^{19}F NMR yield: 68%. Isolated in 53% yield (94.1 mg) as a colourless oil after purification by silica gel chromatography (17 → 25% CHCl$_3$ in hexanes, dry load).

^{1}H NMR (CDCl$_3$, 500 MHz) δ 7.78 – 7.73 (m, 4H);
^{19}F NMR (CDCl$_3$, 470 MHz) δ −78.6 – −79.1 (m, 1F), −79.5 – −80.0 (m, 1F), −80.3 – −80.5 (m, 6F), −128.3 (dd, *J* = 10.1, 10.1 Hz, 1F), −145.5 – −145.7 (m, 1F);
^{13}C{^{1}H,^{19}F} NMR (CDCl$_3$, 126 MHz, partial ^{13}C{^{19}F} decoupling observed) δ 138.6, 132.3, 127.7, 119.4 – 118.5 (m), 117.7, 116.7 – 115.9 (m), 115.4, 99.6 – 98.8 (m);
HRMS (EI): calcd. for $C_{12}H_4[^{79}Br]F_{10}NO$ [M·]$^+$: 446.9311. Found 446.9332.

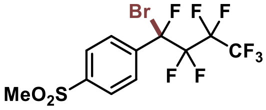

4f Prepared according to General Procedure B in a 4-dram vial from substrate **3f** (374.2 mg, 1.0 mmol, 1.0 equiv.), Ir[dFCF$_3$ppy]$_2$(dtbpy)PF$_6$ (22.4 mg, 0.02 mmol, 0.02 equiv.), photocatalyst **PC1** (16.8 mg, 0.05 mmol, 0.05 equiv.), and LiBr (173.7 mg, 2.0 mmol, 2.0 equiv.) in anhydrous MeCN (5.0 mL). The reaction mixture was allowed to stir for 42 h under UV irradiation (370 nm) using the two light setup. ^{19}F NMR yield: 85%. Isolated in 67% yield (291.0 mg) as a white solid after purification by silica gel chromatography (50 → 75% DCM in hexanes, wet load).

^{1}H NMR (CDCl$_3$, 500 MHz) δ 8.07 – 8.02 (m, 2H), 7.87 – 7.81 (m, 2H), 3.10 (s, 3H);

^{19}F NMR ($CDCl_3$, 470 MHz) δ −80.9 (td, J = 11.0, 2.3 Hz, 3F), −112.1 – −112.9 (m, 1F), −113.3 – −114.1 (m, 1F), −121.3 (dddd, J = 289.3, 16.1, 9.4, 7.1 Hz, 1F), −124.3 (dddd, J = 289.0, 25.0, 12.5, 4.1 Hz, 1F), −127.4 – −127.6 (m, 1F);
^{13}C{^{1}H,^{19}F} NMR ($CDCl_3$, 126 MHz, partial ^{13}C{^{19}F} decoupling observed) δ 143.2, 140.1, 127.9, 127.5, 118.5 – 116.0 (m), 111.7 – 111.1 (m), 109.6 – 108.8 (m), 100.2, 44.5;
HRMS (ESI): calcd. for $C_{11}H_7[^{79}Br]F_8NaO_2S$ $[M+Na]^+$: 456.9115. Found 456.9115.

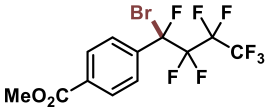

4g The experiment followed General Procedure B in a 1-dram vial from substrate **3g** (35.2 mg, 0.10 mmol, 1.0 equiv.), Ir[dFCF$_3$ppy]$_2$(dtbpy)PF$_6$ (2.2 mg, 0.002 mmol, 0.02 equiv.), photocatalyst **PC1** (1.7 mg, 0.005 mmol, 0.05 equiv.), and LiBr (17.4 mg, 0.20 mmol, 2.0 equiv.) in anhydrous MeCN (0.5 mL). The reaction mixture was allowed to stir for 44 h under UV irradiation (370 nm) using the two light setup. ^{19}F NMR yield: 41%.

^{1}H NMR ($CDCl_3$, 600 MHz) δ 8.10 – 8.07 (m, 2H), 7.69 – 7.66 (m, 2H), 3.92 (s, 3H);
^{19}F NMR ($CDCl_3$, 565 MHz) δ −81.0 (m, 3F), −112.1 – −112.7 (m, 1F), −113.4 – −114.1 (m, 1F), −121.3 (dddd, J = 289.0, 16.3, 7.9, 7.9 Hz, 1F), −124.7 (dddd, J = 288.9, 25.6, 12.8, 4.7 Hz, 1F), −127.0 – −127.2 (m, 1F);
HRMS/LRMS: not found under a variety of ionization techniques (ESI, EI, CI, APCI, MALDI).

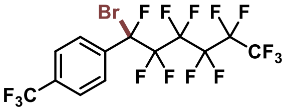

4h Prepared according to General Procedure B in a 1-dram vial from substrate **3h** (92.8 mg, 0.20 mmol, 1.0 equiv.), Ir[dFCF$_3$ppy]$_2$(dtbpy)PF$_6$ (4.5 mg, 0.004 mmol, 0.02 equiv.), photocatalyst **PC2** (4.7 mg, 0.01 mmol, 0.05 equiv.), and LiBr (34.7 mg, 0.40 mmol, 2.0 equiv.) in anhydrous MeCN (1.0 mL). The reaction mixture was allowed to stir for 36 h under UV irradiation (370 nm) using the two light setup. ^{19}F NMR yield: 60%. Isolated in 40% yield (41.7 mg), >98:2 *rr* as a colourless oil after purification by silica gel chromatography (100% pentane, dry load).

^{1}H NMR ($CDCl_3$, 500 MHz) δ 7.80 – 7.69 (m, 4H);
^{19}F NMR ($CDCl_3$, 470 MHz) δ −63.2 (s, 3F), −80.9 (tt, J = 10.1, 2.1 Hz, 3F), −111.1 – −112.0 (m, 1F), −112.9 – −113.7 (m, 1F), −117.0 – −118.0 (m, 1F), −119.6 – −120.5 (m, 1F), −121.7 – −122.6 (m, 1F), −122.7 – −123.5 (m, 1F), −125.2 – −126.1 (m, 1F), −126.2 – −127.2 (m, 2F);
^{13}C{^{1}H,^{19}F} NMR ($CDCl_3$, 126 MHz, partial ^{13}C{^{19}F} decoupling observed) δ 138.4, 133.8 – 132.8 (m), 124.7 – 122.4 (m), 126.9, 125.8, 118.7 – 115.9 (m), 112.1 – 111.8 (m), 111.4, 110.4, 109.1 – 108.0 (m), 100.9;
LRMS (EI): calcd. for $C_{13}H_4[^{79}Br]F_{15}$ $[M\cdot]^+$: 523.93. Found 523.89. calcd. for $C_{13}H_4[^{81}Br]F_{15}$ $[M\cdot]^+$: 525.92. Found 525.85.

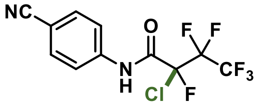

5a Prepared according to General Procedure C in a ½-dram vial from substrate **1a** (31.4 mg, 0.10 mmol, 1.0 equiv.), photocatalyst **PC2** (2.4 mg, 0.005 mmol, 0.05 equiv.), activated 3Å molecular sieves (×2), and LiCl (8.5 mg, 0.20 mmol, 2.0 equiv.) in anhydrous MeCN (0.5 mL). The reaction mixture was allowed to stir for 4 h under UV/green irradiation (370 + 525 nm) using the two light setup. ^{19}F NMR yield: 70%. Isolated in 63% yield (21.0 mg) as a beige solid after purification by silica gel chromatography (15% EtOAc in hexanes, dry load).

^{1}H NMR ($CDCl_3$, 500 MHz) δ 8.26 (br s, 1H), 7.77 – 7.73 (m, 2H), 7.72 – 7.68 (m, 2H);
^{19}F NMR ($CDCl_3$, 470 MHz) δ −78.8 (d, *J* = 11.5 Hz, 3F), −118.8 (dd, *J* = 282.5, 5.6 Hz, 1F), −120.9 (dd, *J* = 282.3, 7.4 Hz, 1F), −130.3 – −130.5 (m, 1F);
^{13}C{^{1}H,^{19}F} NMR ($CDCl_3$, 126 MHz, partial ^{13}C{^{19}F} decoupling observed) δ 157.6, 139.4, 133.7, 120.8, 118.2, 109.8, 109.7 – 109.1 (m), 100.7;
HRMS (ESI): calcd. for $C_{11}H_4[^{35}Cl]F_6N_2O$ [M−H]$^-$: 328.9922. Found 328.9916.

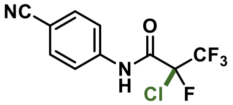

5b Prepared according to General Procedure C in a ½-dram vial from substrate **1b** (26.4 mg, 0.10 mmol, 1.0 equiv.), photocatalyst **PC2** (2.4 mg, 0.005 mmol, 0.05 equiv.), activated 3Å molecular sieves (×2), and LiCl (8.5 mg, 0.20 mmol, 2.0 equiv.) in anhydrous MeCN (0.5 mL). The reaction mixture was allowed to stir for 14 h under UV/green irradiation (370 + 525 nm) using the two light setup. ^{19}F NMR yield: 55%. Isolated in 51% yield (14.2 mg) as a white solid after purification by silica gel chromatography (15 → 20% EtOAc in hexanes, dry load).

^{1}H NMR ($CDCl_3$, 500 MHz) δ 8.22 (br s, 1H), 7.77 – 7.73 (m, 2H), 7.72 – 7.68 (m, 2H);
^{19}F NMR ($CDCl_3$, 470 MHz) δ −79.1 (d, *J* = 6.6 Hz, 3F), −131.9 – −132.0 (m, 1F);
^{13}C{^{1}H,^{19}F} NMR ($CDCl_3$, 126 MHz, partial ^{13}C{^{19}F} decoupling observed) δ 157.6, 139.4, 133.7, 120.7, 118.2, 109.8, 99.7 – 99.0 (m);
HRMS (ESI): calcd. for $C_{10}H_4[^{35}Cl]F_4N_2O$ [M−H]$^-$: 278.9954. Found 278.9948.

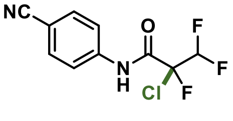

5c Prepared according to General Procedure C in a ½-dram vial from substrate **1c** (24.6 mg, 0.10 mmol, 1.0 equiv.), photocatalyst **PC2** (2.4 mg, 0.005 mmol, 0.05 equiv.), activated 3Å molecular sieves (×2), and LiCl (8.5 mg, 0.20 mmol, 2.0 equiv.) in anhydrous MeCN (0.5 mL). The reaction mixture was allowed to stir for 20 h under UV/green irradiation (370 + 525 nm) using the two light setup. ^{19}F NMR yield: 56%. Isolated in 49% yield (12.9 mg) as a white solid after purification by silica gel chromatography (15 → 20% EtOAc in hexanes, dry load).

^{1}H NMR ($CDCl_3$, 500 MHz) δ 8.20 (br s, 1H), 7.76 – 7.72 (m, 2H), 7.72 – 7.68 (m, 2H), 6.27 (td, *J* = 53.2, 10.9 Hz, 1H);

^{19}F NMR ($CDCl_3$, 470 MHz) δ −128.1 (ddd, J = 283.1, 52.7, 16.9 Hz, 1F), −138.2 (ddd, J = 283.1, 53.0, 12.3 Hz, 1F), −141.4 – −141.6 (m, 1F);
^{13}C{^{1}H,^{19}F} NMR ($CDCl_3$, 126 MHz, partial ^{13}C{^{19}F} decoupling observed) δ 160.1, 139.5, 133.7, 120.6, 118.3, 111.3 – 110.7 (m), 109.7, 100.6 – 100.2 (m);
HRMS (ESI): calcd. for $C_{10}H_5[^{35}Cl]F_3N_2O$ [M−H]$^-$: 261.0048. Found 261.0048.

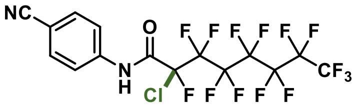

5g Prepared according to General Procedure C in a ½-dram vial from substrate **1g** (51.4 mg, 0.10 mmol, 1.0 equiv.), photocatalyst **PC2** (2.4 mg, 0.005 mmol, 0.05 equiv.), activated 3Å molecular sieves (×2), and LiCl (8.5 mg, 0.20 mmol, 2.0 equiv.) in anhydrous MeCN (0.5 mL). The reaction mixture was allowed to stir for 20 h under UV/green irradiation (370 + 525 nm) using the two light setup. ^{19}F NMR yield: 52%. Isolated in 42% yield (22.1 mg) as a beige solid after purification by silica gel chromatography (15 → 20% EtOAc in hexanes, dry load).

^{1}H NMR (Acetone-d_6, 500 MHz) δ 10.55 (br s, 1H), 8.00 – 7.96 (m, 2H), 7.87 – 7.83 (m, 2H);
^{19}F NMR (Acetone-d_6, 470 MHz) δ −81.6 (tt, J = 10.1, 2.1 Hz, 3F), −113.4 – −114.2 (m, 1F), −116.8 – −117.6 (m, 1F), −118.7 – −119.5 (m, 1F), −119.6 – −120.4 (m, 1F), −122.3 – −122.6 (m, 2F), −123.0 – −123.3 (m, 2F), −126.6 – −126.8 (m, 2F), −130.1 – −130.5 (m, 1F);
^{13}C{^{1}H,^{19}F} NMR (Acetone-d_6, 126 MHz, partial ^{13}C{^{19}F} decoupling observed) δ 159.1, 141.5, 134.2, 122.3, 122.2 – 122.1 (m), 119.0, 112.8 – 112.5 (m), 112.4, 111.7, 111.2, 110.0, 109.7 – 108.9 (m), 102.0;
HRMS (ESI): calcd. for $C_{15}H_4[^{35}Cl]F_{14}N_2O$ [M−H]$^-$: 528.9794. Found 528.9785.

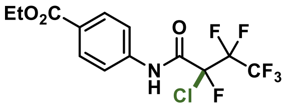

5j The experiment followed General Procedure C in a ½-dram vial from substrate **1j** (36.1 mg, 0.10 mmol, 1.0 equiv.), photocatalyst **PC2** (2.4 mg, 0.005 mmol, 0.05 equiv.), activated 3Å molecular sieves (×2), and LiCl (8.5 mg, 0.20 mmol, 2.0 equiv.) in anhydrous MeCN (0.5 mL). The reaction mixture was allowed to stir for 4 h under UV/green irradiation (370 + 525 nm) using the two light setup. ^{19}F NMR yield: 50%.

^{1}H NMR ($CDCl_3$, 600 MHz) δ 8.46 (br s, 1H), 8.03 – 8.00 (m, 2H), 7.68 – 7.65 (m, 2H), 4.34 (q, J = 7.1 Hz, 2H), 1.36 (t, J = 7.1 Hz, 3H);
^{19}F NMR ($CDCl_3$, 565 MHz) δ −78.9 (d, J = 11.6 Hz, 3F), −118.8 (dd, J = 281.7, 5.7 Hz, 1F), −121.0 (dd, J = 281.5, 7.5 Hz, 1F), −130.0 – −130.2 (m, 1F);
HRMS (ESI): calcd. for $C_{13}H_9[^{35}Cl]F_6NO_3$ [M−H]$^-$: 376.0181. Found 376.0178.

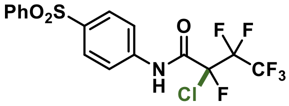

5m Prepared according to General Procedure C in a ½-dram vial from substrate **1m** (42.9 mg, 0.10 mmol, 1.0 equiv.), photocatalyst **PC2** (4.7 mg, 0.01 mmol, 0.10 equiv.), activated 3Å molecular sieves (×2), and LiCl (8.5 mg, 0.20 mmol, 2.0 equiv.) in anhydrous MeCN (0.5 mL). The reaction mixture was allowed to stir for 20 h under UV/green irradiation (370 + 525 nm) using the two light setup. ^{19}F NMR yield: 47%. Isolated in 43% yield (19.2 mg) as a white solid after purification by silica gel chromatography (20% EtOAc in hexanes, dry load).

^{1}H NMR ($CDCl_3$, 600 MHz) δ 8.23 (br s, 1H), 7.99 – 7.95 (m, 2H), 7.94 – 7.90 (m, 2H), 7.75 – 7.71 (m, 2H), 7.61 – 7.56 (m, 1H), 7.54 – 7.49 (m, 2H);
^{19}F NMR ($CDCl_3$, 565 MHz) δ −78.8 (d, *J* = 11.5 Hz, 3F), −118.8 (dd, *J* = 282.4, 5.6 Hz, 1F), −120.9 (dd, *J* = 282.4, 7.5 Hz, 1F), −130.3 – −130.5 (m, 1F);
^{13}C{^{1}H,^{19}F} NMR ($CDCl_3$, 126 MHz, partial ^{13}C{^{19}F} decoupling observed) δ 157.6, 141.6 – 141.3 (m), 139.7, 139.2 – 138.9 (m), 133.6, 129.6, 129.4, 127.8, 120.9, 110.0 – 108.8 (m), 100.7;
HRMS (ESI): calcd. for $C_{16}H_9[^{35}Cl]F_6NO_3S$ $[M-H]^-$: 443.9901. Found 443.9895.

5 Starting Material Synthesis & Characterization Data

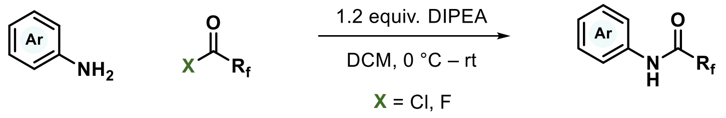

General Procedure D (Perfluoroalkylamides from Perfluoroacyl Halides): adapted from a literature procedure.[11] To a 50 mL 2-neck round bottom flask charged with a stir bar was added aniline substrate (2.0 mmol, 1.0 equiv.) and DIPEA (310 mg, 2.4 mmol, 1.2 equiv.). The flask was cooled to 0 °C in an ice bath and subsequently put under an N_2 atmosphere. Anhydrous DCM (10 mL) was then added, followed by dropwise addition of the corresponding perfluoroacyl halide (2.0 mmol, 1.0 equiv.) under a stream of N_2. The flask was then removed from the ice bath, allowed to warm to room temperature, and stirred for 14–20 hours. Upon completion of the reaction as monitored by TLC or ^{19}F NMR, the reaction mixture was concentrated *in vacuo*. The crude material was then dissolved in EtOAc (40 mL) and washed sequentially with 1 M HCl (1 × 10 mL) and brine (1 × 10 mL). The combined organics were dried over Na_2SO_4, filtered, concentrated *in vacuo*, and purified by silica gel chromatography.

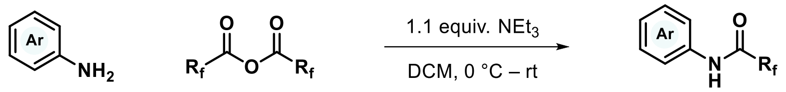

General Procedure E (Perfluoroalkylamides from Perfluoroalkylanhydrides): adapted from a literature procedure.[12] To a 50 mL 2-neck round bottom flask charged with a stir bar was added aniline substrate (1.5 mmol, 1.0 equiv.) and NEt_3 (167 mg, 1.65 mmol, 1.1 equiv.). The flask was cooled to 0 °C in an ice bath and subsequently put under an N_2 atmosphere. Anhydrous DCM (5.0 mL) was then added, followed by dropwise addition of the corresponding perfluoroalkylanhydride (1.65 mmol, 1.1 equiv.) under a stream of N_2. The flask was then removed from the ice bath, allowed to warm to room temperature, and stirred for 14–20 hours. Upon completion of the reaction as monitored by TLC or ^{19}F NMR, the reaction mixture was concentrated *in vacuo*. The crude material was then dissolved in EtOAc (40 mL) and washed sequentially with saturated NH_4Cl (1 × 10 mL) and brine (1 × 10 mL). The combined organics were dried over Na_2SO_4, filtered, concentrated *in vacuo*, and purified by silica gel chromatography.

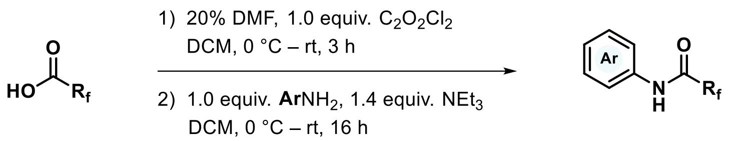

General Procedure F (Perfluoroalkylamides from Perfluoroalkylacids): adapted from a literature procedure.[13] To a 25 mL round bottom flask (flask 1) charged with a stir bar was added perfluoroalkyl acid (2.5 mmol, 1.0 equiv.) and anhydrous DCM (3.5 mL). The flask was cooled to 0 °C in an ice bath and subsequently put under an N_2 atmosphere. This was followed by the dropwise addition of DMF (38.6 µL, 0.5 mmol, 0.2 equiv.). A solution of oxalyl chloride (215 µL, 2.5 mmol, 1.0 equiv.) in anhydrous DCM (1.25 mL) was then added dropwise, and the reaction mixture was warmed to room

temperature and stirred for 3 h. In a separate 2-neck round bottom flask (flask 2) charged with a stir bar was added aniline substrate (2.5 mmol, 1.0 equiv.) and anhydrous DCM (5 mL). The flask was cooled to 0 °C in an ice bath and subsequently put under an N_2 atmosphere. This was followed by the addition of NEt_3 (488 µL, 3.5 mmol, 1.4 equiv.). The contents of flask 1 were then added dropwise to flask 2 under a stream of N_2, and the reaction was warmed to room temperature and stirred for an additional 16 h. Upon completion of the reaction as monitored by TLC or ^{19}F NMR, the reaction mixture was diluted in Et_2O (25 mL), filtered through a celite pad, concentrated *in vacuo*, and purified by silica gel chromatography.

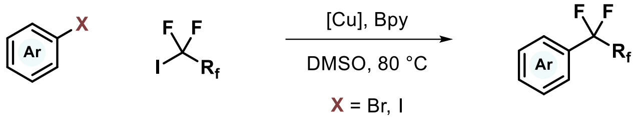

General Procedure G (Perfluoroalkylarenes from Perfluoroalkyl Halides): adapted from a literature procedure.[14] To a 4-dram vial charged with a stir bar was added copper powder (731 mg, 11.5 mmol, 2.3 equiv.), 2,2'-bipyridine (62.5 mg, 0.4 mmol, 0.08 equiv.), aryl halide (5.0 mmol, 1.0 equiv.), and anhydrous DMSO (8.0 mL). The reaction mixture was put under an N_2 atmosphere, followed by the addition of the corresponding perfluoroalkyl iodide (6.0 mmol, 1.2 equiv.) under a stream of N_2. The reaction mixture was then heated to 80 °C in an aluminum block and stirred for 48–72 h. Upon completion of the reaction as determined by TLC or ^{19}F NMR, the crude mixture was diluted with $CHCl_3$ (50 mL) and filtered through a celite pad. The crude organics were then washed with concentrated aqueous ammonia (30%, 3 × 20 mL). In some cases, additional washes were needed to completely remove the excess copper. The organics were then washed with brine (1 × 20 mL), dried over Na_2SO_4, filtered, concentrated *in vacuo*, and purified by silica gel chromatography.

Note: *if the perfluoroalkyl iodide is a solid, it should be added to the reaction mixture prior to the addition of DMSO.*

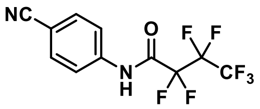

1a Prepared according to General Procedure D from 4-aminobenzonitrile (591 mg, 5.0 mmol, 1.0 equiv.), DIPEA (775 mg, 6.0 mmol, 1.2 equiv.), and perfluorobutyryl chloride (747 µL, 5.0 mmol, 1.0 equiv.) in anhydrous DCM (25 mL). The reaction mixture was allowed to stir for 16 h at room temperature. Isolated in 75% yield (1.18 g) as a white solid after purification by silica gel chromatography (20% EtOAc in hexanes, dry load).

^{1}H NMR ($CDCl_3$, 500 MHz) δ 8.56 (br s, 1H), 7.81 – 7.76 (m, 2H), 7.72 – 7.67 (m, 2H);
^{19}F NMR ($CDCl_3$, 470 MHz) δ −80.5 (t, *J* = 8.7 Hz, 3F), −120.1 (q, *J* = 8.8 Hz, 2F), −126.6 (s, 2F);
^{13}C{^{1}H,^{19}F} NMR ($CDCl_3$, 126 MHz, partial $^{13}C\{^{19}F\}$ decoupling observed) δ 155.9, 139.5, 133.6, 120.9, 118.3, 117.6 – 116.0 (m), 109.6, 108.4, 108.3 – 108.2 (m);
HRMS (ESI): calcd. for $C_{11}H_4F_7N_2O$ $[M-H]^-$: 313.0217. Found 313.0216.

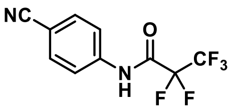

1b Prepared according to General Procedure E from 4-aminobenzonitrile (1.01 g, 8.5 mmol, 1.0 equiv.), NEt_3 (946 mg, 9.35 mmol, 1.1 equiv.), and pentafluoropropionic anhydride (1.85 mL, 9.35 mmol, 1.1 equiv.) in anhydrous DCM (9 mL). The reaction mixture was allowed to stir for 20 h at room temperature. Isolated in 75% yield (1.68 g) as a white solid after purification by silica gel chromatography (15% EtOAc in hexanes, dry load).

^{1}H NMR ($CDCl_3$, 500 MHz) δ 8.39 (br s, 1H), 7.80 – 7.74 (m, 2H), 7.73 – 7.67 (m, 2H);
^{19}F NMR ($CDCl_3$, 470 MHz) δ −82.4 (s, 3F), −122.3 (s, 2F);
^{13}C{^{1}H,^{19}F} NMR ($CDCl_3$, 126 MHz, partial ^{13}C{^{19}F} decoupling observed) δ 156.0, 139.4, 133.7, 120.8, 118.2, 118.0 – 116.4 (m), 109.8, 106.8;
HRMS (ESI): calcd. for $C_{10}H_4F_5N_2O$ $[M-H]^-$: 263.0249. Found 263.0251.

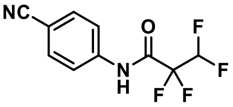

1c Prepared according to General Procedure E from 4-aminobenzonitrile (177 mg, 1.5 mmol, 1.0 equiv.), NEt_3 (167 mg, 1.65 mmol, 1.1 equiv.), and 2,2,3,3-tetrafluoropropanoic anhydride (285 μL, 1.65 mmol, 1.1 equiv.) in anhydrous DCM (5 mL). The reaction mixture was allowed to stir for 14 h at room temperature. Isolated in 82% yield (301 mg) as a white solid after purification by silica gel chromatography (20% EtOAc in hexanes, dry load).

^{1}H NMR (MeCN-d_3, 500 MHz) δ 9.48 (br s, 1H), 7.85 – 7.79 (m, 2H), 7.77 – 7.71 (m, 2H), 6.40 (tt, *J* = 52.2, 5.3 Hz, 1H);
^{19}F NMR (MeCN-d_3, 470 MHz) δ −125.5 – −125.6 (m, 2F), −139.9 (dt, *J* = 50.0 Hz, 6.8 Hz, 2F);
^{13}C{^{1}H,^{19}F} NMR (MeCN-d_3, 126 MHz) δ 158.5, 140.3, 133.4, 121.1, 118.4, 109.2, 109.1, 108.7;
HRMS (ESI): calcd. for $C_{10}H_5F_4N_2O$ $[M-H]^-$: 245.0343. Found 245.0349.

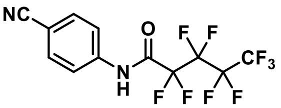

1d Prepared according to General Procedure D from 4-aminobenzonitrile (473 mg, 4.0 mmol, 1.0 equiv.), DIPEA (620 mg, 4.8 mmol, 1.2 equiv.), and nonafluoropentanoyl chloride (744 μL, 4.4 mmol, 1.1 equiv.) in anhydrous DCM (20 mL). The reaction mixture was allowed to stir for 20 h at room temperature. Isolated in 35% yield (508 mg) as a white solid after purification by silica gel chromatography (15% EtOAc in hexanes, dry load).

^{1}H NMR ($CDCl_3$, 500 MHz) δ 8.46 (br s, 1H), 7.80 – 7.74 (m, 2H), 7.72 – 7.67 (m, 2H);
^{19}F NMR ($CDCl_3$, 470 MHz) δ −80.9 (t, *J* = 9.7 Hz, 3F), −119.4 (t, *J* = 12.2 Hz, 2F), −123.0 – −123.3 (m, 2F), −125.7 – −125.9 (m, 2F);
^{13}C{^{1}H,^{19}F} NMR ($CDCl_3$, 126 MHz, partial ^{13}C{^{19}F} decoupling observed) δ 155.8, 139.5, 133.7, 120.9, 118.2, 116.6 – 115.8 (m), 110.1, 109.7, 108.8, 108.7 – 108.2 (m);
HRMS (ESI): calcd. for $C_{12}H_4F_9N_2O$ $[M-H]^-$: 363.0185. Found 363.0173.

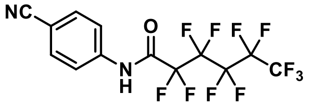

1e Prepared according to General Procedure F from perfluorohexanoic acid (785 mg, 2.5 mmol, 1.0 equiv.), DMF (38.6 µL, 0.5 mmol, 0.2 equiv.), oxalyl chloride (215 µL, 2.5 mmol, 1.0 equiv.), 4-aminobenzonitrile (295 mg, 2.5 mmol, 1.0 equiv.), and NEt_3 (488 µL, 3.5 mmol, 1.4 equiv.) in anhydrous DCM (9.8 mL). The contents of flask 1 were stirred at room temperature for 3 h, and the combined contents in flask 2 were allowed to stir for 16 h at room temperature. Isolated in 20% yield (213 mg) as a white solid after purification by silica gel chromatography (20% EtOAc in hexanes, dry load).

^{1}H NMR (Acetone-d_6, 500 MHz) δ 10.68 (br s, 1H), 8.02 – 7.96 (m, 2H), 7.89 – 7.83 (m, 2H);
^{19}F NMR (Acetone-d_6, 470 MHz) δ −81.6 – −81.8 (m, 3F), −119.6 – −119.8 (m, 2F), −122.9 – −123.2 (m, 4F), −126.7 – −127.0 (m, 2F);
^{13}C{^{1}H,^{19}F} NMR (Acetone-d_6, 126 MHz, partial ^{13}C{^{19}F} decoupling observed) δ 156.7, 141.3, 134.2, 122.2, 118.9, 117.4 – 116.7 (m), 111.5, 111.3, 110.0, 109.7, 109.6 – 109.0 (m);
HRMS (ESI): calcd. for $C_{13}H_4F_{11}N_2O$ [M−H]$^-$: 413.0153. Found 413.0159.

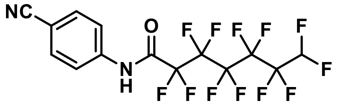

1f Prepared according to General Procedure D from 4-aminobenzonitrile (473 mg, 4.0 mmol, 1.0 equiv.), DIPEA (620 mg, 4.8 mmol, 1.2 equiv.), and 7*H*-perfluoroheptanoyl chloride (875 µL, 4.0 mmol, 1.0 equiv.) in anhydrous DCM (20 mL). The reaction mixture was allowed to stir for 16 h at room temperature. Isolated in 85% yield (1.51 g) as a white solid after purification by silica gel chromatography (20% EtOAc in hexanes, wet load).

^{1}H NMR ($CDCl_3$, 500 MHz) δ 8.38 (br s, 1H), 7.80 – 7.74 (m, 2H), 7.73 – 7.67 (m, 2H), 6.06 (tt, *J* = 51.9, 5.0 Hz, 1H);
^{19}F NMR ($CDCl_3$, 470 MHz) δ −119.2 (t, *J* = 12.8 Hz, 2F), −121.5 – −121.8 (m, 2F), −122.1 – −122.4 (m, 2F), −123.2 – −123.5 (m, 2F), −129.1 – −129.3 (m, 2F), −136.8 – −137.2 (m, 2F);
^{13}C{^{1}H,^{19}F} NMR ($CDCl_3$, 126 MHz, partial ^{13}C{^{19}F} decoupling observed) δ 155.9, 139.4, 133.7, 120.8, 118.2, 111.0, 110.9 – 110.7 (m), 110.6, 110.2 – 110.0 (m), 109.8, 109.0, 107.7;
HRMS (ESI): calcd. for $C_{14}H_5F_{12}N_2O$ [M−H]$^-$: 445.0216. Found 445.0223.

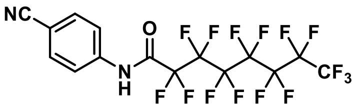

1g Prepared according to General Procedure D from 4-aminobenzonitrile (236 mg, 2.0 mmol, 1.0 equiv.), DIPEA (310 mg, 2.4 mmol, 1.2 equiv.), and perfluorooctanoyl chloride (496 µL, 2.0 mmol, 1.0 equiv.) in anhydrous DCM (10 mL). The reaction mixture was allowed to stir for 16 h at room temperature. Isolated in 75% yield (1.03 g) as a white solid after purification by silica gel chromatography (15% EtOAc in hexanes, dry load).

^{1}H NMR (MeCN-d_3, 500 MHz) δ 9.60 (br s, 1H), 7.84 – 7.79 (m, 2H), 7.78 – 7.73 (m, 2H);

^{19}F NMR (MeCN-d$_3$, 470 MHz) δ −81.6 (tt, *J* = 10.3, 2.3 Hz, 3F), −119.6 (tt, *J* = 13.1, 2.7 Hz, 2F), −121.9 – −122.1 (m, 2F), −122.3 – −122.6 (m, 2F), −122.8 – −123.0 (m, 2F), −123.1 – −123.3 (m, 2F), −126.5 – −126.7 (m, 2F);
^{13}C{^{1}H,^{19}F} NMR (MeCN-d$_3$, 126 MHz, partial ^{13}C{^{19}F} decoupling observed) δ 156.9, 141.1, 134.4, 122.3, 119.3, 119.1 – 116.2 (m), 111.9, 111.8, 111.7, 111.2, 110.1, 109.8, 109.6 – 109.2 (m);
HRMS (ESI): calcd. for $C_{15}H_4F_5N_2O$ [M−H]$^-$: 513.0090. Found 513.0085.

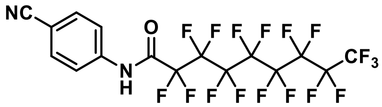

1h Prepared according to General Procedure D from 4-aminobenzonitrile (148 mg, 1.25 mmol, 1.0 equiv.), DIPEA (199 mg, 1.5 mmol, 1.2 equiv.), and perfluorononanoyl chloride (350 μL, 1.25 mmol, 1.0 equiv.) in anhydrous DCM (6 mL). The reaction mixture was allowed to stir for 14 h at room temperature. Isolated in 66% yield (468 mg) as a white solid after purification by silica gel chromatography (15% EtOAc in hexanes, dry load).

^{1}H NMR (MeCN-d$_3$, 500 MHz) δ 9.58 (br s, 1H), 7.84 – 7.79 (m, 2H), 7.78 – 7.74 (m, 2H);
^{19}F NMR (MeCN-d$_3$, 470 MHz) δ −81.5 (tt, *J* = 10.3, 2.1 Hz, 3F), −119.6 (tt, *J* = 13.1, 2.5 Hz, 2F), −121.8 – −122.1 (m, 2F), −122.1 – −122.5 (m, 4F), −122.7 – −123.0 (m, 2F), −123.0 – −123.3 (m, 2F), −126.5 – −126.7 (m, 2F);
^{13}C{^{1}H,^{19}F} NMR (MeCN-d$_3$, 126 MHz, partial ^{13}C{^{19}F} decoupling observed) δ 156.9, 141.1, 134.4, 122.3, 119.3, 111.9, 111.8, 111.7, 111.6, 111.2, 110.1, 109.8, 108.6 – 108.3 (m);
HRMS (ESI): calcd. for $C_{16}H_4F_{17}N_2O$ [M−H]$^-$: 563.0058. Found 563.0057.

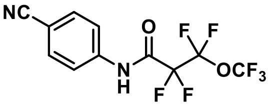

1i Prepared according to General Procedure F from perfluoro-3-methoxypropanoic acid (575 mg, 2.5 mmol, 1.0 equiv.), DMF (38.6 μL, 0.5 mmol, 0.2 equiv.), oxalyl chloride (215 μL, 2.5 mmol, 1.0 equiv.), 4-aminobenzonitrile (295 mg, 2.5 mmol, 1.0 equiv.), and NEt_3 (488 μL, 3.5 mmol, 1.4 equiv.) in anhydrous DCM (9.8 mL). The contents of flask 1 were stirred at room temperature for 3 h, and the combined contents in flask 2 were allowed to stir for 16 h at room temperature. Isolated in 28% yield (230 mg) as a white solid after purification by silica gel chromatography (15% EtOAc in hexanes, dry load).

^{1}H NMR ($CDCl_3$, 500 MHz) δ 8.28 (br s, 1H), 7.79 – 7.74 (m, 2H), 7.73 – 7.68 (m, 2H);
^{19}F NMR ($CDCl_3$, 470 MHz) δ −54.9 (t, *J* = 8.9 Hz, 3F), −87.1 (qt, *J* = 8.9, 2.5 Hz, 2F), −122.1 – −122.2 (m, 2F);
^{13}C{^{1}H,^{19}F} NMR ($CDCl_3$, 126 MHz, partial ^{13}C{^{19}F} decoupling observed) δ 155.8, 139.3, 133.7, 120.8, 120.7, 118.2, 115.2, 109.9, 107.3 – 106.8 (m);
HRMS (ESI): calcd. for $C_{11}H_4F_7N_2O_2$ [M−H]$^-$: 329.0166. Found 329.0165.

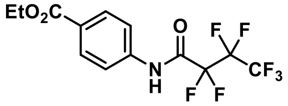

1j Prepared according to General Procedure D from benzocaine (330 mg, 2.0 mmol, 1.0 equiv.), DIPEA (310 mg, 2.4 mmol, 1.2 equiv.), and perfluorobutyryl chloride (299 μL, 2.0 mmol, 1.0 equiv.) in

anhydrous DCM (10 mL). The reaction mixture was allowed to stir for 16 h at room temperature. Isolated in 73% yield (522 mg) as a white solid after purification by silica gel chromatography (10% EtOAc in hexanes, dry load).

^{1}H NMR ($CDCl_3$, 500 MHz) δ 8.37 (br s, 1H), 8.10 – 8.02 (m, 2H), 7.72 – 7.64 (m, 2H), 4.37 (q, *J* = 7.1 Hz, 2H), 1.39 (t, *J* = 7.1 Hz, 3H);
^{19}F NMR ($CDCl_3$, 470 MHz) δ −80.5 (t, *J* = 8.8 Hz, 3F), −120.2 (q, *J* = 8.9 Hz, 2F), −126.7 (s, 2F);
^{13}C{^{1}H,^{19}F} NMR ($CDCl_3$, 126 MHz, partial ^{13}C{^{19}F} decoupling observed) δ 165.9, 155.6, 139.3, 131.1, 128.4, 120.1, 119.0 – 116.0 (m), 108.5, 108.4 – 108.3 (m), 61.4, 14.4;
HRMS (ESI): calcd. for $C_{13}H_9F_7NO_3$ $[M-H]^-$: 360.0476. Found 360.0467.

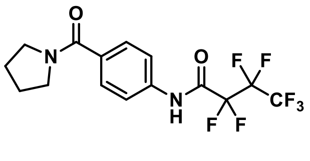

1k Prepared according to General Procedure D from (4-aminophenyl)(pyrrolidin-1-yl)methanone (380 mg, 2.0 mmol, 1.0 equiv.), DIPEA (310 mg, 2.4 mmol, 1.2 equiv.), and perfluorobutyryl chloride (299 μL, 2.0 mmol, 1.0 equiv.) in anhydrous DCM (10 mL). The reaction mixture was allowed to stir for 14 h at room temperature. Isolated in 55% yield (422 mg) as a white solid after purification by silica gel chromatography (66% EtOAc in hexanes, dry load).

^{1}H NMR ($CDCl_3$, 500 MHz) δ 9.04 (br s, 1H), 7.57 – 7.51 (m, 2H), 7.47 – 7.41 (m, 2H), 3.63 (t, *J* = 6.9 Hz, 2H), 3.41 (t, *J* = 6.8 Hz, 2H), 2.01 – 1.85 (m, 4H);
^{19}F NMR ($CDCl_3$, 470 MHz) δ −80.5 (t, *J* = 8.7 Hz, 3F), −120.1 (q, *J* = 8.7 Hz, 2F), −126.7 (s, 2F);
^{13}C{^{1}H,^{19}F} NMR ($CDCl_3$, 126 MHz, partial ^{13}C{^{19}F} decoupling observed) δ 169.0, 155.9, 136.9, 134.9, 128.2, 120.9, 119.0 – 116.1 (m), 108.6, 108.4, 49.8, 46.4, 26.5, 24.5;
HRMS (ESI): calcd. for $C_{15}H_{12}F_7N_2O_2$ $[M-H]^-$: 385.0792. Found 385.0798.

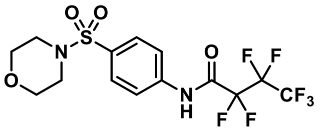

1l Prepared according to General Procedure D from 4-(morpholinosulfonyl)aniline (485 mg, 2.0 mmol, 1.0 equiv.), DIPEA (310 mg, 2.4 mmol, 1.2 equiv.), and perfluorobutyryl chloride (299 μL, 2.0 mmol, 1.0 equiv.) in anhydrous DCM (10 mL). The reaction mixture was allowed to stir for 16 h at room temperature. Isolated in 65% yield (572 mg) as an off-white solid after purification by silica gel chromatography (33% EtOAc in hexanes, dry load).

^{1}H NMR (MeCN-d_3, 500 MHz) δ 9.62 (br s, 1H), 7.92 – 7.87 (m, 2H), 7.81 – 7.76 (m, 2H), 3.68 – 3.64 (m, 4H), 2.95 – 2.90 (m, 4H);
^{19}F NMR (MeCN-d_3, 470 MHz) δ −81.4 (t, *J* = 8.8 Hz, 3F), −120.6 (q, *J* = 8.8 Hz, 2F), −127.4 (s, 2F);
^{13}C{^{1}H,^{19}F} NMR (MeCN-d_3, 126 MHz, partial ^{13}C{^{19}F} decoupling observed) δ 156.9, 141.3, 133.2, 130.1, 122.1, 119.0 – 117.1 (m), 109.6 – 109.4 (m), 109.3, 66.7, 47.1;
HRMS (ESI): calcd. for $C_{14}H_{12}F_7N_2O_4S$ $[M-H]^-$: 437.0411. Found 437.0417.

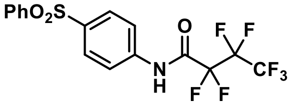

1m Prepared according to General Procedure E from 4-(phenylsulfonyl)aniline (700 mg, 3.0 mmol, 1.0 equiv.), NEt_3 (334 mg, 3.3 mmol, 1.1 equiv.), and heptafluorobutyric anhydride (809 μL, 3.3 mmol, 1.1 equiv.) in anhydrous DCM (6 mL). The reaction mixture was allowed to stir for 16 h at room temperature. Isolated in 81% yield (1.04 g) as a white solid after purification by silica gel chromatography (20% EtOAc in hexanes, dry load).

^{1}H NMR ($CDCl_3$, 500 MHz) δ 8.39 (br s, 1H), 7.97 – 7.89 (m, 4H), 7.78 – 7.72 (m, 2H), 7.61 – 7.56 (m, 1H), 7.54 – 7.48 (m, 2H);
^{19}F NMR ($CDCl_3$, 470 MHz) δ −80.4 (t, *J* = 8.9 Hz, 3F), 120.2 (q, *J* = 8.9 Hz, 2F), 126.6 (s, 2F);
^{13}C{^{1}H,^{19}F} NMR ($CDCl_3$, 126 MHz, partial ^{13}C{^{19}F} decoupling observed) δ 155.8, 141.3, 139.6, 139.0, 133.6, 129.6, 129.3, 127.7, 121.0, 118.8 – 116.0 (m), 108.4, 108.3 – 108.2 (m);
HRMS (ESI): calcd. for $C_{16}H_9F_7NO_2S$ $[M-H]^-$: 428.0197. Found 428.0189.

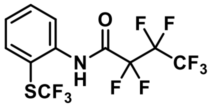

1n Prepared according to General Procedure D from 2-(trifluoromethylthio)aniline (386 mg, 2.0 mmol, 1.0 equiv.), DIPEA (310 mg, 2.4 mmol, 1.2 equiv.), and perfluorobutyryl chloride (299 μL, 2.0 mmol, 1.0 equiv.) in anhydrous DCM (10 mL). The reaction mixture was allowed to stir for 20 h at room temperature. Isolated in 65% yield (502 mg) as a white solid after purification by silica gel chromatography (5% EtOAc in hexanes, dry load).

^{1}H NMR ($CDCl_3$, 500 MHz) δ 9.12 (br s, 1H), 8.48 (d, *J* = 8.4 Hz, 1H), 7.76 (d, *J* = 7.9 Hz, 1H), 7.63 (td, *J* = 7.9, 1.2 Hz, 1H), 7.31 (td, *J* = 7.7, 1.1 Hz, 1H);
^{19}F NMR ($CDCl_3$, 470 MHz) δ −42.6 (s, 3F), −80.6 (t, *J* = 8.7 Hz, 3F), −120.5 (q, *J* = 8.7 Hz, 2F), −126.9 (s, 2F);
^{13}C{^{1}H,^{19}F} NMR ($CDCl_3$, 126 MHz, partial ^{13}C{^{19}F} decoupling observed) δ 155.3, 139.2, 138.9, 133.9, 130.0 – 127.3 (m), 126.8, 121.5, 118.8 – 116.2 (m), 113.4, 108.6, 108.5 – 108.3 (m);
HRMS (ESI): calcd. for $C_{11}H_4F_{10}NOS$ $[M-H]^-$: 387.9859. Found 387.9865.

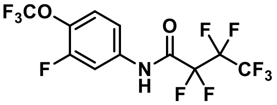

1o Prepared according to General Procedure D from 3-fluoro-4-(trifluoromethoxy)aniline (390 mg, 2.0 mmol, 1.0 equiv.), DIPEA (310 mg, 2.4 mmol, 1.2 equiv.), and perfluorobutyryl chloride (299 μL, 2.0 mmol, 1.0 equiv.) in anhydrous DCM (10 mL). The reaction mixture was allowed to stir for 20 h at room temperature. Isolated in 74% yield (582 mg) as a white solid after purification by silica gel chromatography (5% EtOAc in hexanes, dry load).

^{1}H NMR ($CDCl_3$, 500 MHz) δ 8.18 (br s, 1H), 7.68 (dd, *J* = 11.1, 2.5 Hz, 1H), 7.36 – 7.31 (m, 1H), 7.30 – 7.26 (m, 1H);
^{19}F NMR ($CDCl_3$, 470 MHz) δ −59.1 (d, *J* = 4.9 Hz, 3F), −80.6 (t, *J* = 8.7 Hz, 3F), −120.4 (q, *J* = 8.8 Hz, 2F), −124.8 – −124.9 (m, 1F), −126.7 (s, 2F);

$^{13}C\{^1H,^{19}F\}$ NMR ($CDCl_3$, 126 MHz, partial $^{13}C\{^{19}F\}$ decoupling observed) δ 155.7, 154.8, 134.9, 134.4, 124.6, 122.3 – 118.8 (m), 117.8 – 116.7 (m), 116.5, 110.3, 108.5, 108.4 – 108.2 (m);
HRMS (ESI): calcd. for $C_{12}H_3F_{13}NO$ $[M-H]^-$: 389.9994. Found 389.9993.

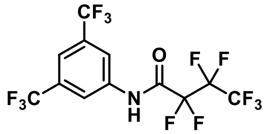

1p Prepared according to General Procedure D from 3,5-bis(trifluoromethyl)aniline (458 mg, 2.0 mmol, 1.0 equiv.), DIPEA (310 mg, 2.4 mmol, 1.2 equiv.), and perfluorobutyryl chloride (299 μL, 2.0 mmol, 1.0 equiv.) in anhydrous DCM (10 mL). The reaction mixture was allowed to stir for 16 h at room temperature. Isolated in 79% yield (671 mg) as a white solid after purification by silica gel chromatography (10% EtOAc in hexanes, dry load).

^1H NMR ($CDCl_3$, 500 MHz) δ 8.33 (br s, 1H), 8.10 (s, 2H), 7.77 (s, 1H);
^{19}F NMR ($CDCl_3$, 470 MHz) δ −63.2 (s, 6F), −80.5 (t, *J* = 8.8 Hz, 3F), −120.2 (q, *J* = 8.8 Hz, 2F), −126.6 (s, 2F);
$^{13}C\{^1H,^{19}F\}$ NMR ($CDCl_3$, 126 MHz, partial $^{13}C\{^{19}F\}$ decoupling observed) δ 155.9, 136.5, 133.6 – 132.6 (m), 123.9 – 121.5 (m), 120.5, 120.0, 118.6 – 115.8 (m), 108.3, 108.2 – 108.1 (m);
HRMS (ESI): calcd. for $C_{12}H_3F_{13}NO$ $[M-H]^-$: 424.0013. Found 424.0021.

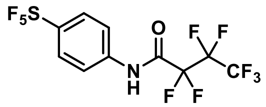

1q Prepared according to General Procedure D from 4-aminophenylsulfur pentafluoride (438 mg, 2.0 mmol, 1.0 equiv.), DIPEA (310 mg, 2.4 mmol, 1.2 equiv.), and perfluorobutyryl chloride (299 μL, 2.0 mmol, 1.0 equiv.) in anhydrous DCM (10 mL). The reaction mixture was allowed to stir for 16 h at room temperature. Isolated in 82% yield (677 mg) as a white solid after purification by silica gel chromatography (10% EtOAc in hexanes, dry load).

^1H NMR ($CDCl_3$, 500 MHz) δ 8.16 (br s, 1H), 7.82 – 7.77 (m, 2H), 7.72 – 7.67 (m, 2H);
^{19}F NMR ($CDCl_3$, 470 MHz) δ 84.4 – 82.9 (m, 1F), 63.3 – 62.8 (m, 4F), −80.5 (t, *J* = 8.8 Hz, 3F), −120.3 (q, *J* = 8.8 Hz, 2F), −126.7 (s, 2F);
$^{13}C\{^1H,^{19}F\}$ NMR ($CDCl_3$, 126 MHz, partial $^{13}C\{^{19}F\}$ decoupling observed) δ 155.8, 151.7 – 150.8 (m), 137.8, 127.9 – 127.3 (m), 120.3, 118.9 – 116.1 (m), 108.5, 108.4 – 108.2 (m);
HRMS (ESI): calcd. for $C_{10}H_4F_{12}NOS$ $[M-H]^-$: 413.9827. Found 413.9833.

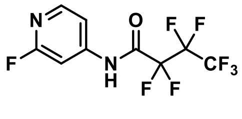

1r Prepared according to General Procedure D from 4-amino-2-fluoropyridine (168 mg, 1.5 mmol, 1.0 equiv.), DIPEA (233 mg, 1.8 mmol, 1.2 equiv.), and perfluorobutyryl chloride (224 μL, 1.5 mmol, 1.0 equiv.) in anhydrous DCM (8 mL). The reaction mixture was allowed to stir for 16 h at room temperature. Isolated in 69% yield (318 mg) as a colourless oil after purification by silica gel chromatography (20% EtOAc in hexanes, dry load).

^{1}H NMR ($CDCl_3$, 500 MHz) δ 8.87 (br s, 1H), 8.20 (d, *J* = 5.7 Hz, 1H), 7.41 (s, 1H), 7.39 (d, *J* = 5.7 Hz, 1H);
^{19}F NMR ($CDCl_3$, 470 MHz) δ −65.2 (s, 1F), −80.5 (t, *J* = 8.9 Hz, 3F), −120.2 (q, *J* = 8.9 Hz, 2F), −126.6 (s, 2F);
^{13}C{^{1}H,^{19}F} NMR ($CDCl_3$, 126 MHz, partial ^{13}C{^{19}F} decoupling observed) δ 165.8 – 163.8 (m), 156.3, 149.0 – 148.6 (m), 146.9, 118.7 – 116.1 (m), 112.5, 108.3, 108.2, 100.8 – 100.2 (m);
HRMS (ESI): calcd. for $C_9H_3F_8N_2O$ $[M-H]^-$: 307.0123. Found 307.0123.

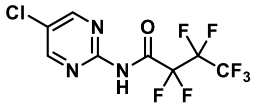

1s Prepared according to General Procedure D from 2-amino-5-chloropyrimidine (259 mg, 2.0 mmol, 1.0 equiv.), DIPEA (310 mg, 2.4 mmol, 1.2 equiv.), and perfluorobutyryl chloride (299 µL, 2.0 mmol, 1.0 equiv.) in anhydrous DCM (10 mL). The reaction mixture was allowed to stir for 16 h at room temperature. Isolated in 23% yield (145 mg) as a light brown solid after purification by silica gel chromatography (20% EtOAc in hexanes, dry load).

^{1}H NMR ($MeCN-d_3$, 500 MHz) δ 9.86 (br s, 1H), 8.71 (s, 2H);
^{19}F NMR ($MeCN-d_3$, 470 MHz) δ −81.4 (t, *J* = 8.9 Hz, 3F), −120.6 (q, *J* = 8.9 Hz, 2F), −127.3 (s, 2F);
^{13}C{^{1}H,^{19}F} NMR ($MeCN-d_3$, 126 MHz, partial ^{13}C{^{19}F} decoupling observed) δ 158.0, 155.9, 155.2, 128.8, 109.8 – 109.2 (m) 109.1;
HRMS (ESI): calcd. for $C_8H_2ClF_7N_3O$ $[M-H]^-$: 323.9780. Found 323.9772.

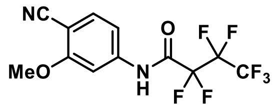

1t Prepared according to General Procedure E from 4-amino-2-methoxybenzonitrile (222 mg, 1.5 mmol, 1.0 equiv.), NEt_3 (167 mg, 1.65 mmol, 1.1 equiv.), and heptafluorobutyric anhydride (405 µL, 1.65 mmol, 1.1 equiv.) in anhydrous DCM (5 mL). The reaction mixture was allowed to stir for 16 h at room temperature. Isolated in 78% yield (401 mg) as a white solid after purification by silica gel chromatography (15% EtOAc in hexanes, dry load).

^{1}H NMR ($CDCl_3$, 500 MHz) δ 8.57 (br s, 1H), 7.62 (d, *J* = 1.7 Hz, 1H), 7.55 (d, *J* = 8.4 Hz, 1H), 7.12 (dd, *J* = 8.4, 1.7 Hz, 1H), 3.95 (s, 3H);
^{19}F NMR ($CDCl_3$, 470 MHz) δ −80.5 (t, *J* = 8.8 Hz, 3F), −120.0 (q, *J* = 8.8 Hz, 2F), −126.6 (s, 2F);
^{13}C{^{1}H,^{19}F} NMR ($CDCl_3$, 126 MHz, partial ^{13}C{^{19}F} decoupling observed) δ 162.4, 155.9, 141.2, 134.6, 119.0 – 116.2 (m), 116.1, 112.4, 108.4, 108.3, 103.6, 99.0, 56.4;
HRMS (ESI): calcd. for $C_{12}H_6F_7N_2O_2$ $[M-H]^-$: 343.0323. Found 343.0332.

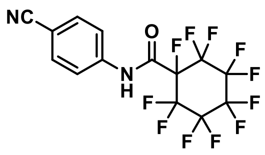

1u Prepared according to General Procedure D from 4-aminobenzonitrile (236 mg, 2.0 mmol, 1.0 equiv.), DIPEA (310 mg, 2.4 mmol, 1.2 equiv.), and perfluorocyclohexanecarbonyl fluoride (65% purity, 577 µL, 2.0 mmol, 1.0 equiv.) in anhydrous DCM (10 mL). The reaction mixture was allowed to stir for

16 h at room temperature. Isolated in 57% yield (486 mg) as an off-white solid after purification by silica gel chromatography (15% EtOAc in hexanes, dry load).

^{1}H NMR ($CDCl_3$, 500 MHz) δ 8.45 (br s, 1H), 7.79 – 7.75 (m, 2H), 7.73 – 7.68 (m, 2H);
^{19}F NMR ($CDCl_3$, 470 MHz) δ −118.5 – −119.4 (m, 2F), −122.2 – −123.7 (m, 3F), −130.5 – −131.6 (m, 2F), −137.8 – −138.8 (m, 2F), −140.2 – −141.3 (m, 1F), −178.9 – −179.2 (m, 1F);
^{13}C{^{1}H,^{19}F} NMR ($CDCl_3$, 126 MHz, partial ^{13}C{^{19}F} decoupling observed) δ 153.7, 139.4, 133.7, 120.9, 118.2, 109.9, 109.6 – 109.3 (m), 108.5 – 107.3 (m), 88.2 – 86.1 (m);
HRMS (ESI): calcd. for $C_{14}H_4F_{11}N_2O$ [M−H]$^-$: 425.0153. Found 425.0147.

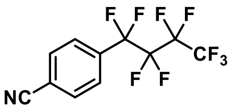

3a Prepared according to General Procedure G from 4-iodobenzonitrile (1.15 g, 5.0 mmol, 1.0 equiv.), copper powder (731 mg, 11.5 mmol, 2.3 equiv.), 2,2'-bipyridine (62.5 mg, 0.4 mmol, 0.08 equiv.), and nonafluoro-1-iodobutane (1.03 mL, 6.0 mmol, 1.2 equiv.) in anhydrous DMSO (8.0 mL). The reaction mixture was allowed to stir for 48 h at 80 °C. Isolated in 65% yield (1.05 g) as a colourless oil after purification by silica gel chromatography (5% EtOAc in hexanes, wet load).

^{1}H NMR ($CDCl_3$, 500 MHz) δ 7.83 (d, J = 8.4 Hz, 2H), 7.73 (d, J = 8.4 Hz, 2H);
^{19}F NMR ($CDCl_3$, 470 MHz) δ −81.0 (tt, J = 9.8, 2.6 Hz, 3F), −111.9 (t, J = 13.3 Hz, 2F), −122.5 – −122.7 (m, 2F), −125.5 – −125.6 (m, 2F);
^{13}C{^{1}H,^{19}F} NMR ($CDCl_3$, 126 MHz, partial ^{13}C{^{19}F} decoupling observed) δ 133.3, 132.6, 127.9, 117.5, 116.5, 116.4 – 116.0 (m), 115.1, 110.3, 109.3 – 108.7 (m);
HRMS (EI): calcd. for $C_{11}H_4F_9N$ [M·]$^+$: 321.0195. Found 321.0193.

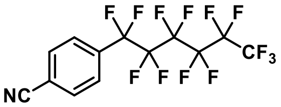

3b Prepared according to General Procedure G from 4-iodobenzonitrile (610 mg, 2.67 mmol, 1.0 equiv.), copper powder (359 mg, 5.6 mmol, 2.1 equiv.), 2,2'-bipyridine (31.2 mg, 0.2 mmol, 0.08 equiv.), and perfluorohexyl iodide (692 µL, 3.2 mmol, 1.2 equiv.) in anhydrous DMSO (4.0 mL). The reaction mixture was allowed to stir for 72 h at 80 °C. Isolated in 61% yield (688 mg) as a white solid after purification by silica gel chromatography (5% EtOAc in hexanes, wet load).

^{1}H NMR ($CDCl_3$, 500 MHz) δ 7.83 (d, J = 8.2 Hz, 2H), 7.74 (d, J = 8.2 Hz, 2H);
^{19}F NMR ($CDCl_3$, 470 MHz) δ −80.8 (tt, J = 9.8, 2.1 Hz, 3F), −111.6 (t, J = 14.6 Hz, 2F), −121.3 – −121.5 (m, 2F), −121.6 – −121.8 (m, 2F), −122.7 – −123.0 (m, 2F), −126.1 – −126.3 (m, 2F);
^{13}C{^{1}H,^{19}F} NMR ($CDCl_3$, 126 MHz, partial ^{13}C{^{19}F} decoupling observed) δ 133.4, 132.6, 127.9, 117.5, 116.5, 116.4 – 115.6 (m), 115.3, 111.3, 110.8, 110.4, 108.9 – 108.3 (m);
HRMS (EI): calcd. for $C_{13}H_4F_{13}N$ [M·]$^+$: 421.0131. Found 421.0145.

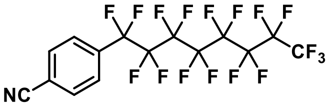

3c Prepared according to General Procedure G from 4-iodobenzonitrile (458 mg, 2.0 mmol, 1.0 equiv.), copper powder (267 mg, 4.2 mmol, 2.1 equiv.), 2,2'-bipyridine (25.0 mg, 0.16 mmol, 0.08 equiv.), and perfluorooctyl iodide (1.31 g, 2.4 mmol, 1.2 equiv.) in anhydrous DMSO (3.0 mL). The reaction mixture was allowed to stir for 72 h at 80 °C. Isolated in 22% yield (230 mg) as a white solid after purification by silica gel chromatography (17 → 25% $CHCl_3$ in hexanes, dry load).

^{1}H NMR ($CDCl_3$, 500 MHz) δ 7.83 (d, *J* = 8.3 Hz, 2H), 7.74 (d, *J* = 8.3 Hz, 2H);
^{19}F NMR ($CDCl_3$, 470 MHz) δ −80.8 (tt, *J* = 9.9, 2.0 Hz, 3F), −111.6 (t, *J* = 14.3 Hz, 2F), −121.0 – −121.3 (m, 2F), −121.5 – −121.7 (m, 2F), −121.7 – −122.0 (m, 4F), −122.6 – −122.9 (m, 2F), −126.0 – −126.2 (m, 2F);
^{13}C{^{1}H,^{19}F} NMR ($CDCl_3$, 126 MHz, partial ^{13}C{^{19}F} decoupling observed) δ 133.4, 132.6, 128.0, 117.5, 116.5, 116.2 – 115.8 (m), 115.3, 111.4, 110.9, 110.8, 110.4, 108.7 – 108.3 (m);
HRMS (EI): calcd. for $C_{15}H_4F_{17}N$ $[M\cdot]^+$: 521.0067. Found 521.0095.

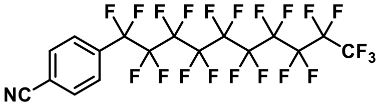

3d Prepared according to General Procedure G from 4-iodobenzonitrile (687 mg, 3.0 mmol, 1.0 equiv.), copper powder (400 mg, 6.3 mmol, 2.1 equiv.), 2,2'-bipyridine (37.5 mg, 0.24 mmol, 0.08 equiv.), and perfluorodecyl iodide (2.13 g, 3.3 mmol, 1.1 equiv.) in anhydrous DMSO (5.25 mL). The reaction mixture was allowed to stir for 72 h at 80 °C. Isolated in 28% yield (519 mg) as a white solid after purification by silica gel chromatography (15% EtOAc in hexanes, dry load) and subsequent recrystallization (EtOAc/pentane).

^{1}H NMR ($CDCl_3$, 600 MHz) δ 7.85 – 7.81 (m, 2H), 7.76 – 7.72 (m, 2H);
^{19}F NMR ($CDCl_3$, 565 MHz) δ −80.8 (tt, *J* = 9.6, 2.0 Hz, 3F), −111.6 (t, *J* = 14.5 Hz, 2F), −121.0 – −121.2 (m, 2F), −121.6 – −121.8 (m, 8F), −121.8 – −122.0 (m, 2F), −122.6 – −122.8 (m, 2F), −126.0 – −126.2 (m, 2F);
^{13}C{^{1}H,^{19}F} NMR ($CDCl_3$, 126 MHz, partial ^{13}C{^{19}F} decoupling observed) δ 133.4, 132.6, 128.0, 117.5, 116.5, 115.3, 111.4, 111.0 – 110.9 (m), 110.88, 110.84, 110.80, 110.3, 108.9 – 107.6 (m);
HRMS/LRMS: not found under a variety of ionization techniques (ESI, EI, CI, APCI, MALDI).

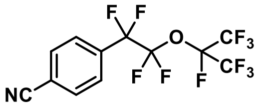

3e Prepared according to General Procedure G from 4-iodobenzonitrile (458 mg, 2.0 mmol, 1.0 equiv.), copper powder (267 mg, 4.2 mmol, 2.1 equiv.), 2,2'-bipyridine (25.0 mg, 0.16 mmol, 0.08 equiv.), and 2-iodotetrafluoroethyl heptafluoroisopropyl ether (989 mg, 2.4 mmol, 1.2 equiv.) in anhydrous DMSO (3.0 mL). The reaction mixture was allowed to stir for 48 h at 80 °C. Isolated in 47% yield (363 mg) as a colourless oil after purification by silica gel chromatography (5% EtOAc in hexanes, dry load).

^{1}H NMR ($CDCl_3$, 500 MHz) δ 7.81 (d, *J* = 8.2 Hz, 2H), 7.71 (d, *J* = 8.2 Hz, 2H);
^{19}F NMR ($CDCl_3$, 470 MHz) δ −80.5 – −80.6 (m, 6F), −84.8 – −85.0 (m, 2F), −115.4 – −115.6 (m, 2F), −145.2 (tt, *J* = 21.4, 2.0 Hz, 1F);

$^{13}C\{^{1}H,^{19}F\}$ NMR ($CDCl_3$, 126 MHz, partial $^{13}C\{^{19}F\}$ decoupling observed) δ 133.3, 132.6, 127.7, 119.3 – 118.5 (m), 117.5, 117.3, 116.4, 116.3 – 116.0 (m), 113.3;
HRMS (EI): calcd. for $C_{12}H_4F_{11}NO$ $[M\cdot]^+$: 387.0112. Found 387.0130.

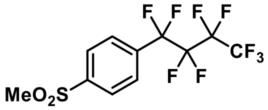

3f Prepared according to General Procedure G from 1-iodo-4-(methylsulfonyl)benzene (626 mg, 2.67 mmol, 1.0 equiv.), copper powder (359 mg, 5.6 mmol, 2.1 equiv.), 2,2'-bipyridine (31.2 mg, 0.2 mmol, 0.08 equiv.), and nonafluoro-1-iodobutane (551 µL, 3.2 mmol, 1.2 equiv.) in anhydrous DMSO (4.0 mL). The reaction mixture was allowed to stir for 48 h at 80 °C. Isolated in 81% yield (810 mg) as a white solid after purification by silica gel chromatography (100% DCM, wet load).

^{1}H NMR ($CDCl_3$, 500 MHz) δ 8.12 (d, *J* = 8.2 Hz, 2H), 7.83 (d, *J* = 8.2 Hz, 2H) 3.11 (s, 3H);
^{19}F NMR ($CDCl_3$, 470 MHz) δ −81.0 (tt, *J* = 9.8, 2.5 Hz, 3F), −111.4 (t, *J* = 13.5 Hz, 2F), −122.4 – −122.6 (m, 2F), −125.4 – −125.6 (m, 2F);
$^{13}C\{^{1}H,^{19}F\}$ NMR ($CDCl_3$, 126 MHz, partial $^{13}C\{^{19}F\}$ decoupling observed) δ 144.4, 134.3, 128.4, 128.0, 117.0 – 116.0 (m), 115.2, 110.3, 109.4 – 108.4 (m), 44.5;
HRMS (ESI): calcd. for $C_{11}H_7F_9NaO_2S$ $[M+Na]^+$: 396.9915. Found 396.9916.

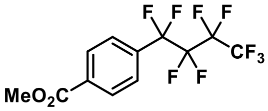

3g Prepared according to General Procedure G from methyl-4-iodobenzoate (786 mg, 3.0 mmol, 1.0 equiv.), copper powder (400 mg, 6.3 mmol, 2.1 equiv.), 2,2'-bipyridine (37.5 mg, 0.24 mmol, 0.08 equiv.), and nonafluoro-1-iodobutane (568 µL, 3.3 mmol, 1.1 equiv.) in anhydrous DMSO (5.25 mL). The reaction mixture was allowed to stir for 72 h at 80 °C. Isolated in 75% yield (789 mg) as a colourless oil after purification by silica gel chromatography (5% EtOAc in hexanes, dry load).

^{1}H NMR ($CDCl_3$, 600 MHz) δ 8.19 – 8.15 (m, 2H), 7.70 – 7.66 (m, 2H) 3.96 (s, 3H);
^{19}F NMR ($CDCl_3$, 565 MHz) δ −81.1 (tt, *J* = 9.8, 2.7 Hz, 3F), −111.5 (t, *J* = 13.3 Hz, 2F), −122.7 – −122.8 (m, 2F), −125.5 – −125.7 (m, 2F);
$^{13}C\{^{1}H,^{19}F\}$ NMR ($CDCl_3$, 126 MHz, partial $^{13}C\{^{19}F\}$ decoupling observed) δ 166.0, 133.7, 133.1, 130.0, 127.2, 117.6 (d, *J* = 272.5 Hz), 115.6 (t, *J* = 157.8 Hz), 110.4, 109.5 – 108.5 (m), 52.7;
LRMS (EI): calcd. for $C_{12}H_7F_9O_2$ $[M\cdot]^+$: 354.03. Found 354.05.

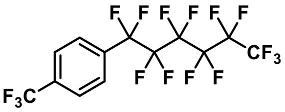

3h Prepared according to General Procedure G from 4-bromobenzotrifluoride (600 mg, 2.67 mmol, 1.0 equiv.), copper powder (359 mg, 5.6 mmol, 2.1 equiv.), 2,2'-bipyridine (31.2 mg, 0.2 mmol, 0.08 equiv.), and perfluorohexyl iodide (692 µL, 3.2 mmol, 1.2 equiv.) in anhydrous DMSO (4.0 mL). The

reaction mixture was allowed to stir for 48 h at 80 °C. Isolated in 72% yield (895 mg) as a colourless oil after purification by silica gel chromatography (100% hexanes, wet load).

^{1}H NMR ($CDCl_3$, 500 MHz) δ 7.82 (d, *J* = 8.3 Hz, 2H), 7.76 (d, *J* = 8.3 Hz, 2H);
^{19}F NMR ($CDCl_3$, 470 MHz) δ −63.4 (s, 3F), −80.9 (tt, *J* = 9.9, 2.1 Hz, 3F), −111.3 (t, *J* = 14.3 Hz, 2F), −121.3 – −121.6 (m, 2F), −121.7 – −121.9 (m, 2F), −122.7 – −123.0 (m, 2F), −126.1 – −126.3 (m, 2F);
^{13}C{^{1}H,^{19}F} NMR ($CDCl_3$, 126 MHz, partial ^{13}C{^{19}F} decoupling observed) δ 134.6 – 134.2 (m), 132.7, 127.7, 125.9, 122.6 – 122.2 (m), 118.7 – 116.0 (m), 115.5, 111.4, 110.9, 110.5, 108.8 – 108.4 (m);
LRMS (EI): calcd. for $C_{13}H_4F_{16}$ $[M\cdot]^+$: 464.01. Found 464.01.

6 Additional Reaction Optimization Data

a) Bromodefluorination of Perfluoroalkylamide 1a

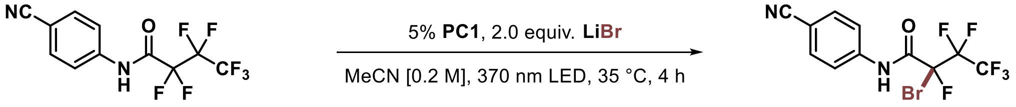

1a **2a**

variation to standard conditions

	yield **2a** (%)	conv. **1a** (%)
**no change** (*n* = 15, σ = 1.9)	**86**	**100**
390 nm LED instead of 370 nm	84	96
456 nm LED instead of 370 nm	22	34
2.5 mol% **PC1** instead of 5 mol%	84	100
with 5.0 equiv H_2O	65	80
reaction under air instead of N_2	33	56
no **PC1**	0	0
no 370 nm LED	0	0
no LiBr	0	28
starting from **2a** (stability test)	100	0 (**2a**)
additional 525 nm LED	84	100

bromine sources

	yield **2a** (%)	conv. **1a** (%)
NaBr	20	34
KBr	32	66
NH_4Br	25	51
TBABr	14	56
$NiBr_2$• glyme	35	49
trimethylsulfonium bromide	26	68
CBr_4	0	0
CH_2Br_2	0	30
NBS	0	0
Br_2	0	0

LiBr equiv.

	yield **2a** (%)	conv. **1a** (%)
0.5	41	65
1.0	85	94
1.5	83	100
2.5	85	100
3.0	84	100
3.5	86	100
4.0	86	100
6.0	83	100
8.0	85	90
10.0	50	49

solvents

	yield **2a** (%)	conv. **1a** (%)
DMSO	78	100
DMF	48	75
DMAc	39	100
acetone	20	83
DCE	48	72
NMP	0	100
MeOH	0	81
THF	0	100
MeCN [0.1 M]	84	100
MeCN [0.4 M]	81	100

additives (1.0 equiv.)

	yield **2a** (%)	conv. **1a** (%)
$Zn(OTf)_2$	3	27
$ZnCl_2$	39	53
$La(OTf)_3$	67	81
TEMPO	0	49
DIPEA	15	60
PPh_3	17	71
$NaIO_4$	10	81
HE	44	54

HE = Hantzsch Ester

photocatalyst variation (% yield 2a, % conversion 1a)

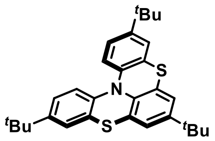

PC2 83% (100%)
370 nm LED
84% (100%)
370 nm + 525 nm LED

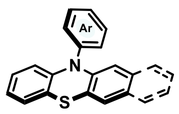

N-aryl-10*H*-phenothiazine
370 nm LED

Ar = 4-NO_2	**PC3**	0% (0%)
Ar = 4-SMe	**PC4**	76% (100%)
Ar = 4-CF_3	**PC5**	70% (90%)
Ar = Ph (PTH)	**PC6**	64% (82%)
Ar = 2-pyridyl-4-Me	**PC16**	46% (53%)
Phenyl-(benzo)PTH	**PC17**	40% (54%)

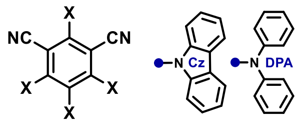

4CzIPN (X = Cz) **PC7** 0% (0%)
4DPAIPN (X = DPA) **PC8** 0% (0%)
456 nm LED

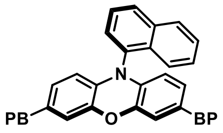

PC9 17% (20%)
456 nm LED

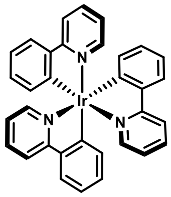

PC10 0% (0%)
370 nm LED

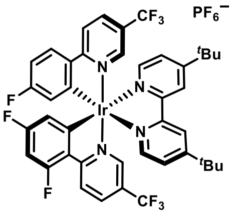

[Ir] PC11 0% (0%)
456 nm LED

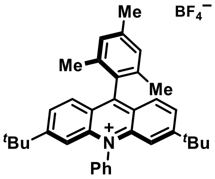

PC12 0% (0%)
456 nm LED

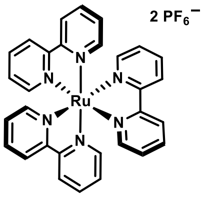

PC13 0% (0%)
456 nm LED

NaDT **PC14** 0% (0%) 370 nm LED

$Cu(dap)_2Cl$ **PC15** 0% (0%) 456 nm LED

b) Bromodefluorination of Perfluoroalkylarene 3a

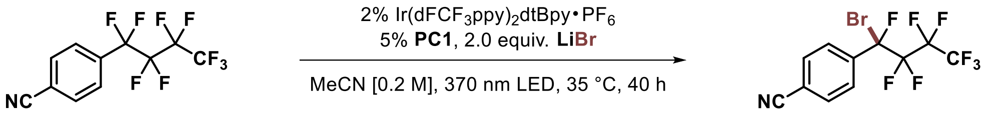

variation to standard conditions

	yield **4a** (%)	conv. **3a** (%)		yield **4a** (%)	conv. **3a** (%)
**no change**	**67**	**95**	with 5.0 equiv. H_2O	0	0
**PC2** instead of **PC1**	65	94	with 1.0 equiv. ^{t}BuOK	0	75
**PC3** instead of **PC1**	60	89	no light	0	0
**PC5** instead of **PC1**	62	95	no Ir(dFCF$_3$ppy)$_2$dtBpy•PF$_6$	51	94
390 nm LED instead of 370 nm	59	90	no **PC1**	0	0
456 nm LED instead of 370 nm	21	33	no LiBr	0	56
4.0 equiv. LiBr instead of 2.0 equiv.	60	95	reaction under air instead of N_2	0	18
6.0 equiv. LiBr instead of 2.0 equiv.	61	86	glassware not pre-dried	42	70
DMSO instead of MeCN	10	91			

Comment on the role of Ir[dFCF$_3$ppy]$_2$(dtbpy)PF$_6$: although the reaction delivers appreciable amounts of **4a** in the absence of Ir (51% ^{19}F NMR yield), this co-photocatalyst may act as a supplemental bromide oxidant (*[Ir]^{III} E^{red} 1.21 V vs. SCE) and **PC1$^{\cdot+}$** reductant ([Ir]^{II} E^{ox} –1.37 V vs. SCE).

c) Chlorodefluorination of Perfluoroalkylamide 1a

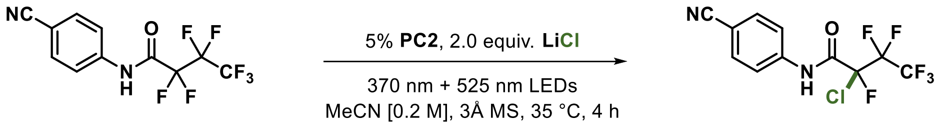

variation to standard conditions

	yield **5a** (%)	conv. **1a** (%)		yield **5a** (%)	conv. **1a** (%)
**no change** (*n* = 3)	**70**	**99**	1.2 equiv. LiCl instead of 2.0	60	92
standard conditions from Fig. 2	37	75	NaCl instead of LiCl	11	58
**PC1** instead of **PC2**	39	72	KCl instead of LiCl	9	62
**PC6** (PTH) instead of **PC2**	26	67	TBACl instead of LiCl	13	66
without 525 nm LED	40	72	NCS instead of LiCl	0	39
456 nm LED instead of 525 nm	17	80	acetone instead of MeCN	44	90
without 3Å MS (*n* = 3)	60	87	DMSO instead of MeCN	18	74
2 equiv. H_2O instead of 3Å MS	29	84	DMF instead of MeCN	0	100

d) Iododefluorination of Perfluoroalkylamide 1a

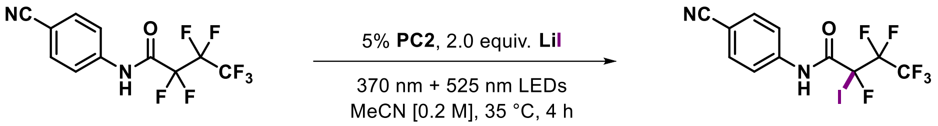

1a → **6a**

variation to standard conditions

	yield **6a** (%)	conv. **1a** (%)
**no change**	trace (<1)	10
TBAI + **PC1** instead of LiI + **PC2**	7	26
**TBAI instead of LiI**	10	27
456 nm instead of 370 nm	0	0
*reactions below with **PC1**, only 370 nm light*		
TBAI instead of LiI	11	39
NIS instead of LiI	0	0
DIH instead of LiI	0	0
I_2 instead of LiI	0	0
NaI instead of LiI	0	28
12.0 equiv. LiI instead of 2.0	0	17

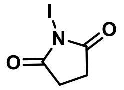

NIS

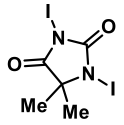

DIH

e) Comment on Reaction Mass Balance

side products identified under the standard conditions

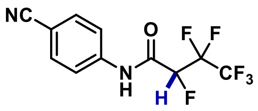

s1 (from **1a**)
monohydrodefluorination
general procedure C

identified by ^{19}F NMR
identified by HRMS

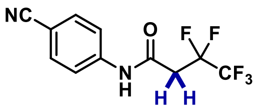

s2 (from **1a**)
dihydrodefluorination
general procedure C

identified by ^{19}F NMR
identified by HRMS

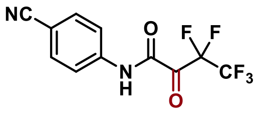

s3 (from **1a**)
α-oxidation
general procedure A, C

identified by ^{19}F NMR
identified by HRMS

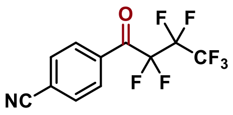

s4 (from **3a**)
benzylic oxidation
general procedure B

identified by ^{19}F NMR

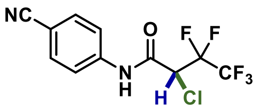

s5 (from **1a**)
hydrochlorodefluorination
general procedure C

identified by HRMS

side products not observed under the standard conditions

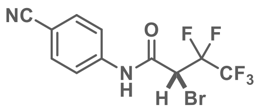

s6 (from **1a**)
hydrobromodefluorination
general procedure A

not identified by HRMS
not identified by ^{19}F NMR

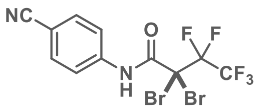

s7 (from **1a**)
dibromodefluorination
general procedure A

not identified by HRMS
not identified by ^{19}F NMR

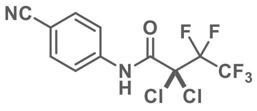

s8 (from **1a**)
dichlorodefluorination
general procedure C

not identified by HRMS
not identified by ^{19}F NMR

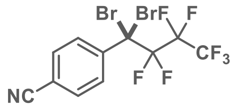

s9 (from **3a**)
dibromodefluorination
general procedure B

not identified by HRMS
not identified by ^{19}F NMR

s1 – ^{19}**F NMR** ($CDCl_3$, 376 MHz) δ −82.3 (d, *J* = 10.6 Hz, 3F), −121.9 – −122.7 (m, 1F), −127.5 – −128.4 (m, 1F), −203.1 – −203.4 (m, 1F);
HRMS (ESI): calcd. for $C_{11}H_5F_6N_2O$ $[M-H]^-$: 295.0312. Found 295.0342.

s2 – ^{19}**F NMR** ($CDCl_3$, 376 MHz) δ −85.4 (s, 3F), −115.4 (t, *J* = 17.7 Hz, 2F);
HRMS (ESI): calcd. for $C_{11}H_6F_5N_2O$ $[M-H]^-$: 277.0406. Found 277.0430.

s3 – ^{19}**F NMR** ($CDCl_3$, 376 MHz) δ −79.4 (m, 3F), 125.4 (s, 2F);
HRMS (ESI): calcd. for $C_{11}H_4F_5N_2O_2$ $[M-H]^-$: 291.0198. Found 291.0221.

s4 – ^{19}**F NMR** ($CDCl_3$, 376 MHz) δ −80.5 (t, *J* = 11.0 Hz, 3F), −113.6 – −113.7 (m, 2F), 125.4 (s, 2F);

s5 – **HRMS (ESI)**: calcd. for $C_{11}H_5[^{35}Cl]F_5N_2O$ $[M-H]^-$: 311.0016. Found 311.0028.

Discussion: side products observed for General Procedures A, B, and C are discussed below.

General Procedure A (Bromodefluorination of 1a): under the standard conditions, the total mass balance at 4 h is typically 85–90% (100% conv. **1a**, ~85% **2a**). The only identifiable side product was **s3**, which is usually formed in 1–2% yield. We believe the loss of the remaining mass balance (~10%) could be due to the formation of radical polymerization products. Hydrobromodefluorination and dibromodefluorination side products **s6**–**7** were not observed under the standard conditions.

General Procedure B (Bromodefluorination of 3a): under the standard conditions, the total mass balance at 40 h is typically 70–75% (~95% conv. **3a**, ~65% **4a**). The only identifiable side product was **s4**, which is usually formed in 1–2% yield. We believe the loss of the remaining mass balance (~30%) could be due to the formation of radical polymerization products. Dibromodefluorination side product **s9** was not observed under the standard conditions.

General Procedure C (Chlorodefluorination of 1a): under the standard conditions, the total mass balance at 4 h is typically 80–85% (100% conv. **1a**, ~70% **5a**). Monohydrodefluorination side product **s1** is typically formed in 5–10% yield. Dihydrodefluorination side product **s2** is typically formed in 2–5% yield. Oxidation side product **s3** is typically formed in 1–2% yield, while hydrochlorodefluorination side product **s5** is only detected in trace amounts by HRMS. We believe the loss of the remaining mass balance (~15%) could be due to the formation of radical polymerization products. Dichlorodefluorination side product **s8** was not observed under the standard conditions.

7 Less Successful Scope Examples

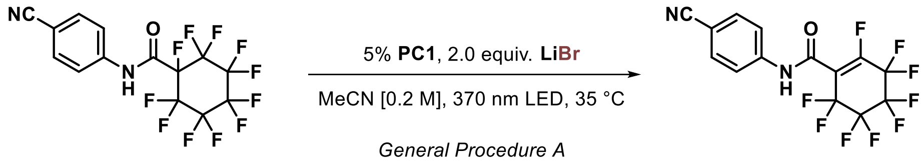

1u

2u 57% (**47%**)

2u Prepared according to General Procedure A in a 1-dram vial from substrate **1u** (85.2 mg, 0.20 mmol, 1.0 equiv.), photocatalyst **PC1** (3.4 mg, 0.01 mmol, 0.05 equiv.), and LiBr (34.7 mg, 0.40 mmol, 2.0 equiv.) in anhydrous MeCN (1.0 mL). The reaction mixture was allowed to stir for 21 h under UV irradiation (370 nm) using the two light setup. ^{19}F NMR yield: 57%. Isolated in 47% yield (36.1 mg) as an off-white solid after purification by silica gel chromatography (15 → 20% EtOAc in hexanes, dry load).

^{1}H NMR ($CDCl_3$, 500 MHz) δ 8.32 (br s, 1H), 7.78 – 7.73 (m, 2H), 7.72 – 7.67 (m, 2H);
^{19}F NMR ($CDCl_3$, 470 MHz) δ −108.4 – −108.7 (m, 2F), −117.2 – −117.4 (m, 1F), −119.3 – −119.6 (m, 2F), −133.6 – −133.8 (m, 2F), −133.9 – −134.1 (m, 2F);
^{13}C{^{1}H,^{19}F} NMR ($CDCl_3$, 126 MHz, partial ^{13}C{^{19}F} decoupling observed) δ 153.1, 151.5, 140.2, 133.6, 120.5, 118.2, 115.4, 110.2, 109.0, 107.8 – 107.2 (m), 107.1;
HRMS (ESI): calcd. for $C_{14}H_4F_9N_2O$ [M−H]$^-$: 387.0185. Found 387.0191.

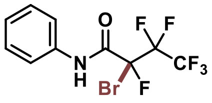

2v 15% (29% conv.)
General Procedure A

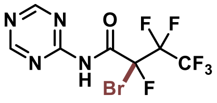

2w 23% (75% conv.)
General Procedure A

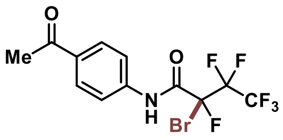

2x 25% (72% conv.)
General Procedure A

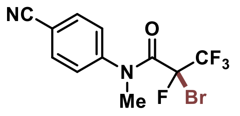

2y 0% (27% conv.)
General Procedure A

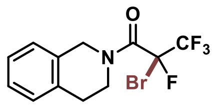

2z 0% (15% conv.)
General Procedure A

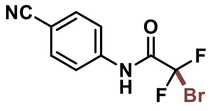

2aa 0% (0% conv.)
General Procedure A

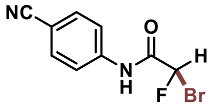

2ab 32% (52% conv.)
General Procedure A

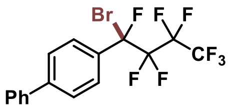

4i 13% (81% conv.)
General Procedure B

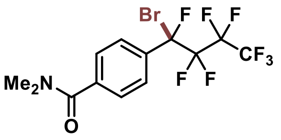

4j 18% (43% conv.)
General Procedure B

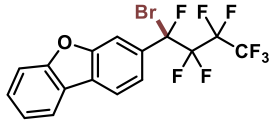

4k 20% (85% conv.)
General Procedure B

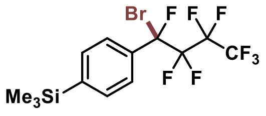

4l 13% (81% conv.)
General Procedure B

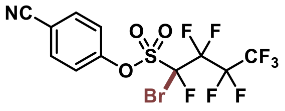

7a 0% (73% conv.)
General Procedure A

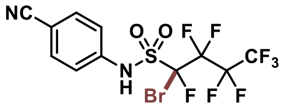

7b 0% (0% conv.)
General Procedure A

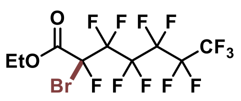

7c 0% (0% conv.)
General Procedure A

8 Reaction Kinetics & Mechanistic Experiments

General Information for All Kinetic Experiments: ^{19}F NMR kinetic analysis (*ex situ*) was performed using a Bruker AVIII HD 600 MHz spectrometer. Substrate conversion and product yield were determined by removing a small aliquot (40 μL) of the reaction and placing it into 0.60 mL $CDCl_3$ for calibrated ^{19}F NMR analysis (D_1 = 30 s, O1p = -100.0 ppm), using FNP as the reference signal. This was done by preparing a stock solution of FNP (81.3 mg) in $CDCl_3$ (3.0 mL) and adding a 40 μL aliquot of this solution to the $CDCl_3$ solution containing the reaction aliquot (corresponding to a 1:1 integration between starting material or product (1 fluorine) and the FNP internal standard signal at −123.5 ppm). The small differences in reaction volume for the following experiments, where the concentration of a single component was systematically varied relative to the standard conditions, were accounted for on an individual experiment basis for the internal standard stock solution calculation. FNP must be added directly to the reaction aliquot, as it is not stable under the optimized reaction conditions. Sampling time was not counted towards the total reaction time (no reactivity is observed without an LED source, see **Section 8b** below for an intermittent illumination experiment). RPKA and VTNA was performed as reported in the literature; different excess experiments were limited to <80% conversion of **1a** given the observed product inhibition, and the orders in each component were determined by the best visual overlap of the kinetic curves (to the nearest single decimal place).[15–16]

a) NMR Reaction Kinetics & Variable Time Normalization Analysis (VTNA)
[i] Standard Reaction Conditions

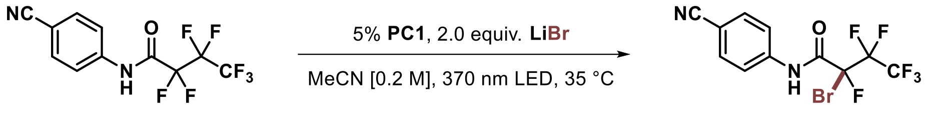

1a (1.0 mmol) **2a** 86%

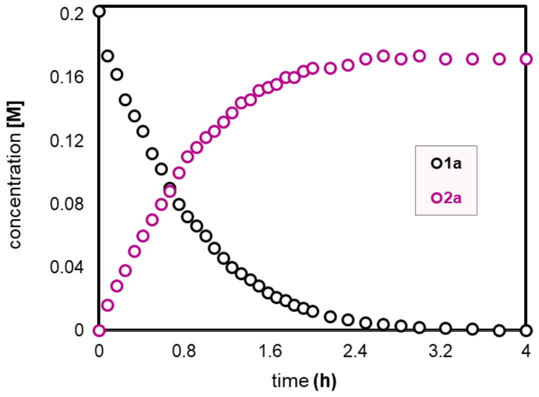

The experiment followed General Procedure A in a 4-dram vial using substrate **1a** (314.2 mg, 1.0 mmol, 1.0 equiv.), photocatalyst **PC1** (16.8 mg, 0.05 mmol, 0.05 equiv.), and LiBr (173.7 mg, 2.0 mmol, 2.0 equiv.) in anhydrous MeCN (5.0 mL). The reaction mixture was stirred under UV irradiation (370 nm) using the two light setup. The reaction mixture was sampled at the following times (minutes): 0, 5, 10, 15, 20, 25, 30, 35, 40, 45, 50, 55, 60, 65, 70, 75, 80, 85, 90, 95, 100, 105, 110, 115, 120, 130, 140, 150, 160, 170, 180, 195, 210, 225, 240. Terminal ^{19}F NMR yield **2a**: 86%.

[ii] Product Inhibition & Catalyst Degradation: Same Excess Experiments

Note: *plots below for **Exp 1** (standard conditions) were generated using the data collected above in **Section 8a-[i]**.*

*Same excess **without** added product (**2a**)*

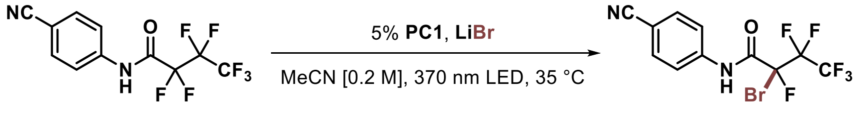

1a **2a**

Exp 1 (std. cond.)	
**1a**	0.2 M
**PC1**	0.01 M
**LiBr**	0.4 M
**2a**	0.0 M

Exp 2 (same x.s.)	
**1a**	0.09 M
**PC1**	0.01 M
**LiBr**	0.29 M
**2a**	0.0 M

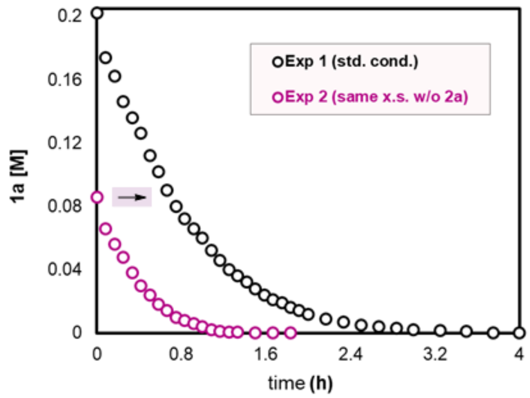

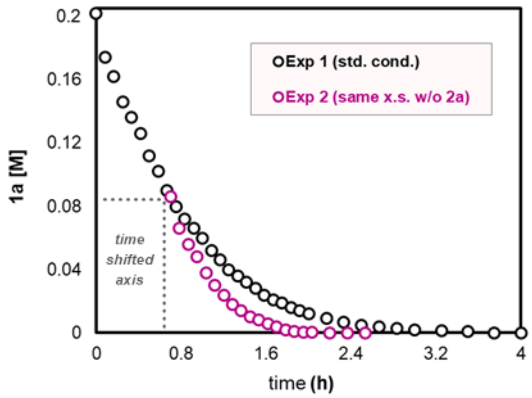

The experiment (**Exp 2**) followed General Procedure A in a 4-dram vial using substrate **1a** (141.4 mg, 0.45 mmol, 0.45 equiv.), photocatalyst **PC1** (16.8 mg, 0.05 mmol, 0.05 equiv.), and LiBr (125.9 mg, 1.45 mmol, 1.45 equiv.) in anhydrous MeCN (5.0 mL). The reaction mixture was stirred under UV irradiation (370 nm) using the two light setup. The reaction mixture was sampled at the following times (minutes, including a 40 minute time shift relative to T_0): 40, 45, 50, 55, 60, 65, 70, 75, 80, 85, 90, 95, 100, 105, 110, 115, 120, 130, 140, 150. Terminal ^{19}F NMR yield **2a**: 36%.

Discussion: this same excess experiment is designed to mimic the reaction after 40 min (55% conversion of **1a**) *without* any product that would be normally be present at that time. No overlay between the substrate conversion curves of the same excess experiment (**Exp 2**) and the standard conditions (**Exp 1**) was observed when applying a time shift, suggesting product inhibition and/or catalyst degradation is present under the standard conditions.

*Same excess **with** added product (**2a**)*

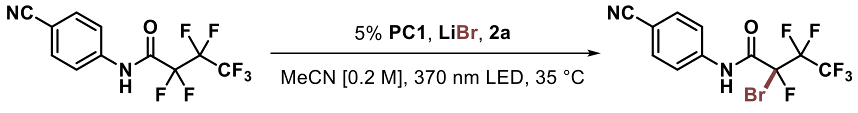

1a **2a**

Exp 1 (std. cond.)	
**1a**	0.2 M
**PC1**	0.01 M
**LiBr**	0.4 M
**2a**	0.0 M

Exp 3 (same x.s.)	
**1a**	0.09 M
**PC1**	0.01 M
**LiBr**	0.29 M
**2a**	0.088 M

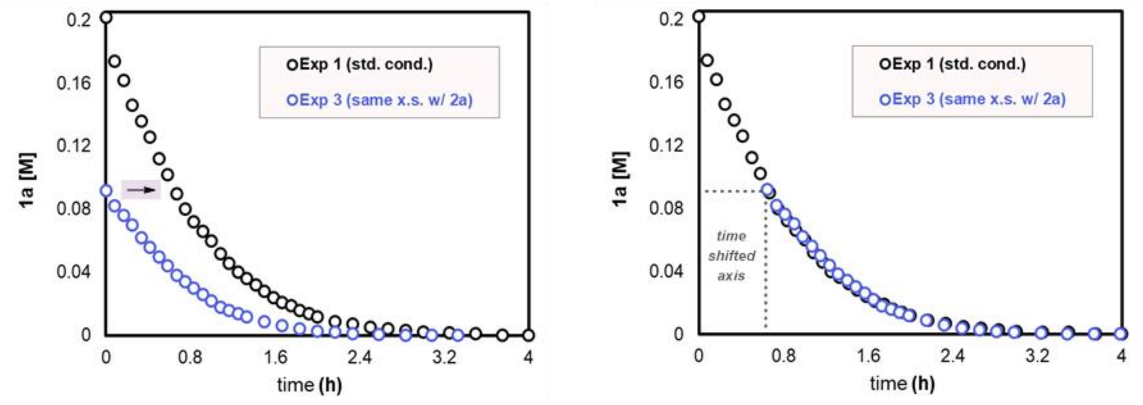

The experiment (**Exp 3**) followed General Procedure A in a 4-dram vial using substrate **1a** (141.4 mg, 0.45 mmol, 0.45 equiv.), photocatalyst **PC1** (16.8 mg, 0.05 mmol, 0.05 equiv.), LiBr (125.9 mg, 1.45 mmol, 1.45 equiv.), and product **2a** (165.0 mg, 0.44 mmol, 0.44 equiv.) in anhydrous MeCN (5.0 mL). The reaction mixture was stirred under UV irradiation (370 nm) using the two light setup. The reaction mixture was sampled at the following times (minutes, including a 40 minute time shift relative to T_0): 40, 45, 50, 55, 60, 65, 70, 75, 80, 85, 90, 95, 100, 105, 110, 115, 120, 130, 140, 150, 160, 170, 180, 195, 210. Terminal ^{19}F NMR yield **2a**: 77%.

Discussion: this same excess experiment is designed to mimic the reaction after 40 min (55% conversion of **1a**) *with* the amount of product that would normally be present at that time (44% or 0.088 M). Excellent overlay between the substrate conversion curves of the same excess experiment (**Exp 3**) and the standard conditions (**Exp 1**) was observed when applying a time shift, suggesting product inhibition is present under the standard conditions, while no detectable catalyst deactivation is present.

[iii] Order in Light (370 nm LED)

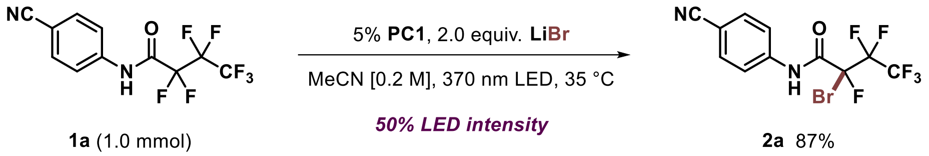

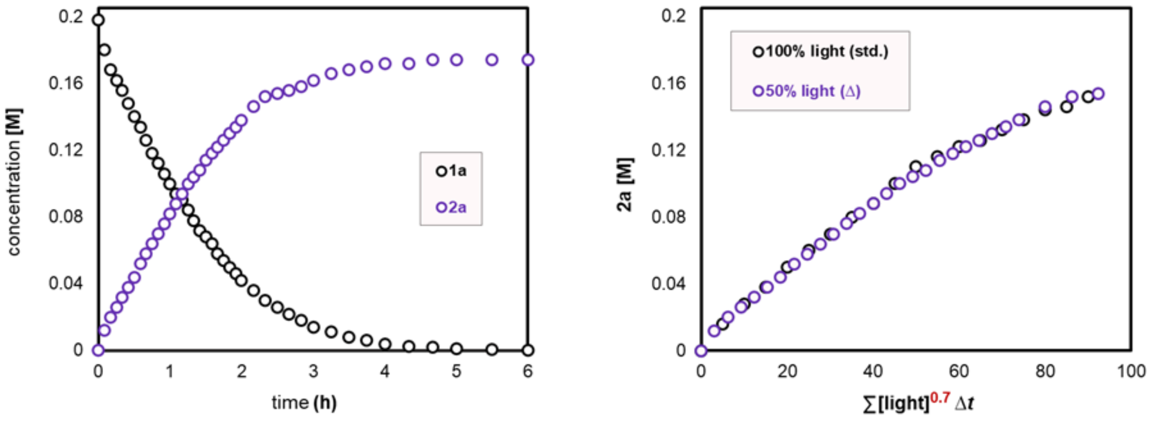

The experiment followed General Procedure A in a 4-dram vial using substrate **1a** (314.2 mg, 1.0 mmol, 1.0 equiv.), photocatalyst **PC1** (16.8 mg, 0.05 mmol, 0.05 equiv.), and LiBr (173.7 mg, 2.0 mmol, 2.0 equiv.) in anhydrous MeCN (5.0 mL). The reaction mixture was stirred under UV irradiation (370 nm, 50% light intensity) using the two light setup. The reaction mixture was sampled at the following times (minutes): 0, 5, 10, 15, 20, 25, 30, 35, 40, 45, 50, 55, 60, 65, 70, 75, 80, 85, 90, 95, 100, 105, 110, 115, 120, 130, 140, 150, 160, 170, 180, 195, 210, 225, 240, 260, 280, 300, 330, 360. Terminal ^{19}F NMR yield **2a**: 87%.

Discussion: optimal visual overlap of the VTNA curves is achieved when the order of light is set to 0.7. While conPET processes typically display a biphotonic dependence in light, pseudo-monophotonic dependence can occur when a photocatalyst exhibits saturation kinetics.[17] Our system displays near-saturation kinetics in **PC1** (see below), which may contribute to the apparent pseudo-monophotonic dependence.[17] Given the partial order obtained, we aimed to be conservative with our conclusions based on the light order, apart from proposing that the rate-determining step involves the absorption of photons.

[iv] Order in Photocatalyst (PC1)

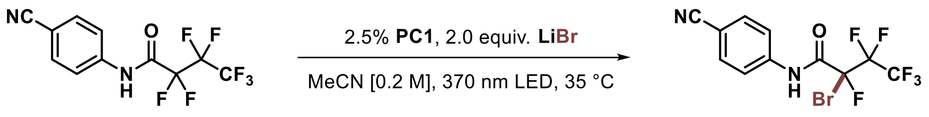

1a (1.0 mmol) **2a** 89%

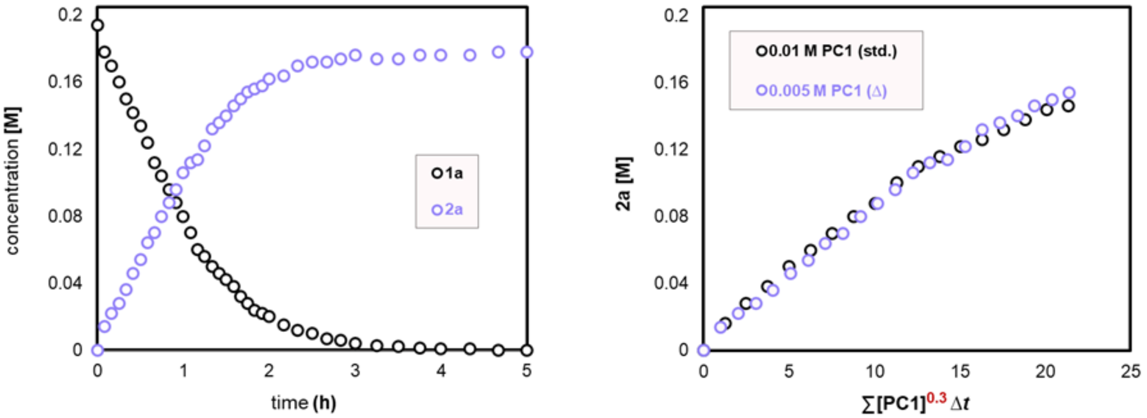

The experiment followed General Procedure A in a 4-dram vial using substrate **1a** (314.2 mg, 1.0 mmol, 1.0 equiv.), photocatalyst **PC1** (8.4 mg, 0.025 mmol, 0.025 equiv.), and LiBr (173.7 mg, 2.0 mmol, 2.0 equiv.) in anhydrous MeCN (5.0 mL). The reaction mixture was stirred under UV irradiation (370 nm) using the two light setup. The reaction mixture was sampled at the following times (minutes): 0, 5, 10, 15, 20, 25, 30, 35, 40, 45, 50, 55, 60, 65, 70, 75, 80, 85, 90, 95, 100, 105, 110, 115, 120, 130, 140, 150, 160, 170, 180, 195, 210, 225, 240, 260, 280, 300. Terminal ^{19}F NMR yield **2a**: 89%.

Discussion: optimal visual overlap of the VTNA curves is achieved when the order of **PC1** is set to 0.3. Considering the large positive order in light and the computationally proposed mechanism, this result suggests that **PC1** is involved in the rate-determining step, yet it exhibits near-saturation kinetics, which ultimately gives a small positive order for the majority of the reaction.

[v] Order in Product (2a)

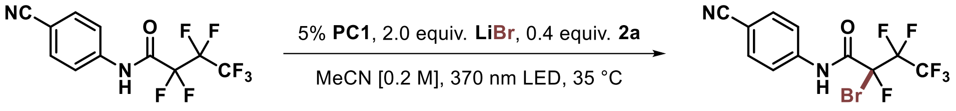

1a (1.0 mmol) **2a** 82%

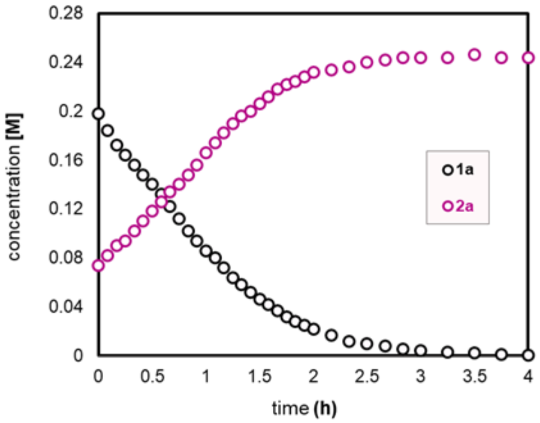

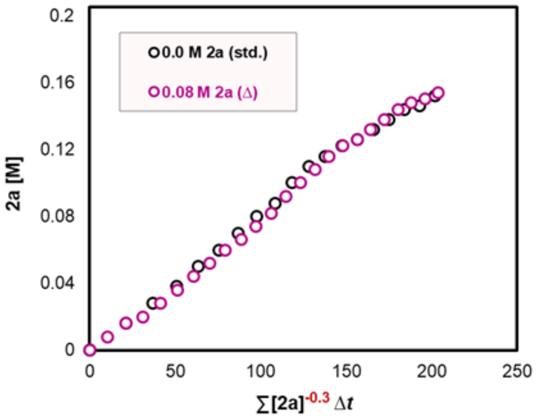

The experiment followed General Procedure A in a 4-dram vial using substrate **1a** (314.2 mg, 1.0 mmol, 1.0 equiv.), photocatalyst **PC1** (16.8 mg, 0.05 mmol, 0.05 equiv.), LiBr (173.7 mg, 2.0 mmol, 2.0 equiv.), and product **2a** (150.0 mg, 0.40 mmol, 0.40 equiv.) in anhydrous MeCN (5.0 mL). The reaction mixture was stirred under UV irradiation (370 nm) using the two light setup. The reaction mixture was sampled at the following times (minutes): 0, 5, 10, 15, 20, 25, 30, 35, 40, 45, 50, 55, 60, 65, 70, 75, 80, 85, 90, 95, 100, 105, 110, 115, 120, 130, 140, 150, 160, 170, 180, 195, 210, 225, 240. Terminal ^{19}F NMR yield **2a**: 82% (corrected for the amount of **2a** present at the start of the reaction).

Discussion: in order to obtain overlay of the product curves, the y-axis of this experiment was shifted downwards by 0.08 M, since this amount of **2a** was already present at T_0. Optimal visual overlap of the VTNA curves is achieved when the order of **2a** is set to −0.3. Alternatively, VTNA can be performed on the curve of **1a**, resulting in the same order of −0.3. This corroborates the inhibition effect observed in the same excess experiments, where the reaction rate begins to diminish as more product is formed. Furthermore, the slight negative order fits our hypothesis of photon absorption being rate-limiting as described above, since when more product is present, more photons follow the unproductive reaction pathway (**PC1** quenching by **2a**).

[vi] Order in Substrate (1a)

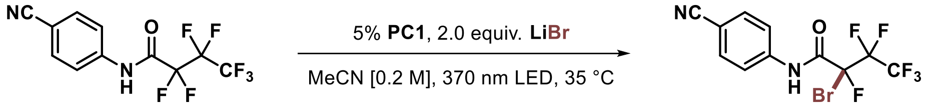

1a (0.60 mmol, 0.60 equiv.) **2a** 82%

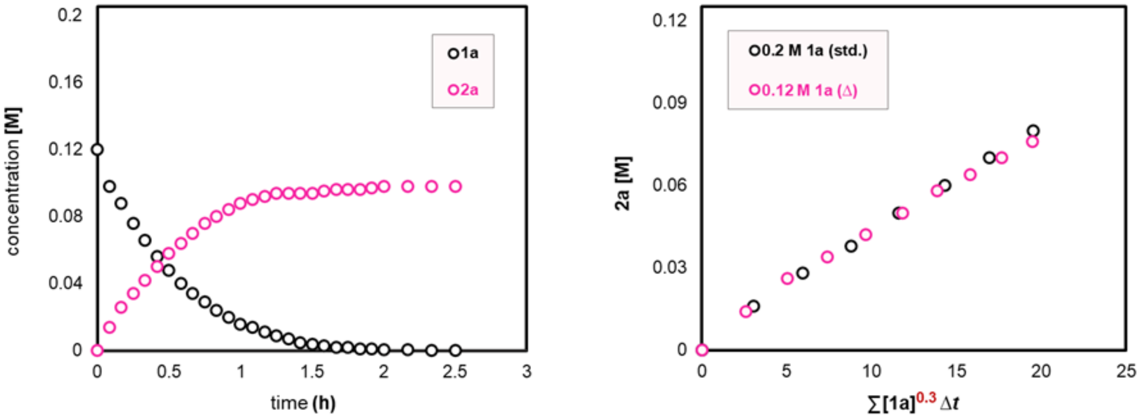

The experiment followed General Procedure A in a 4-dram vial using substrate **1a** (188.5 mg, 0.60 mmol, 0.6 equiv.), photocatalyst **PC1** (16.8 mg, 0.05 mmol, 0.05 equiv.), and LiBr (173.7 mg, 2.0 mmol, 2.0 equiv.) in anhydrous MeCN (5.0 mL). The reaction mixture was stirred under UV irradiation (370 nm) using the two light setup. The reaction mixture was sampled at the following times (minutes): 0, 5, 10, 15, 20, 25, 30, 35, 40, 45, 50, 55, 60, 65, 70, 75, 80, 85, 90, 95, 100, 105, 110, 115, 120, 130, 140, 150. Terminal ^{19}F NMR yield **2a**: 82% (corrected for the amount of **1a** present at the start of the reaction).

Discussion: due to the lower starting concentration of **1a**, VTNA was performed only for the early stages of the reaction (i.e. <0.09 M **2a**). Optimal visual overlap of the VTNA curves is achieved when the order of **1a** is set to 0.3. Analogous to the small negative order in product **2a**, the small positive order in substrate **1a** befits a rate-determining step where photon absorption is limiting. In this case, a higher concentration of **1a** increases the likelihood that the absorbed photons would result in a productive pathway towards the formation of **2a**.

[vii] Order in LiBr

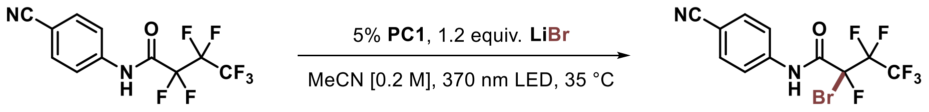

1a (1.0 mmol) **2a** 85%

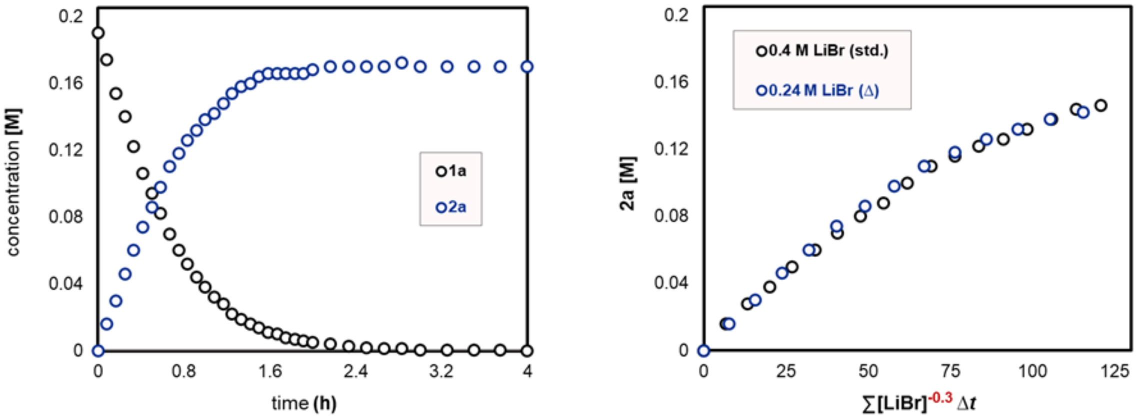

The experiment followed General Procedure A in a 4-dram vial using substrate **1a** (314.2 mg, 1.0 mmol, 1.0 equiv.), photocatalyst **PC1** (16.8 mg, 0.05 mmol, 0.05 equiv.), and LiBr (104.2 mg, 1.2 mmol, 1.2 equiv.)

in anhydrous MeCN (5.0 mL). The reaction mixture was stirred under UV irradiation (370 nm) using the two light setup. The reaction mixture was sampled at the following times (minutes): 0, 5, 10, 15, 20, 25, 30, 35, 40, 45, 50, 55, 60, 65, 70, 75, 80, 85, 90, 95, 100, 105, 110, 115, 120, 130, 140, 150, 160, 170, 180, 195, 210, 225, 240. Terminal ^{19}F NMR yield **2a**: 85%.

Discussion: optimal visual overlap of the VTNA curves is achieved when the order of LiBr is set to −0.3. The partial negative order may be a result of H_2O absorption by the hygroscopic LiBr salt, as H_2O was shown to slow the rate of substrate conversion and product formation (see **Section 6a** for additional optimization experiments).

[viii] Normalization Incorporating All Reaction Components

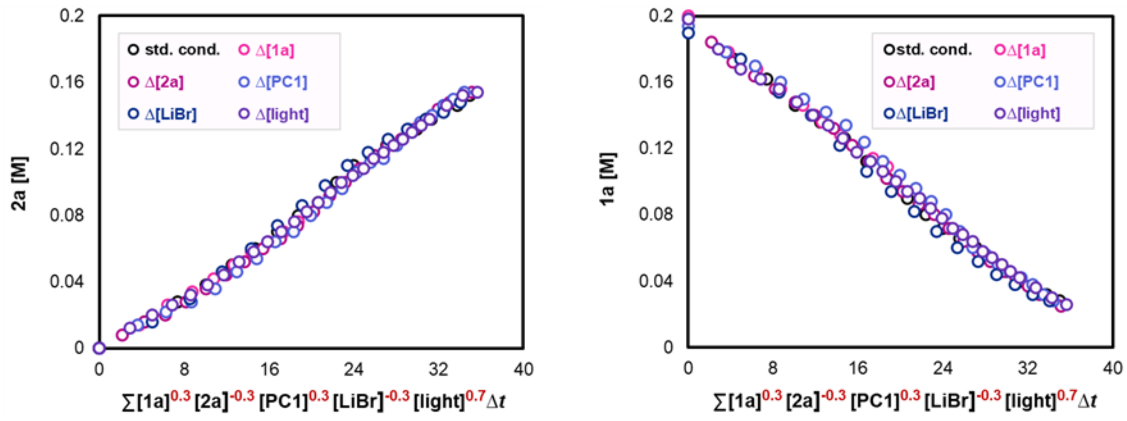

Discussion: the VTNA timescale utilizing the orders obtained in the above experiments plotted against either the concentration of product **2a** or substrate **1a** both lead to good overlay of all different excess curves. Overall, the positive order in light source power (0.7) in combination with lower orders for all other reaction constituents (≤0.3) suggests photon absorption is predominantly rate-determining, with the orders in **PC1** and **1a** being slightly positive (assisting a productive pathway after photon absorption), and those in **2a** and LiBr being slightly negative (as a result of premature quenching leading to an unproductive pathway). Based on literature precedent, the near-saturation kinetics observed in **PC1** are likely responsible for the apparent pseudo-monophotonic dependence,[17] as opposed to biphotonic dependence which is more commonly seen for conPET processes. We aimed to be conservative with our conclusions based on the light order, apart from proposing that the rate-determining step involves the absorption of photons.

b) Intermittent Illumination (On/Off) Experiment

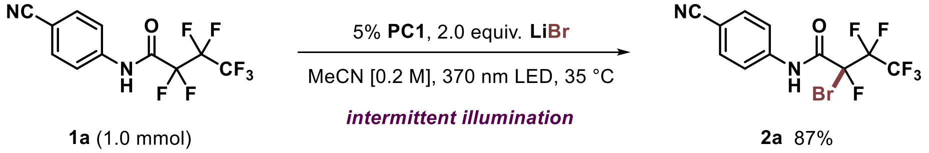

1a (1.0 mmol) **2a** 87%

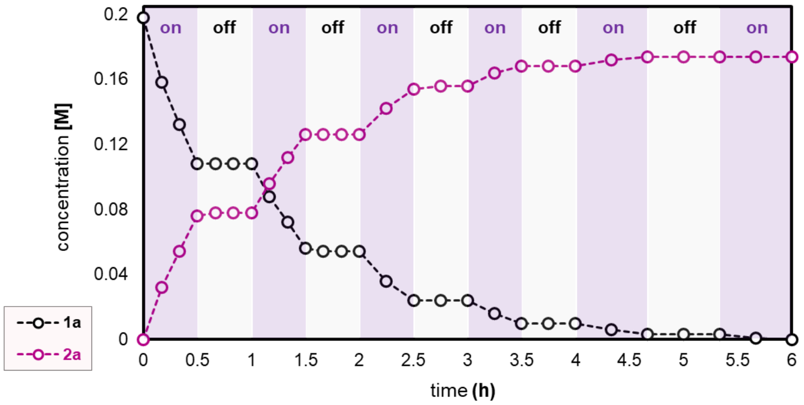

The experiment followed General Procedure A in a 4-dram vial using substrate **1a** (314.2 mg, 1.0 mmol, 1.0 equiv.), photocatalyst **PC1** (16.8 mg, 0.05 mmol, 0.05 equiv.), and LiBr (173.7 mg, 2.0 mmol, 2.0 equiv.) in anhydrous MeCN (5.0 mL). The reaction mixture was stirred under UV irradiation (370 nm) using the two light setup. The LEDs were ***on*** for the following time periods (minutes): 0 → 30, 60 → 90, 120 → 150, 180 → 210, 240 → 280, 320 → 360. The LEDs were ***off*** for the following time periods (minutes): 30 → 60, 90 → 120, 150 → 180, 210 → 240, 280 → 320. The reaction mixture was sampled at the following times (minutes): 0, 10, 20, 30, 40, 50, 60, 70, 80, 90, 100, 110, 120, 135, 150, 165, 180, 195, 210, 225, 240, 260, 280, 300, 320, 340, 360. Terminal ^{19}F NMR yield **2a**: 87%.

Discussion: during the dark reaction intervals, no conversion of substrate **1a** or formation of product **2a** was observed. Moreover, when the dark reaction intervals are removed from the time-course plot and a time shift is applied, the curves for starting material conversion and product formation directly overlap with those of the standard reaction (see below).

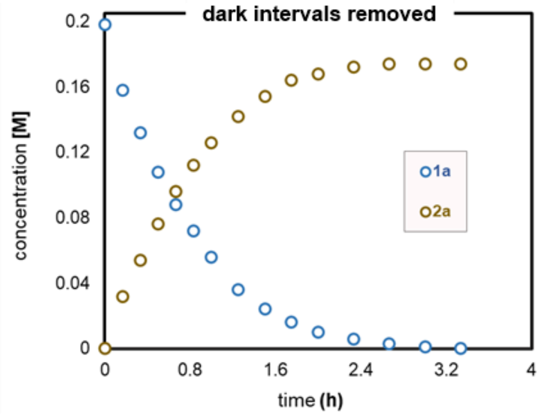

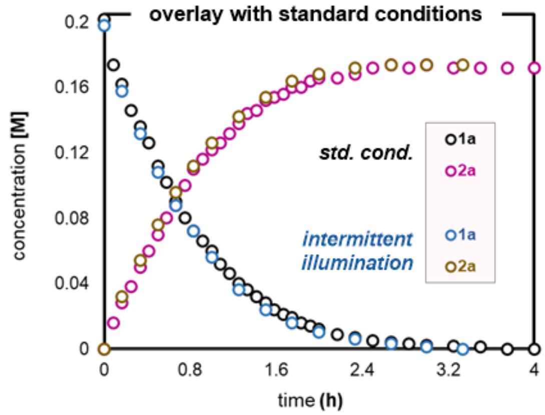

c) LiF Recovery Experiment

1a (1.0 mmol) → [5% **PC1**, 1.2 equiv. **LiBr**; MeCN [0.2 M], 370 nm LED, 35 °C] → 2a 86% + LiF (**72%**)

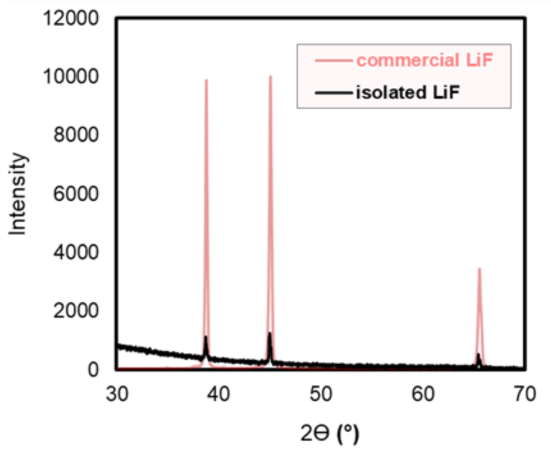

The experiment followed General Procedure A in a 4-dram vial using substrate **1a** (314.2 mg, 1.0 mmol, 1.0 equiv.), photocatalyst **PC1** (16.8 mg, 0.05 mmol, 0.05 equiv.), and LiBr (104.2 mg, 1.2 mmol, 1.2 equiv.) in anhydrous MeCN (5.0 mL). The reaction mixture was stirred under UV irradiation (370 nm) using the two light setup, and the reaction was sampled at 12 h. ^{19}F NMR yield **2a**: 86%. The reaction mixture was then diluted with EtOAc (50 mL) and the organics were extracted with H_2O (6 × 10 mL). The combined aqueous extracts were filtered through a celite pad and subsequently concentrated *in vacuo*. The resulting solid was dried under vacuum (<300 mTorr) at 150 °C for 2 h. LiF was isolated in 72% yield (22.6 mg) as a white solid and characterized by PXRD, matching a reference sample and PDF No. 01-072-1538.

d) Radical Trap Experiments
[i] Replacing LiBr with TEMPO

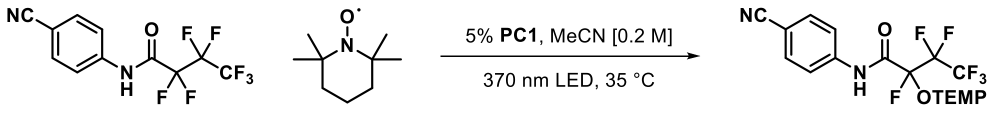

1a 2.0 equiv. **s10** 40% (**40%**)

The experiment followed General Procedure A in a 4-dram vial using substrate **1a** (314.2 mg, 1.0 mmol, 1.0 equiv.), photocatalyst **PC1** (16.8 mg, 0.05 mmol, 0.05 equiv.), and (2,2,6,6-tetramethylpiperidin-1-yl)oxyl (TEMPO, 312.6 mg, 2.0 mmol, 2.0 equiv.) in anhydrous MeCN (5.0 mL). The reaction mixture was allowed to stir for 20 h under UV irradiation (370 nm) using the two light setup. ^{19}F NMR yield: 40%. TEMPO adduct **s10** was isolated in 40% yield (178.9 mg) as a yellow solid after purification by silica gel chromatography (15% EtOAc in hexanes, dry load).

^{1}H NMR ($CDCl_3$, 500 MHz) δ 9.14 (br s, 1H), 7.73 – 7.65 (m, 4H), 1.76 – 1.40 (m, 6H), 1.38 (d, *J* = 3.0 Hz, 3H), 1.27 (m, 6H), 1.20 (s, 3H);
^{19}F NMR ($CDCl_3$, 470 MHz) δ −79.2 (d, *J* = 11.6 Hz, 3F), −100.4 – −100.6 (m, 1F), −121.5 – −121.6 (m, 2F);

HRMS (ESI): calcd. for $C_{20}H_{22}F_6N_3O_2$ $[M-H]^-$: 450.1622. Found 450.1642.

[ii] Bromodefluorination of 1a with Added TEMPO

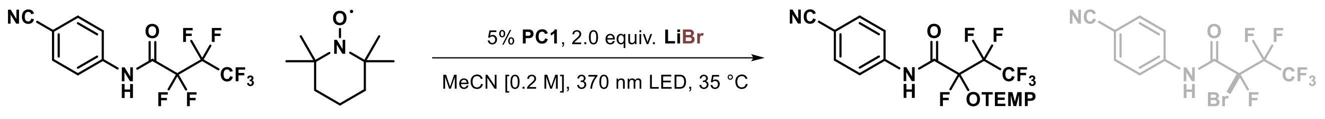

1a 1.0 equiv. **s10** 28% **2a** (not detected)

The experiment followed General Procedure A in a ½-dram vial using substrate **1a** (15.7 mg, 0.05 mmol, 1.0 equiv.), photocatalyst **PC1** (0.84 mg, 0.0025 mmol, 0.05 equiv.), (2,2,6,6-tetramethylpiperidin-1-yl)oxyl (TEMPO, 7.8 mg, 0.05 mmol, 1.0 equiv.), and LiBr (8.7 mg, 0.1 mmol, 2.0 equiv.) in anhydrous MeCN (0.25 mL). The reaction mixture was allowed to stir for 4 h under UV irradiation (370 nm) using the one light setup. ^{19}F NMR yield (**s10**): 28%. Brominated product **2a** was not detected by ^{19}F NMR or HRMS.

[iii] Bromodefluorination of 1a with Added 1,1-Diphenylethylene (DPE)

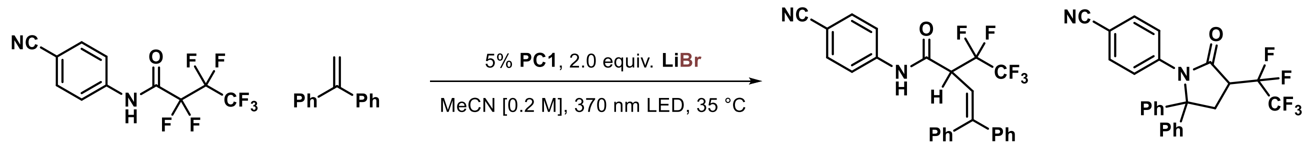

1a 2.0 equiv. **s11** 40% (**38%**) **s12** 18% (**11%**)

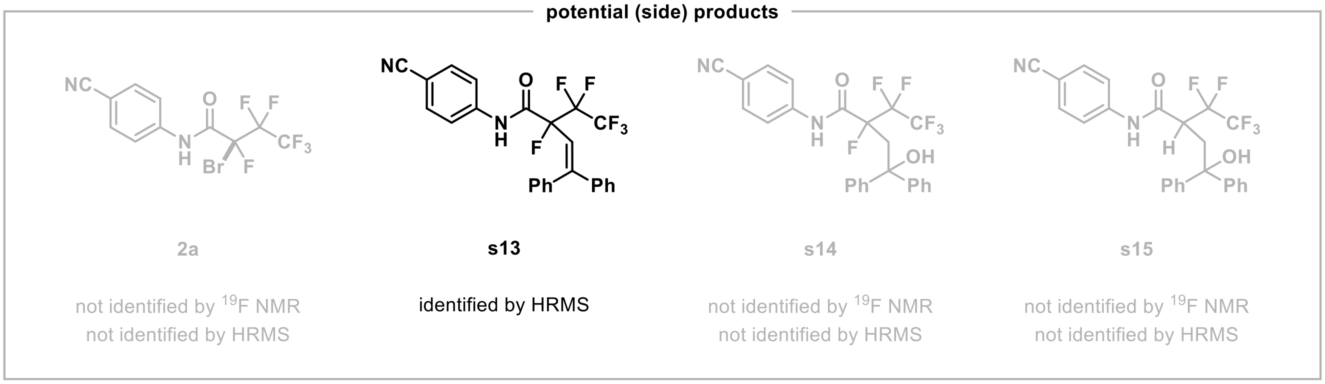

The experiment followed General Procedure A in a 1-dram vial using substrate **1a** (31.4 mg, 0.10 mmol, 1.0 equiv.), photocatalyst **PC1** (1.7 mg, 0.005 mmol, 0.05 equiv.), 1,1-diphenylethylene (36.1 mg, 0.20 mmol, 2.0 equiv.), and LiBr (17.4 mg, 0.20 mmol, 2.0 equiv.) in anhydrous MeCN (0.5 mL). The reaction mixture was allowed to stir for 4 h under UV irradiation (370 nm) using the one light setup. ^{19}F NMR yield (**s11**): 40%. ^{19}F NMR yield (**s12**): 18%. DPE adduct **s11** was isolated in 38% yield (17.4 mg) as a yellow solid, and lactam **s12** was isolated in 11% yield (5.0 mg) as a yellow solid after purification by silica gel chromatography (20% EtOAc in hexanes, dry load). Brominated product **2a** was not detected by ^{19}F NMR or HRMS. DPE adduct **s13** was detected by HRMS.

s11 – **^{1}H NMR** ($CDCl_3$, 400 MHz) δ 7.92 (br s, 1H), 7.67 – 7.57 (m, 4H), 7.48 – 7.39 (m, 3H), 7.32 – 7.27 (m, 3H), 7.26 – 7.21 (m, 2H), 7.20 – 7.14 (m, 2H), 6.34 (d, *J* = 10.3 Hz, 1H), 3.96 – 3.83 (m, 1H);
^{19}F NMR ($CDCl_3$, 376 MHz) δ −82.4 (s, 3F), −114.7 (dd, *J* = 272.1, 8.2 Hz, 1F), −120.2 (dd, *J* = 272.1, 19.6 Hz, 1F);
HRMS (ESI): calcd. for $C_{25}H_{16}F_5N_2O$ $[M-H]^-$: 455.1188. Found 455.1187.

s12 – **^{1}H NMR** ($CDCl_3$, 400 MHz) δ 7.57 – 7.23 (m, 14H), 3.26 – 3.06 (m, 2H), 2.91 (dd, *J* = 11.9, 6.8 Hz, 1H);
^{19}F NMR ($CDCl_3$, 376 MHz) δ −82.4 (s, 3F), −118.0 (dd, *J* = 276.7, 9.7 Hz, 1F), −121.3 (dd, *J* = 276.4, 18.8 Hz, 1F);
HRMS (ESI): calcd. for $C_{25}H_{18}F_5N_2O$ $[M+H]^+$: 457.1334. Found 457.1345.

s13 – **HRMS (ESI)**: calcd. for $C_{25}H_{15}F_6N_2O$ $[M-H]^-$: 473.1094. Found 473.1105.

[iv] DPE Trapping of 1a with 1.0 equiv. H_2O

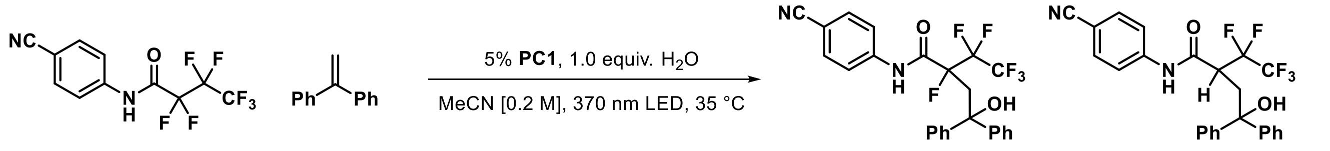

1a 2.0 equiv. **s14** (HRMS) **s15** (HRMS)

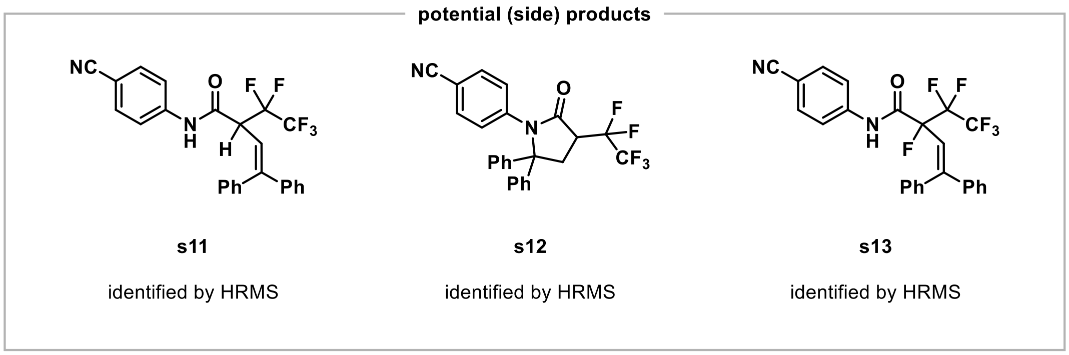

The experiment followed General Procedure A in a 1-dram vial using substrate **1a** (31.4 mg, 0.10 mmol, 1.0 equiv.), photocatalyst **PC1** (1.7 mg, 0.005 mmol, 0.05 equiv.), 1,1-diphenylethylene (36.1 mg, 0.20 mmol, 2.0 equiv.), and H_2O (1.8 mg, 0.10 mmol, 1.0 equiv.) in anhydrous MeCN (0.5 mL). The reaction mixture was allowed to stir for 4 h under UV irradiation (370 nm) using the one light setup. DPE adducts **s11**–**15** were all detected by HRMS.

s14 – **HRMS (ESI)**: calcd. for $C_{25}H_{18}F_5N_2O_2$ $[M-H]^-$: 473.1294. Found 473.1282.

s15 – **HRMS (ESI)**: calcd. for $C_{25}H_{17}F_6N_2O_2$ $[M-H]^-$: 491.1200. Found 491.1197.

e) Bromodefluorination Using PC1 Radical Cation ($PC1^{\cdot+}$)

[i] Synthesis of PC1•PF_6

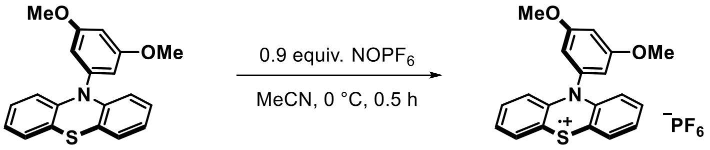

Adapted from a literature procedure.[18] To a 10 mL Schlenk tube charged with a stir bar was added **PC1** (201 mg, 0.60 mmol, 1.0 equiv.). The reaction vessel was subsequently placed under an N_2 atmosphere, and anhydrous MeCN (2.0 mL) was added. The Schlenk tube was then cooled to 0 °C while stirring. A solution of $NOPF_6$ (94.5 mg, 0.54 mmol, 0.9 equiv.) in anhydrous MeCN (2.0 mL) was added dropwise, and the reaction was stirred for a further 0.5 h at 0 °C. After this period, the reaction mixture was flash-frozen using LN_2, and five freeze-pump-thaw cycles were performed to remove the NO gas. The reaction mixture was then allowed to warm to rt, and concentrated *in vacuo*. Isolated in 51% yield (131 mg) as a dark red/black solid after filtering and washing with cold hexanes. **PC1•PF_6** was characterized by absorption spectroscopy as shown below. The absorption profile matches what we recorded spectroelectrochemically for **$PC1^{\cdot+}$** in **Section 9b**.

Note: ***PC1•PF_6*** *was stable for up to two weeks when stored under an inert atmosphere in the dark.*

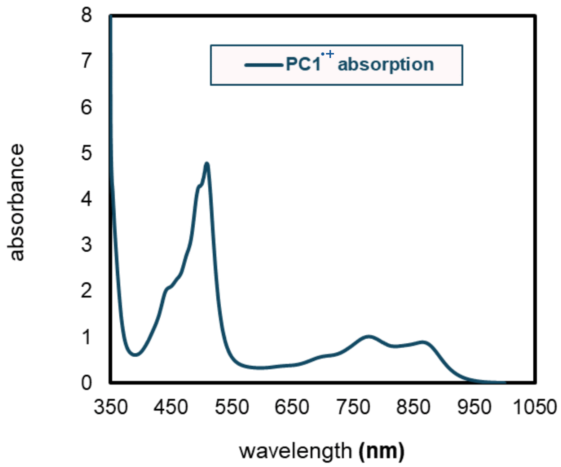

[ii] Bromodefluorination Using PC1•PF_6

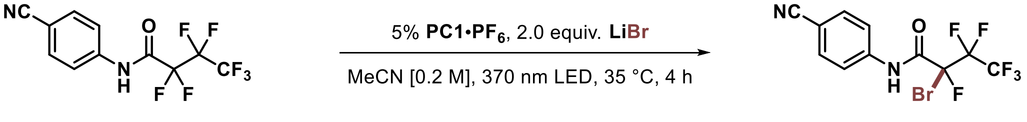

The experiment followed General Procedure A in a 1-dram vial using substrate **1a** (31.4 mg, 0.10 mmol, 1.0 equiv.), photocatalyst **PC1•PF_6** (2.4 mg, 0.005 mmol, 0.05 equiv.), and LiBr (17.4 mg, 0.20 mmol, 2.0 equiv.) in anhydrous MeCN (0.5 mL). The reaction mixture was stirred under UV irradiation (370 nm) using the one light setup. Full conversion of **1a** was observed. ^{19}F NMR yield **2a**: 80%.

9 Absorption Spectroscopy (UV-Vis, Spectroelectrochemistry)

a) Steady-State Absorption

[i] PC1: Concentration Dependence

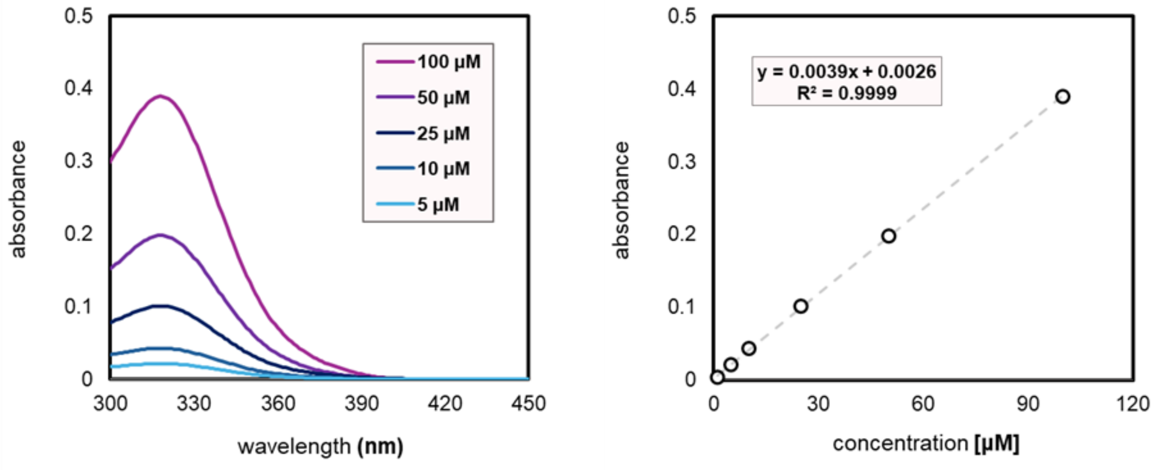

The experiment was performed using different concentrations of **PC1** in MeCN (100, 50, 25, 10, 5 µM) at room temperature. A calibration curve was constructed using the absorbance values obtained from the peak maxima (λ_{max} = 318 nm) at the different concentrations used. The molar extinction coefficient (ε) was calculated to be 3880 M^{-1} cm^{-1} using the slope of the linear trendline.

[ii] PC2: Concentration Dependence

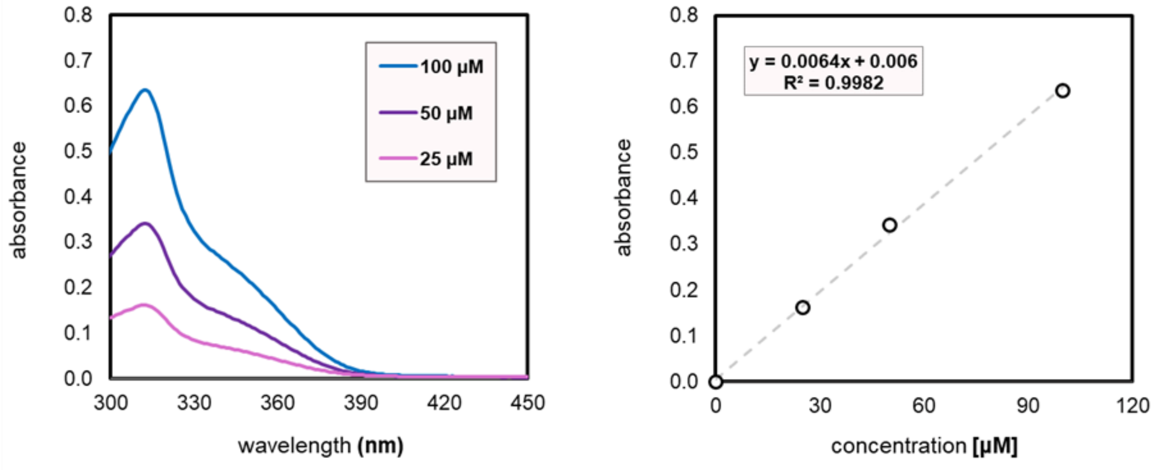

The experiment was performed using different concentrations of **PC2** in MeCN (100, 50, 25 µM) at room temperature. A calibration curve was constructed using the absorbance values obtained from the peak maxima (λ_{max} = 312 nm) at the different concentrations used. The molar extinction coefficient (ε) was calculated to be 6367 M^{-1} cm^{-1} using the slope of the linear trendline.

b) Spectroelectrochemistry

[i] PC1 Radical Cation ($PC1^{\cdot+}$): Concentration Dependence

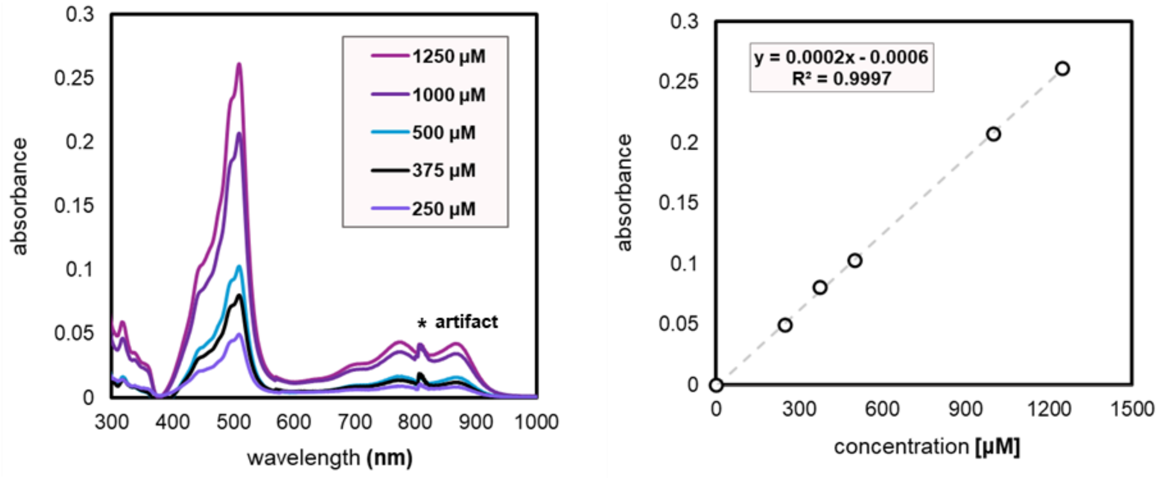

The experiment was performed using different concentrations of **PC1** in MeCN (1250, 1000, 500, 375, 250 μM) at room temperature, with a constant applied potential of +0.65 V vs. Ag/AgCl in order to generate the corresponding radical cation **$PC1^{\cdot+}$**. A calibration curve was constructed using the absorbance values obtained from the peak maxima (λ_{max} = 509 nm) at the different concentrations used. The molar extinction coefficient (ε) was calculated to be 10450 M^{-1} cm^{-1} using the slope of the linear trendline. See **Section 11a** for a cyclic voltammogram of **PC1** (**PC1** E^{ox} +0.78 V vs. Ag/AgCl in MeCN; +0.73 V vs. SCE).

Note: *the sharp peak at 800 nm is an artifact arising from detector changeover.*

[ii] PC1 Dication ($PC1^{2+}$) Generation Using a Larger Applied Potential

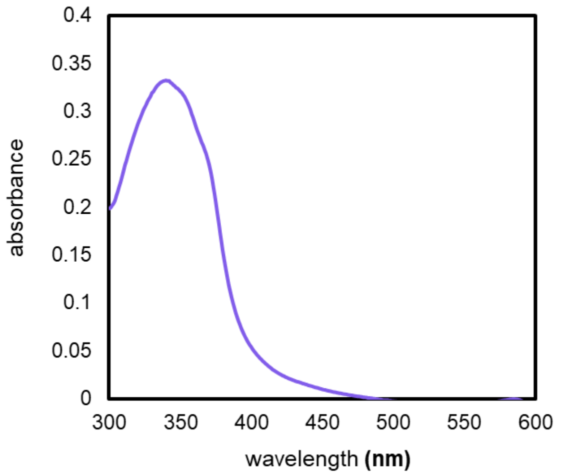

The experiment was performed using **PC1** (1000 μM in MeCN) at room temperature with a constant applied potential of +1.50 V vs. Ag/AgCl in order to generate the corresponding dication **$PC1^{2+}$**. See **Section 11a** for a cyclic voltammogram of **PC1** (**$PC1^{\cdot+}$** E^{ox} +1.47 V vs. Ag/AgCl in MeCN; +1.42 V vs. SCE).

[iii] PC2 Radical Cation (PC2$^{\bullet+}$)

Note: *concentration-dependent studies were not performed due to the poor solubility of **PC2** in MeCN at concentrations greater than 500 μM.*

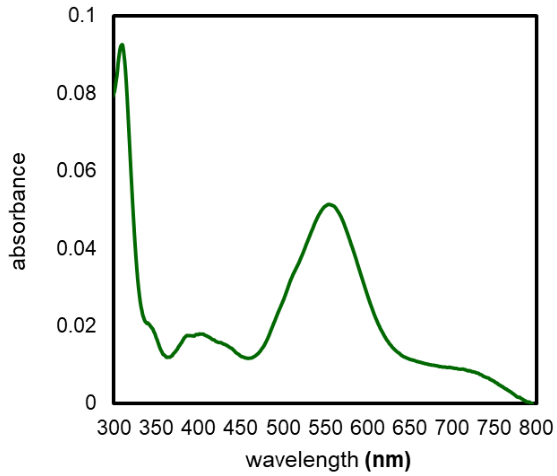

The experiment was performed using **PC2** (~500 μM in MeCN) at room temperature with a constant applied potential of +0.80 V vs. Ag/AgCl in order to generate the corresponding radical cation **PC2$^{\bullet+}$**. See **Section 11a** for a cyclic voltammogram of **PC2** (**PC2$^{\bullet+}$** E^{ox} +0.87 V vs. Ag/AgCl in MeCN; +0.82 V vs. SCE).

[iv] PC2 Dication (PC2^{2+}) Generation Using a Larger Applied Potential

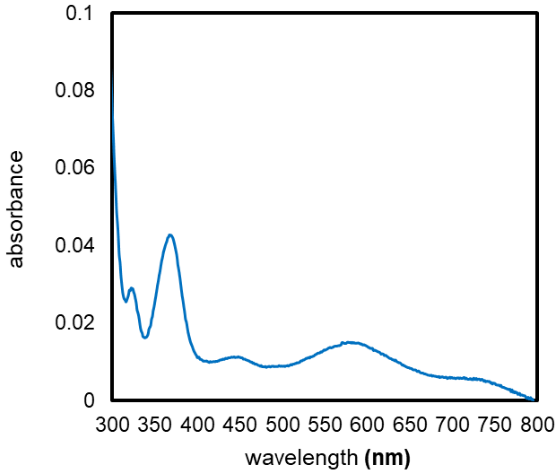

The experiment was performed using **PC2** (~500 μM in MeCN) at room temperature with a constant applied potential of +1.50 V vs. Ag/AgCl in order to generate the corresponding dication **PC2^{2+}**. See **Section 11a** for a cyclic voltammogram of **PC2** (**PC2$^{\bullet+}$** E^{ox} +1.48 V vs. Ag/AgCl in MeCN; +1.44 V vs. SCE).

Note: *some residual radical cation (**PC2$^{\bullet+}$**) is still present in the spectrum above. Full conversion of the radical cation to the dication (**PC2^{2+}**) was not observed, as the dication undergoes another oxidation event at +1.67 V vs. Ag/AgCl in MeCN (+1.63 V vs. SCE) and rapidly decomposes.*

c) Photocatalyst Absorption Comparison

Note: *this is a direct comparison of absorption spectra obtained above in* ***Sections 9a*** *&* ***9b****. No new data were collected for the following plots.*

[i] PC1: Neutral vs. Radical Cation

Concentration-dependent absorption was conducted for both **PC1** & **PC1**$^{•+}$, thus the following data were compared by plotting the molar extinction coefficient as a function of wavelength. The absorption maxima for **PC1** (λ_{max} = 318 nm) was then normalized against the emission intensity of the 370 nm LED lamp (**Section 10a**) in order to directly compare the absorbance of **PC1** & **PC1**$^{•+}$ in the region of the LED emission (350–400 nm). The righthand plot is a normalized inset of the lefthand plot.

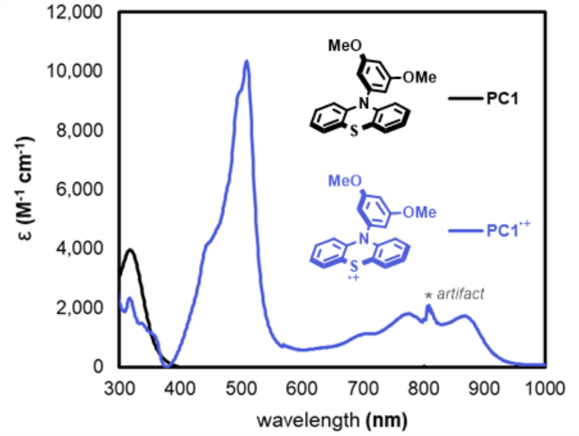

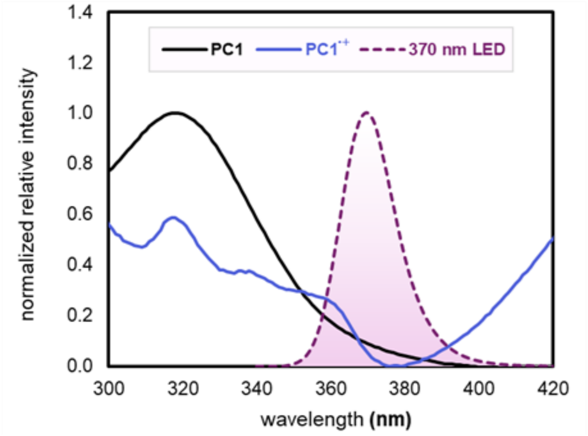

[ii] PC2: Neutral vs. Radical Cation

Concentration-dependent absorption was only conducted for **PC2**, thus the following data were compared by plotting absorbance as a function of wavelength. The absorption maxima for **PC2** (λ_{max} = 312 nm) and **PC2**$^{•+}$ (λ_{max} = 554 nm) were then normalized against the emission intensity of the 525 nm LED lamp (**Section 10a**).

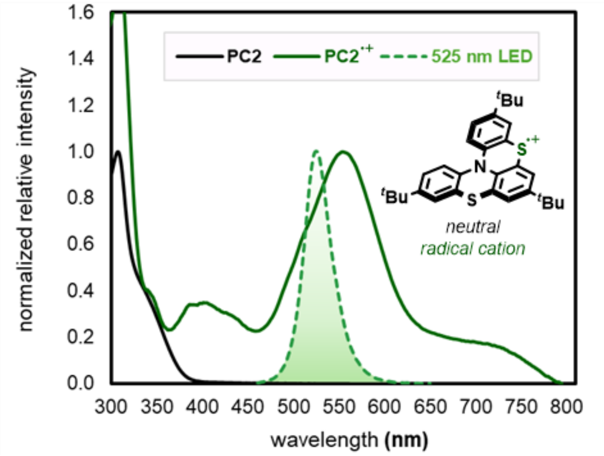

10 Emission Spectroscopy (Steady-State, Time-Resolved)

a) LED Lamp Emission

Raw emission data for the PR160L-525 and PR160L-370 Gen 2 LEDs were graciously provided by Kessil and are plotted below. https://kessil.com/products/science_main.php

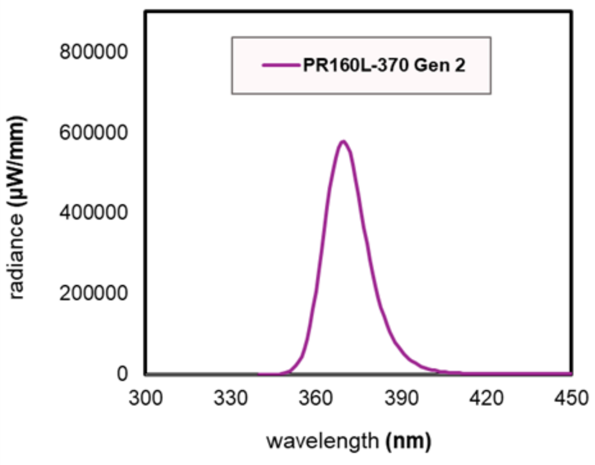

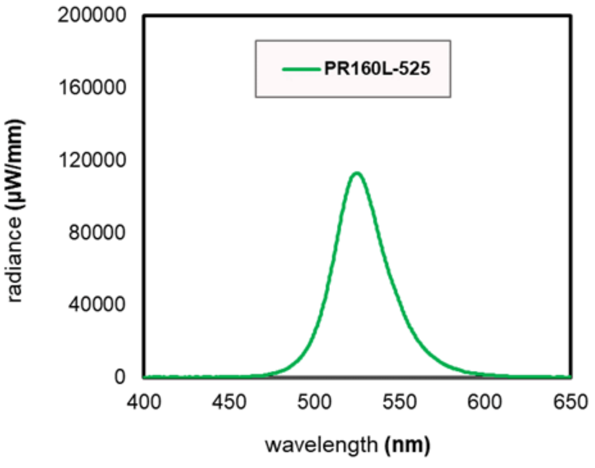

b) Steady-State Fluorescence
[i] PC1 Emission

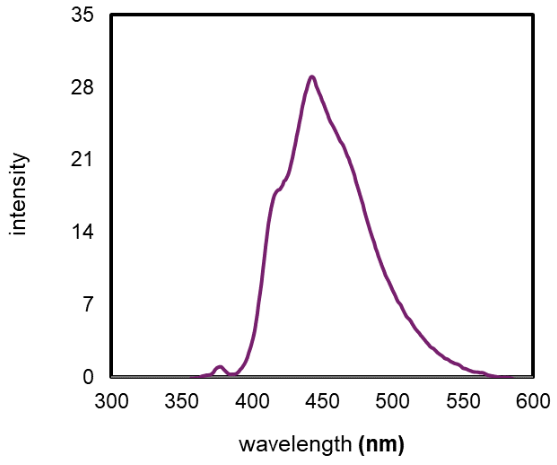

50 μM **PC1** (MeCN), λ_{exc} = 340 nm, $\Delta\lambda_{exc}$ = 5 nm, $\Delta\lambda_{em}$ = 5 nm. The maximum emission intensity (λ_{max}) was observed at 443 nm.

[ii] PC2 Emission

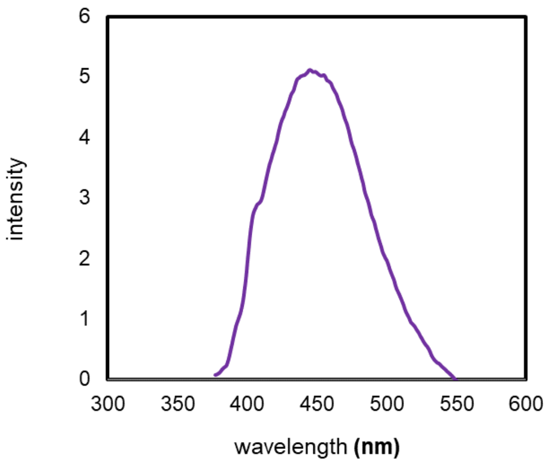

50 μM **PC2** (MeCN), λ_{exc} = 360 nm, $\Delta\lambda_{exc}$ = 5 nm, $\Delta\lambda_{em}$ = 5 nm. The maximum emission intensity (λ_{max}) was observed at 445 nm.

[iii] PC1 Excitation

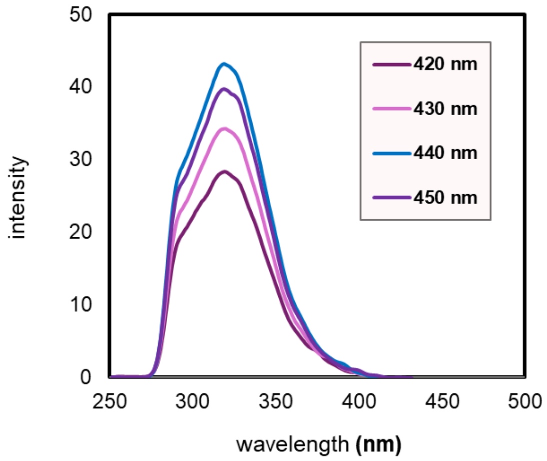

Excitation spectra of **PC1** (50 μM in MeCN) were recorded at λ_{em} = 420, 430, 440, and 450 nm ($\Delta\lambda_{exc}$ = 5 nm, $\Delta\lambda_{em}$ = 5 nm). Identical excitation spectra were observed for **PC1** at different emission wavelengths. Notably, all four emission wavelengths resulted in similar excitation profiles (280–400 nm) with λ_{max} = 318 nm, which resembles the absorption spectrum of **PC1**. The similar absorption and excitation profiles suggest that emission occurs from a locally-excited state.

[iv] PC2 Excitation

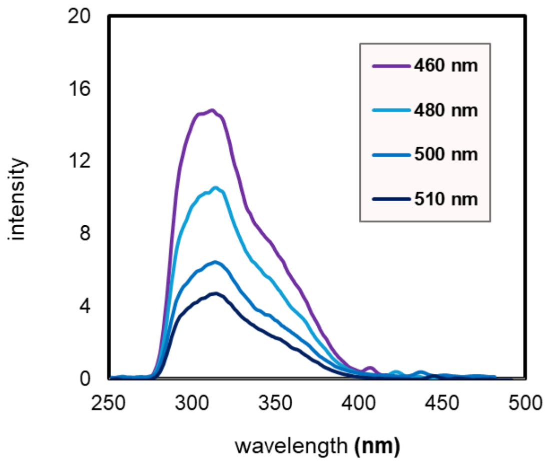

Excitation spectra of **PC2** (50 µM in MeCN) were recorded at λ_{em} = 460, 480, 500, and 510 nm ($\Delta\lambda_{exc}$ = 5 nm, $\Delta\lambda_{em}$ = 5 nm). Identical excitation spectra were observed for **PC2** at different emission wavelengths. Notably, all four emission wavelengths resulted in similar excitation profiles (280–400 nm) with λ_{max} = 312 nm, which resembles the absorption spectrum of **PC2**. The similar absorption and excitation profiles suggest that emission occurs from a locally-excited state.

[v] PC1: Concentration Dependence

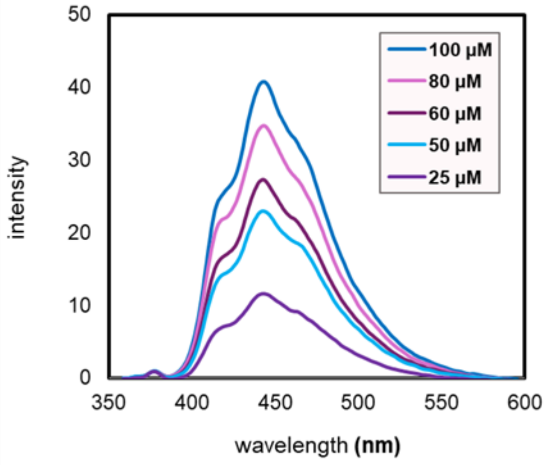

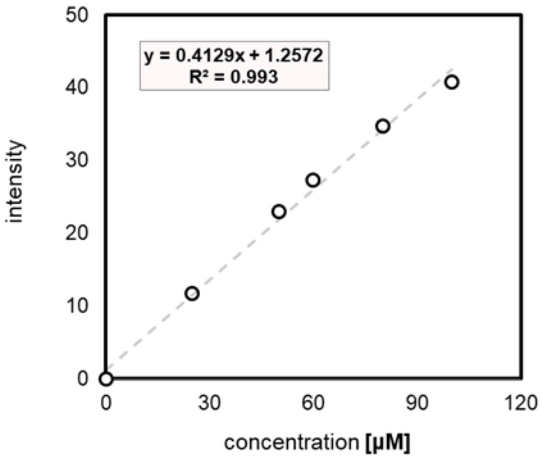

The experiment was performed using different concentrations of **PC1** in MeCN (100, 80, 60, 50, 25 µM) at room temperature (λ_{exc} = 340 nm, $\Delta\lambda_{exc}$ = 5 nm, $\Delta\lambda_{em}$ = 5 nm). A calibration curve was constructed using the intensity values obtained from the peak maxima (λ_{max} = 443 nm) at the different concentrations used, which revealed a linear trend.

[vi] PC1 Radical Cation ($PC1^{\bullet+}$): Near-IR Fluorescence

Note: the following emission spectrum was recorded on an Edinburgh Instruments FS5 Spectrofluorometer due to the requirement of near-IR detection.

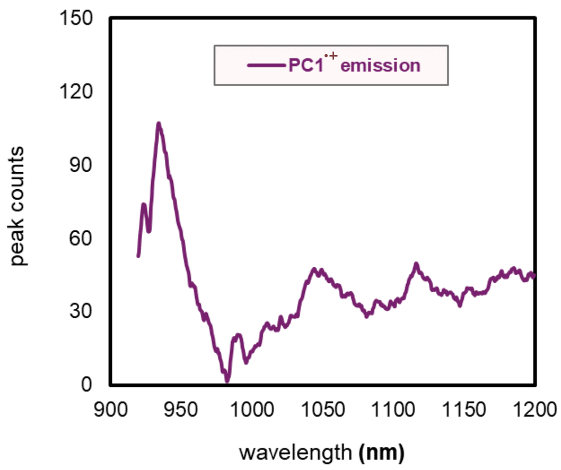

The synthesis of **PC1•PF₆** is described in **Section 8e**. The emission spectrum of **PC1•PF₆** (~100 μM in MeCN) was recorded via TCSPC using an excitation wavelength (λ_{exc}) of 880 nm. The emission spectrum obtained above (λ_{max} = 935 nm) closely resembles that of the *N*-phenylphenothiazine radical cation ($PTH^{\bullet+}$, λ_{max} = 924 nm).[19]

[vii] Steady-State Stern-Volmer Fluorescence Quenching of PC1

Steady-state quenching studies were performed for **PC1** using **1a**, **2a**, and LiBr as quenchers.

Substrate 1a

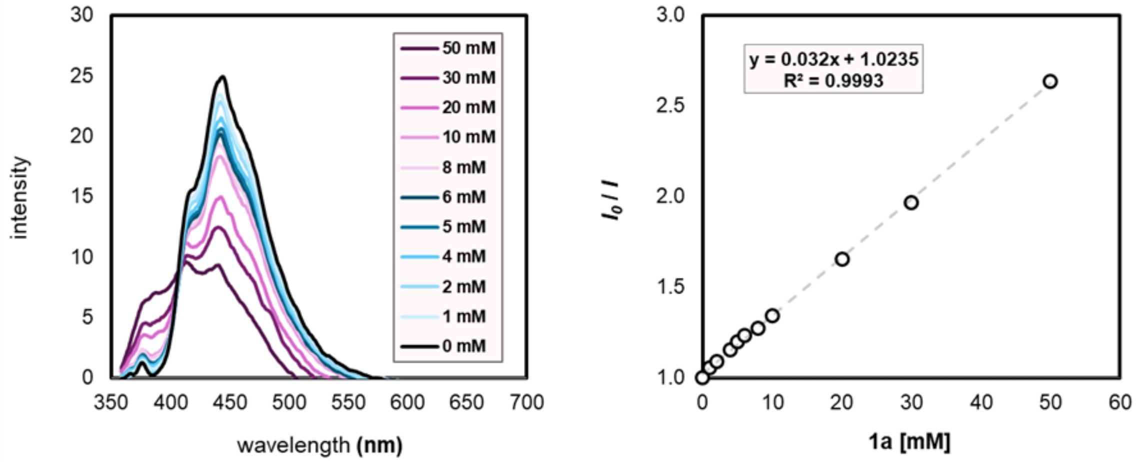

The experiment was performed using **PC1** (λ_{exc} = 340 nm, $\Delta\lambda_{exc}$ = 5 nm, $\Delta\lambda_{em}$ = 5 nm) with varying concentrations of **1a**. A stock solution of **PC1** (50 μM in MeCN) was prepared, which was used to make a separate stock solution of **1a** (50 mM in MeCN). Serial dilutions were then performed with the **PC1**

stock solution to prepare different solutions of **1a** at varying concentrations (30, 20, 10, 8, 6, 5, 4, 2, 1 mM), such that the concentration of **PC1** remained constant (50 μM). From the emission spectra, the maximum fluorescence intensity (I) at the global emission maximum was noted (λ_{max} = 443 nm for **PC1**). The fluorescence intensity of **PC1** in the absence of **1a** was denoted as I_0, while those in the presence of different concentrations of **1a** were labeled as I. A plot of I_0/I against the concentration of **1a** revealed a linear relationship, with a Stern-Volmer quenching constant (K_{SV}) of 32.0 M^{-1} (slope of the linear trendline).

Product 2a

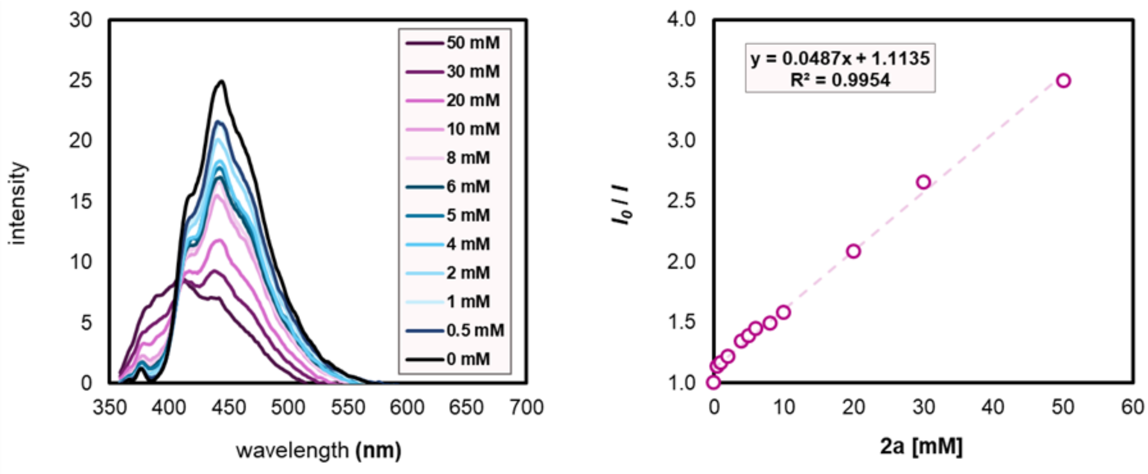

The experiment was performed using **PC1** (λ_{exc} = 340 nm, $\Delta\lambda_{exc}$ = 5 nm, $\Delta\lambda_{em}$ = 5 nm) with varying concentrations of **2a**. A stock solution of **PC1** (50 μM in MeCN) was prepared, which was used to make a separate stock solution of **2a** (50 mM in MeCN). Serial dilutions were then performed with the **PC1** stock solution to prepare different solutions of **2a** at varying concentrations (30, 20, 10, 8, 6, 5, 4, 2, 1, 0.5 mM), such that the concentration of **PC1** remained constant (50 μM). From the emission spectra, the maximum fluorescence intensity (I) at the global emission maximum was noted (λ_{max} = 443 nm for **PC1**). The fluorescence intensity of **PC1** in the absence of **2a** was denoted as I_0, while those in the presence of different concentrations of **2a** were labeled as I. A plot of I_0/I against the concentration of **2a** revealed a linear relationship, with a Stern-Volmer quenching constant (K_{SV}) of 48.7 M^{-1} (slope of the linear trendline).

LiBr

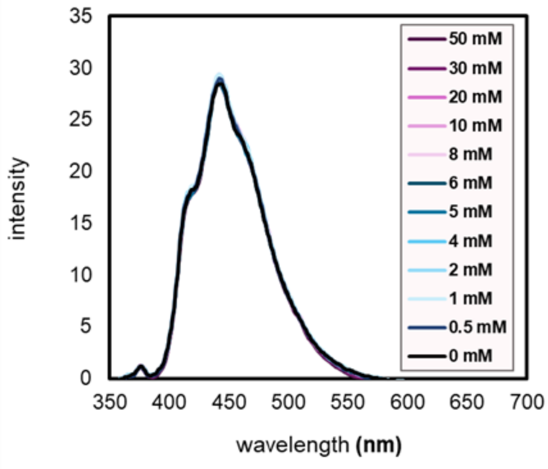

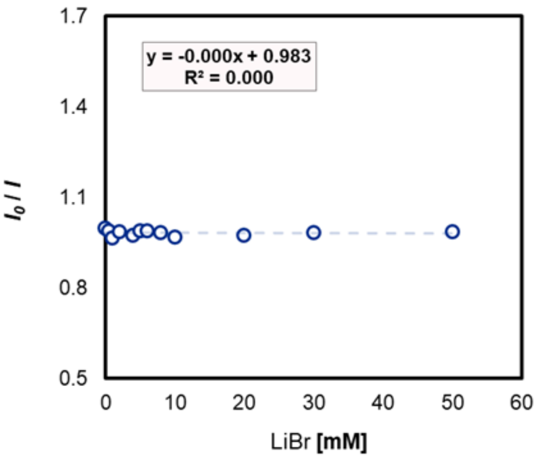

The experiment was performed using **PC1** (λ_{exc} = 340 nm, $\Delta\lambda_{exc}$ = 5 nm, $\Delta\lambda_{em}$ = 5 nm) with varying concentrations of LiBr. A stock solution of **PC1** (50 μM in MeCN) was prepared, which was used to make a separate stock solution of LiBr (50 mM in MeCN). Serial dilutions were then performed with the **PC1** stock solution to prepare different solutions of LiBr at varying concentrations (30, 20, 10, 8, 6, 5, 4, 2, 1, 0.5 mM), such that the concentration of **PC1** remained constant (50 μM). From the emission spectra, the maximum fluorescence intensity (I) at the global emission maximum was noted (λ_{max} = 443 nm for **PC1**). The fluorescence intensity of **PC1** in the absence of LiBr was denoted as I_0, while those in the presence of different concentrations of **2a** were labeled as I. A plot of I_0/I against the concentration of **2a** revealed no evidence of fluorescence quenching.

c) Time-Resolved Fluorescence

[i] Instrument Response Function (IRF)

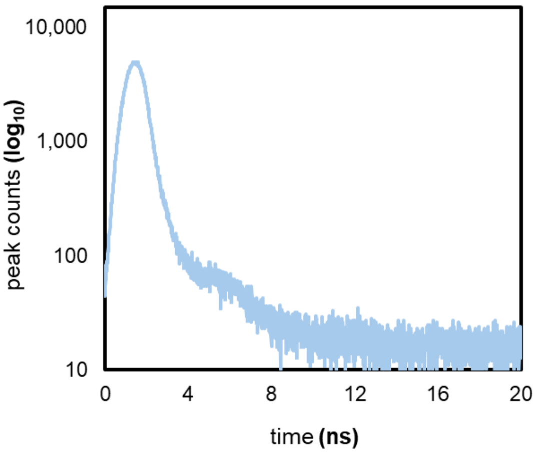

The IRF was recorded using a colloidal suspension of alumina (0.5 μM) in H_2O. λ_{exc} = 365 nm, λ_{em} = 440 nm, 850 ps pulse width, 5000 peak counts (TCSPC), 20 ns time range.

[ii] PC1 Fluorescence Lifetime Decay

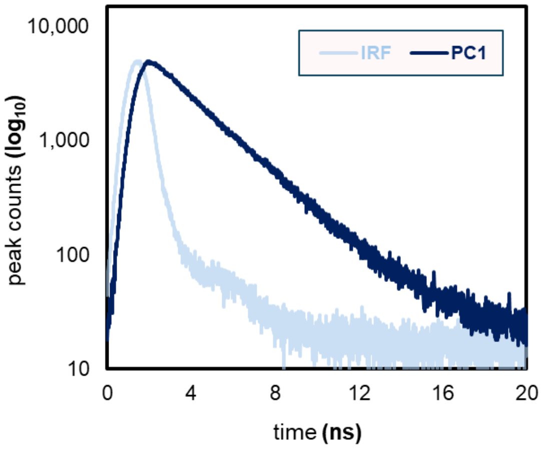

50 µM **PC1** (MeCN), λ_{exc} = 365 nm, λ_{em} = 440 nm, 850 ps pulse width, 5000 peak counts (TCSPC), 20 ns time range. τ = 2.54 ns, calculated using the tail fit method (χ^2 = 1.07, monoexponential decay).

[iii] Time-Resolved Stern-Volmer Fluorescence Quenching of PC1

Time-resolved quenching studies were performed for **PC1** using **1a** and **2a** as quenchers.

Substrate 1a

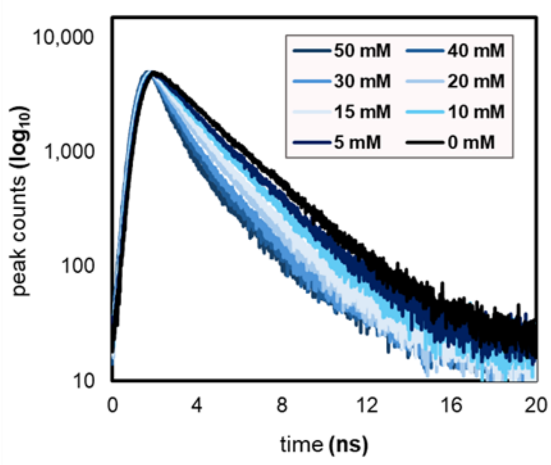

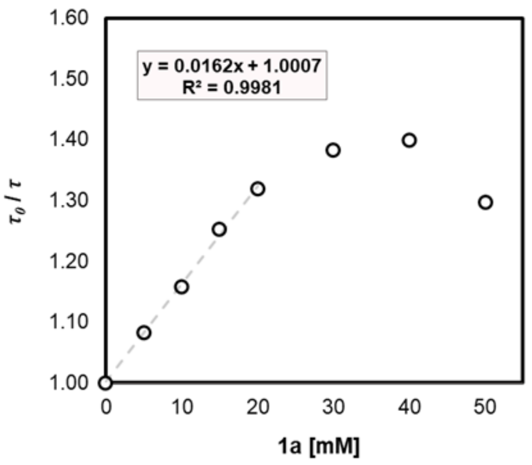

The experiment was performed using **PC1** (λ_{exc} = 365 nm, λ_{em} = 440 nm, 850 ps pulse width, 5000 peak counts (TCSPC), 20 ns time range) with varying concentrations of **1a**. A stock solution of **PC1** (50 µM in MeCN) was prepared, which was used to make a separate stock solution of **1a** (50 mM in MeCN). Serial dilutions were then performed with the **PC1** stock solution to prepare different solutions of **1a** at varying concentrations (40, 30, 20, 15, 10, 5 mM), such that the concentration of **PC1** remained constant (50 µM). The fluorescence lifetime of **PC1** in the absence of **1a** was denoted as τ_0 (2.54 ns, χ^2 = 1.07), while those in the presence of different concentrations of **1a** were labeled as τ. A plot of τ_0/τ against the concentration of **1a** (up to 20 mM) revealed a linear relationship, indicating a dynamic

quenching component is present. Non-dynamic contributions become more pronounced at quencher concentrations >20 mM, thus we have estimated K_{SV} and k_q (bimolecular quenching constant) using the linear regime of the curve (≤20 mM **1a**). These non-dynamic contributions are likely due to inner-filter effects of the substrate and static quenching, which is consistent with the steady-state data. From the slope of the linear trendline, the K_{SV} was determined to be 16.2 M^{-1}. This value differs from that obtained in steady-state measurements (32.0 M^{-1}), which indicates that an additional static quenching component is likely present even at concentrations of ≤20 mM **1a**. As such, we determined k_q and K_{SV} of the time-resolved dynamic component to be 16.2 M^{-1} and 6.37 × 10^9 M^{-1} s^{-1} respectively, using the relationship $K_{SV} = k_q(\tau)$.

Product 2a

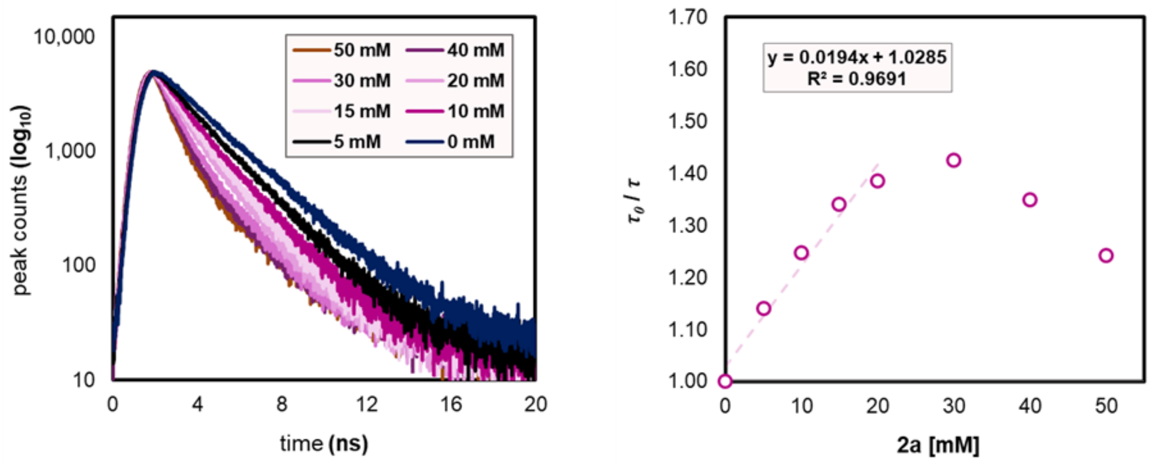

The experiment was performed using **PC1** (λ_{exc} = 365 nm, λ_{em} = 440 nm, 850 ps pulse width, 5000 peak counts (TCSPC), 20 ns time range) with varying concentrations of **2a**. A stock solution of **PC1** (50 µM in MeCN) was prepared, which was used to make a separate stock solution of **2a** (50 mM in MeCN). Serial dilutions were then performed with the **PC1** stock solution to prepare different solutions of **2a** at varying concentrations (40, 30, 20, 15, 10, 5 mM), such that the concentration of **PC1** remained constant (50 µM). The fluorescence lifetime of **PC1** in the absence of **2a** was denoted as τ_0 (2.54 ns, χ^2 = 1.07), while those in the presence of different concentrations of **2a** were labeled as τ. A plot of τ_0/τ against the concentration of **2a** (up to 20 mM) revealed a linear relationship, indicating a dynamic quenching component is present. Non-dynamic contributions become more pronounced at quencher concentrations >20 mM, thus we have estimated K_{SV} and k_q (bimolecular quenching constant) using the linear regime of the curve (≤20 mM **2a**). These non-dynamic contributions are likely due to inner-filter effects of the substrate and static quenching, which is consistent with the steady-state data. From the slope of the linear trendline, the K_{SV} was determined to be 19.4 M^{-1}. This value differs from that obtained in steady-state measurements (48.7 M^{-1}), which indicates that an additional static quenching component is likely present even at concentrations of ≤20 mM **2a**. As such, we determined k_q and K_{SV} of the time-resolved dynamic component to be 19.4 M^{-1} and 7.64 × 10^9 M^{-1} s^{-1} respectively, using the relationship $K_{SV} = k_q(\tau)$.

11 Electrochemistry

General Information for Electrochemistry: the reference electrode was an Ag/AgCl (sat. KCl) aqueous electrode, with Pt wire used as the counter electrode. Redox potentials were determined using the peak maxima and converted to SCE by subtracting 0.044 V from the values obtained vs. Ag/AgCl.[2]

a) Cyclic Voltammetry

[i] PC1

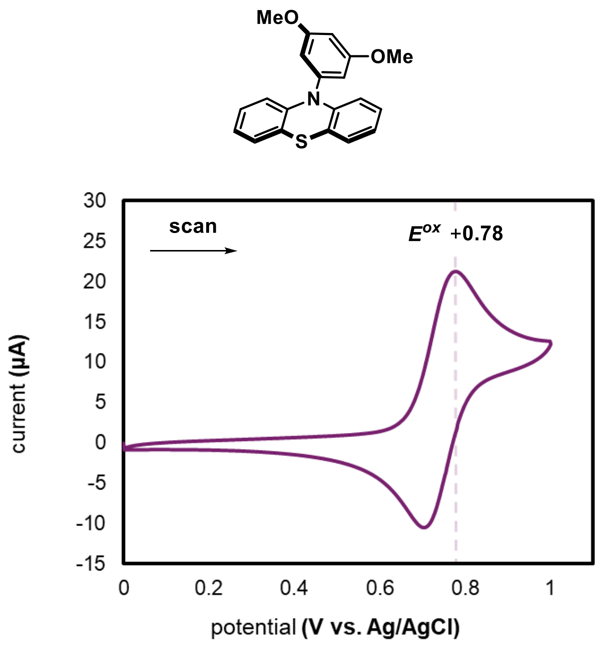

0.5 M **PC1** (1.0 M $TBAPF_6$ in MeCN), ν = 100 mV/s. E^{ox} = +0.777 V vs. Ag/AgCl; +0.733 V vs. SCE.

Wide

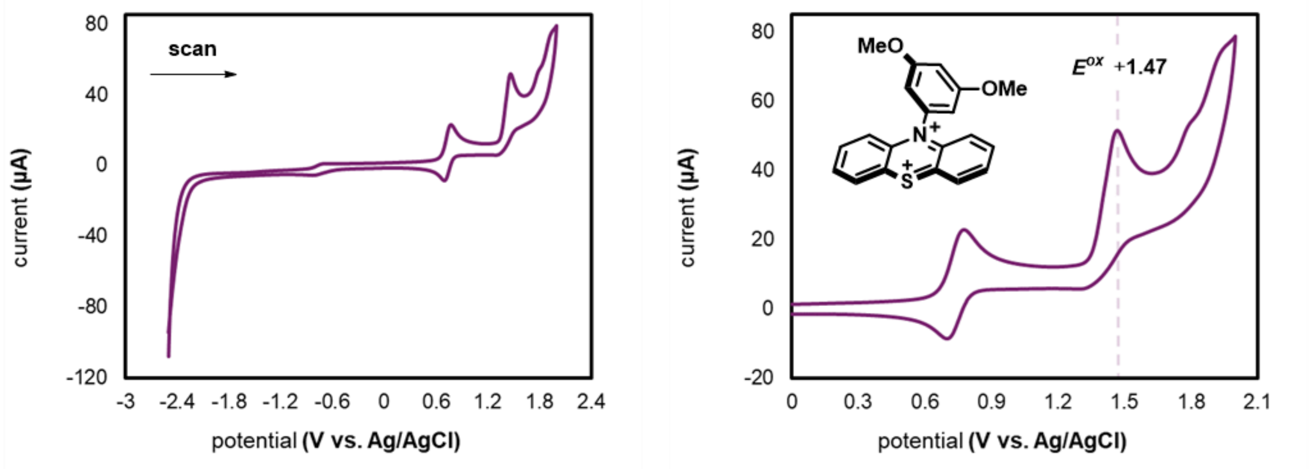

A wider scan for **PC1** was also performed. 0.5 M **PC1** (1.0 M $TBAPF_6$ in MeCN), ν = 100 mV/s. E^{ox} = +0.777 V vs. Ag/AgCl; +0.733 V vs. SCE. A second oxidation event was observed at E^{ox} = +1.465 V vs. Ag/AgCl; +1.421 V vs. SCE, which is attributed to the dication (**PC1^{2+}**).

[ii] PC2

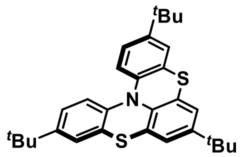

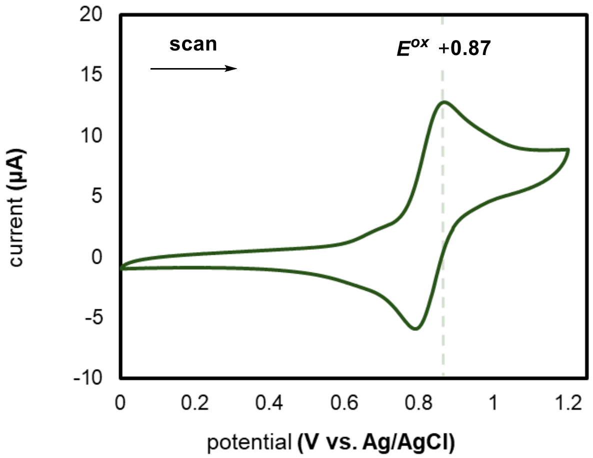

0.5 M **PC2** (1.0 M $TBAPF_6$ in MeCN), ν = 100 mV/s. E^{ox} = +0.865 V vs. Ag/AgCl; +0.821 V vs. SCE.

Wide

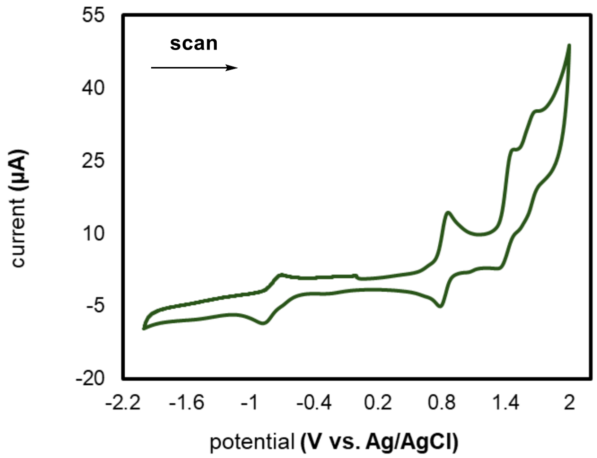

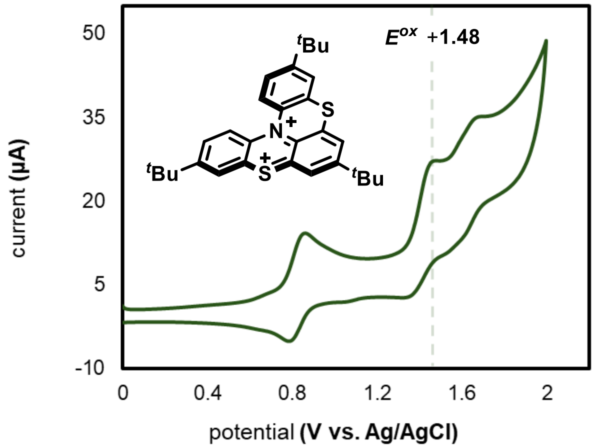

A wider scan for **PC2** was also performed. 0.5 M **PC2** (1.0 M $TBAPF_6$ in MeCN), ν = 100 mV/s. E^{ox} = +0.863 V vs. Ag/AgCl; +0.819 V vs. SCE. A second oxidation event was observed at E^{ox} = +1.479 V vs. Ag/AgCl; +1.435 V vs. SCE, which is attributed to the dication (**PC2^{2+}**). A third oxidation event was observed at

E^{ox} = +1.698 V vs. Ag/AgCl; +1.654 V vs. SCE, which we speculate could be the tricationic species (**PC2^{3+}**), although further characterization is needed to confirm this hypothesis.

[iii] 1a

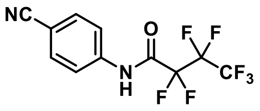

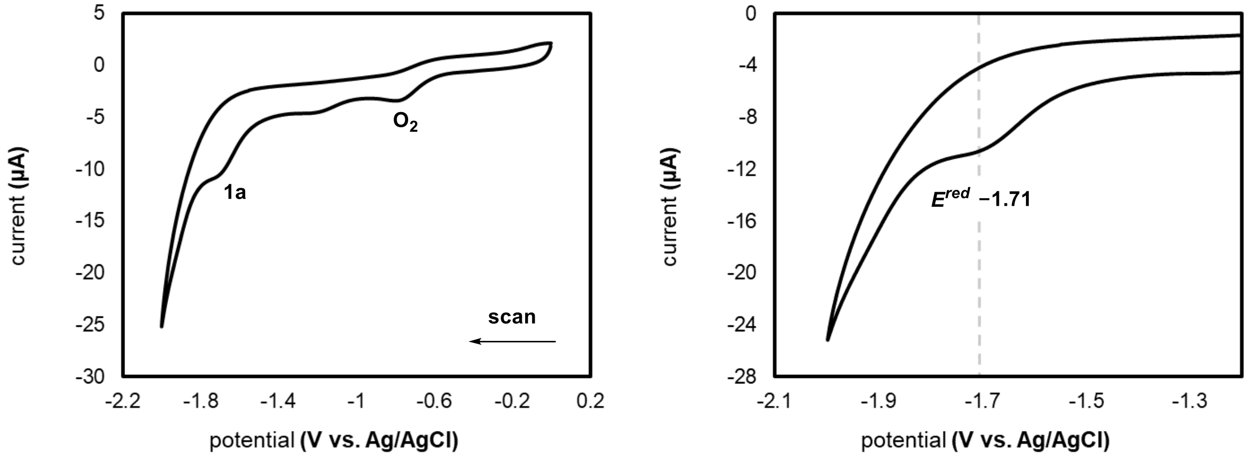

0.5 M **1a** (1.0 M TBAPF$_6$ in MeCN), ν = 100 mV/s. E^{red} = −1.709 V vs. Ag/AgCl; −1.753 V vs. SCE.

[iv] 1c

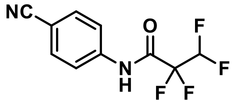

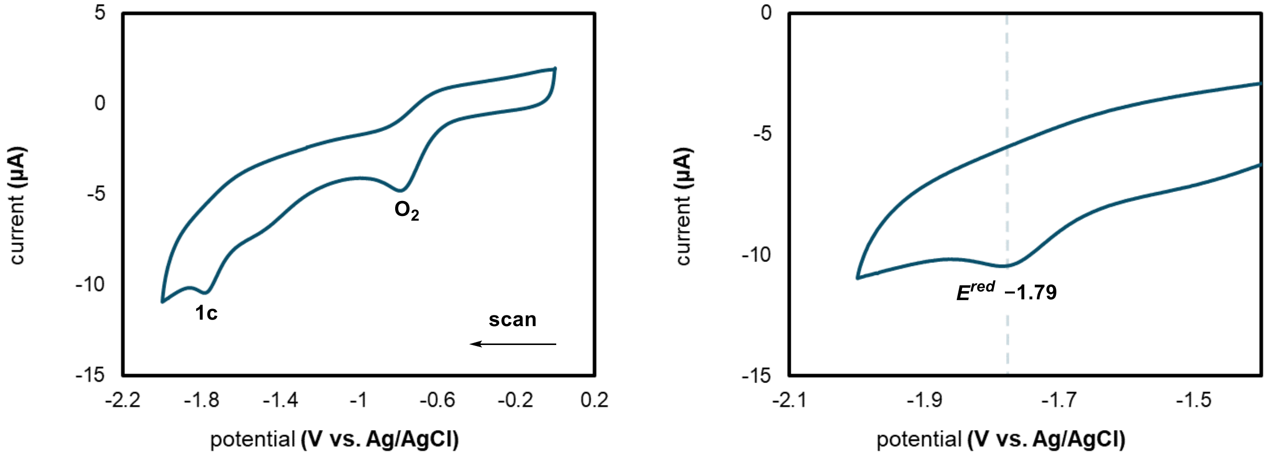

0.5 M **1c** (1.0 M TBAPF$_6$ in MeCN), ν = 100 mV/s. E^{red} = −1.788 V vs. Ag/AgCl; −1.832 V vs. SCE.

[v] 1j

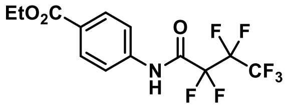

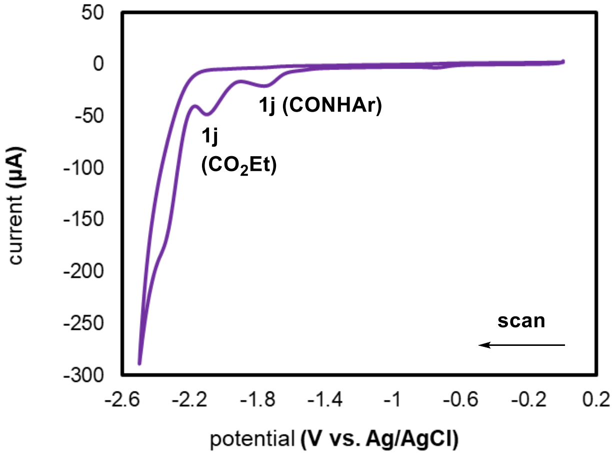

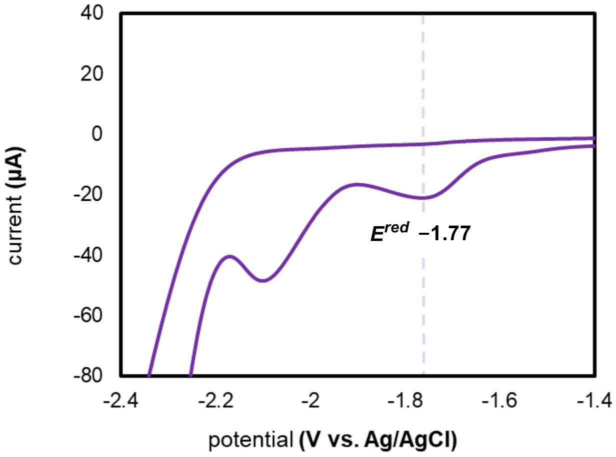

0.5 M **1j** (1.0 M $TBAPF_6$ in MeCN), ν = 100 mV/s. E^{red} = −1.765 V vs. Ag/AgCl; −1.809 V vs. SCE. A second reduction event was observed at E^{red} = −2.101 V vs. Ag/AgCl; −2.145 V vs. SCE, which is attributed to reduction of the aryl ester group.

[vi] 1n

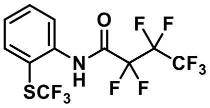

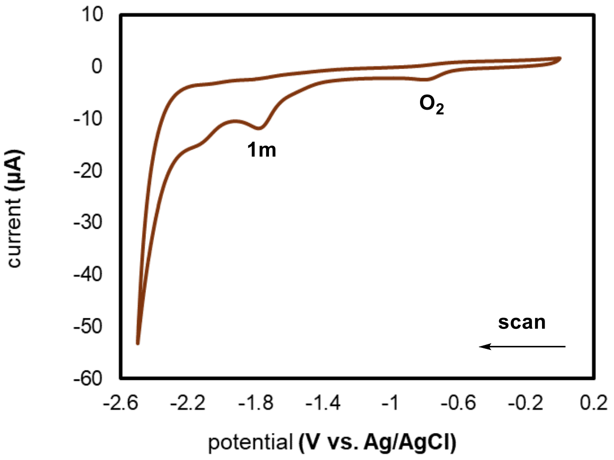

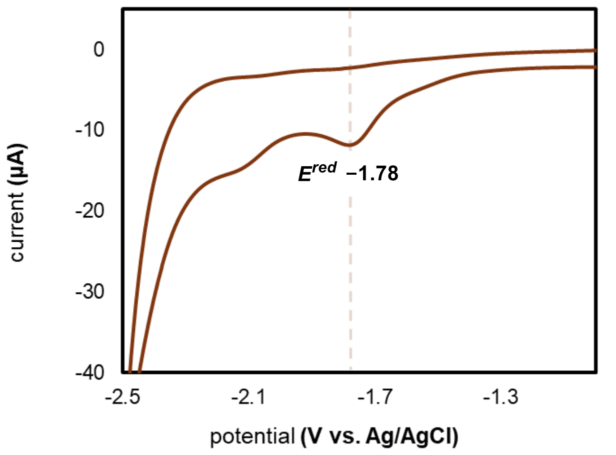

0.5 M **1n** (1.0 M $TBAPF_6$ in MeCN), ν = 100 mV/s. E^{red} = −1.782 V vs. Ag/AgCl; −1.826 V vs. SCE.

[vii] 1v

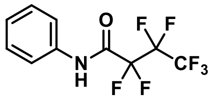

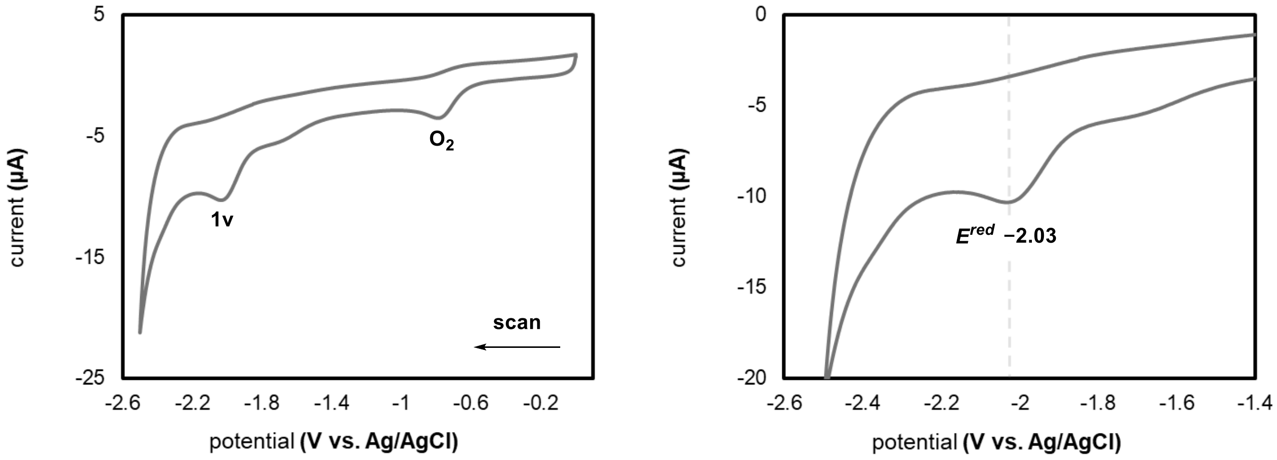

0.5 M **1v** (1.0 M $TBAPF_6$ in MeCN), ν = 100 mV/s. E^{red} = −2.032 V vs. Ag/AgCl; −2.076 V vs. SCE.

[viii] 2a

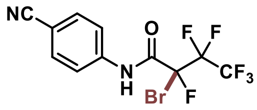

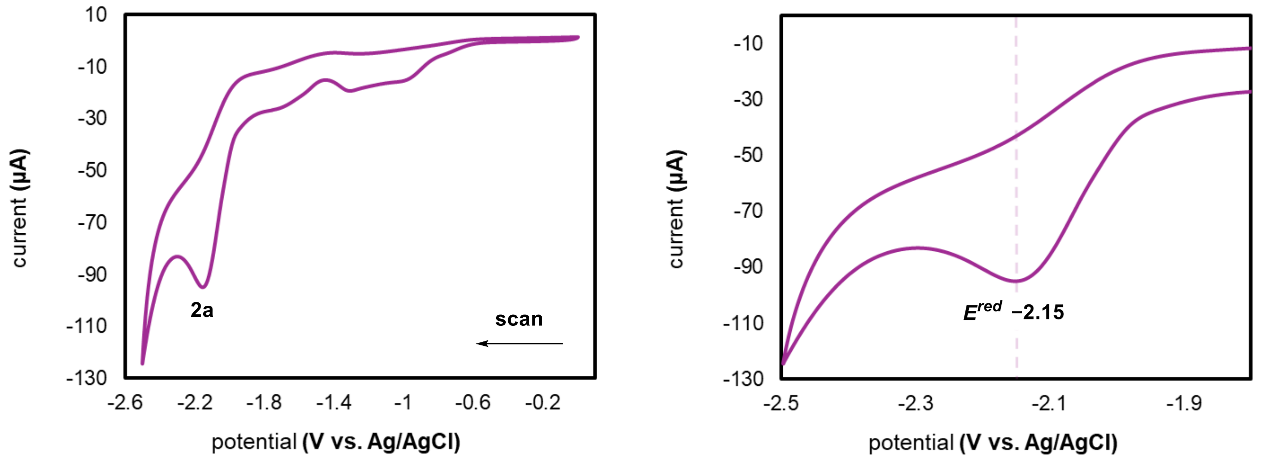

0.5 M **2a** (1.0 M $TBAPF_6$ in MeCN), ν = 100 mV/s. E^{red} = −2.152 V vs. Ag/AgCl; −2.196 V vs. SCE.

[ix] 3a

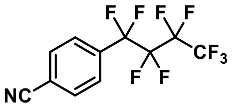

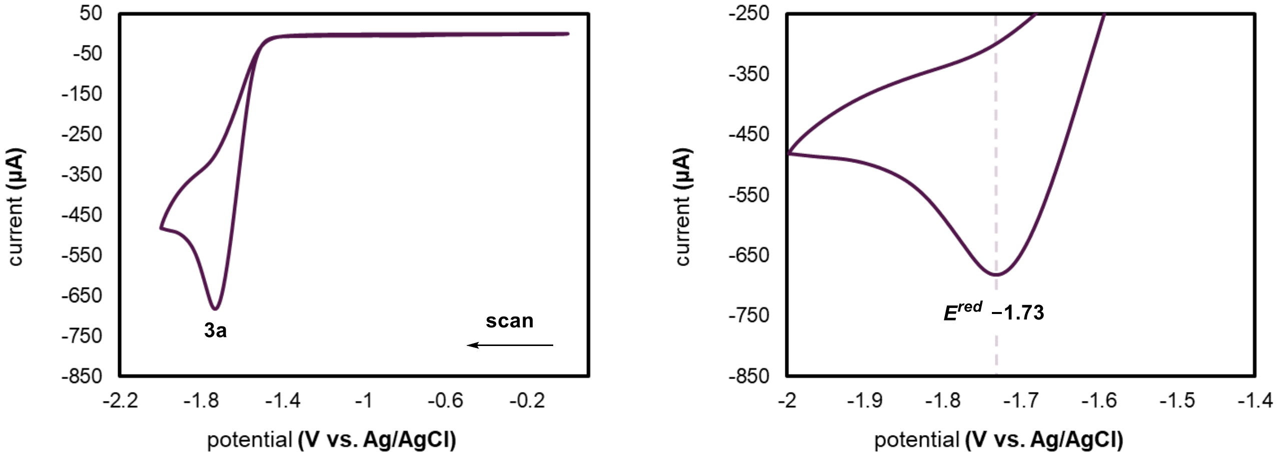

0.5 M **3a** (1.0 M $TBAPF_6$ in MeCN), ν = 100 mV/s. E^{red} = −1.733 V vs. Ag/AgCl; −1.777 V vs. SCE.

b) Randles-Ševčík Plots & Frontier Molecular Orbital Calculations

[i] PC1

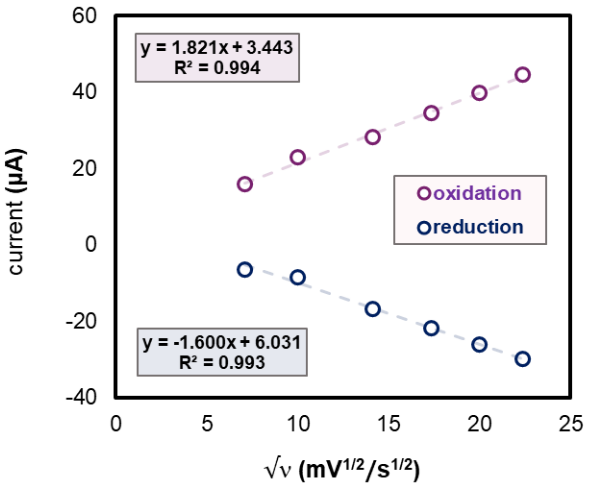

The experiment was performed using 0.5 M **PC1** (1.0 M $TBAPF_6$ in MeCN). The current was measured at the respective oxidation and reduction peak potentials using 5 different scan rates (ν = 50, 100, 200, 300, 500 mV/s). Plotting the square root of the scan rate against the measured currents revealed a strong linear relationship for both the oxidation (slope = 1.821 μA $mV^{1/2}$ $s^{1/2}$) and reduction (slope = −1.600 μA $mV^{1/2}$ $s^{1/2}$) events. Since the slopes of the two curves are nearly equal in value and of opposite sign, this oxidation/reduction event can be classified as quasi-reversible.

Frontier Molecular Orbital Energies

$E_g = (hc)/\lambda_{onset}$
$E_{LUMO} = -e(E_{red,\ onset} + 4.8\ \text{V})$
$E_{HOMO} = E_{LUMO} - E_g$

Where:

h = Planck's constant (4.14×10^{-15} eV)
c = speed of light (2.998×10^{8} m/s)
λ_{onset} = onset absorption wavelength of **PC1** (362×10^{-9} m)
e = charge of an electron (1.602×10^{-19} J; 1 J = 6.242×10^{18} eV)
$E_{red,\ onset}$ = onset reduction potential of **PC1** (0.800 V vs. Ag/AgCl; 0.38 V vs. Fc/Fc^{+})

$E_{g(\mathbf{PC1})}$ = 3.43 eV
$E_{LUMO(\mathbf{PC1})}$ = −5.18 eV
$E_{HOMO(\mathbf{PC1})}$ = −8.61 eV

[ii] PC2

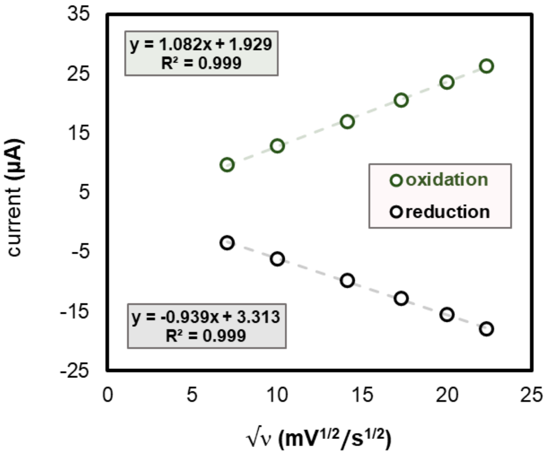

The experiment was performed using 0.5 M **PC2** (1.0 M $TBAPF_6$ in MeCN). The current was measured at the respective oxidation and reduction peak potentials using 5 different scan rates (ν = 50, 100, 200, 300, 500 mV/s). Plotting the square root of the scan rate against the measured currents revealed a strong linear relationship for both the oxidation (slope = 1.082 μA $mV^{1/2}\ s^{1/2}$) and reduction (slope = −0.939 μA $mV^{1/2}\ s^{1/2}$) events. Since the slopes of the two curves are nearly equal in value and of opposite sign, this oxidation/reduction event can be classified as quasi-reversible.

Frontier Molecular Orbital Energies

Calculations were performed in the same manner as for **PC1**.

λ_{onset} = onset absorption wavelength of **PC2** (385 × 10^{-9} m)
$E_{red,\ onset}$ = onset reduction potential of **PC2** (0.891 V vs. Ag/AgCl; 0.471 V vs. Fc/Fc^{+})

$E_{g(\mathbf{PC2})}$ = 3.22 eV
$E_{LUMO(\mathbf{PC2})}$ = −5.27 eV
$E_{HOMO(\mathbf{PC2})}$ = −8.49 eV

12 Excited-State Redox Potential Calculations

Excited-state redox potentials of **PC1** and **PC2** were calculated using the Rehm-Weller equation, as described by Nicewicz and Wasielewski.[19–20] E^{red} (**PC**$^{\bullet+}$/**PC**) values were measured by CV in **Section 11a**. Absorption (**Section 9**) and emission (**Section 10**) data were used for the following plots.

$^*E^{ox}$ (***PC**/**PC**$^{\bullet+}$) = E^{red} (**PC**$^{\bullet+}$/**PC**) – $E_{0,0}$

$^*E^{red}$ (***PC**$^{\bullet+}$/**PC**) = E^{red} (**PC**$^{\bullet+}$/**PC**) + $E_{0,0}$

Where:

$E_{0,0} = (hc)/\lambda_{0,0}$

Where:

$\lambda_{0,0}$ = crossover wavelength, where intersection between the normalized absorbance and emission spectra is observed (roughly ½ of the Stokes shift).

hc = 1240 nm•eV

Note: *for these equations a conversion factor of 1eV = 1V is assumed.*[20]

a) PC1

[i] Excited-State Oxidation Potential (*PC1)

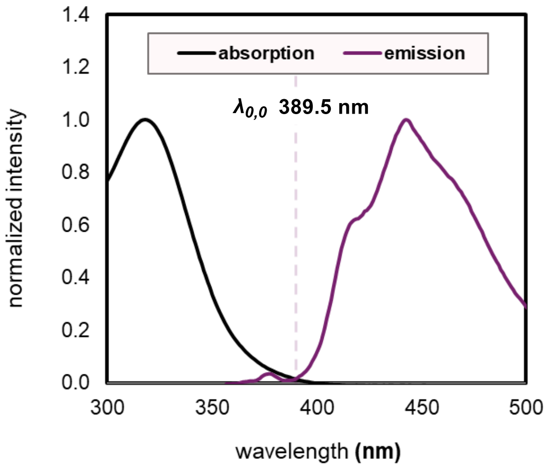

$\lambda_{0,0}$ = 389.5 nm

Therefore:

$E_{0,0}$ = (1240 nm•eV)/389.5 nm = 3.184 eV, or 3.184 V

Therefore:

$^*E^{ox}$ (***PC**/**PC**$^{\bullet+}$) = E^{red} (**PC**$^{\bullet+}$/**PC**) – $E_{0,0}$ = 0.733 V – 3.184 V = **–2.45 V vs. SCE in MeCN**

[ii] Excited-State Reduction Potential (*PC1$^{\bullet+}$)

Note: *the near-IR detector used was unable to measure values below 920 nm, therefore we* ***approximated*** *the $E_{0,0}$ value using an extrapolated emission curve. The emission spectrum closely resembles that of *PTH$^{\bullet+}$.*[19]

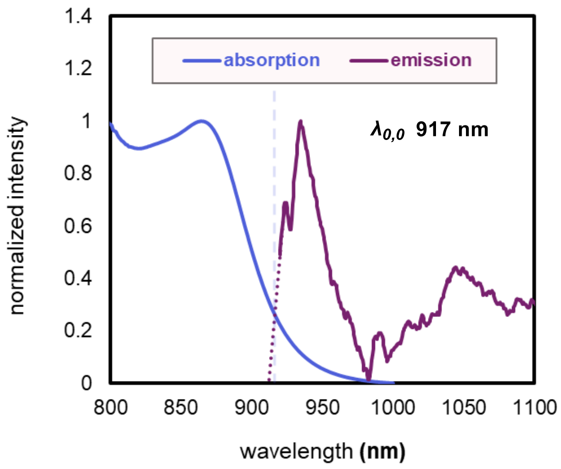

$\lambda_{0,0}$ = ~917 nm

Therefore:

$E_{0,0}$ = (1240 nm•eV)/917 nm = 1.352 eV, or 1.352 V

Therefore:

$^*E^{red}$ (***PC**$^{\bullet+}$/**PC**) = E^{red} (**PC**$^{\bullet+}$/**PC**) + $E_{0,0}$ = 0.733 V + 1.352 V = **+2.09 V vs. SCE in MeCN**

b) PC2

Excited-State Oxidation Potential (*PC2)

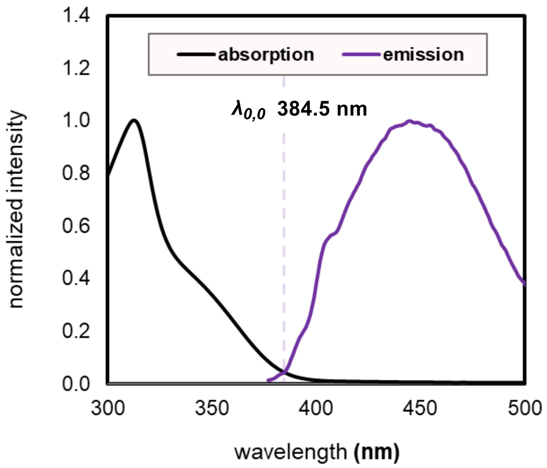

$\lambda_{0,0}$ = 384.5 nm

Therefore:

$E_{0,0}$ = (1240 nm•eV)/384.5 nm = 3.225 eV, or 3.225 V

Therefore:

$^*E^{ox}$ (***PC**/**PC**$^{\bullet+}$) = E^{red} (**PC**$^{\bullet+}$/**PC**) – $E_{0,0}$ = 0.821 V – 3.225 V = **– 2.40 V vs. SCE in MeCN**

13 Femtosecond Transient Absorption Spectroscopy

General Information & Methodology: pump-probe measurements were conducted with a broadband femtosecond transient absorption spectrometer (Spectra-Physics, Newport Corp.) from Helios. A 1 kHz Ti:Sapphire regenerative amplifier (Solstice, Spectra-Physics, Newport Corp.) delivered ultrafast laser pulses (800 nm, <100 fs FWHM) to an optical parametric amplifier (TOPAS Prime, Spectra-Physics) and a frequency mixer (Niruvis, Light Conversion) to generate pump pulses at 533 nm (0.34 μJ cm^{-2}). 800 nm seed pulses were delayed on a 6 ns mechanical delay stage and passed through a sapphire crystal to produce a white light probe spectrum (400–800 nm). Spatial and temporal overlap of focused pump and probe beams was achieved using a 0.5 mm path length cuvette. Laser fluences were calculated based on the probe beam size of 0.5 mm^2 at the sample. The data were analyzed using Surface Xplorer where background and chirp corrections were applied post-measurement. Global analysis was conducted to deconvolute the various spectral components. Early times were used as a reference, and the algorithm was left to freely optimize the second component. Measurements were recorded using a solution of **PC1•PF$_6$** in anhydrous MeCN (1 mM) under ambient conditions. The synthesis of **PC1•PF$_6$** is described in **Section 8e**.

a) PC1$^{•+}$ Pump-Probe Measurement

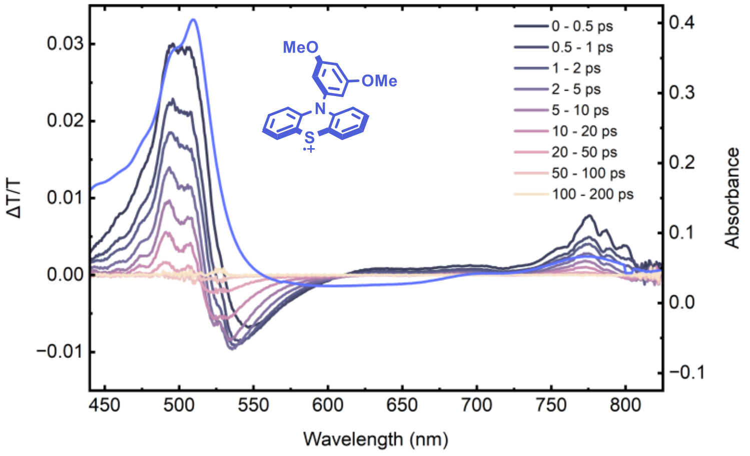

Scanning femtosecond transient absorption spectra of **PC1•PF$_6$** in MeCN (1 mM) recorded at different delay times using an excitation pump pulse of 533 nm. The transient absorption spectra are overlayed with the absorption spectrum of **PC1$^{•+}$** as previously described in **Section 8e/9b**.

b) Global Analysis

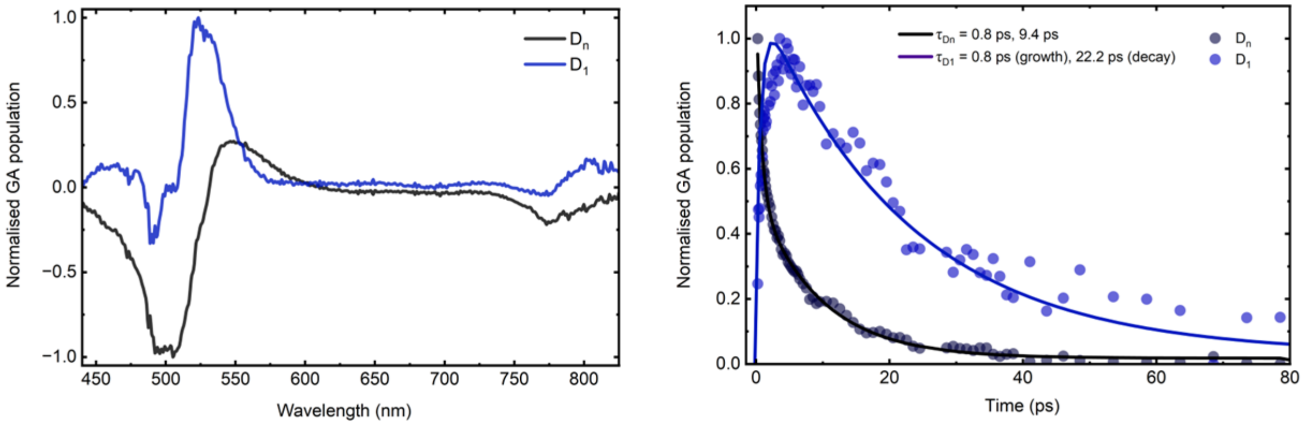

Shown above are deconvoluted spectra of the time-resolved pump-probe measurement conducted on **PC1•PF$_6$** in MeCN (1 mM); normalized spectral components followed by their associated kinetics. Two components were observed (denoted D_n & D_1), and exponential fitting was performed to track their respective decay profiles using global analysis. Biexponential fitting of component D_n revealed two distinct decay events (τ_1 = 0.8 ps, τ_2 = 9.4 ps). Exponential fitting of component D_1 revealed a growth event (τ = 0.8 ps, corresponding to the τ_1 decay of D_n) and a decay event (τ = 22.2 ps). In accordance with the literature which assigns the $D_1 \rightarrow D_0$ excited-state decay of *PTH$^{\cdot+}$ to be 32.3 ps when excited with a 517 nm laser,[19] the obtained lifetime value of τ = 22.2 ps for ***PC1$^{\cdot+}$** represents decay from the lowest energy doublet excited state back to the doublet ground state ($D_1 \rightarrow D_0$).

14 Computational Analysis

General Information: computational calculations were performed using the University of Oxford Advanced Research Computing (ARC) facility.[21] For initial guess structure generation, conformational sampling was performed using the Global Optimizer Algorithm (GOAT) implemented in the ORCA 6.1.0 software package,[22–23] using a GFN2-xTB Hamiltonian.[24–25] Conformational sampling was performed in acetonitrile using the analytical linearized Poisson–Boltzmann (ALPB) solvation model.[26] A 6.0 kcal/mol energy window was applied during sampling.

Geometry optimizations and vibrational frequency calculations were performed using Gaussian 16, rev. C.01[27] at the M06-2X-D3/def2-TZVP[28–30] level of theory. Solvation in acetonitrile was modeled using the continuum solvation model based on solute electron density (SMD),[31] a variation of the integral equation formalism variant of the polarizable continuum model (IEFPCM).[32] Minima were identified by the absence of imaginary frequency vibrational modes; transition structures by the presence of a single imaginary mode. Single-point energy calculations were performed using ORCA at the ωB97X-D3/def2-TZVPPD/SMD(MeCN)[33] level of theory. All bond lengths are reported in Ångstroms (Å). Thermochemistry was evaluated at 298.15 K and a concentration of 0.20 M using Paton's GoodVibes script.[34] The entropic contribution of low vibrational modes was corrected using a frequency cutoff value of 100 cm^{-1}.[35] The minimum energy crossing point (MECP) between triplet and singlet was calculated using ORCA with keywords "SurfCrossOpt" and "SurfCrossNumFreq". Natural Bonding Orbital (NBO) analysis was performed using NBO7.0 integrated in ORCA.[36]

Time-dependent density functional theory (TD-DFT) calculations were performed using ORCA at the CAM-B3LYP/def2-TZVPPD[37–38] level of theory, unless specified otherwise. 20 excited states were calculated. Natural transition orbitals (NTOs) were obtained applying a contribution threshold of 10^{-3}.

Dynamic vertical triplet energy (DvTE) was calculated by adopting the method originally developed by Paton and co-workers.[39] Initial geometry optimization was performed using Gaussian 16 at the M06-2X/6-31(d)[40] level of theory with the SMD solvation model. 50 quasiclassical molecular dynamics (MD) trajectories were generated using the Milo[41] package developed by Ess and co-workers, interfaced with Gaussian 16. Each MD simulation was run for 500 fs using a time step of 1 fs at the M06-2X/MIDI![42] level of theory with the SMD solvation model at 298.15 K. Through the total 500 fs, snapshots were extracted every 8 fs, generating a total of 3150 geometries. For each geometry, single point energies of singlet S_0 and triplet T_1 were calculated using Gaussian 16 at the M06-2X/6-31(d) level of theory with the SMD solvation model. Vertical S_0–T_1 gaps of the snapshot geometries were extracted and plotted in a histogram with an approximated normal distribution using matplotlib[43] and scikit-learn,[44] respectively. The DvTE was determined by including 0.8% of the distribution.

a) Conformational Analysis of PC1 and PC1$^{\bullet+}$

Ground-state *N*-arylphenothiazines have been reported to adopt two possible conformations: quasi-equatorial (*intra*) and quasi-axial (*extra*), depending on the aryl substituents.[45]

DFT calculations suggest that both ground-state **PC1** and radical cation **PC1$^{\bullet+}$** prefer the *intra* conformation ($\Delta G_{extra-intra}$ = +0.9 and +11.4 kcal/mol respectively).

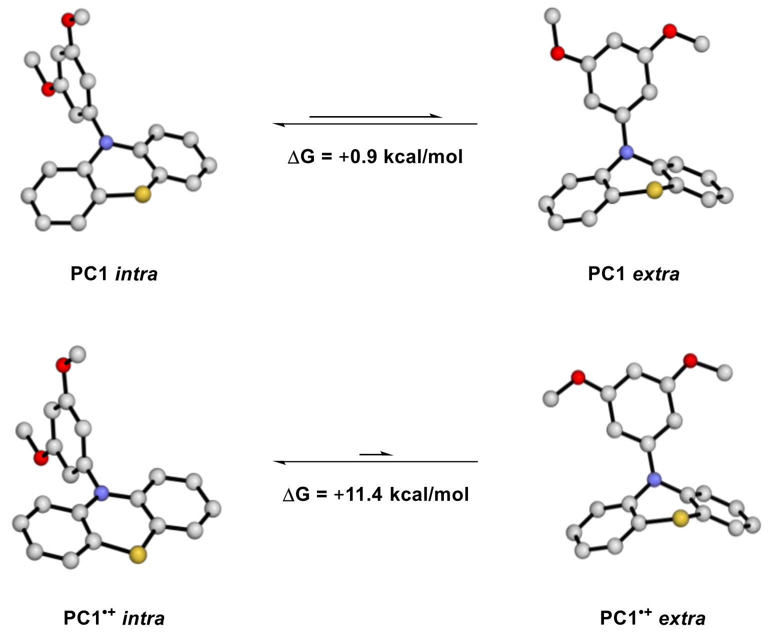

b) TD-DFT Calculations of PC1 and PC1•+
[i] PC1

TD-DFT calculations were performed on the **PC1** excited-state *intra* conformer (***PC1**). Comparison between calculated absorption spectral data and the experimentally recorded UV-Vis absorption spectrum revealed that the CAM-B3LYP functional better reproduces the experimental data.

functional	$\lambda_{max,abs}$[a] (nm) (ε [M^{-1} cm^{-1}])	$\lambda_{max,calcd}$ (nm)	Energy (eV)	Oscillator strength (*f*)	Molecular orbital contribution
ωB97X	318 (3880)	297.3	4.170	0.004	**87a (HOMO) → 88a (LUMO): 0.846**<br>87a (HOMO) → 90a (LUMO+3): 0.041
ωB97X-D3		302.8	4.095	0.005	**87a (HOMO) → 88a (LUMO+3): 0.867**<br>87a (HOMO) → 90a (LUMO+3): 0.034
CAM-B3LYP		313.0	3.961	0.005	**87a (HOMO) → 88a (LUMO): 0.893**<br>87a (HOMO) → 90a (LUMO+3): 0.032
CAM-B3LYP-D3BJ		313.0	3.961	0.004	**87a (HOMO) → 88a (LUMO): 0.893**<br>87a (HOMO) → 90a (LUMO+3): 0.032

Table S1. TD-DFT calculations of the first excited state for the *intra* conformer of **PC1**. [a]Recorded in MeCN, T = 298 K, c = 100 μM.

[ii] $PC1^{\cdot+}$

The CAM-B3LYP functional was used for TD-DFT calculations of radical cation **$PC1^{\cdot+}$**. Both the *intra* and *extra* conformers are reported. The energy corresponding to the transition at 363.0 nm ($D_6 \leftarrow D_0$, f = 0.009) for the *intra* conformer of **$PC1^{\cdot+}$** was used to estimate the activation energy of **$PC1^{\cdot+}$** to **$*PC1^{\cdot+}$** in the Gibbs free energy profile.

$PC1^{\cdot+}$ (*intra*)

Excited state	Wavelength (nm)	Energy (eV)	Oscillator strength (f)	Molecular orbital contribution
1	632.0	1.962	0.040	**85b → 87b: 0.955**
2	536.2	2.312	0.008	82b → 87b: 0.018<br>83b → 87b: 0.014<br>**86b → 87b: 0.954**
3	433.2	2.862	0.269	87a → 88a: 0.011<br>**83b → 87b: 0.922**<br>85b → 88b: 0.011<br>86b → 87b: 0.013
4	412.0	3.009	0.000(2)	81b → 87b: 0.050<br>**84b → 87b: 0.928**
5	396.2	3.129	0.001	84a → 90a: 0.015<br>87a → 88a: 0.021<br>**82b → 87b: 0.882**<br>85b → 90b: 0.016<br>86b → 87b: 0.022
6	363.0	3.415	0.009	82a → 95a: 0.011<br>84a → 90a: 0.019<br>**87a → 88a: 0.860**<br>83b → 87b: 0.011
7	312.4	3.969	0.000(0)	81a → 91a: 0.010<br>85a → 91a: 0.130<br>86a → 88a: 0.012<br>86a → 90a: 0.042<br>**86a → 91a: 0.152**<br>86a → 97a: 0.103<br>84b → 90b: 0.016<br>84b → 91b: 0.119<br>86b → 88b: 0.013<br>86b → 90b: 0.086<br>86b → 91b: 0.114<br>86b → 97b: 0.099
8	304.8	4.067	0.006	82a → 88a: 0.043<br>82a → 90a: 0.044<br>83a → 90a: 0.013<br>84a → 89a: 0.101

				**87a → 89a: 0.378**<br>87a → 95a: 0.026<br>75b → 87b: 0.010<br>80b → 87b: 0.083<br>82b → 88b: 0.018<br>82b → 90b: 0.029<br>83b → 88b: 0.070<br>85b → 89b: 0.090

Table S2. Selected data for TD-DFT calculations of the **PC1** excited-state radical cation (***PC1·+**, *intra*). The weight of the individual excitation is listed if the value is larger than 0.01. In unrestricted DFT calculations, up (α) and down (β) spins for each electron pair differ in special orbitals and hence the notation, such as HOMO and LUMO, are not used in the orbital contribution.

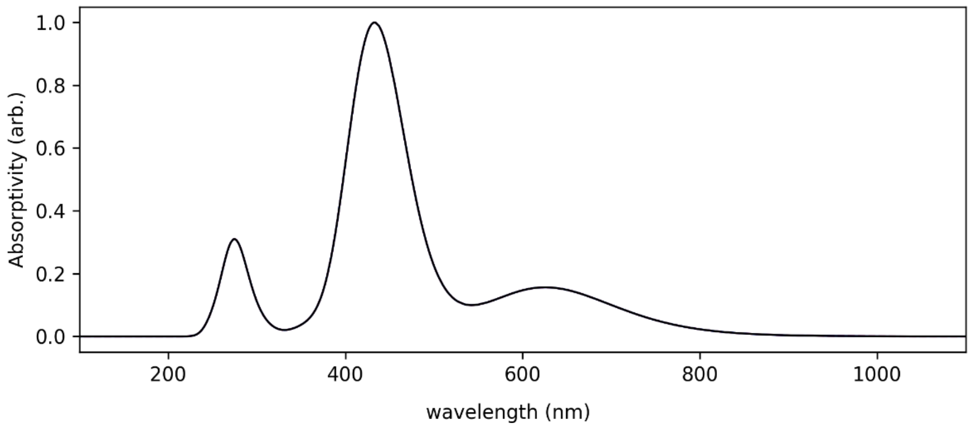

PC1·+ (*extra*)

Excited state	Wavelength (nm)	Energy (eV)	Oscillator strength (*f*)	Molecular orbital contribution
1	993.9	1.247	0.000(0)	**85b → 87b: 0.965**
2	715.7	1.732	0.135	84b → 87b: 0.075<br>**86b → 87b: 0.896**
3	540.0	2.296	0.119	81b → 87b: 0.027<br>**84b → 87b: 0.833**<br>86b → 87b: 0.083
4	462.9	2.678	0.044	71b → 87b: 0.013<br>**83b → 87b: 0.950**
5	394.6	3.142	0.000(0)	82b → 87b: 0.962
6	363.8	3.408	0.006	85a → 96a: 0.015<br>68b → 87b: 0.011<br>80b → 87b: 0.011<br>**81b → 87b: 0.848**<br>84b → 87b: 0.033
7	314.5	3.942	0.014	82a → 88a: 0.019

				83a → 90a: 0.046<br>83a → 91a: 0.030<br>84a → 90a: 0.044<br>84a → 91a: 0.019<br>86a → 88a: 0.020<br>86a → 89a: 0.036<br>87a → 88a: 0.051<br>87a → 89a: 0.151<br>87a → 92a: 0.044<br>81b → 87b: 0.012<br>81b → 88b: 0.015<br>82b → 89b: 0.047<br>82b → 91b: 0.030<br>83b → 89b: 0.051<br>83b → 91b: 0.018<br>84b → 88b: 0.028<br>**86b → 90b: 0.190**<br>86b → 95b: 0.012
8	313.0	3.961	0.001	82a → 91a: 0.018<br>83a → 89a: 0.080<br>84a → 88a: 0.051<br>84a → 92a: 0.026<br>86a → 91a: 0.033<br>**87a → 90a: 0.245**<br>81b → 91b: 0.010<br>82b → 90b: 0.072<br>83b → 88b: 0.055<br>84b → 91b: 0.028<br>86b → 89b: 0.188<br>86b → 91b: 0.019

Table S3. Selected data for TD-DFT calculations of the **PC1** excited-state radical cation (***PC1**$^{\cdot+}$, *extra*). The weight of the individual excitation is listed if the value is larger than 0.01. In unrestricted DFT calculations, up (α) and down (β) spins for each electron pair differ in special orbitals and hence the notation, such as HOMO and LUMO, are not used in the orbital contribution.

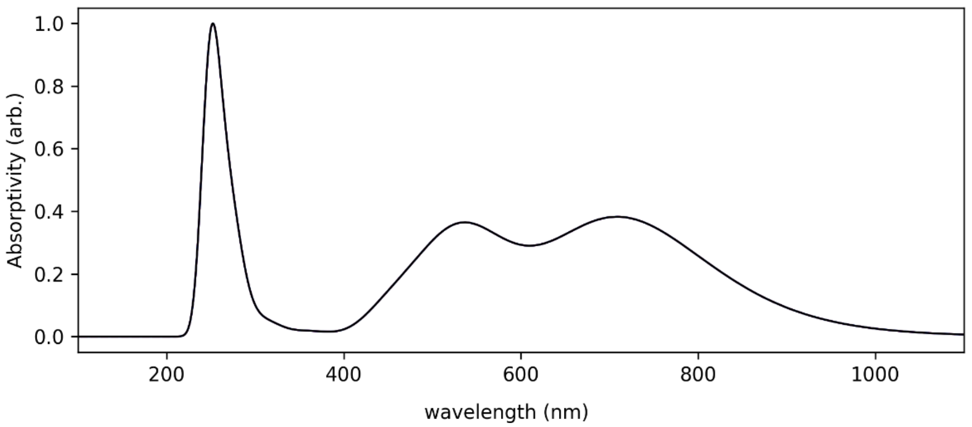

c) Gibbs Free Energy Profiles

In the computed Gibbs free energy profile, the energies for excited states ***PC1** and ***PC1**$^{\bullet+}$ were derived from TD-DFT calculations using the CAM-B3LYP functional.

[i] Computed Profile of Bromodefluorination (kcal/mol)

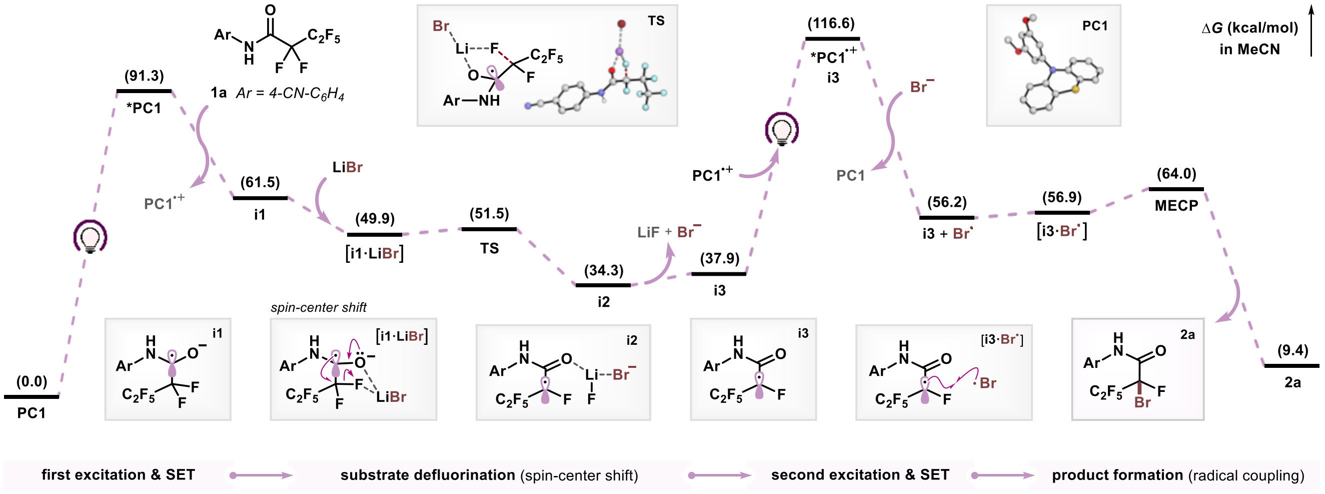

[ii] Computed Activation Barrier for Spin-Center Shift of i1

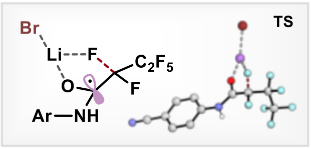

TS (with LiBr): $\Delta G^{\ddagger}$ **= 1.6 kcal/mol**

TS' (w/o LiBr): $\Delta G^{\ddagger}$ **= 3.1 kcal/mol**

When assisted by LiBr, the spin-center shift event displays a more stable transition state structure compared to the unassisted pathway ($\Delta\Delta G^{\ddagger}$ = −1.5 kcal/mol).

[iii] Alternative Pathways for Bromide Oxidation

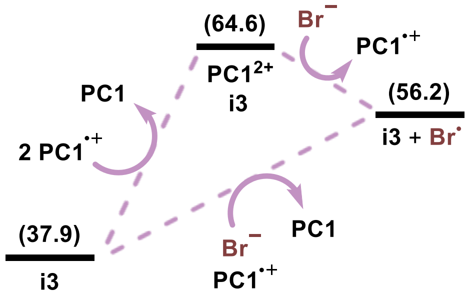

Pathways leading to the oxidation of bromide by ground-state **PC1**$^{\cdot+}$ (ΔG = +18.3 kcal/mol) or **PC1**$^{2+}$ (ΔG = +26.7 kcal/mol) are thermodynamically demanding and therefore less likely contributors for productive bromodefluorination.

[iv] Computed Pathways for Defluorination of Trifluoroacetamide 1aa

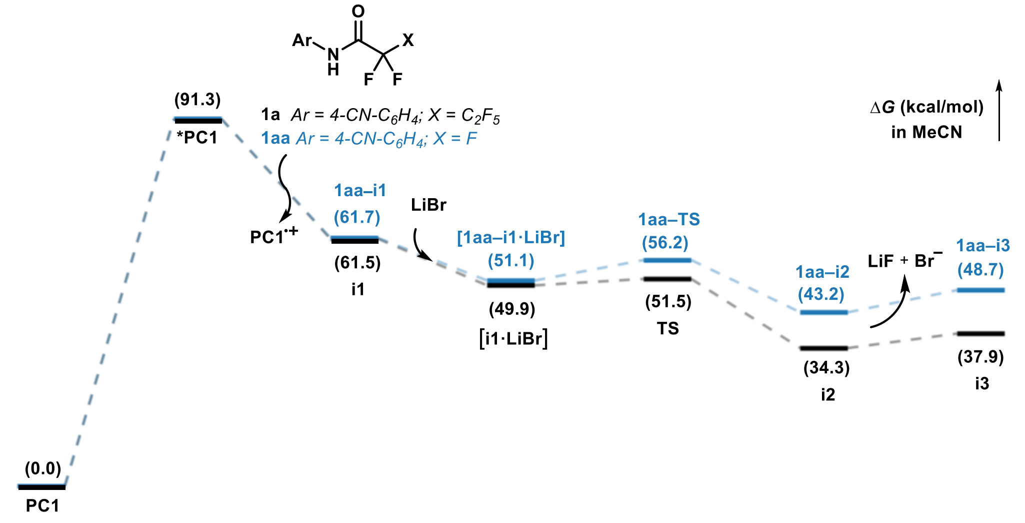

The activation barrier for defluorination of **1aa** is larger compared to that of model substrate **1a** ($\Delta\Delta G$ = +3.5 kcal/mol). In addition, formation of radical **1aa–i3** from the ground state is less favorable than the corresponding radical from **1a** ($\Delta\Delta G$ = +10.8 kcal/mol).

d) Natural Bond Orbital (NBO) Analysis of Radical Anion 2a'

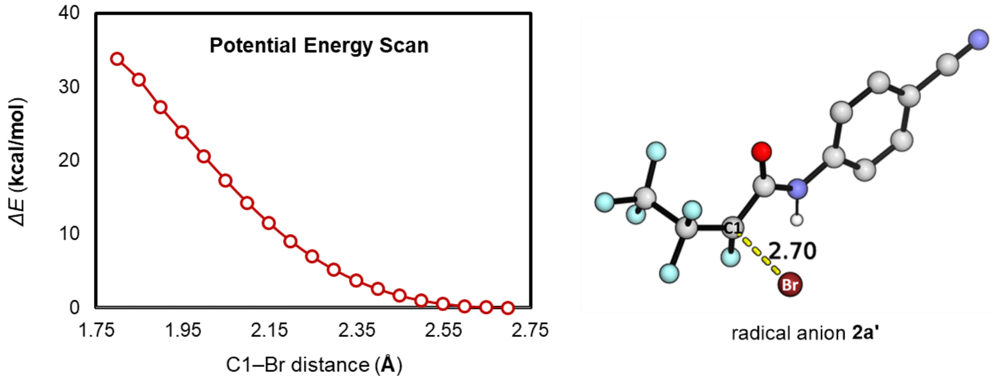

	Natural charge	Natural spin density
C1	0.302	0.582
Br	–0.732	0.245

Table S4. NBO analysis of radical anion **2a'**.

Upon quenching **PC1**, the resultant radical anion of brominated product **2a** is predicted to form a species where the C–Br distance is 2.70 Å. NBO analysis revealed that the Wiberg bond index (WBI)

for the C–Br bond is 0.303, and the natural charge on Br is –0.732. This charge depletion by Br may contribute to the challenging defluorination, leaving **2a** intact.

e) TD-DFT Calculations of PC2$^{\bullet+}$ and Natural Transition Orbitals (NTO)

TD-DFT calculations of **PC2**$^{\bullet+}$ revealed two primary transitions at 580 nm ($D_2 \leftarrow D_0$, f = 0.091) and 502 nm ($D_3 \leftarrow D_0$, f = 0.133) respectively, which overlap with the emission profile of the green LED source used (see **Section 10a** for LED emission profiles).

Excited state	Wavelength (nm)	Energy (eV)	Oscillator strength (f)	Molecular orbital contribution
1	936.8	1.323	0.089	**125b → 126b: 0.971**
2	579.5	2.139	0.091	**124b → 126b: 0.954**
3	501.5	2.472	0.133	**123b → 126b: 0.947**
4	462.3	2.682	0.167	**122b → 126b: 0.934**
5	396.7	3.125	0.001	**121b → 126b: 0.939**

Table S5. Selected data for TD-DFT calculations of the **PC2** excited-state radical cation (***PC2**$^{\bullet+}$). The weight of the individual excitation is listed if the value is larger than 0.01. In unrestricted DFT calculations, up (α) and down (β) spins for each electron pair differ in special orbitals and hence the notation, such as HOMO and LUMO, are not used in the orbital contribution.

Below are NTOs for the second and third excited states of **PC2**$^{\bullet+}$. Contribution of the NTO donor–acceptor pair toward the transition is labeled in brackets (97%).

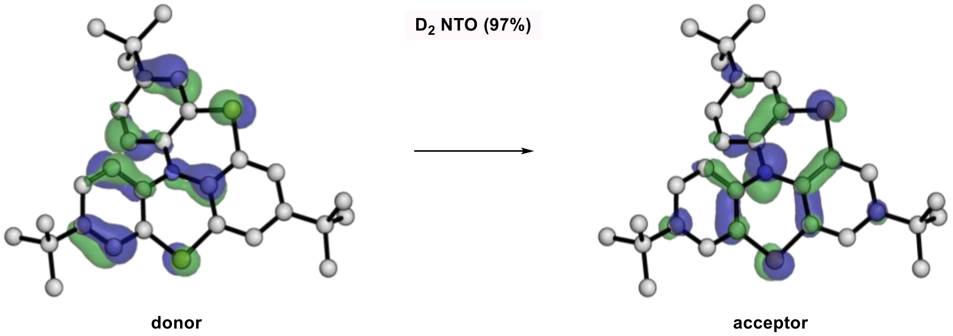

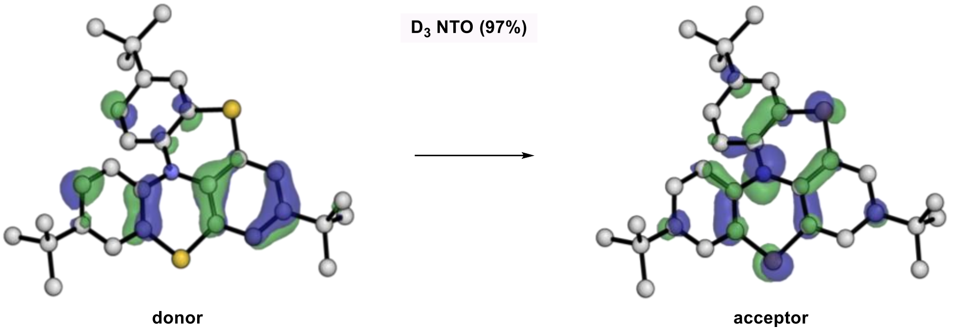

f) Dynamic Vertical Triplet Energy (DvTE)

The histogram for the DvTE was obtained from geometries in molecular dynamics simulations of **1a**.

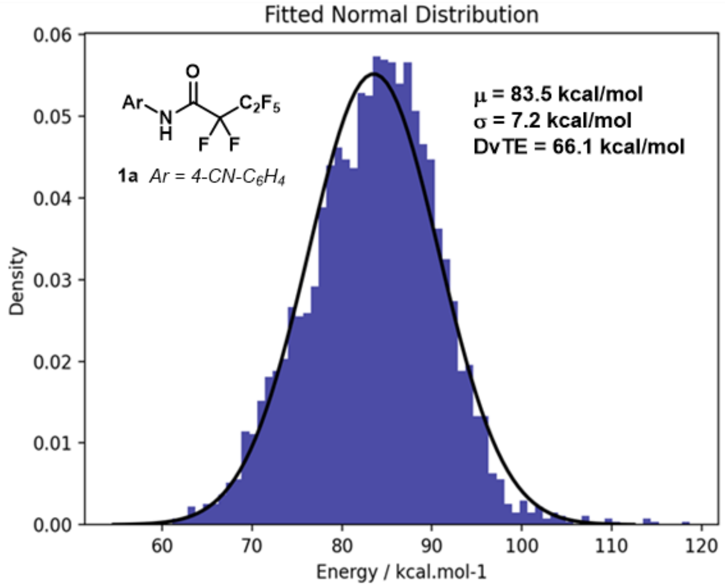

g) XYZ Coordinates

PC1 *intra*

C 2.605025 3.661842 -0.564572

C 3.302421 2.535918 -0.148897

C 2.636410 1.341610 0.088142

C 1.258596 1.228282 -0.139830

C 0.569043 2.368419 -0.558901

C 1.235382 3.570849 -0.755903

C 1.258708 -1.228316 -0.139605

C 2.636533 -1.341471 0.088393

C 3.302661 -2.535759 -0.148418

H 4.371780 -2.584523 0.020716

C 2.605375 -3.661830 -0.563884

C 1.235725 -3.571006 -0.755233

C 0.569268 -2.368603 -0.558460

H 3.127747 4.594830 -0.728535

H 4.371537 2.584818 0.020222

H -0.497174 2.323876 -0.725633

H 0.669060 4.438055 -1.070988

H 3.128189 -4.594798 -0.727667

H 0.669487 -4.438326 -1.070154

H -0.496953 -2.324200 -0.725200

S 3.522348 0.000178 0.808196

N 0.599913 -0.000026 0.077937

C -0.836648 -0.000066 0.060565

C -1.520967 -0.000244 -1.136240

C -1.495689 0.000123 1.287306

C -2.919664 -0.000246 -1.116478

H -0.993736 -0.000380 -2.081741

C -2.886230 0.000137 1.289549

H -0.914233 0.000264 2.197294

C -3.603941 -0.000053 0.089862

H -4.683269 -0.000034 0.138830

O -3.525549 -0.000435 -2.323531

O -3.635646 0.000331 2.413236

C -2.957540 0.000598 3.659985

H -3.731312 0.000754 4.423558

H -2.337324 -0.891890 3.768958

H -2.337336 0.893140 3.768579

C -4.944025 -0.000497 -2.353800

H -5.346936 0.892084 -1.869922

H -5.222724 -0.000684 -3.404513

H -5.346863 -0.892948 -1.869623

PC1 *extra*

C -2.667529 -3.696571 0.211520

C -2.837318 -2.682649 -0.719411

C -2.146583 -1.484835 -0.565566

C -1.294912 -1.298682 0.522071

C -1.167920 -2.301834 1.475546

C -1.840288 -3.503913 1.312743

C -1.385256 1.094778 0.488024

C -2.247329 1.184776 -0.603886

C -3.024950 2.322826 -0.792506

H -3.700909 2.380023 -1.636837

C -2.932289 3.373920 0.107580

C -2.095588 3.275687 1.213990

C -1.336851 2.131629 1.412484

H -3.198701 -4.631381 0.086699

H -3.506693 -2.814400 -1.560607

H -0.521977 -2.134693 2.328765

H -1.724644 -4.287925 2.049822

H -3.531010 4.262669 -0.044915

H -2.040176 4.087241 1.927912

H -0.682972 2.037957 2.270965

S -2.344993 -0.172045 -1.732558

N -0.582024 -0.071449 0.645948

C 0.794457 -0.028482 0.348606

C 1.525878 -1.202153 0.207281

C 1.438231 1.212561 0.194052

C 2.894158 -1.140184 -0.071202

H 1.073052 -2.177351 0.299118

C 2.795870 1.242888 -0.083309

H 0.882024 2.129969 0.281459

C 3.548071 0.071841 -0.215584

H 4.603545 0.148698 -0.428710

O 3.510581 -2.339588 -0.186931

O 3.491043 2.392657 -0.247656

C 2.794394 3.620781 -0.122283

H 3.533318 4.403147 -0.277411

H 2.009149 3.708145 -0.877023

H 2.356852 3.726365 0.873442

C 4.897681 -2.344600 -0.479274

H 5.191221 -3.390108 -0.533100

H 5.099657 -1.860030 -1.437400

H 5.467773 -1.846706 0.308542

PC1$^{\bullet+}$ *intra*

C 2.501488 3.746555 -0.085293

C 3.266837 2.608827 -0.100804

C 2.656445 1.346790 -0.095871

C 1.250511 1.228379 -0.076324

C 0.488368 2.414896 -0.059839

C 1.103837 3.640509 -0.064183

C 1.250385 -1.228336 -0.076713

C 2.656308 -1.346887 -0.096268

C 3.266567 -2.608986 -0.101567

H 4.347774 -2.670846 -0.117548
C 2.501099 -3.746639 -0.086423
C 1.103459 -3.640455 -0.065336
C 0.488119 -2.414778 -0.060635
H 2.975733 4.718430 -0.089086
H 4.348051 2.670576 -0.116800
H -0.589382 2.359553 -0.044111
H 0.495428 4.534928 -0.051432
H 2.975244 -4.718563 -0.090485
H 0.494955 -4.534814 -0.052880
H -0.589626 -2.359332 -0.044931
S 3.720749 -0.000100 -0.116168
N 0.615748 0.000054 -0.072865
C -0.833710 0.000110 -0.054076
C -1.501041 0.000427 -1.252954
C -1.466527 -0.000209 1.182447
C -2.902706 0.000434 -1.223985
H -0.970229 0.000661 -2.195632
C -2.856932 -0.000237 1.186523
H -0.877592 -0.000447 2.087651
C -3.577153 0.000095 -0.013435
H -4.656191 0.000057 0.040840
O -3.510897 0.000757 -2.424903
O -3.600683 -0.000575 2.308826
C -2.920141 -0.001201 3.556034
H -3.693354 -0.001502 4.319783
H -2.300761 -0.894467 3.661833
H -2.300631 0.891874 3.662678
C -4.931254 0.000866 -2.451238
H -5.330609 0.893671 -1.965520
H -5.211395 0.001211 -3.501288
H -5.330738 -0.892182 -1.966073

PC1$^{\bullet+}$ *extra*

C -2.718487 -3.504848 0.363806
C -2.995173 -2.494489 -0.542837
C -2.244219 -1.323915 -0.522519
C -1.199465 -1.199157 0.392002
C -0.956459 -2.188546 1.337297
C -1.715924 -3.345285 1.315504
C -1.199391 1.199208 0.391995
C -2.244138 1.324026 -0.522526
C -2.995017 2.494648 -0.542853
H -3.804799 2.599352 -1.253673
C -2.718261 3.504998 0.363779
C -1.715702 3.345381 1.315473
C -0.956312 2.188593 1.337275
H -3.310167 -4.410730 0.347526
H -3.804960 -2.599148 -1.253658
H -0.175167 -2.044253 2.072540
H -1.528812 -4.120902 2.045848
H -3.309881 4.410919 0.347490
H -1.528533 4.120995 2.045804
H -0.175022 2.044259 2.072512
S -2.616646 0.000064 -1.621444
N -0.420293 0.000002 0.346887
C 0.923648 -0.000033 0.152815
C 1.617976 -1.234994 0.027069
C 1.618033 1.234899 0.027078
C 2.980018 -1.219729 -0.156091
H 1.080719 -2.166543 0.046733
C 2.980077 1.219576 -0.156081
H 1.080813 2.166470 0.046748
C 3.676630 -0.000093 -0.234444
H 4.747553 -0.000120 -0.390129

O 3.739891 -2.312614 -0.286007
O 3.740014 2.312422 -0.285984
C 3.096721 3.581790 -0.232455
H 3.885805 4.320409 -0.339510
H 2.380032 3.685051 -1.049643
H 2.590067 3.717020 0.725406
C 3.096500 -3.581932 -0.232500
H 2.589861 -3.717152 0.725370
H 2.379779 -3.685103 -1.049672
H 3.885520 -4.320613 -0.339603

1a

C 2.387011 -0.600394 0.788751
C 1.889785 0.479640 0.060000
C 2.736953 1.228278 -0.760622
C 4.073299 0.906064 -0.856563
C 4.573492 -0.174528 -0.127731
C 3.729537 -0.921824 0.689632
H 1.740616 -1.183833 1.422825
H 2.336780 2.064082 -1.321662
H 4.730864 1.484884 -1.491019
H 4.123855 -1.758959 1.249968
C 5.961154 -0.517481 -0.222589
N 7.073614 -0.792198 -0.299259
N 0.541380 0.871501 0.089798
H 0.305240 1.646493 -0.519082
C -0.444036 0.371229 0.856199
O -0.377886 -0.505887 1.683707
C -1.829073 1.007652 0.578876
C -2.642187 0.291944 -0.528029
C -2.827430 -1.232930 -0.354529
F -2.548139 0.988096 1.705843
F -1.710089 2.290103 0.177269
F -3.857349 0.851599 -0.577233
F -2.026090 0.493745 -1.701990
F -3.685609 -1.669031 -1.265551
F -1.670691 -1.856739 -0.530226
F -3.298729 -1.518506 0.849132

LiBr

Li 0.000000 0.000000 -2.110300
Br 0.000000 0.000000 0.180883

i1

C -2.623126 -0.141221 -1.406954
C -1.811318 0.333983 -0.338139
C -2.421225 0.553556 0.928070
C -3.755780 0.310000 1.103371
C -4.576100 -0.168007 0.035970
C -3.953261 -0.385518 -1.233417
H -2.165974 -0.310919 -2.376246
H -1.824219 0.914495 1.750829
H -4.203626 0.482888 2.074566
H -4.547803 -0.748499 -2.062750
C -5.940056 -0.413934 0.224678
N -7.074902 -0.618115 0.381972
N -0.470558 0.558033 -0.603699
H -0.186318 0.342450 -1.552065
C 0.489567 1.034697 0.210374
O 0.366150 1.386506 1.390618
C 1.880918 1.078422 -0.405414
C 2.891652 0.076323 0.207084
C 2.470541 -1.409567 0.156822
F 1.874035 0.856577 -1.741393

F 2.432478 2.300920 -0.207205

F 4.055834 0.179917 -0.460875

F 3.116133 0.386291 1.489498

F 2.222768 -1.789911 -1.089739

F 3.456669 -2.156128 0.638546

F 1.387205 -1.618142 0.893185

i1·LiBr

C 2.469766 0.961386 0.219453

C 2.470057 -0.016075 -0.799825

C 3.711002 -0.441838 -1.326635

C 4.892676 0.072480 -0.855886

C 4.885391 1.043573 0.157421

C 3.660631 1.474964 0.682036

H 1.533551 1.300674 0.632275

H 3.718598 -1.188645 -2.111926

H 5.834104 -0.268443 -1.267286

H 3.650856 2.220716 1.467150

C 6.108313 1.582624 0.646001

N 7.098634 2.018180 1.041340

N 1.333951 -0.590455 -1.290464

H 1.482181 -1.324416 -1.971845

C 0.005798 -0.175333 -1.133656

O -0.323544 0.938476 -0.577372

C -0.988333 -1.287342 -1.097759

C -1.046555 -2.172083 0.175082

C -1.087657 -1.389546 1.506181

F -2.240803 -0.744409 -1.223171

F -0.814829 -2.170511 -2.117708

F -2.129266 -2.972889 0.142249

F 0.046090 -2.953203 0.209775

F -1.345764 -2.225508 2.503599

F 0.077141 -0.804408 1.740440

F -2.042119 -0.460060 1.484411

Li -2.077580 1.367065 -0.421138

Br -4.024768 2.591973 -0.080370

TS

N -1.302111 -0.852666 0.715151

C -0.071023 -0.410759 0.252689

O 0.095389 0.572766 -0.553955

C 1.104337 -0.904741 0.936826

F 1.757924 0.261232 1.652554

F 0.890549 -1.816354 1.890431

Br 2.610718 3.664676 -0.076299

Li 1.476982 1.637492 0.109867

H -1.271668 -1.647151 1.340798

C -2.556304 -0.419119 0.390056

C -3.649550 -1.092839 0.980806

C -2.817870 0.650461 -0.491586

C -4.940629 -0.715503 0.712059

H -3.455170 -1.916201 1.657770

C -4.118483 1.021403 -0.755444

H -1.998191 1.172435 -0.956904

C -5.194631 0.351706 -0.163003

H -5.766292 -1.240107 1.175299

H -4.310413 1.844317 -1.432323

C -6.532898 0.747113 -0.445647

N -7.615514 1.066517 -0.673437

C 2.300668 -1.355731 0.075250

C 1.980923 -2.494437 -0.919258

F 2.783474 -0.322198 -0.640368

F 3.281156 -1.801101 0.878995

F 1.182474 -2.075535 -1.889000

F 1.398038 -3.506892 -0.286896

F 3.109569 -2.931804 -1.465784

i2

N -0.707760 -1.742198 0.290111

C 0.391919 -1.042142 -0.059321

O 0.385149 0.017928 -0.681968

C 1.647718 -1.630653 0.375897

F 1.260727 0.443717 1.976313

F 1.617537 -2.690537 1.161731

Br -0.348758 3.494744 0.262618

Li 0.793038 1.446978 0.658629

H -0.567575 -2.650065 0.717535

C -2.044929 -1.373914 0.082901

C -2.999533 -2.384392 0.226667

C -2.442801 -0.069074 -0.215908

C -4.339467 -2.106157 0.063394

H -2.677805 -3.390142 0.466836

C -3.788657 0.206518 -0.378899

H -1.723977 0.730160 -0.310842

C -4.737803 -0.804508 -0.245766

H -5.077373 -2.889616 0.171960

H -4.104284 1.216425 -0.605438

C -6.127660 -0.506024 -0.420980

N -7.242442 -0.268088 -0.562223

C 3.052256 -1.224871 0.063762

C 3.311999 -0.001575 -0.849633

F 3.663448 -2.270379 -0.553143

F 3.733720 -1.014688 1.207587

F 2.874824 1.134175 -0.316641

F 2.777105 -0.170170 -2.045918

F 4.629352 0.109614 -0.996567

i3

C 3.004854 1.433590 -0.262464

C 2.077318 0.426898 0.030330

C 2.516627 -0.878094 0.266923

C 3.869913 -1.160460 0.207946

C 4.789421 -0.156405 -0.084822

C 4.350754 1.148395 -0.321058

H 2.655499 2.442955 -0.441826

H 1.814129 -1.662061 0.493178

H 4.214709 -2.169709 0.389968

H 5.064384 1.929041 -0.547608

C 6.186839 -0.462030 -0.143447

N 7.308275 -0.706587 -0.190202

N 0.734814 0.810798 0.066308

H 0.562588 1.788265 -0.134826

C -0.348481 0.036899 0.347054

O -0.335665 -1.152421 0.613429

C -1.617773 0.763138 0.309637

F -1.646090 2.020396 -0.077826

C -2.970242 0.164852 0.576099

C -3.590970 -0.554738 -0.646193

F -3.821401 1.153671 0.908786

F -2.927428 -0.716318 1.584712

F -3.695567 0.299910 -1.658304

F -2.835784 -1.574716 -1.022988

F -4.800083 -1.006373 -0.343168

i3·LiBr

C -2.221987 -0.333389 -0.995440

C -1.630852 -0.954589 0.116022
C -2.435524 -1.613503 1.053882
C -3.800321 -1.659534 0.891890
C -4.391320 -1.043405 -0.217913
C -3.602504 -0.387630 -1.152800
H -1.614543 0.145959 -1.744824
H -1.971822 -2.088425 1.909301
H -4.417584 -2.169493 1.619333
H -4.058764 0.085815 -2.011919
C -5.812549 -1.089794 -0.387750
N -6.952298 -1.127838 -0.523003
N -0.261303 -0.953523 0.357807
H 0.030614 -1.442344 1.196534
C 0.714834 -0.356215 -0.382409
O 0.557923 0.276967 -1.411333
C 2.063665 -0.519681 0.151131
C 3.268560 0.052879 -0.529437
C 4.589416 -0.230992 0.223969
F 2.267390 -1.212349 1.251525
F 3.408721 -0.451511 -1.772708
F 3.164268 1.391048 -0.650996
F 4.804075 -1.536209 0.316984
F 5.595290 0.317711 -0.441894
F 4.551269 0.291193 1.442406
Br -2.185820 2.212527 0.501254

2a

C -3.020439 -0.148635 1.397915
C -2.200212 0.218981 0.328306
C -2.754583 0.493923 -0.921685
C -4.124804 0.394415 -1.090617
C -4.940535 0.024776 -0.024678
C -4.384137 -0.246163 1.226642
H -2.576887 -0.359257 2.363437
H -2.131878 0.782744 -1.751498
H -4.562921 0.606332 -2.056821
H -5.019331 -0.532278 2.053981
C -6.356967 -0.077704 -0.212683
N -7.492474 -0.160158 -0.363519
N -0.823115 0.292683 0.596413
H -0.563010 0.130576 1.562262
C 0.171044 0.497529 -0.287608
O 0.076366 0.687840 -1.476086
C 1.579938 0.456565 0.354347
C 2.468975 -0.664480 -0.234595
C 1.881150 -2.092468 -0.089942
F 1.519515 0.240867 1.691279
F 2.690221 -0.456729 -1.534198
F 3.645180 -0.664776 0.409851
F 1.710256 -2.407968 1.184767
F 0.712972 -2.179783 -0.714729
F 2.720652 -2.959466 -0.635564
Br 2.444186 2.161479 0.049200

TS′

C -2.271590 -0.237448 1.428947
C -1.465480 0.464542 0.515571
C -2.024280 0.885880 -0.703391
C -3.345643 0.615040 -0.988197
C -4.142206 -0.079051 -0.072610
C -3.589774 -0.506367 1.139990
H -1.840746 -0.567228 2.366706
H -1.414892 1.426796 -1.412485
H -3.771225 0.939377 -1.929221

H -4.201700 -1.047923 1.849726
C -5.509846 -0.352863 -0.373169
N -6.612556 -0.574607 -0.615060
N -0.148060 0.676299 0.817043
H 0.187130 0.301656 1.694667
C 0.723057 1.542912 0.116547
O 0.369354 2.668604 -0.295568
C 2.079028 1.089548 0.094252
F 2.628578 0.941845 1.658852
C 2.519539 -0.269508 -0.436702
C 1.716215 -1.543969 -0.084429
F 2.939202 1.990620 -0.397817
F 3.797915 -0.515698 -0.082035
F 2.515818 -0.209172 -1.796255
F 1.531565 -1.681030 1.221636
F 0.540788 -1.567463 -0.698728
F 2.410221 -2.599731 -0.510052

PC1^{2+}

C -2.514518 3.713042 -0.094743
C -3.288388 2.593392 -0.107334
C -2.664597 1.329238 -0.091991
C -1.234133 1.212782 -0.064959
C -0.468661 2.405699 -0.049891
C -1.099800 3.611063 -0.064253
C -1.234859 -1.212872 -0.063526
C -2.665387 -1.328536 -0.090632
C -3.289890 -2.592353 -0.104781
H -4.370833 -2.651067 -0.127065
C -2.516637 -3.712421 -0.090857
C -1.101866 -3.611209 -0.060088
C -0.470048 -2.406187 -0.046908
H -2.978706 4.689731 -0.106732
H -4.369297 2.652670 -0.129460
H 0.608604 2.355296 -0.027212
H -0.507060 4.515629 -0.052567
H -2.981364 -4.688863 -0.101976
H -0.509644 -4.516097 -0.047262
H 0.607243 -2.356315 -0.024012
S -3.678302 0.000642 -0.111999
N -0.625623 -0.000231 -0.056794
C 0.841095 -0.000631 -0.043901
C 1.483483 -0.001880 -1.253085
C 1.465846 0.000585 1.192825
C 2.886402 -0.001913 -1.231234
H 0.941721 -0.002717 -2.189231
C 2.857827 0.000659 1.183046
H 0.880349 0.001548 2.099934
C 3.566325 -0.000633 -0.023763
H 4.645777 -0.000514 0.022438
O 3.483822 -0.003160 -2.433708
O 3.607751 0.002026 2.296800
C 2.938839 0.004074 3.551959
H 2.321339 -0.889465 3.664340
H 3.720217 0.004973 4.307026
H 2.321867 0.898323 3.661612
C 4.905544 -0.002711 -2.472425
H 5.307125 0.891156 -1.991160
H 5.307745 -0.895446 -1.989575
H 5.175058 -0.003553 -3.525040

2aa-i1

C -1.189909 -1.033440 -0.002095

C -0.569592 0.245538 -0.001783

C -1.399704 1.401347 -0.000897

C -2.759356 1.299163 0.000066

C -3.396941 0.015482 0.000092

C -2.553529 -1.141355 -0.001179

H -0.577862 -1.922086 -0.003041

H -0.933798 2.381377 -0.000792

H -3.367608 2.195295 0.000886

H -3.008515 -2.124511 -0.001458

C -4.788390 -0.101486 0.001321

N -5.949562 -0.198984 0.002359

N 0.807489 0.443550 -0.002349

H 1.094234 1.414910 -0.003436

C 1.793695 -0.464198 -0.001014

O 1.690517 -1.694080 0.000457

C 3.202310 0.119765 0.000454

F 3.895198 -0.299595 -1.067772

F 3.242796 1.459593 -0.006720

F 3.887829 -0.287689 1.078306

2aa-i1·LiBr

C -2.108550 -0.837560 -0.003915

C -2.142771 0.574243 -0.069830

C -3.404906 1.216191 -0.102261

C -4.568341 0.492902 -0.064887

C -4.527139 -0.910152 0.003521

C -3.283468 -1.554228 0.030889

H -1.158774 -1.346044 0.020199

H -3.440511 2.297922 -0.154244

H -5.523310 1.002139 -0.088982

H -3.245552 -2.635337 0.079675

C -5.731303 -1.665264 0.042224

N -6.707916 -2.275947 0.073216

N -1.031644 1.358529 -0.118555

H -1.215442 2.343899 -0.262111

C 0.312524 0.983564 -0.184617

O 0.732566 -0.190280 0.148386

C 1.251106 2.116686 0.016331

F 2.503447 1.735990 -0.327346

F 0.945251 3.214072 -0.693068

Li 2.478651 -0.607047 -0.017055

Br 4.495643 -1.768415 -0.028223

F 1.366148 2.549821 1.305968

2aa-TS

N -0.742458 1.702603 0.045202

C 0.536580 1.454068 0.522174

O 0.876060 0.414819 1.175313

C 1.581499 2.293481 0.040921

F 2.539670 1.304840 -0.906510

F 1.302192 3.290166 -0.768435

Br 3.342306 -2.369466 -0.146597

Li 2.326741 -0.272049 0.130718

H -0.924185 2.647843 -0.266411

C -1.830637 0.863828 0.046219

C -3.076532 1.418988 -0.304978

C -1.759176 -0.506096 0.355393

C -4.208511 0.641076 -0.347372

H -3.134763 2.474260 -0.543411

C -2.900185 -1.280517 0.312617

H -0.815113 -0.949150 0.627114

C -4.132276 -0.721767 -0.035316

H -5.159470 1.080259 -0.619427

H -2.838995 -2.335122 0.548904

C -5.304509 -1.533980 -0.073911

N -6.250135 -2.188487 -0.105346

F 2.537444 2.642495 0.887832

2aa-i2

N 0.108288 -1.931441 -0.077264

C 1.286769 -1.441160 -0.532216

O 1.438375 -0.365150 -1.111521

C 2.420053 -2.310619 -0.323782

F 2.504939 -0.268065 1.528269

F 2.406133 -3.302249 0.528042

Br 1.448877 3.131092 0.032750

Li 2.139534 0.863597 0.291016

H 0.112873 -2.873151 0.296366

C -1.137214 -1.294525 -0.082932

C -2.248664 -2.092505 0.209385

C -1.302749 0.071459 -0.326977

C -3.511260 -1.543079 0.252917

H -2.110127 -3.149283 0.402587

C -2.572245 0.619166 -0.281542

H -0.459418 0.710233 -0.538270

C -3.677552 -0.179402 0.003868

H -4.368830 -2.162862 0.477874

H -2.704565 1.677785 -0.463056

C -4.986101 0.399678 0.048879

N -6.036645 0.863076 0.085211

F 3.618488 -1.913691 -0.630074

2aa-i3

C -1.079157 1.404943 0.014443

C -0.263142 0.267422 -0.024237

C -0.844450 -1.003590 -0.048662

C -2.222664 -1.122339 -0.033991

C -3.030176 0.011997 0.004834

C -2.450555 1.282668 0.028689

H -0.622324 2.386828 0.032256

H -0.229251 -1.886837 -0.079281

H -2.675755 -2.104687 -0.053914

H -3.076336 2.164592 0.058027

C -4.454929 -0.123895 0.018942

N -5.598657 -0.232047 0.029915

N 1.114224 0.486398 -0.044885

H 1.398392 1.458387 -0.069595

C 2.110306 -0.446402 -0.017353

O 1.971655 -1.655780 0.048943

C 3.443911 0.124026 -0.134430

F 3.683405 1.403103 0.018116

F 4.490839 -0.616601 0.090366

2a′

C 3.209573 0.285916 1.351054

C 2.316536 -0.116888 0.346371

C 2.821707 -0.640521 -0.850290

C 4.188721 -0.752182 -1.025659

C 5.069379 -0.349520 -0.023352

C 4.569525 0.172952 1.172643

H 2.817332 0.691750 2.275505

H 2.149872 -0.954524 -1.630748

H 4.577664 -1.156840 -1.950895

H 5.249457 0.487107 1.953393

C 6.480834 -0.471443 -0.217985
N 7.615370 -0.569616 -0.373688
N 0.964169 0.028854 0.618099
H 0.746287 0.437837 1.516868
C -0.105100 -0.225910 -0.210154
O -0.008622 -0.665786 -1.346920
C -1.383432 0.110835 0.386320
C -2.688895 -0.384638 -0.148921
C -2.964546 -1.888664 0.106560
F -1.419258 0.278306 1.721948
F -3.707488 0.262412 0.462439
F -2.801033 -0.199691 -1.472859
F -4.174303 -2.222780 -0.337899
F -2.071869 -2.653232 -0.508454
F -2.915137 -2.149517 1.411084
Br -1.451784 2.723634 -0.283014

PC2$^{\cdot+}$

C -1.100406 4.107616 -0.207494
C 0.055701 3.700819 0.438107
C 0.417667 2.354483 0.495660
C -0.403731 1.381002 -0.073491
C -1.549857 1.789893 -0.770176
C -1.883720 3.119434 -0.831729
C 1.245500 -0.401620 0.005037
C 2.261241 0.421885 0.530223
C 3.578191 0.006774 0.511197
H 4.332145 0.665283 0.924761
C 3.950379 -1.229977 -0.023542
C 2.950161 -2.043163 -0.540060
C 1.617871 -1.646653 -0.526859
H 0.714630 4.423471 0.900654
H -2.159587 1.052696 -1.274386
H -2.768693 3.403158 -1.386314
H 3.189324 -3.009866 -0.961681
S 1.891210 1.932394 1.320988
N -0.083191 0.010666 0.011814
C -1.123357 -0.930484 0.091468
C -0.996362 -2.203394 -0.470263
C -2.310496 -0.610605 0.768931
C -2.063599 -3.102966 -0.424968
C -3.340880 -1.513149 0.821660
H -2.399361 0.345283 1.266488
C -3.252133 -2.775944 0.203421
H -4.237014 -1.240628 1.363959
H -1.928884 -4.074324 -0.881702
C -1.527214 5.567956 -0.288004
C -2.919687 5.711574 0.342707
H -2.902832 5.413259 1.393325
H -3.238862 6.754599 0.287047
H -3.661471 5.102626 -0.176406
C -0.559930 6.493956 0.446047
H -0.919686 7.521085 0.365503
H -0.490395 6.244125 1.506997
H 0.442398 6.454742 0.014114
C -1.591410 5.992511 -1.761993
H -0.614819 5.886903 -2.239586
H -2.315009 5.398401 -2.322174
H -1.893325 7.040200 -1.825800
C 5.419093 -1.638065 -0.013376
C 5.910317 -1.688855 1.440388
H 5.339779 -2.419590 2.017789
H 6.962088 -1.982354 1.457868

H 5.822115 -0.717628 1.929840
C 6.234940 -0.595465 -0.790515
H 5.895684 -0.529615 -1.826545
H 6.160055 0.395093 -0.339249
H 7.287389 -0.886882 -0.791366
C 5.639179 -3.007097 -0.653387
H 5.319574 -3.019589 -1.697662
H 6.703693 -3.245065 -0.624779
H 5.104873 -3.793987 -0.116848
C -4.436298 -3.731381 0.271831
C -4.752608 -4.035793 1.742972
H -5.013587 -3.132126 2.295666
H -5.600860 -4.721576 1.797534
H -3.898390 -4.506602 2.234506
C -5.651919 -3.057336 -0.380611
H -5.451946 -2.827063 -1.429433
H -6.510024 -3.731421 -0.334462
H -5.921132 -2.131530 0.130266
C -4.159035 -5.047294 -0.451551
H -3.943235 -4.887030 -1.510151
H -3.320059 -5.584532 -0.003980
H -5.041692 -5.685094 -0.381269
S 0.458405 -2.706210 -1.274425

15 X-Ray Crystallography

Crystals were grown for x-ray crystallography using the solvent layering method. In a 2-dram vial, 100 mg **2a** was dissolved in ~1 mL $CHCl_3$ to make a concentrated solution. Pentane (5 mL) was then layered gently on top of the $CHCl_3$ solution, and the vial cap was closed. The vial was allowed to sit undisturbed at room temperature for 24 h, after which thick, block-like crystals were present in the $CHCl_3$ layer.

The following is a crystal structure of **2a** with 50% thermal ellipsoids. Hydrogen atoms have been omitted for clarity.

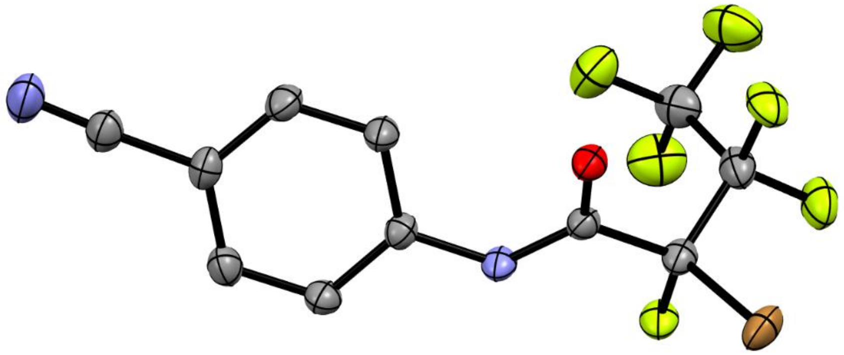

**Compound**	**2a**
**Formula**	$C_{11}H_5BrF_6N_2O$
**Fw (g·mol⁻¹)**	375.065
**Cell Setting**	Monoclinic
**Space Group**	$P2_1/c$
***a* (Å)**	10.70537(15)
***b* (Å)**	12.55247(14)
**c (Å)**	9.91368(13)
**α (°)**	90.0
**β (°)**	102.9397(13)
**γ (°)**	90.0
***V* ($Å^{-3}$)**	1298.36(3)
Z	4
**ρ_{calc} ($g{\cdot}cm^{-1}$)**	1.919
**Radiation, λ (Å)**	1.54184
**μ (mm^{-1})**	1.263
**$R_{(int)}$**	0.029
**Parameters**	190
**R_1**	2.20
**ωR_2**	5.78
**GooF**	0.957
***T* (K)**	150.0
**CCDC Deposition No.**	2545637

Table S6. Selected x-ray collection data and refinement parameters for compound **2a**.

16 References

[1] Fulmer, G. R.; Miller, A. J. M.; Sherden, N. H.; Gottlieb, H. E.; Nudelman, A.; Stoltz, B. M.; Bercaw, J. E.; Goldberg, K. I. NMR Chemical Shifts of Trace Impurities: Common Laboratory Solvents, Organics, and Gases in Deuterated Solvents Relevant to the Organometallic Chemist. *Organometallics* **2010**, *29* (9), 2176–2179.

[2] Bard, A. J.; Faulkner, L. R. *Electrochemical Methods: Fundamentals and Applications (2nd Edition)*. John Wiley & Sons, Hoboken, NJ, 2000.

[3] Cosier, J.; Glazer, A. M. A nitrogen-gas-stream cryostat for general X-ray diffraction studies. *J. Appl. Crystallogr.* **1986**, *19* (2), 105–107.

[4] Rigaku Oxford Diffraction. 2021.

[5] Palatinus, L.; Chapuis, G. SUPERFLIP – a computer program for the solution of crystal structures by charge flipping in arbitrary dimensions. *J. Appl. Crystallogr.* **2007**, *40* (4), 786–790.

[6] Betteridge, P. W.; Carruthers, J. R.; Cooper, R. I.; Prout, K.; Watkin, D. J. CRYSTALS version 12: software for guided crystal structure analysis. *J. Appl. Crystallogr.* **2003**, *36* (6), 1487.

[7] Cooper, R. I.; Thompson, A. L.; Watkin, D. J. CRYSTALS enhancements: dealing with hydrogen atoms in refinement. *J. Appl. Crystallogr.* **2010**, *43* (5), 1100–1107.

[8] Speck, F.; Rombach, D.; Wagenknecht, H.-A. *N*-Arylphenothiazines as strong donors for photoredox catalysis – pushing the frontiers of nucleophilic addition of alcohols to alkenes. *Beilstein J. Org. Chem.* **2019**, *15*, 52–59.

[9] Ando, H.; Takamura, H.; Kadota, I.; Tanaka, K. Strongly reducing helical phenothiazines as recyclable organophotoredox catalysts. *Chem. Commun.* **2024**, *60* (36), 4765–4768.

[10] Chen, S.; Li, Z.; Hu, K.; Feng, W.; Mao, G.; Xiao, F.; Deng, G.-J. Three-component selective synthesis of phenothiazines and bis-phenothiazines under metal-free conditions. *Org. Biomol. Chem.* **2023**, *21* (9), 1920–1926.

[11] Kees, K. L.; Smith, T. M.; McCaleb, M. L.; Prozialeck, D. H.; Cheeseman, R. S.; Christos, T. E.; Patt, W. C.; Steiner, K. E. Perfluorocarbon-based antidiabetic agents. *J. Med. Chem.* **1992**, *35* (5), 944–953.

[12] Childress, E. S.; Kharel, Y.; Brown, A. M.; Bevan, D. R.; Lynch, K. R.; Santos, W. L. Transforming Sphingosine Kinase 1 Inhibitors into Dual and Sphingosine Kinase 2 Selective Inhibitors: Design, Synthesis, and in Vivo Activity. *J. Med. Chem.* **2017**, *60* (9), 3933–3957.

[13] Bunnell, A.; Lalloo, N.; Brigham, C.; Sanford, M. S. Palladium-Catalyzed Decarbonylative Coupling of (Hetero)Aryl Boronate Esters with Difluorobenzyl Glutarimides. *Org. Lett.* **2023**, *25* (41), 7584–7588.

[14] Sugihara, N.; Nishimoto, Y.; Osakada, Y.; Fujitsuka, M.; Abe, M.; Yasuda, M. Sequential C−F Bond Transformation of the Difluoromethylene Unit in Perfluoroalkyl Groups: A Combination of Fine-Tuned Phenothiazine Photoredox Catalyst and Lewis Acid. *Angew. Chem. Int. Ed.* **2024**, *63* (14), e202401117.

[15] Blackmond, D. G. Reaction Progress Kinetic Analysis: A Powerful Methodology for Mechanistic Studies of Complex Catalytic Reactions. *Angew. Chem. Int. Ed.* **2005**, *44* (28), 4302–4320.

[16] Burés, J. Variable Time Normalization Analysis: General Graphical Elucidation of Reaction Orders from Concentration Profiles. *Angew. Chem. Int. Ed.* **2016**, *55* (52), 16084–16087.

[17] Pfund, B.; Gejsnæs-Schaad, D.; Lazarevski, B.; Wenger, O. S. Picosecond reactions of excited radical ion super-reductants. *Nat. Commun.* **2024**, *15* (1), 4738.

[18] Targos, K.; Williams, O. P.; Wickens, Z. K. Unveiling Potent Photooxidation Behavior of Catalytic Photoreductants. *J. Am. Chem. Soc.* **2021**, *143* (11), 4125–4132.

[19] Christensen, J. A.; Phelan, B. T.; Chaudhuri, S.; Acharya, A.; Batista, V. S.; Wasielewski, M. R. Phenothiazine Radical Cation Excited States as Super-oxidants for Energy-Demanding Reactions. *J. Am. Chem. Soc.* **2018**, *140* (15), 5290–5299.

[20] Romero, N. A.; Nicewicz, D. A. Organic Photoredox Catalysis. *Chem. Rev.* **2016**, *116* (17), 10075–10166.

[21] Richards, A. (2015). University of Oxford Advanced Research Computing. Zenodo.10.5281/zenodo.22558

[22] Neese, F. The ORCA program system. *WIREs Comput. Mol. Sci.* **2012**, *2* (1), 73–78.

[23] Neese, F. Software update: The ORCA program system—Version 5.0. *WIREs Comput. Mol. Sci.* **2022**, *12* (5), e1606.

[24] de Souza, B. GOAT: A Global Optimization Algorithm for Molecules and Atomic Clusters. *Angew. Chem. Int. Ed.* **2025**, *64* (18), e202500393.

[25] Bannwarth, C.; Ehlert, S.; Grimme, S. GFN2-xTB—An Accurate and Broadly Parametrized Self-Consistent Tight-Binding Quantum Chemical Method with Multipole Electrostatics and Density-Dependent Dispersion Contributions. *J. Chem. Theory Comput.* **2019**, *15* (3), 1652–1671.

[26] Ehlert, S.; Stahn, M.; Spicher, S.; Grimme, S. Robust and Efficient Implicit Solvation Model for Fast Semiempirical Methods. *J. Chem. Theory Comput.* **2021**, *17* (7), 4250–4261.

[27] Frisch, M. J.; Trucks, G. W.; Schlegel, H. B.; Scuseria, G. E.; Robb, M. A.; Cheeseman, J. R.; Scalmani, G.; Barone, V.; Petersson, G. A.; Nakatsuji, H.; Li, X.; Caricato, M.; Marenich, A. V.; Bloino, J.; Janesko, B. G.; Gomperts, R.; Mennucci, B.; Hratchian, H. P.; Ortiz, J. V.; Izmaylov, A. F.; Sonnenberg, J. L.; Williams-Young, D.; Ding, F.; Lipparini, F.; Egidi, F.; Goings, J.; Peng, B.; Petrone, A.; Henderson, T.; Ranasinghe, D.; Zakrzewski, V. G.; Gao, J.; Rega, N.; Zheng, G.; Liang, W.; Hada, M.; Ehara, M.; Toyota, K.; Fukuda, R.; Hasegawa, J.; Ishida, M.; Nakajima, T.; Honda, Y.; Kitao, O.; Nakai, H.; Vreven, T.; Throssell, K.; Montgomery, J. A., Jr.; Peralta, J. E.; Ogliaro, F.; Bearpark, M. J.; Heyd, J. J.; Brothers, E. N.; Kudin, K. N.; Staroverov, V. N.; Keith, T. A.; Kobayashi, R.; Normand, J.; Raghavachari, K.; Rendell, A. P.; Burant, J. C.; Iyengar, S. S.; Tomasi, J.; Cossi, M.; Millam, J. M.; Klene, M.; Adamo, C.; Cammi, R.; Ochterski, J. W.; Martin, R. L.; Morokuma, K.; Farkas, O.; Foresman, J. B.; Fox, D. J. Gaussian 16, Revision C.01; Gaussian, Inc., Wallingford, CT, 2016.

[28] Zhao, Y.; Truhlar, D. G. A new local density functional for main-group thermochemistry, transition metal bonding, thermochemical kinetics, and noncovalent interactions. *J. Chem. Phys.* **2006**, *125* (19), 194101.

[29] Grimme, S.; Antony, J.; Ehrlich, S.; Krieg, H. A consistent and accurate *ab initio* parametrization of density functional dispersion correction (DFT-D) for the 94 elements H-Pu. *J. Chem. Phys.* **2010**, *132* (15), 154104.

[30] Weigend, F.; Ahlrichs, R. Balanced basis sets of split valence, triple zeta valence and quadruple zeta valence quality for H to Rn: Design and assessment of accuracy. *Phys. Chem. Chem. Phys.* **2005**, *7* (18), 3297–3305.

[31] Marenich, A. V.; Cramer, C. J.; Truhlar, D. G. Universal Solvation Model Based on Solute Electron Density and on a Continuum Model of the Solvent Defined by the Bulk Dielectric Constant and Atomic Surface Tensions. *J. Phys. Chem. B* **2009**, *113* (18), 6378–6396.

[32] Cancès, E.; Mennucci, B.; Tomasi, J. A new integral equation formalism for the polarizable continuum model: Theoretical background and applications to isotropic and anisotropic dielectrics. *J. Chem. Phys.* **1997**, *107* (8), 3032–3041.

[33] Chai, J.-D.; Head-Gordon, M. Long-range corrected hybrid density functionals with damped atom–atom dispersion corrections. *Phys. Chem. Chem. Phys.* **2008**, *10* (44), 6615–6620.

[34] Luchini, G.; Alegre-Requena, J.; Funes-Ardoiz, I.; Paton, R. GoodVibes: Automated Thermochemistry for Heterogeneous Computational Chemistry Data. *F1000Research* **2020**, *9*, 291.
[35] Grimme, S. Supramolecular Binding Thermodynamics by Dispersion-Corrected Density Functional Theory. *Chem. Eur. J.* **2012**, *18* (32), 9955–9964.
[36] Glendening, E. D.; Landis, C. R.; Weinhold, F. NBO 7.0: New vistas in localized and delocalized chemical bonding theory. *J. Comput. Chem.* **2019**, *40*, 2234–2241.
[37] Becke, A. D. Density-functional thermochemistry. III. The role of exact exchange. *J. Chem. Phys.* **1993**, *98* (7), 5648–5652.
[38] Stephens, P. J.; Devlin, F. J.; Chabalowski, C. F.; Frisch, M. J. Ab Initio Calculation of Vibrational Absorption and Circular Dichroism Spectra Using Density Functional Force Fields. *J. Phys. Chem.* **1994**, *98* (45), 11623–11627.
[39] Popescu, M. V.; Paton, R. S. Dynamic vertical triplet energies: Understanding and predicting triplet energy transfer. *Chem* **2024**, *10* (11), 3428–3443.
[40] Hehre, W. J.; Ditchfield, R.; Pople, J. A. Self—Consistent Molecular Orbital Methods. XII. Further Extensions of Gaussian—Type Basis Sets for Use in Molecular Orbital Studies of Organic Molecules. *J. Chem. Phys.* **1972**, *56* (5), 2257–2261.
[41] Teynor, M. S.; Wohlgemuth, N.; Carlson, L.; Huang, J.; Pugh, S. L.; Grant, B. O.; Hamilton, R. S.; Carlsen, R.; Ess, D. H. Milo, Revision 1.0.3; Brigham Young University, Provo UT, 2021.
[42] Easton, R. E.; Giesen, D. J.; Welch, A.; Cramer, C. J.; Truhlar, D. G. The MIDI! basis set for quantum mechanical calculations of molecular geometries and partial charges. *Theoret. Chim. Acta* **1996**, *93* (5), 281–301.
[43] Hunter, J. D. Matplotlib: A 2D Graphics Environment. *Comput. Sci. Eng.* **2007**, *9* (3), 90–95.
[44] Pedregosa, F.; Varoquaux, G.; Gramfort, A.; Michel, V.; Thirion, B.; Grisel, O.; Blondel, M.; Prettenhofer, P.; Weiss, R.; Dubourg, V.; Vanderplas, J.; Passos, A.; Cournapeau, D.; Brucher, M.; Perrot, M.; Duchesnay, É. Scikit-learn: Machine Learning in Python. *J. Mach. Learn. Res.* **2011**, *12* (85), 2825–2830.
[45] Mayer, L.; May, L.; Müller, T. J. J. The interplay of conformations and electronic properties in *N*-aryl phenothiazines. *Org. Chem. Front.* **2020**, *7* (10), 1206–1217.

17 NMR Spectra

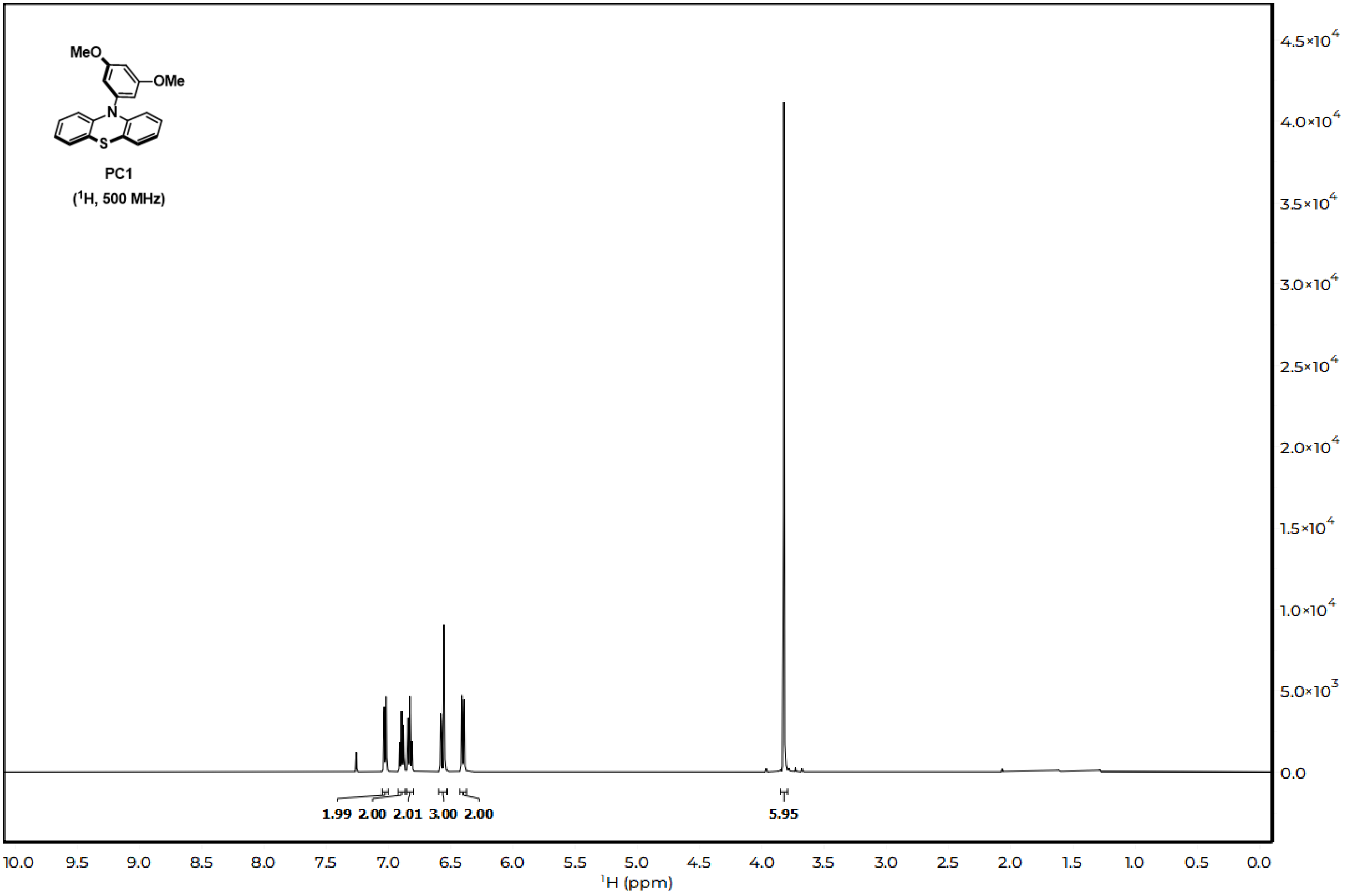

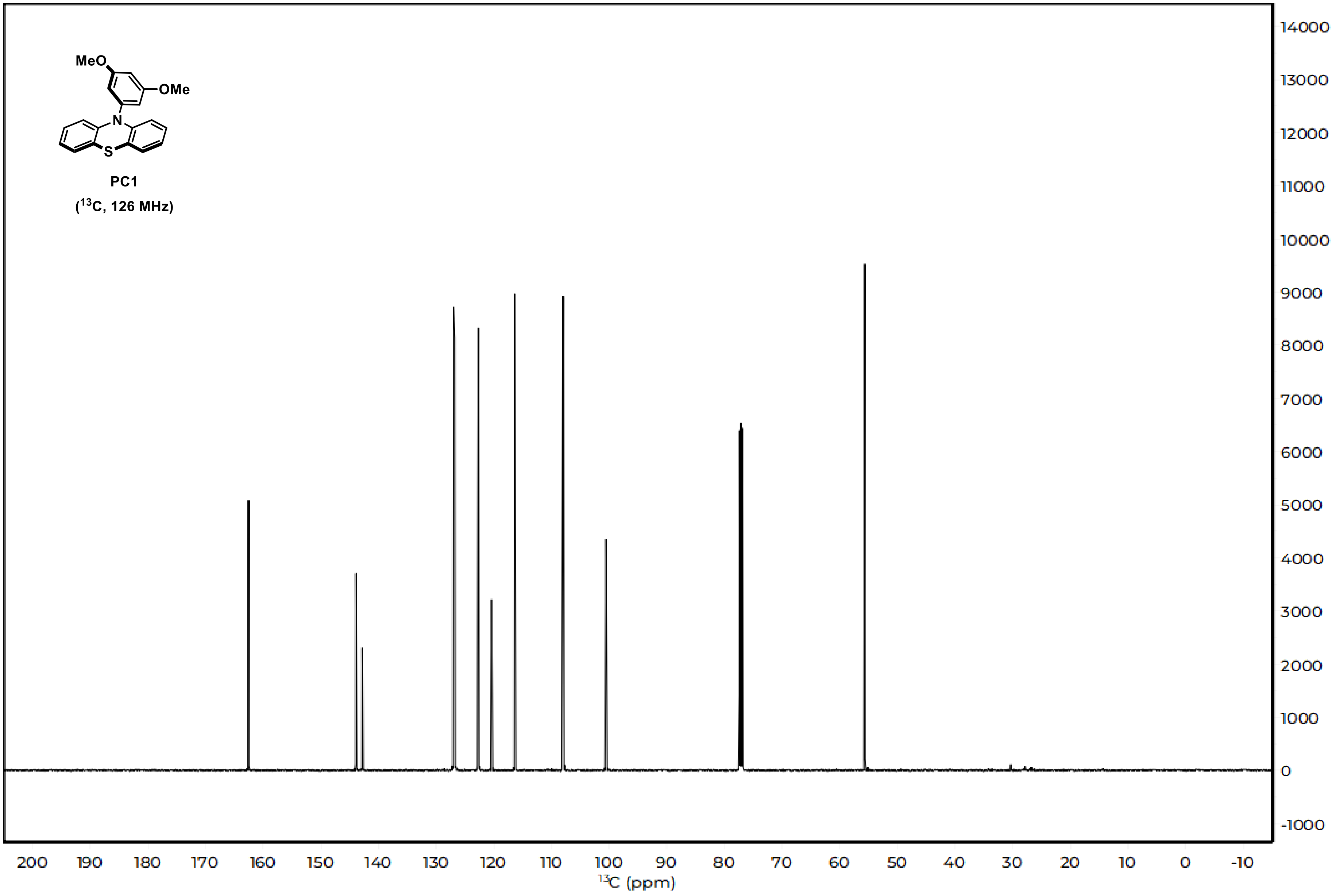

MeO
OMe
N
S
PC1
(13C, 126 MHz)
14000
13000
12000
11000
10000
9000
8000
7000
6000
5000
4000
3000
2000
1000
0
-1000
200
190
180
170
160
150
140
130
120
110
100
90
80
70
60
50
40
30
20
10
0
-10
13C (ppm)

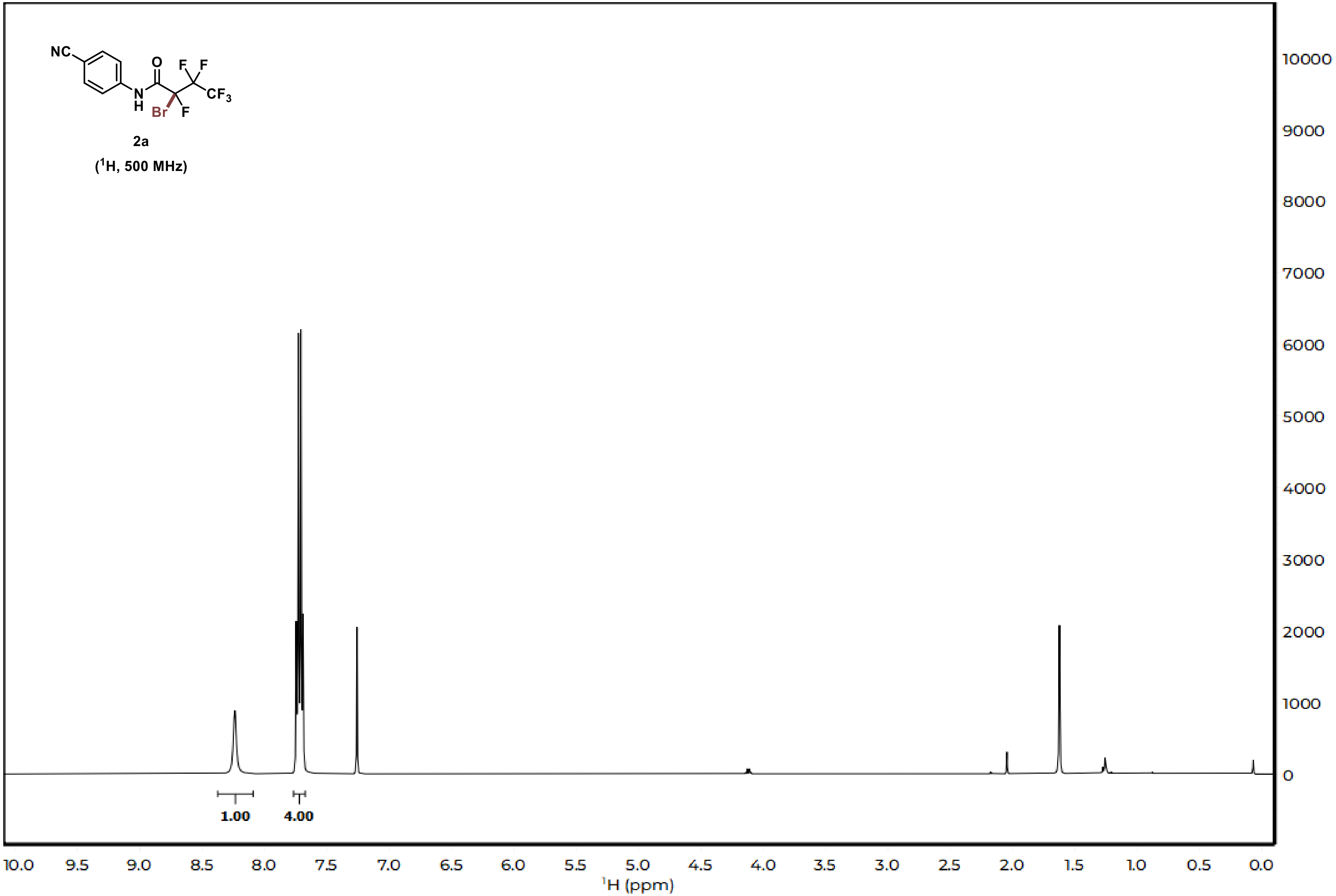

NC
O
F F
CF3
N
H
Br
F
2a
(1H, 500 MHz)
10000
9000
8000
7000
6000
5000
4000
3000
2000
1000
0
1.00
4.00
10.0 9.5 9.0 8.5 8.0 7.5 7.0 6.5 6.0 5.5 5.0 4.5 4.0 3.5 3.0 2.5 2.0 1.5 1.0 0.5 0.0
1H (ppm)

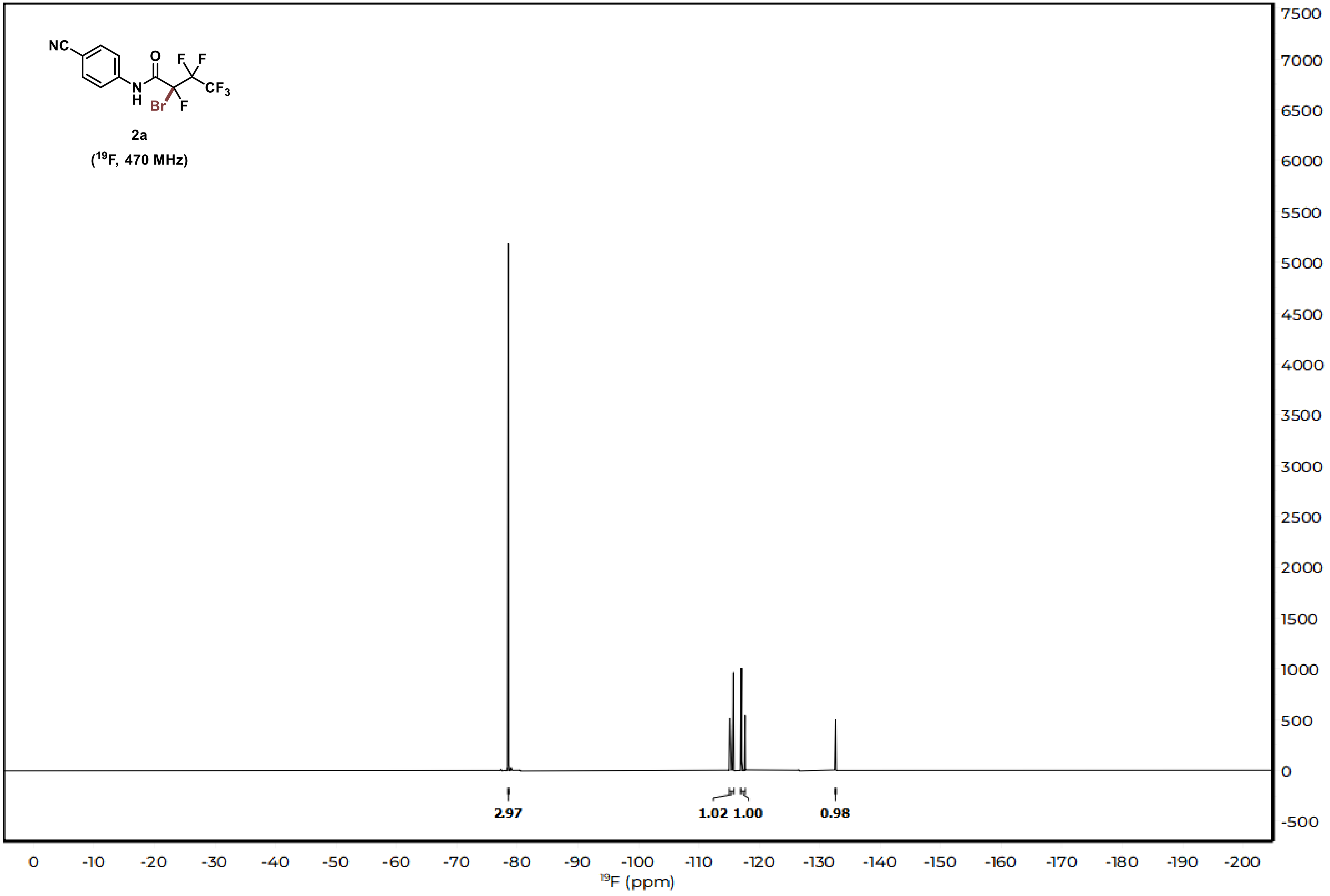

NC
O
F F
N
H
Br
F
CF_3
2a
(^{19}F, 470 MHz)
2.97
1.02 1.00
0.98
7500
7000
6500
6000
5500
5000
4500
4000
3500
3000
2500
2000
1500
1000
500
0
-500
0
-10
-20
-30
-40
-50
-60
-70
-80
-90
-100
-110
-120
-130
-140
-150
-160
-170
-180
-190
-200
^{19}F (ppm)

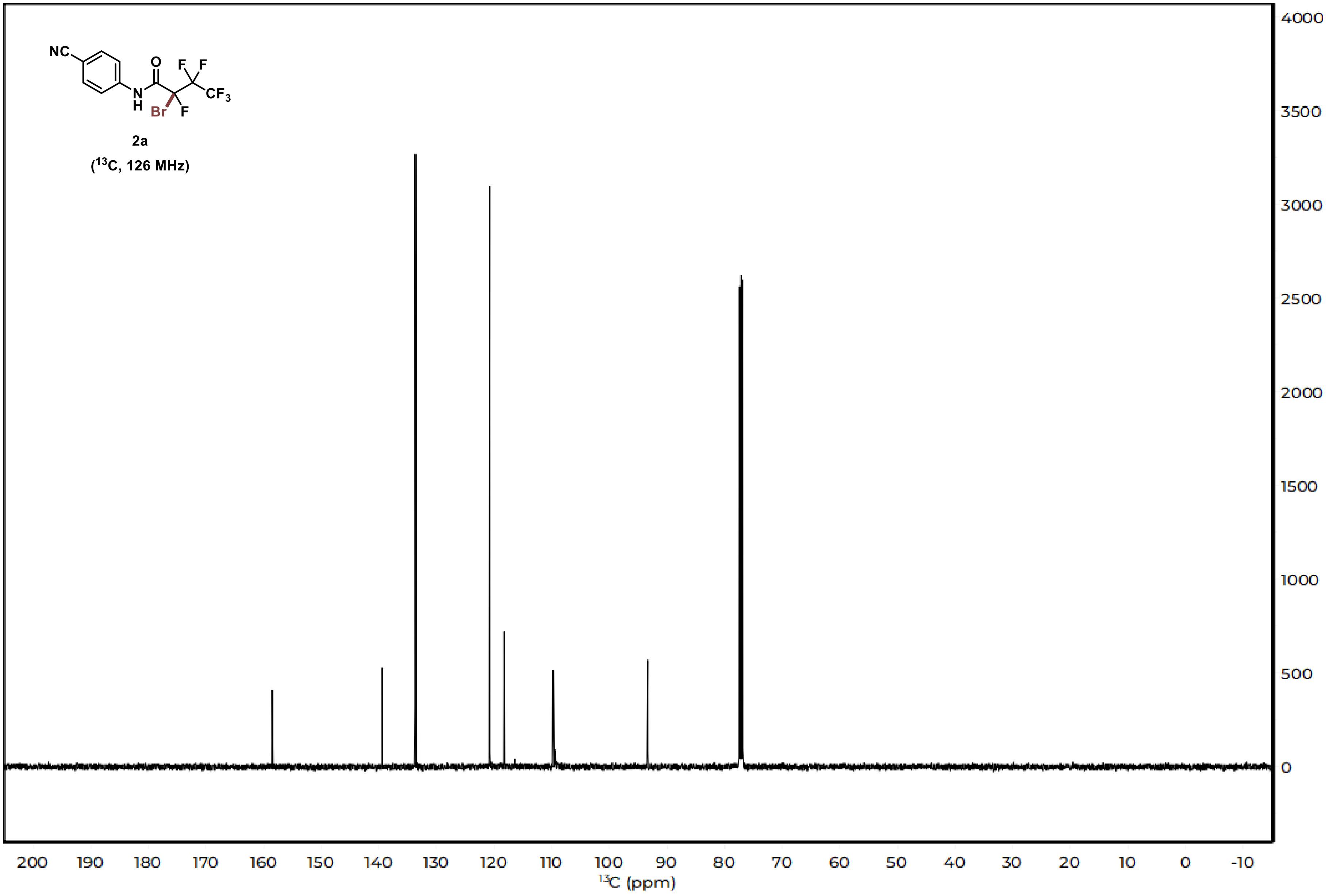
NC
O
F F
N
H
CF3
Br F
2a
(13C, 126 MHz)
4000
3500
3000
2500
2000
1500
1000
500
0
200 190 180 170 160 150 140 130 120 110 100 90 80 70 60 50 40 30 20 10 0 -10
13C (ppm)

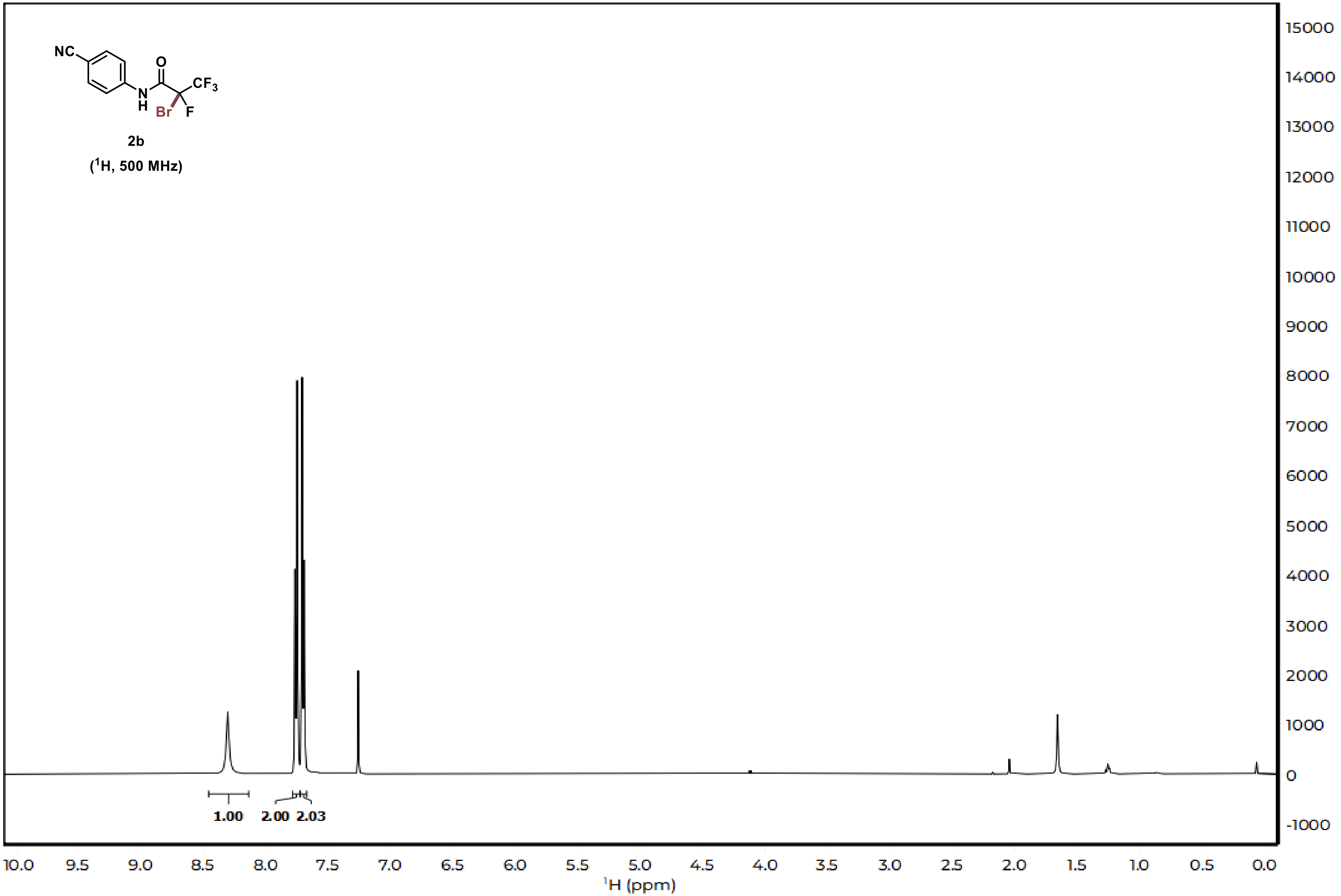
NC
O
CF3
N
H
Br
F
2b
(1H, 500 MHz)
15000
14000
13000
12000
11000
10000
9000
8000
7000
6000
5000
4000
3000
2000
1000
0
-1000
1.00
2.00
2.03
10.0
9.5
9.0
8.5
8.0
7.5
7.0
6.5
6.0
5.5
5.0
4.5
4.0
3.5
3.0
2.5
2.0
1.5
1.0
0.5
0.0
1H (ppm)

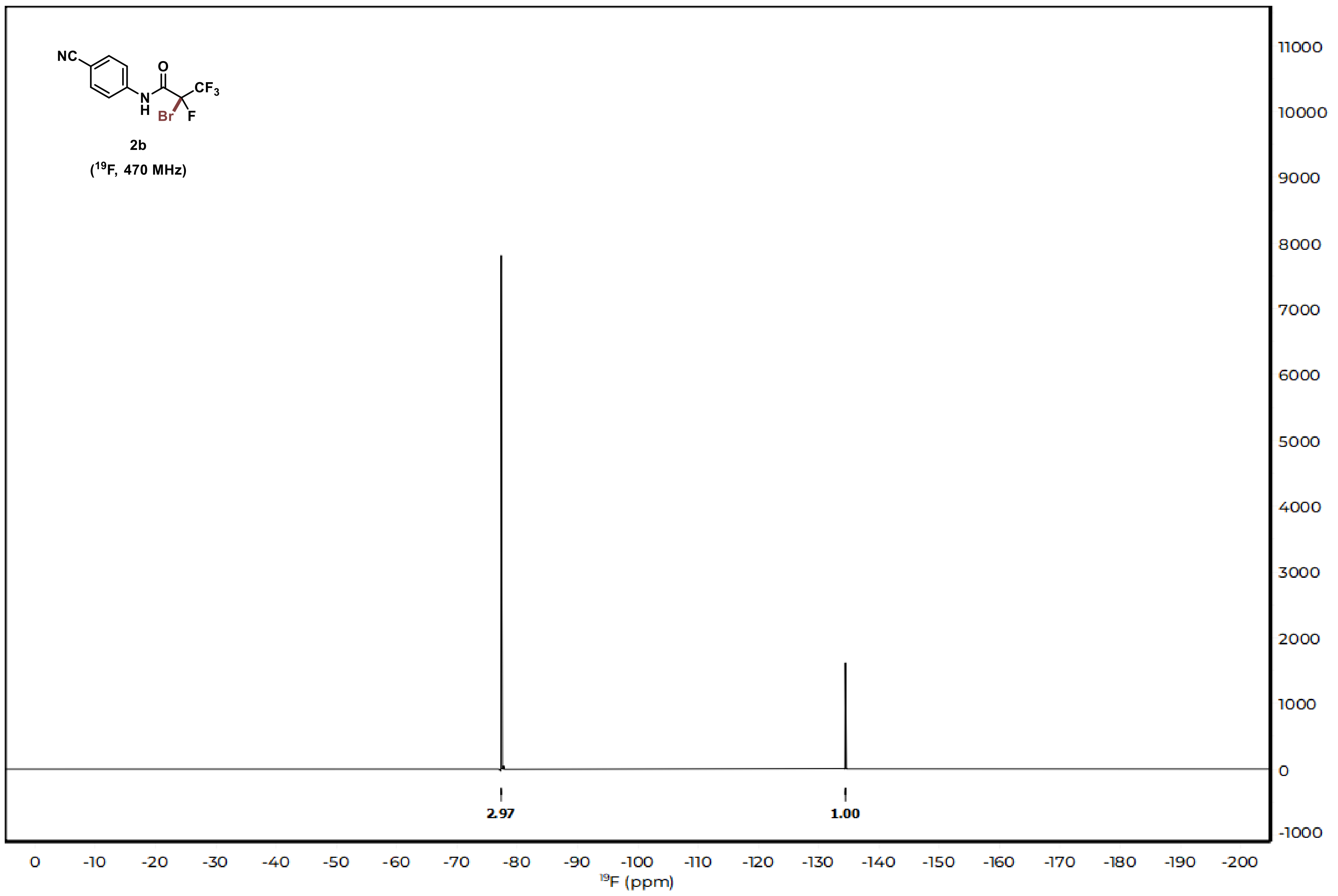

NC
O
CF3
N
H
Br
F
2b
(19F, 470 MHz)
11000
10000
9000
8000
7000
6000
5000
4000
3000
2000
1000
0
-1000
2.97
1.00
0
-10
-20
-30
-40
-50
-60
-70
-80
-90
-100
-110
-120
-130
-140
-150
-160
-170
-180
-190
-200
19F (ppm)

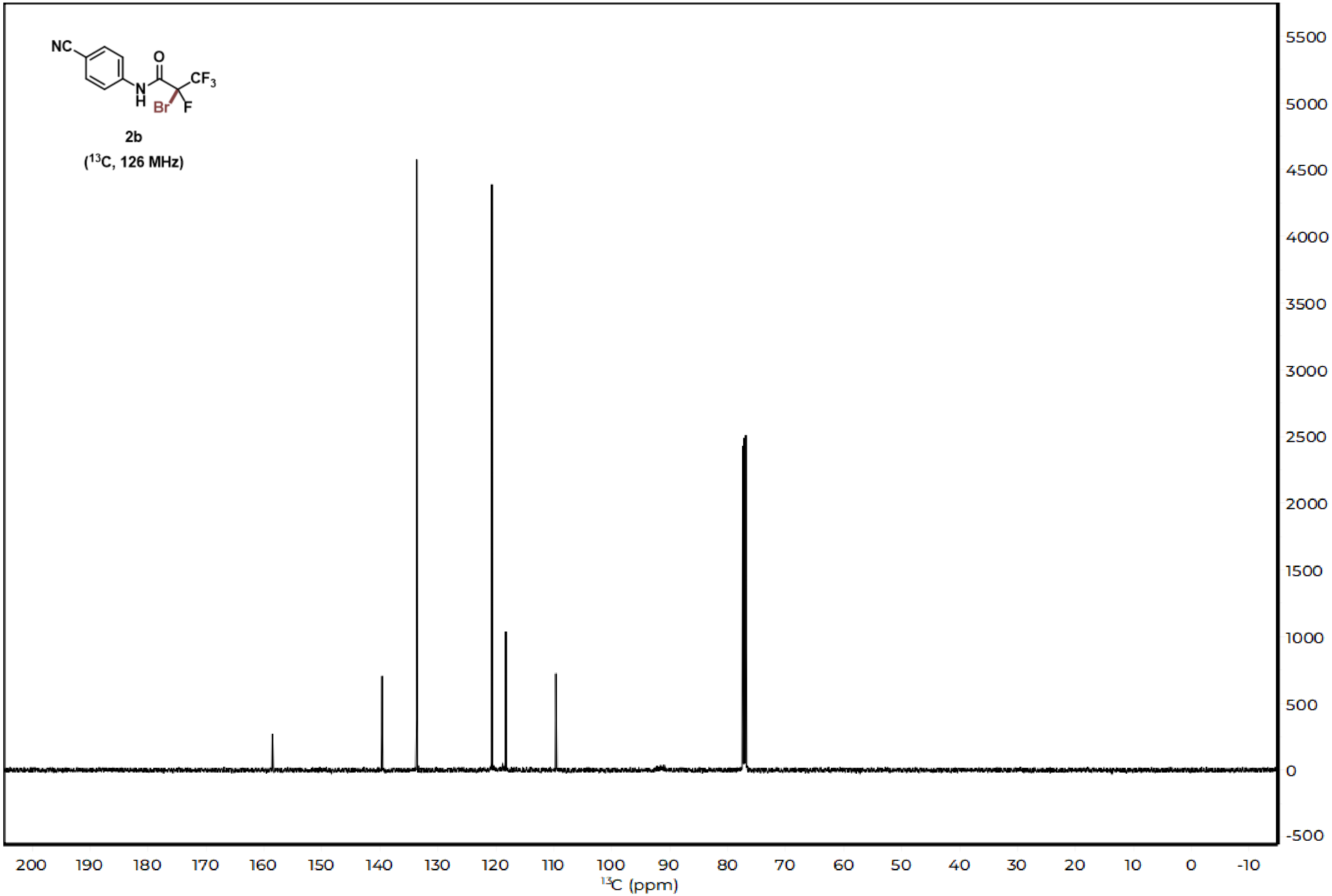
NC
O
N
H
CF3
Br
F
2b
(13C, 126 MHz)
5500
5000
4500
4000
3500
3000
2500
2000
1500
1000
500
0
-500
200 190 180 170 160 150 140 130 120 110 100 90 80 70 60 50 40 30 20 10 0 -10
13C (ppm)

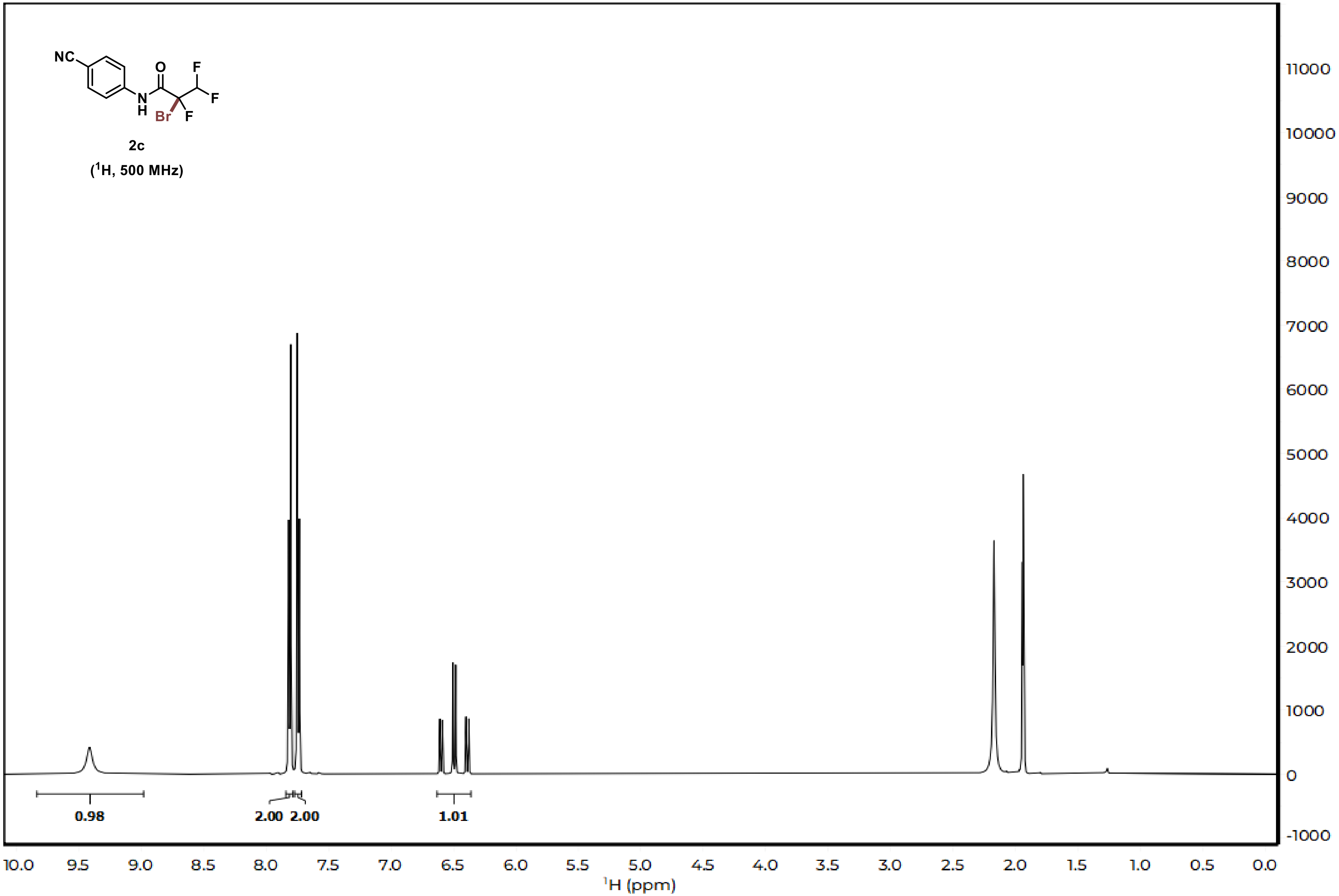
NC
O
F
F
N
H
Br
F
2c
(1H, 500 MHz)
11000
10000
9000
8000
7000
6000
5000
4000
3000
2000
1000
0
-1000
0.98
2.00 2.00
1.01
10.0 9.5 9.0 8.5 8.0 7.5 7.0 6.5 6.0 5.5 5.0 4.5 4.0 3.5 3.0 2.5 2.0 1.5 1.0 0.5 0.0
1H (ppm)

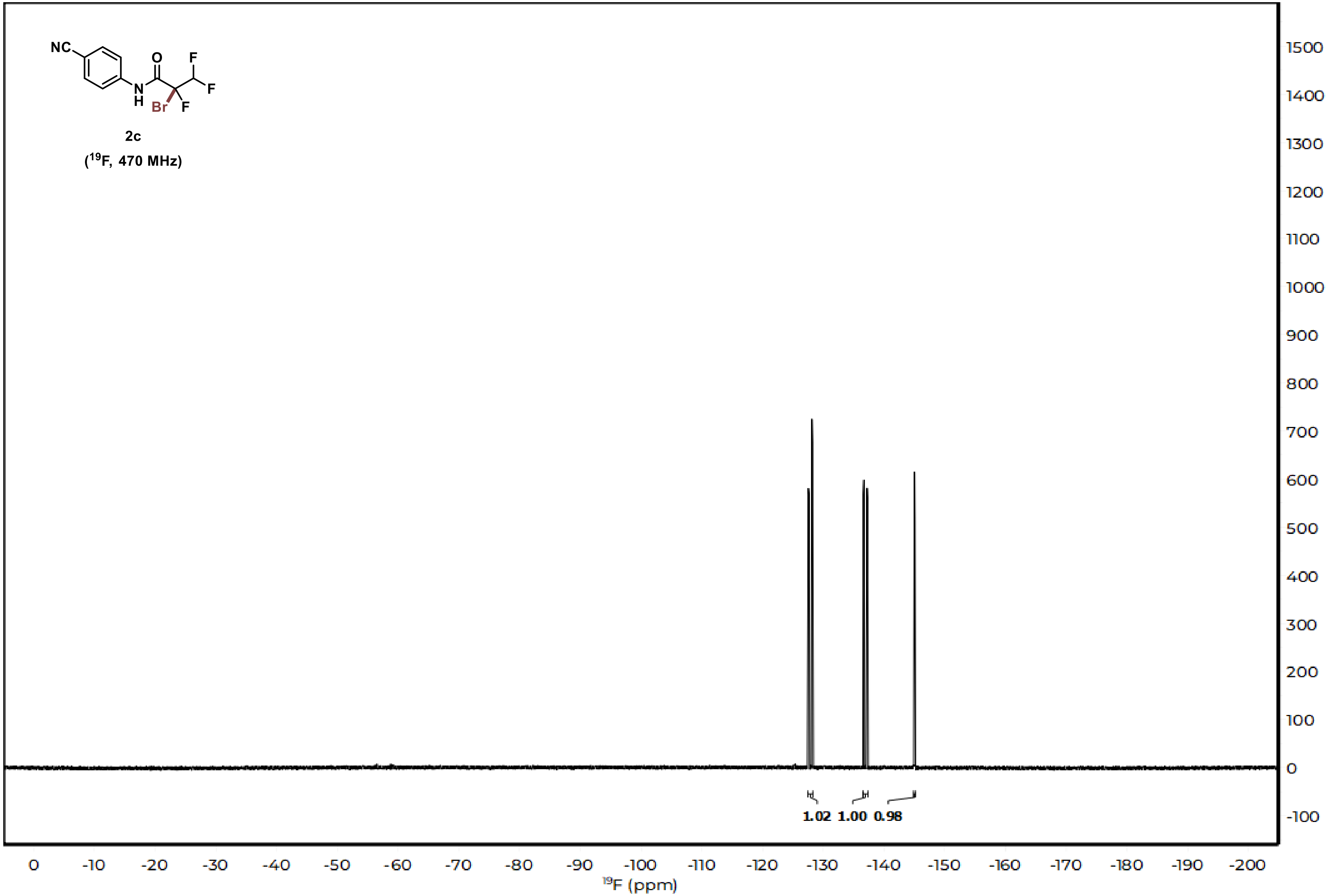

NC
O
F
F
N
H
Br
F
2c
(^{19}F, 470 MHz)
1500
1400
1300
1200
1100
1000
900
800
700
600
500
400
300
200
100
0
-100
1.02 1.00 0.98
0 -10 -20 -30 -40 -50 -60 -70 -80 -90 -100 -110 -120 -130 -140 -150 -160 -170 -180 -190 -200
^{19}F (ppm)

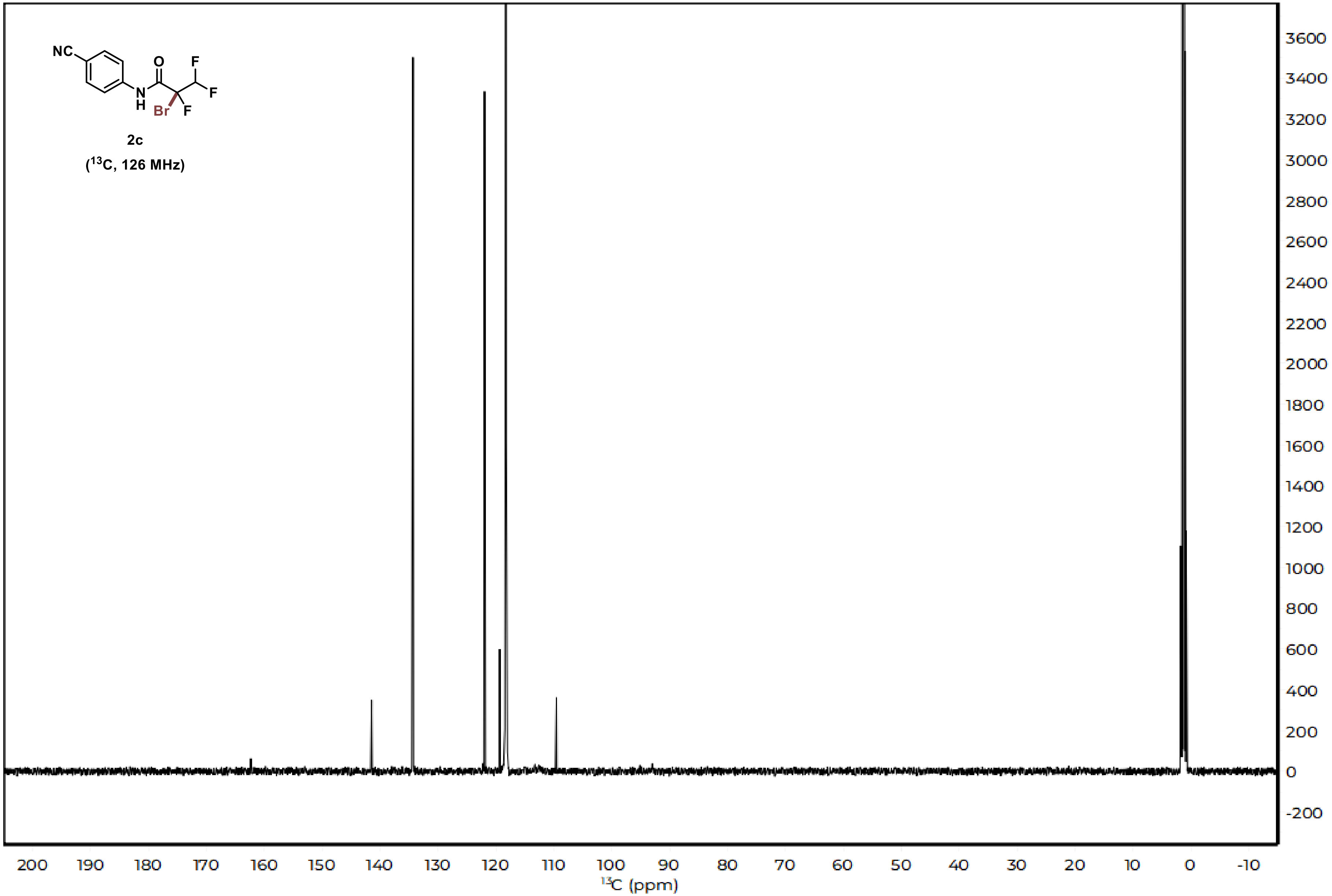
NC
O
F
F
N
H
Br
F
2c
(13C, 126 MHz)
3600
3400
3200
3000
2800
2600
2400
2200
2000
1800
1600
1400
1200
1000
800
600
400
200
0
-200
200 190 180 170 160 150 140 130 120 110 100 90 80 70 60 50 40 30 20 10 0 -10
13C (ppm)

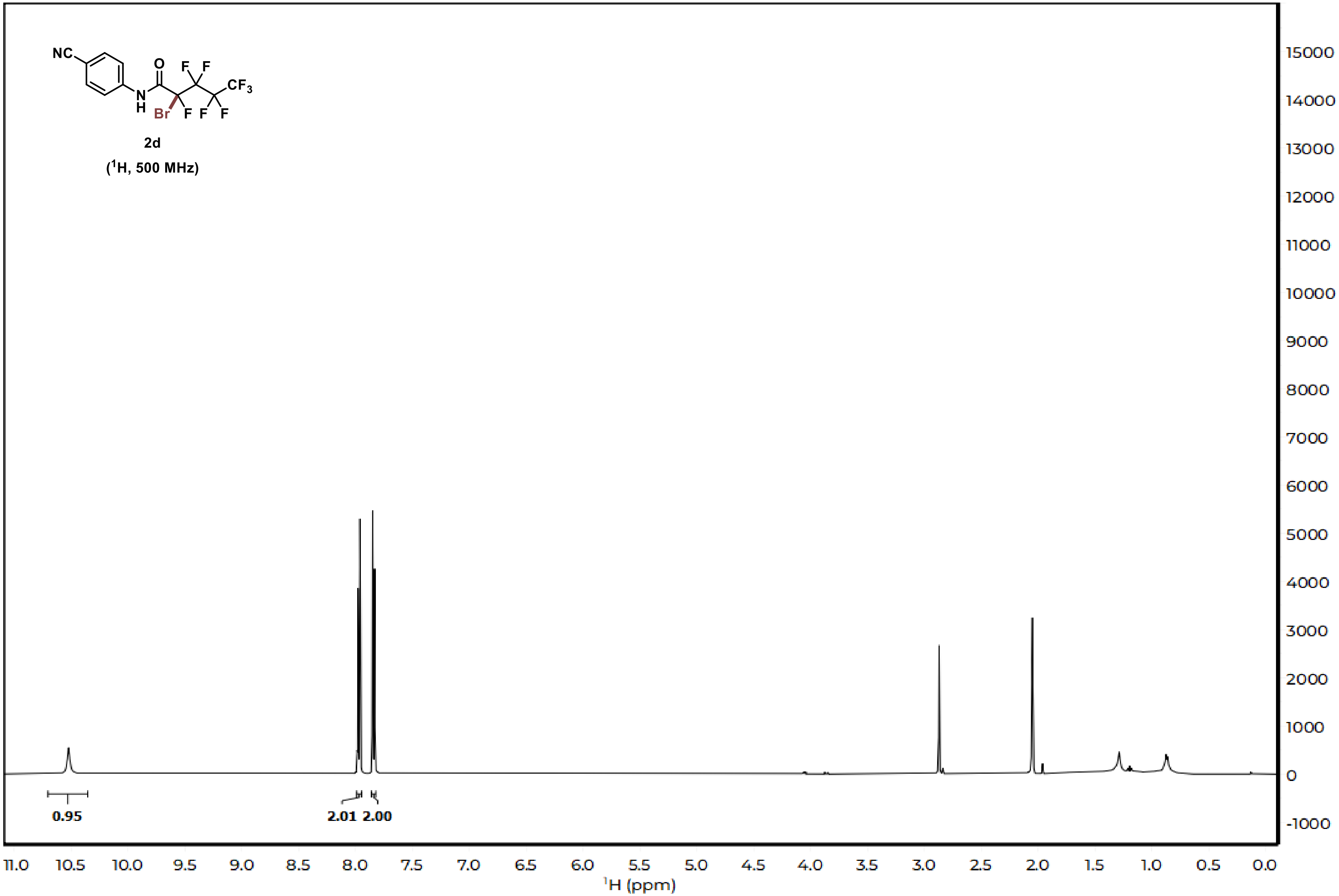

NC
O
F F
CF3
N
H
Br
F F F
2d
(1H, 500 MHz)
15000
14000
13000
12000
11000
10000
9000
8000
7000
6000
5000
4000
3000
2000
1000
0
-1000
0.95
2.01 2.00
11.0 10.5 10.0 9.5 9.0 8.5 8.0 7.5 7.0 6.5 6.0 5.5 5.0 4.5 4.0 3.5 3.0 2.5 2.0 1.5 1.0 0.5 0.0
1H (ppm)

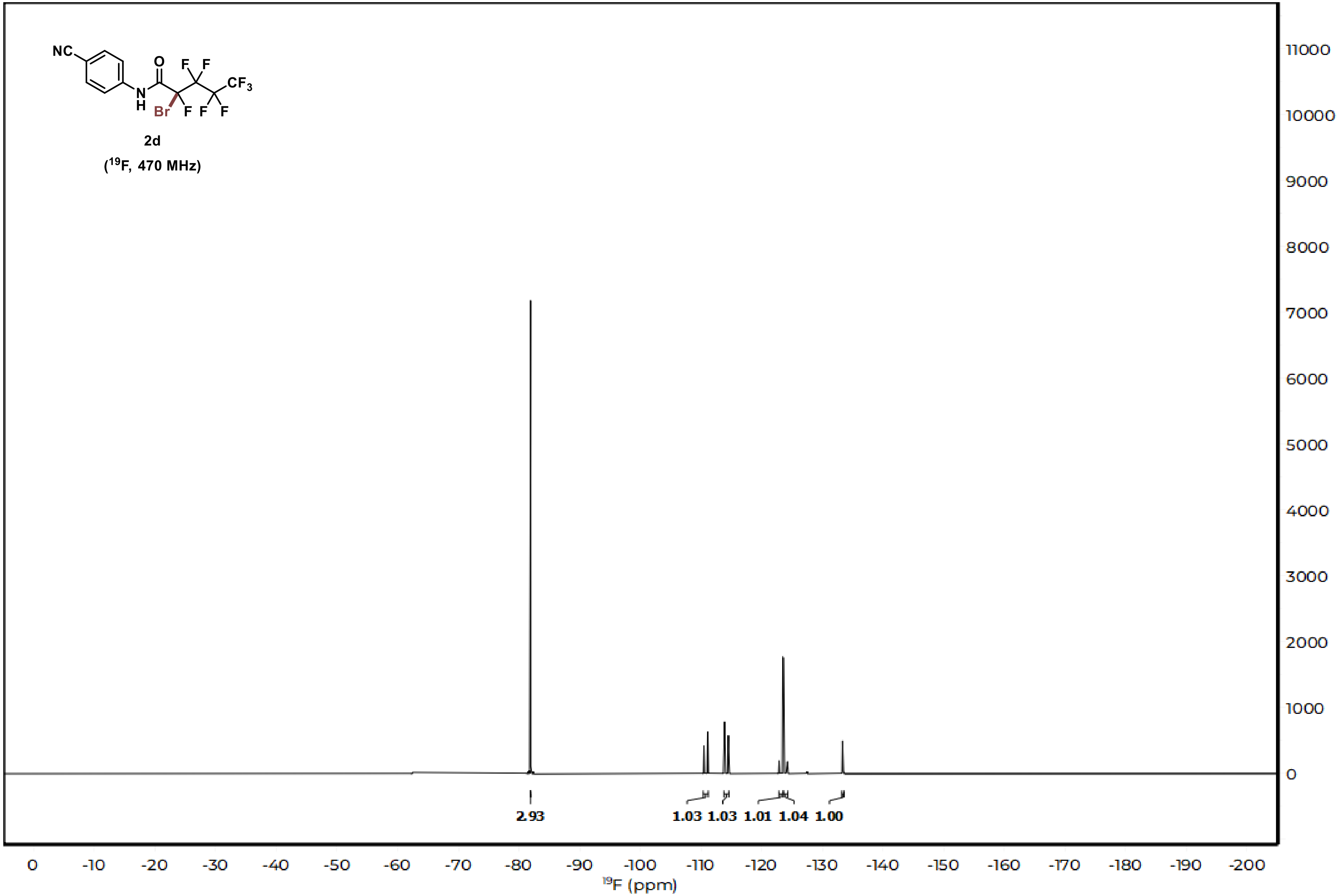
NC
O
F F
CF3
N
H
Br
F F F
2d
(19F, 470 MHz)
11000
10000
9000
8000
7000
6000
5000
4000
3000
2000
1000
0
2.93
1.03 1.03 1.01 1.04 1.00
0 -10 -20 -30 -40 -50 -60 -70 -80 -90 -100 -110 -120 -130 -140 -150 -160 -170 -180 -190 -200
19F (ppm)

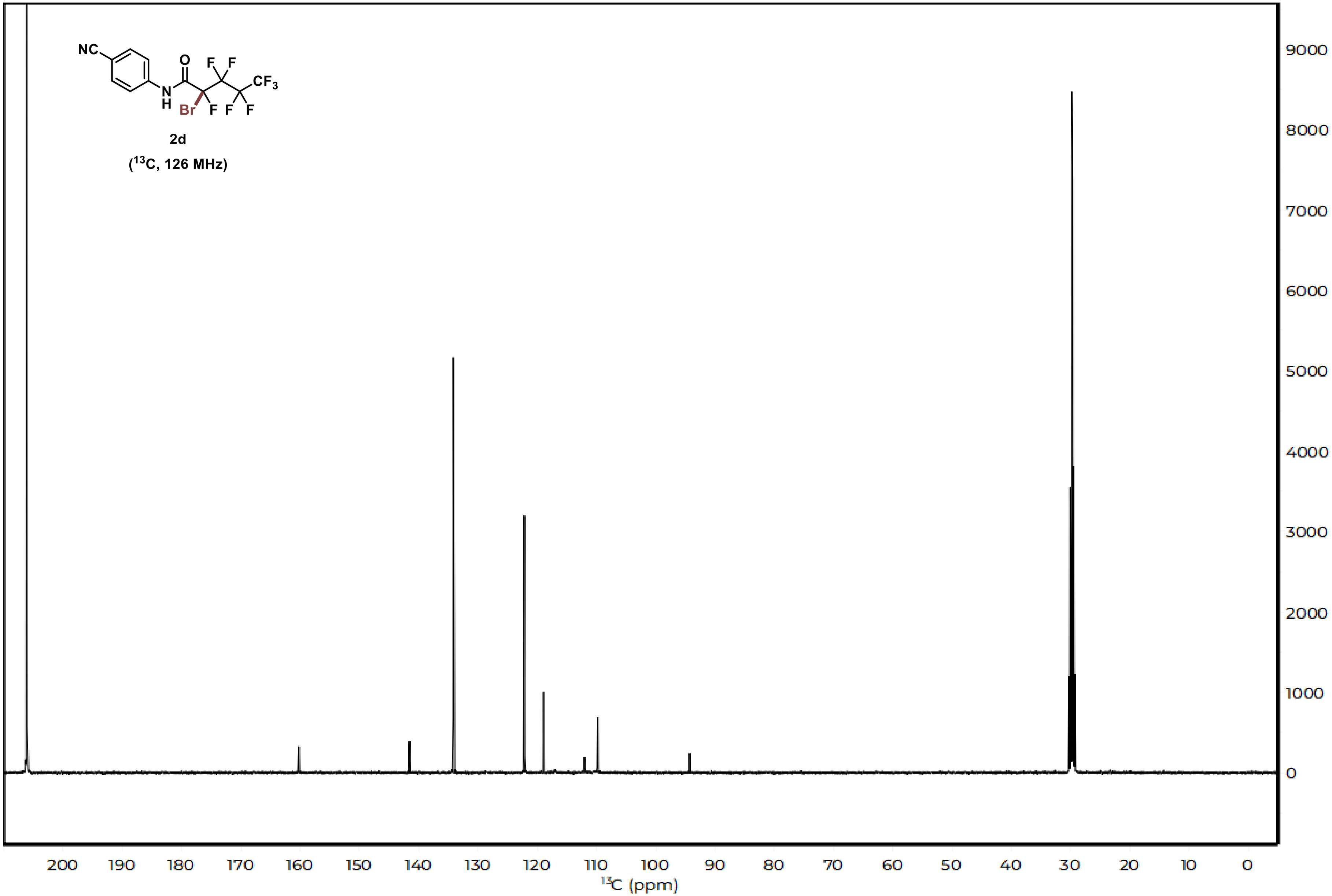
NC
O
F F
CF3
N
H
Br
F F F
2d
(13C, 126 MHz)
9000
8000
7000
6000
5000
4000
3000
2000
1000
0
200 190 180 170 160 150 140 130 120 110 100 90 80 70 60 50 40 30 20 10 0
13C (ppm)

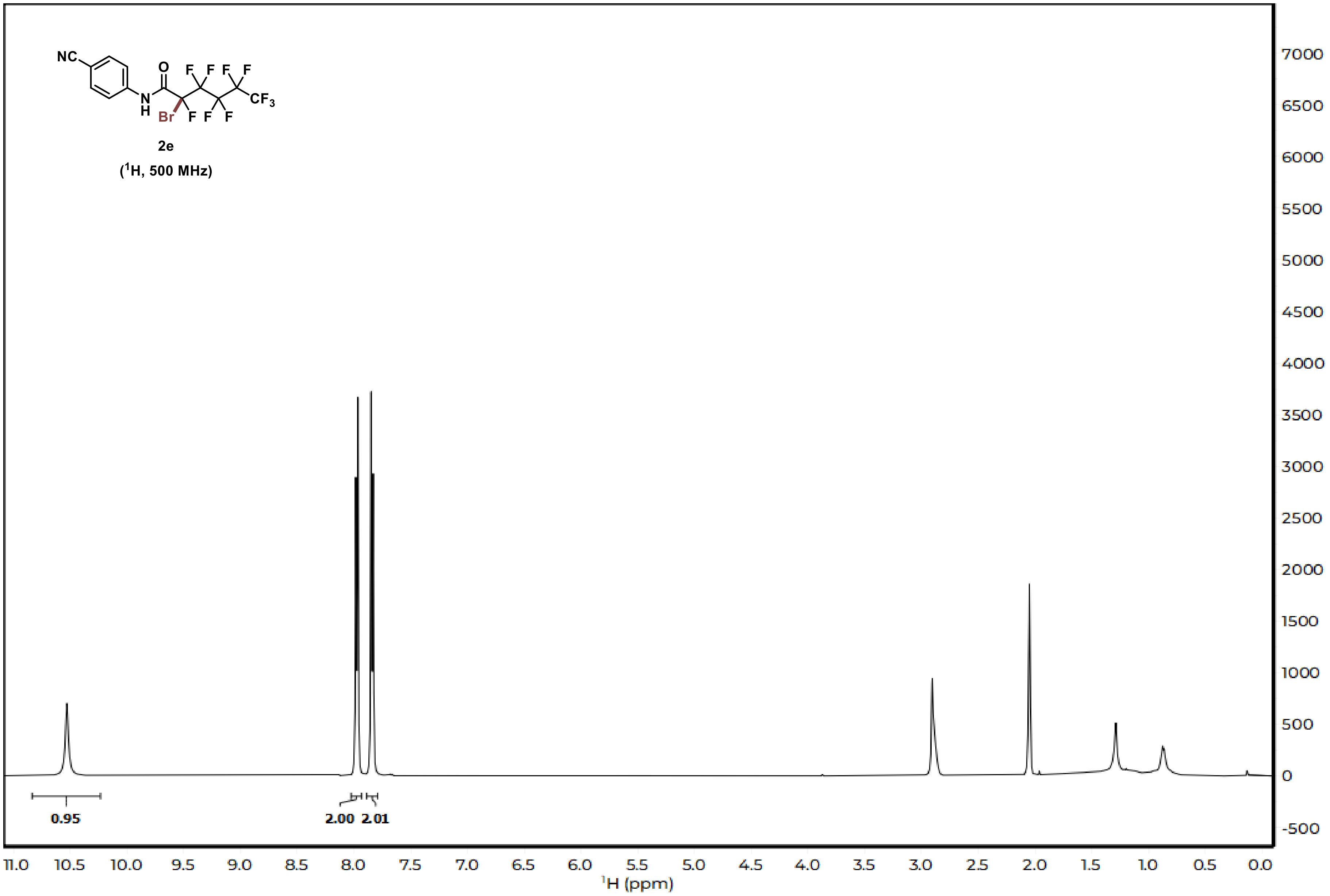
NC
O
F F F F
CF3
N
H
Br
F F F
2e
(1H, 500 MHz)
7000
6500
6000
5500
5000
4500
4000
3500
3000
2500
2000
1500
1000
500
0
-500
0.95
2.00 2.01
11.0 10.5 10.0 9.5 9.0 8.5 8.0 7.5 7.0 6.5 6.0 5.5 5.0 4.5 4.0 3.5 3.0 2.5 2.0 1.5 1.0 0.5 0.0
1H (ppm)

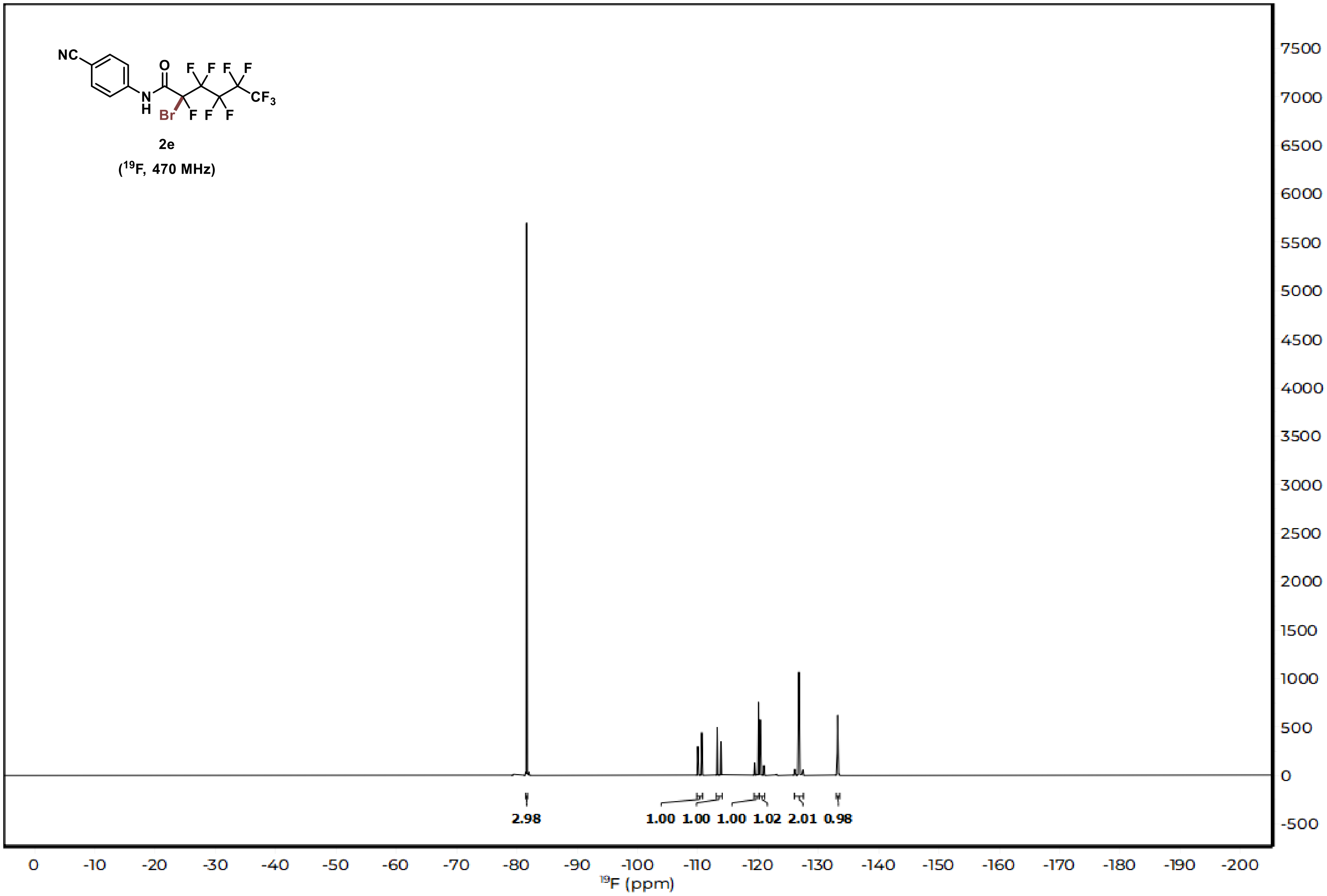

NC
O
F F F F
N
H
Br
F F F
CF3
2e
(19F, 470 MHz)
2.98
1.00 1.00 1.00 1.02 2.01 0.98
7500
7000
6500
6000
5500
5000
4500
4000
3500
3000
2500
2000
1500
1000
500
0
-500
0 -10 -20 -30 -40 -50 -60 -70 -80 -90 -100 -110 -120 -130 -140 -150 -160 -170 -180 -190 -200
19F (ppm)

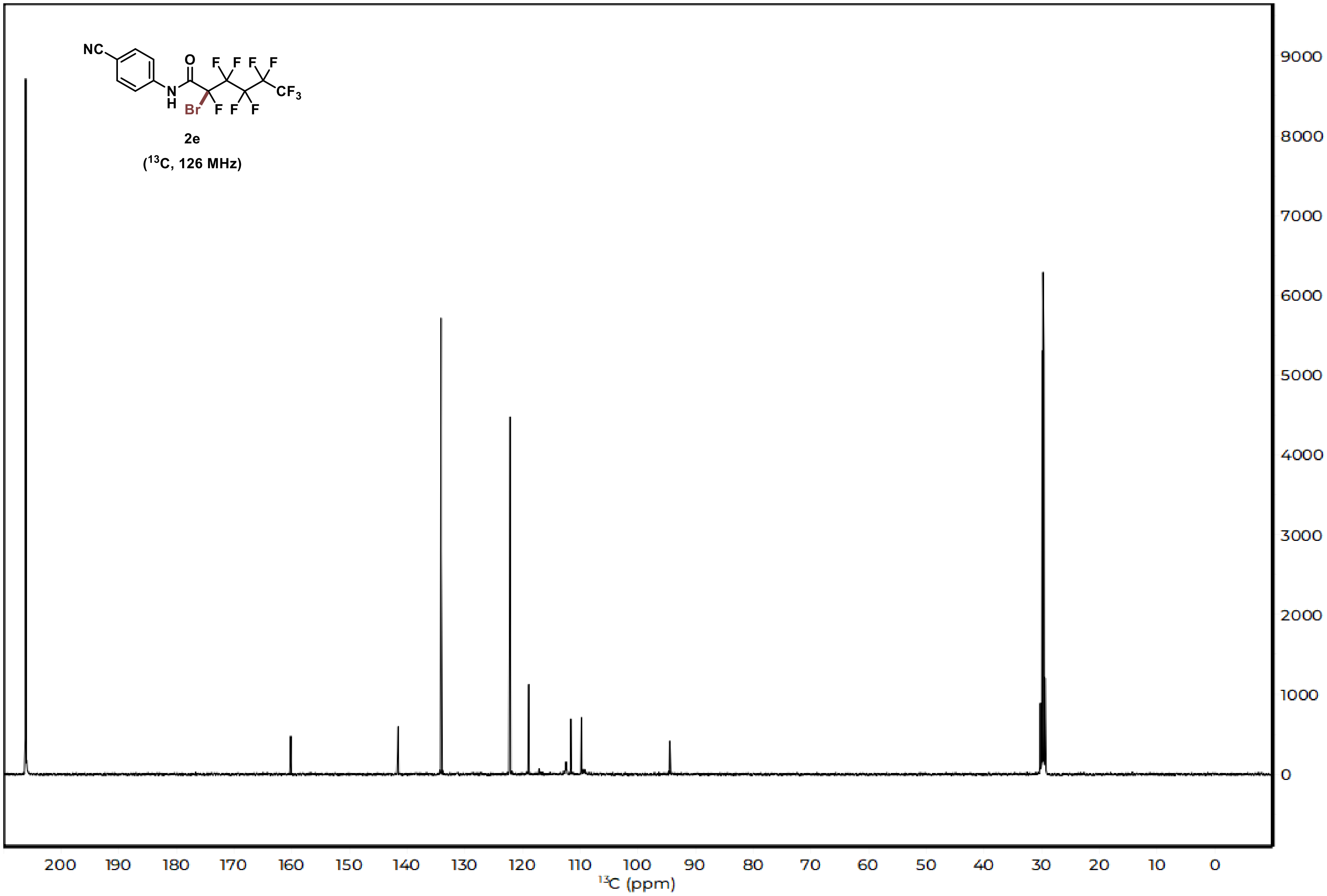
NC
O
F F F F
N
H
Br
F F F
CF3
2e
(13C, 126 MHz)
9000
8000
7000
6000
5000
4000
3000
2000
1000
0
200 190 180 170 160 150 140 130 120 110 100 90 80 70 60 50 40 30 20 10 0
13C (ppm)

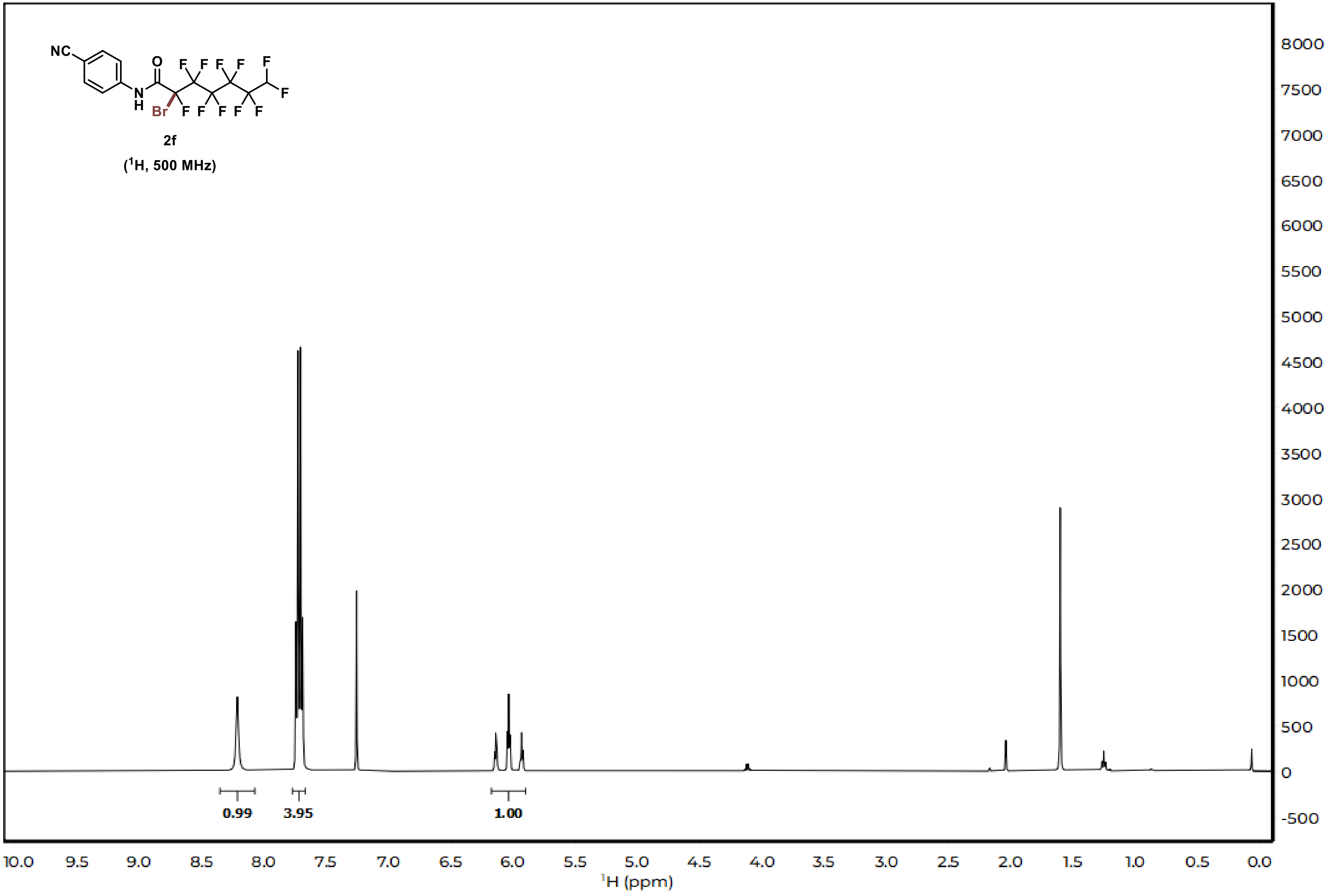
NC
O
F F F F F
N
H
Br F F F F F
F
2f
(¹H, 500 MHz)
0.99
3.95
1.00
8000
7500
7000
6500
6000
5500
5000
4500
4000
3500
3000
2500
2000
1500
1000
500
0
-500
10.0 9.5 9.0 8.5 8.0 7.5 7.0 6.5 6.0 5.5 5.0 4.5 4.0 3.5 3.0 2.5 2.0 1.5 1.0 0.5 0.0
¹H (ppm)

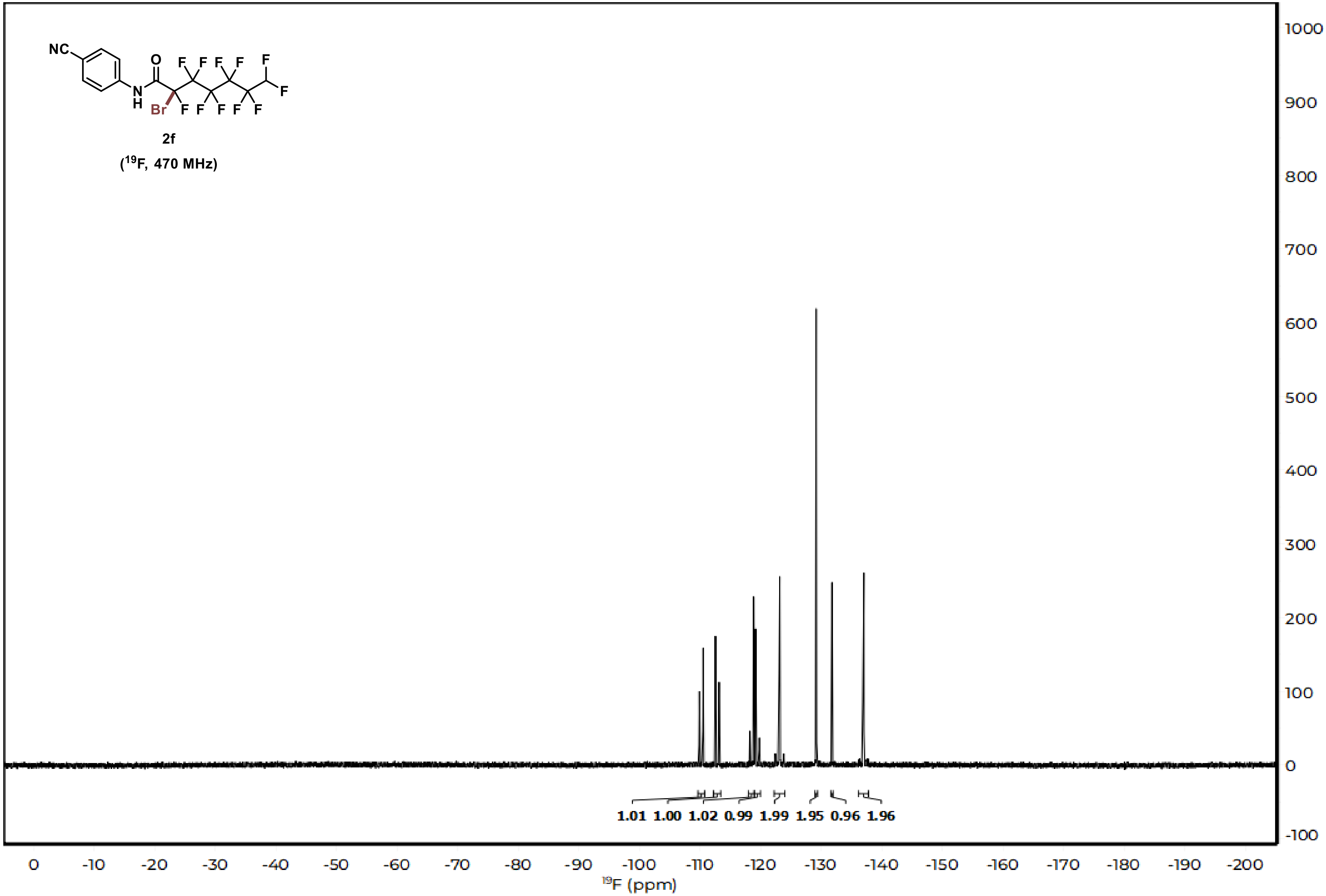
NC
O
F F F F F
F
N
H
Br F F F F F
2f
(19F, 470 MHz)
1000
900
800
700
600
500
400
300
200
100
0
-100
1.01 1.00 1.02 0.99 1.99 1.95 0.96 1.96
0 -10 -20 -30 -40 -50 -60 -70 -80 -90 -100 -110 -120 -130 -140 -150 -160 -170 -180 -190 -200
19F (ppm)

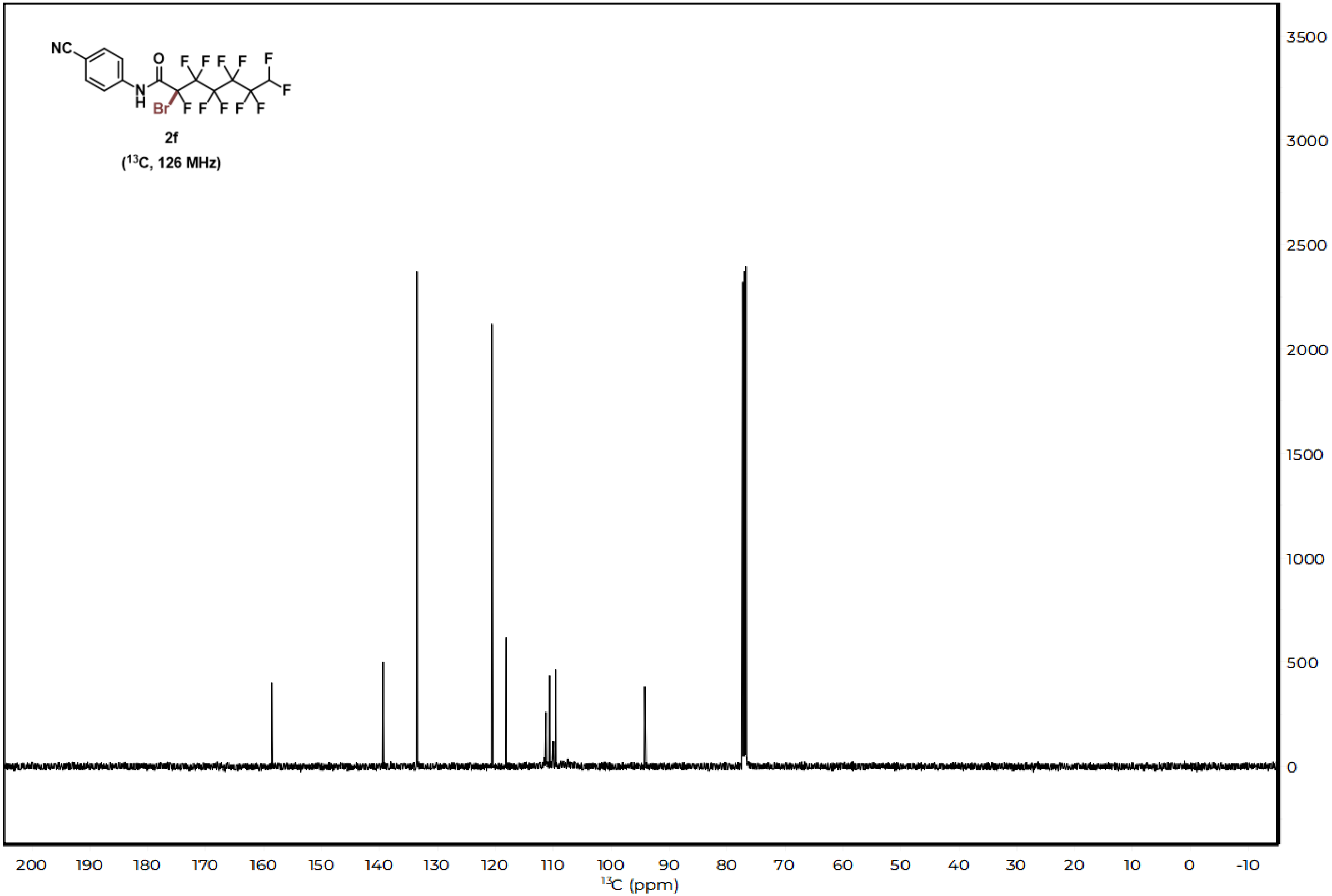
2f
(13C, 126 MHz)
3500
3000
2500
2000
1500
1000
500
0
200 190 180 170 160 150 140 130 120 110 100 90 80 70 60 50 40 30 20 10 0 -10
13C (ppm)

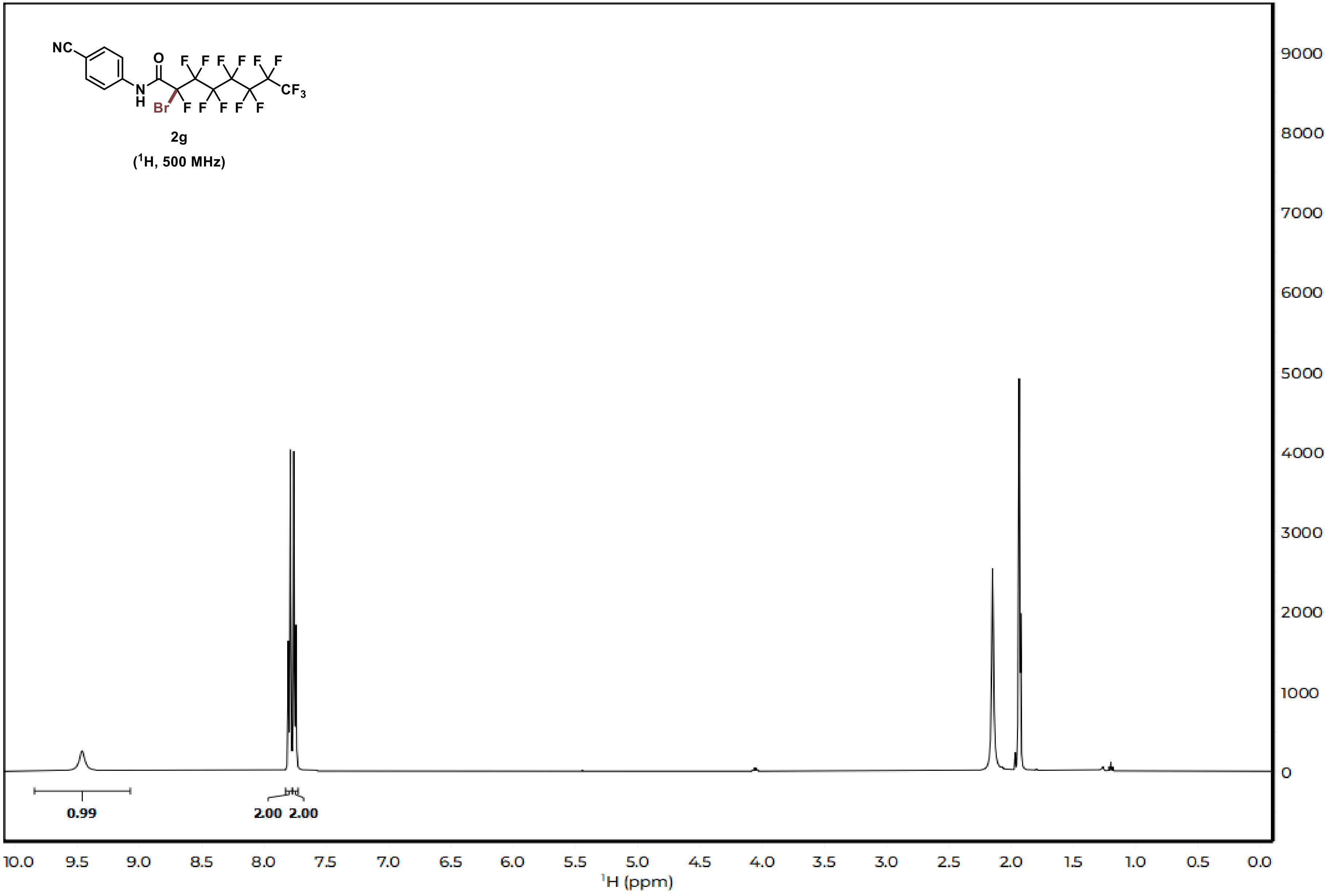
NC
O
N
H
Br
F F F F F F
F F F F F
CF3
2g
(1H, 500 MHz)
9000
8000
7000
6000
5000
4000
3000
2000
1000
0
0.99
2.00 2.00
10.0 9.5 9.0 8.5 8.0 7.5 7.0 6.5 6.0 5.5 5.0 4.5 4.0 3.5 3.0 2.5 2.0 1.5 1.0 0.5 0.0
1H (ppm)

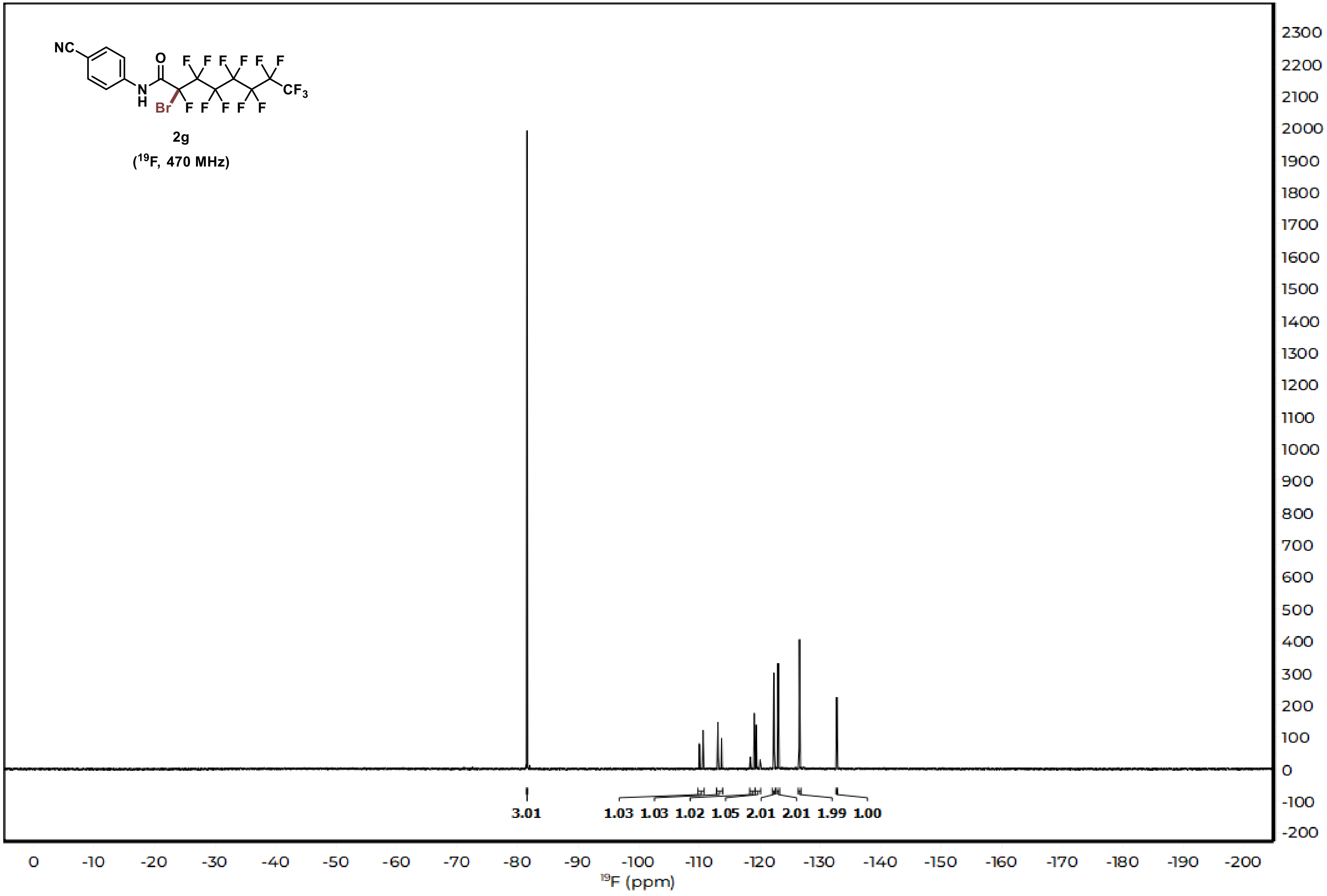

NC
O
N
H
Br
F F F F F F
F F F F F
CF3
2g
(19F, 470 MHz)
3.01
1.03 1.03 1.02 1.05 2.01 2.01 1.99 1.00
2300 2200 2100 2000 1900 1800 1700 1600 1500 1400 1300 1200 1100 1000 900 800 700 600 500 400 300 200 100 0 -100 -200
0 -10 -20 -30 -40 -50 -60 -70 -80 -90 -100 -110 -120 -130 -140 -150 -160 -170 -180 -190 -200
19F (ppm)

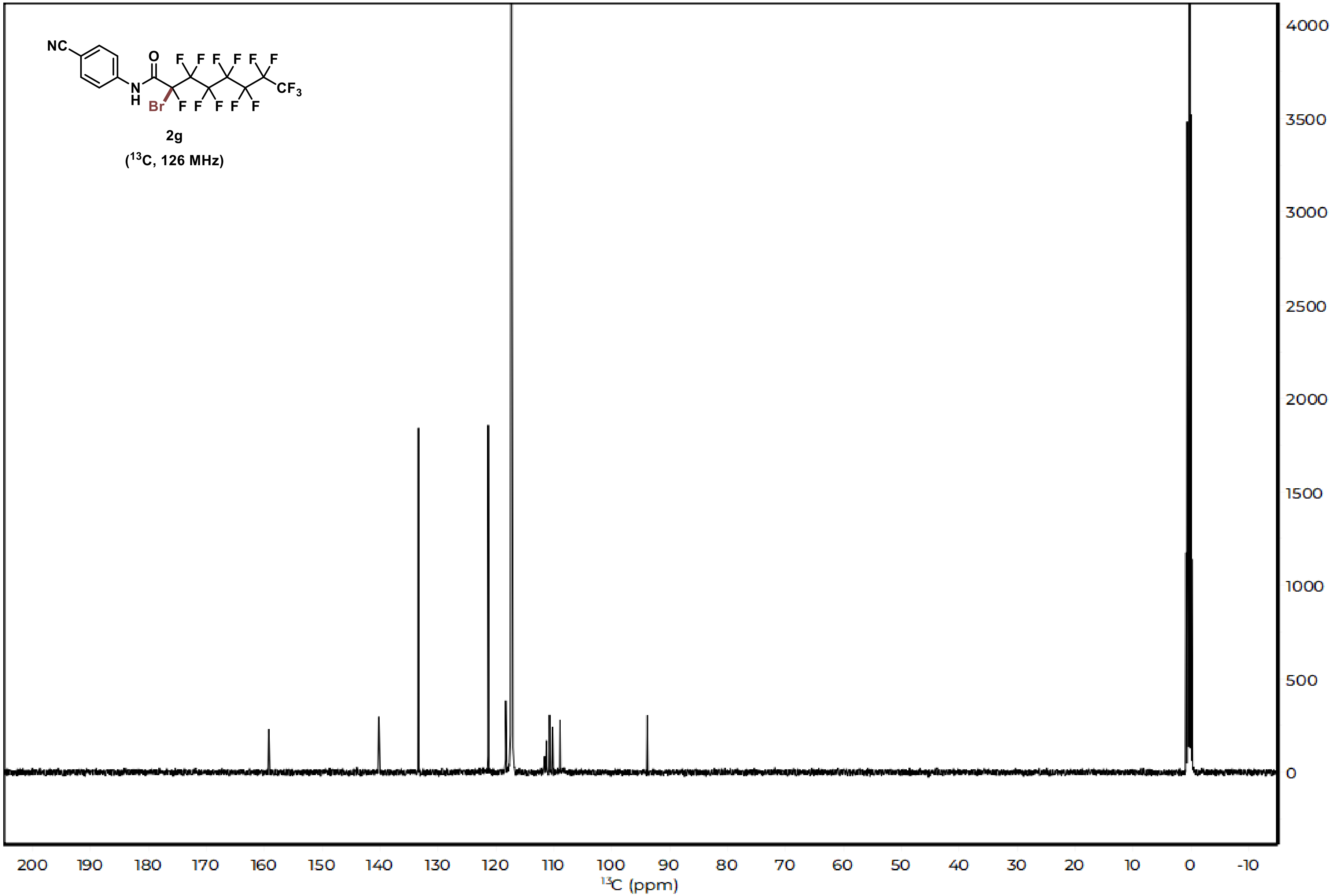

NC
O
N
H
Br
F F F F F F
F F F F F
CF3
2g
(13C, 126 MHz)
4000
3500
3000
2500
2000
1500
1000
500
0
200 190 180 170 160 150 140 130 120 110 100 90 80 70 60 50 40 30 20 10 0 -10
13C (ppm)

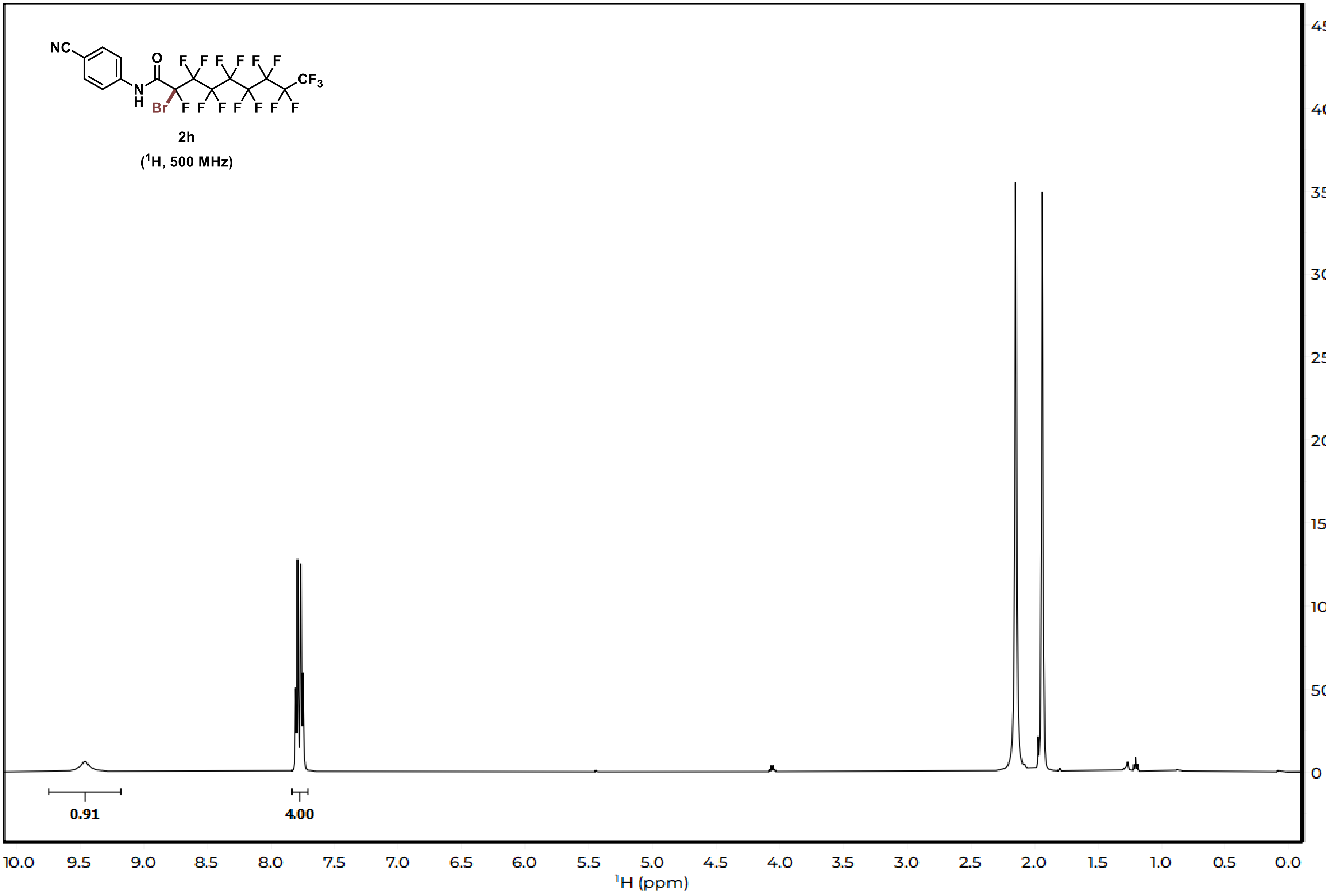
NC
O
F F F F F F
CF3
N
H
Br F F F F F F F
2h
(1H, 500 MHz)
0.91
4.00
10.0 9.5 9.0 8.5 8.0 7.5 7.0 6.5 6.0 5.5 5.0 4.5 4.0 3.5 3.0 2.5 2.0 1.5 1.0 0.5 0.0
1H (ppm)

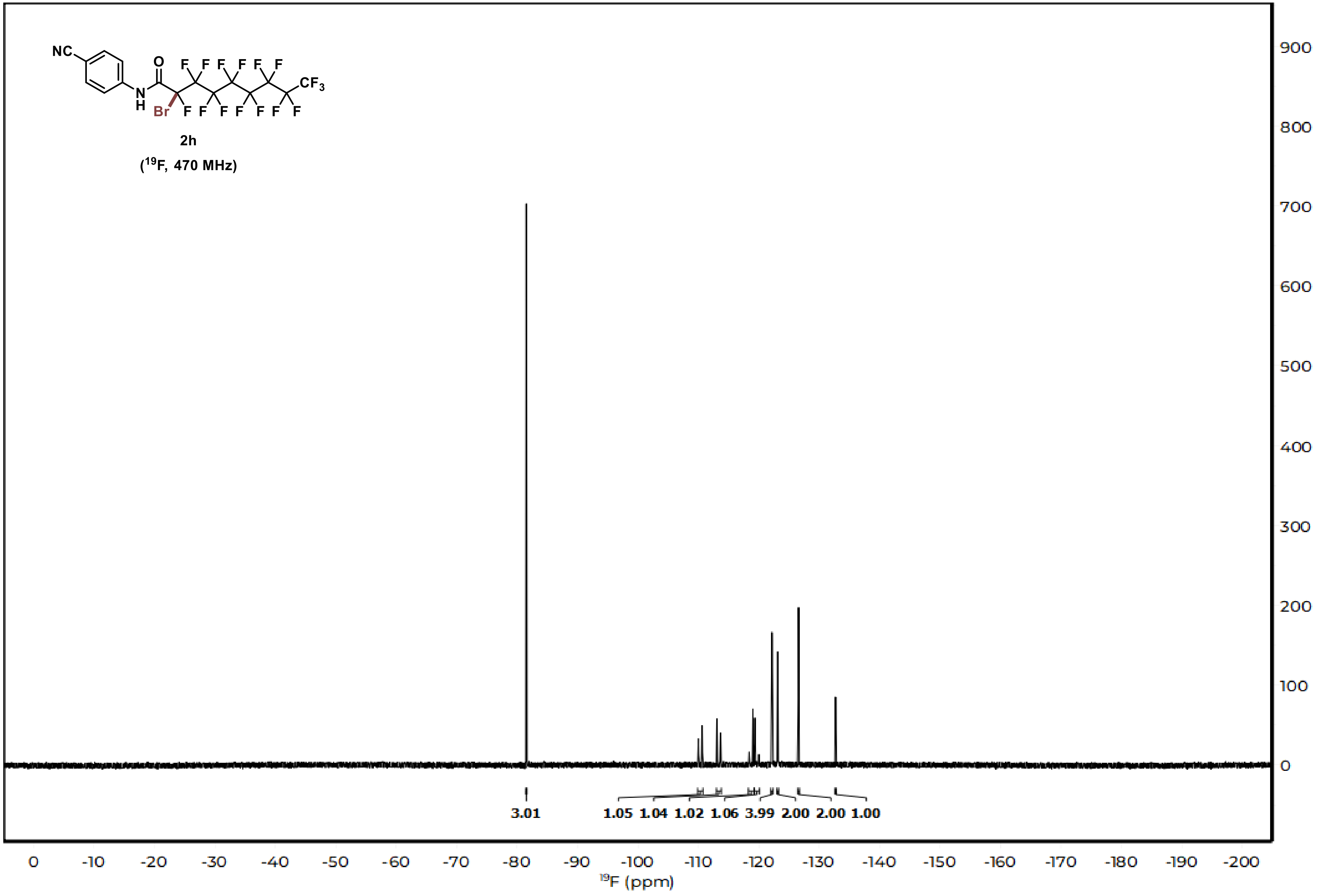

NC
O
F F F F F F
CF3
N
H
Br
F F F F F F F
2h
(19F, 470 MHz)
900
800
700
600
500
400
300
200
100
0
3.01
1.05 1.04 1.02 1.06 3.99 2.00 2.00 1.00
0 -10 -20 -30 -40 -50 -60 -70 -80 -90 -100 -110 -120 -130 -140 -150 -160 -170 -180 -190 -200
19F (ppm)

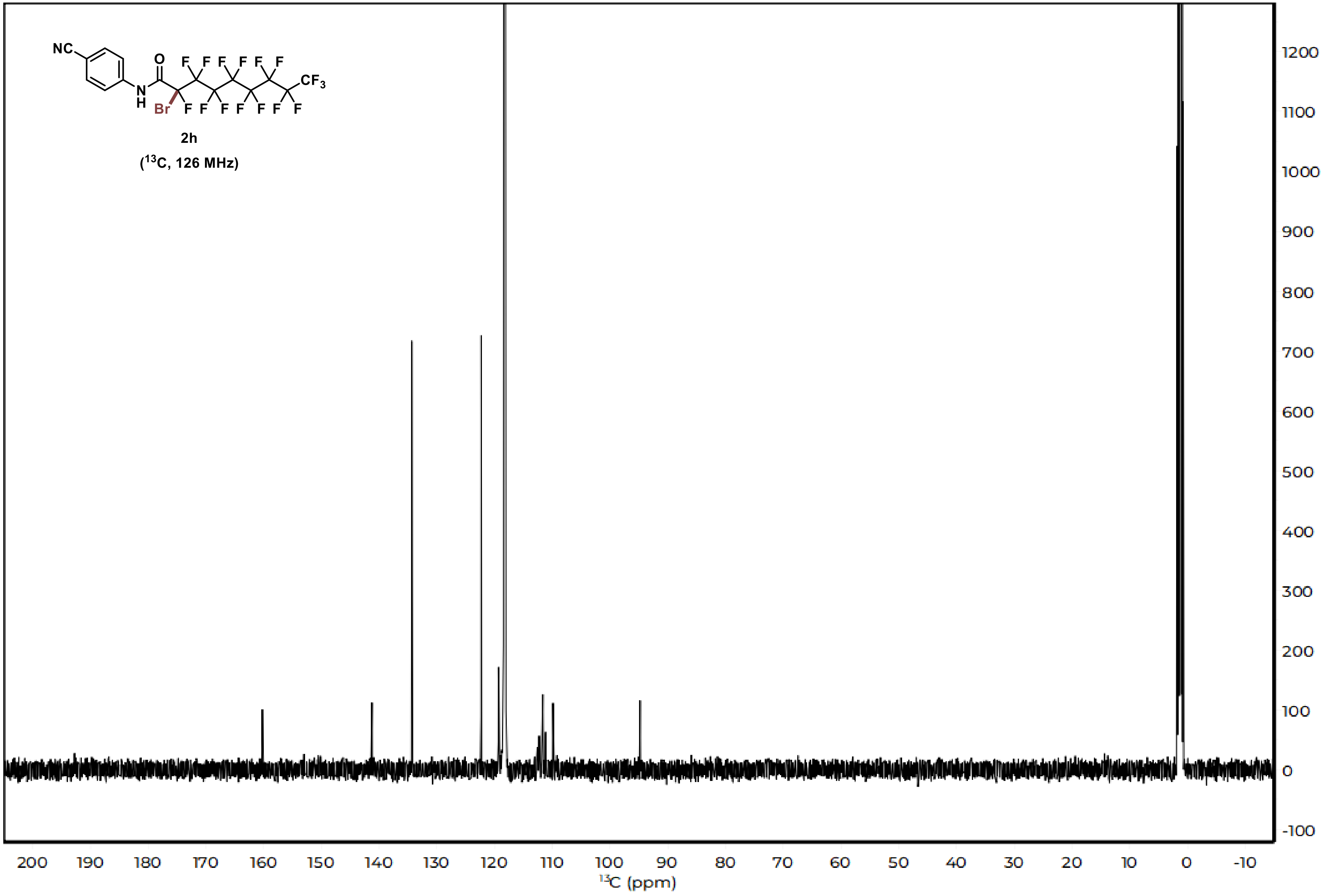
NC
O
N
H
Br
F F F F F F
CF3
F F F F F F
2h
(13C, 126 MHz)
1200
1100
1000
900
800
700
600
500
400
300
200
100
0
-100
200 190 180 170 160 150 140 130 120 110 100 90 80 70 60 50 40 30 20 10 0 -10
13C (ppm)

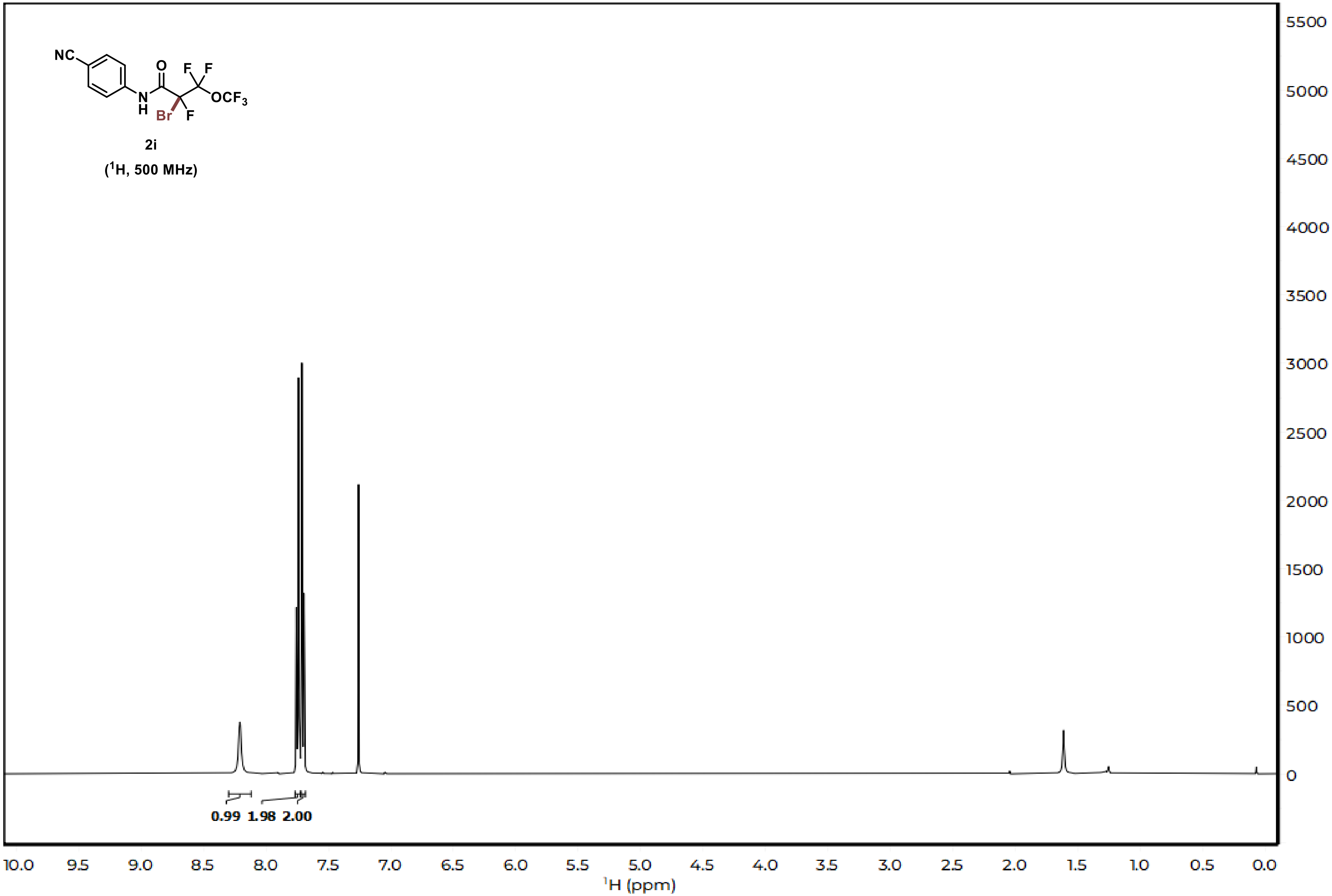

NC
O
F F
N
H
Br
F
OCF3
2i
(1H, 500 MHz)
0.99 1.98 2.00
5500
5000
4500
4000
3500
3000
2500
2000
1500
1000
500
0
10.0 9.5 9.0 8.5 8.0 7.5 7.0 6.5 6.0 5.5 5.0 4.5 4.0 3.5 3.0 2.5 2.0 1.5 1.0 0.5 0.0
1H (ppm)

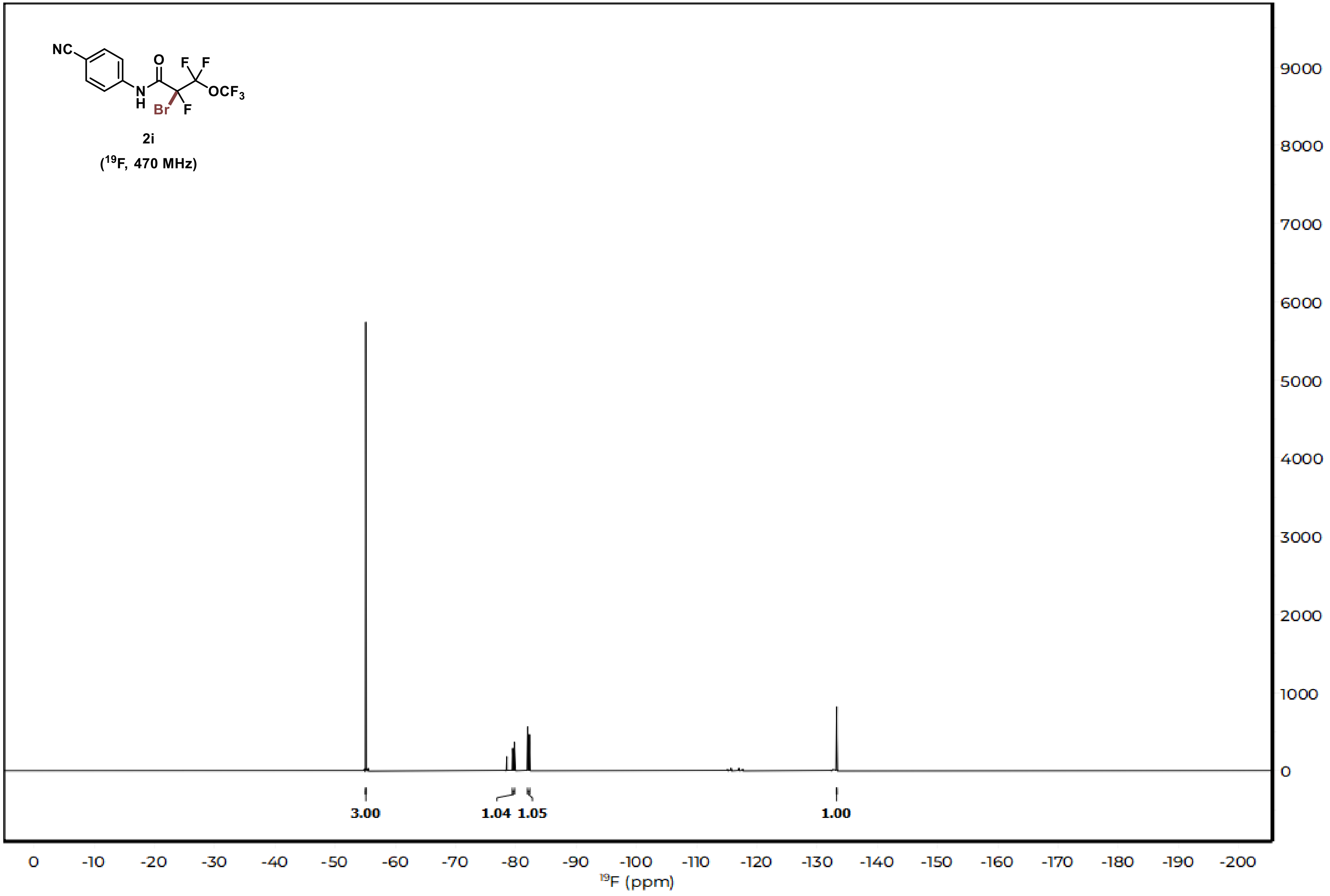
NC
O
F F
OCF3
N
H
Br
F
2i
(19F, 470 MHz)
9000
8000
7000
6000
5000
4000
3000
2000
1000
0
3.00
1.04 1.05
1.00
0 -10 -20 -30 -40 -50 -60 -70 -80 -90 -100 -110 -120 -130 -140 -150 -160 -170 -180 -190 -200
19F (ppm)

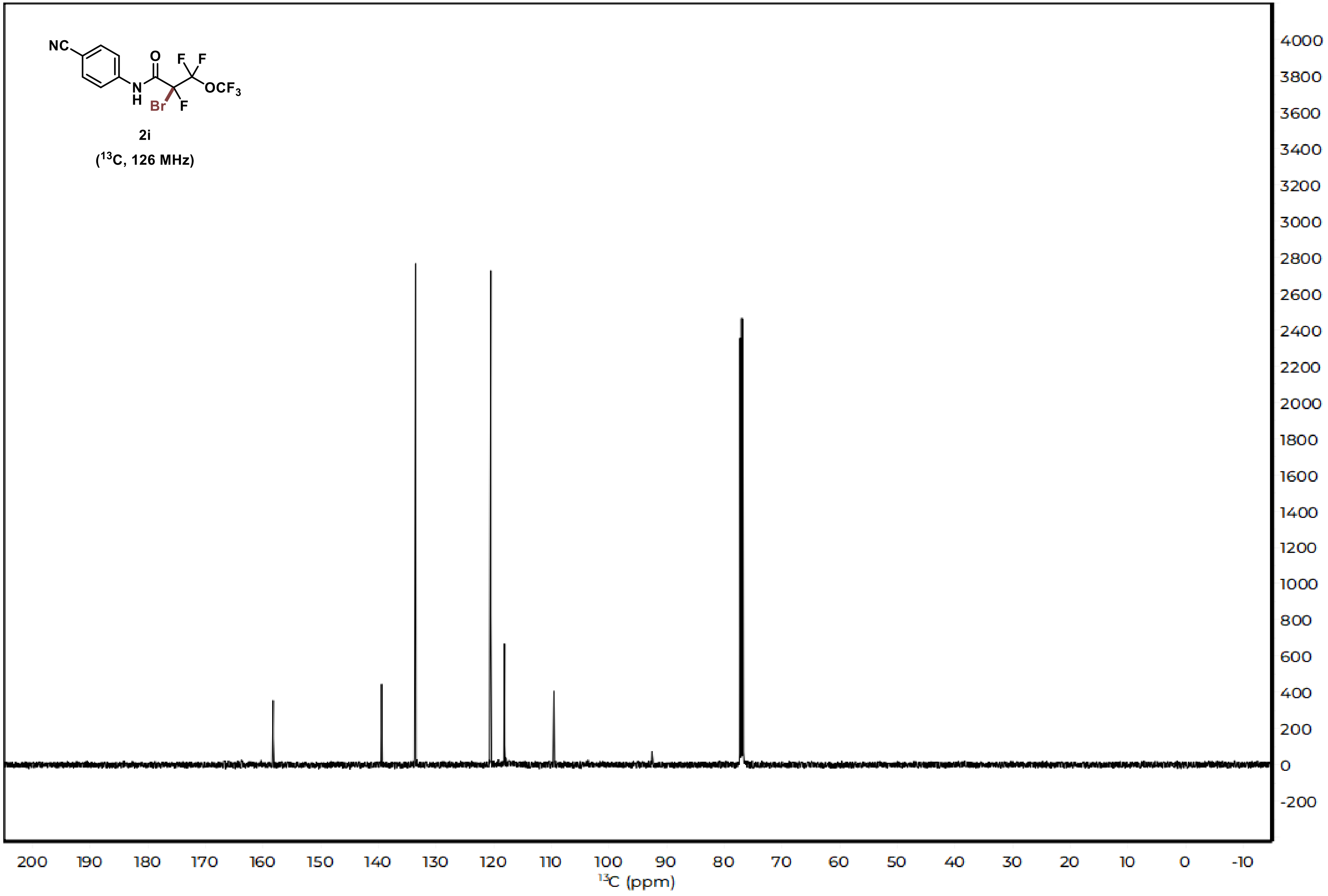
NC
O
F
F
N
H
Br
F
OCF3
2i
(13C, 126 MHz)
4000
3800
3600
3400
3200
3000
2800
2600
2400
2200
2000
1800
1600
1400
1200
1000
800
600
400
200
0
-200
200
190
180
170
160
150
140
130
120
110
100
90
80
70
60
50
40
30
20
10
0
-10
13C (ppm)

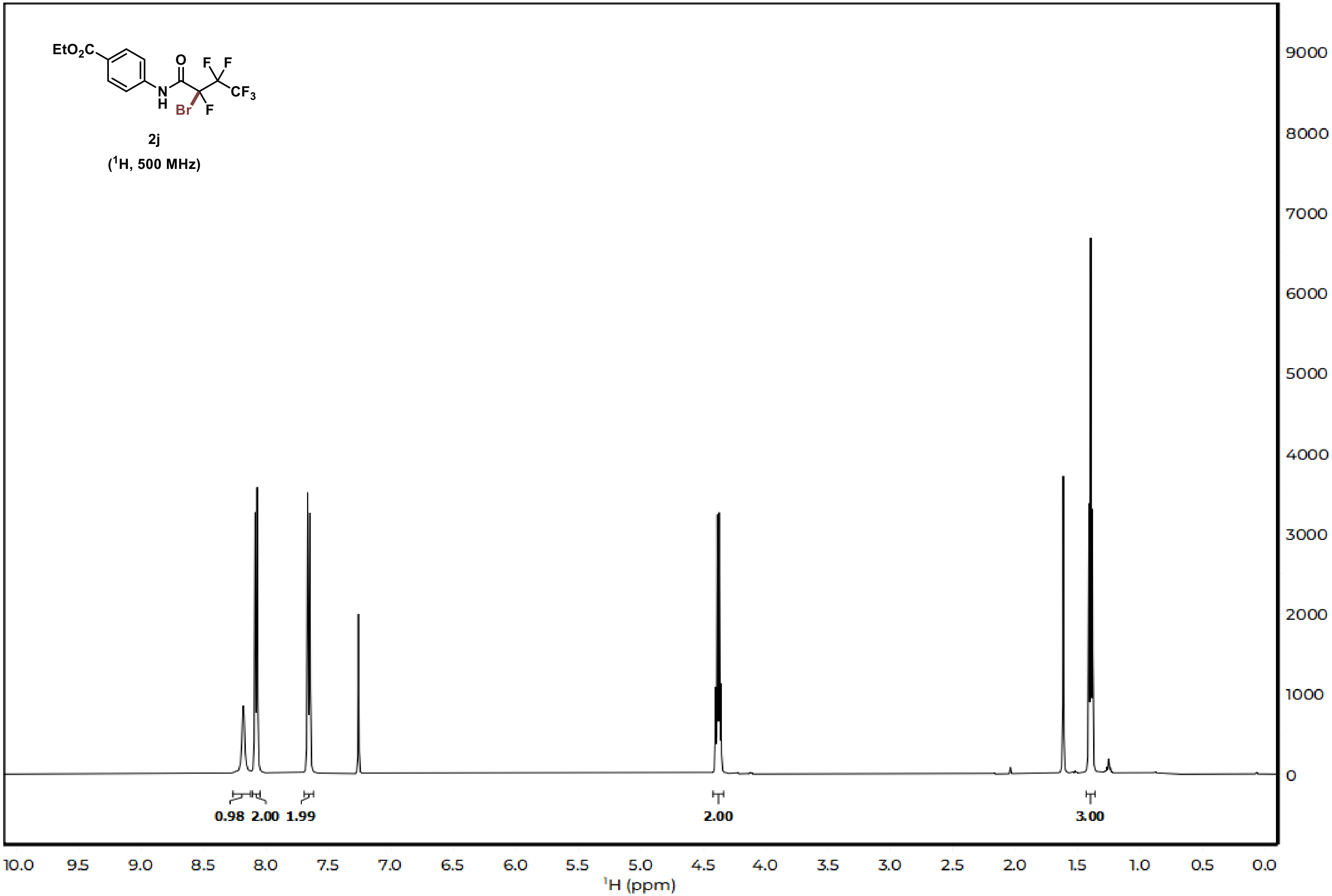

EtO2C
O
F F
N
H
Br
F
CF3
2j
(1H, 500 MHz)
9000
8000
7000
6000
5000
4000
3000
2000
1000
0
0.98 2.00 1.99
2.00
3.00
10.0 9.5 9.0 8.5 8.0 7.5 7.0 6.5 6.0 5.5 5.0 4.5 4.0 3.5 3.0 2.5 2.0 1.5 1.0 0.5 0.0
1H (ppm)

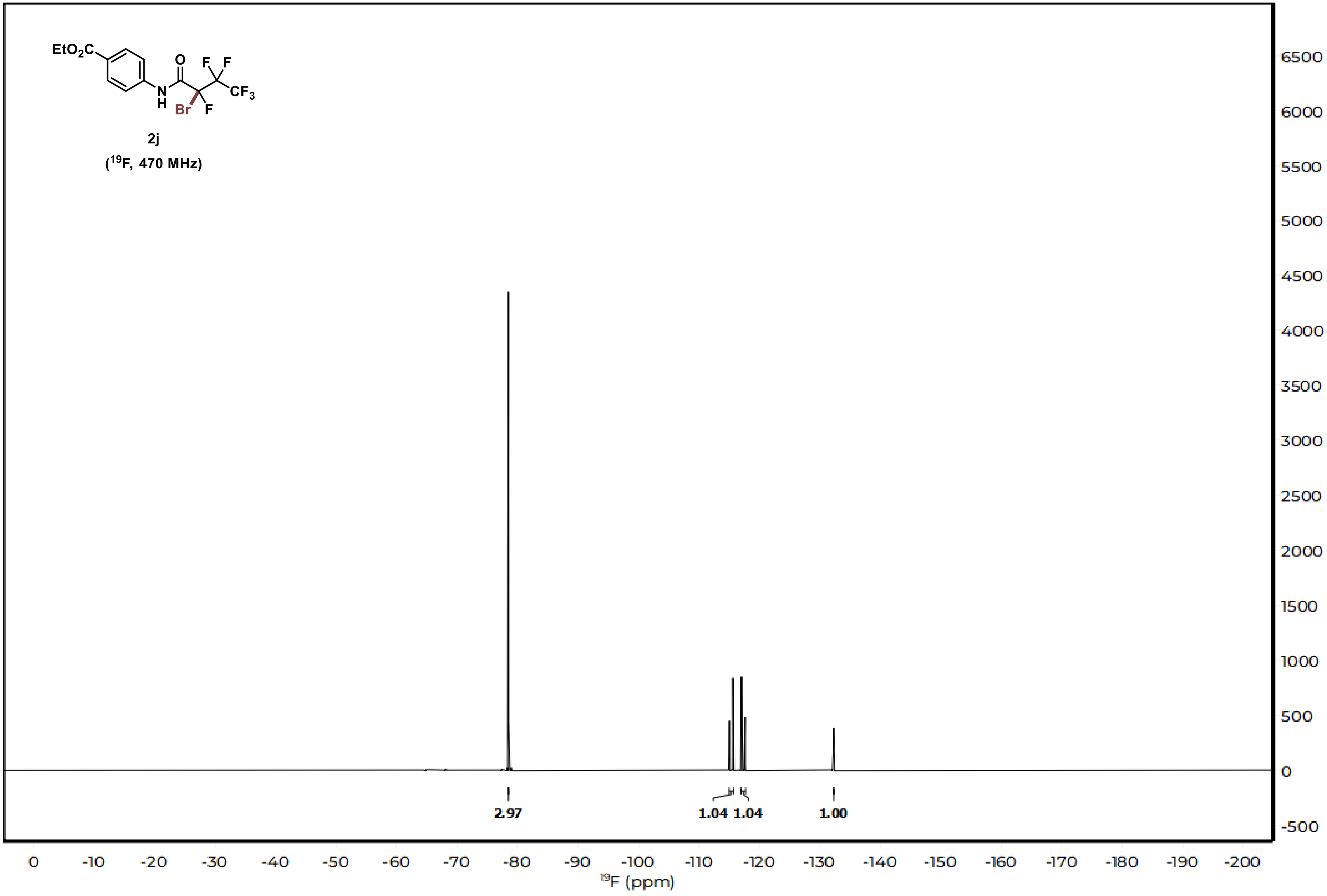

EtO2C
O
F
F
N
H
CF3
Br
F
2j
(19F, 470 MHz)
2.97
1.04 1.04
1.00
19F (ppm)

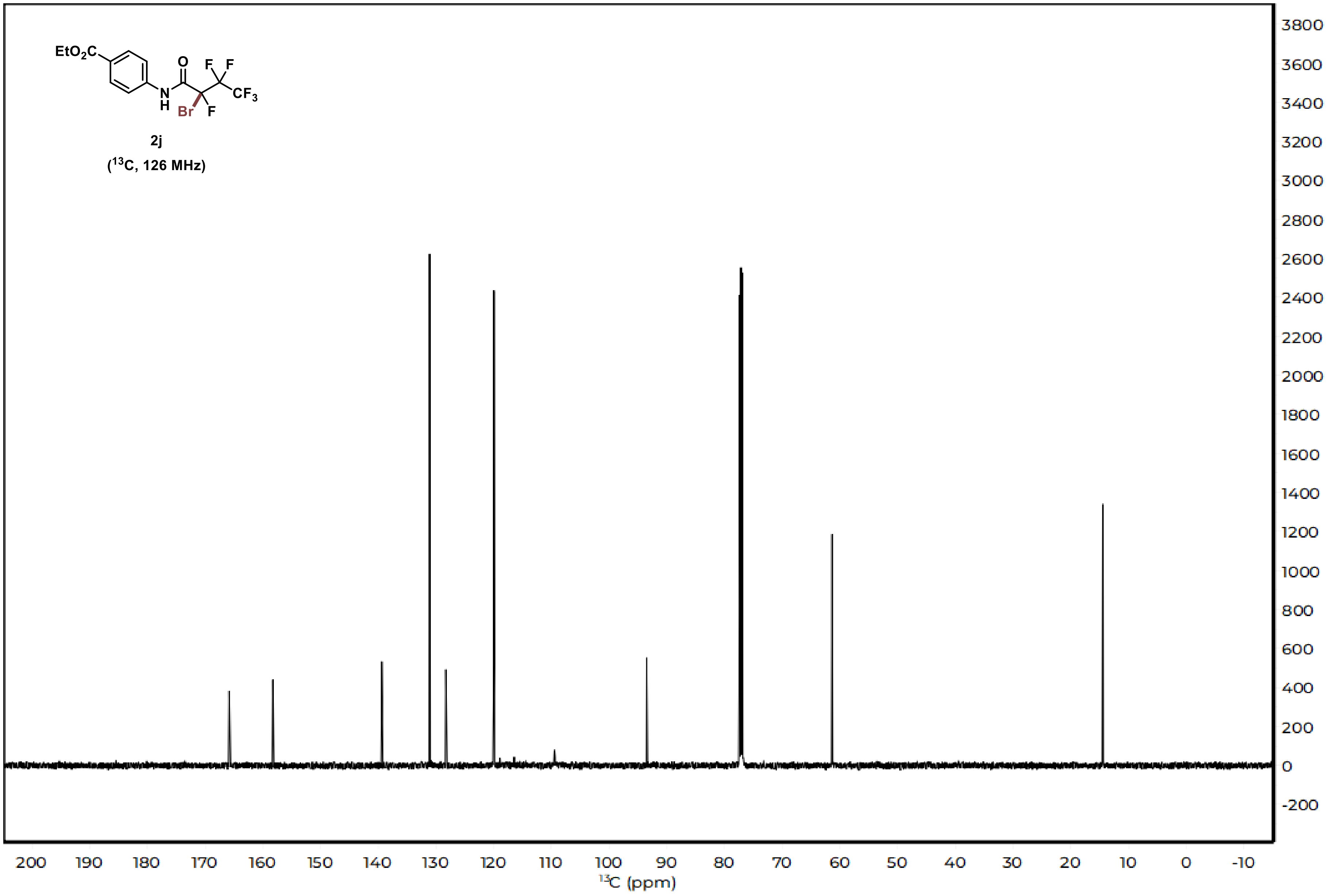

EtO2C
O
F F
N
H
CF3
Br
F
2j
(13C, 126 MHz)
3800
3600
3400
3200
3000
2800
2600
2400
2200
2000
1800
1600
1400
1200
1000
800
600
400
200
0
-200
200
190
180
170
160
150
140
130
120
110
100
90
80
70
60
50
40
30
20
10
0
-10
13C (ppm)

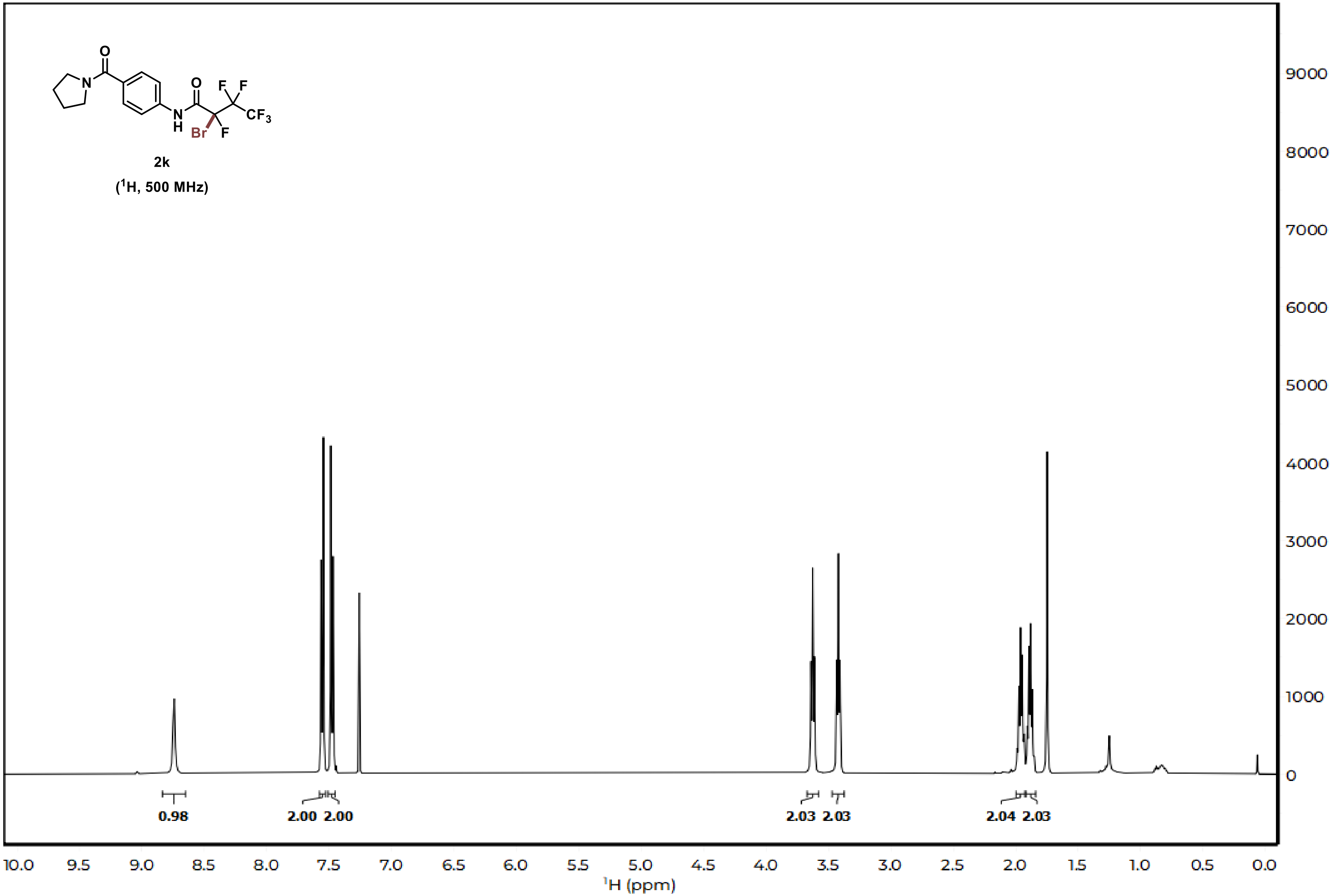

O
N
O
F F
N
H
Br F
CF3
2k
(1H, 500 MHz)
9000
8000
7000
6000
5000
4000
3000
2000
1000
0
0.98
2.00 2.00
2.03 2.03
2.04 2.03
10.0 9.5 9.0 8.5 8.0 7.5 7.0 6.5 6.0 5.5 5.0 4.5 4.0 3.5 3.0 2.5 2.0 1.5 1.0 0.5 0.0
1H (ppm)

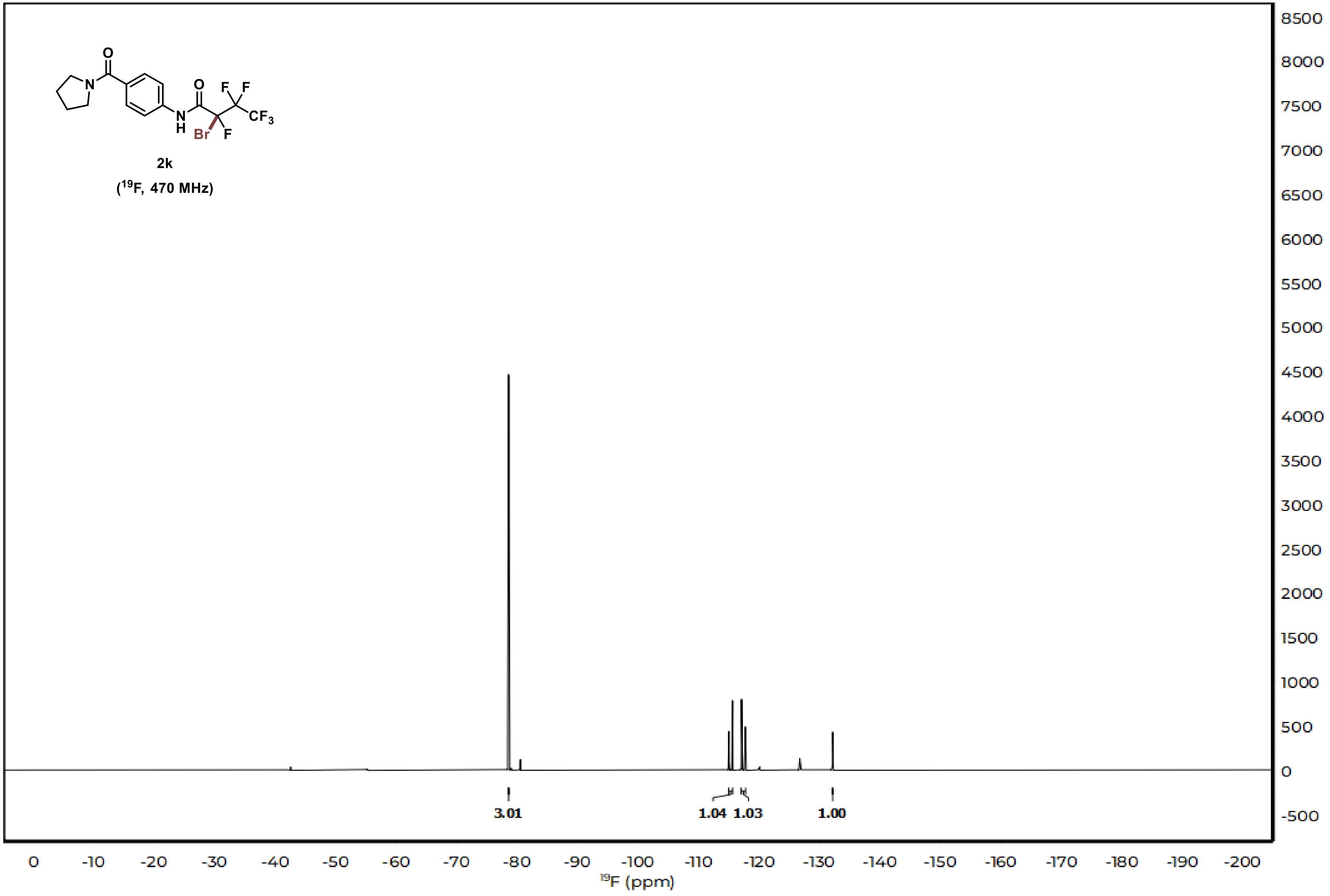
2k
(19F, 470 MHz)
3.01
1.04
1.03
1.00
8500
8000
7500
7000
6500
6000
5500
5000
4500
4000
3500
3000
2500
2000
1500
1000
500
0
-500
0
-10
-20
-30
-40
-50
-60
-70
-80
-90
-100
-110
-120
-130
-140
-150
-160
-170
-180
-190
-200
19F (ppm)

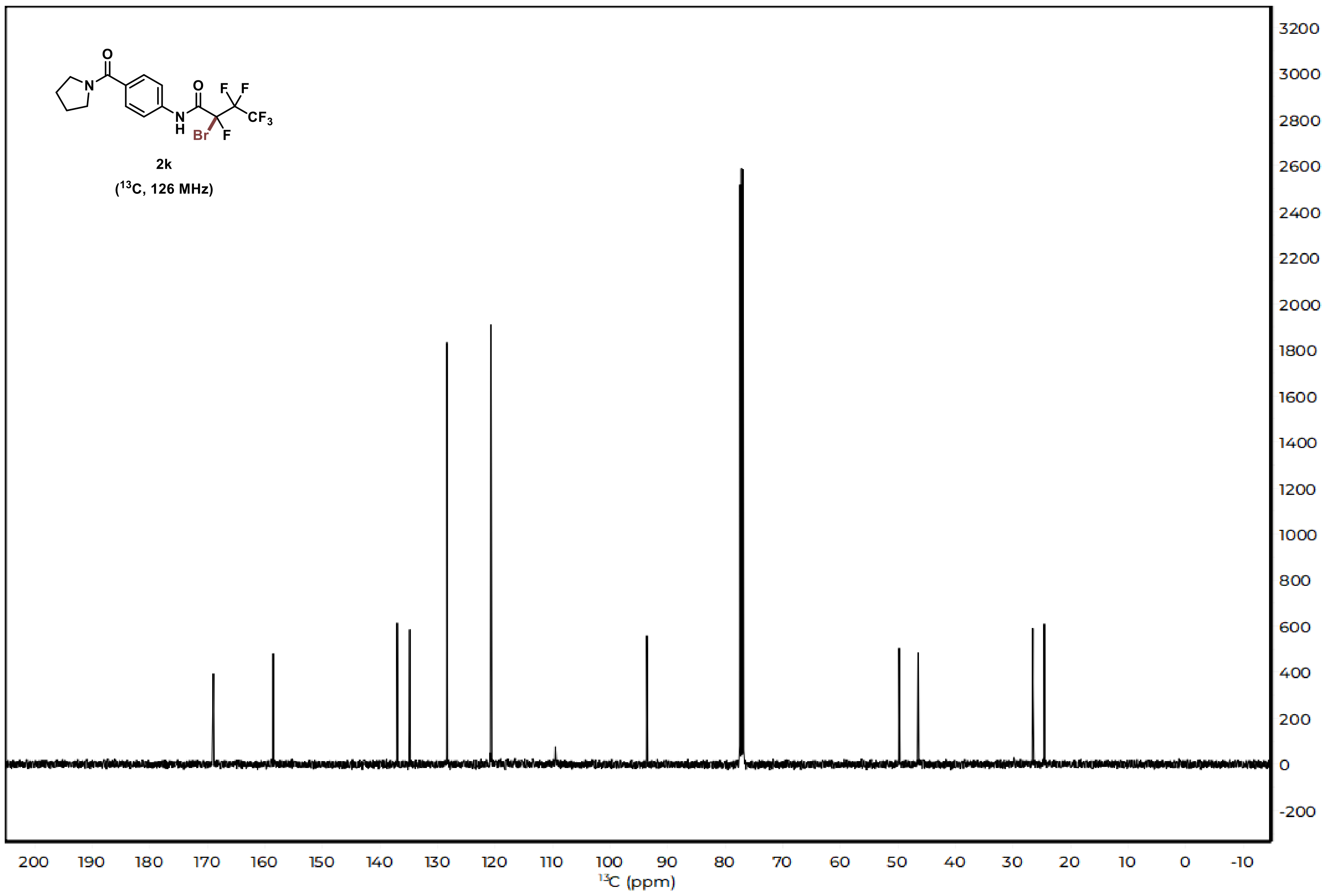

2k
(13C, 126 MHz)
3200
3000
2800
2600
2400
2200
2000
1800
1600
1400
1200
1000
800
600
400
200
0
-200
200
190
180
170
160
150
140
130
120
110
100
90
80
70
60
50
40
30
20
10
0
-10
13C (ppm)

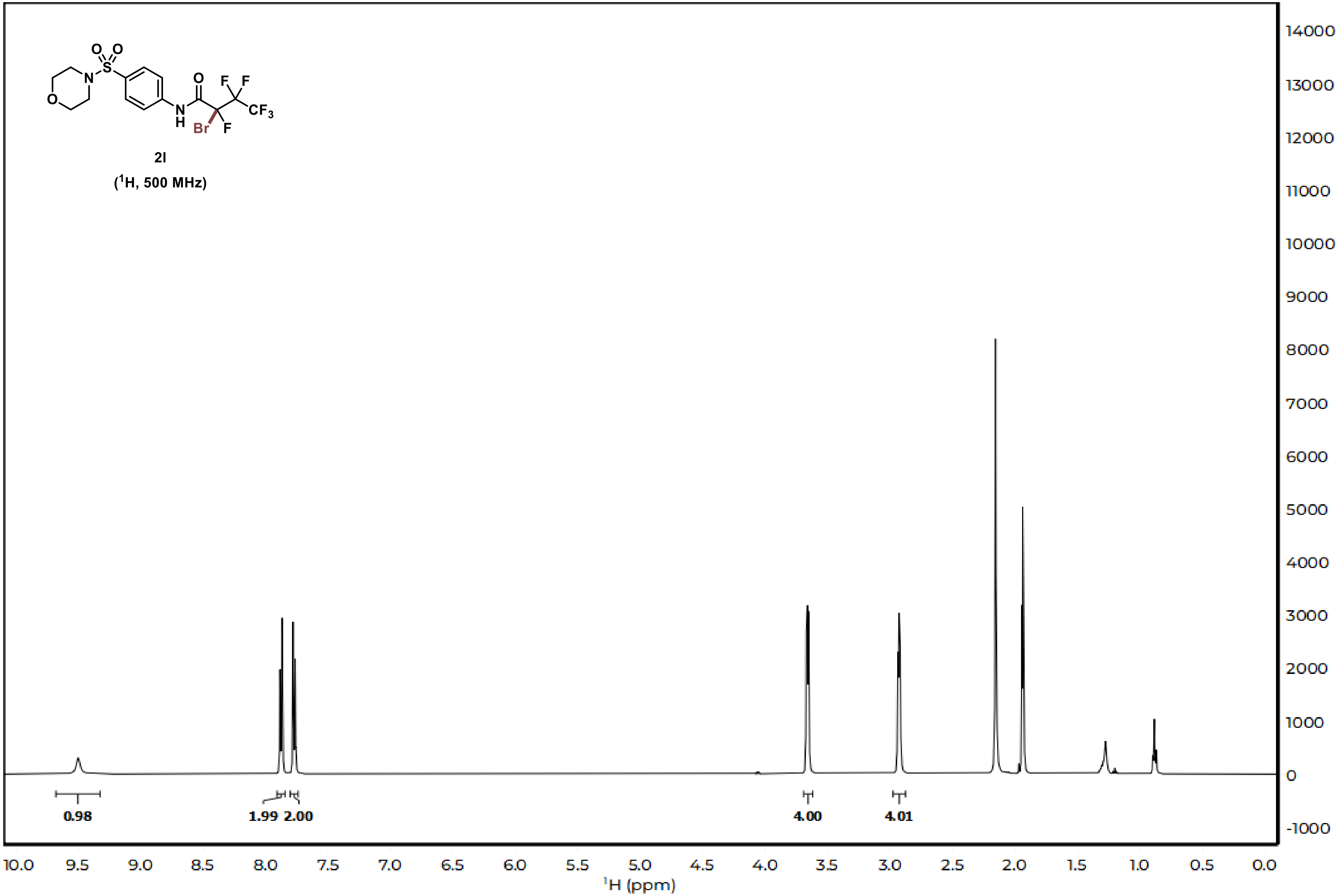

2l
(1H, 500 MHz)
14000
13000
12000
11000
10000
9000
8000
7000
6000
5000
4000
3000
2000
1000
0
-1000
0.98
1.99
2.00
4.00
4.01
10.0
9.5
9.0
8.5
8.0
7.5
7.0
6.5
6.0
5.5
5.0
4.5
4.0
3.5
3.0
2.5
2.0
1.5
1.0
0.5
0.0
1H (ppm)

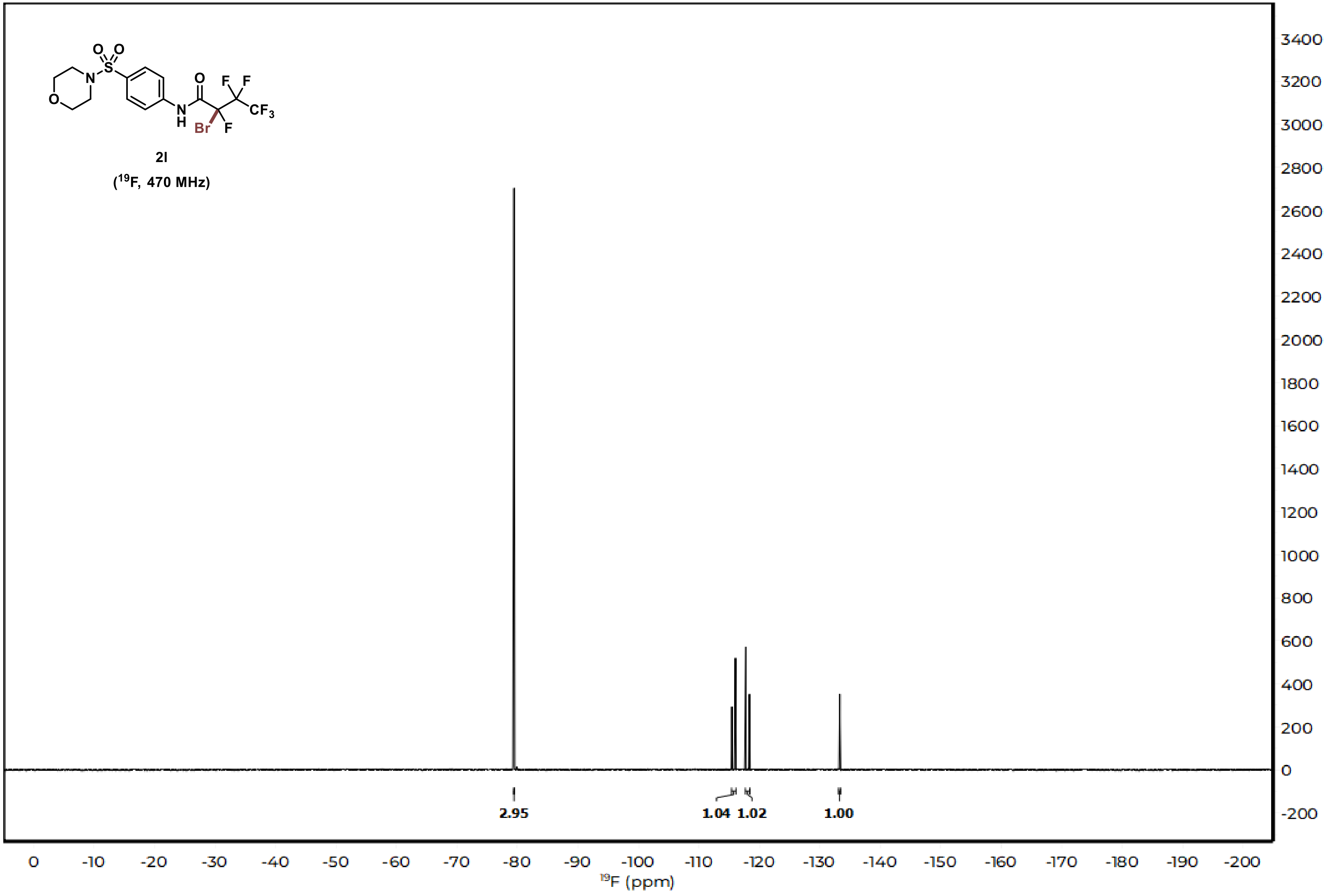

2l
(19F, 470 MHz)
3400
3200
3000
2800
2600
2400
2200
2000
1800
1600
1400
1200
1000
800
600
400
200
0
-200
2.95
1.04
1.02
1.00
0
-10
-20
-30
-40
-50
-60
-70
-80
-90
-100
-110
-120
-130
-140
-150
-160
-170
-180
-190
-200
19F (ppm)

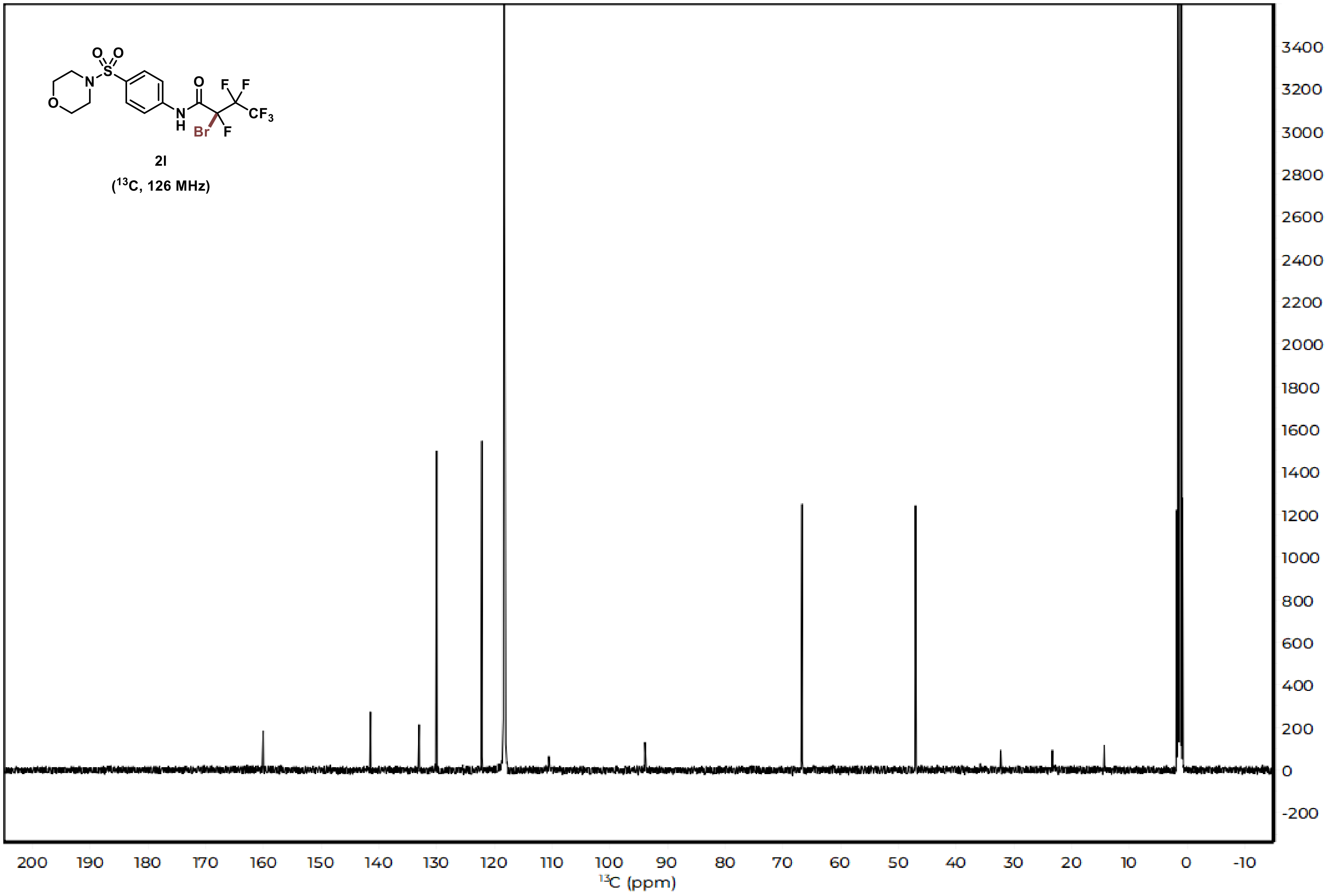
2l
(13C, 126 MHz)
3400
3200
3000
2800
2600
2400
2200
2000
1800
1600
1400
1200
1000
800
600
400
200
0
-200
200
190
180
170
160
150
140
130
120
110
100
90
80
70
60
50
40
30
20
10
0
-10
13C (ppm)

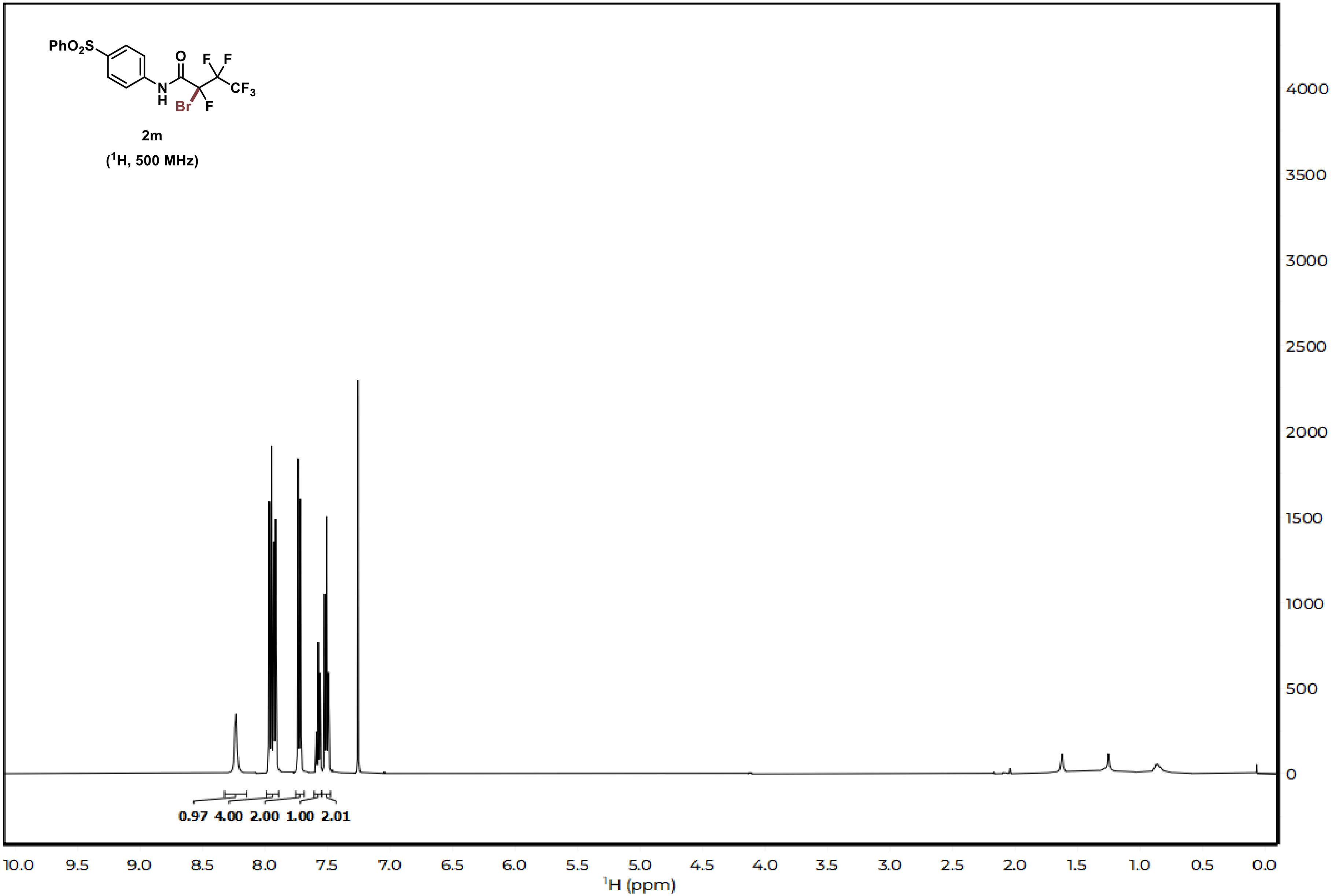
PhO2S
O
F F
N
H
Br
F
CF3
2m
(1H, 500 MHz)
0.97 4.00 2.00 1.00 2.01
4000
3500
3000
2500
2000
1500
1000
500
0
10.0 9.5 9.0 8.5 8.0 7.5 7.0 6.5 6.0 5.5 5.0 4.5 4.0 3.5 3.0 2.5 2.0 1.5 1.0 0.5 0.0
1H (ppm)

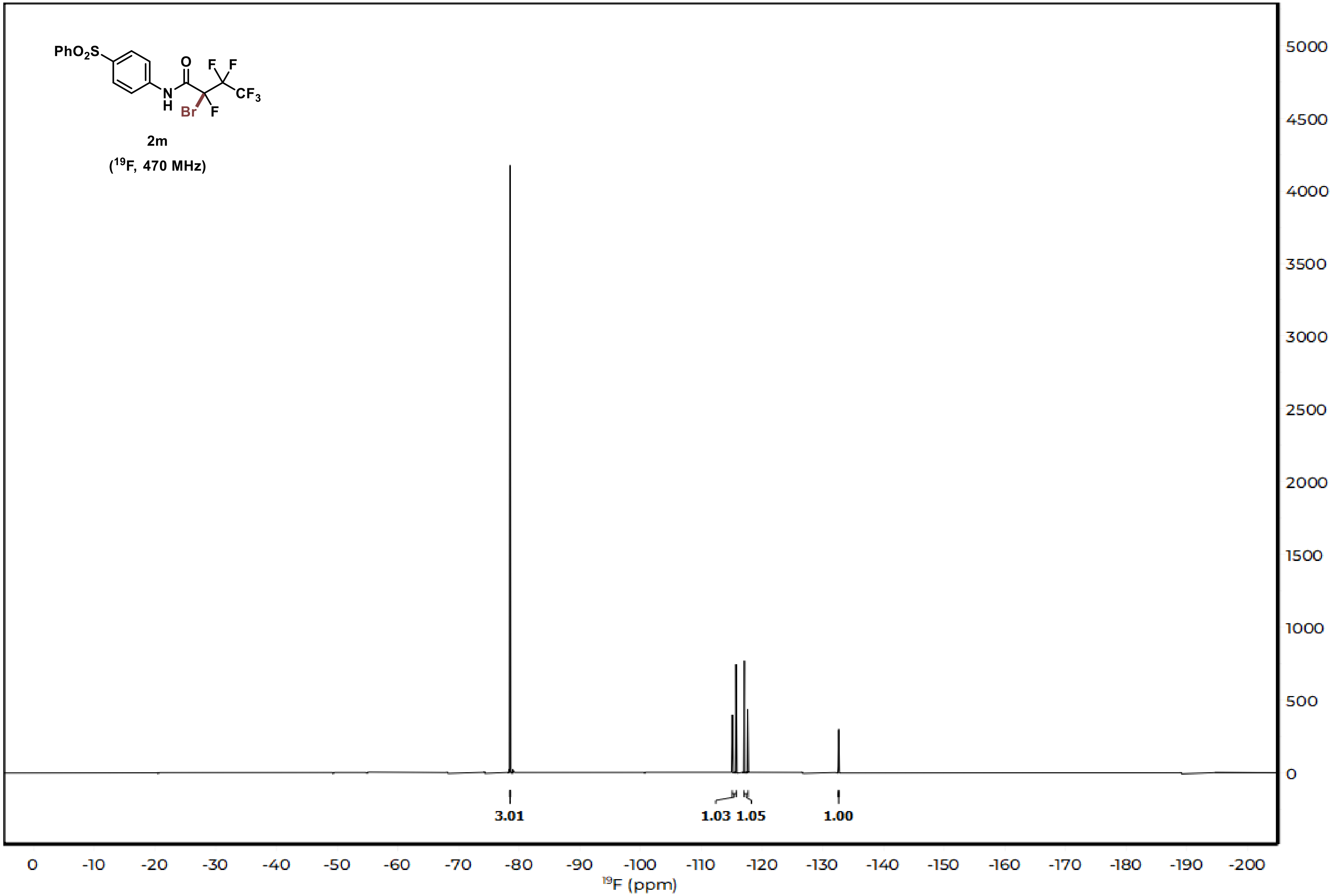

PhO2S
O
F F
N
H
CF3
Br
F
2m
(19F, 470 MHz)
5000
4500
4000
3500
3000
2500
2000
1500
1000
500
0
3.01
1.03 1.05
1.00
0 -10 -20 -30 -40 -50 -60 -70 -80 -90 -100 -110 -120 -130 -140 -150 -160 -170 -180 -190 -200
19F (ppm)

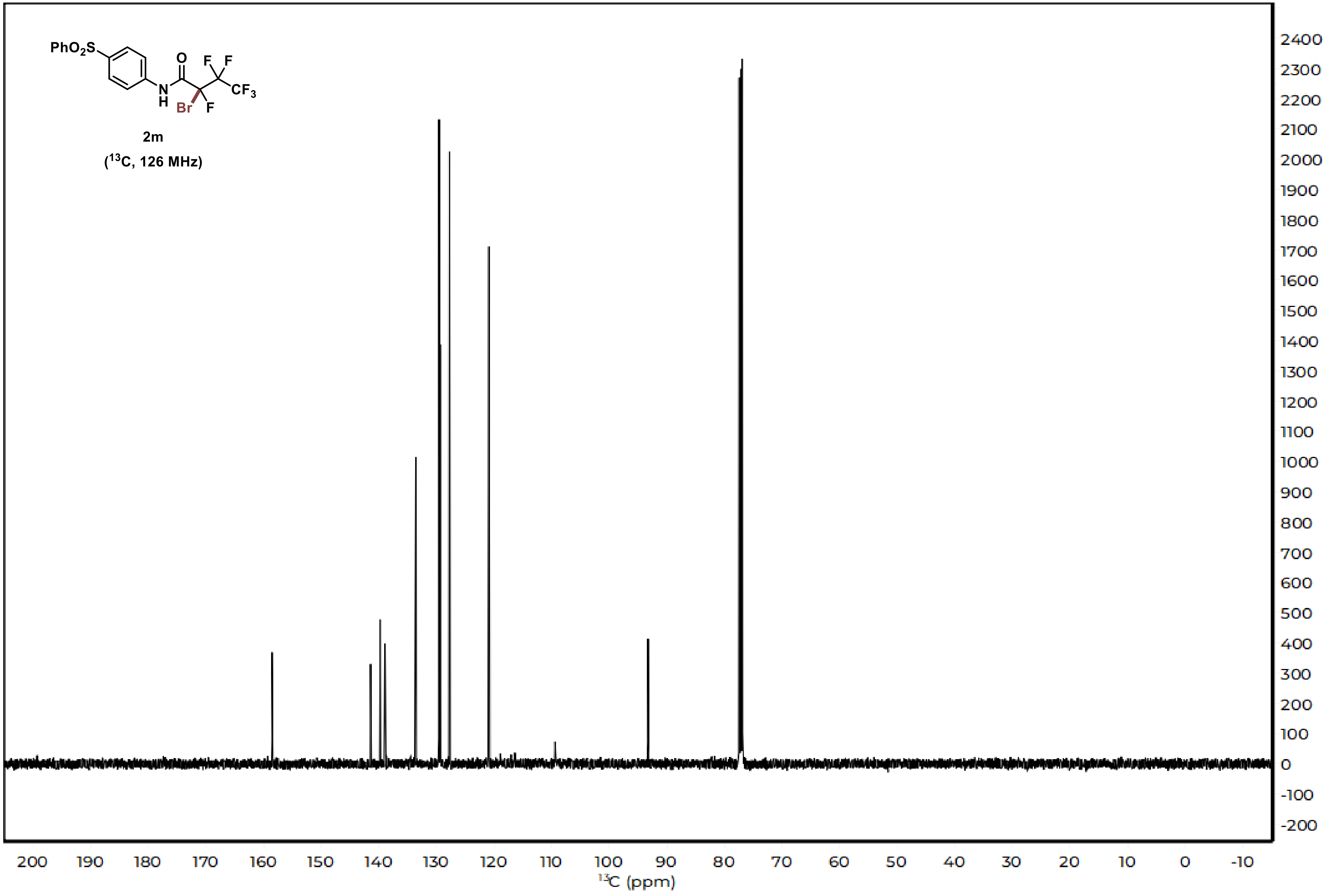
PhO2S
O
F
F
N
H
CF3
Br
F
2m
(13C, 126 MHz)
2400
2300
2200
2100
2000
1900
1800
1700
1600
1500
1400
1300
1200
1100
1000
900
800
700
600
500
400
300
200
100
0
-100
-200
200
190
180
170
160
150
140
130
120
110
100
90
80
70
60
50
40
30
20
10
0
-10
13C (ppm)

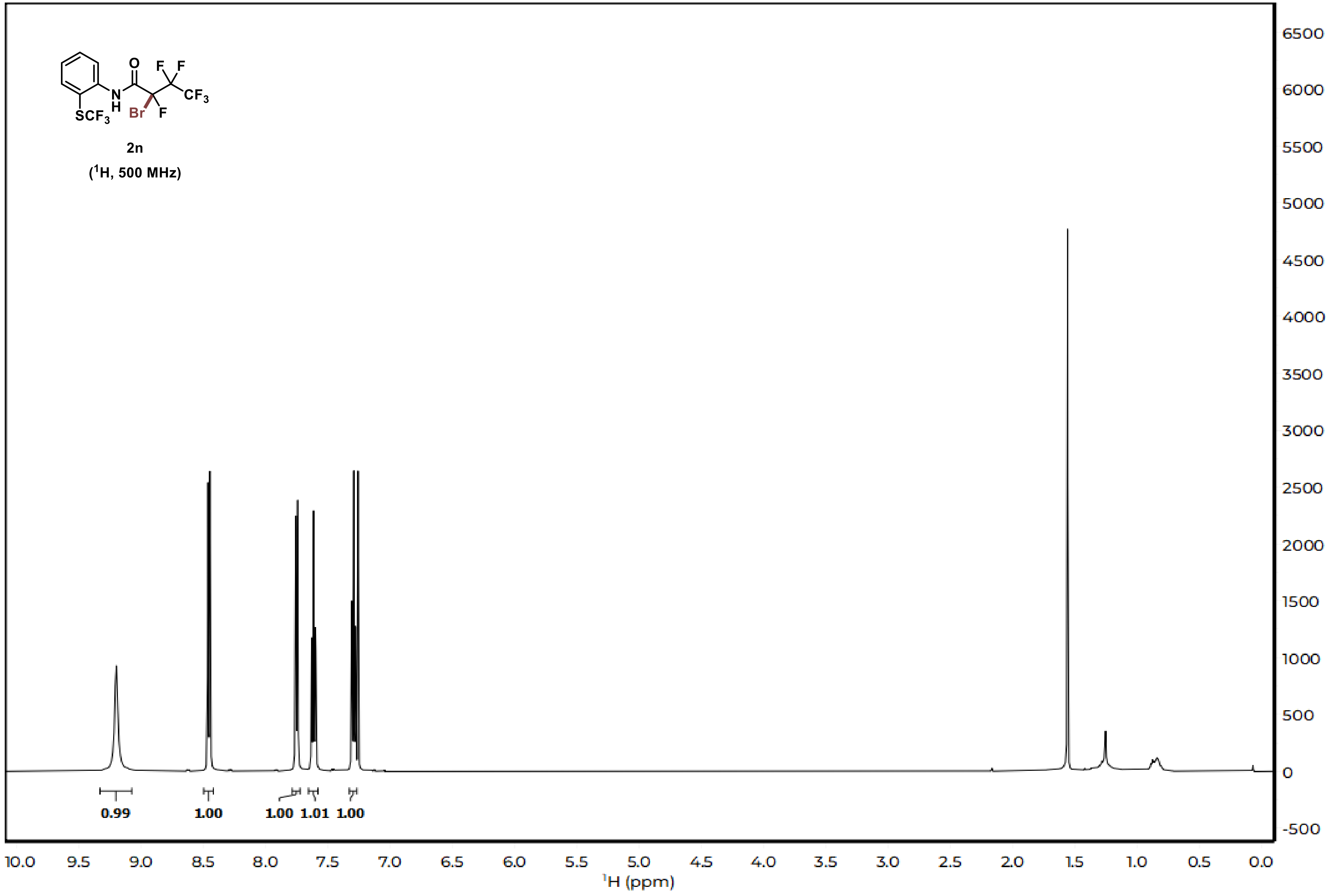

O
F
F
N
H
CF3
SCF3
Br
F
2n
(1H, 500 MHz)
6500
6000
5500
5000
4500
4000
3500
3000
2500
2000
1500
1000
500
0
-500
0.99
1.00
1.00
1.01
1.00
10.0
9.5
9.0
8.5
8.0
7.5
7.0
6.5
6.0
5.5
5.0
4.5
4.0
3.5
3.0
2.5
2.0
1.5
1.0
0.5
0.0
1H (ppm)

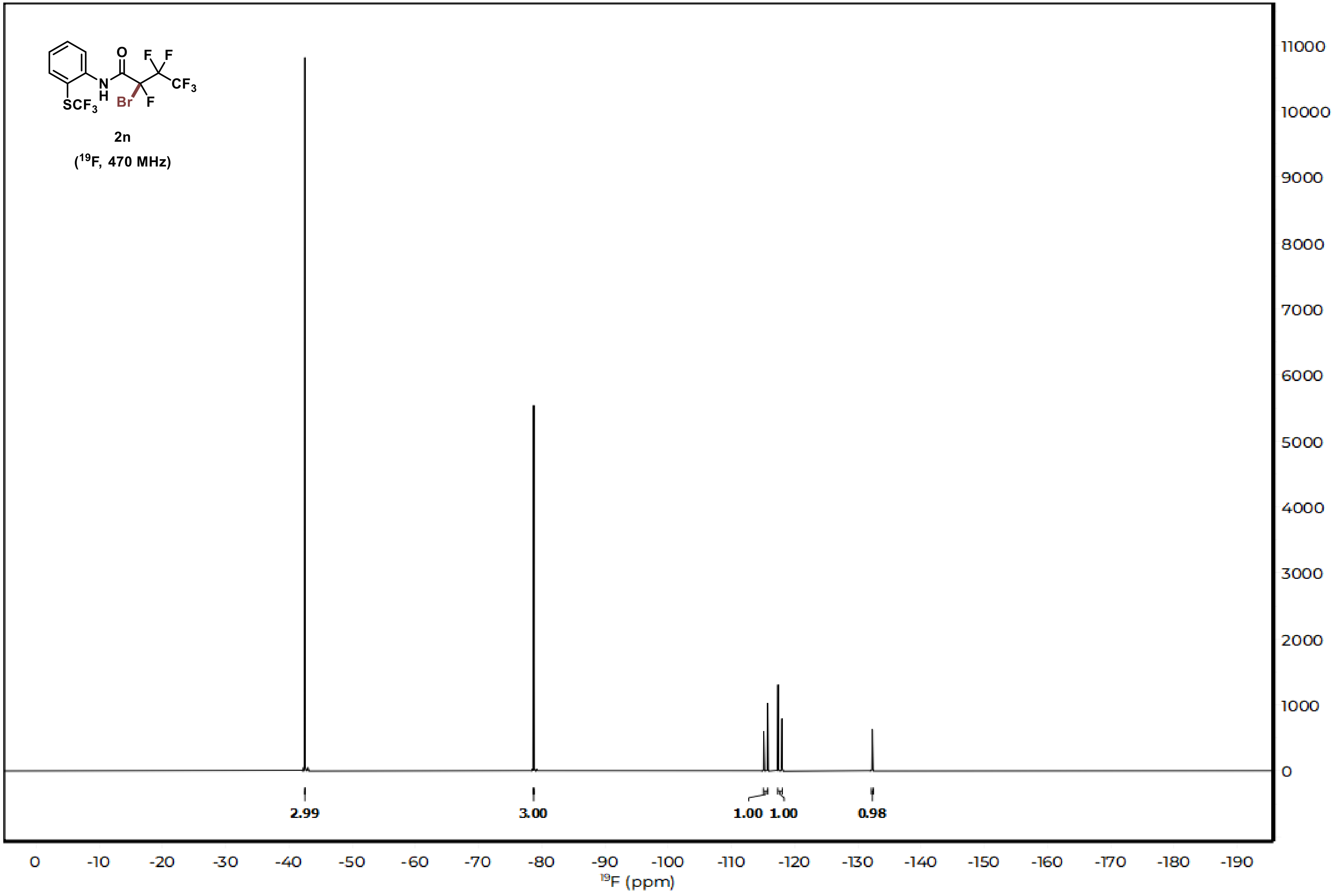
O
F F
N
H
CF3
SCF3
Br
F
2n
(19F, 470 MHz)
11000
10000
9000
8000
7000
6000
5000
4000
3000
2000
1000
0
2.99
3.00
1.00
1.00
0.98
0
-10
-20
-30
-40
-50
-60
-70
-80
-90
-100
-110
-120
-130
-140
-150
-160
-170
-180
-190
19F (ppm)

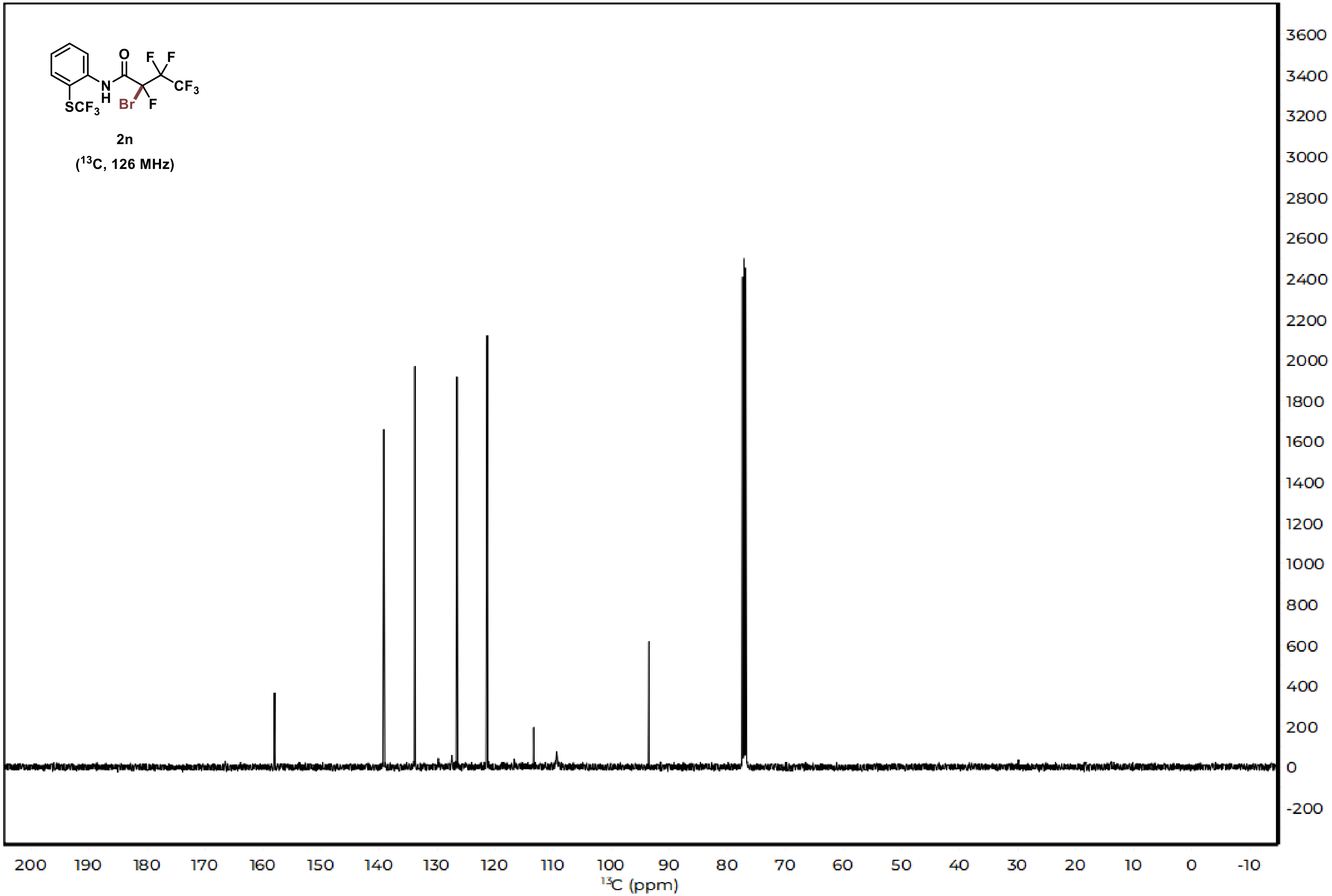
O
F F
N
H
CF3
SCF3
Br
F
2n
(13C, 126 MHz)
3600
3400
3200
3000
2800
2600
2400
2200
2000
1800
1600
1400
1200
1000
800
600
400
200
0
-200
200 190 180 170 160 150 140 130 120 110 100 90 80 70 60 50 40 30 20 10 0 -10
13C (ppm)

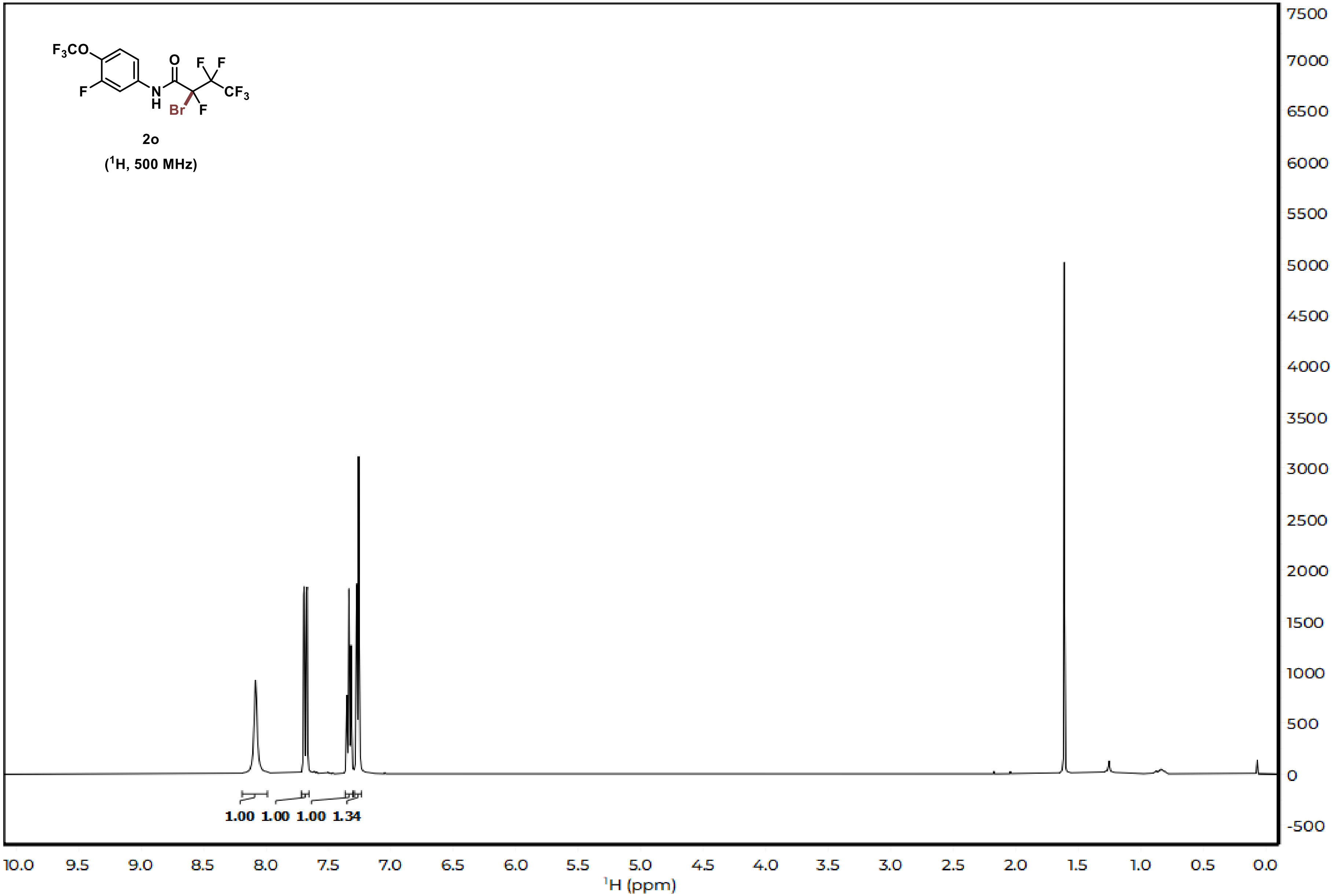
F3CO
F
N
H
O
F F
CF3
Br
F
2o
(1H, 500 MHz)
1.00 1.00 1.00 1.34
7500
7000
6500
6000
5500
5000
4500
4000
3500
3000
2500
2000
1500
1000
500
0
-500
10.0 9.5 9.0 8.5 8.0 7.5 7.0 6.5 6.0 5.5 5.0 4.5 4.0 3.5 3.0 2.5 2.0 1.5 1.0 0.5 0.0
1H (ppm)

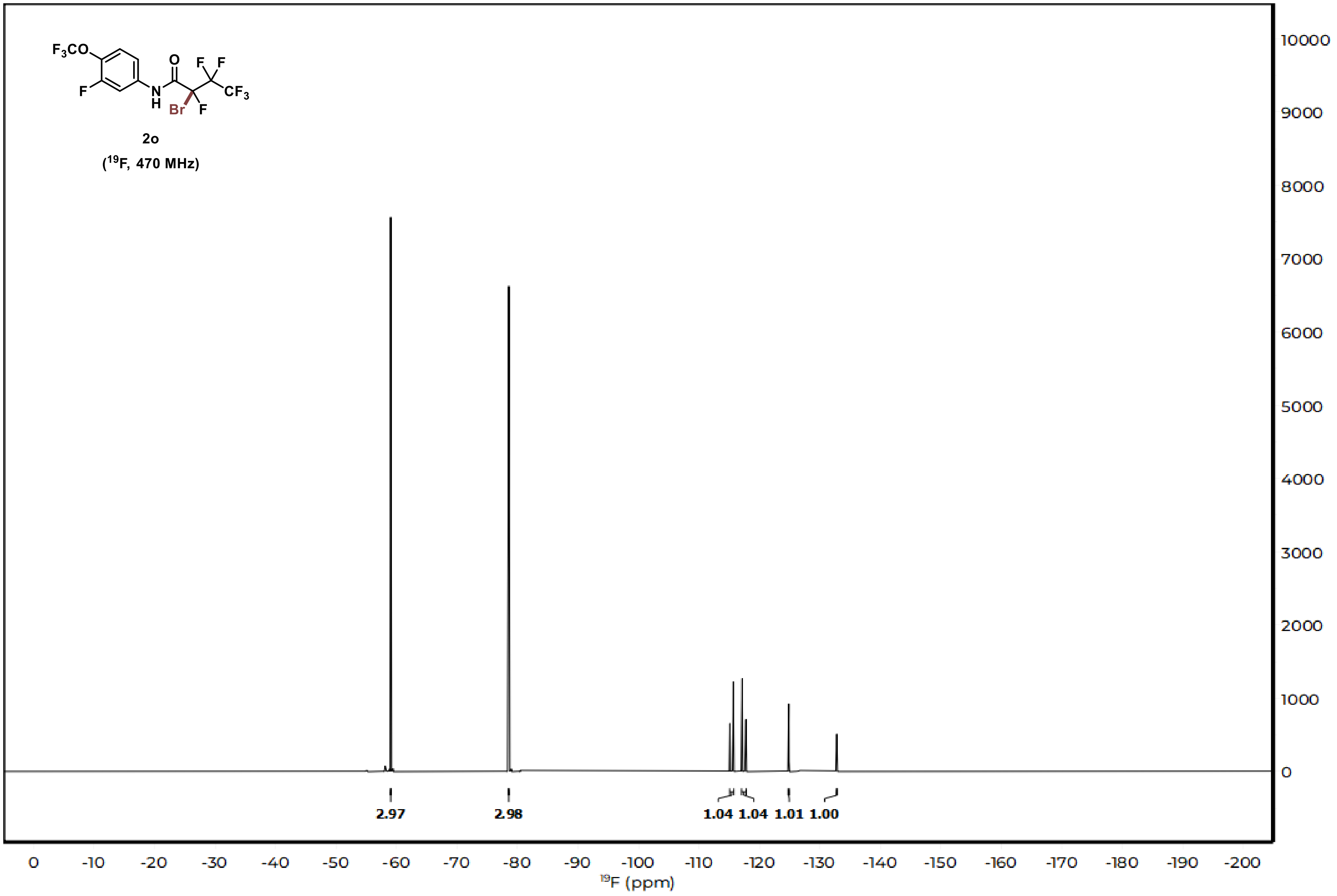

F3CO
F
N
H
O
F F
CF3
Br F
2o
(19F, 470 MHz)
10000
9000
8000
7000
6000
5000
4000
3000
2000
1000
0
2.97
2.98
1.04 1.04 1.01 1.00
0 -10 -20 -30 -40 -50 -60 -70 -80 -90 -100 -110 -120 -130 -140 -150 -160 -170 -180 -190 -200
19F (ppm)

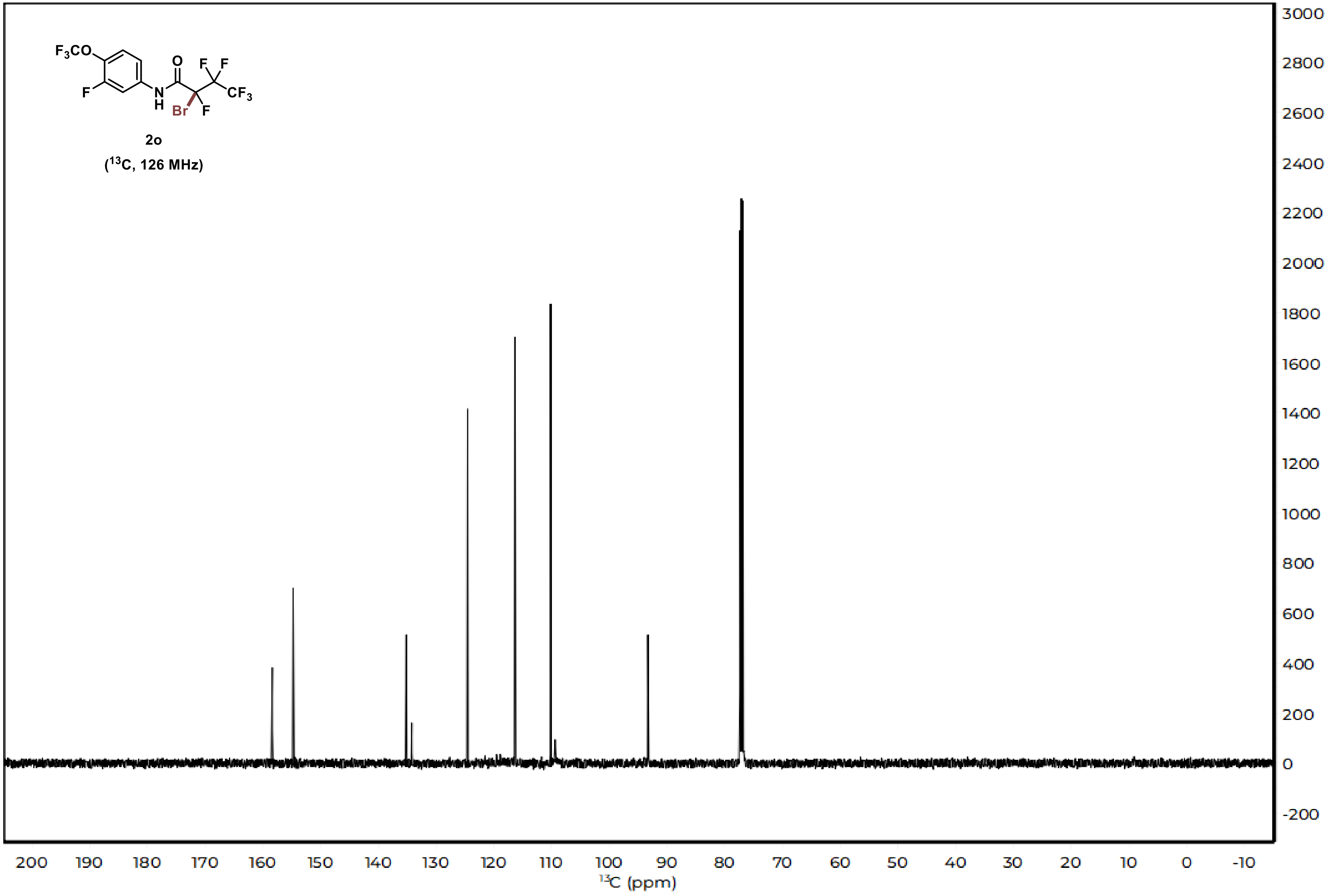

F_3CO
F
O
F F
N
H
Br
F
CF_3
2o
(^{13}C, 126 MHz)
3000
2800
2600
2400
2200
2000
1800
1600
1400
1200
1000
800
600
400
200
0
-200
200 190 180 170 160 150 140 130 120 110 100 90 80 70 60 50 40 30 20 10 0 -10
^{13}C (ppm)

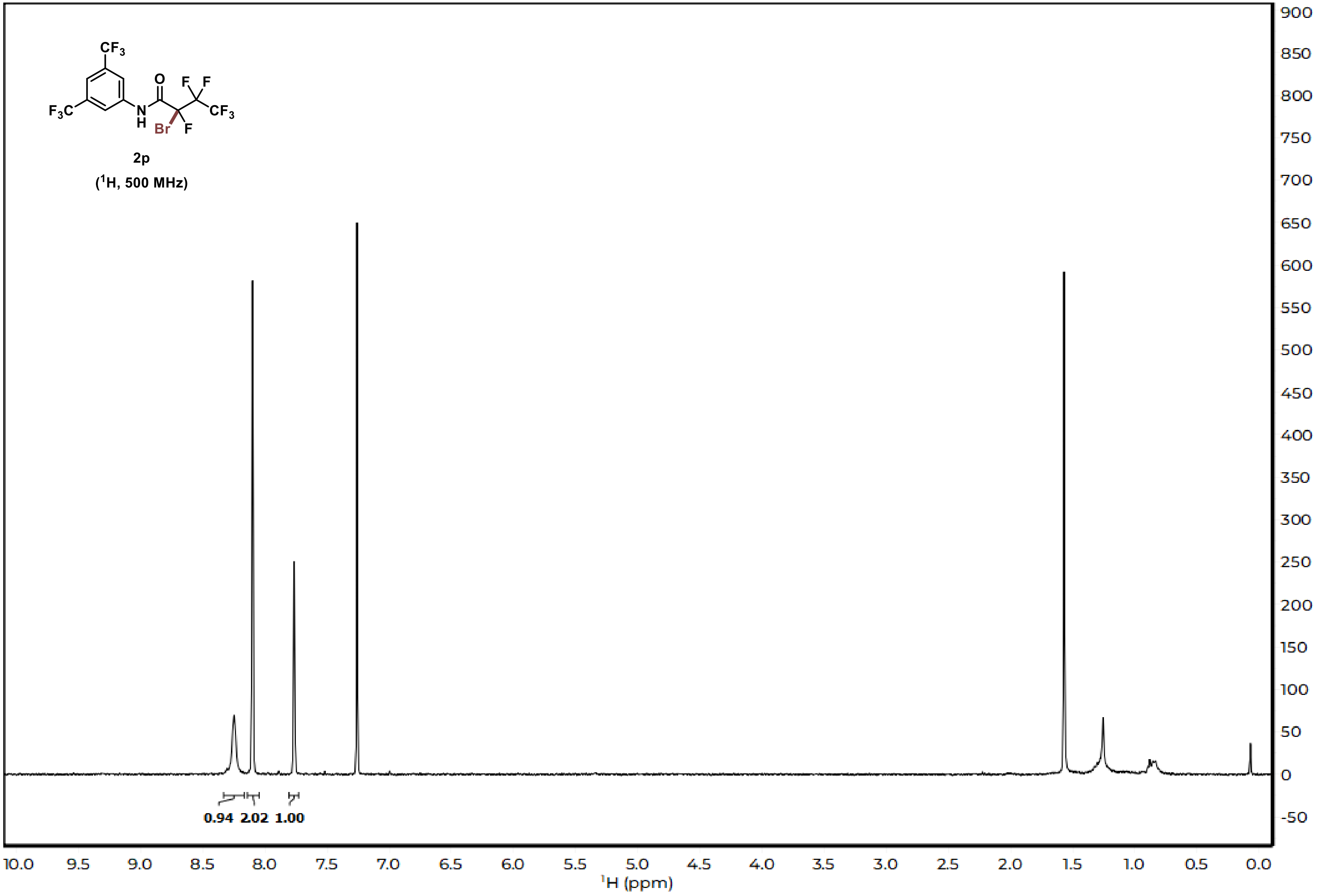

CF3
O
F F
F3C
N
H
CF3
Br
F
2p
(1H, 500 MHz)
0.94 2.02 1.00
1H (ppm)

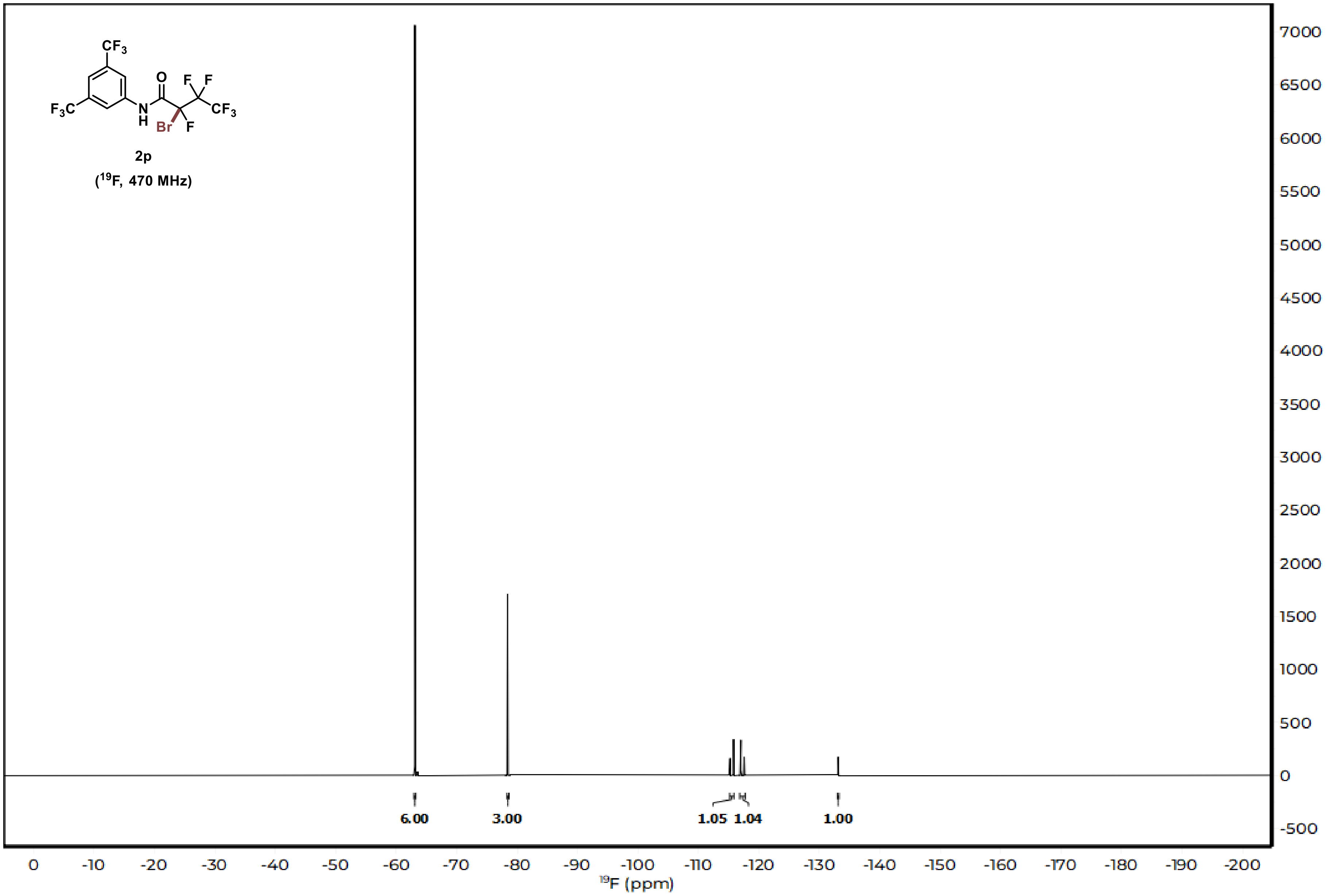
CF3
O
F F
F3C
N
H
CF3
Br
F
2p
(19F, 470 MHz)
6.00
3.00
1.05 1.04
1.00
7000
6500
6000
5500
5000
4500
4000
3500
3000
2500
2000
1500
1000
500
0
-500
0 -10 -20 -30 -40 -50 -60 -70 -80 -90 -100 -110 -120 -130 -140 -150 -160 -170 -180 -190 -200
19F (ppm)

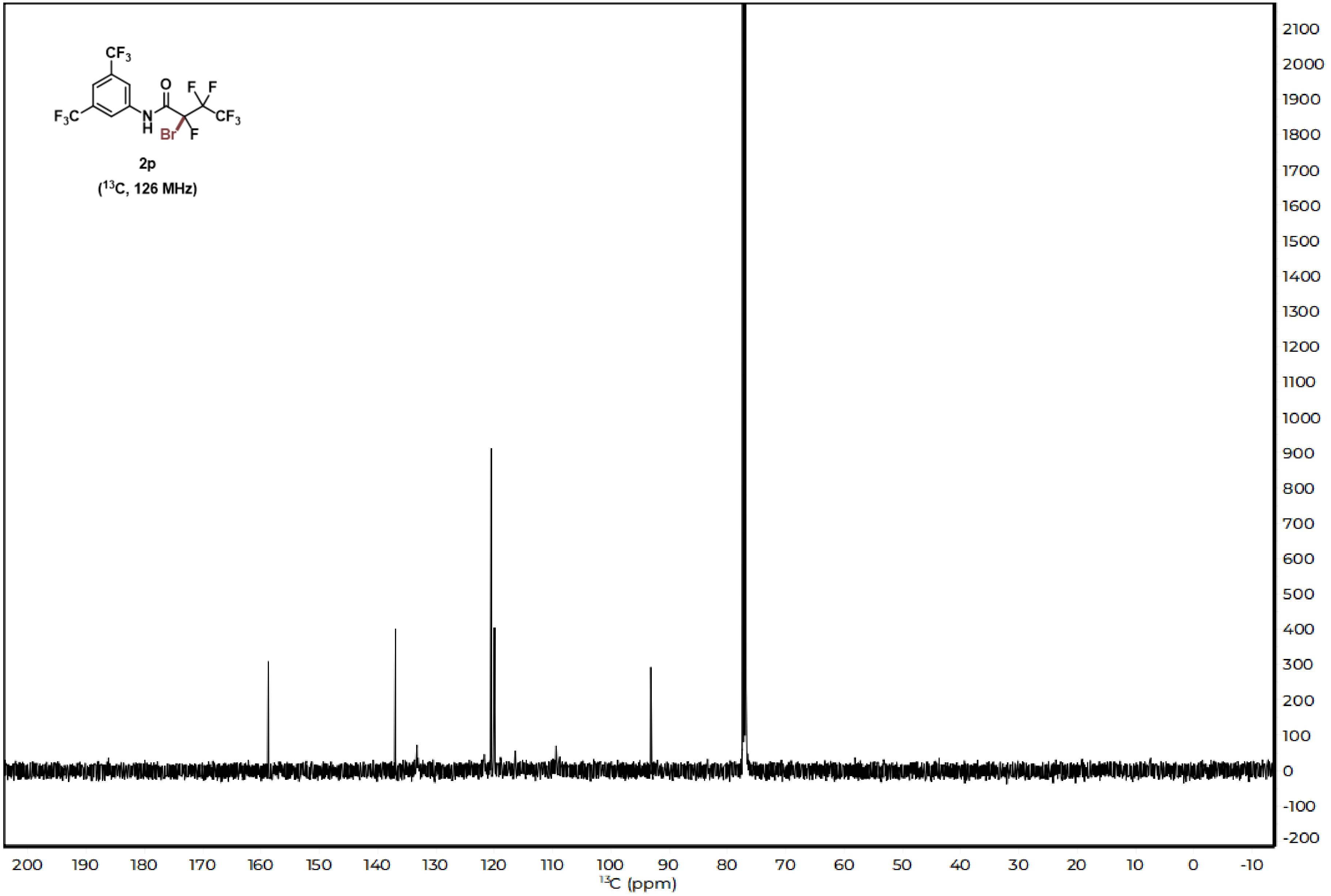
CF3
O
F F
F3C
N
H
Br
F
CF3
2p
(13C, 126 MHz)
2100
2000
1900
1800
1700
1600
1500
1400
1300
1200
1100
1000
900
800
700
600
500
400
300
200
100
0
-100
-200
200 190 180 170 160 150 140 130 120 110 100 90 80 70 60 50 40 30 20 10 0 -10
13C (ppm)

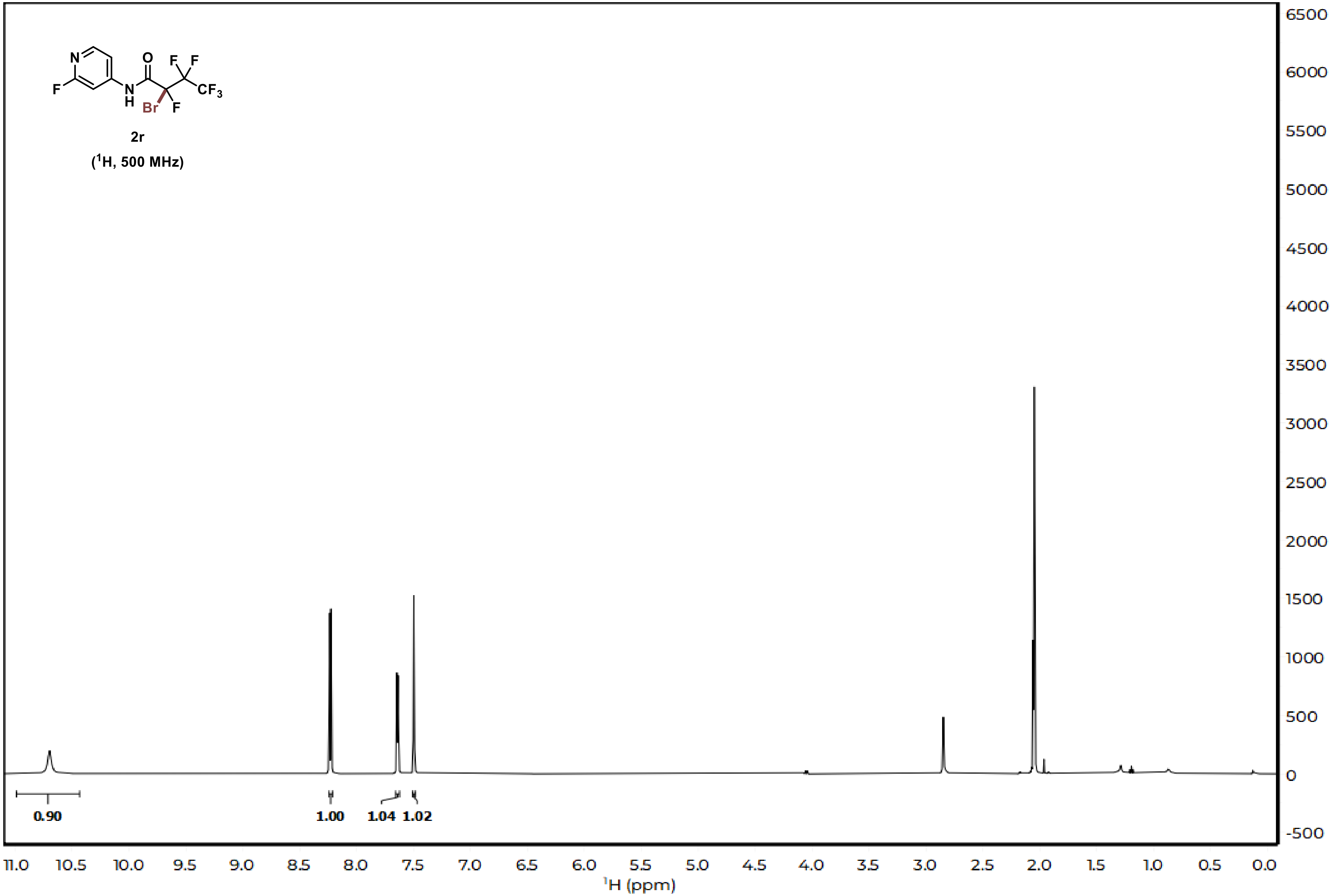

2r
(1H, 500 MHz)
6500
6000
5500
5000
4500
4000
3500
3000
2500
2000
1500
1000
500
0
-500
0.90
1.00
1.04
1.02
11.0
10.5
10.0
9.5
9.0
8.5
8.0
7.5
7.0
6.5
6.0
5.5
5.0
4.5
4.0
3.5
3.0
2.5
2.0
1.5
1.0
0.5
0.0
1H (ppm)

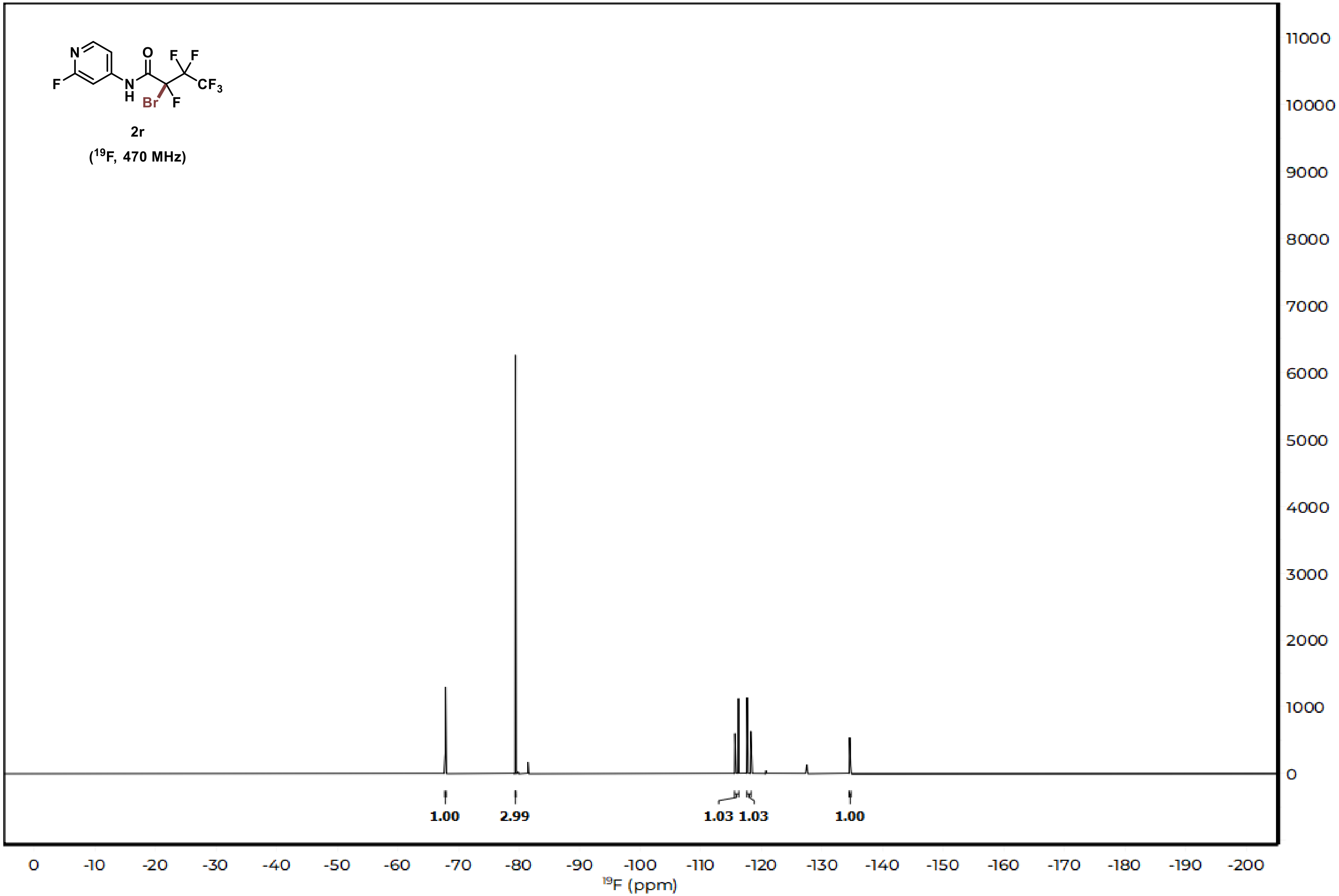
2r
(19F, 470 MHz)
1.00
2.99
1.03 1.03
1.00
19F (ppm)

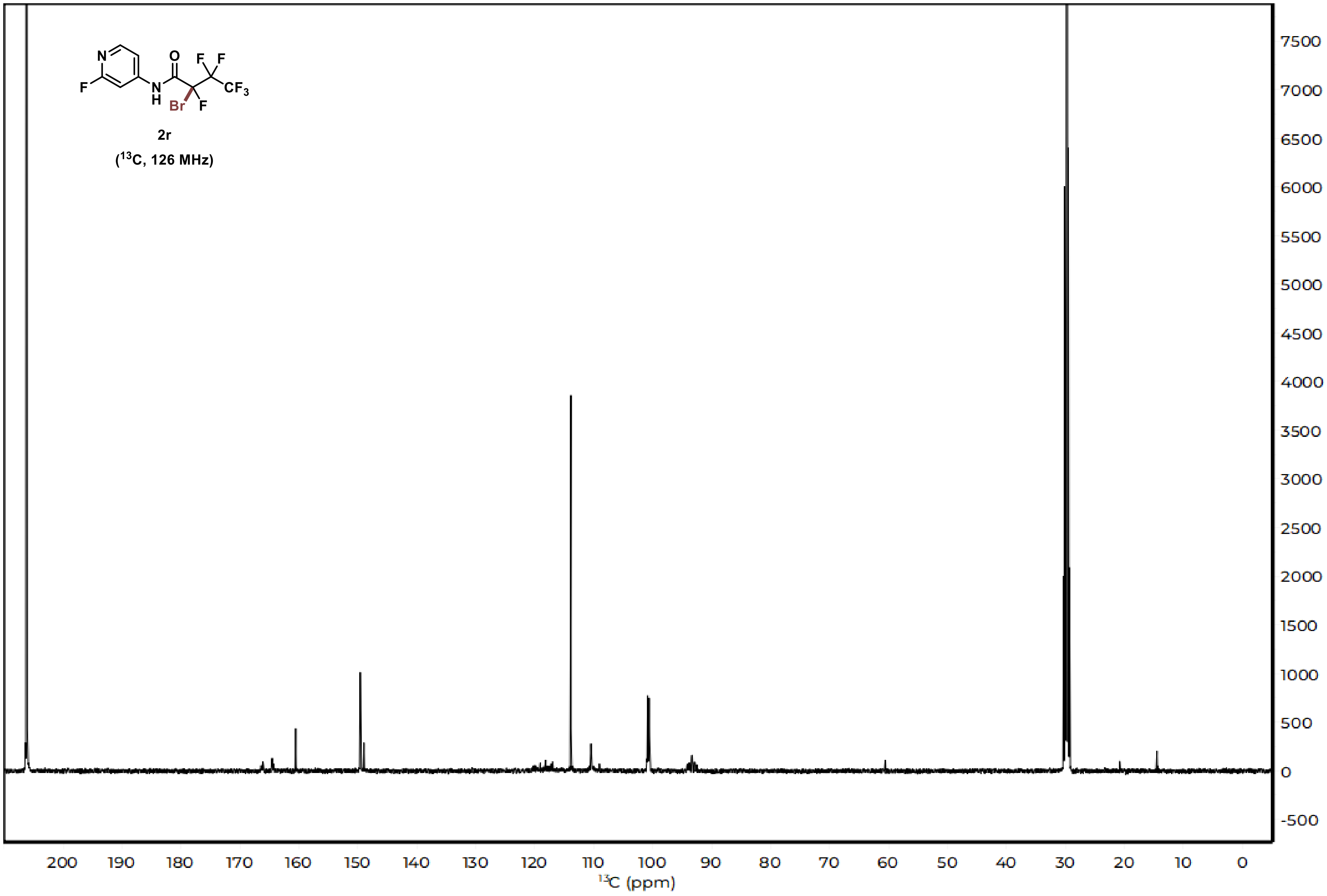
2r
(^{13}C, 126 MHz)
N
F
O
N
H
Br
F
F
F
CF3
7500
7000
6500
6000
5500
5000
4500
4000
3500
3000
2500
2000
1500
1000
500
0
-500
200 190 180 170 160 150 140 130 120 110 100 90 80 70 60 50 40 30 20 10 0
13C (ppm)

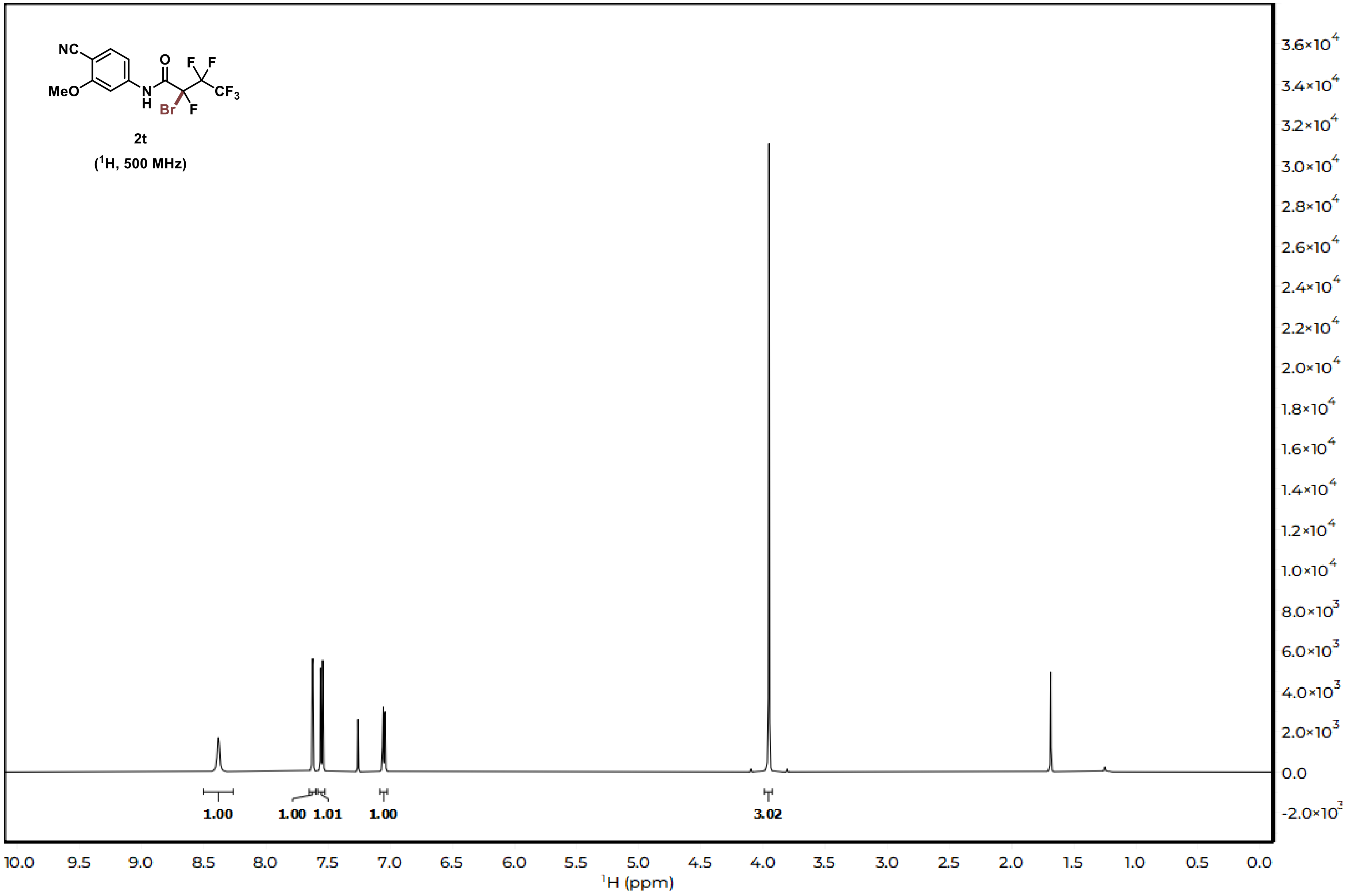
NC
MeO
O
F
F
N
H
CF3
Br
F
2t
(1H, 500 MHz)
1.00
1.00
1.01
1.00
3.02
1H (ppm)

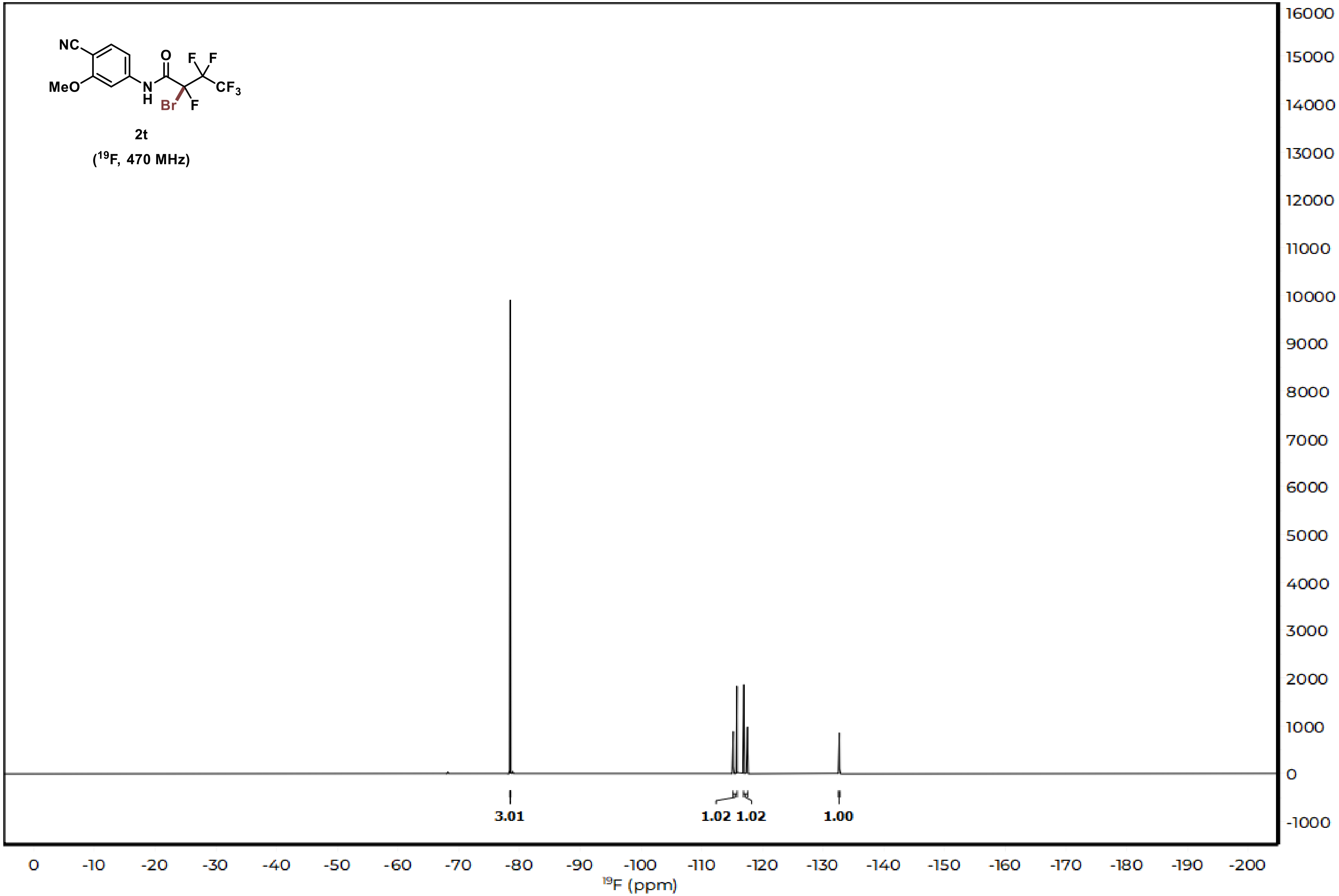

NC
MeO
N
H
O
Br
F
F F
CF3
2t
(19F, 470 MHz)
3.01
1.02 1.02
1.00
16000
15000
14000
13000
12000
11000
10000
9000
8000
7000
6000
5000
4000
3000
2000
1000
0
-1000
0
-10
-20
-30
-40
-50
-60
-70
-80
-90
-100
-110
-120
-130
-140
-150
-160
-170
-180
-190
-200
19F (ppm)

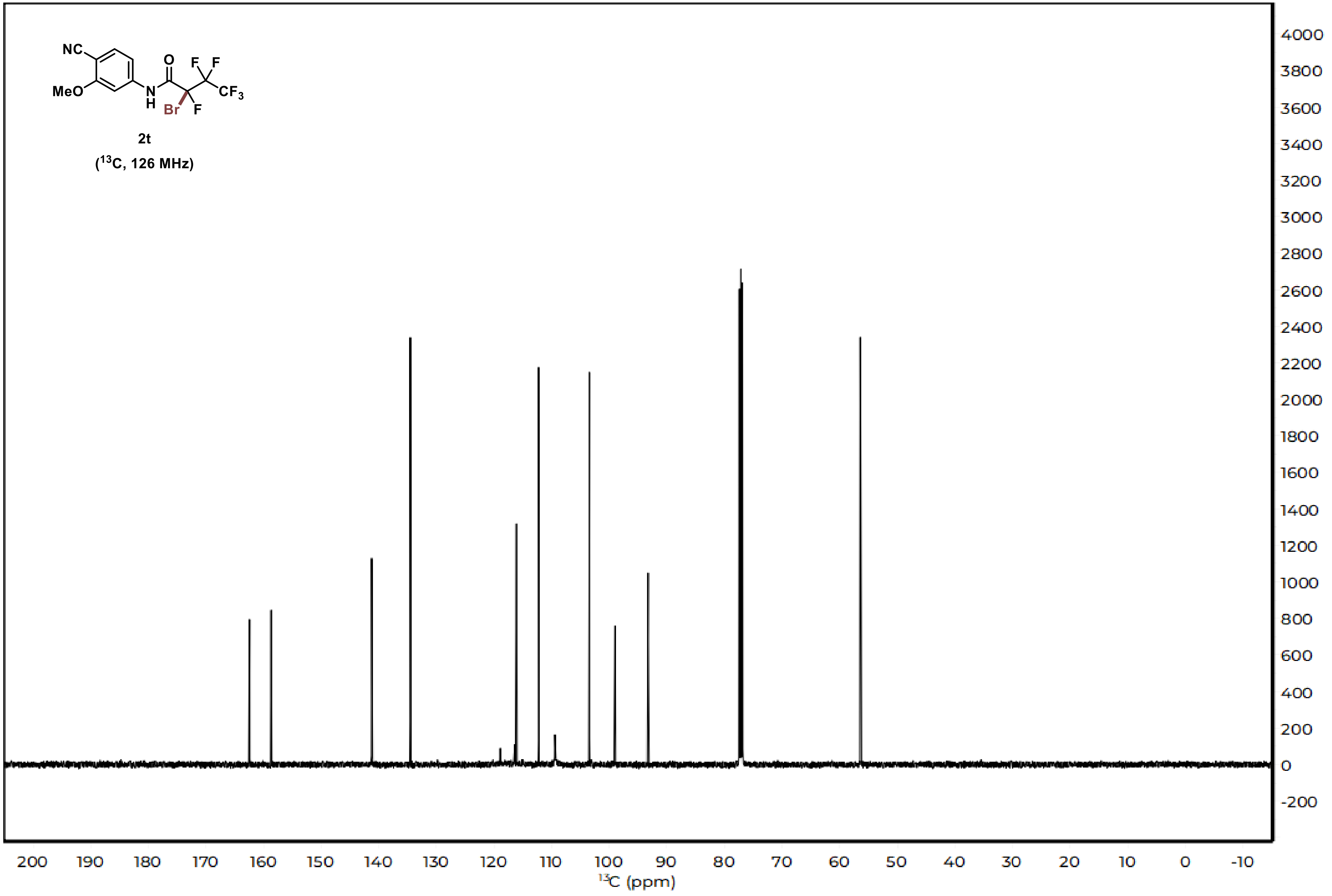
NC
MeO
N
H
O
F
F
CF3
Br
F
2t
(13C, 126 MHz)
4000
3800
3600
3400
3200
3000
2800
2600
2400
2200
2000
1800
1600
1400
1200
1000
800
600
400
200
0
-200
200
190
180
170
160
150
140
130
120
110
100
90
80
70
60
50
40
30
20
10
0
-10
13C (ppm)

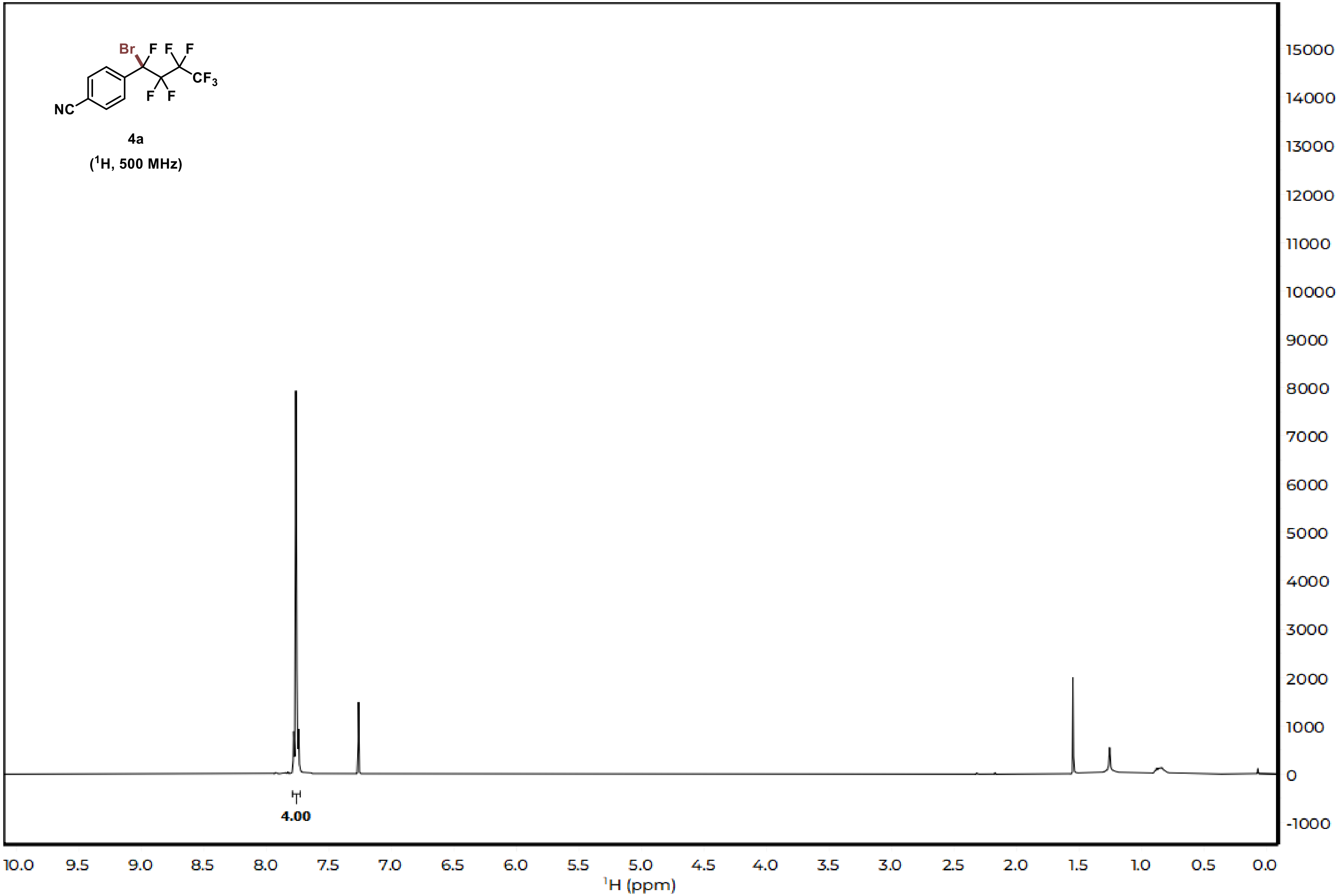

Br
F
F
F
CF3
F
F
NC
4a
(1H, 500 MHz)
15000
14000
13000
12000
11000
10000
9000
8000
7000
6000
5000
4000
3000
2000
1000
0
-1000
4.00
10.0
9.5
9.0
8.5
8.0
7.5
7.0
6.5
6.0
5.5
5.0
4.5
4.0
3.5
3.0
2.5
2.0
1.5
1.0
0.5
0.0
1H (ppm)

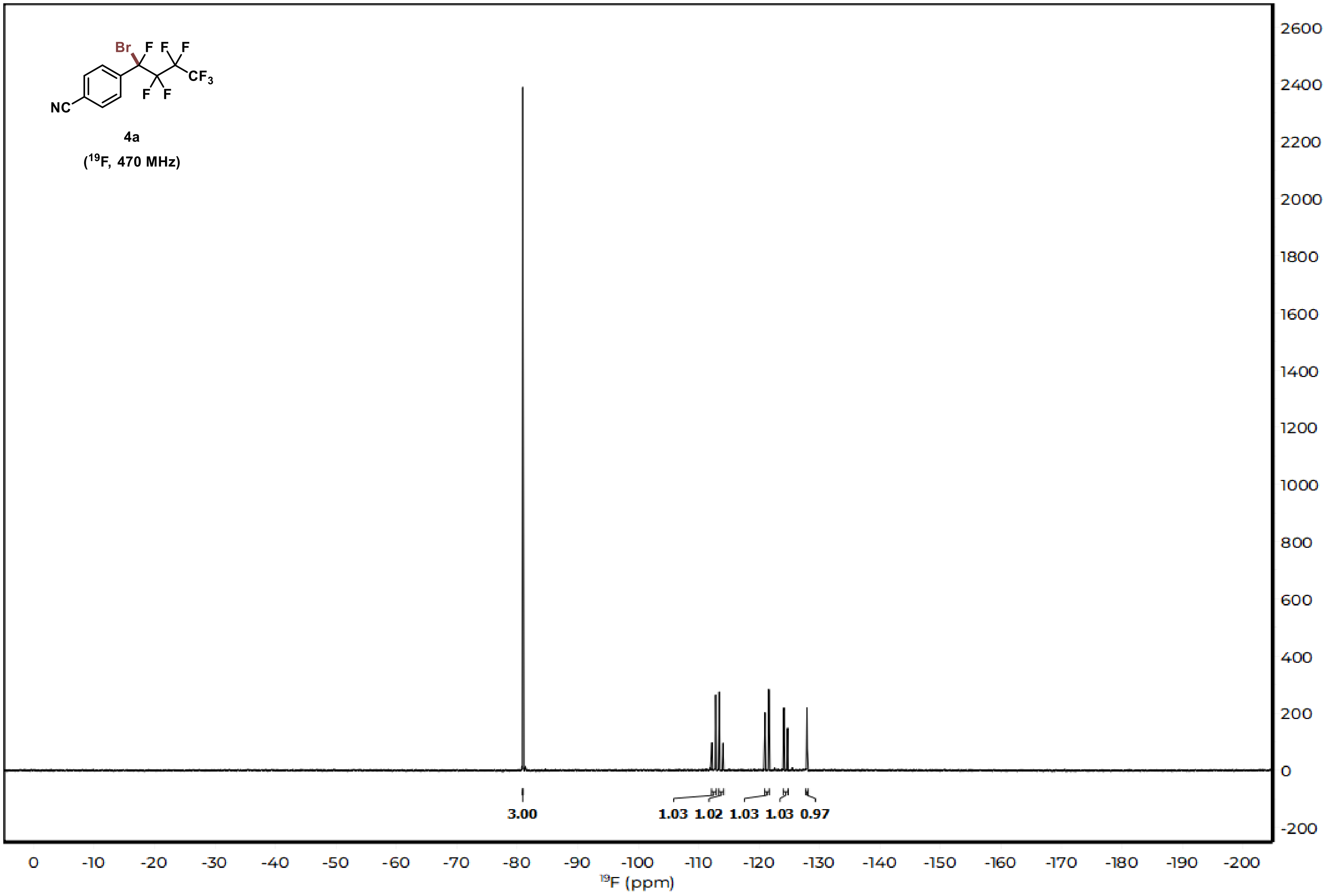
Br
F F F
CF3
F F
NC
4a
(19F, 470 MHz)
3.00
1.03 1.02 1.03 1.03 0.97
19F (ppm)

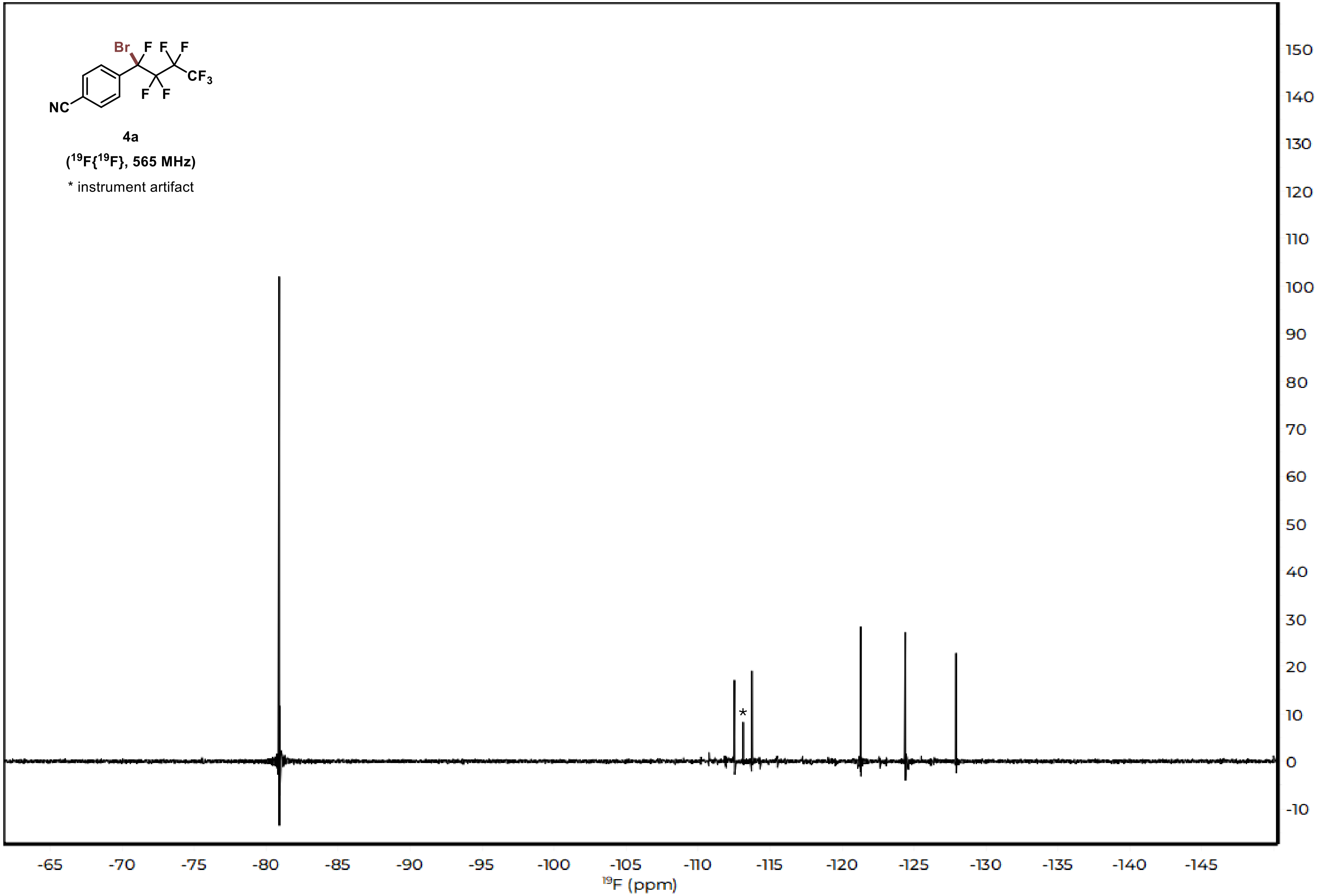

Br
F
F
F
CF3
F
F
NC
4a
(19F{19F}, 565 MHz)
* instrument artifact
*
150
140
130
120
110
100
90
80
70
60
50
40
30
20
10
0
-10
-65
-70
-75
-80
-85
-90
-95
-100
-105
-110
-115
-120
-125
-130
-135
-140
-145
19F (ppm)

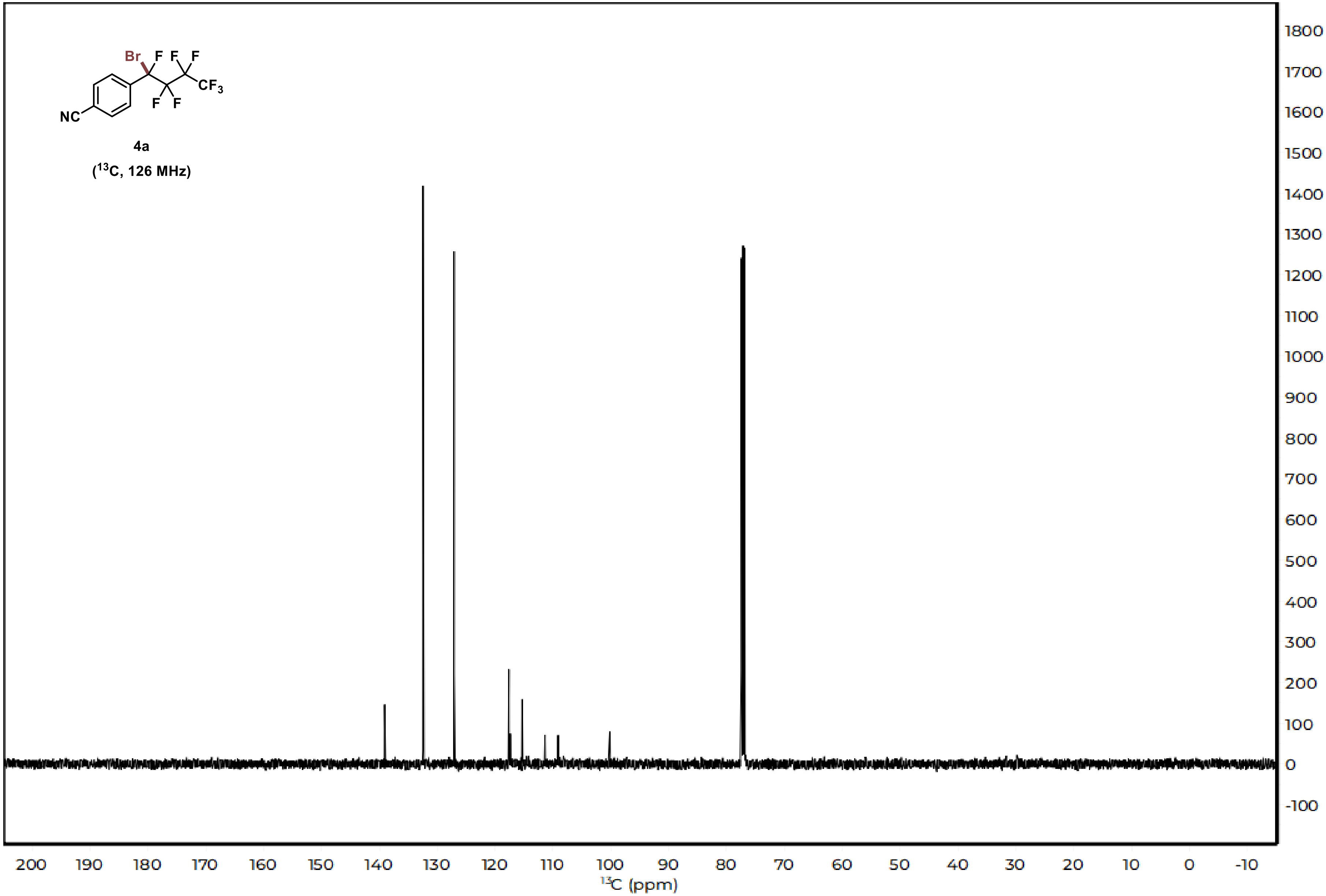

Br F F F
CF3
NC
F F
4a
(13C, 126 MHz)
1800
1700
1600
1500
1400
1300
1200
1100
1000
900
800
700
600
500
400
300
200
100
0
-100
200 190 180 170 160 150 140 130 120 110 100 90 80 70 60 50 40 30 20 10 0 -10
13C (ppm)

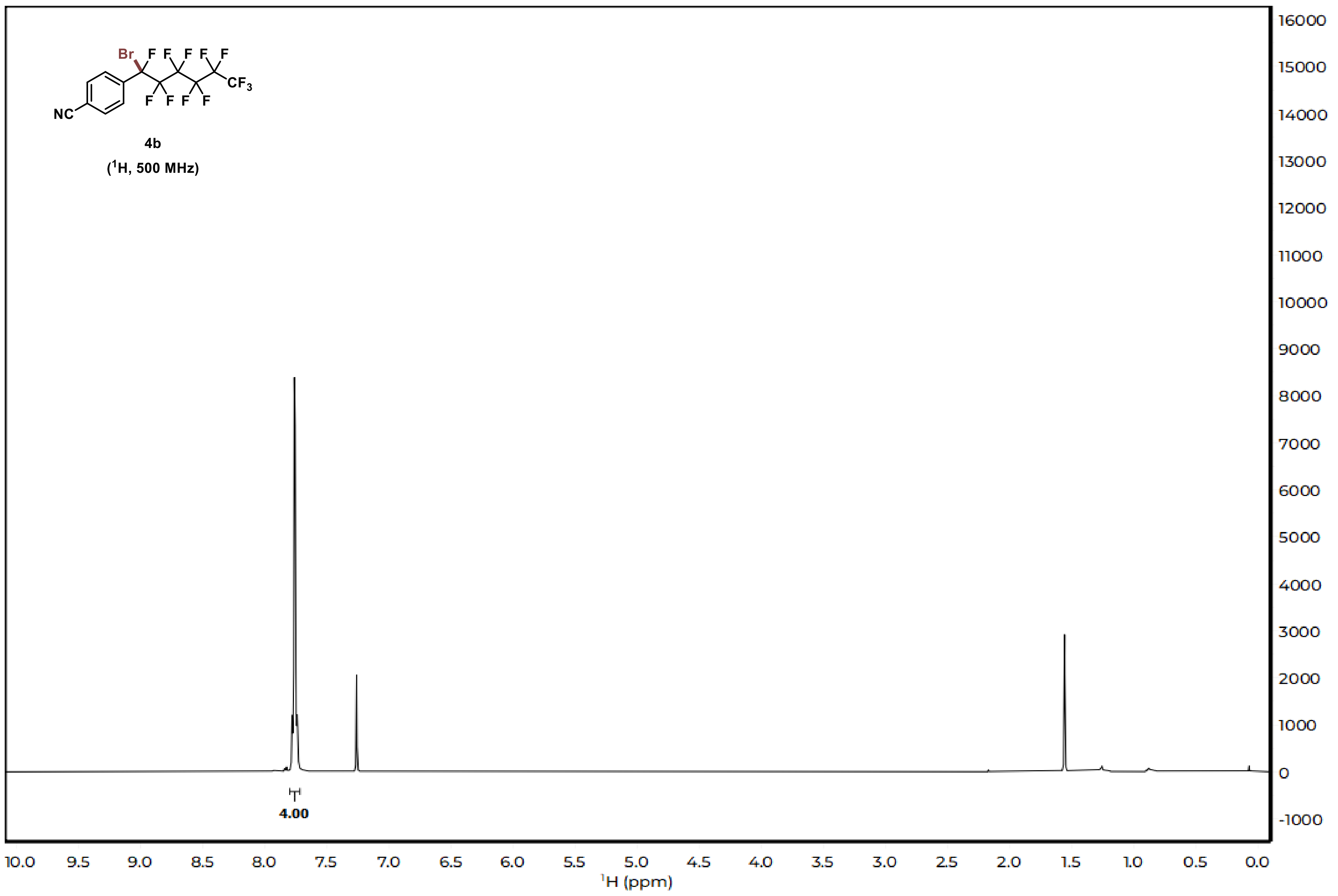
Br
F F F F F
CF3
F F F F
NC
4b
(1H, 500 MHz)
4.00
16000
15000
14000
13000
12000
11000
10000
9000
8000
7000
6000
5000
4000
3000
2000
1000
0
-1000
10.0
9.5
9.0
8.5
8.0
7.5
7.0
6.5
6.0
5.5
5.0
4.5
4.0
3.5
3.0
2.5
2.0
1.5
1.0
0.5
0.0
1H (ppm)

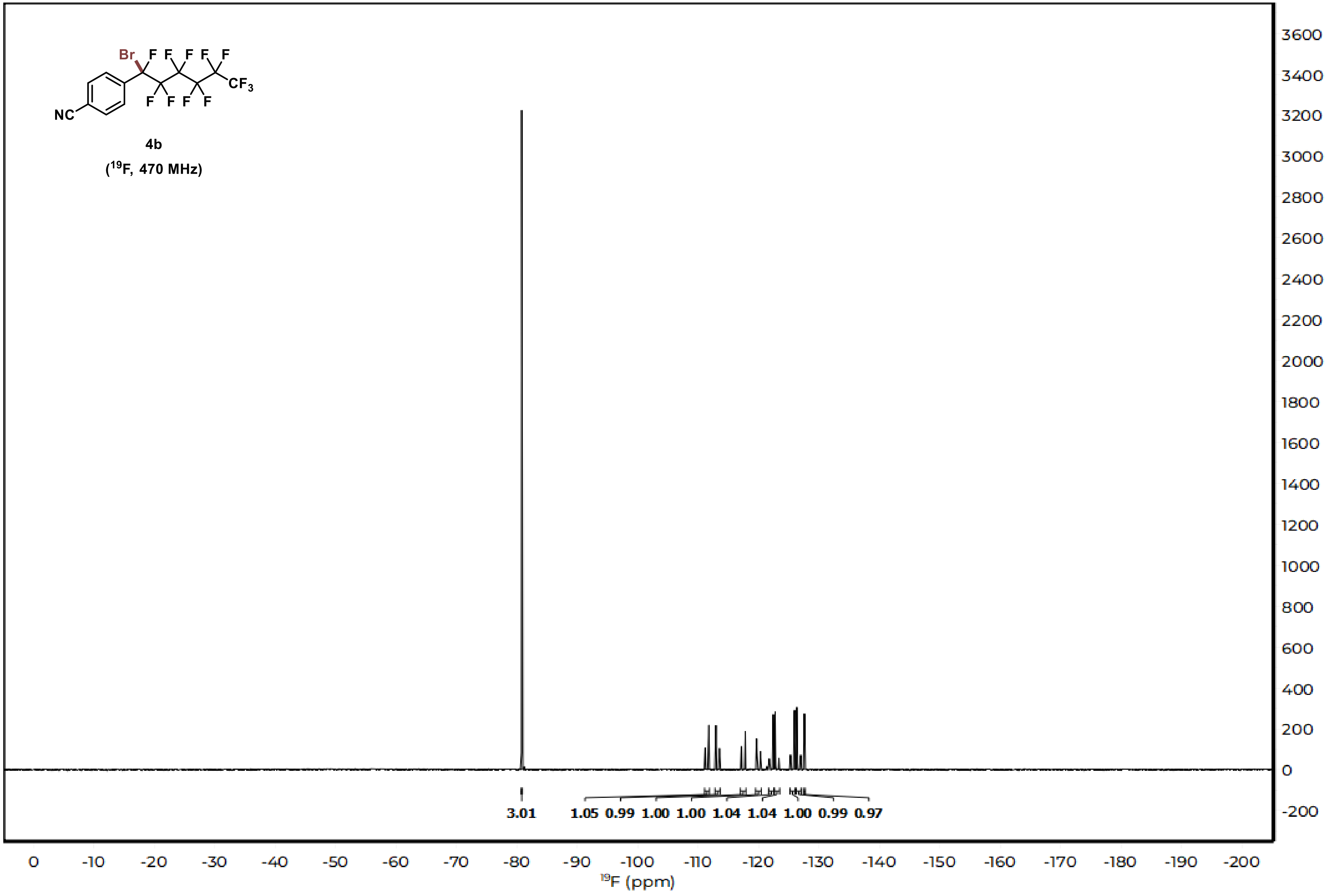
Br
F F F F F
CF3
F F F F
NC
4b
(19F, 470 MHz)
3600
3400
3200
3000
2800
2600
2400
2200
2000
1800
1600
1400
1200
1000
800
600
400
200
0
-200
3.01
1.05 0.99 1.00 1.00 1.04 1.04 1.00 0.99 0.97
0 -10 -20 -30 -40 -50 -60 -70 -80 -90 -100 -110 -120 -130 -140 -150 -160 -170 -180 -190 -200
19F (ppm)

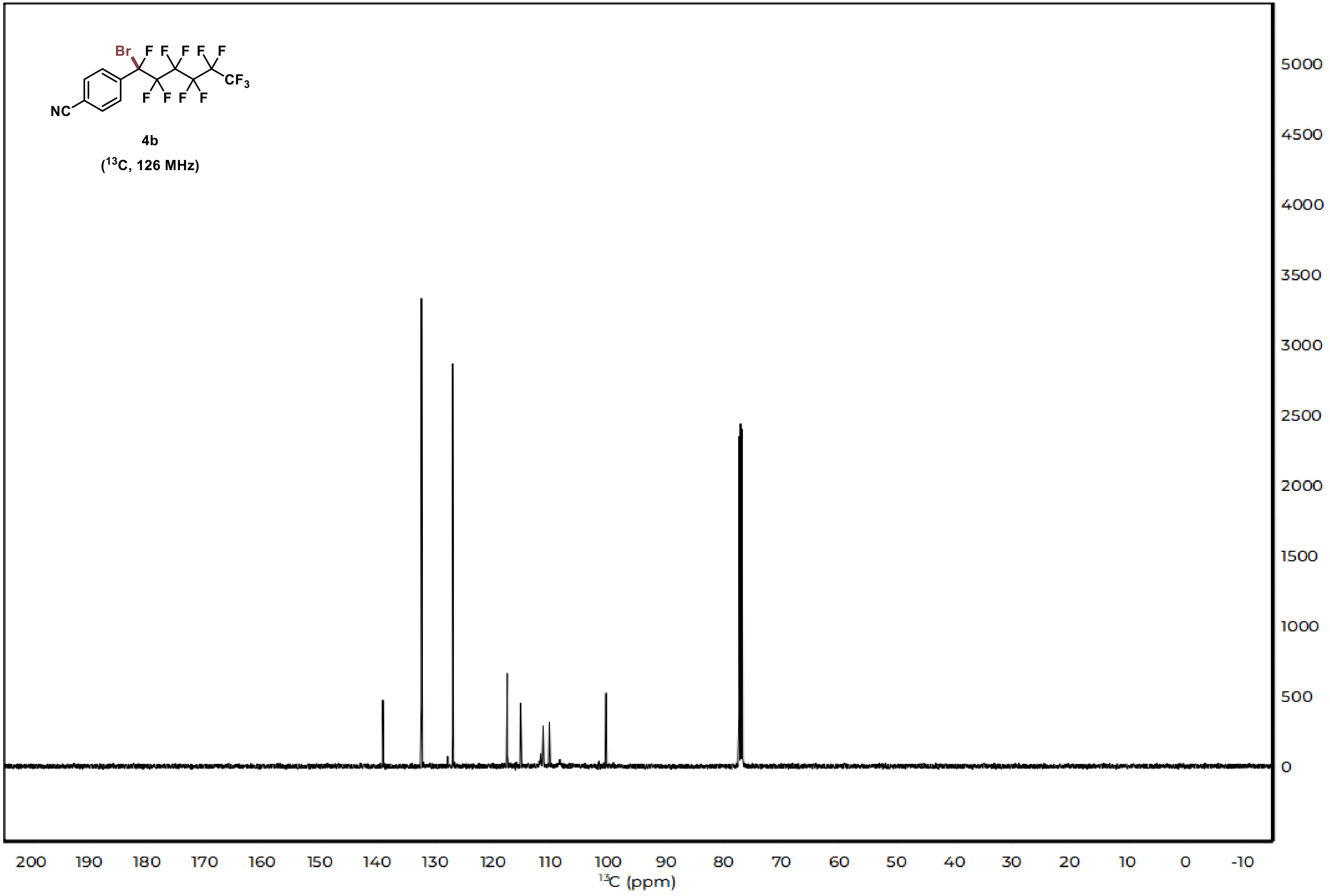

Br
F F F F F
CF3
F F F F
NC
4b
(13C, 126 MHz)
5000
4500
4000
3500
3000
2500
2000
1500
1000
500
0
200 190 180 170 160 150 140 130 120 110 100 90 80 70 60 50 40 30 20 10 0 -10
13C (ppm)

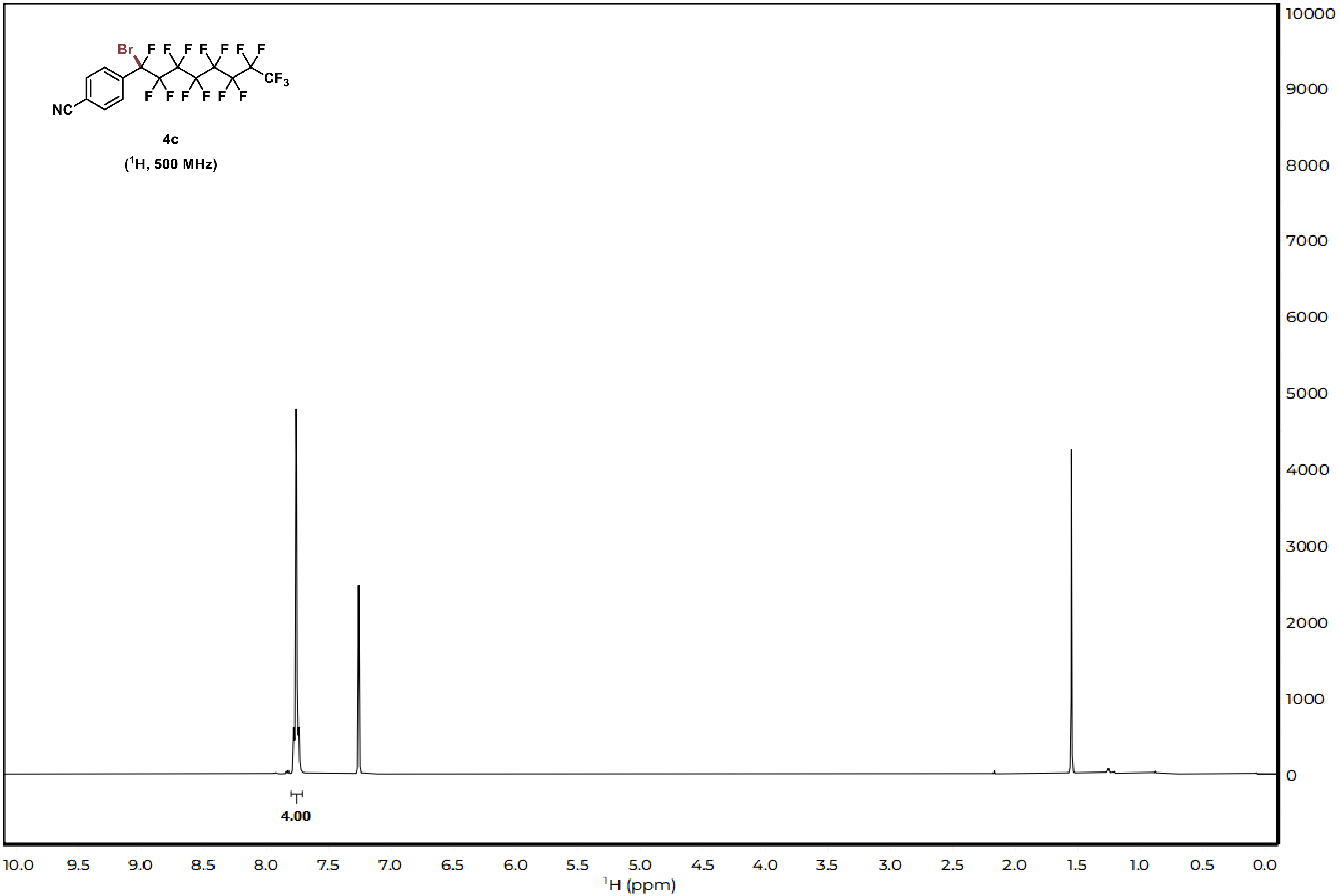

Br
F F F F F F F
CF3
F F F F F F
NC
4c
(1H, 500 MHz)
10000
9000
8000
7000
6000
5000
4000
3000
2000
1000
0
4.00
10.0 9.5 9.0 8.5 8.0 7.5 7.0 6.5 6.0 5.5 5.0 4.5 4.0 3.5 3.0 2.5 2.0 1.5 1.0 0.5 0.0
1H (ppm)

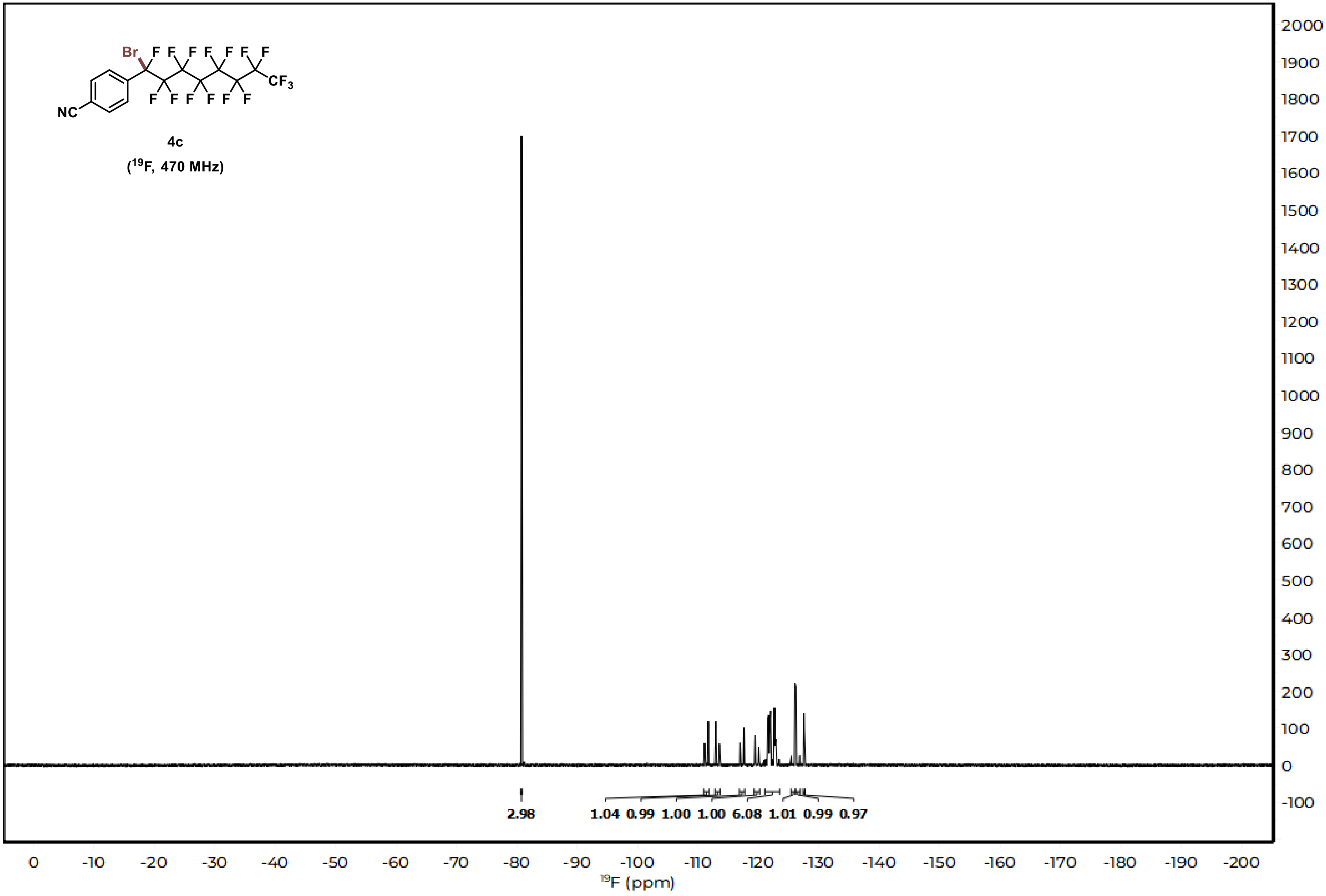

Br
F F F F F F F
CF3
F F F F F F
NC
4c
(19F, 470 MHz)
2000
1900
1800
1700
1600
1500
1400
1300
1200
1100
1000
900
800
700
600
500
400
300
200
100
0
-100
2.98
1.04 0.99 1.00 1.00 6.08 1.01 0.99 0.97
0 -10 -20 -30 -40 -50 -60 -70 -80 -90 -100 -110 -120 -130 -140 -150 -160 -170 -180 -190 -200
19F (ppm)

4c
(^{13}C, 126 MHz)

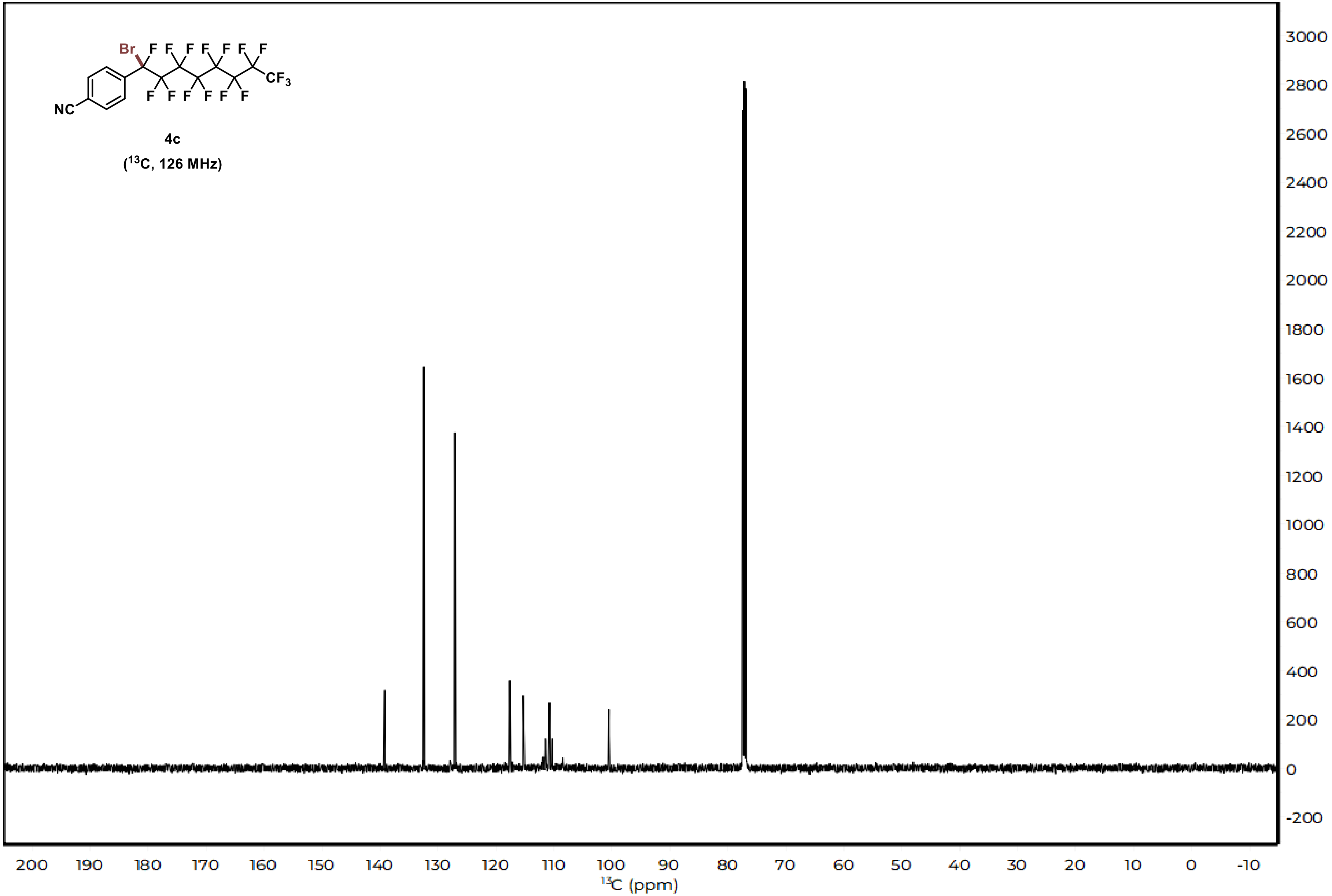

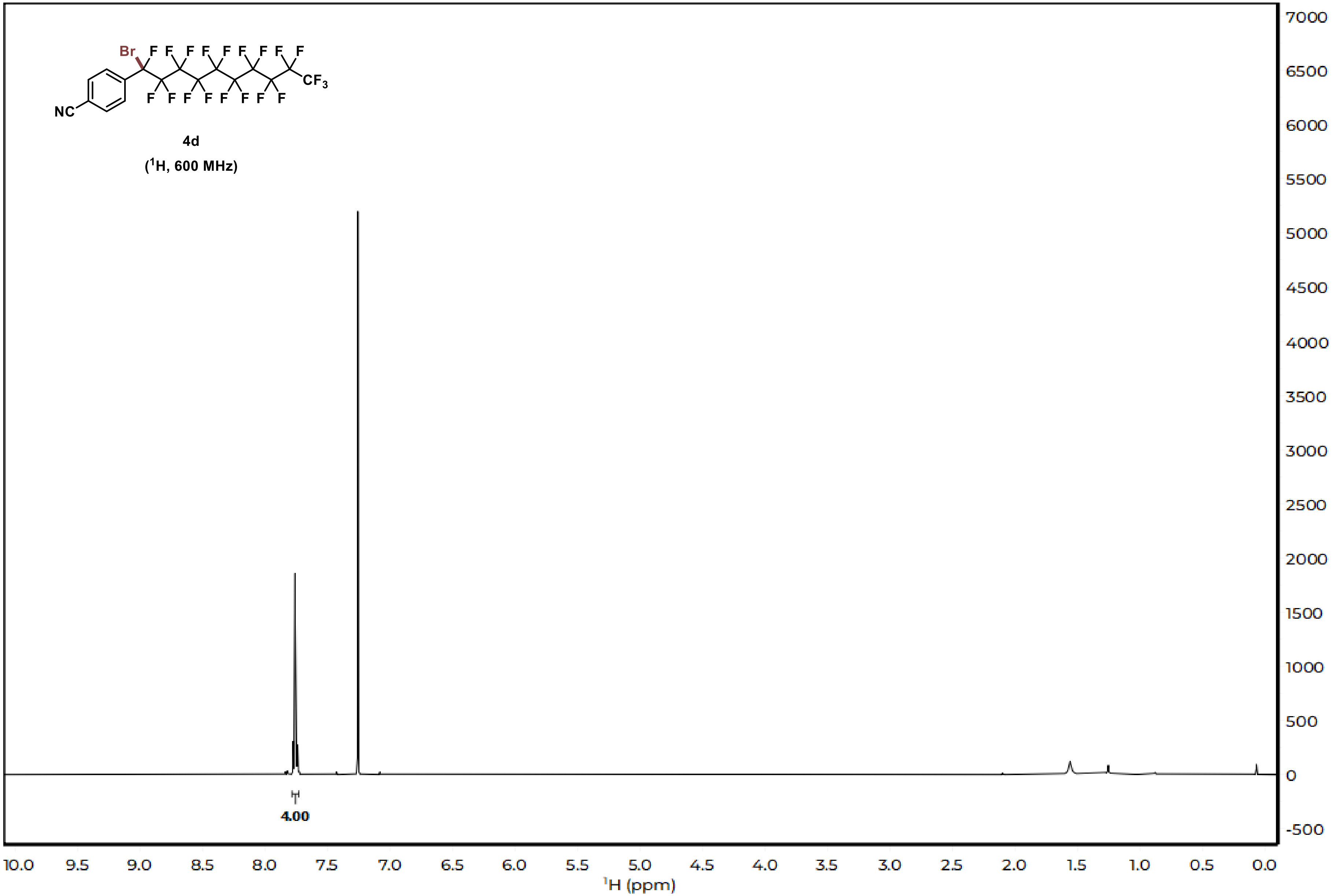

Br
NC
CF3
4d
(1H, 600 MHz)
4.00
1H (ppm)

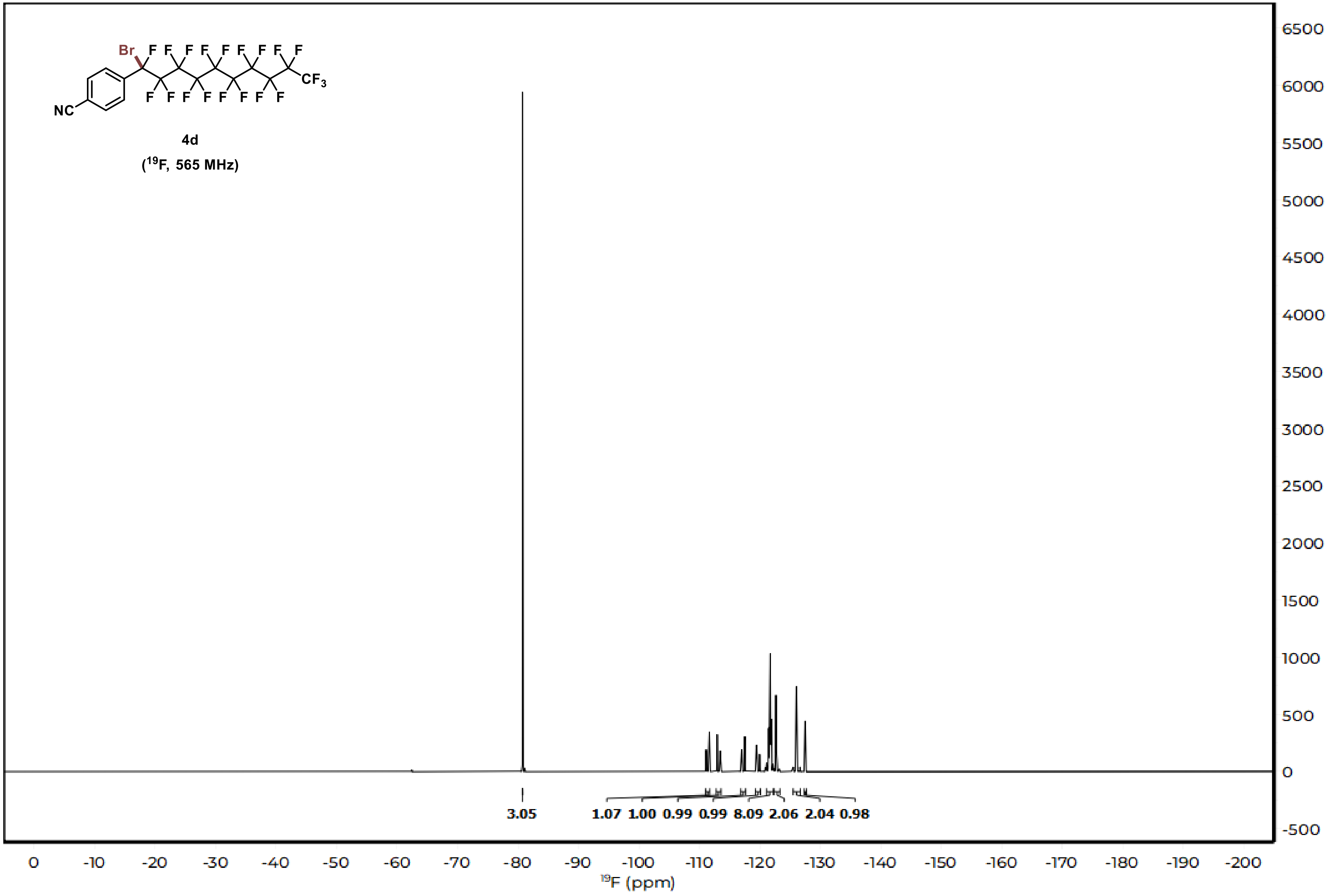

NC
Br
CF3
4d
(19F, 565 MHz)
3.05
1.07 1.00 0.99 0.99 8.09 2.06 2.04 0.98
19F (ppm)

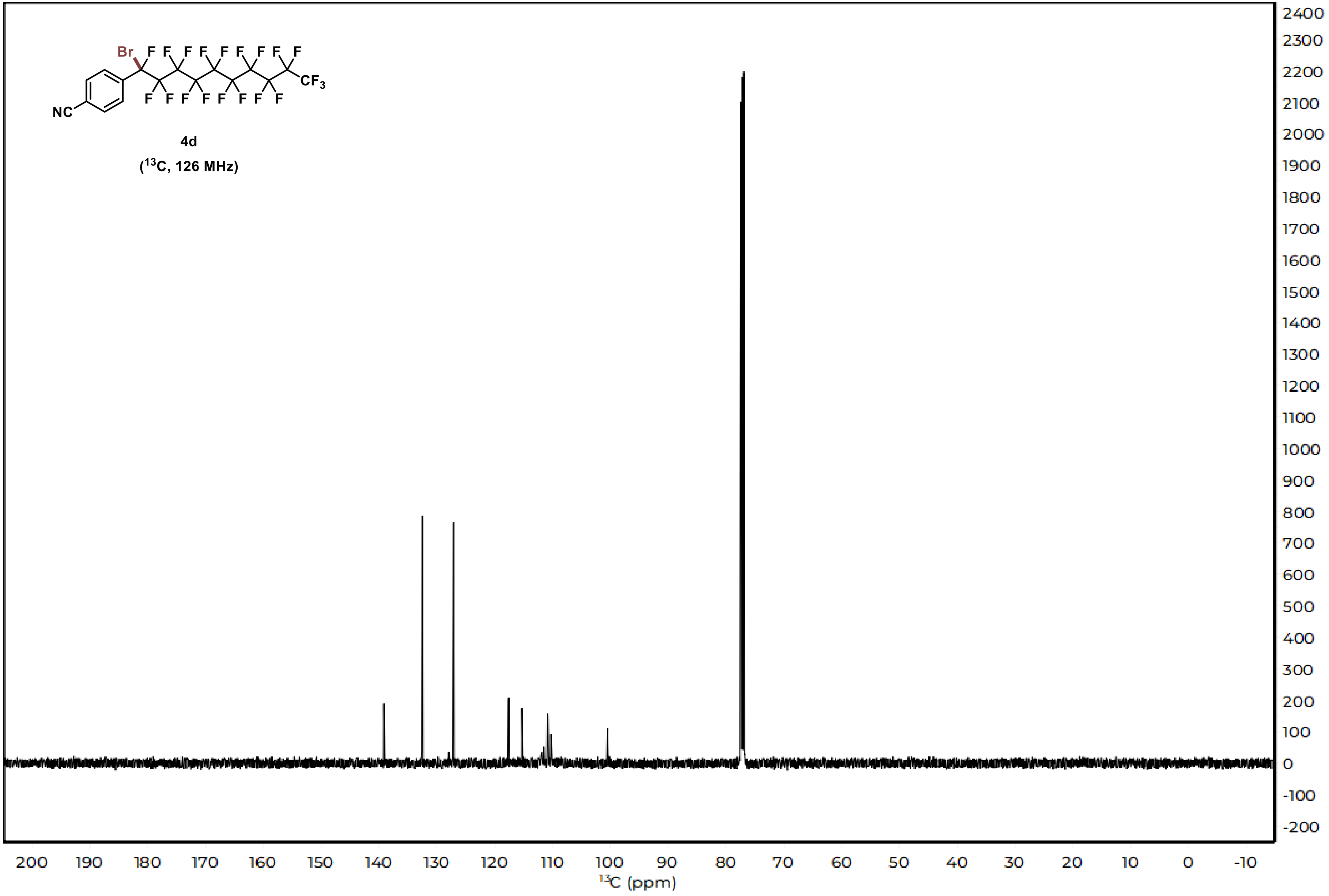

Br
F F F F F F F F F F
F F F F F F F F
CF3
NC
4d
(13C, 126 MHz)
2400
2300
2200
2100
2000
1900
1800
1700
1600
1500
1400
1300
1200
1100
1000
900
800
700
600
500
400
300
200
100
0
-100
-200
200 190 180 170 160 150 140 130 120 110 100 90 80 70 60 50 40 30 20 10 0 -10
13C (ppm)

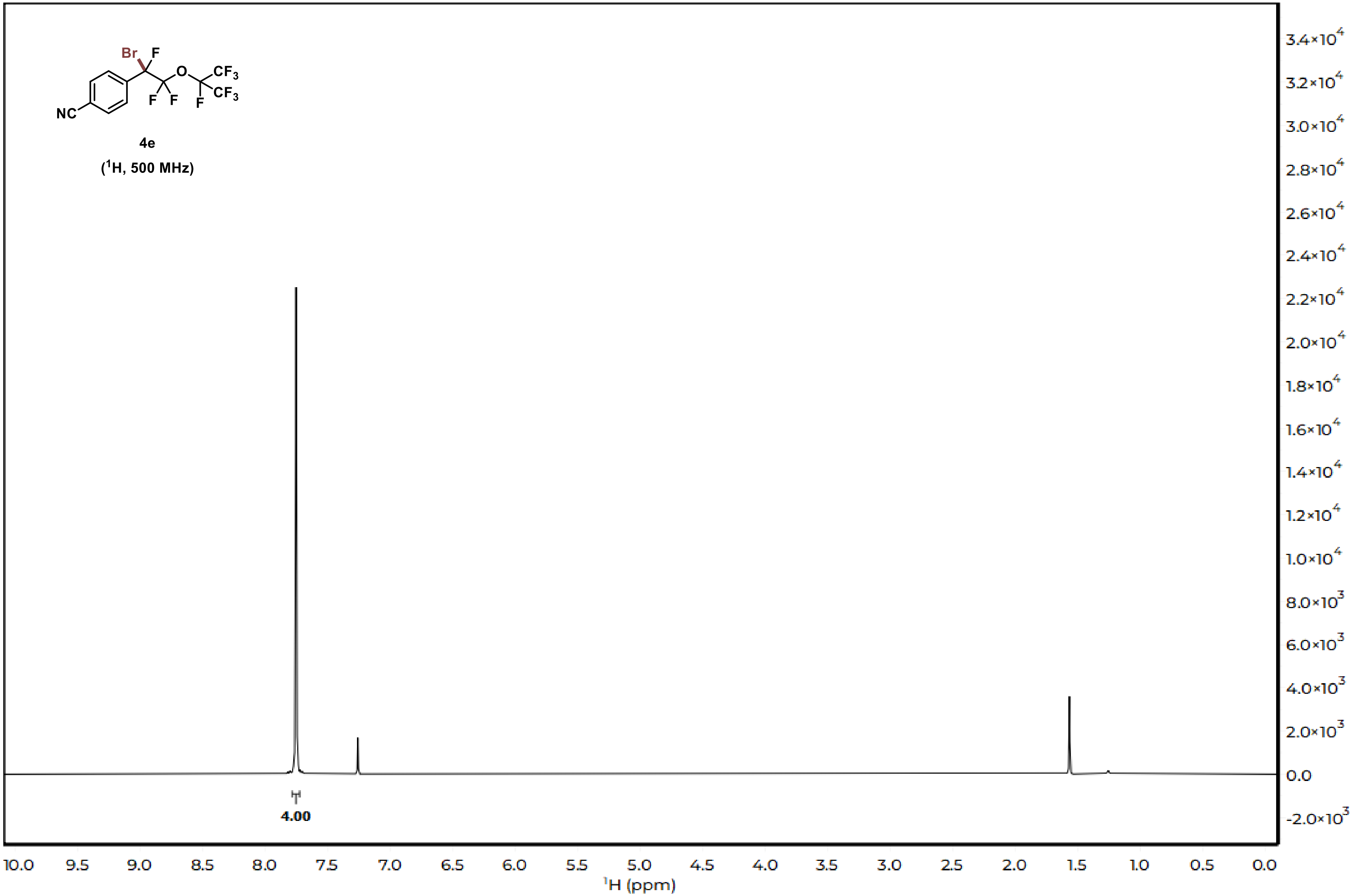

Br
F
O
CF3
CF3
F
F
F
NC
4e
(1H, 500 MHz)
4.00
3.4×10^4
3.2×10^4
3.0×10^4
2.8×10^4
2.6×10^4
2.4×10^4
2.2×10^4
2.0×10^4
1.8×10^4
1.6×10^4
1.4×10^4
1.2×10^4
1.0×10^4
8.0×10^3
6.0×10^3
4.0×10^3
2.0×10^3
0.0
-2.0×10^3
10.0 9.5 9.0 8.5 8.0 7.5 7.0 6.5 6.0 5.5 5.0 4.5 4.0 3.5 3.0 2.5 2.0 1.5 1.0 0.5 0.0
1H (ppm)

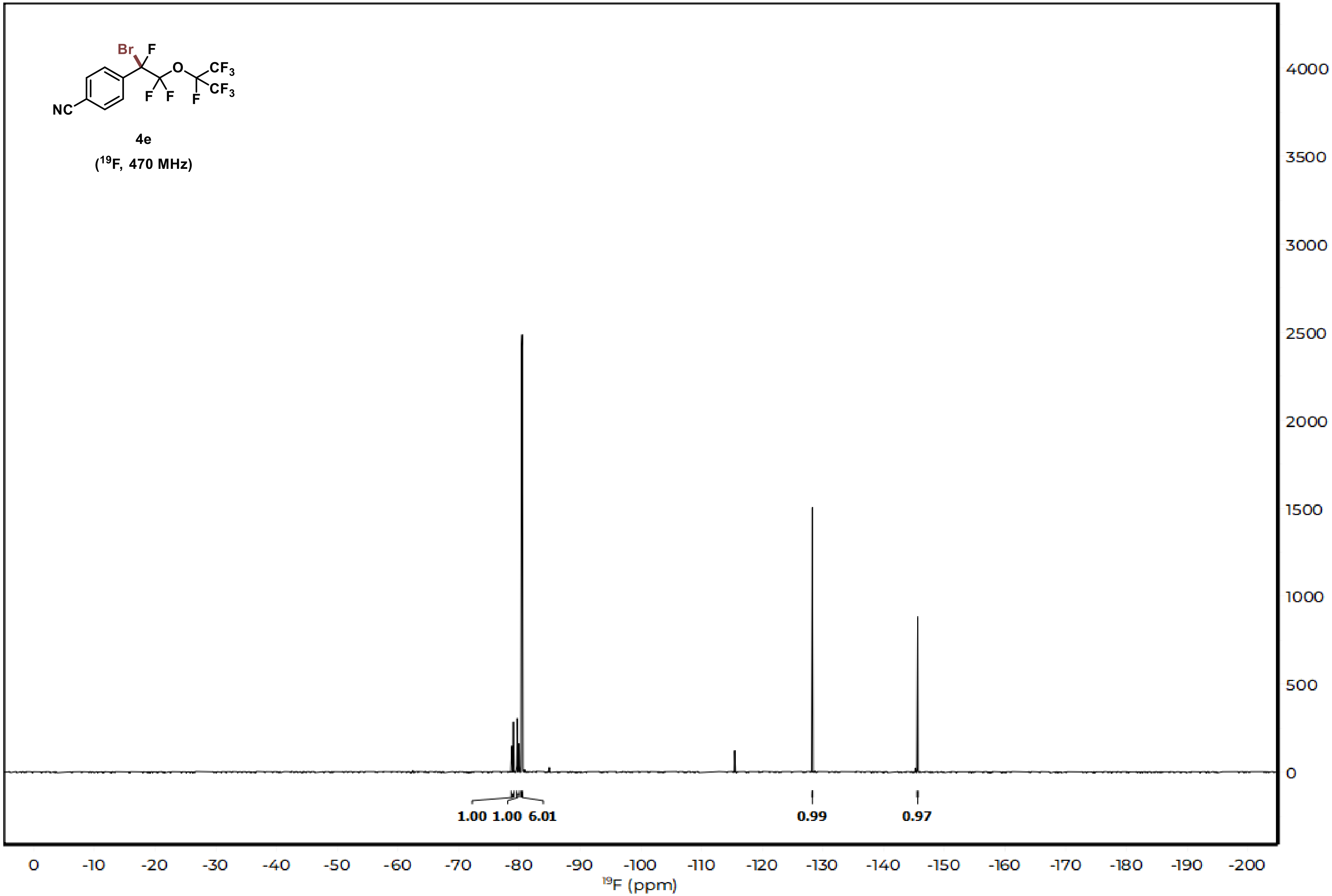
Br
F
O
CF3
CF3
NC
F
F
F
4e
(19F, 470 MHz)
4000
3500
3000
2500
2000
1500
1000
500
0
1.00 1.00 6.01
0.99
0.97
0 -10 -20 -30 -40 -50 -60 -70 -80 -90 -100 -110 -120 -130 -140 -150 -160 -170 -180 -190 -200
19F (ppm)

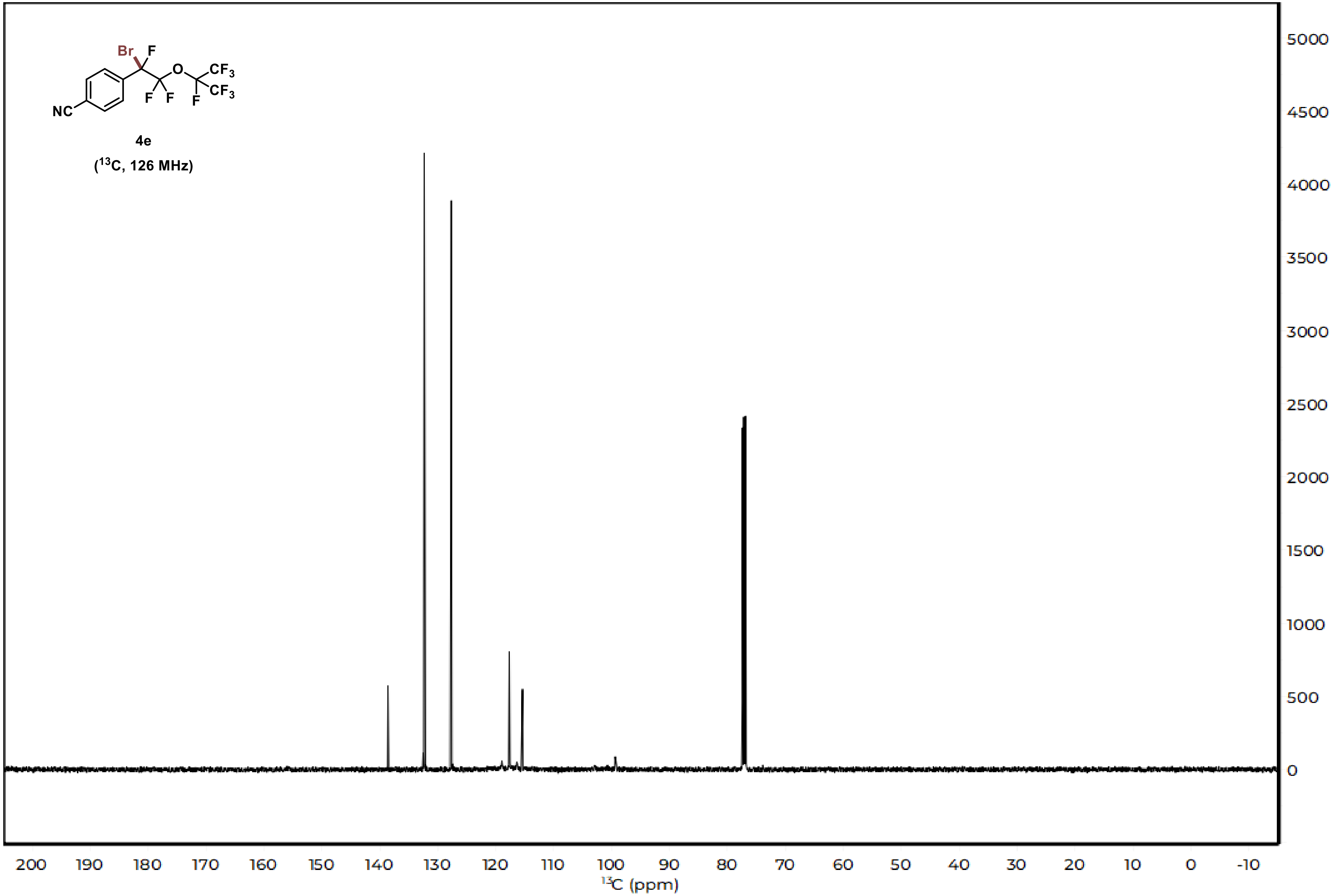
Br
F
O
CF3
CF3
F
F
F
NC
4e
(13C, 126 MHz)
5000
4500
4000
3500
3000
2500
2000
1500
1000
500
0
200 190 180 170 160 150 140 130 120 110 100 90 80 70 60 50 40 30 20 10 0 -10
13C (ppm)

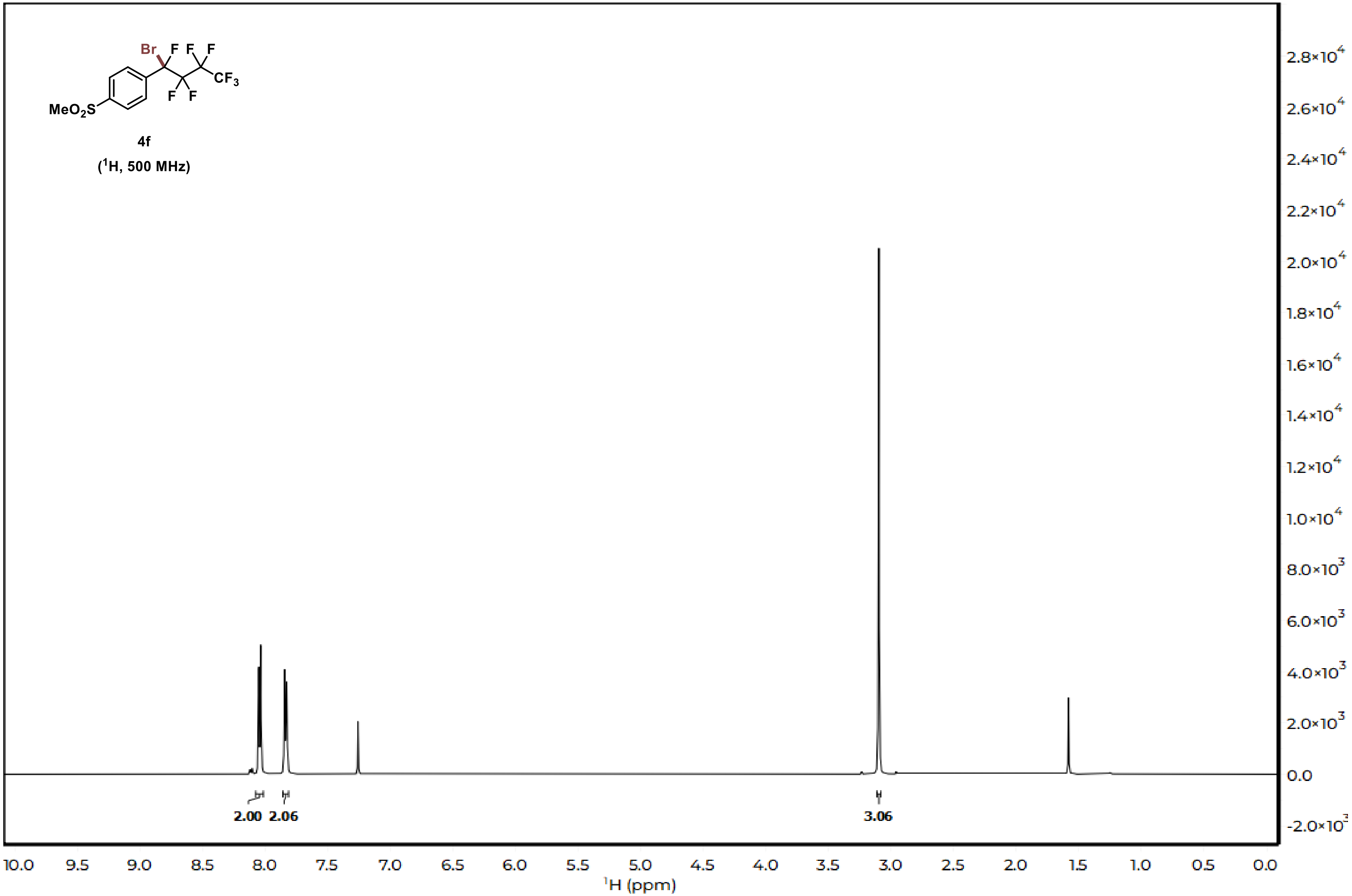
Br
F F F
CF3
F F
MeO2S
4f
(1H, 500 MHz)
2.8×10^4
2.6×10^4
2.4×10^4
2.2×10^4
2.0×10^4
1.8×10^4
1.6×10^4
1.4×10^4
1.2×10^4
1.0×10^4
8.0×10^3
6.0×10^3
4.0×10^3
2.0×10^3
0.0
-2.0×10^3
2.00
2.06
3.06
10.0 9.5 9.0 8.5 8.0 7.5 7.0 6.5 6.0 5.5 5.0 4.5 4.0 3.5 3.0 2.5 2.0 1.5 1.0 0.5 0.0
1H (ppm)

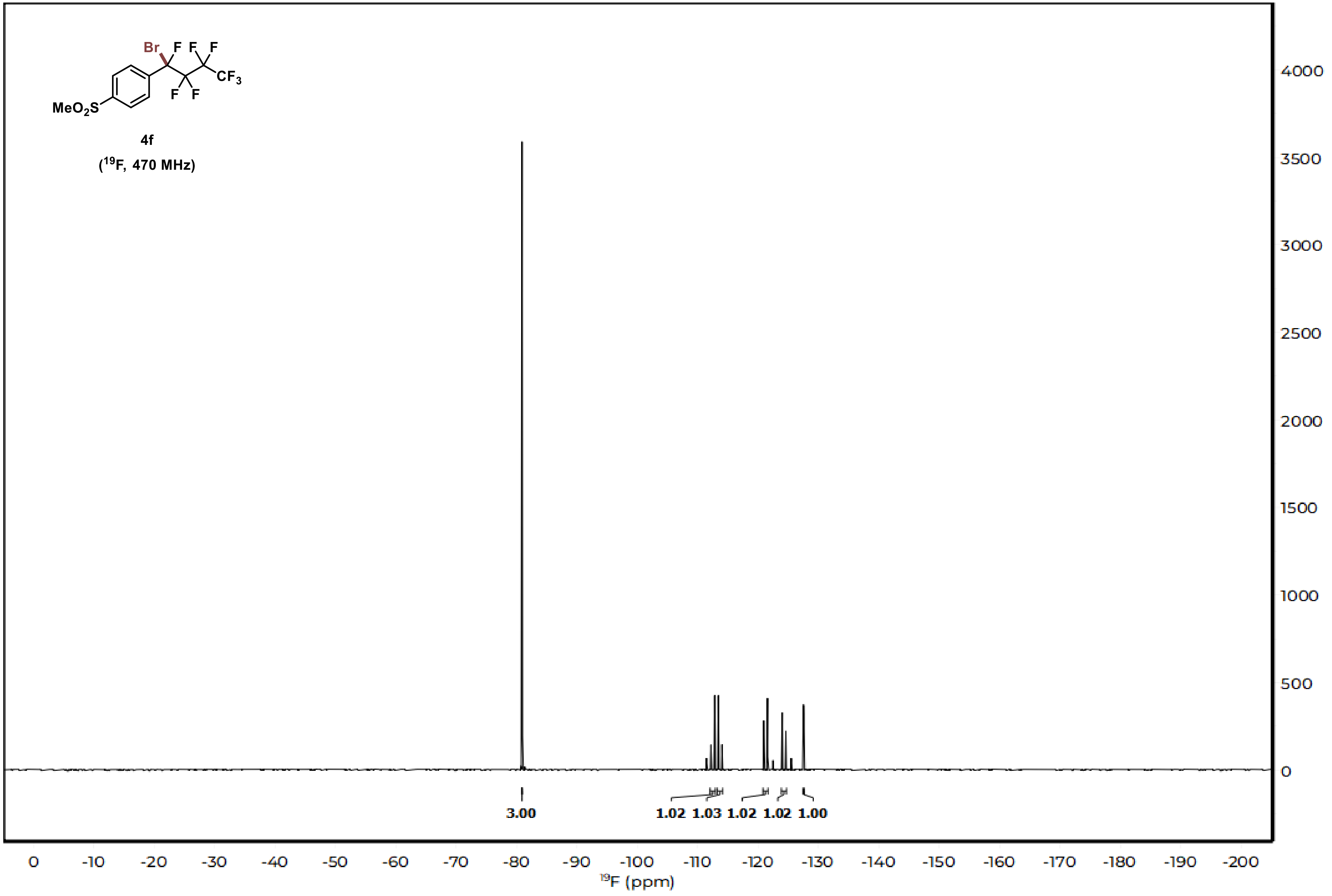

Br
F
F
F
CF3
F
F
MeO2S
4f
(19F, 470 MHz)
4000
3500
3000
2500
2000
1500
1000
500
0
3.00
1.02
1.03
1.02
1.02
1.00
0
-10
-20
-30
-40
-50
-60
-70
-80
-90
-100
-110
-120
-130
-140
-150
-160
-170
-180
-190
-200
19F (ppm)

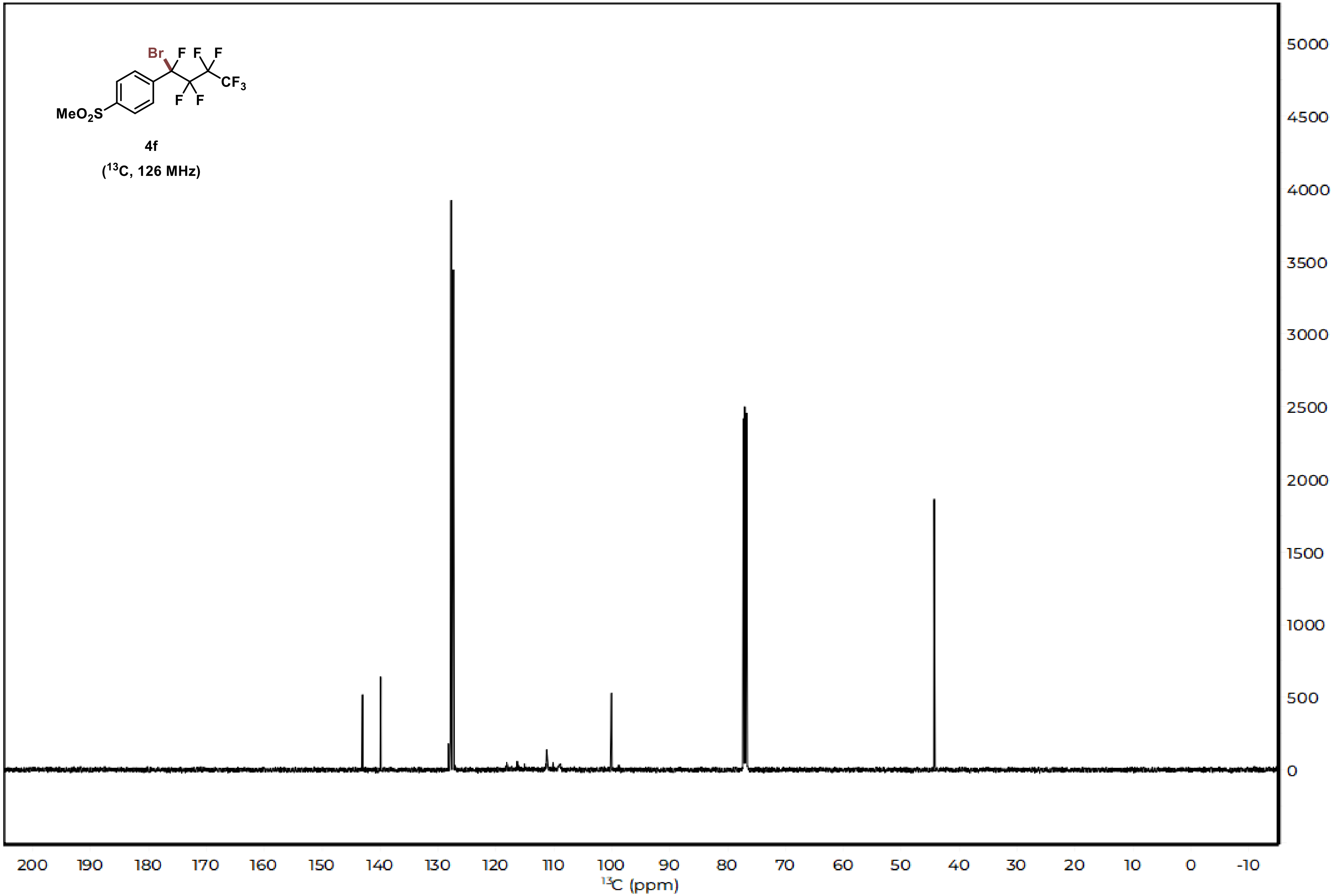
Br F F F
CF3
F F
MeO2S
4f
(13C, 126 MHz)
5000
4500
4000
3500
3000
2500
2000
1500
1000
500
0
200 190 180 170 160 150 140 130 120 110 100 90 80 70 60 50 40 30 20 10 0 -10
13C (ppm)

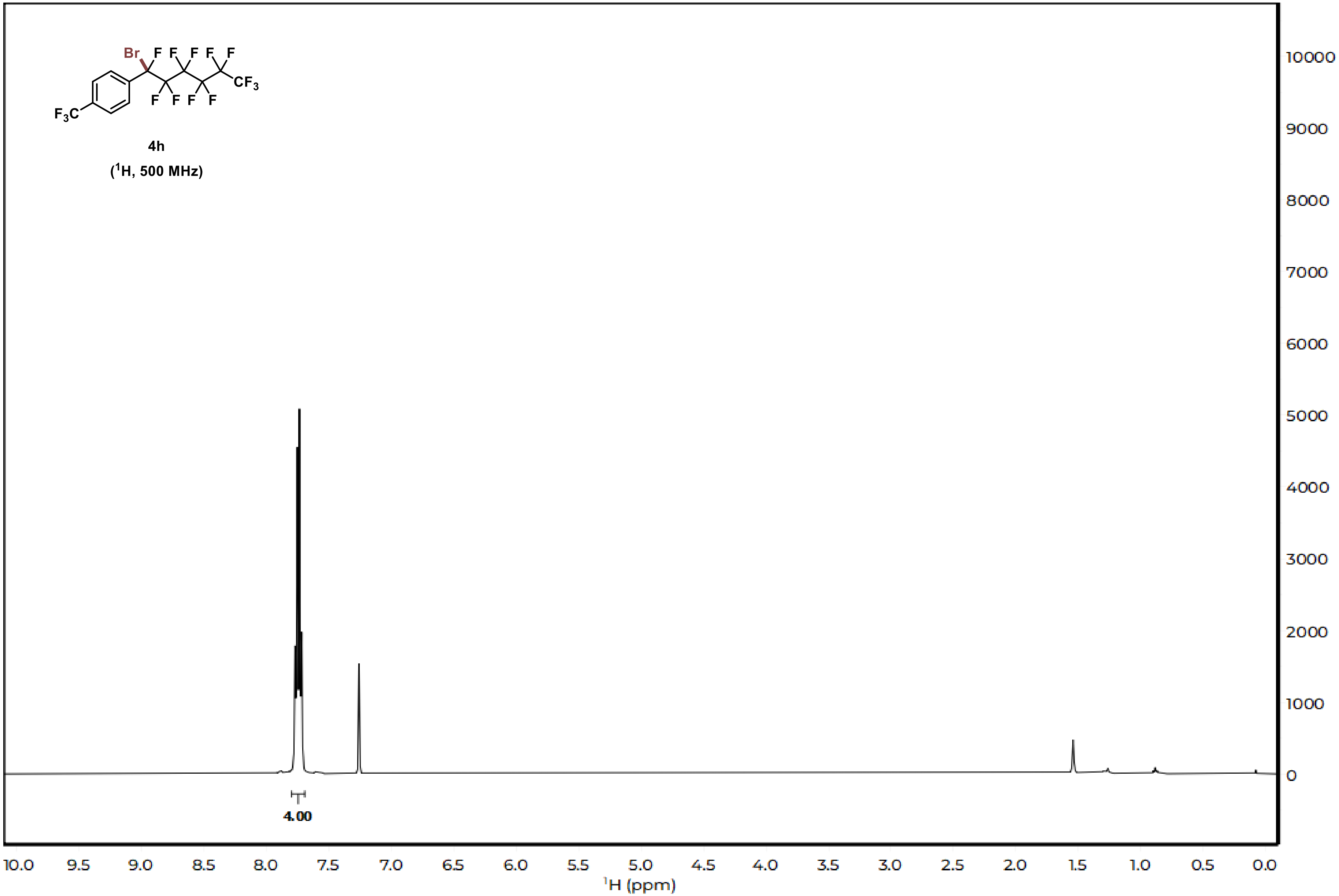

Br
F F F F F
CF3
F3C
F F F F
4h
(1H, 500 MHz)
4.00
10000
9000
8000
7000
6000
5000
4000
3000
2000
1000
0
10.0 9.5 9.0 8.5 8.0 7.5 7.0 6.5 6.0 5.5 5.0 4.5 4.0 3.5 3.0 2.5 2.0 1.5 1.0 0.5 0.0
1H (ppm)

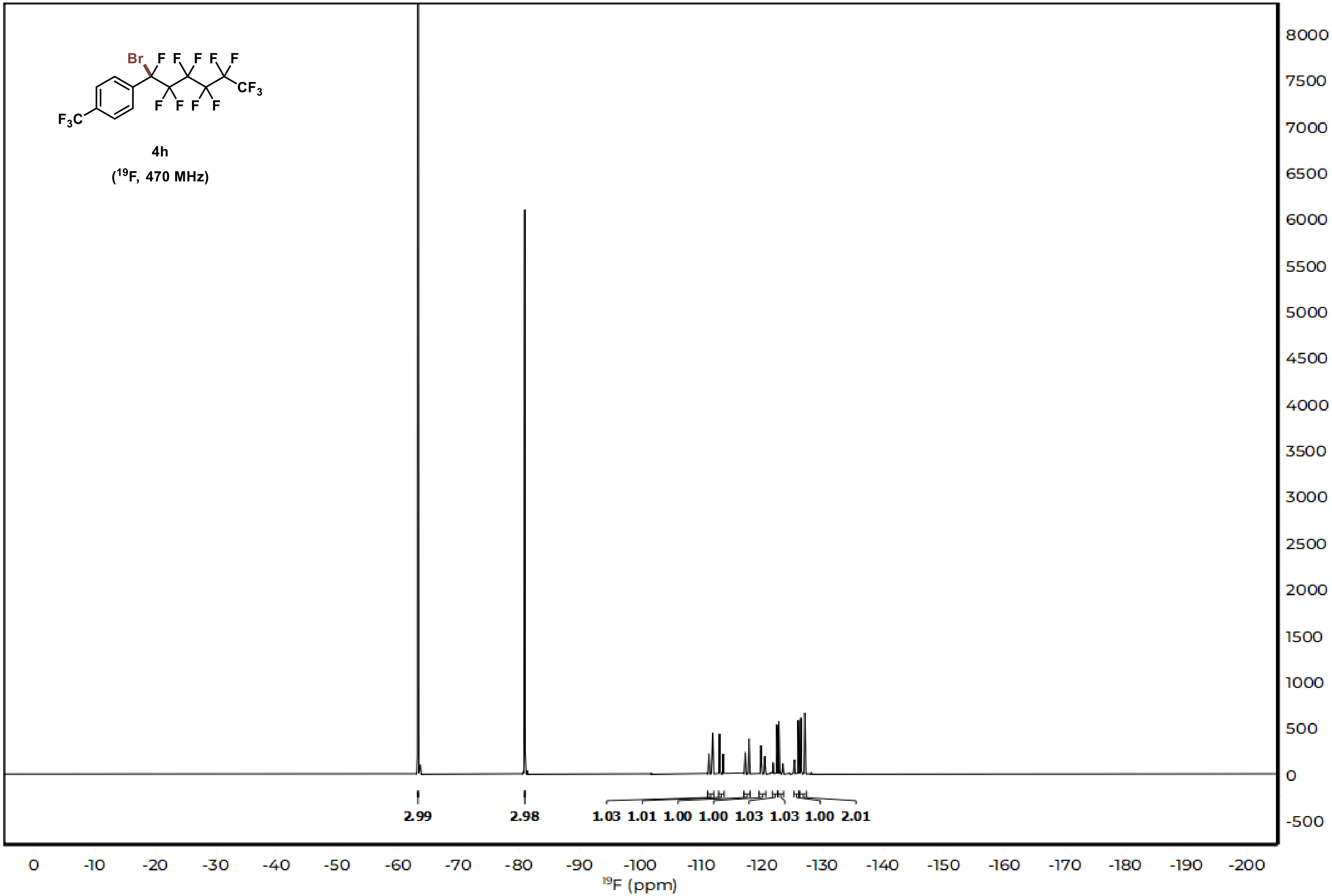
Br
F F F F F
CF3
F F F F
F3C
4h
(19F, 470 MHz)
2.99
2.98
1.03 1.01 1.00 1.00 1.03 1.03 1.00 2.01
8000
7500
7000
6500
6000
5500
5000
4500
4000
3500
3000
2500
2000
1500
1000
500
0
-500
0 -10 -20 -30 -40 -50 -60 -70 -80 -90 -100 -110 -120 -130 -140 -150 -160 -170 -180 -190 -200
19F (ppm)

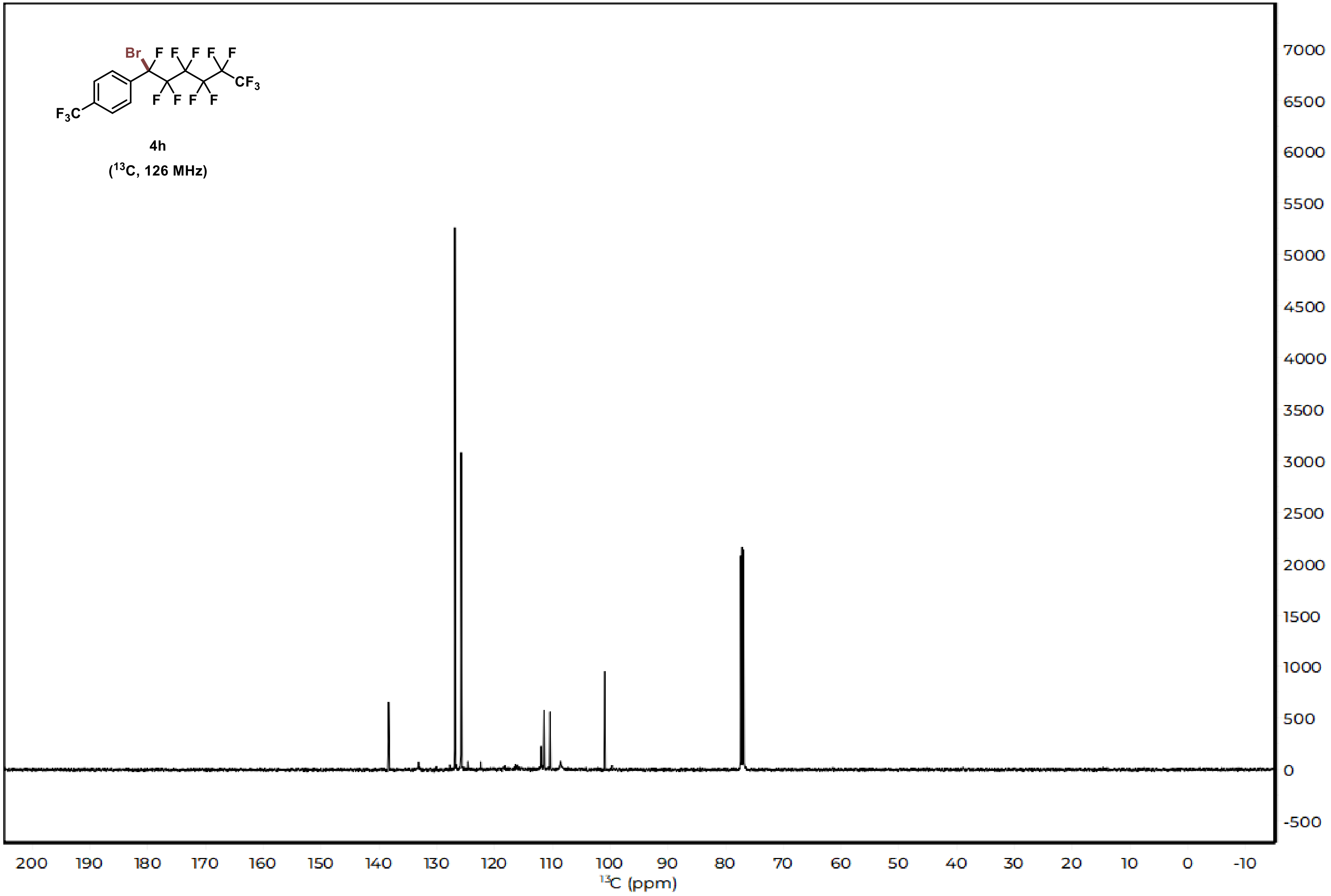
Br F F F F F
CF3
F F F F
F3C
4h
(13C, 126 MHz)
7000
6500
6000
5500
5000
4500
4000
3500
3000
2500
2000
1500
1000
500
0
-500
200 190 180 170 160 150 140 130 120 110 100 90 80 70 60 50 40 30 20 10 0 -10
13C (ppm)

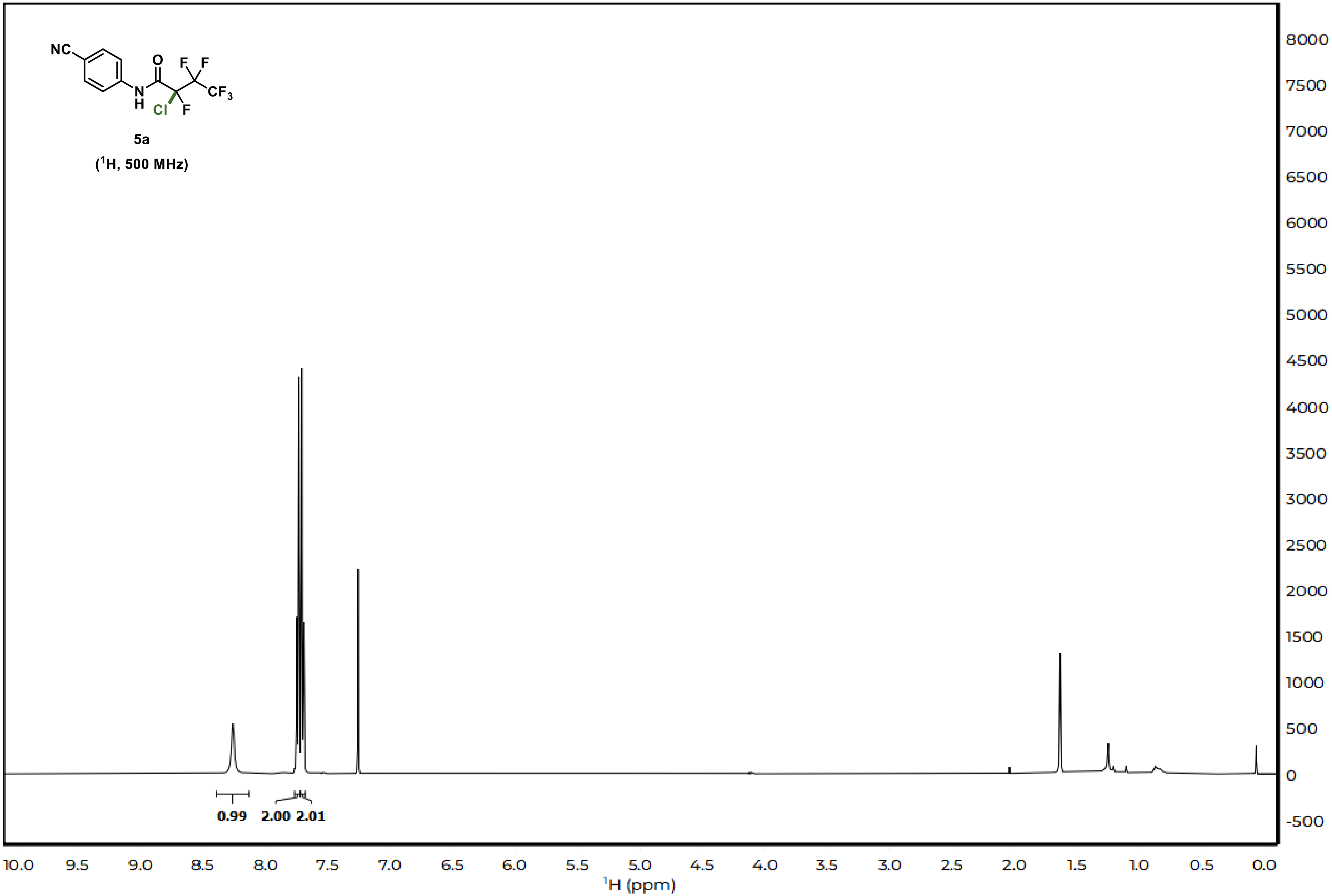

NC
O
F
F
N
H
CF3
Cl
F
5a
(1H, 500 MHz)
0.99
2.00
2.01
1H (ppm)

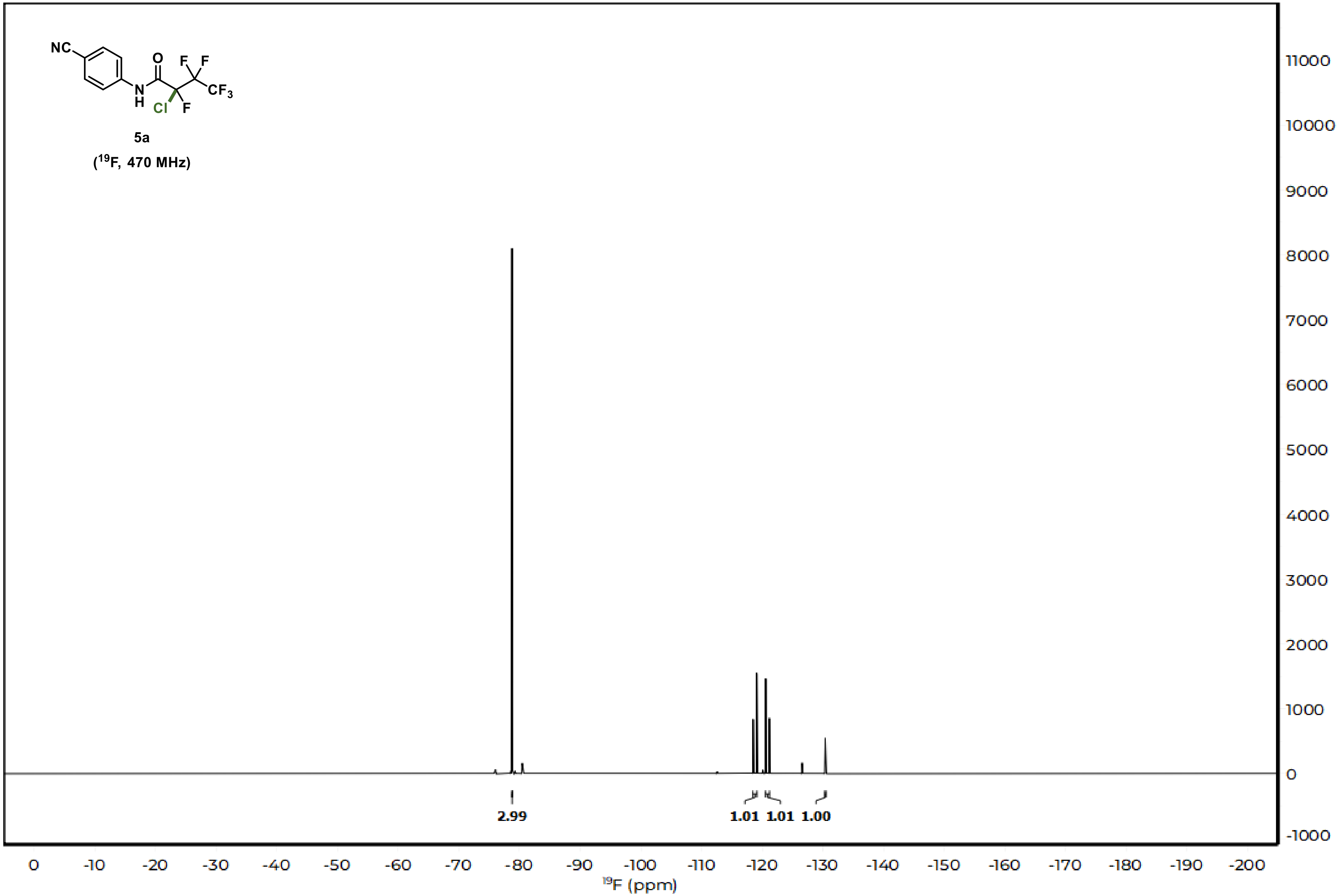

NC
O
F F
N
H
CF3
Cl
F
5a
(19F, 470 MHz)
11000
10000
9000
8000
7000
6000
5000
4000
3000
2000
1000
0
-1000
2.99
1.01
1.01
1.00
0
-10
-20
-30
-40
-50
-60
-70
-80
-90
-100
-110
-120
-130
-140
-150
-160
-170
-180
-190
-200
19F (ppm)

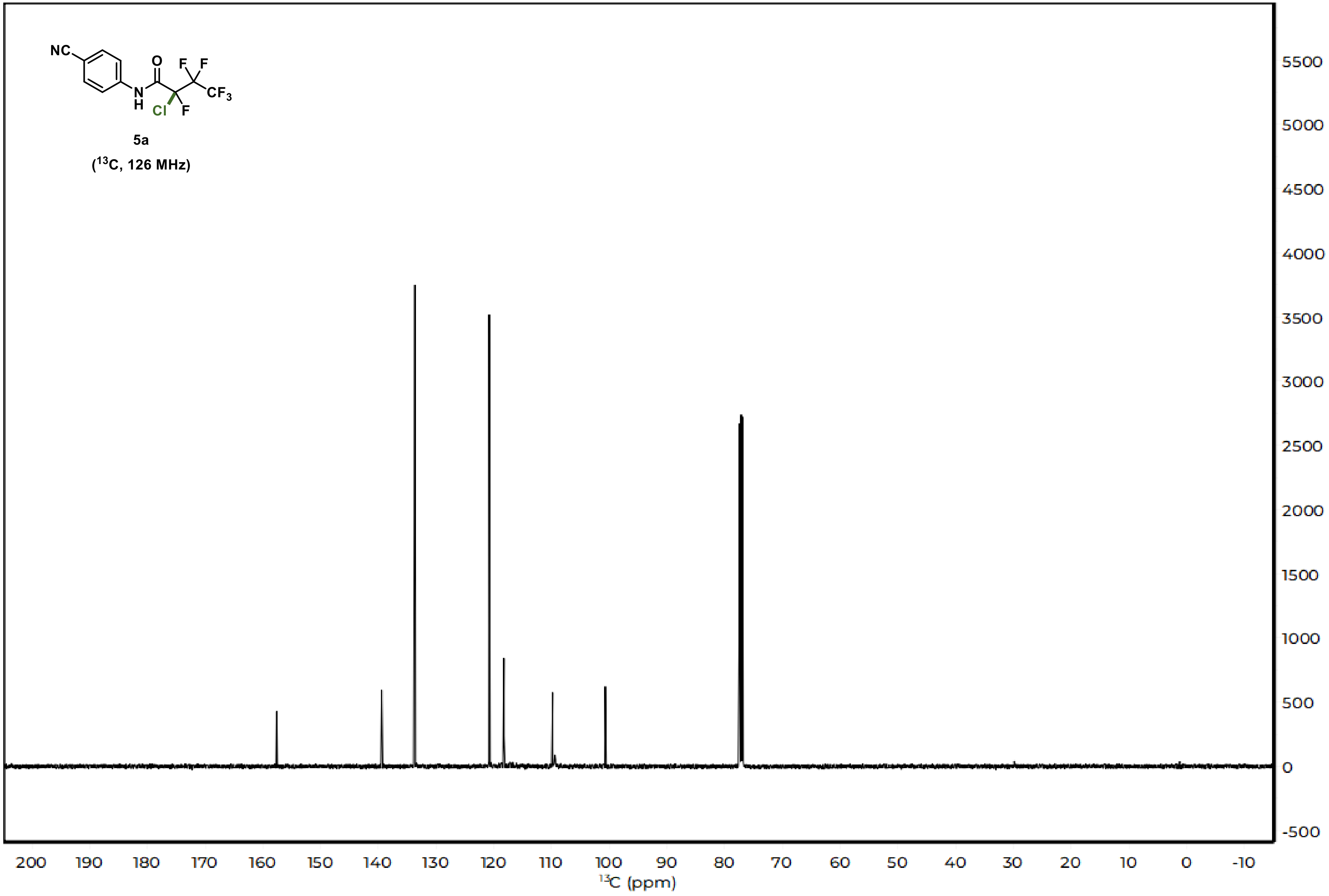
NC
O
F F
N
H
CF3
Cl F
5a
(13C, 126 MHz)
5500
5000
4500
4000
3500
3000
2500
2000
1500
1000
500
0
-500
200 190 180 170 160 150 140 130 120 110 100 90 80 70 60 50 40 30 20 10 0 -10
13C (ppm)

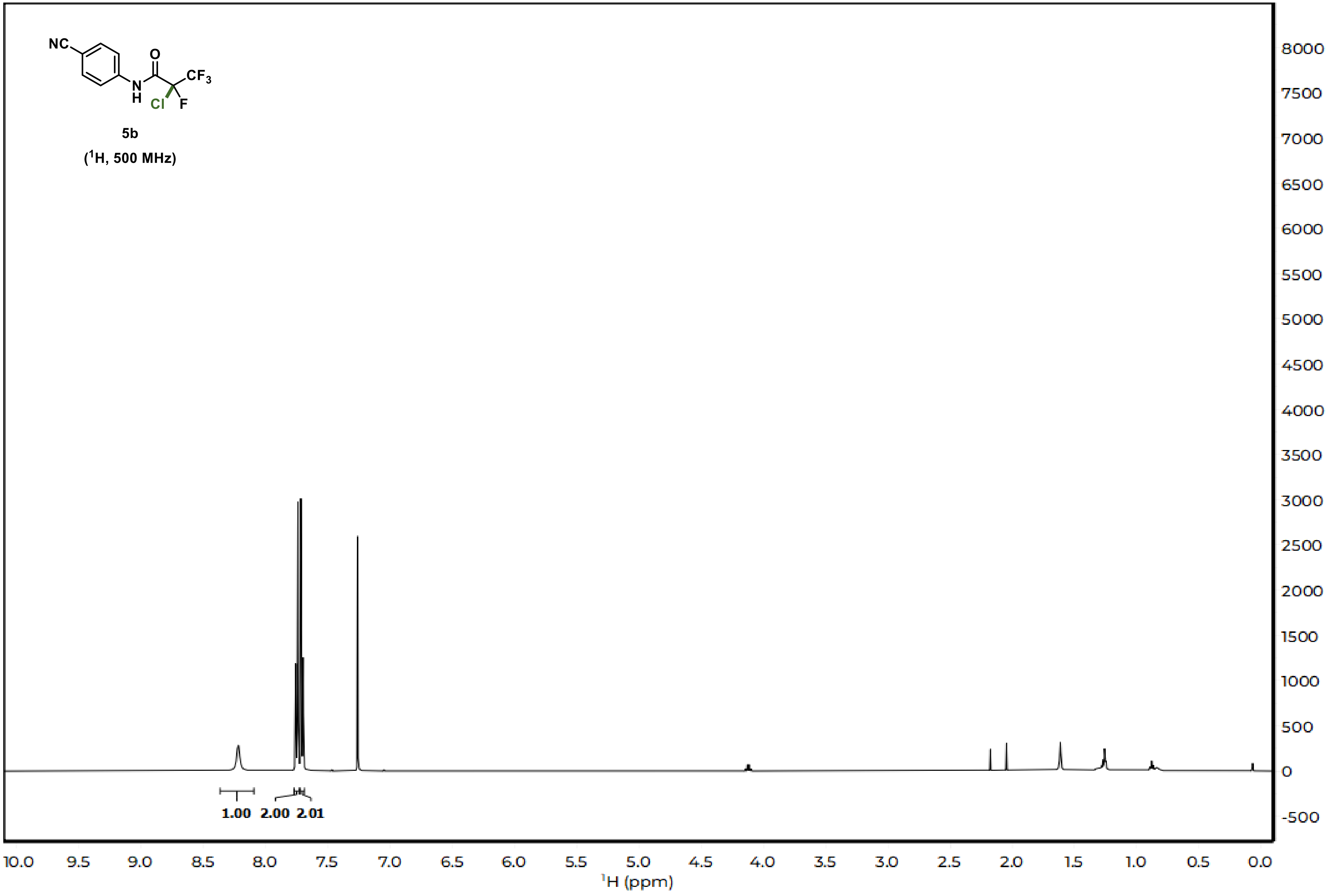

NC
O
N
H
CF3
Cl
F
5b
(1H, 500 MHz)
8000
7500
7000
6500
6000
5500
5000
4500
4000
3500
3000
2500
2000
1500
1000
500
0
-500
1.00
2.00
2.01
10.0
9.5
9.0
8.5
8.0
7.5
7.0
6.5
6.0
5.5
5.0
4.5
4.0
3.5
3.0
2.5
2.0
1.5
1.0
0.5
0.0
1H (ppm)

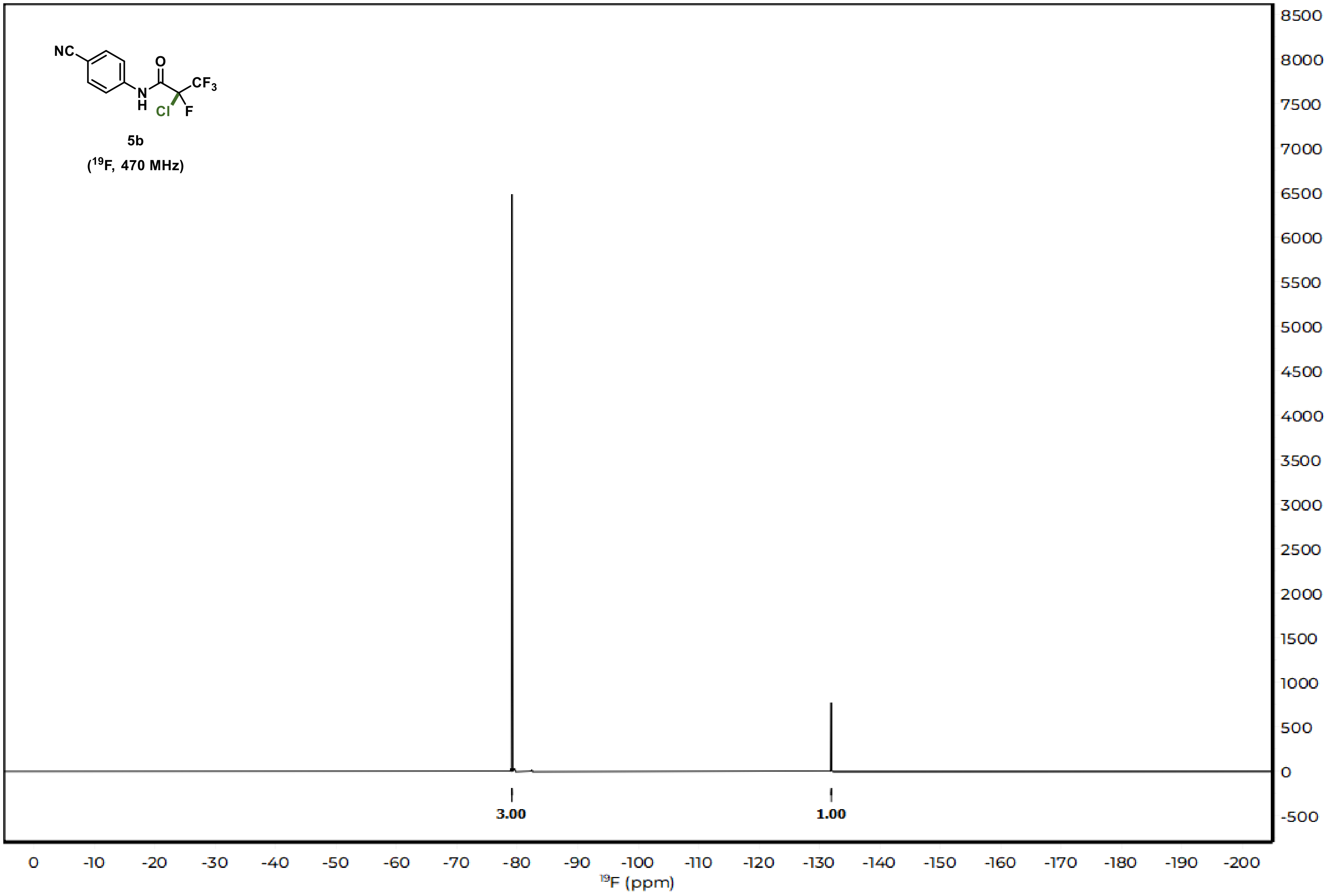
NC
O
CF3
N
H
Cl
F
5b
(19F, 470 MHz)
8500
8000
7500
7000
6500
6000
5500
5000
4500
4000
3500
3000
2500
2000
1500
1000
500
0
-500
3.00
1.00
0
-10
-20
-30
-40
-50
-60
-70
-80
-90
-100
-110
-120
-130
-140
-150
-160
-170
-180
-190
-200
19F (ppm)

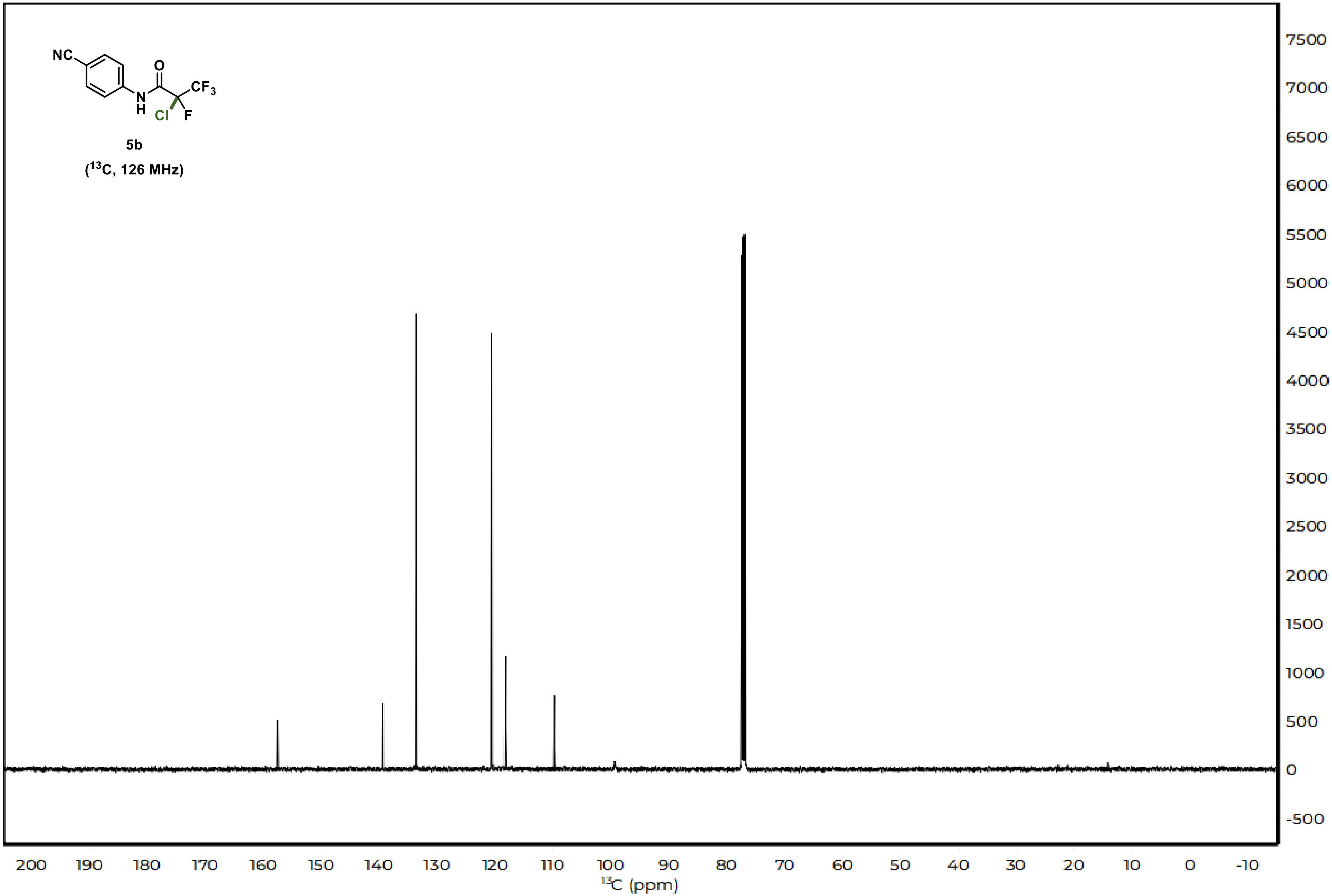

NC
O
CF3
N
H
Cl
F
5b
(13C, 126 MHz)
7500
7000
6500
6000
5500
5000
4500
4000
3500
3000
2500
2000
1500
1000
500
0
-500
200 190 180 170 160 150 140 130 120 110 100 90 80 70 60 50 40 30 20 10 0 -10
13C (ppm)

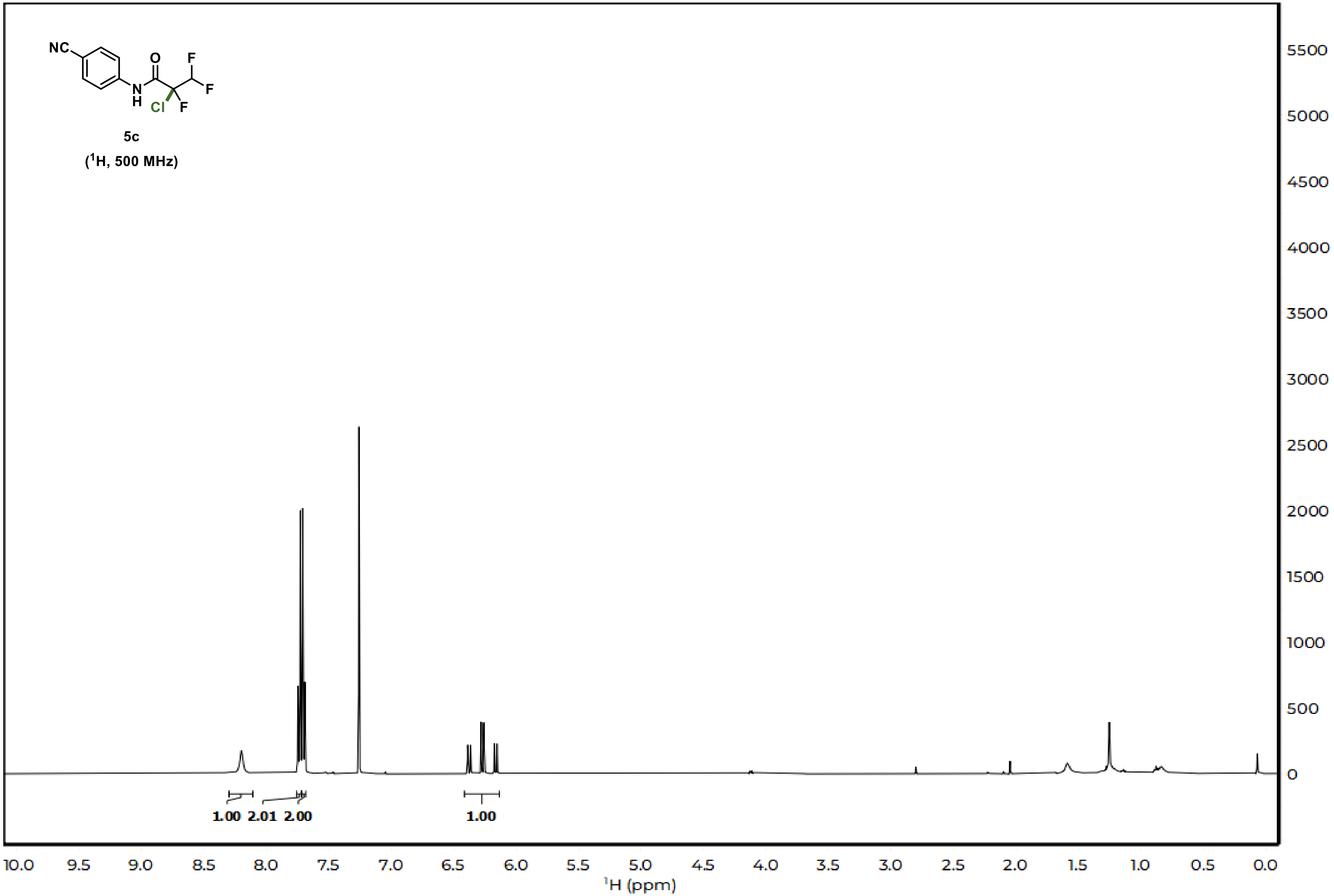
5c
(1H, 500 MHz)
5500
5000
4500
4000
3500
3000
2500
2000
1500
1000
500
0
1.00 2.01 2.00
1.00
10.0 9.5 9.0 8.5 8.0 7.5 7.0 6.5 6.0 5.5 5.0 4.5 4.0 3.5 3.0 2.5 2.0 1.5 1.0 0.5 0.0
1H (ppm)

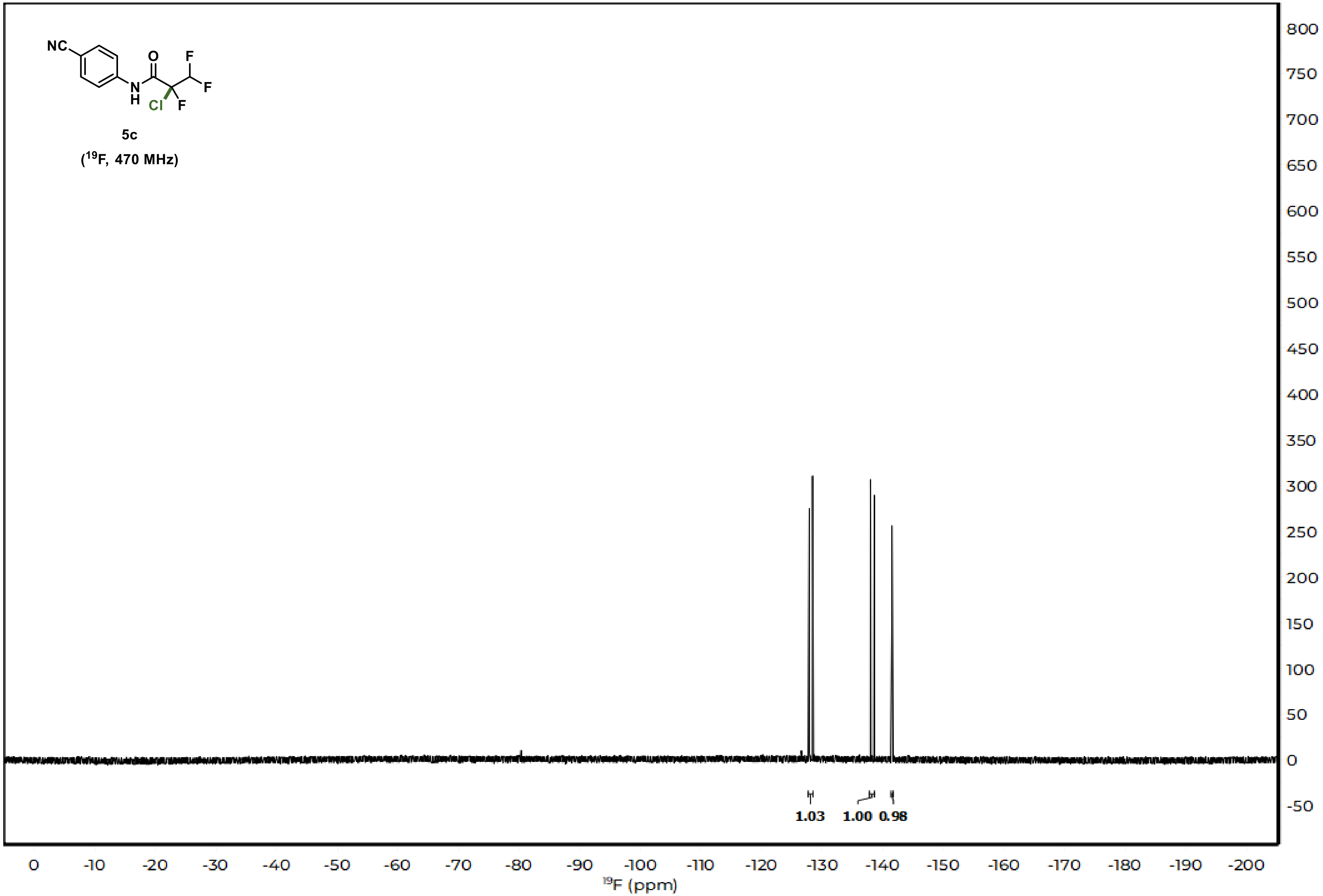

NC
O
F
F
N
H
Cl
F
5c
(19F, 470 MHz)
800
750
700
650
600
550
500
450
400
350
300
250
200
150
100
50
0
-50
1.03
1.00
0.98
0
-10
-20
-30
-40
-50
-60
-70
-80
-90
-100
-110
-120
-130
-140
-150
-160
-170
-180
-190
-200
19F (ppm)

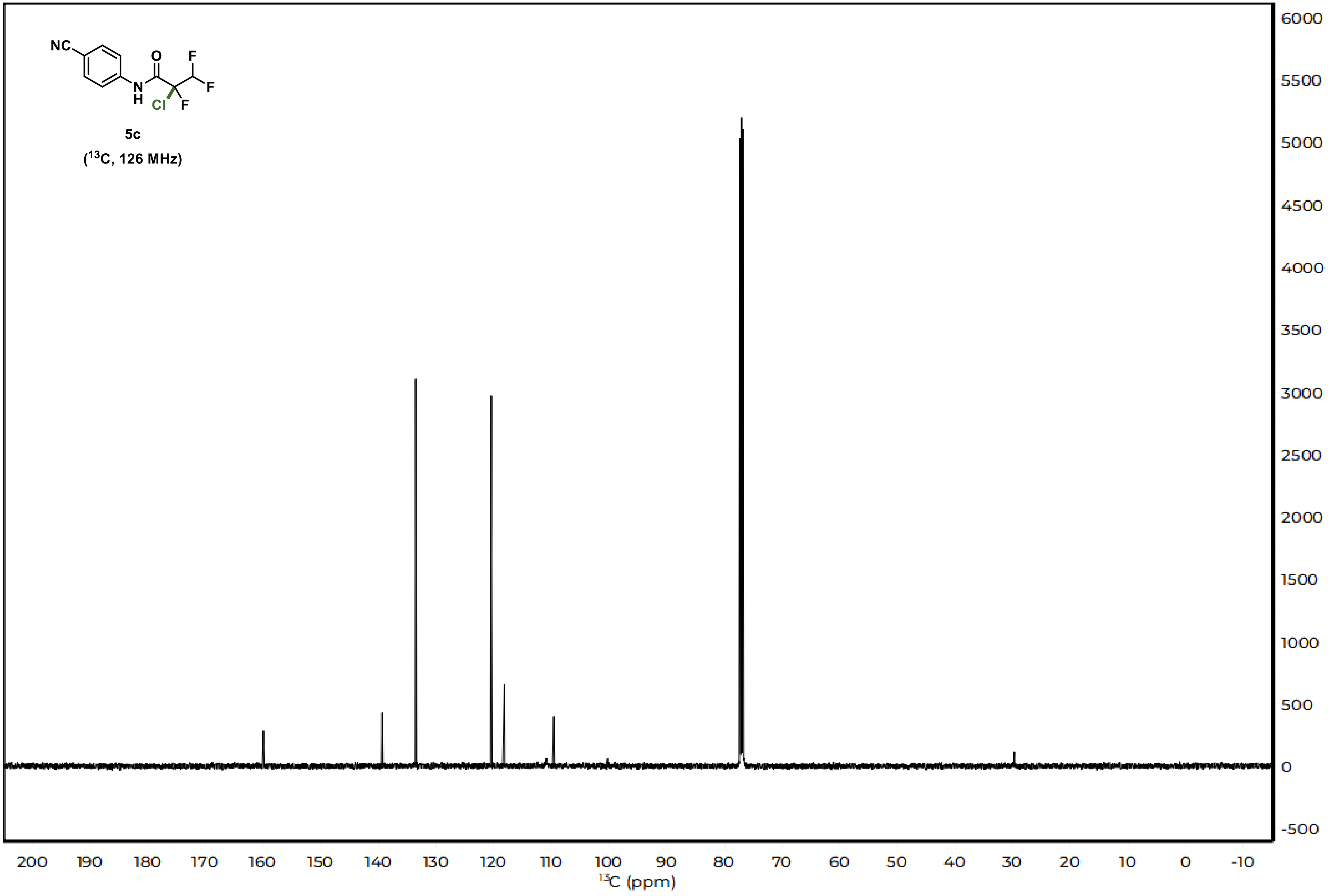
NC
O
F
N
H
Cl
F
F
5c
(13C, 126 MHz)
6000
5500
5000
4500
4000
3500
3000
2500
2000
1500
1000
500
0
-500
200 190 180 170 160 150 140 130 120 110 100 90 80 70 60 50 40 30 20 10 0 -10
13C (ppm)

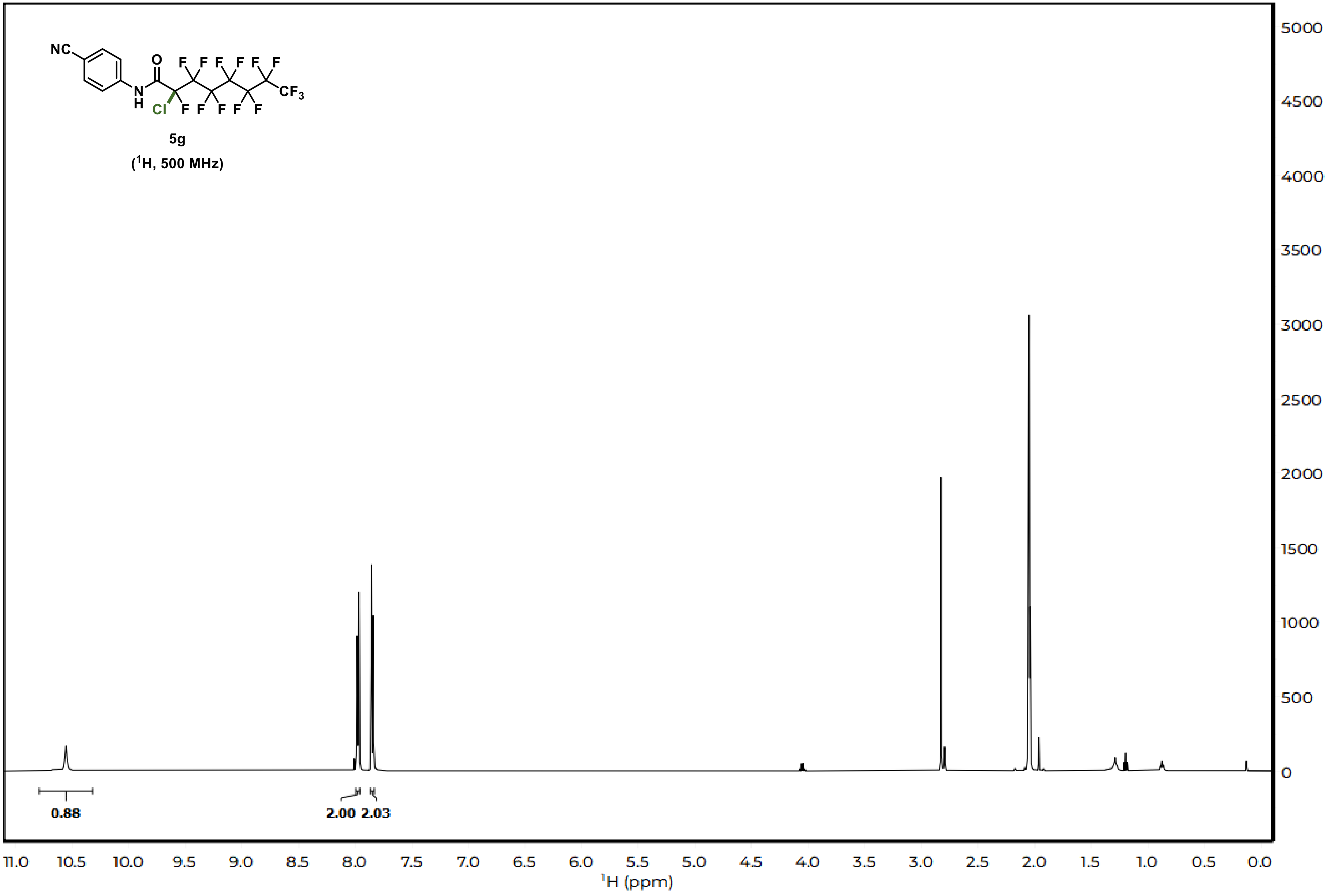

NC
O
F F F F F F
N
H
Cl
F F F F F
CF3
5g
(1H, 500 MHz)
5000
4500
4000
3500
3000
2500
2000
1500
1000
500
0
0.88
2.00 2.03
11.0 10.5 10.0 9.5 9.0 8.5 8.0 7.5 7.0 6.5 6.0 5.5 5.0 4.5 4.0 3.5 3.0 2.5 2.0 1.5 1.0 0.5 0.0
1H (ppm)

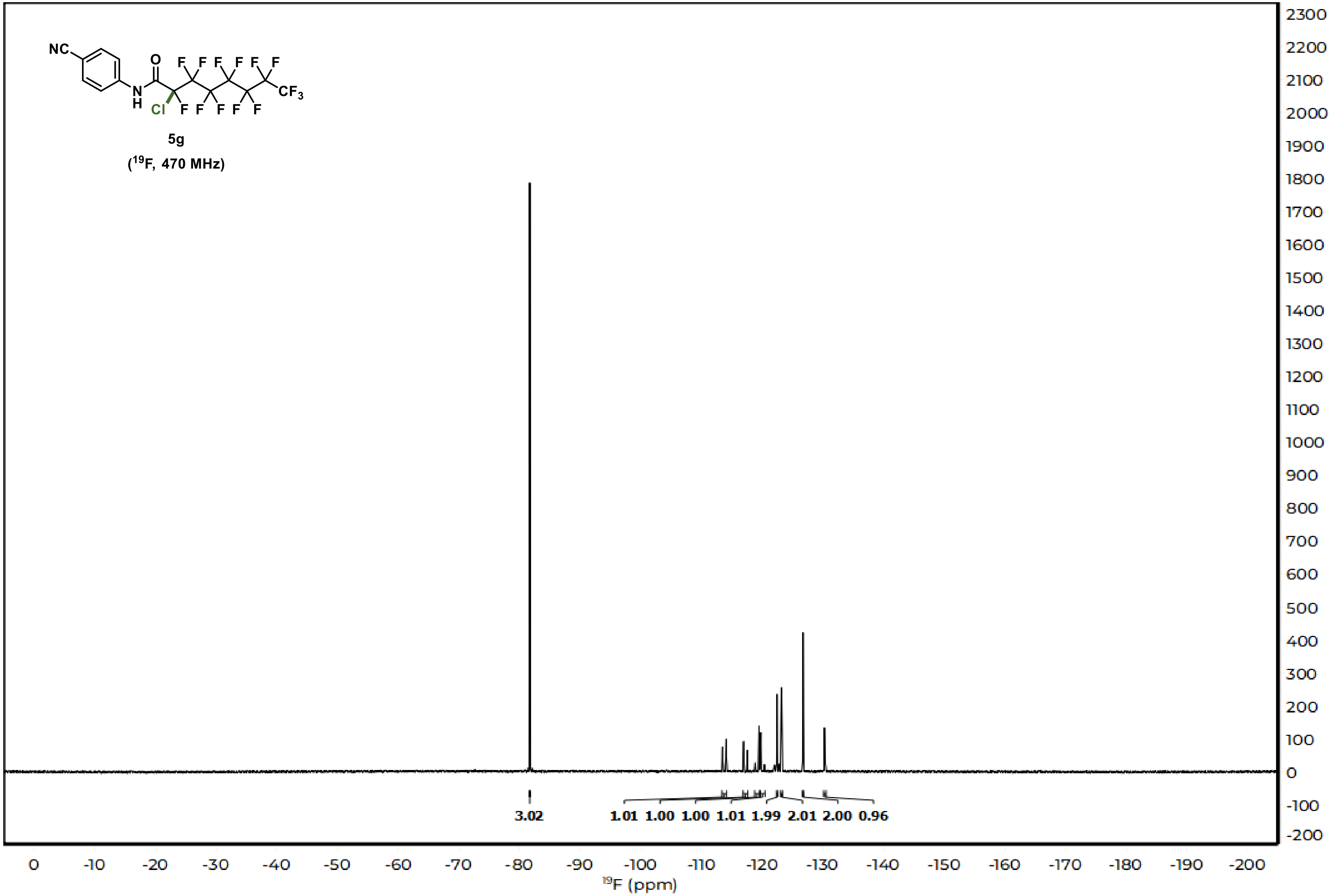
NC
O
F F F F F F
N
H
Cl
F F F F F
CF3
5g
(19F, 470 MHz)
3.02
1.01 1.00 1.00 1.01 1.99 2.01 2.00 0.96
2300
2200
2100
2000
1900
1800
1700
1600
1500
1400
1300
1200
1100
1000
900
800
700
600
500
400
300
200
100
0
-100
-200
0 -10 -20 -30 -40 -50 -60 -70 -80 -90 -100 -110 -120 -130 -140 -150 -160 -170 -180 -190 -200
19F (ppm)

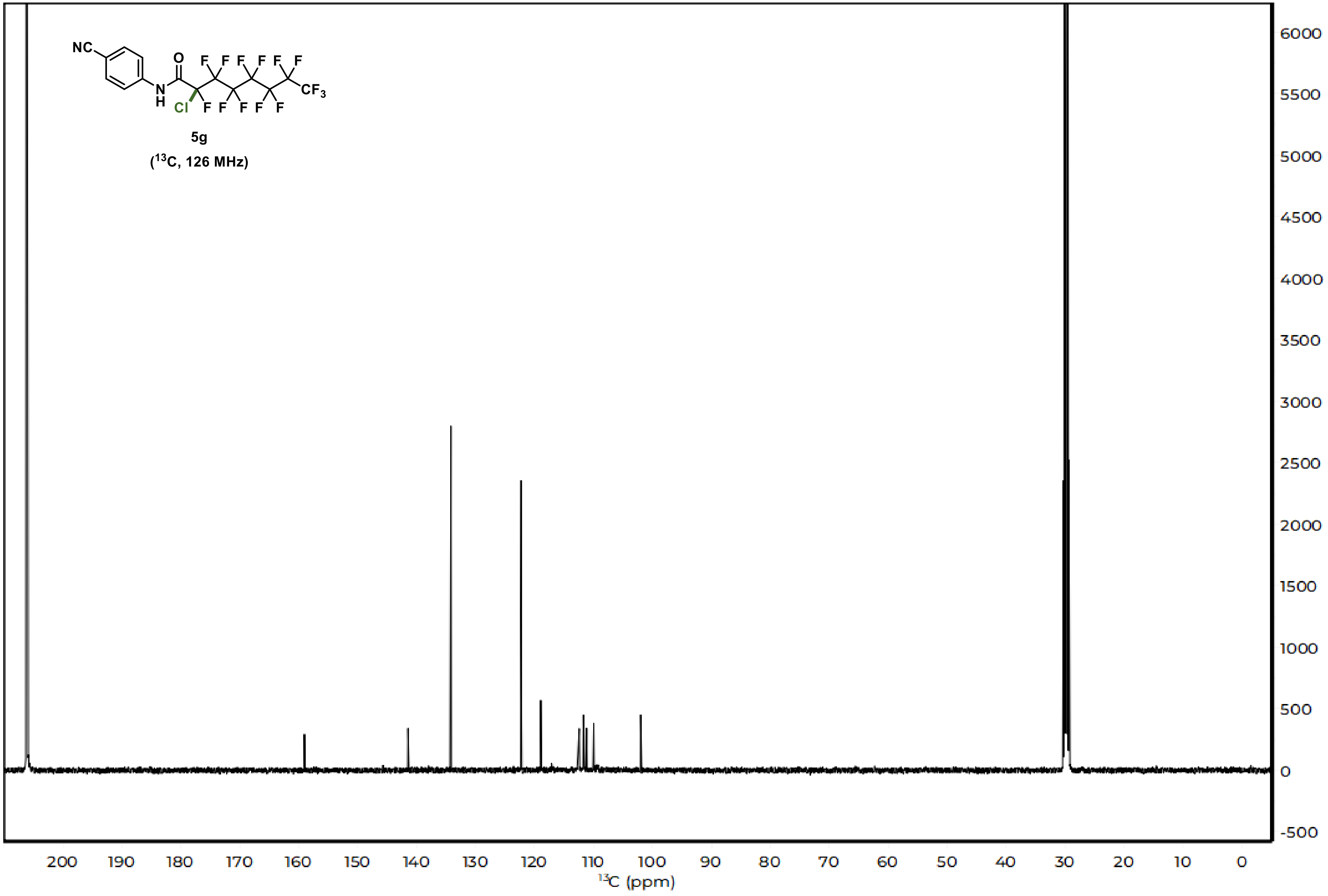
NC
O
N
H
Cl
F F F F F F
F F F F F
CF3
5g
(13C, 126 MHz)
6000
5500
5000
4500
4000
3500
3000
2500
2000
1500
1000
500
0
-500
200 190 180 170 160 150 140 130 120 110 100 90 80 70 60 50 40 30 20 10 0
13C (ppm)

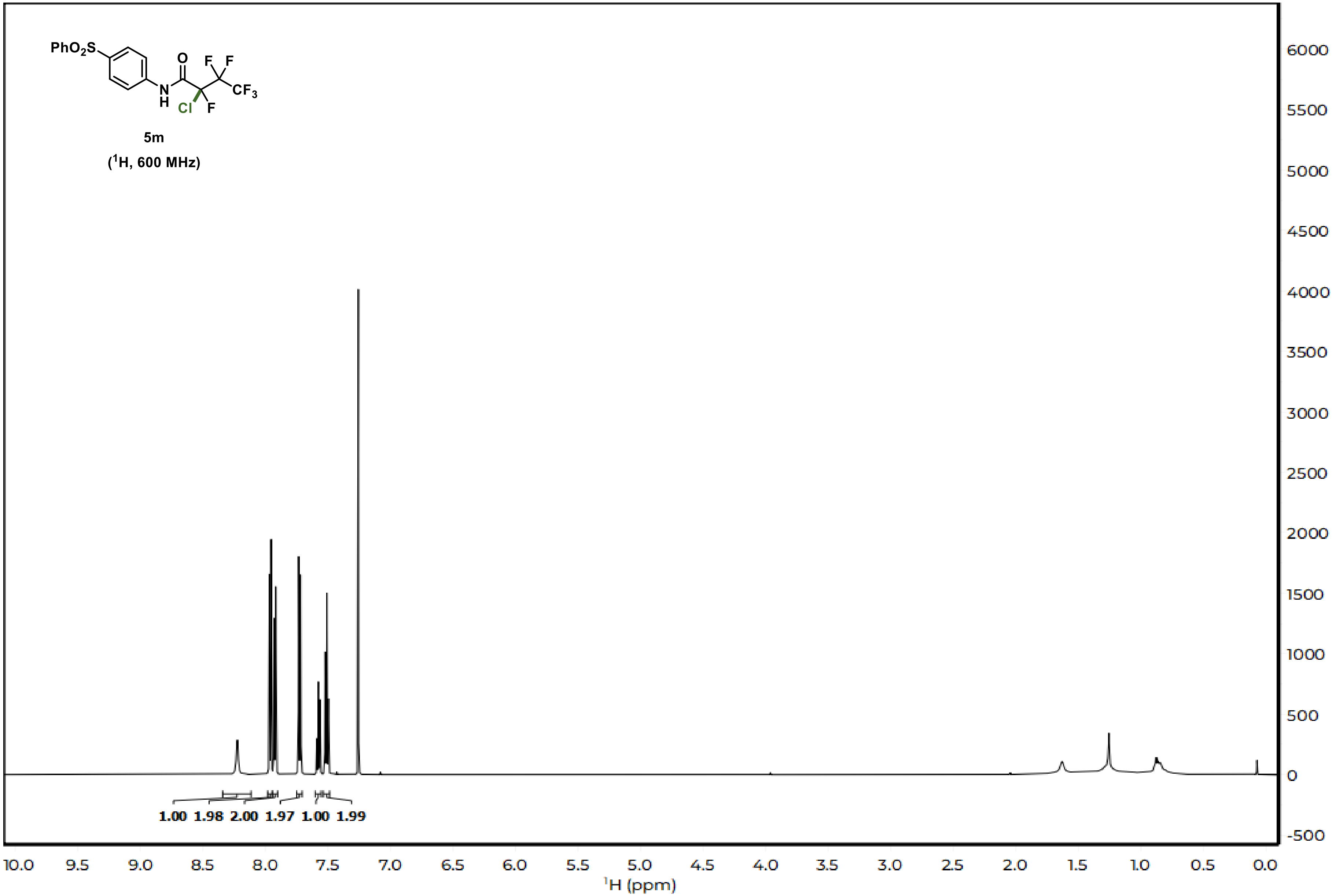

PhO2S
O
F F
N
H
Cl
F
CF3
5m
(1H, 600 MHz)
1.00 1.98 2.00 1.97 1.00 1.99
6000
5500
5000
4500
4000
3500
3000
2500
2000
1500
1000
500
0
-500
10.0 9.5 9.0 8.5 8.0 7.5 7.0 6.5 6.0 5.5 5.0 4.5 4.0 3.5 3.0 2.5 2.0 1.5 1.0 0.5 0.0
1H (ppm)

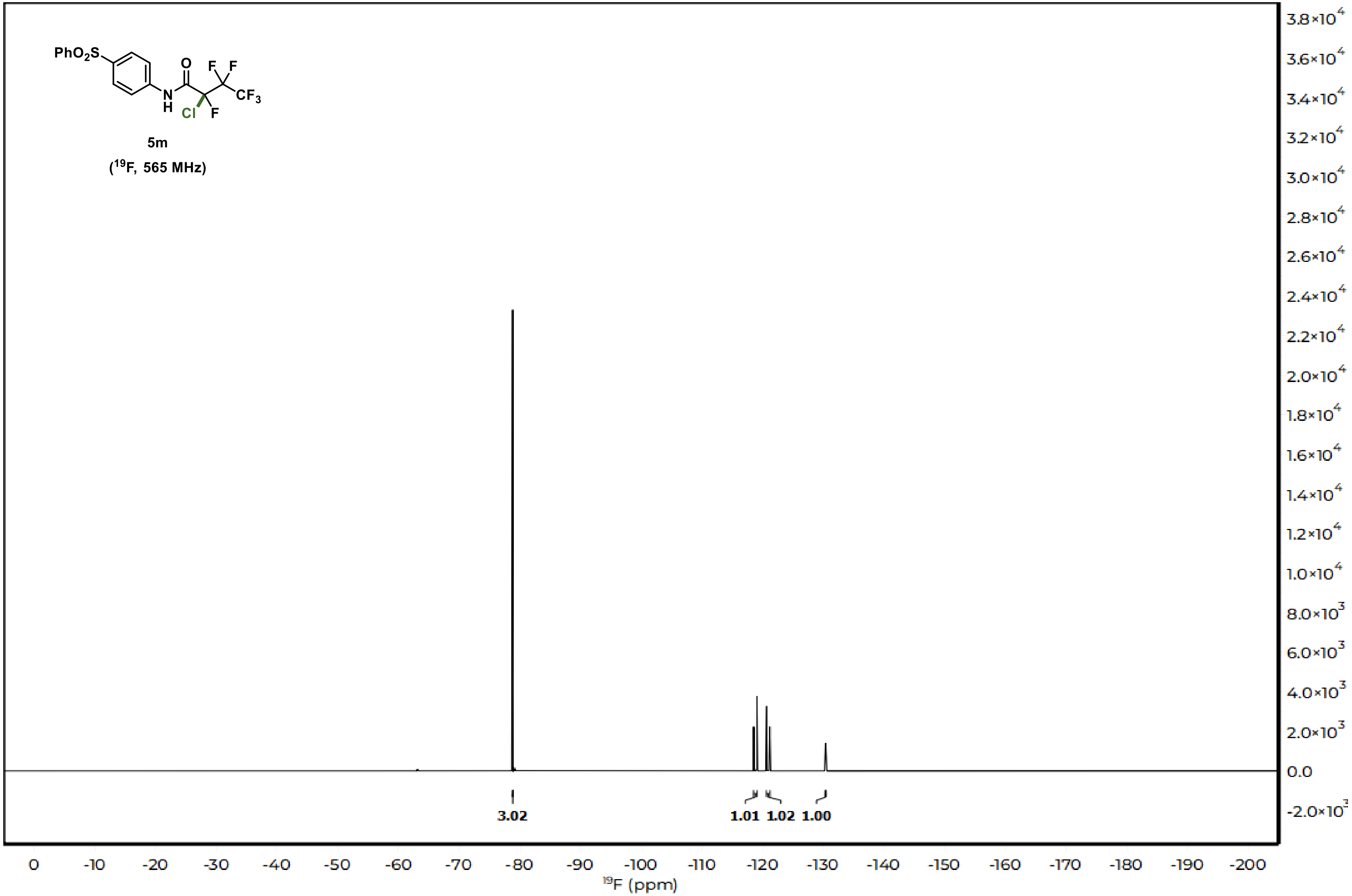

PhO2S
N
H
O
F
F
CF3
Cl
F
5m
(19F, 565 MHz)
3.02
1.01
1.02
1.00
3.8×10^4
3.6×10^4
3.4×10^4
3.2×10^4
3.0×10^4
2.8×10^4
2.6×10^4
2.4×10^4
2.2×10^4
2.0×10^4
1.8×10^4
1.6×10^4
1.4×10^4
1.2×10^4
1.0×10^4
8.0×10^3
6.0×10^3
4.0×10^3
2.0×10^3
0.0
-2.0×10^3
0
-10
-20
-30
-40
-50
-60
-70
-80
-90
-100
-110
-120
-130
-140
-150
-160
-170
-180
-190
-200
19F (ppm)

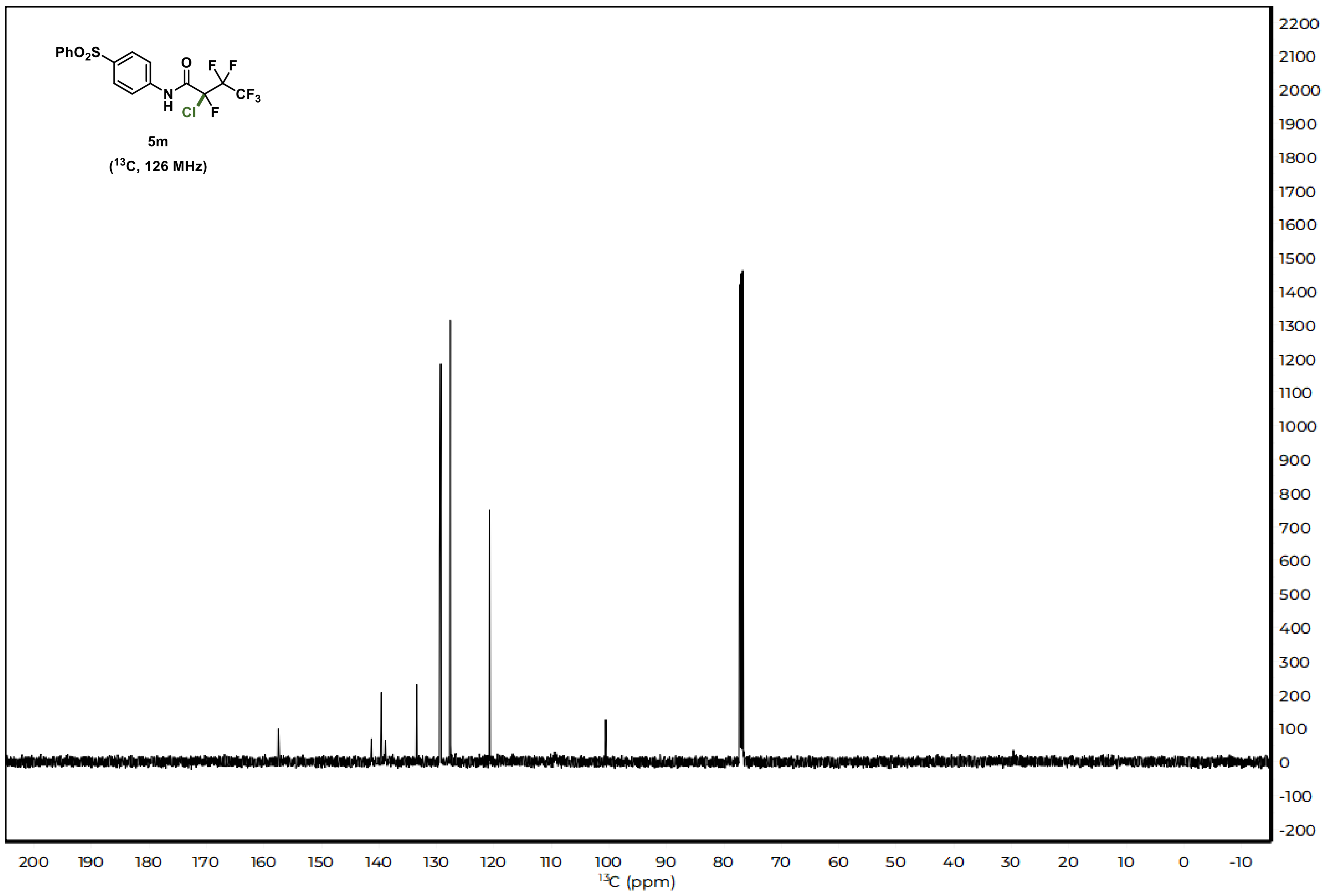
PhO2S
O
F F
N
H
Cl
F
CF3
5m
(13C, 126 MHz)
13C (ppm)

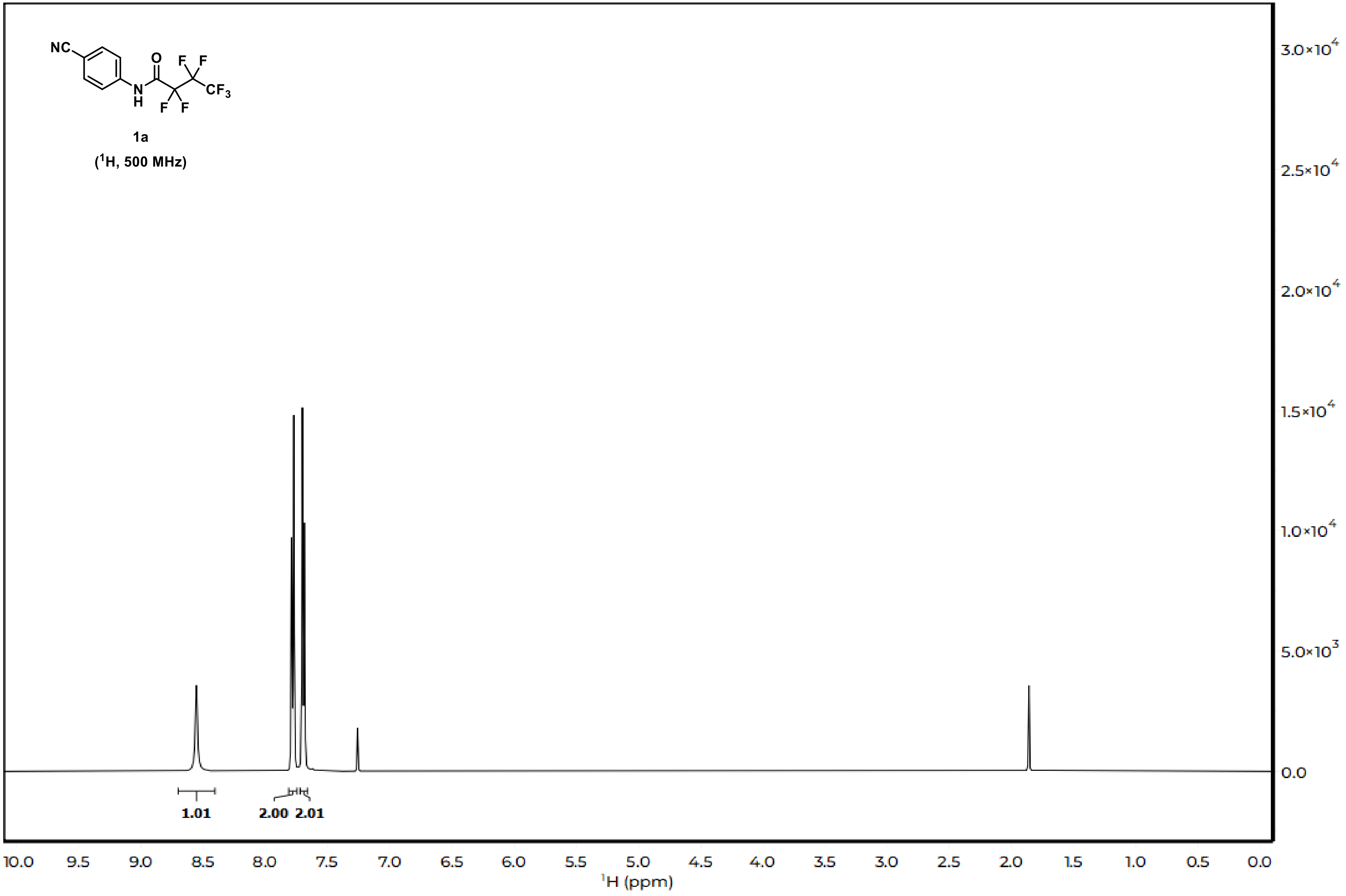
NC
O
F F
N
H
CF3
F F
1a
(1H, 500 MHz)
3.0×10^4
2.5×10^4
2.0×10^4
1.5×10^4
1.0×10^4
5.0×10^3
0.0
1.01
2.00 2.01
10.0 9.5 9.0 8.5 8.0 7.5 7.0 6.5 6.0 5.5 5.0 4.5 4.0 3.5 3.0 2.5 2.0 1.5 1.0 0.5 0.0
1H (ppm)

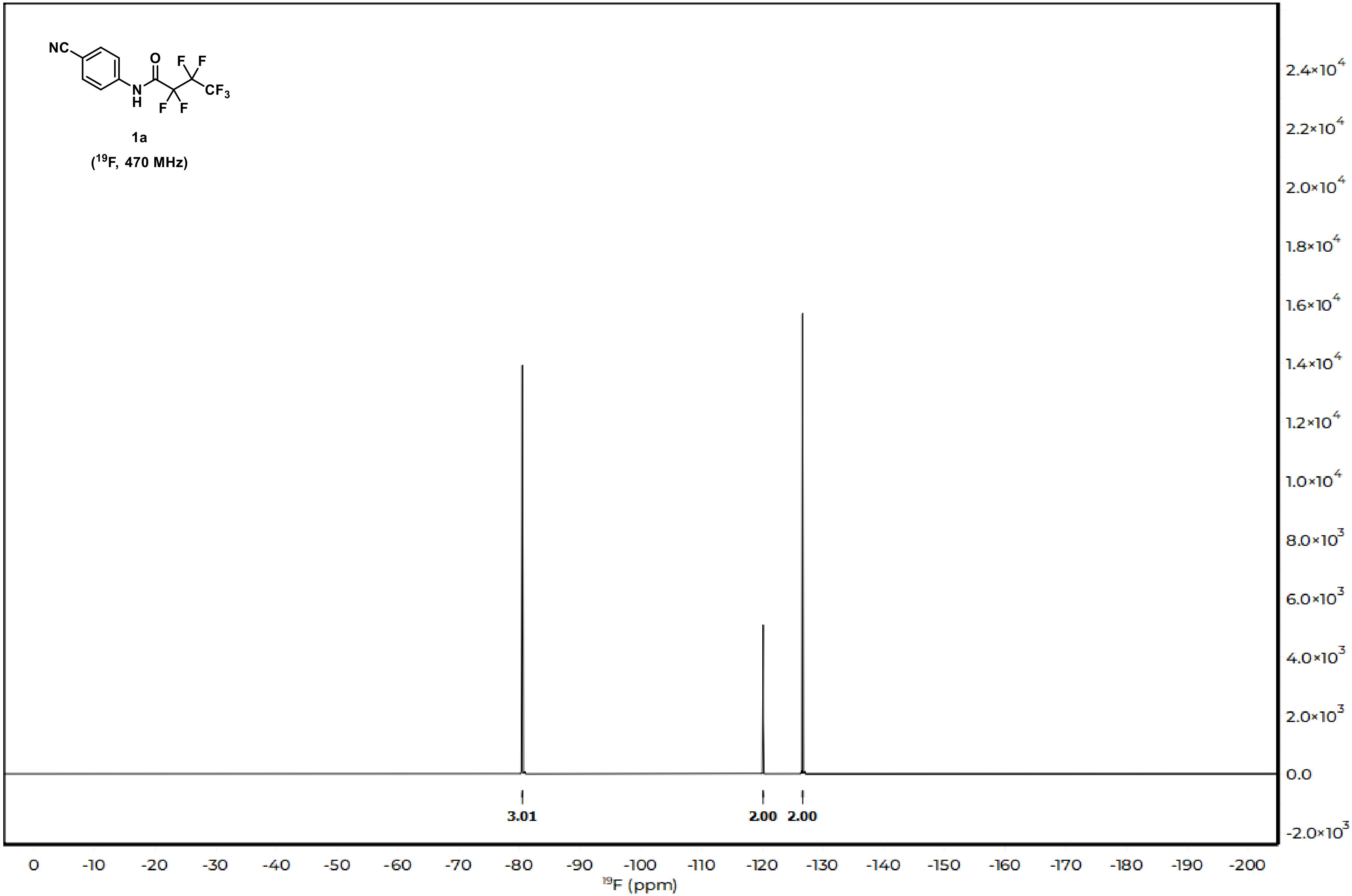
NC
O
F F
CF3
N
H
F F
1a
(19F, 470 MHz)
2.4×10^4
2.2×10^4
2.0×10^4
1.8×10^4
1.6×10^4
1.4×10^4
1.2×10^4
1.0×10^4
8.0×10^3
6.0×10^3
4.0×10^3
2.0×10^3
0.0
-2.0×10^3
3.01
2.00
2.00
0 -10 -20 -30 -40 -50 -60 -70 -80 -90 -100 -110 -120 -130 -140 -150 -160 -170 -180 -190 -200
19F (ppm)

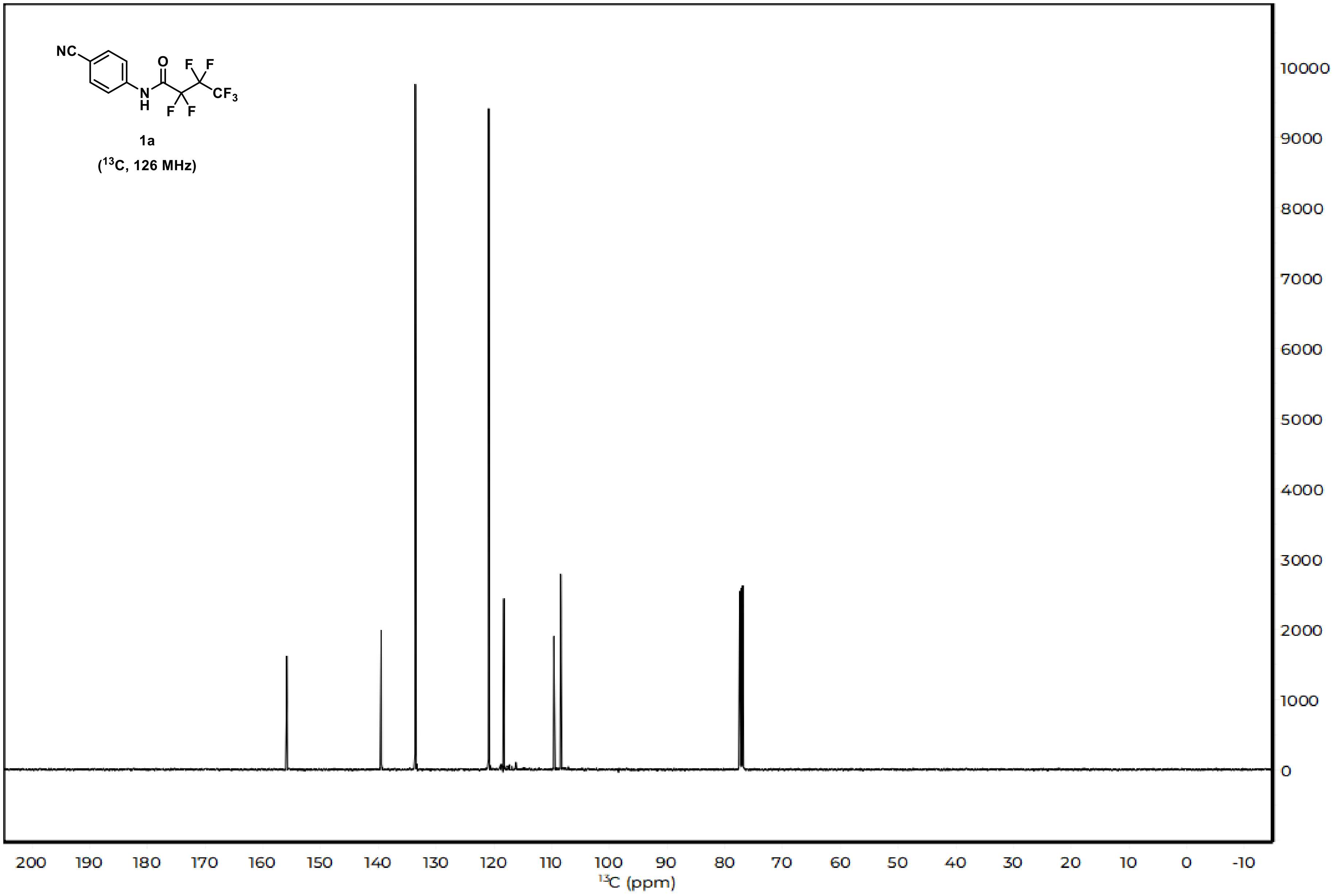
NC
O
F
F
CF3
N
H
F
F
1a
(13C, 126 MHz)
10000
9000
8000
7000
6000
5000
4000
3000
2000
1000
0
200
190
180
170
160
150
140
130
120
110
100
90
80
70
60
50
40
30
20
10
0
-10
13C (ppm)

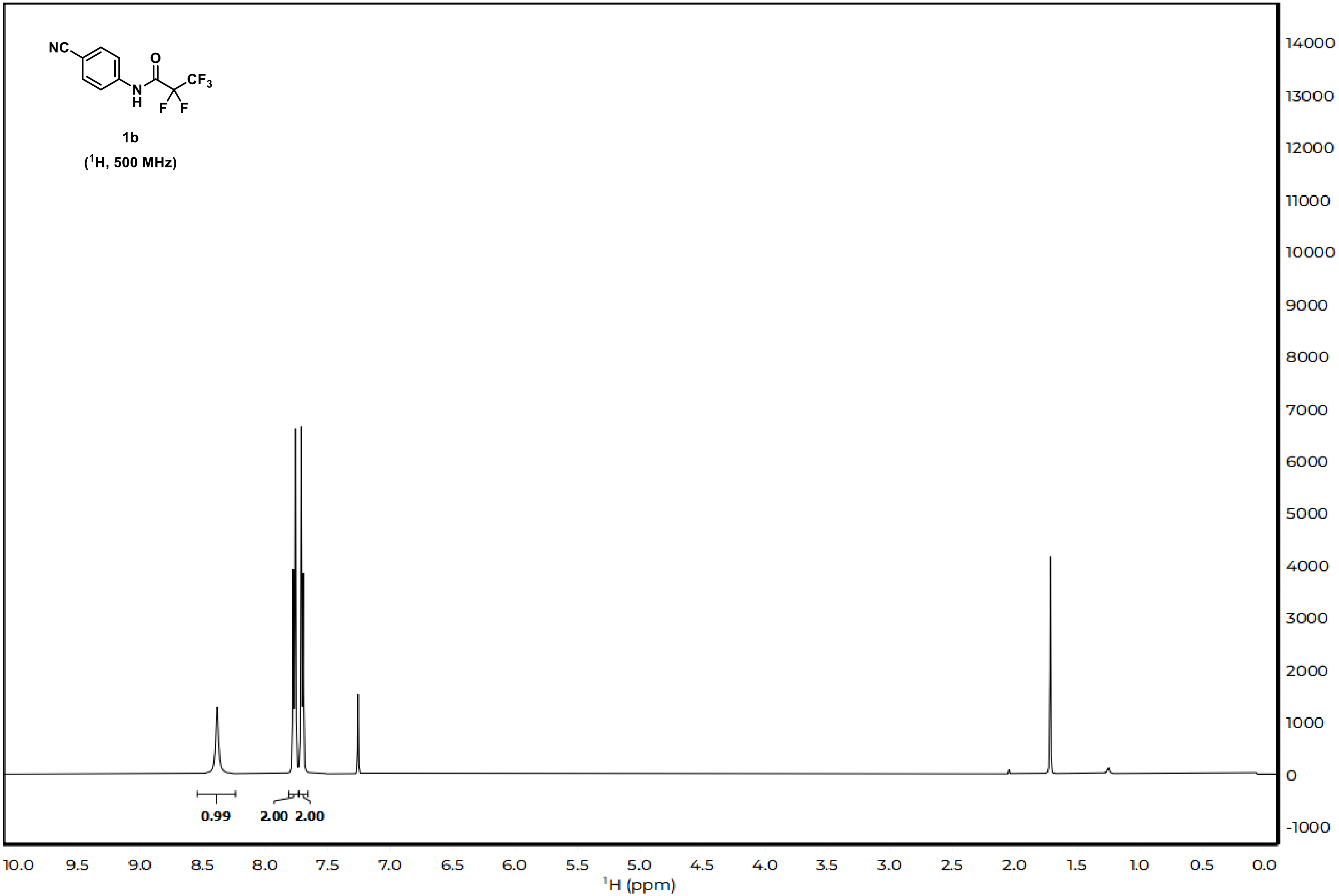

NC
O
CF3
N
H
F F
1b
(1H, 500 MHz)
14000
13000
12000
11000
10000
9000
8000
7000
6000
5000
4000
3000
2000
1000
0
-1000
0.99
2.00 2.00
10.0
9.5
9.0
8.5
8.0
7.5
7.0
6.5
6.0
5.5
5.0
4.5
4.0
3.5
3.0
2.5
2.0
1.5
1.0
0.5
0.0
1H (ppm)

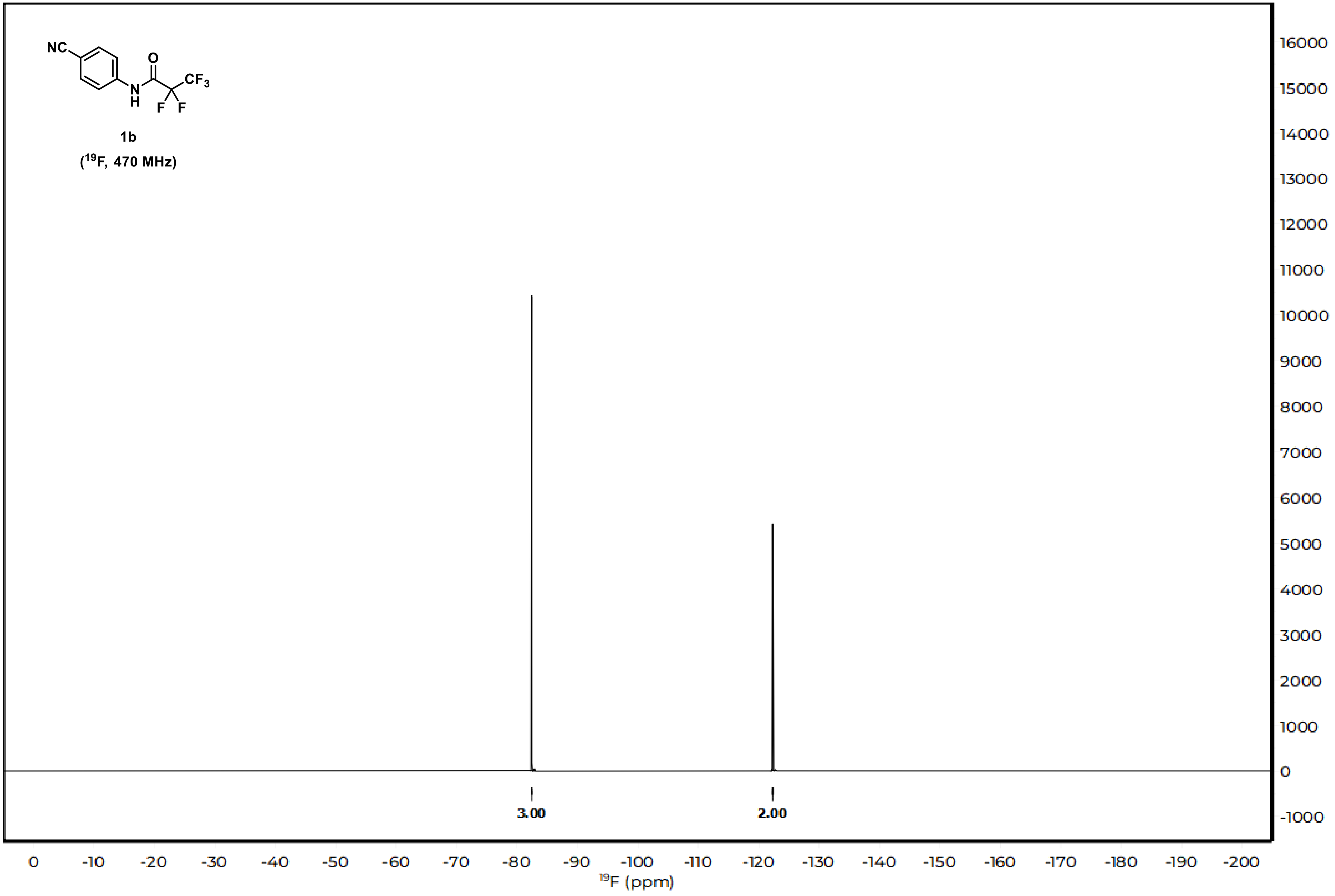
NC
O
CF3
N
H
F F
1b
(19F, 470 MHz)
16000
15000
14000
13000
12000
11000
10000
9000
8000
7000
6000
5000
4000
3000
2000
1000
0
-1000
3.00
2.00
0
-10
-20
-30
-40
-50
-60
-70
-80
-90
-100
-110
-120
-130
-140
-150
-160
-170
-180
-190
-200
19F (ppm)

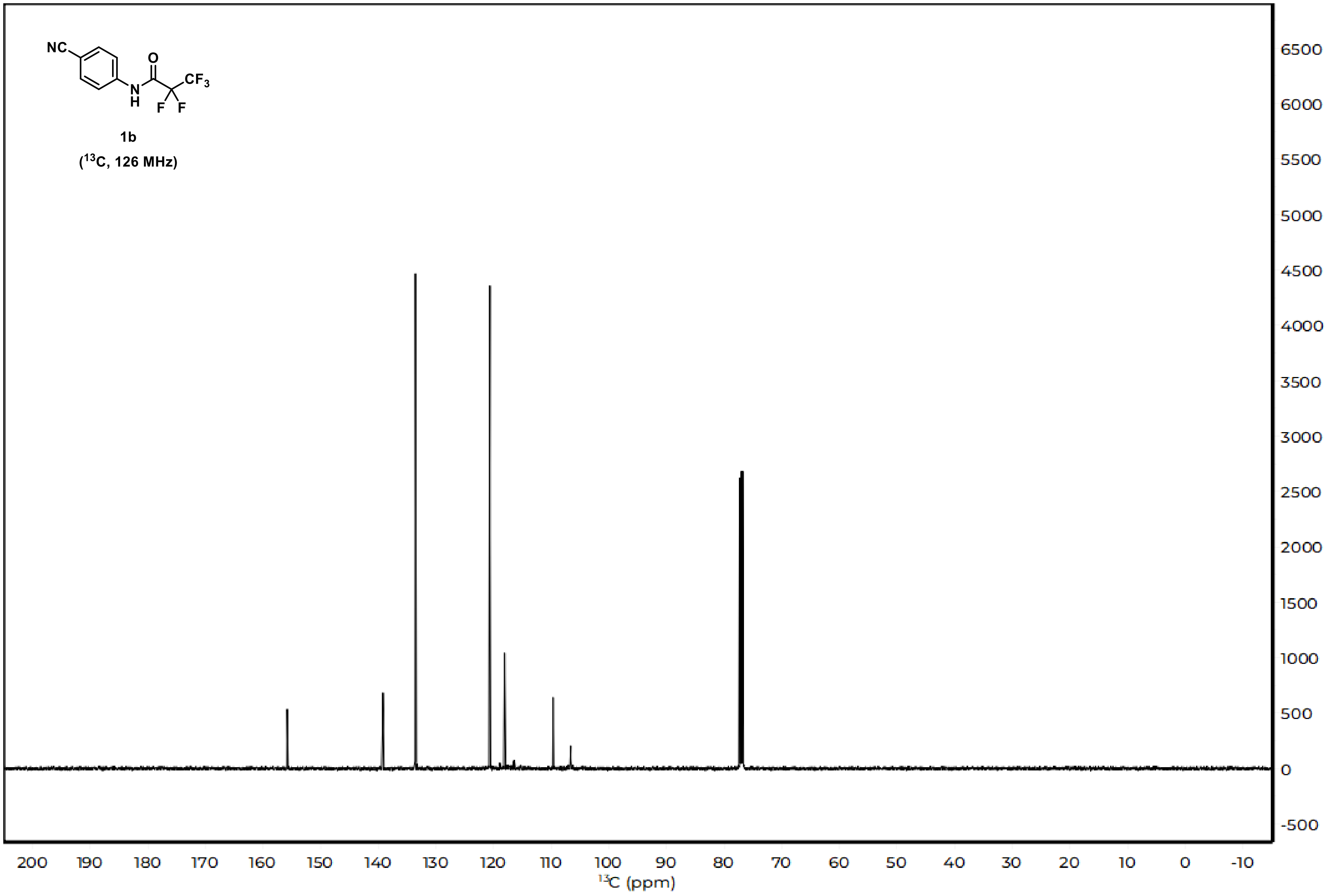
NC
O
N
H
CF3
F
F
1b
(13C, 126 MHz)
6500
6000
5500
5000
4500
4000
3500
3000
2500
2000
1500
1000
500
0
-500
200
190
180
170
160
150
140
130
120
110
100
90
80
70
60
50
40
30
20
10
0
-10
13C (ppm)

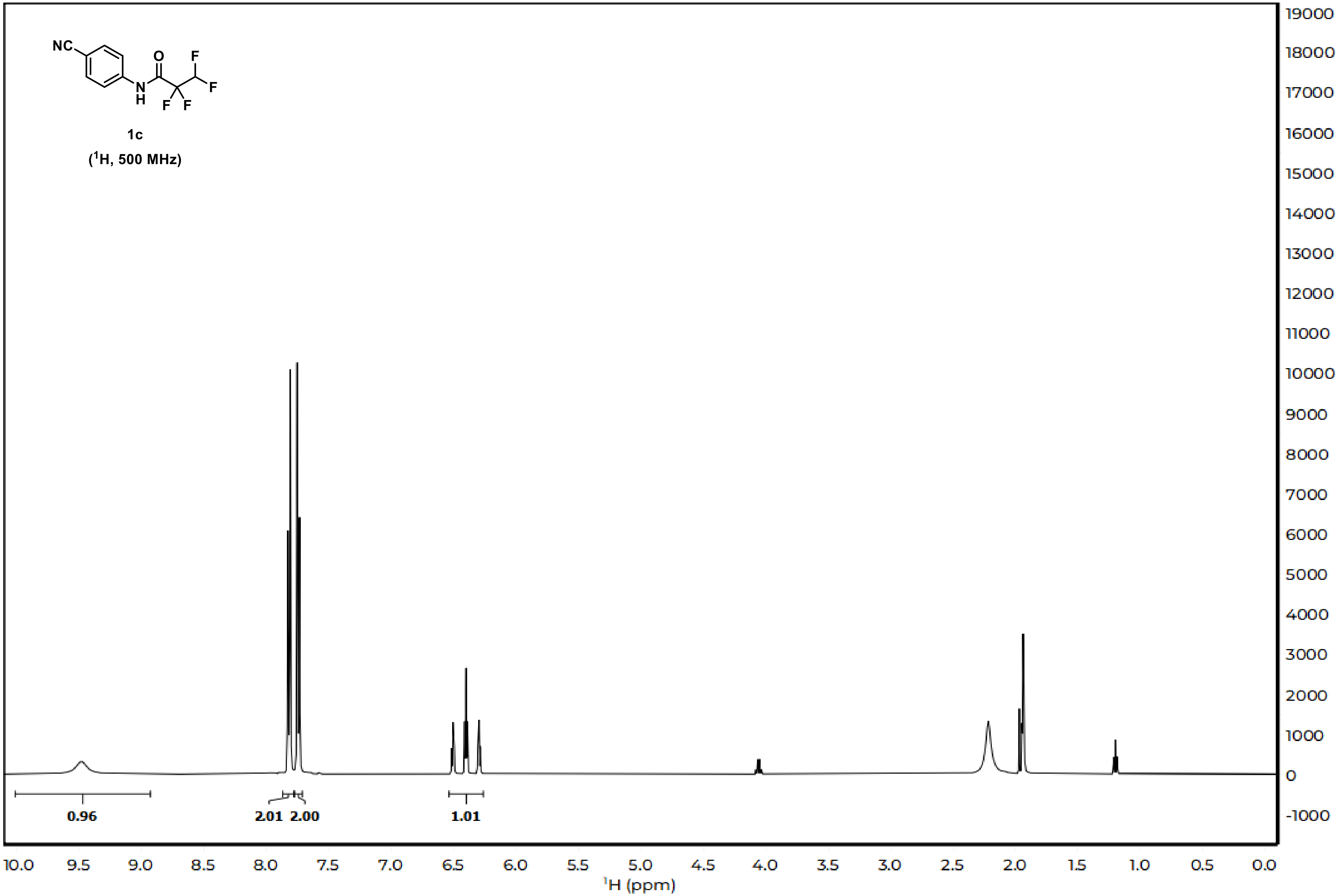

NC
O
F
F
N
H
F
F
1c
(¹H, 500 MHz)
19000
18000
17000
16000
15000
14000
13000
12000
11000
10000
9000
8000
7000
6000
5000
4000
3000
2000
1000
0
-1000
0.96
2.01
2.00
1.01
10.0
9.5
9.0
8.5
8.0
7.5
7.0
6.5
6.0
5.5
5.0
4.5
4.0
3.5
3.0
2.5
2.0
1.5
1.0
0.5
0.0
¹H (ppm)

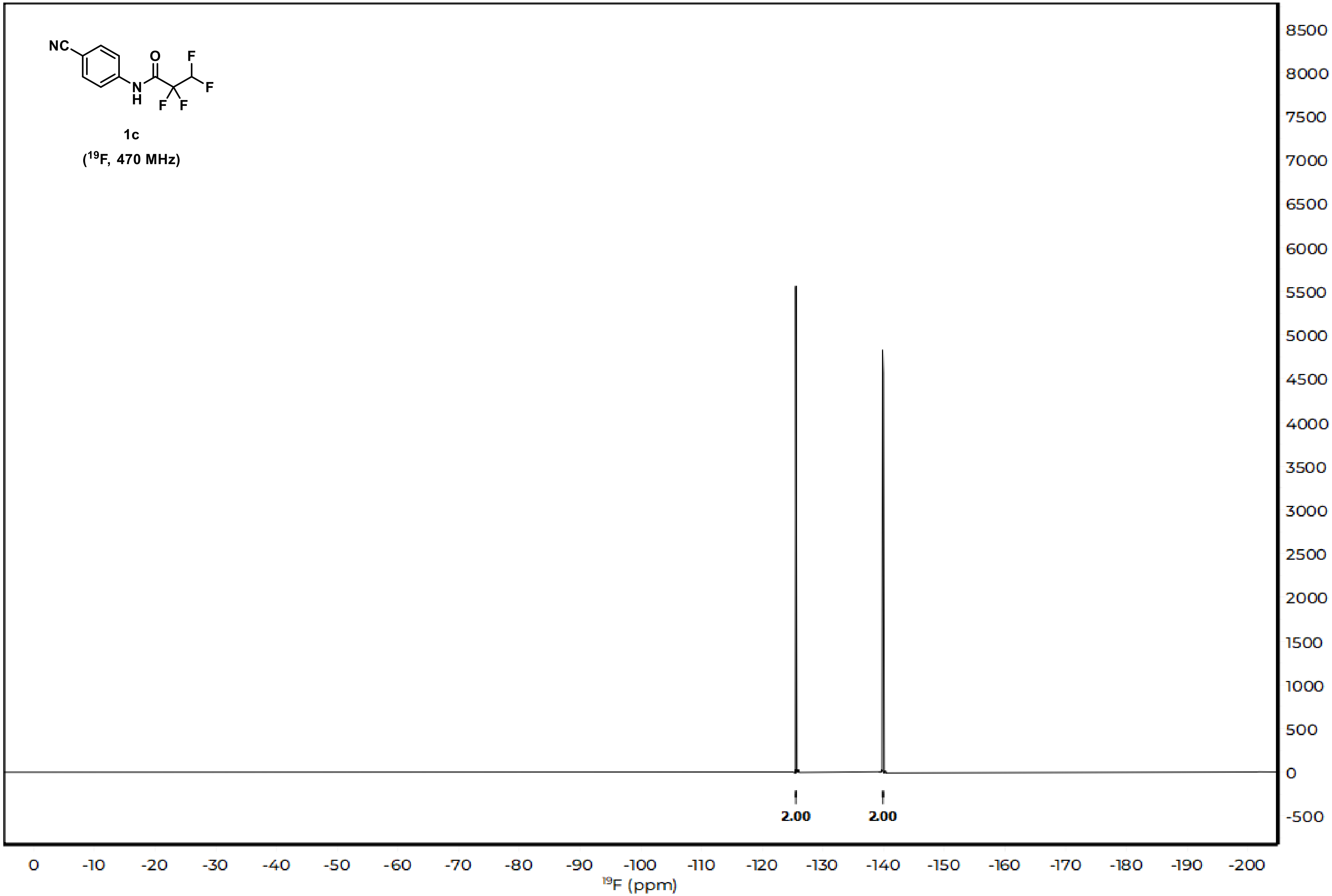
NC
O
F
F
N
H
F
F
1c
(19F, 470 MHz)
8500
8000
7500
7000
6500
6000
5500
5000
4500
4000
3500
3000
2500
2000
1500
1000
500
0
-500
2.00
2.00
0
-10
-20
-30
-40
-50
-60
-70
-80
-90
-100
-110
-120
-130
-140
-150
-160
-170
-180
-190
-200
19F (ppm)

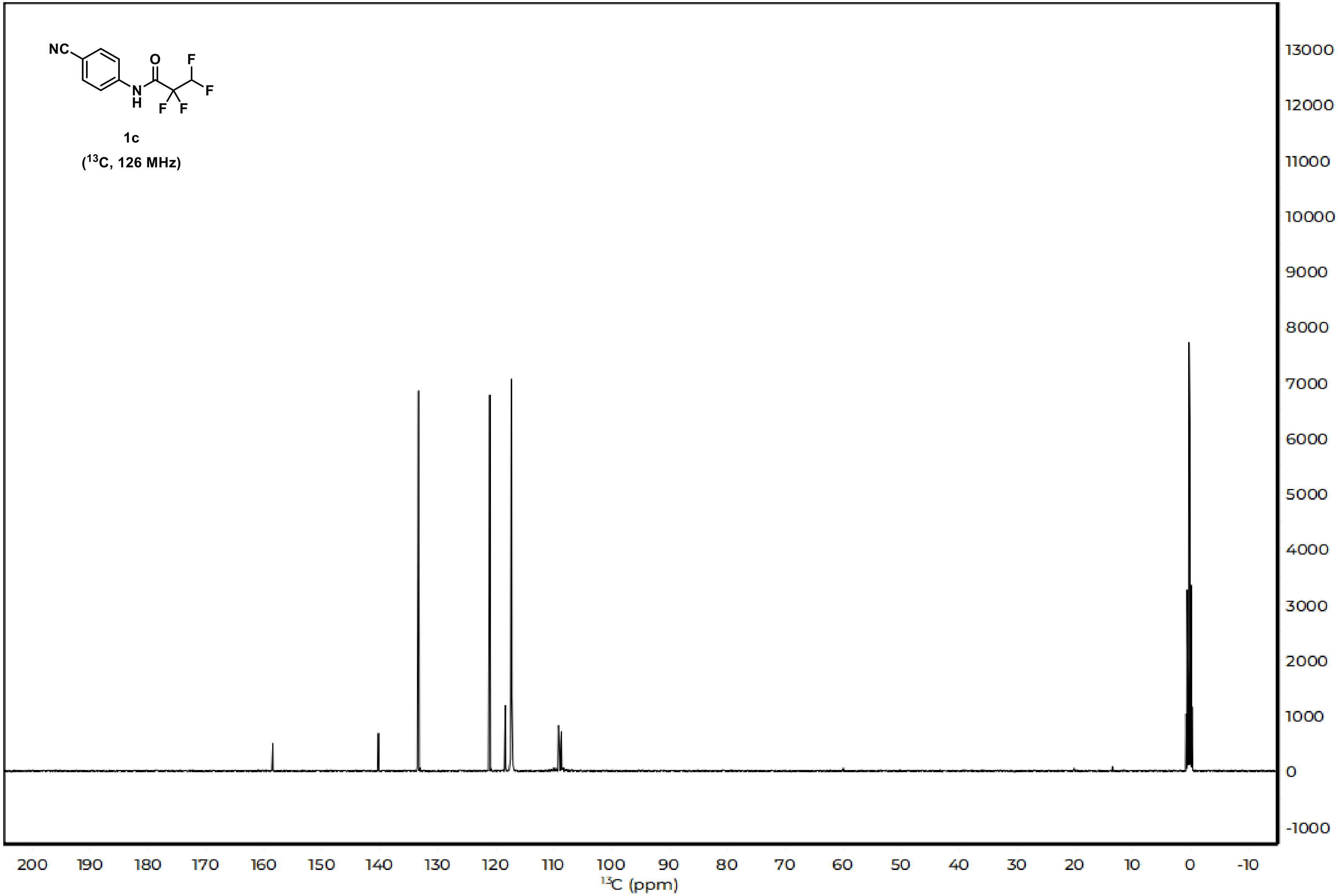
NC
O
F
F
N
H
F F
1c
(13C, 126 MHz)
13000
12000
11000
10000
9000
8000
7000
6000
5000
4000
3000
2000
1000
0
-1000
200 190 180 170 160 150 140 130 120 110 100 90 80 70 60 50 40 30 20 10 0 -10
13C (ppm)

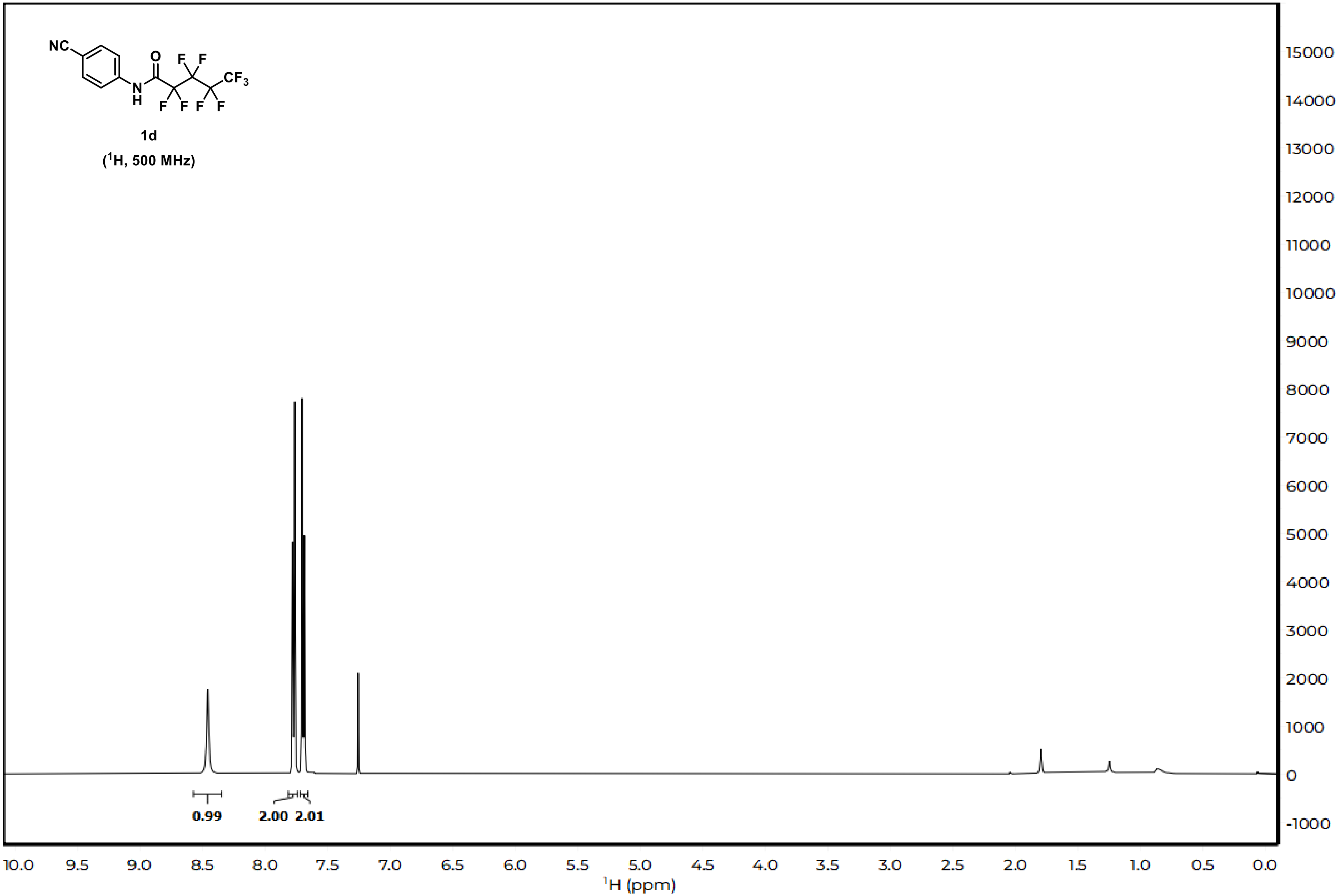

NC
O
F F
CF3
N
H
F F F F
1d
(1H, 500 MHz)
15000
14000
13000
12000
11000
10000
9000
8000
7000
6000
5000
4000
3000
2000
1000
0
-1000
0.99
2.00 2.01
10.0 9.5 9.0 8.5 8.0 7.5 7.0 6.5 6.0 5.5 5.0 4.5 4.0 3.5 3.0 2.5 2.0 1.5 1.0 0.5 0.0
1H (ppm)

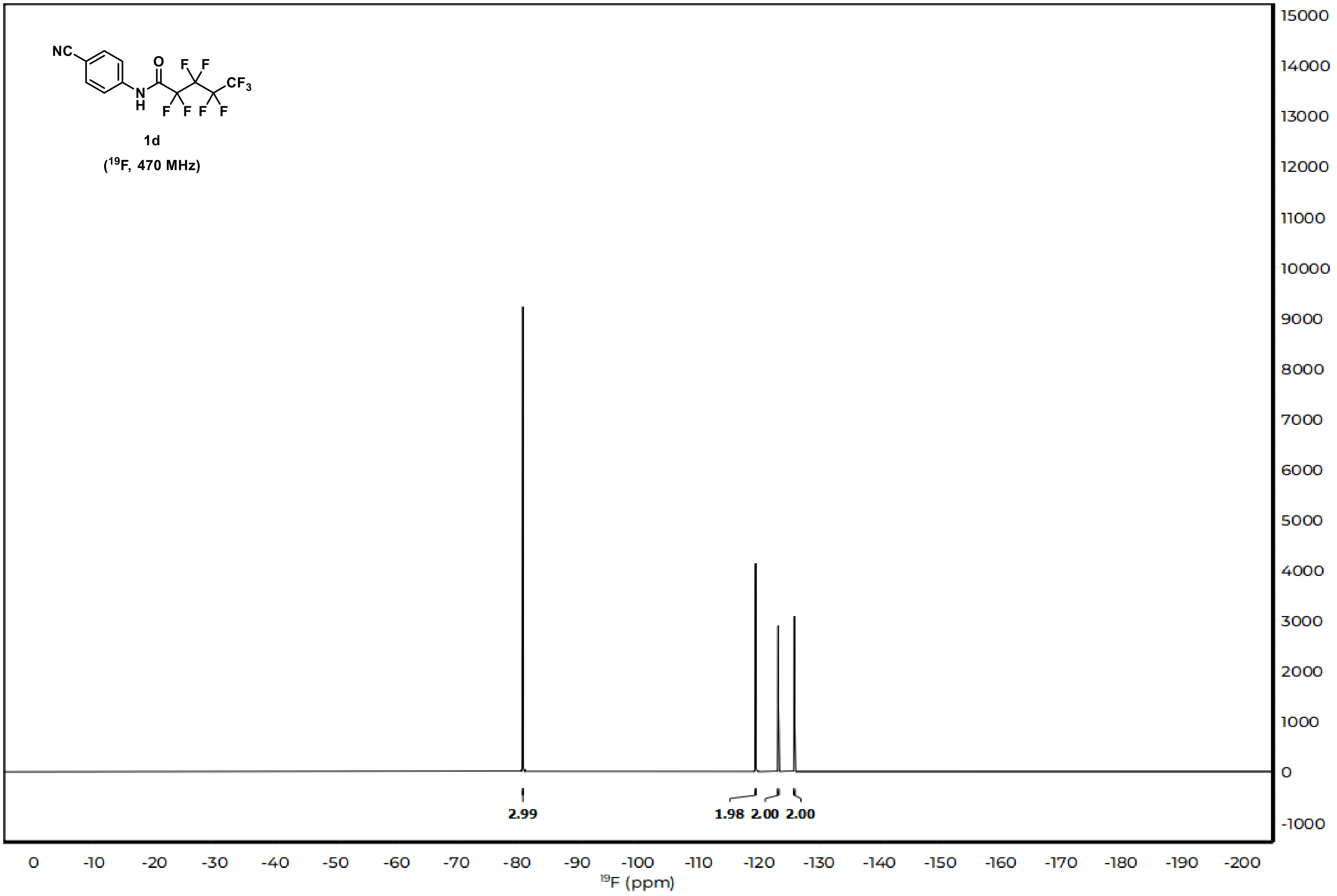
NC
O
F F
CF3
N
H
F F F F
1d
(19F, 470 MHz)
15000
14000
13000
12000
11000
10000
9000
8000
7000
6000
5000
4000
3000
2000
1000
0
-1000
2.99
1.98 2.00 2.00
0 -10 -20 -30 -40 -50 -60 -70 -80 -90 -100 -110 -120 -130 -140 -150 -160 -170 -180 -190 -200
19F (ppm)

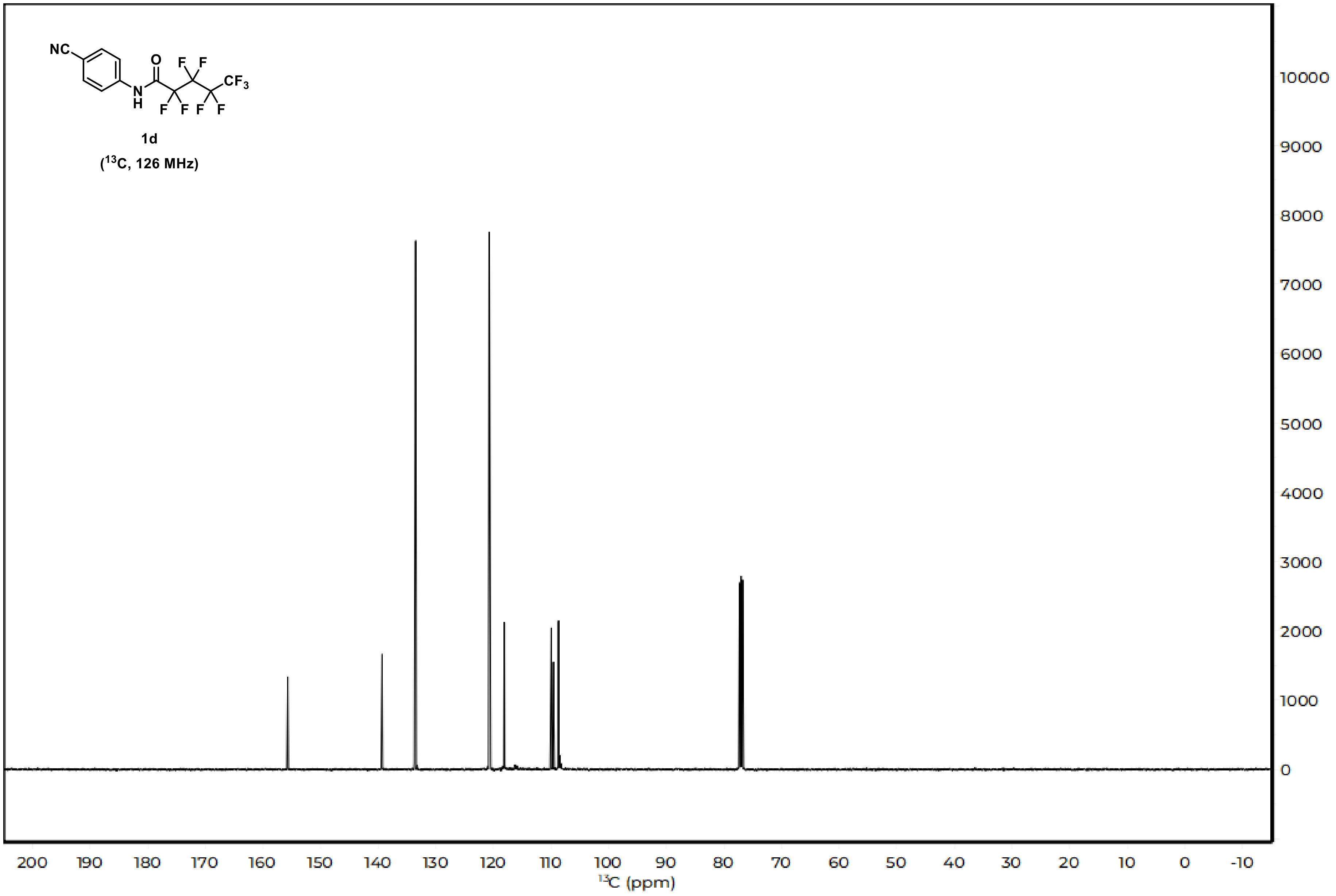
NC
O
F F
CF3
N
H
F F F F
1d
(13C, 126 MHz)
10000
9000
8000
7000
6000
5000
4000
3000
2000
1000
0
200 190 180 170 160 150 140 130 120 110 100 90 80 70 60 50 40 30 20 10 0 -10
13C (ppm)

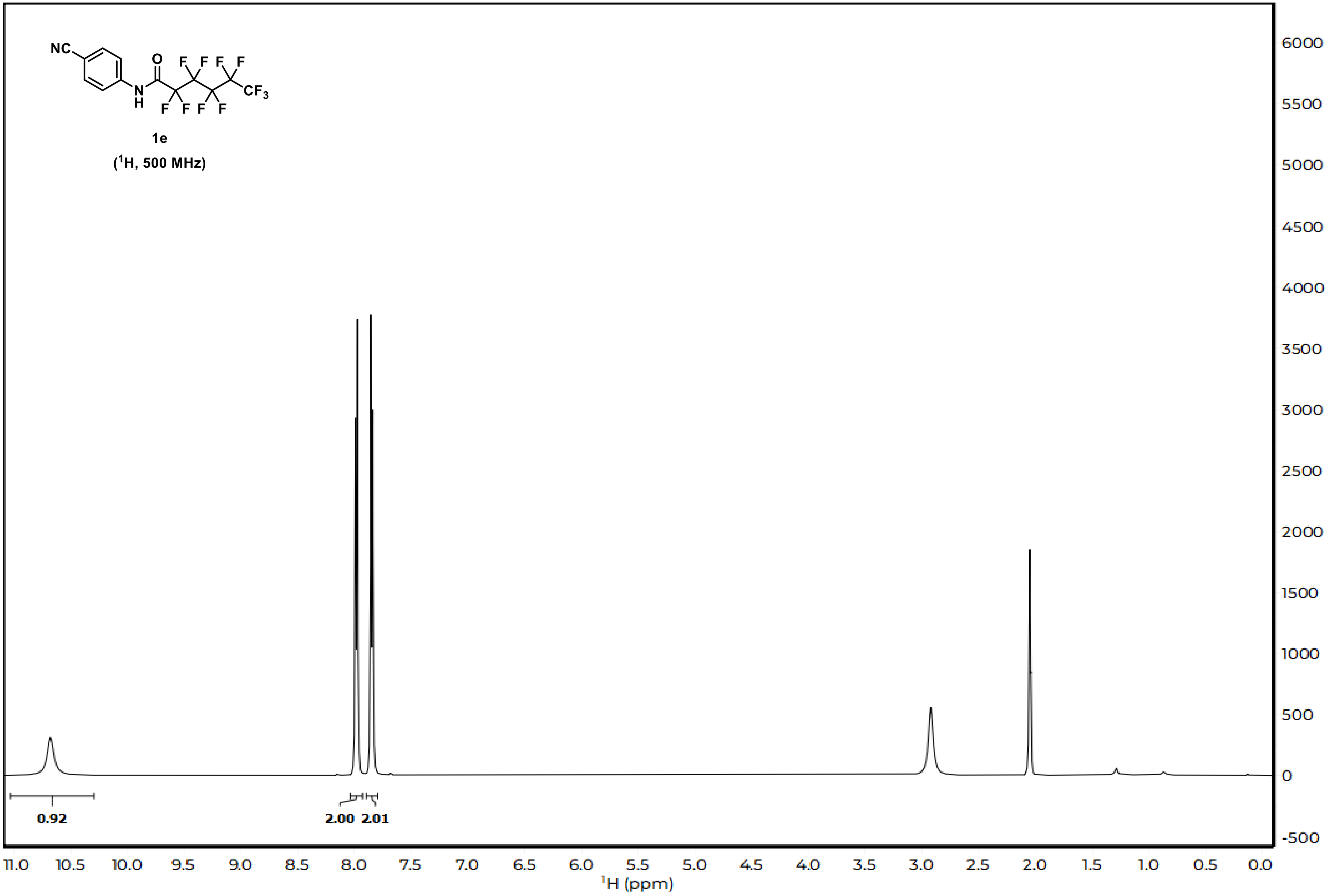

NC
O
F F F F
N
H
F F F F
CF_3
1e
(^{1}H, 500 MHz)
6000
5500
5000
4500
4000
3500
3000
2500
2000
1500
1000
500
0
-500
0.92
2.00 2.01
11.0 10.5 10.0 9.5 9.0 8.5 8.0 7.5 7.0 6.5 6.0 5.5 5.0 4.5 4.0 3.5 3.0 2.5 2.0 1.5 1.0 0.5 0.0
^{1}H (ppm)

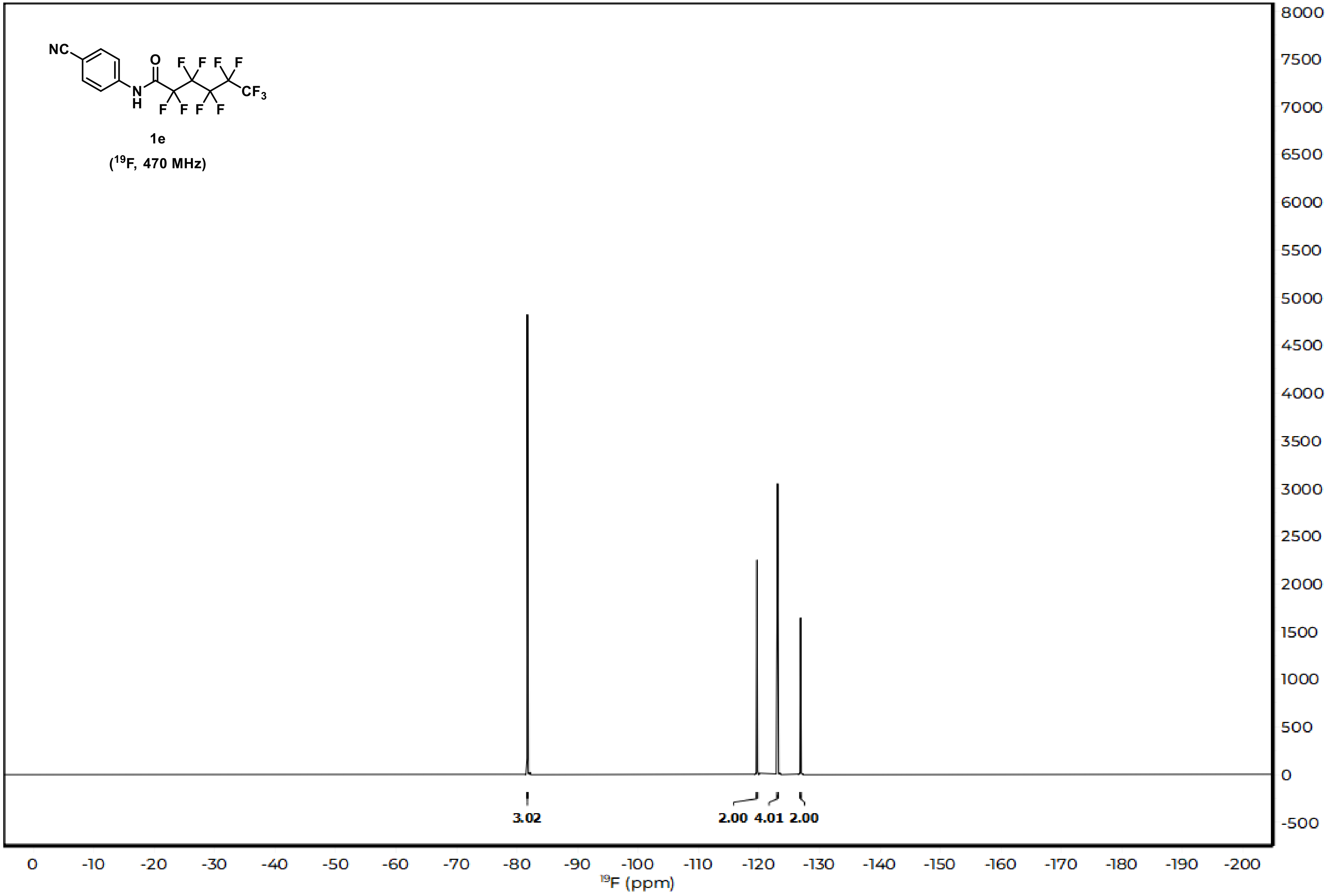
NC
O
N
H
F F F F
F F F F
CF3
1e
(19F, 470 MHz)
3.02
2.00 4.01 2.00
8000
7500
7000
6500
6000
5500
5000
4500
4000
3500
3000
2500
2000
1500
1000
500
0
-500
0 -10 -20 -30 -40 -50 -60 -70 -80 -90 -100 -110 -120 -130 -140 -150 -160 -170 -180 -190 -200
19F (ppm)

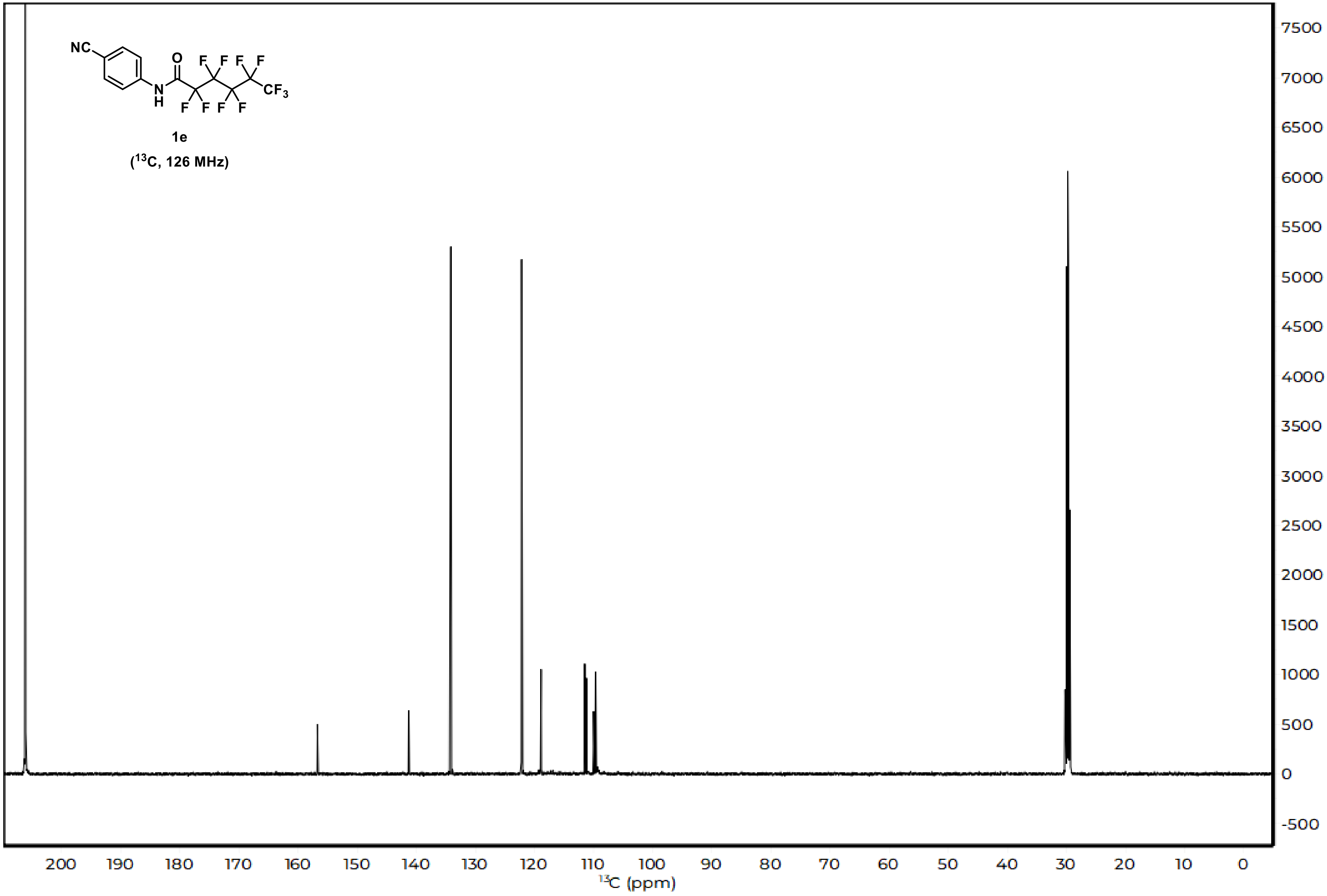
NC
O
N
H
F
CF3
1e
(13C, 126 MHz)
7500
7000
6500
6000
5500
5000
4500
4000
3500
3000
2500
2000
1500
1000
500
0
-500
200
190
180
170
160
150
140
130
120
110
100
90
80
70
60
50
40
30
20
10
0
13C (ppm)

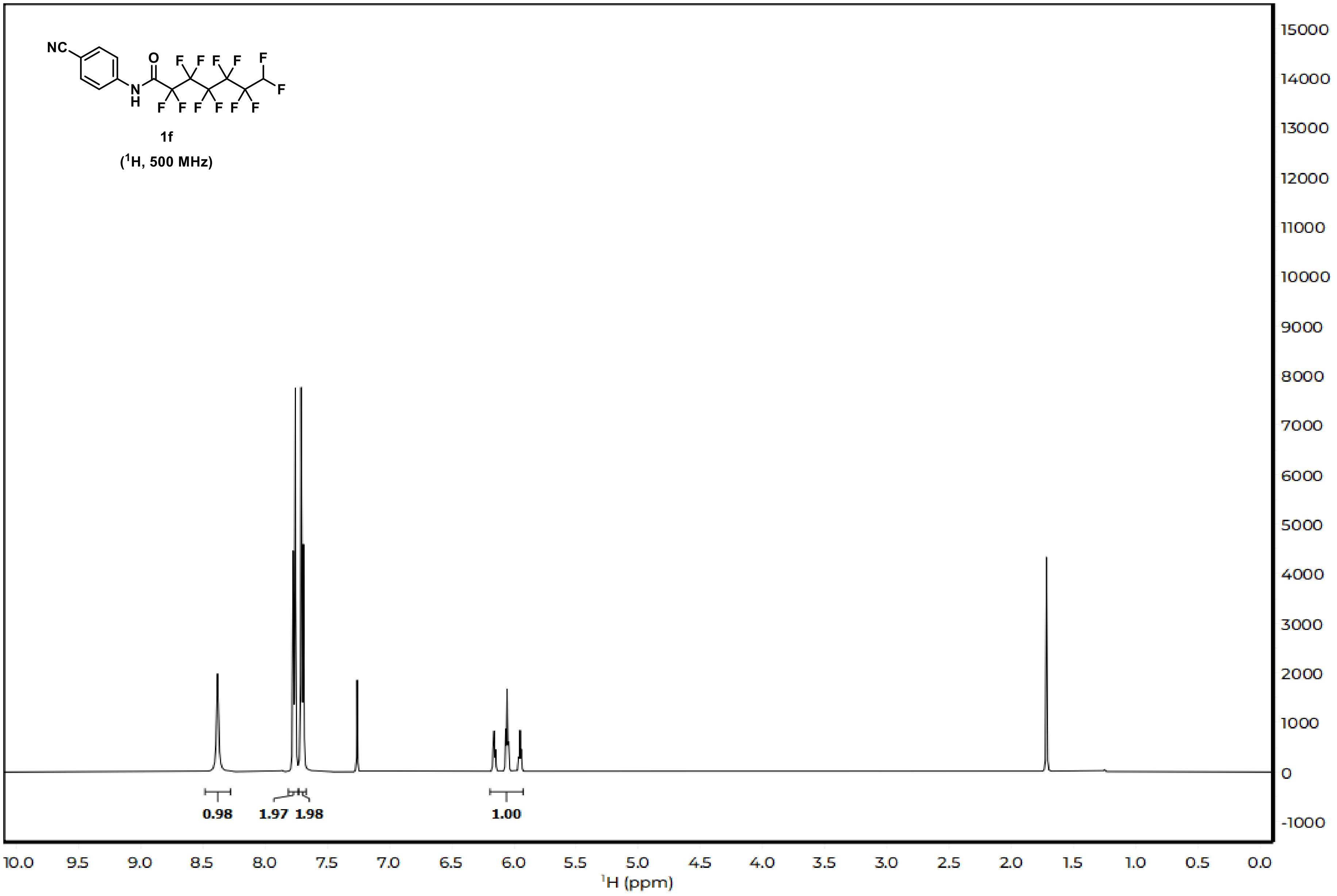
NC
O
N
H
F F F F F
F F F F F F
F
F
1f
(1H, 500 MHz)
15000
14000
13000
12000
11000
10000
9000
8000
7000
6000
5000
4000
3000
2000
1000
0
-1000
0.98
1.97 1.98
1.00
10.0 9.5 9.0 8.5 8.0 7.5 7.0 6.5 6.0 5.5 5.0 4.5 4.0 3.5 3.0 2.5 2.0 1.5 1.0 0.5 0.0
1H (ppm)

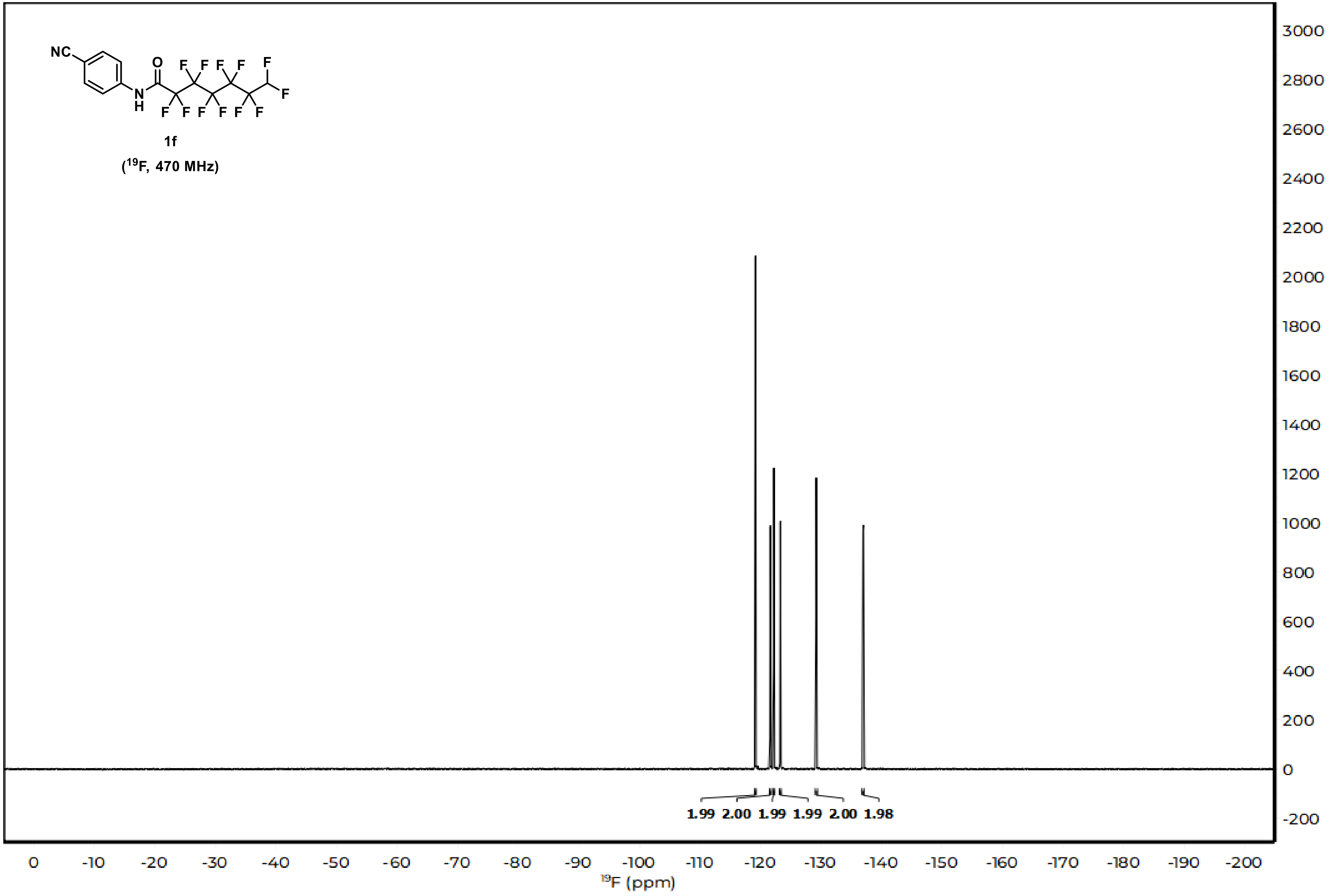

NC
O
N
H
F F F F F
F
F
F F F F F F
1f
(19F, 470 MHz)
3000
2800
2600
2400
2200
2000
1800
1600
1400
1200
1000
800
600
400
200
0
-200
1.99 2.00 1.99 1.99 2.00 1.98
0 -10 -20 -30 -40 -50 -60 -70 -80 -90 -100 -110 -120 -130 -140 -150 -160 -170 -180 -190 -200
19F (ppm)

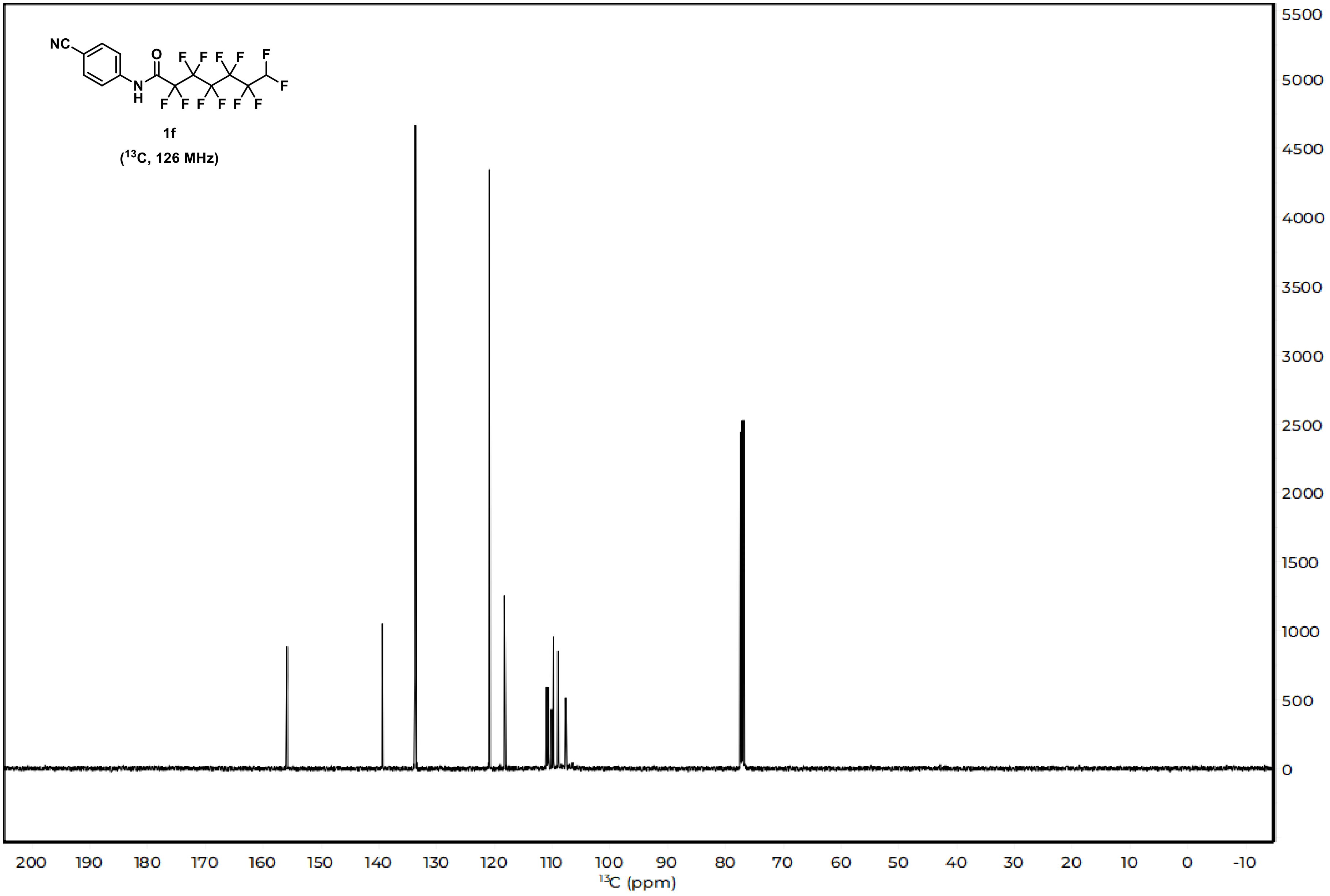
NC
O
N
H
F
1f
(13C, 126 MHz)
13C (ppm)

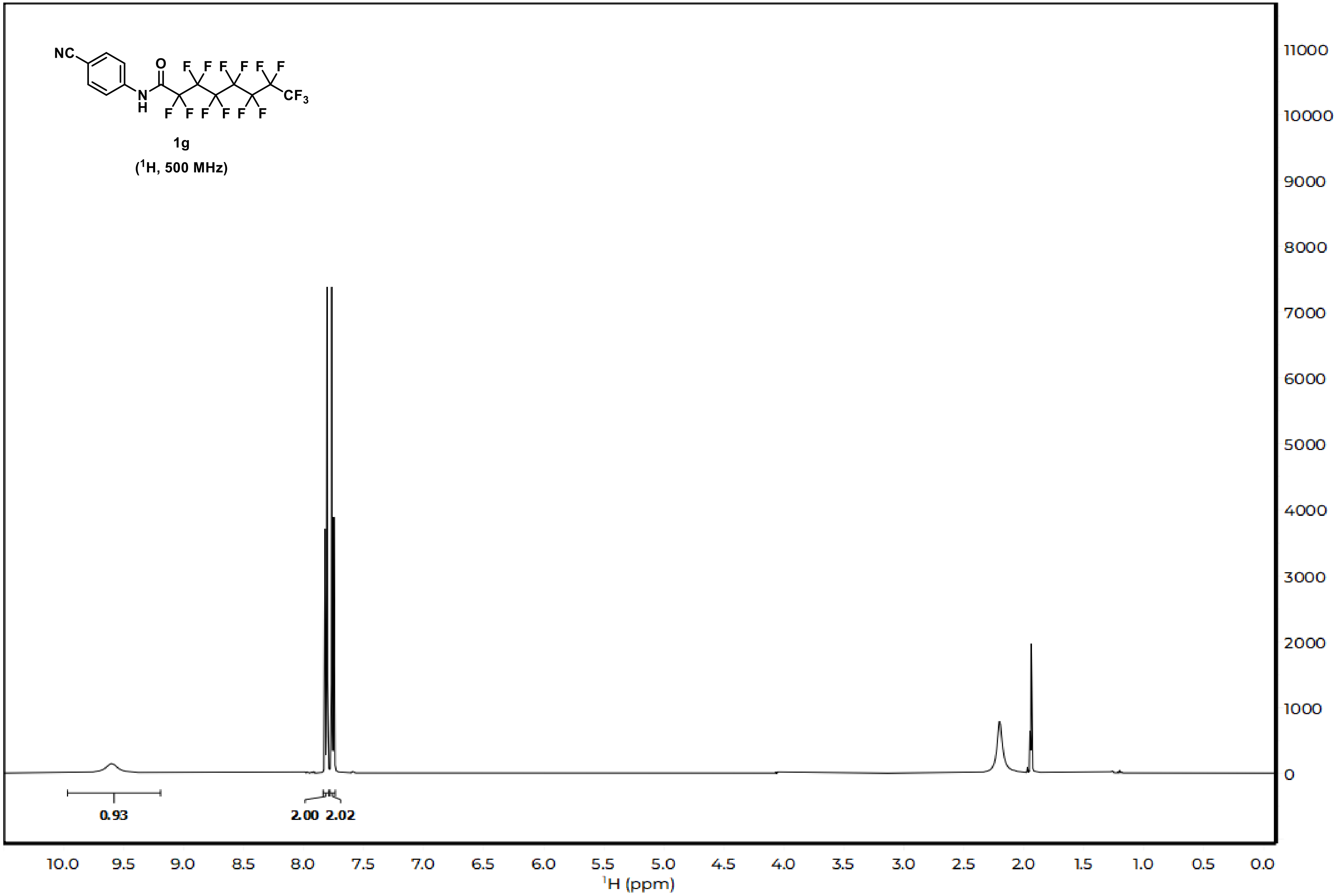
NC
O
N
H
F F F F F F
F F F F F F
CF3
1g
(1H, 500 MHz)
11000
10000
9000
8000
7000
6000
5000
4000
3000
2000
1000
0
0.93
2.00 2.02
10.0 9.5 9.0 8.5 8.0 7.5 7.0 6.5 6.0 5.5 5.0 4.5 4.0 3.5 3.0 2.5 2.0 1.5 1.0 0.5 0.0
1H (ppm)

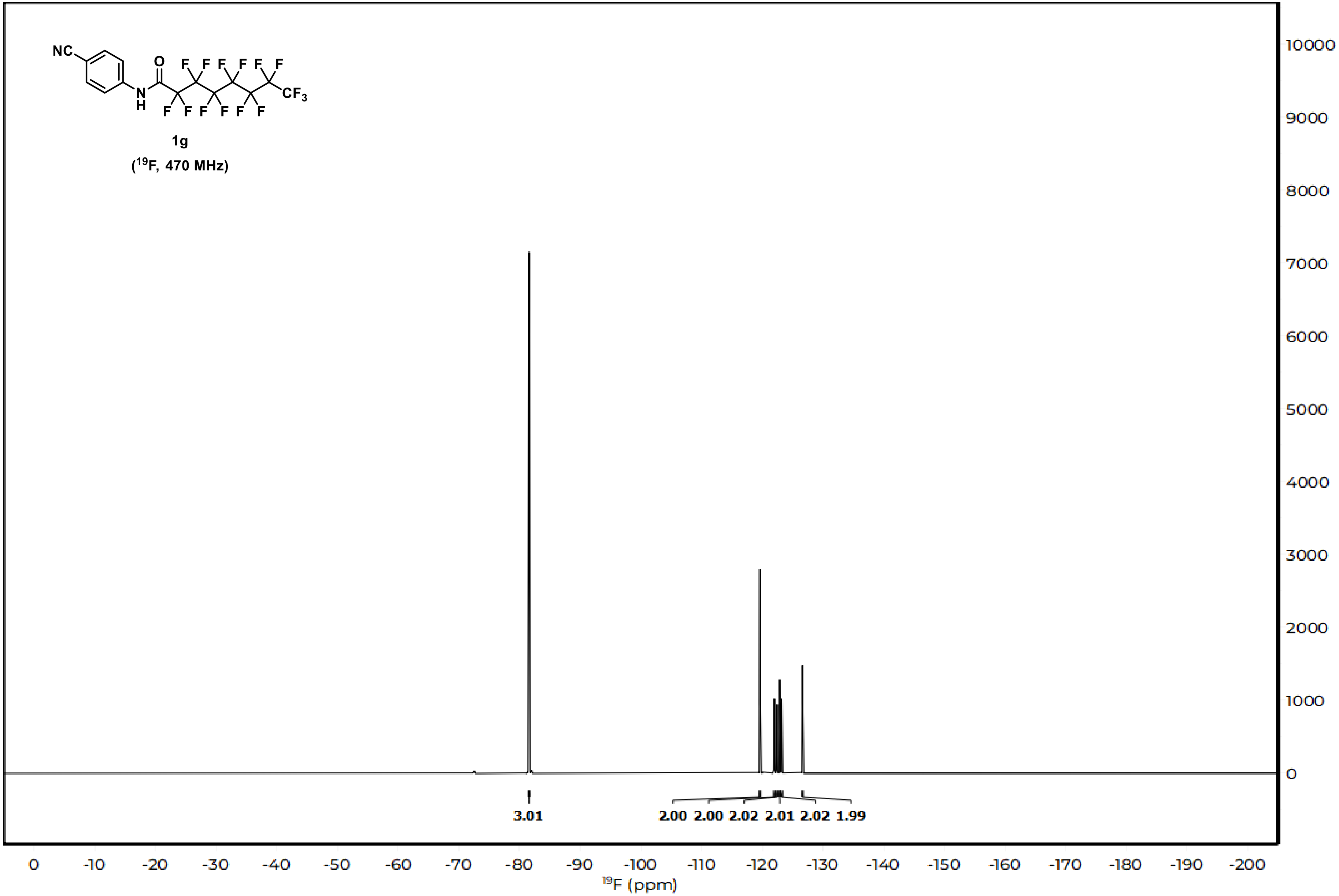

NC
O
F F F F F F
N
H
CF3
F F F F F F
1g
(19F, 470 MHz)
10000
9000
8000
7000
6000
5000
4000
3000
2000
1000
0
3.01
2.00 2.00 2.02 2.01 2.02 1.99
0 -10 -20 -30 -40 -50 -60 -70 -80 -90 -100 -110 -120 -130 -140 -150 -160 -170 -180 -190 -200
19F (ppm)

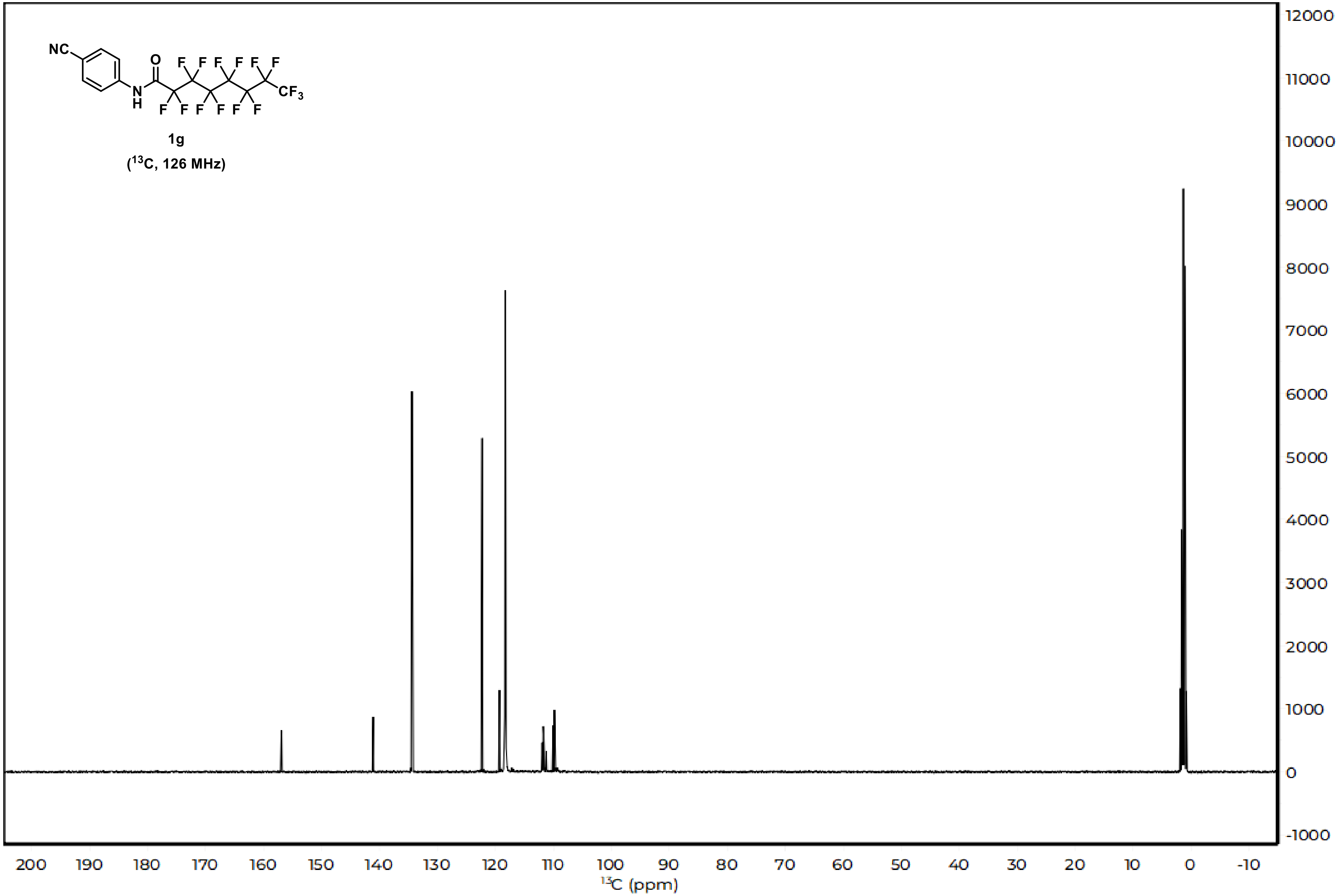

NC
O
N
H
F F F F F F
F F F F F F
CF3
1g
(13C, 126 MHz)
12000
11000
10000
9000
8000
7000
6000
5000
4000
3000
2000
1000
0
-1000
200 190 180 170 160 150 140 130 120 110 100 90 80 70 60 50 40 30 20 10 0 -10
13C (ppm)

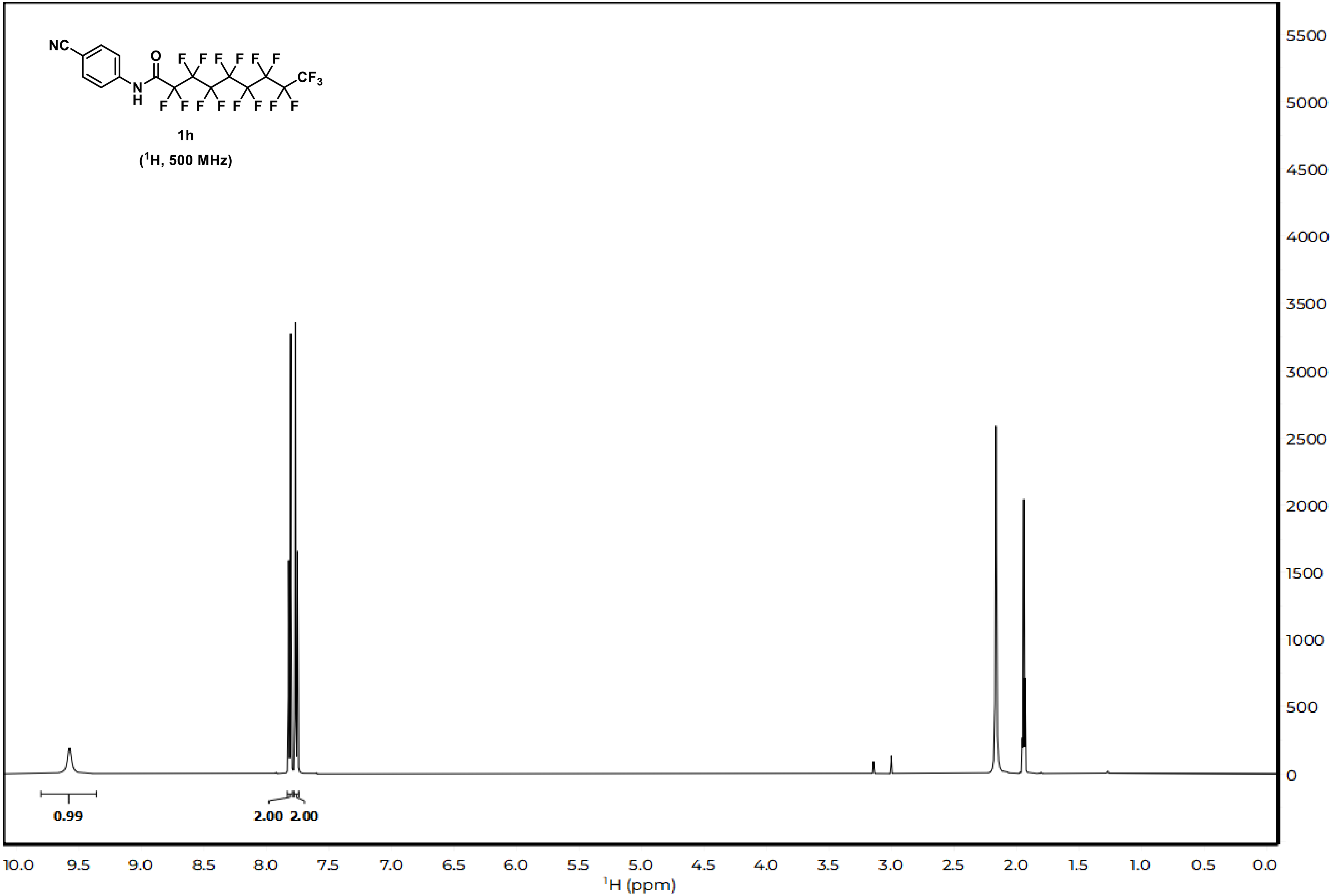
NC
O
N
H
F F F F F F
CF3
F F F F F F
1h
(1H, 500 MHz)
0.99
2.00 2.00
10.0 9.5 9.0 8.5 8.0 7.5 7.0 6.5 6.0 5.5 5.0 4.5 4.0 3.5 3.0 2.5 2.0 1.5 1.0 0.5 0.0
1H (ppm)
5500 5000 4500 4000 3500 3000 2500 2000 1500 1000 500 0

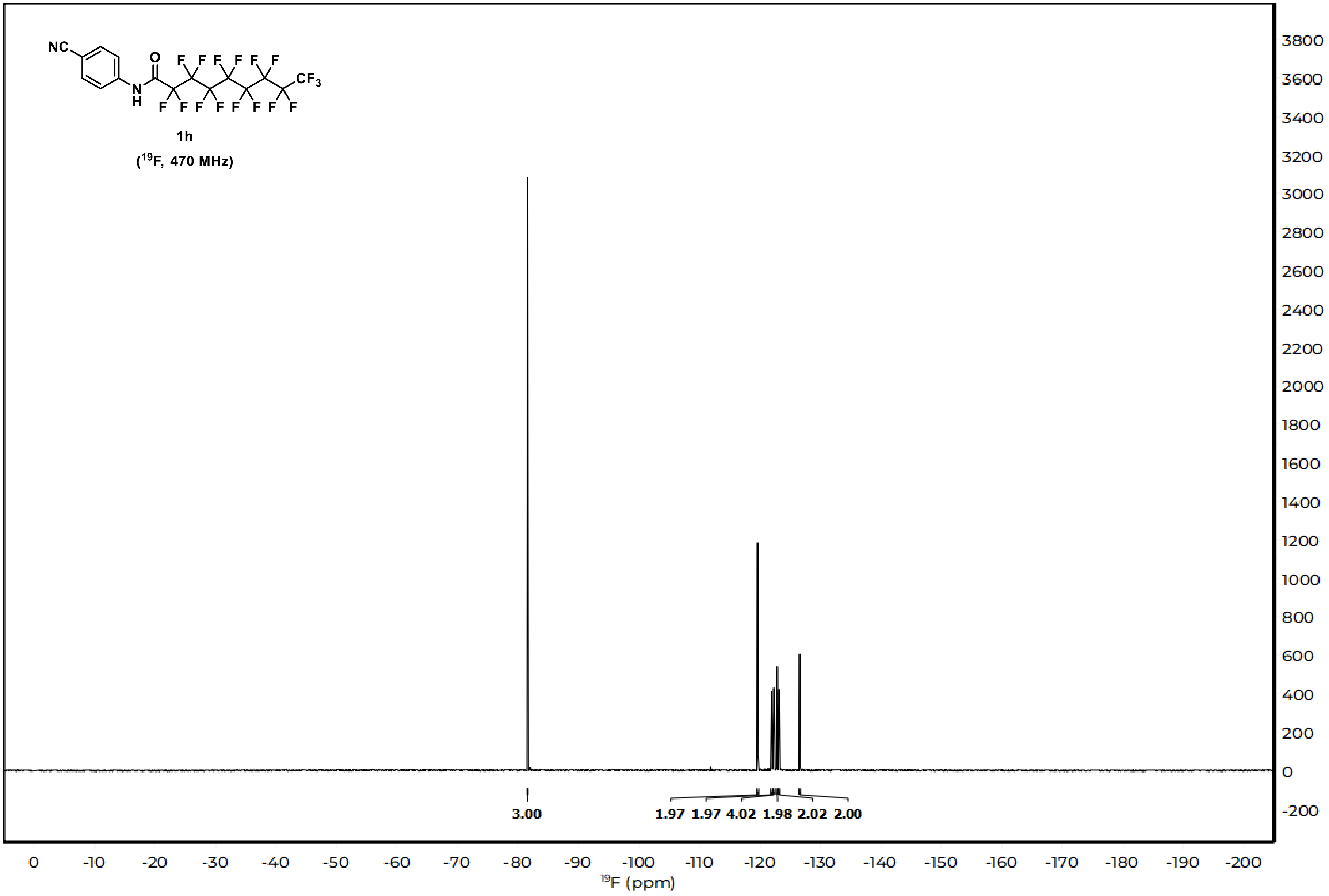
NC
O
N
H
CF3
1h
(19F, 470 MHz)
3.00
1.97 1.97 4.02 1.98 2.02 2.00
3800
3600
3400
3200
3000
2800
2600
2400
2200
2000
1800
1600
1400
1200
1000
800
600
400
200
0
-200
0 -10 -20 -30 -40 -50 -60 -70 -80 -90 -100 -110 -120 -130 -140 -150 -160 -170 -180 -190 -200
19F (ppm)

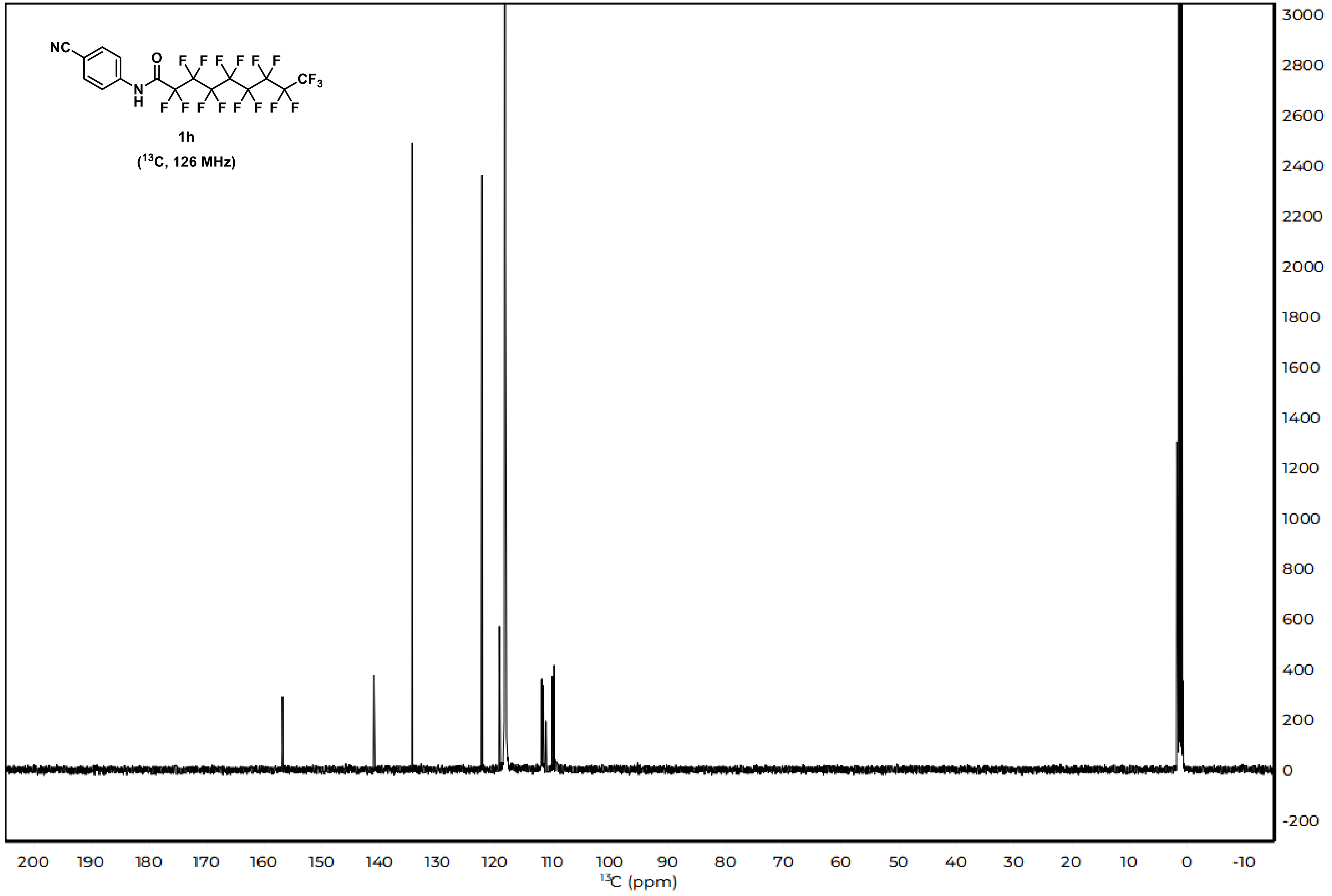

NC
O
N
H
F F F F F F
CF3
F F F F F F F F
1h
(13C, 126 MHz)
3000
2800
2600
2400
2200
2000
1800
1600
1400
1200
1000
800
600
400
200
0
-200
200 190 180 170 160 150 140 130 120 110 100 90 80 70 60 50 40 30 20 10 0 -10
13C (ppm)

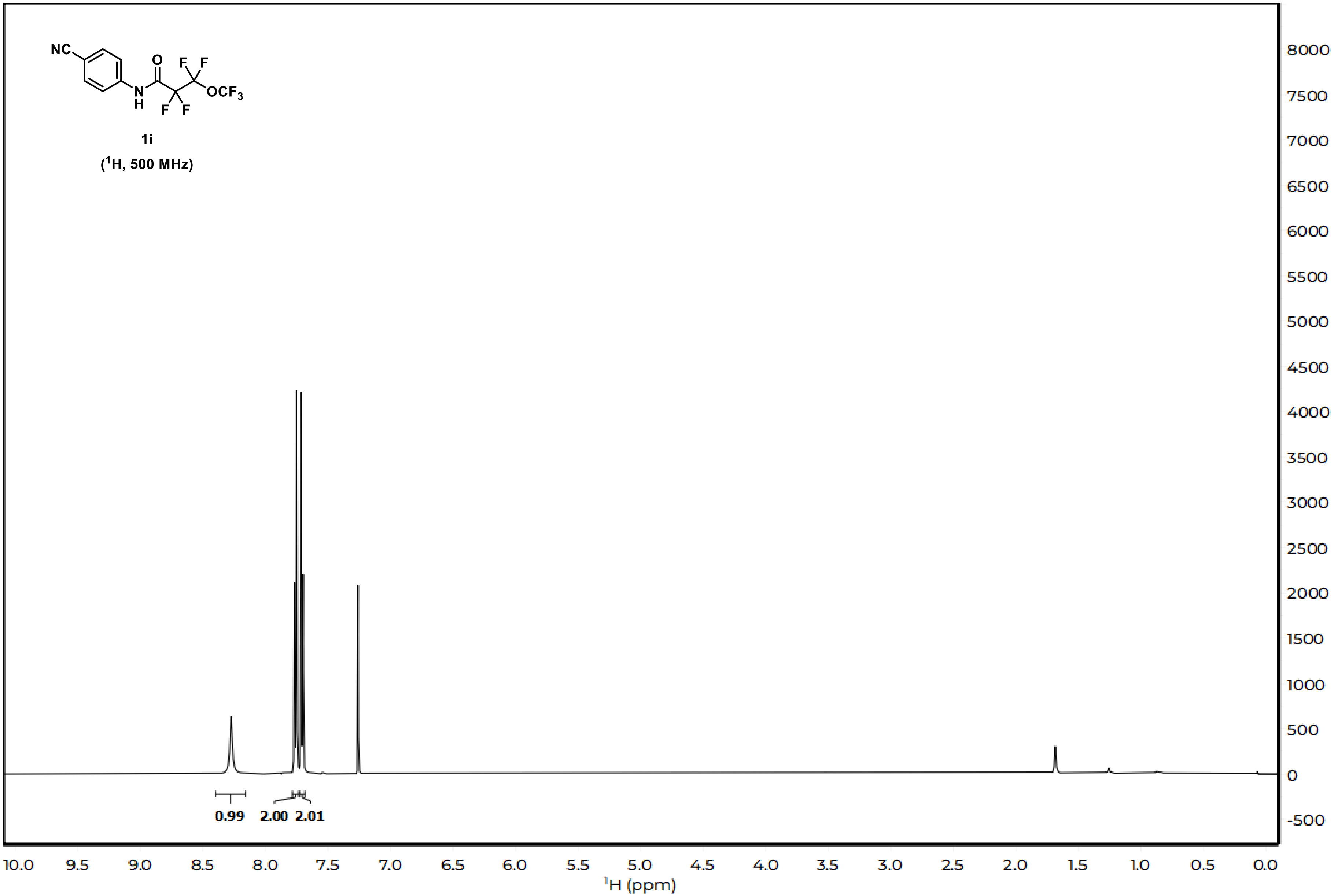
NC
O
F F
N
H
OCF3
F F
1i
(1H, 500 MHz)
8000
7500
7000
6500
6000
5500
5000
4500
4000
3500
3000
2500
2000
1500
1000
500
0
-500
0.99
2.00 2.01
10.0 9.5 9.0 8.5 8.0 7.5 7.0 6.5 6.0 5.5 5.0 4.5 4.0 3.5 3.0 2.5 2.0 1.5 1.0 0.5 0.0
1H (ppm)

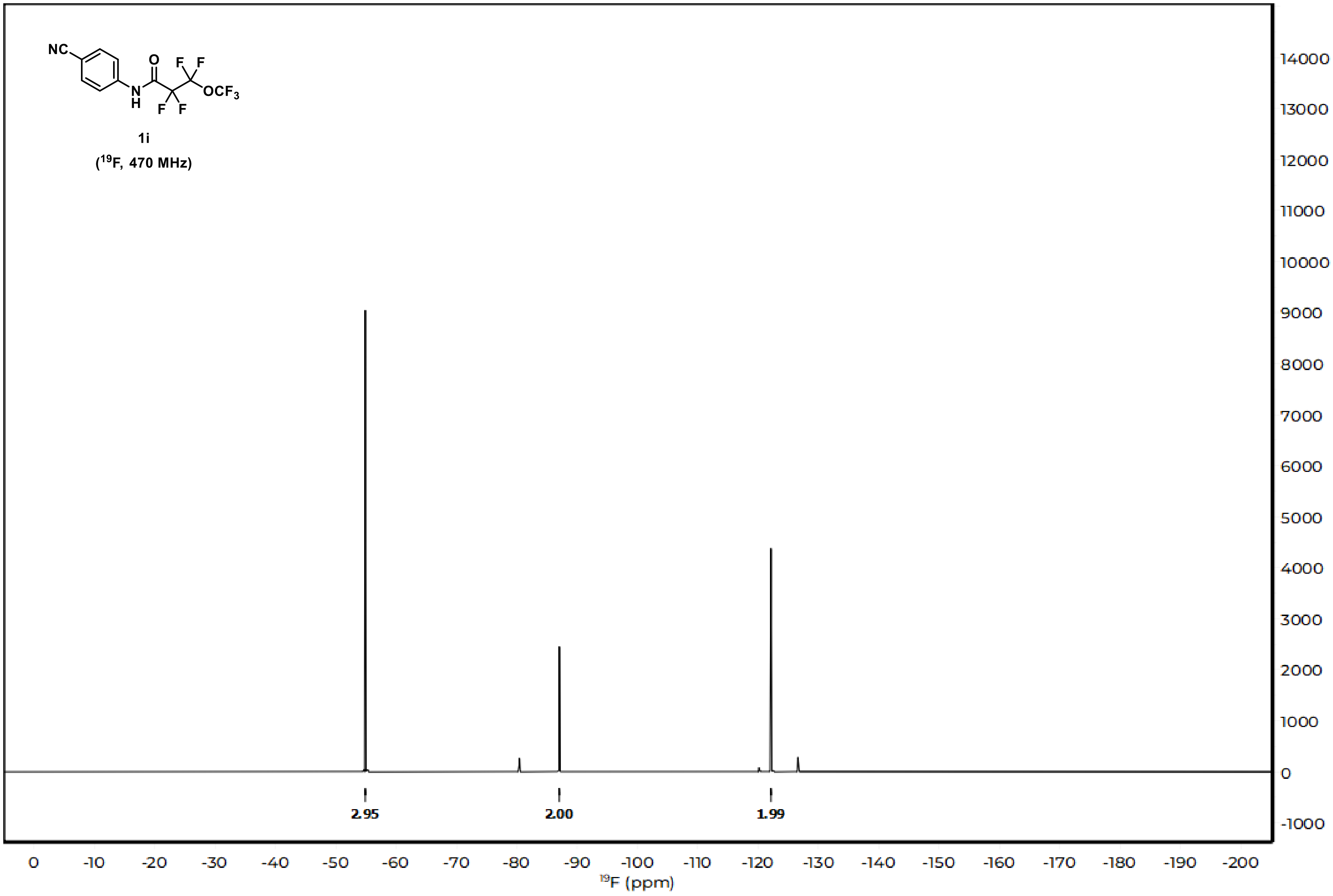

NC
O
F F
N
H
OCF3
F F
1i
(19F, 470 MHz)
14000
13000
12000
11000
10000
9000
8000
7000
6000
5000
4000
3000
2000
1000
0
-1000
2.95
2.00
1.99
0
-10
-20
-30
-40
-50
-60
-70
-80
-90
-100
-110
-120
-130
-140
-150
-160
-170
-180
-190
-200
19F (ppm)

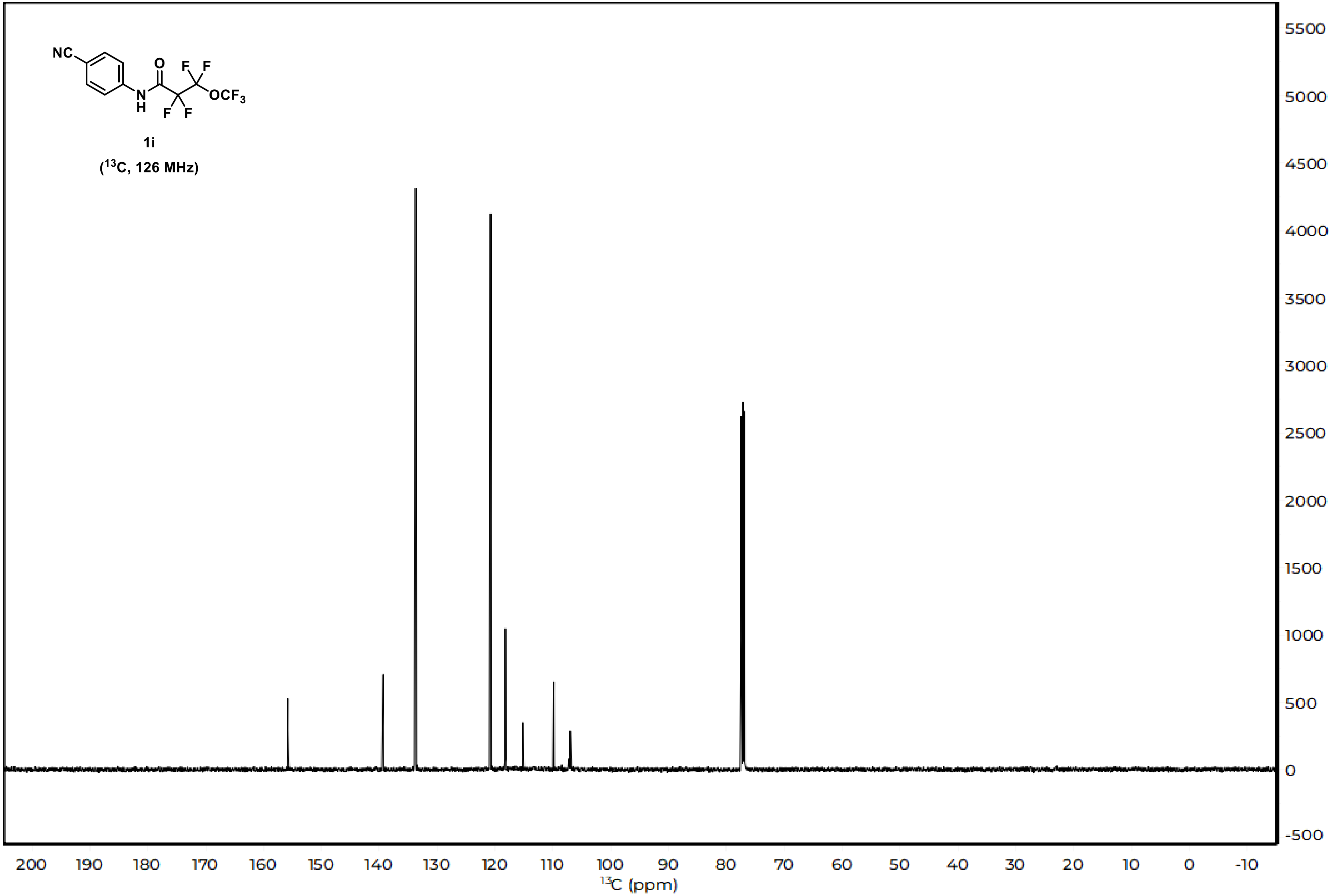
NC
O
F F
OCF3
N
H
F F
1i
(13C, 126 MHz)
5500
5000
4500
4000
3500
3000
2500
2000
1500
1000
500
0
-500
200 190 180 170 160 150 140 130 120 110 100 90 80 70 60 50 40 30 20 10 0 -10
13C (ppm)

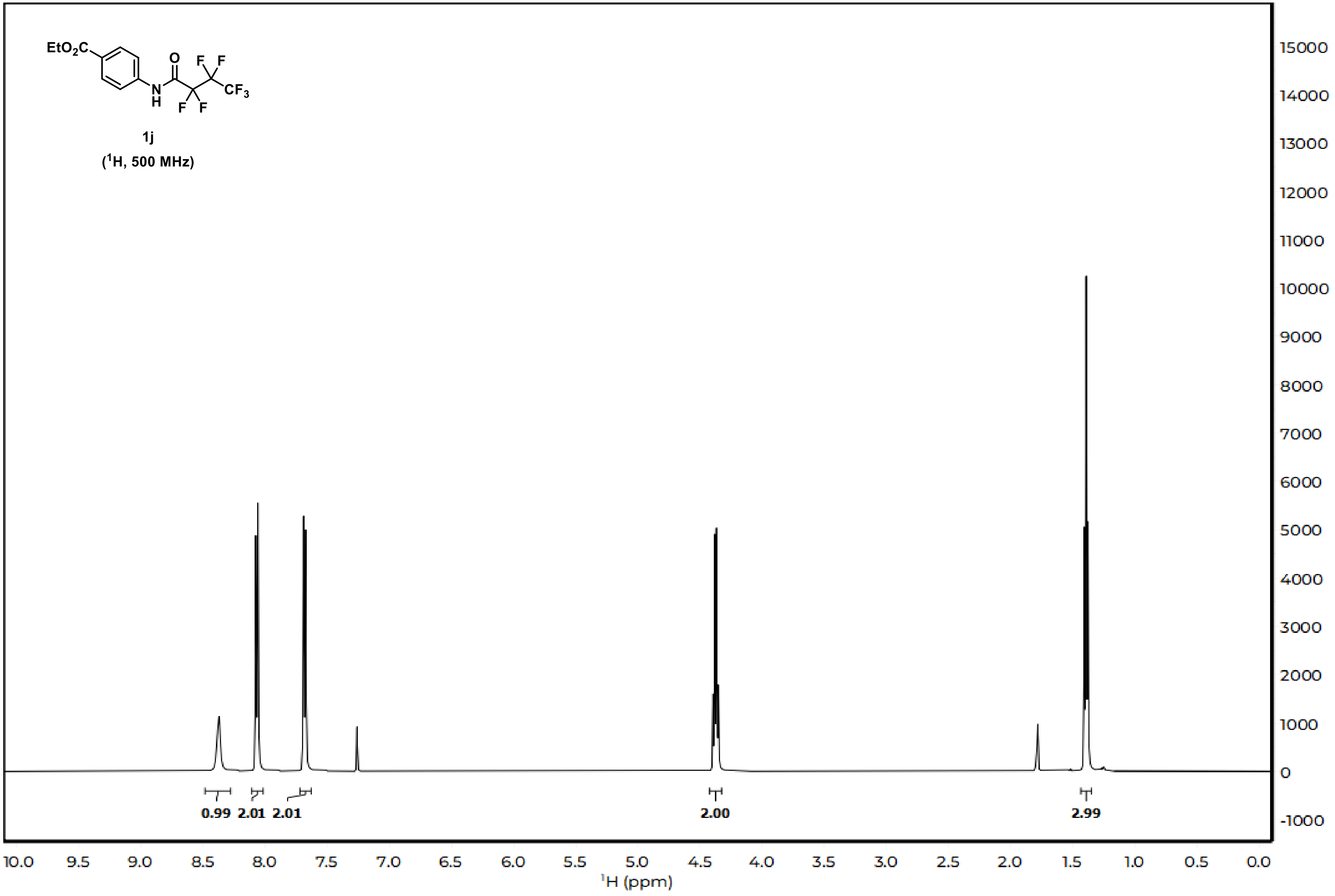

EtO2C
O
F F
N
H
CF3
F F
1j
(1H, 500 MHz)
15000
14000
13000
12000
11000
10000
9000
8000
7000
6000
5000
4000
3000
2000
1000
0
-1000
0.99
2.01
2.01
2.00
2.99
10.0
9.5
9.0
8.5
8.0
7.5
7.0
6.5
6.0
5.5
5.0
4.5
4.0
3.5
3.0
2.5
2.0
1.5
1.0
0.5
0.0
1H (ppm)

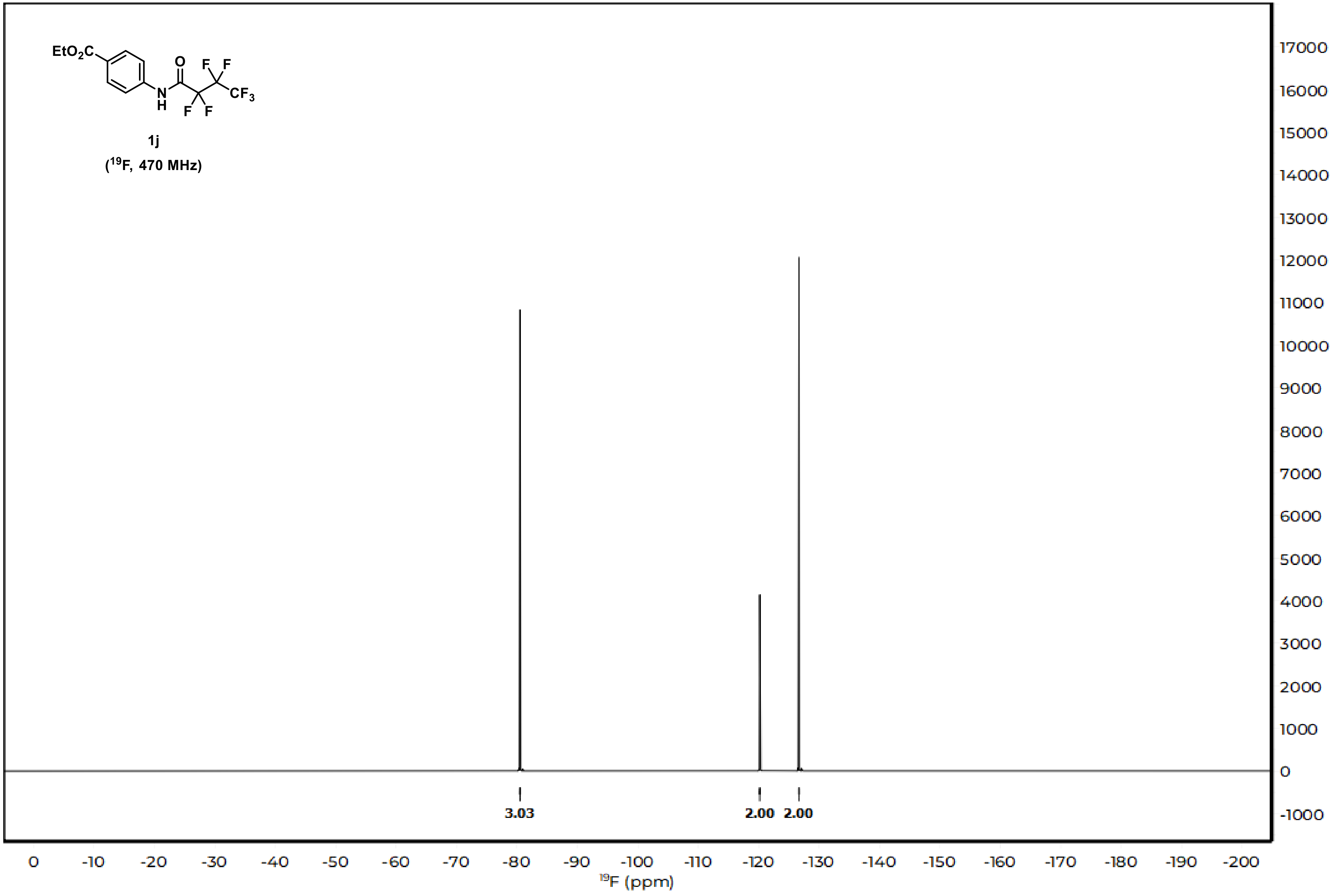

EtO2C
O
F F
N
H
CF3
F F
1j
(19F, 470 MHz)
3.03
2.00 2.00
19F (ppm)

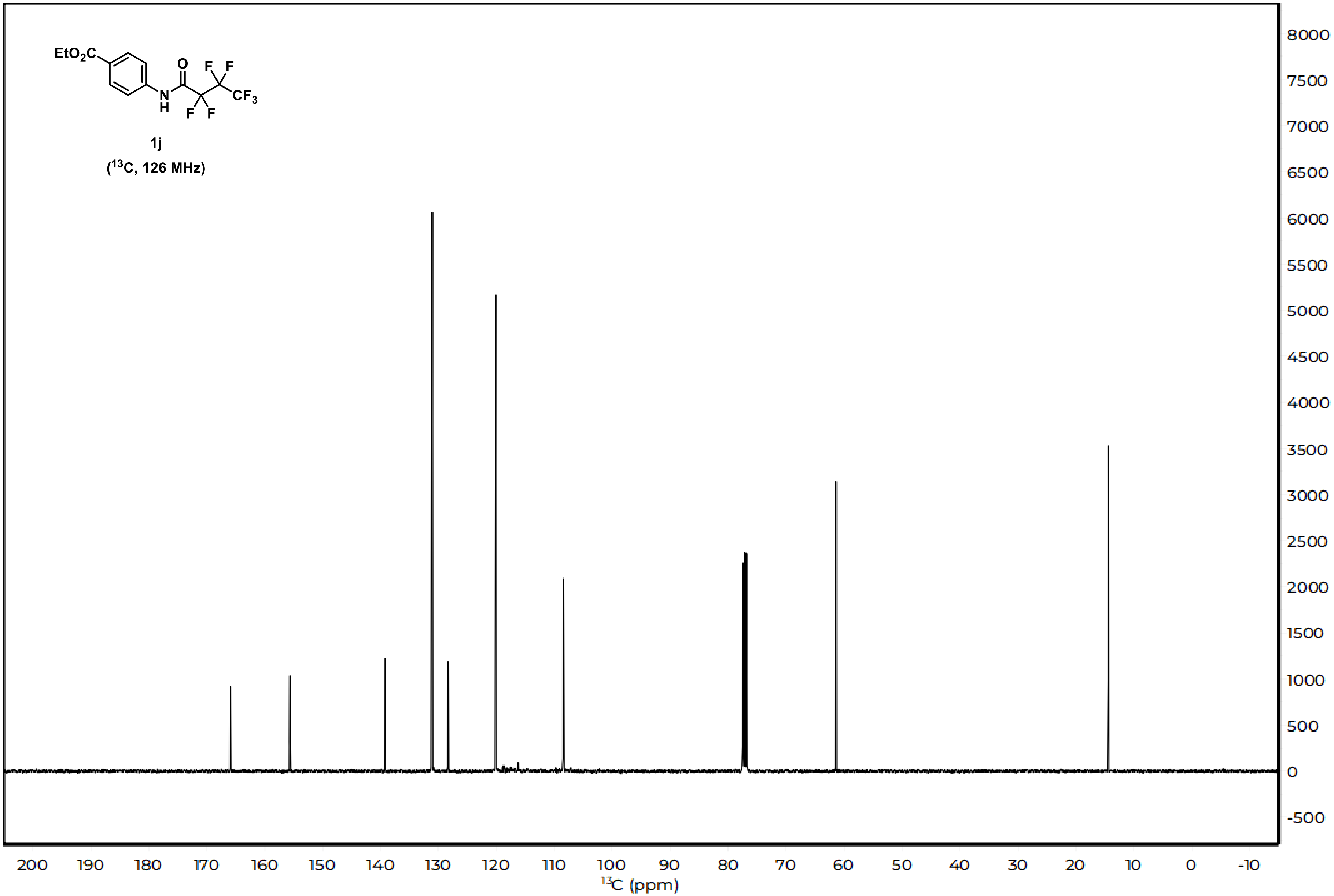

EtO2C
O
F
F
N
H
CF3
F
F
1j
(13C, 126 MHz)
8000
7500
7000
6500
6000
5500
5000
4500
4000
3500
3000
2500
2000
1500
1000
500
0
-500
200
190
180
170
160
150
140
130
120
110
100
90
80
70
60
50
40
30
20
10
0
-10
13C (ppm)

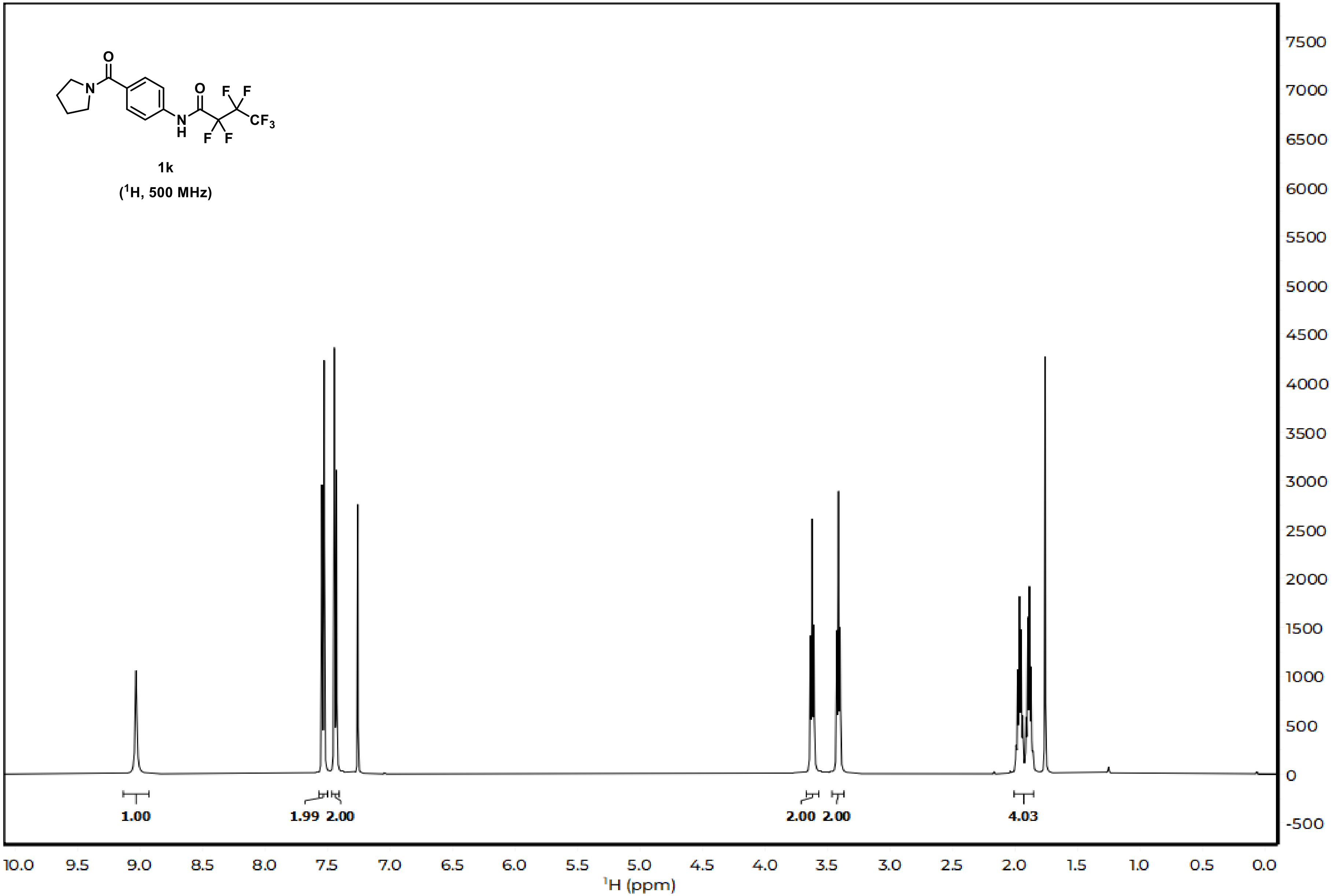

1k
(1H, 500 MHz)
1.00
1.99 2.00
2.00 2.00
4.03
10.0 9.5 9.0 8.5 8.0 7.5 7.0 6.5 6.0 5.5 5.0 4.5 4.0 3.5 3.0 2.5 2.0 1.5 1.0 0.5 0.0
1H (ppm)
7500 7000 6500 6000 5500 5000 4500 4000 3500 3000 2500 2000 1500 1000 500 0 -500

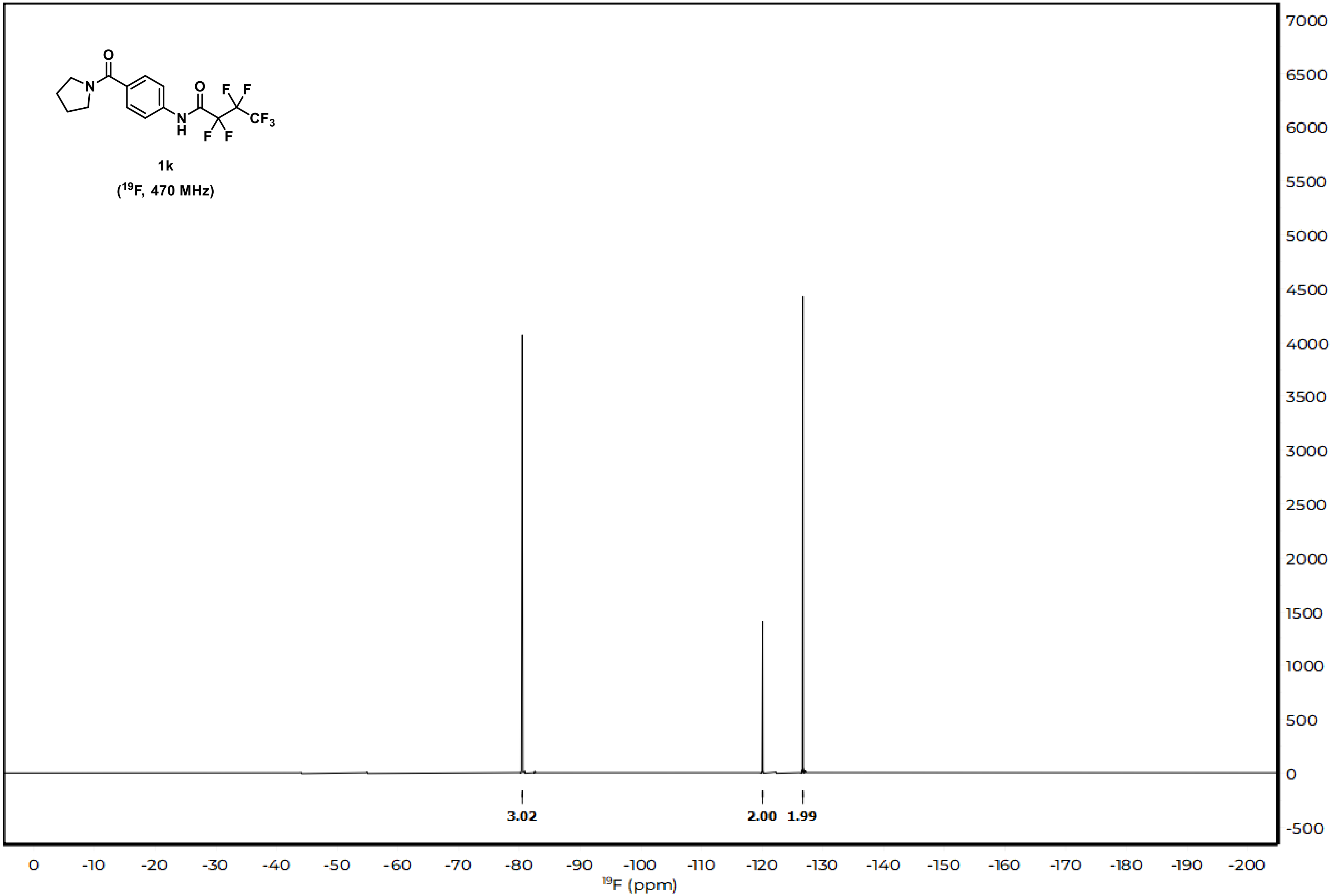
1k
(19F, 470 MHz)
3.02
2.00
1.99
7000
6500
6000
5500
5000
4500
4000
3500
3000
2500
2000
1500
1000
500
0
-500
0
-10
-20
-30
-40
-50
-60
-70
-80
-90
-100
-110
-120
-130
-140
-150
-160
-170
-180
-190
-200
19F (ppm)

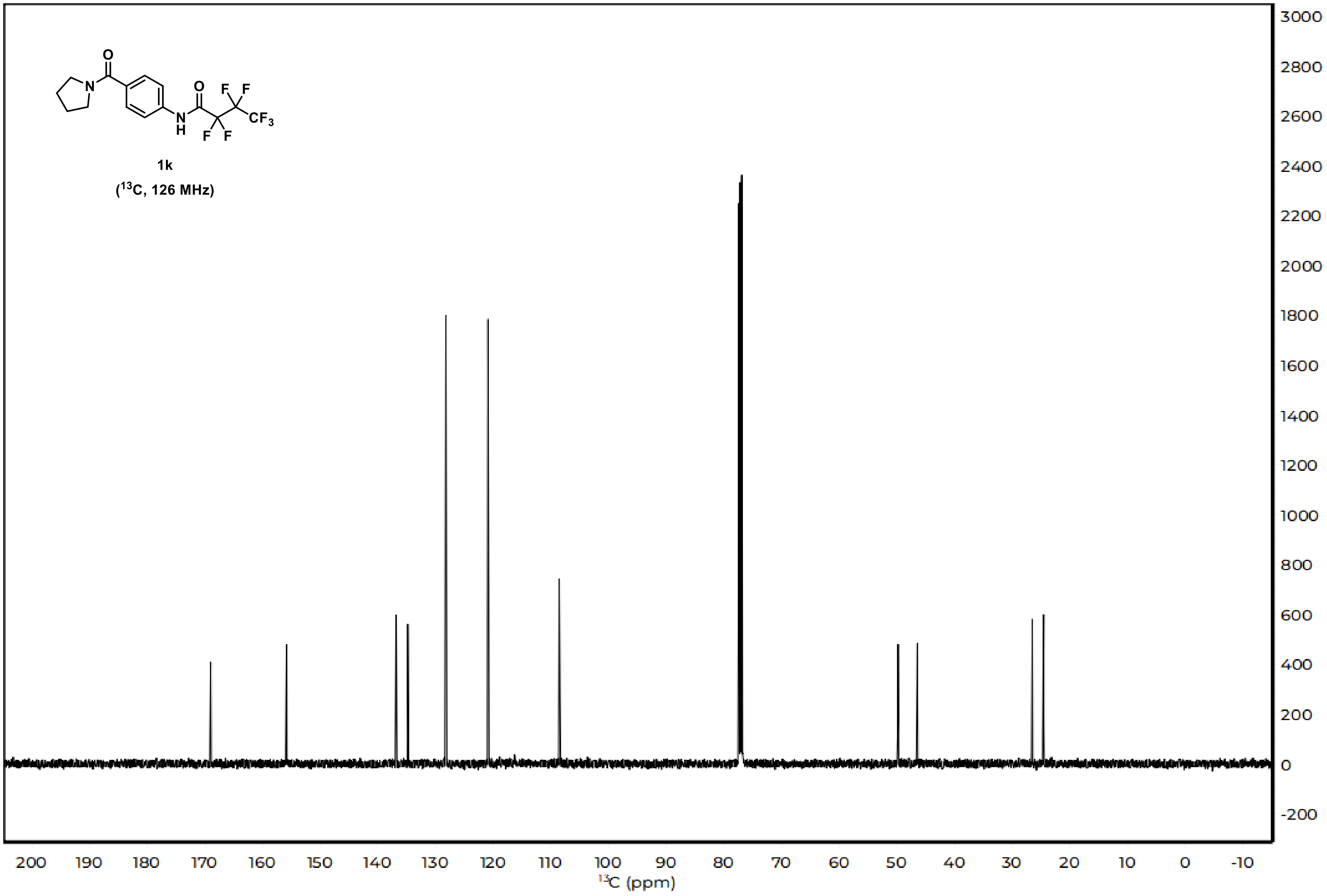

O
N
O
F F
N
H
CF3
F F
1k
(13C, 126 MHz)
3000
2800
2600
2400
2200
2000
1800
1600
1400
1200
1000
800
600
400
200
0
-200
200 190 180 170 160 150 140 130 120 110 100 90 80 70 60 50 40 30 20 10 0 -10
13C (ppm)

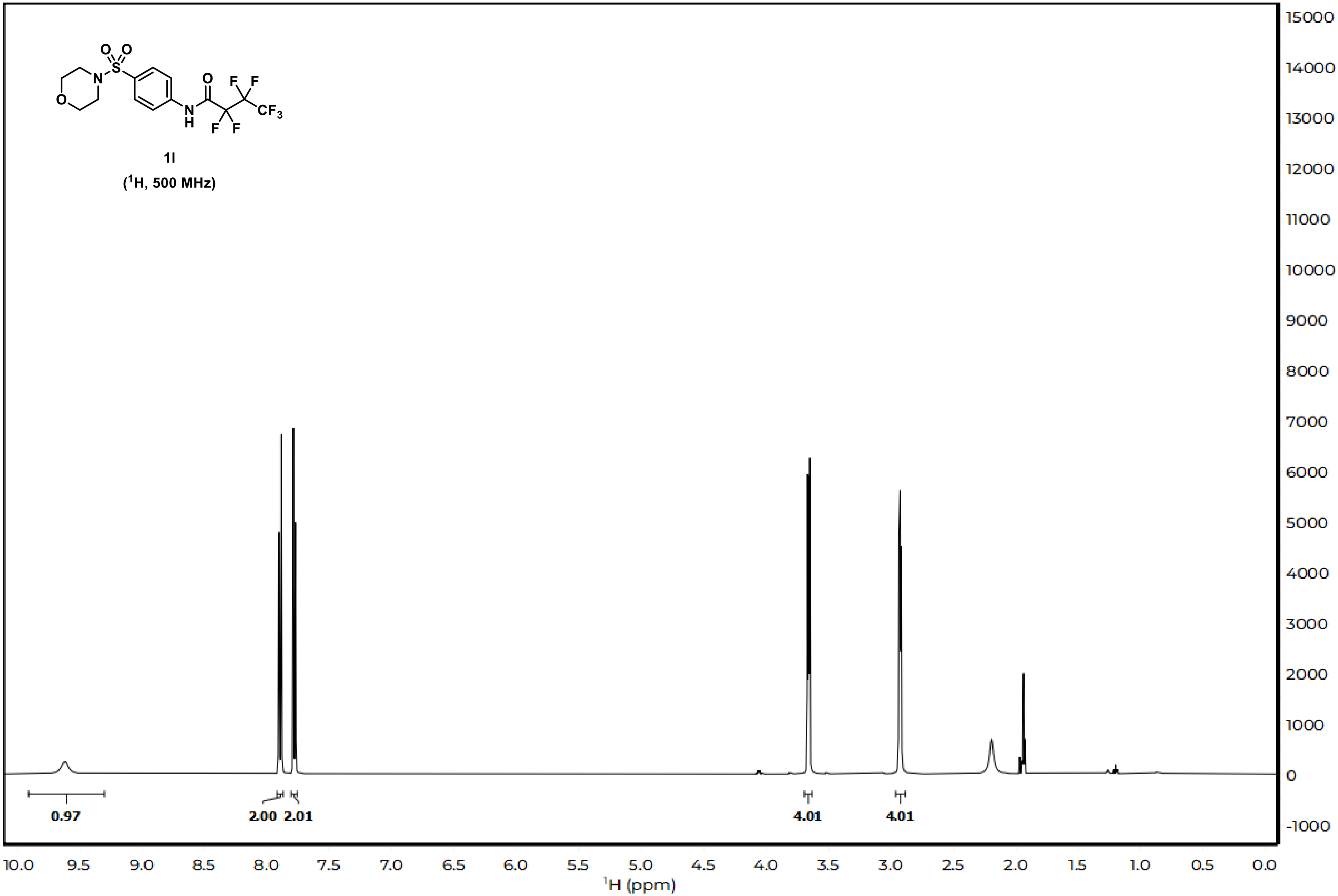
1l
(1H, 500 MHz)
0.97
2.00 2.01
4.01
4.01
1H (ppm)

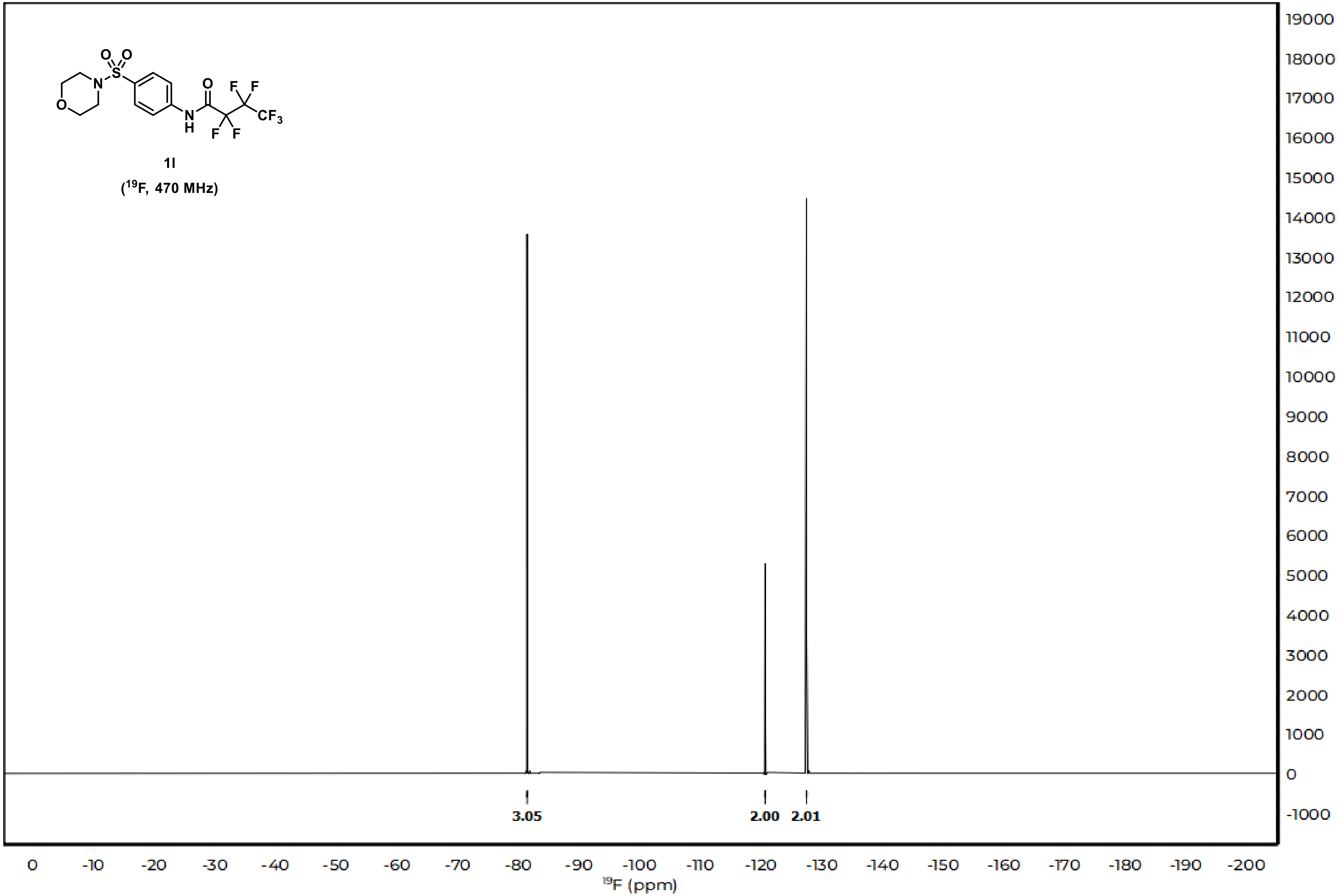
1l
(^{19}F, 470 MHz)
3.05
2.00
2.01
19000
18000
17000
16000
15000
14000
13000
12000
11000
10000
9000
8000
7000
6000
5000
4000
3000
2000
1000
0
-1000
0
-10
-20
-30
-40
-50
-60
-70
-80
-90
-100
-110
-120
-130
-140
-150
-160
-170
-180
-190
-200
^{19}F (ppm)

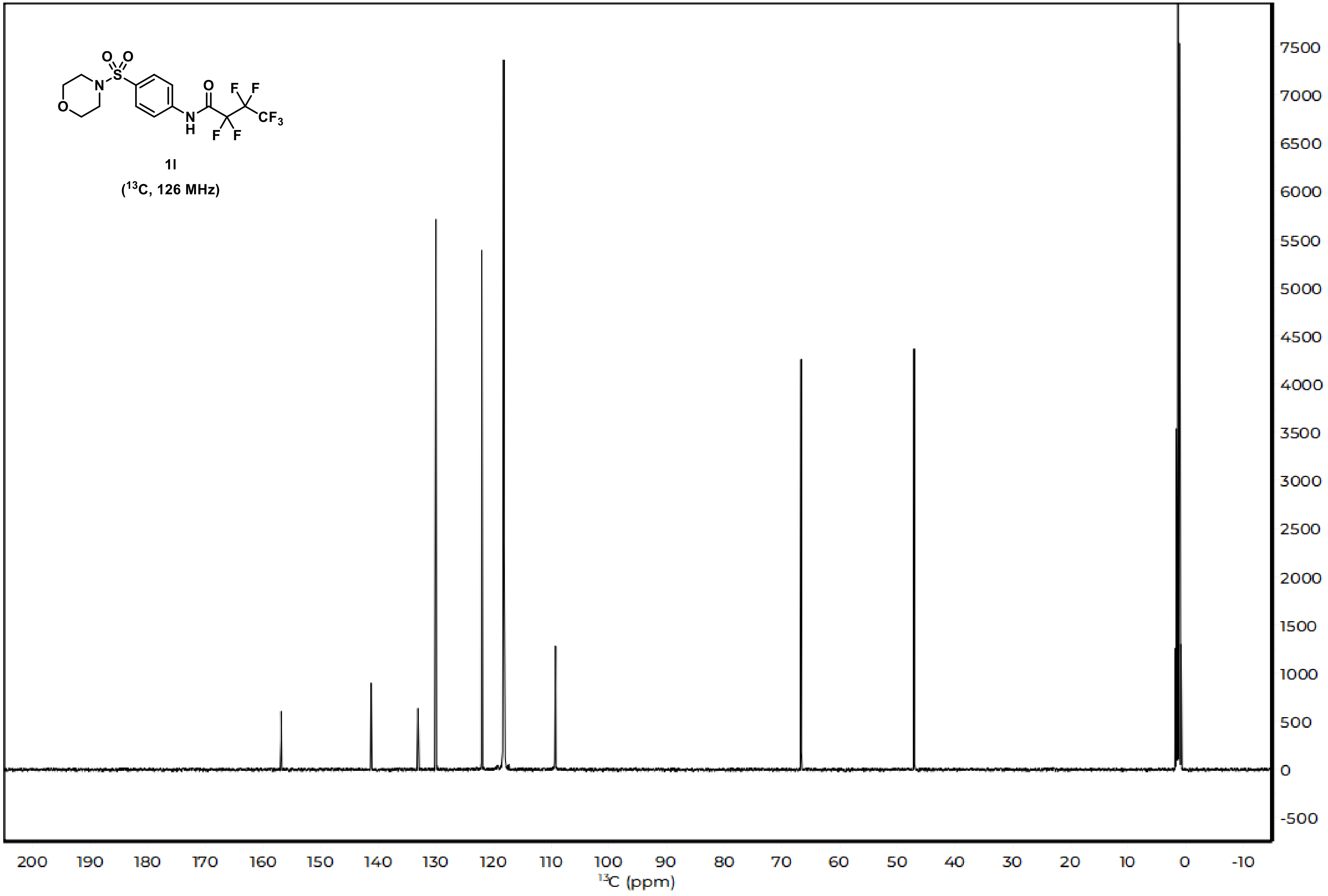

1l
(13C, 126 MHz)
13C (ppm)

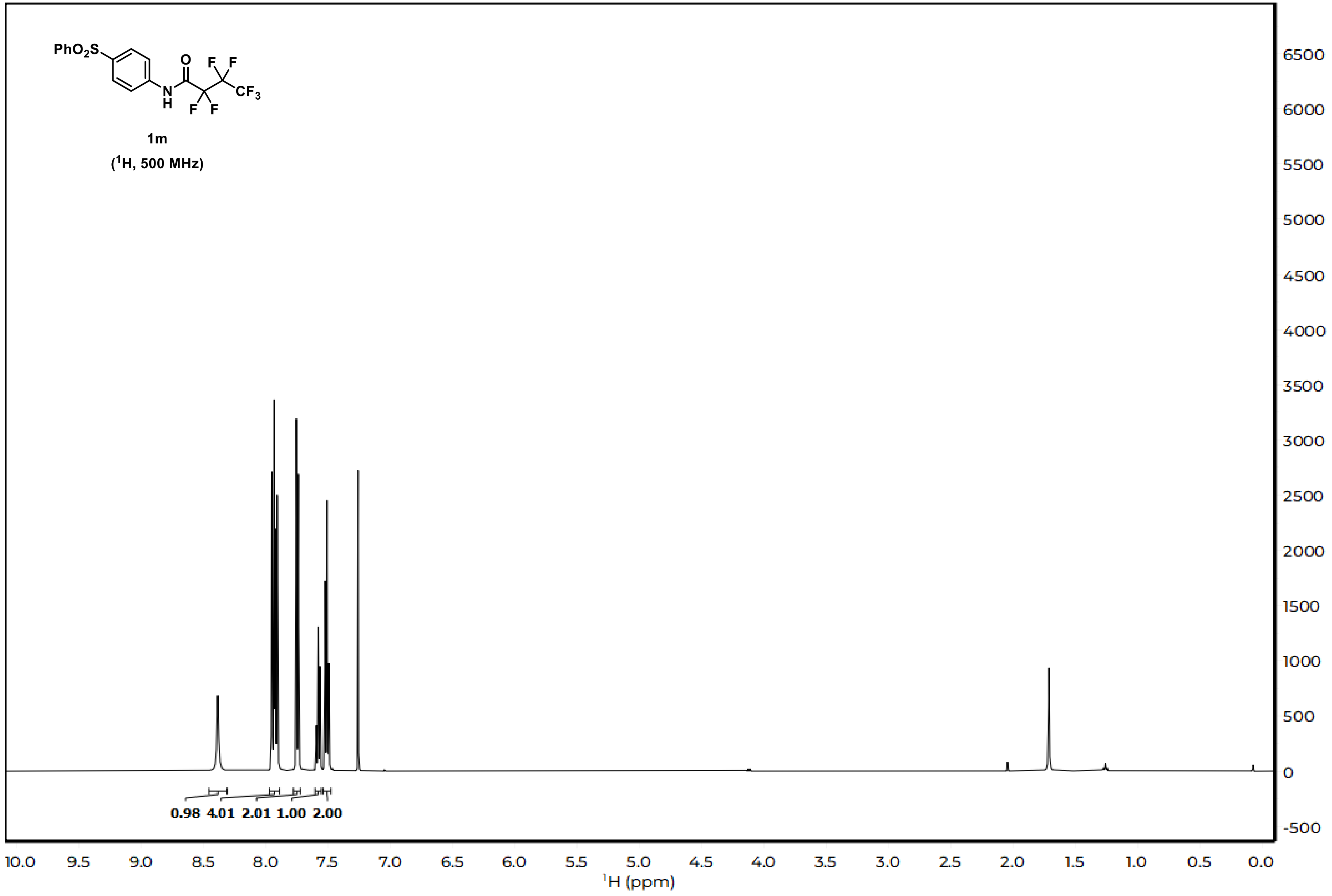

PhO2S
O
F F
N
H
CF3
F F
1m
(1H, 500 MHz)
0.98 4.01 2.01 1.00 2.00
1H (ppm)

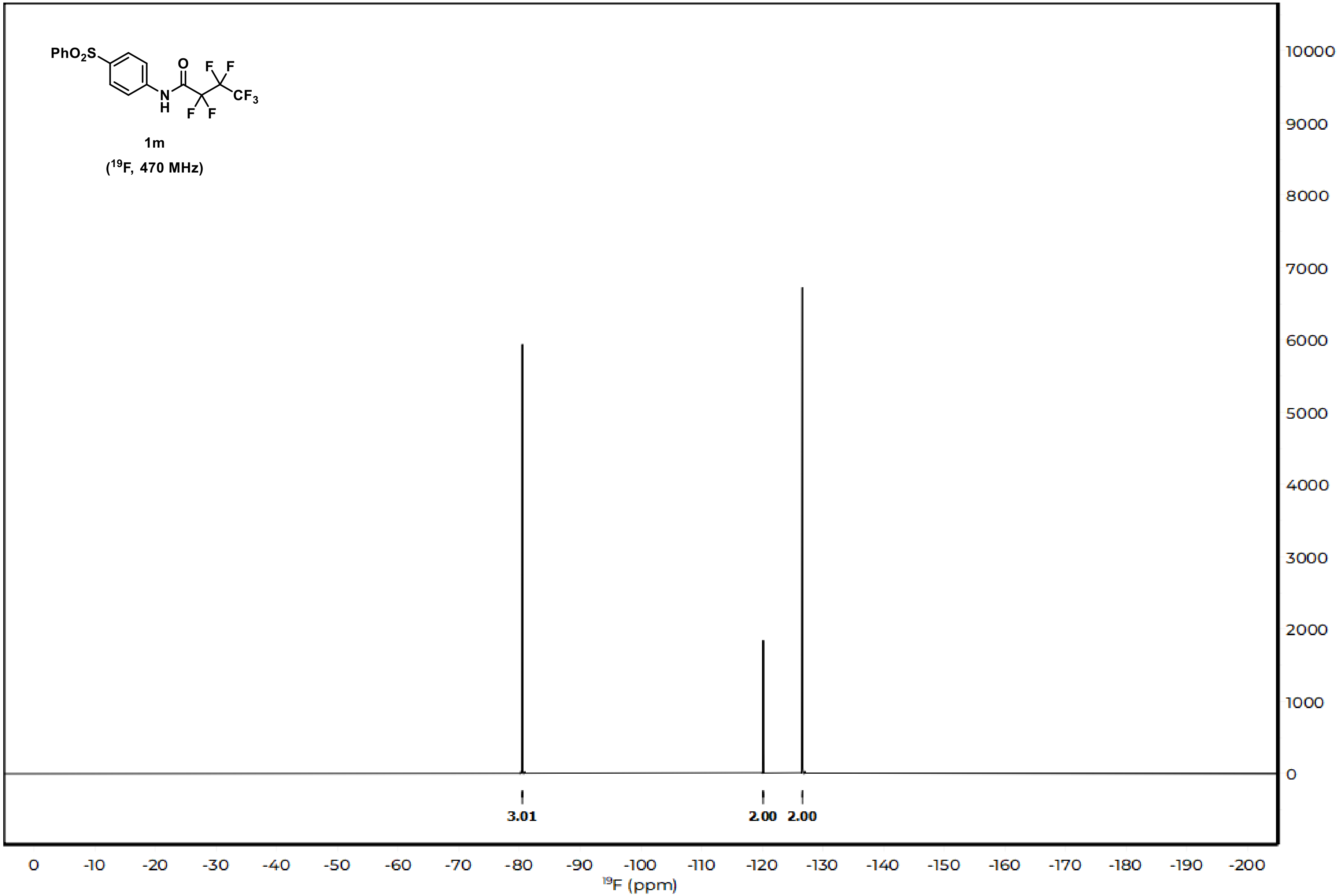
PhO2S
O
F F
N
H
CF3
F F
1m
(19F, 470 MHz)
10000
9000
8000
7000
6000
5000
4000
3000
2000
1000
0
3.01
2.00
2.00
0
-10
-20
-30
-40
-50
-60
-70
-80
-90
-100
-110
-120
-130
-140
-150
-160
-170
-180
-190
-200
19F (ppm)

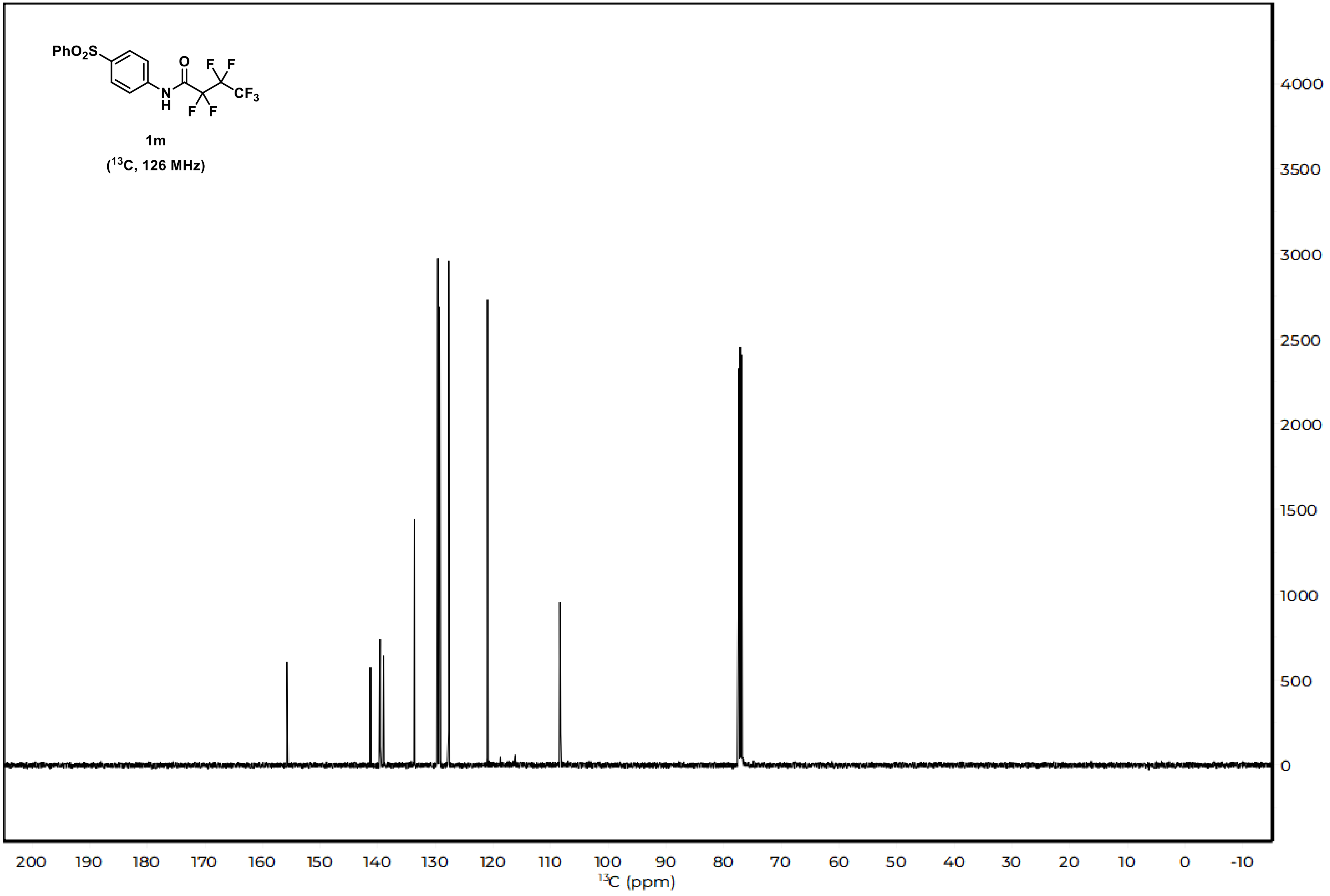
PhO2S
O
F F
N
H
CF3
F F
1m
(13C, 126 MHz)
4000
3500
3000
2500
2000
1500
1000
500
0
200 190 180 170 160 150 140 130 120 110 100 90 80 70 60 50 40 30 20 10 0 -10
13C (ppm)

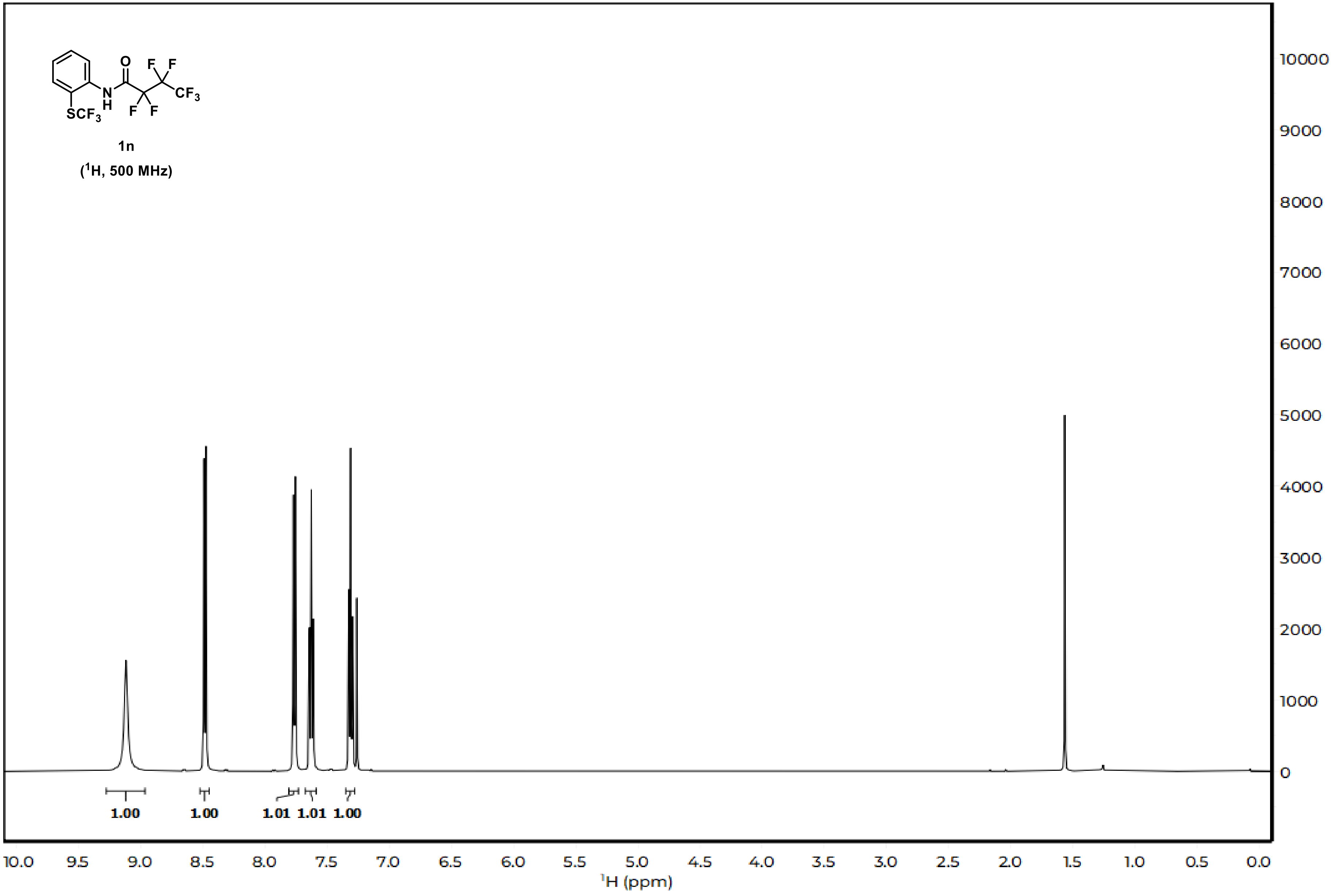

SCF3
1n
(1H, 500 MHz)
1.00
1.00
1.01
1.01
1.00
10000
9000
8000
7000
6000
5000
4000
3000
2000
1000
0
10.0 9.5 9.0 8.5 8.0 7.5 7.0 6.5 6.0 5.5 5.0 4.5 4.0 3.5 3.0 2.5 2.0 1.5 1.0 0.5 0.0
1H (ppm)

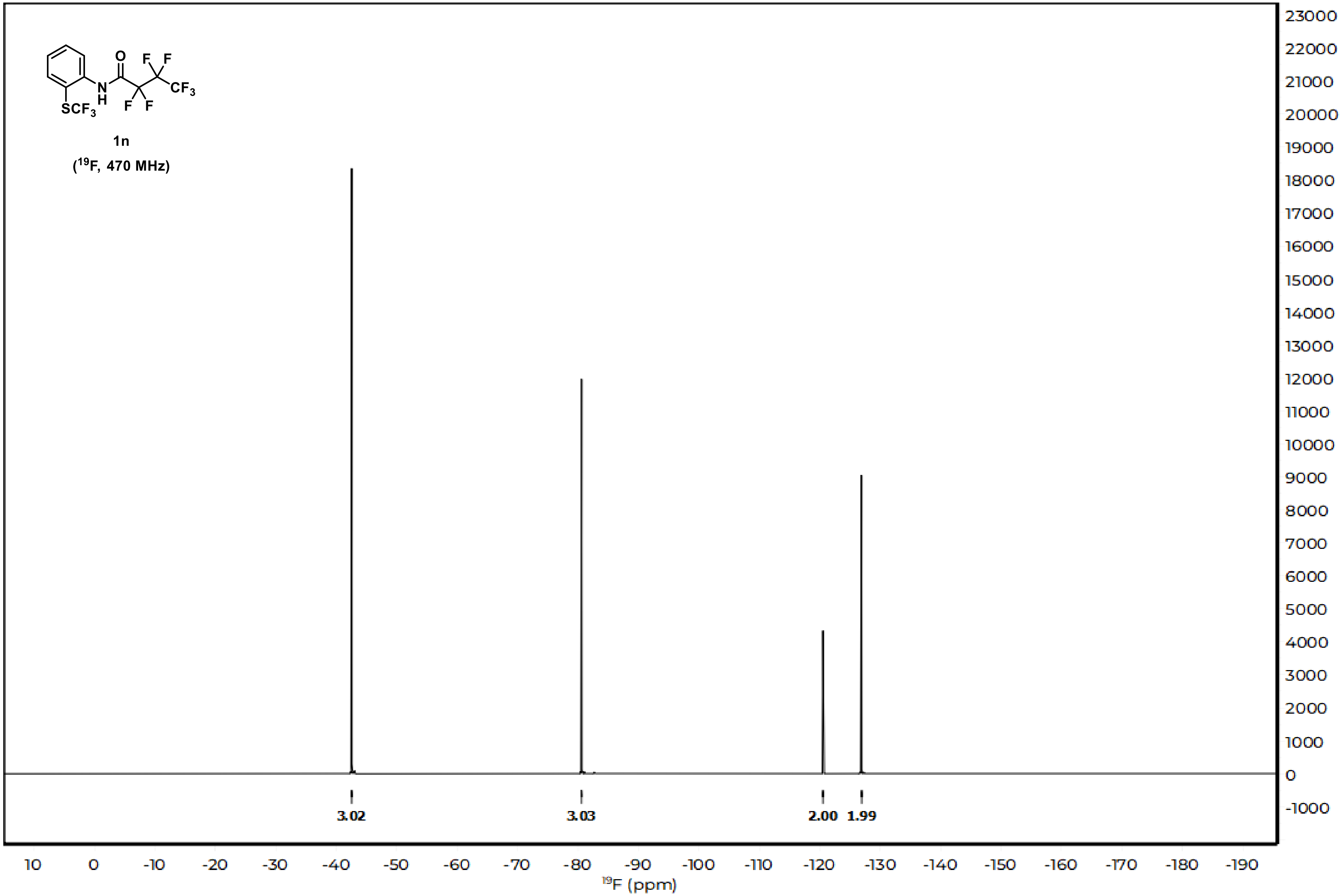

SCF3
N
H
O
F F
CF3
F F
1n
(19F, 470 MHz)
3.02
3.03
2.00
1.99
10
0
-10
-20
-30
-40
-50
-60
-70
-80
-90
-100
-110
-120
-130
-140
-150
-160
-170
-180
-190
19F (ppm)
23000
22000
21000
20000
19000
18000
17000
16000
15000
14000
13000
12000
11000
10000
9000
8000
7000
6000
5000
4000
3000
2000
1000
0
-1000

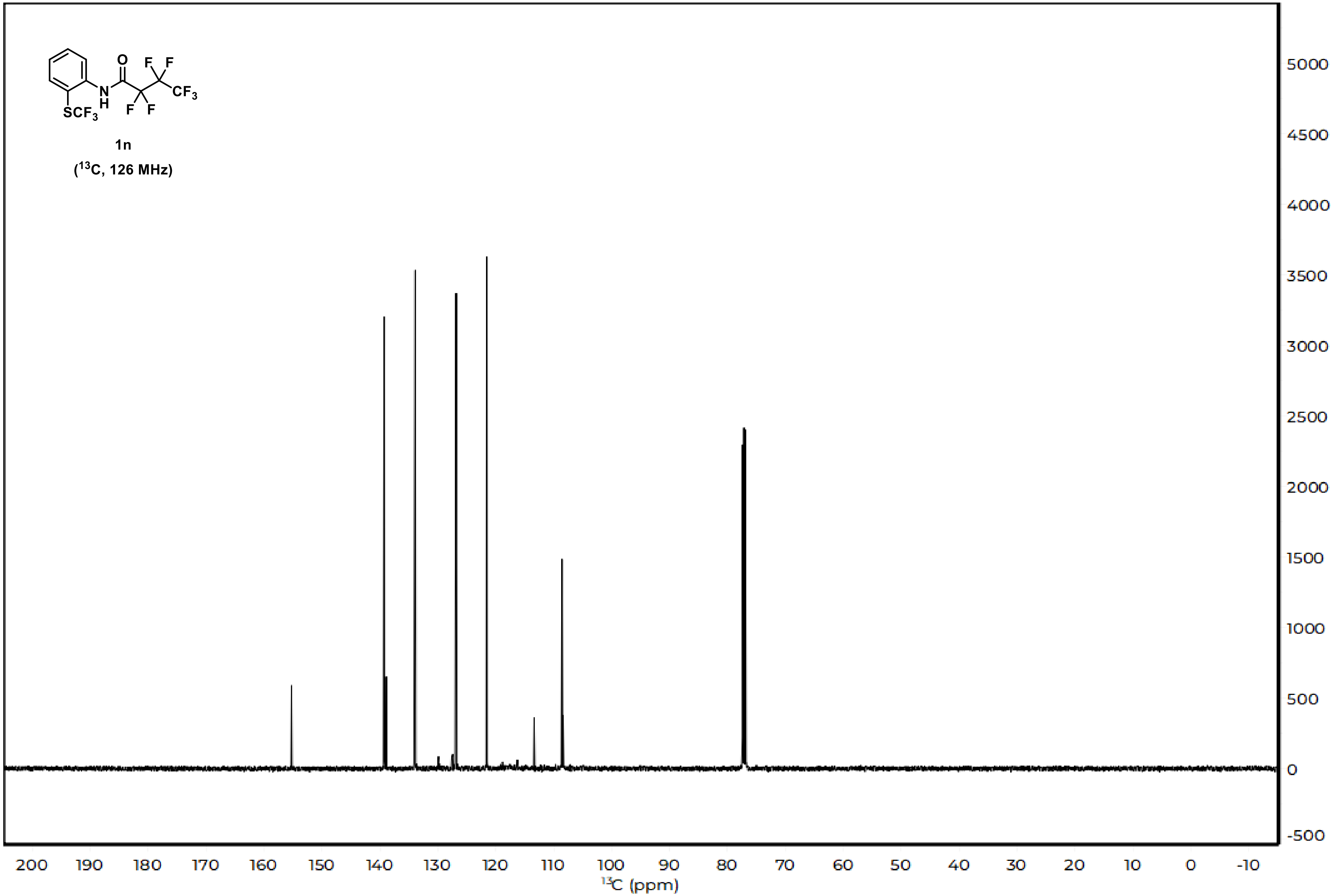

1n
(^{13}C, 126 MHz)
5000
4500
4000
3500
3000
2500
2000
1500
1000
500
0
-500
200 190 180 170 160 150 140 130 120 110 100 90 80 70 60 50 40 30 20 10 0 -10
^{13}C (ppm)

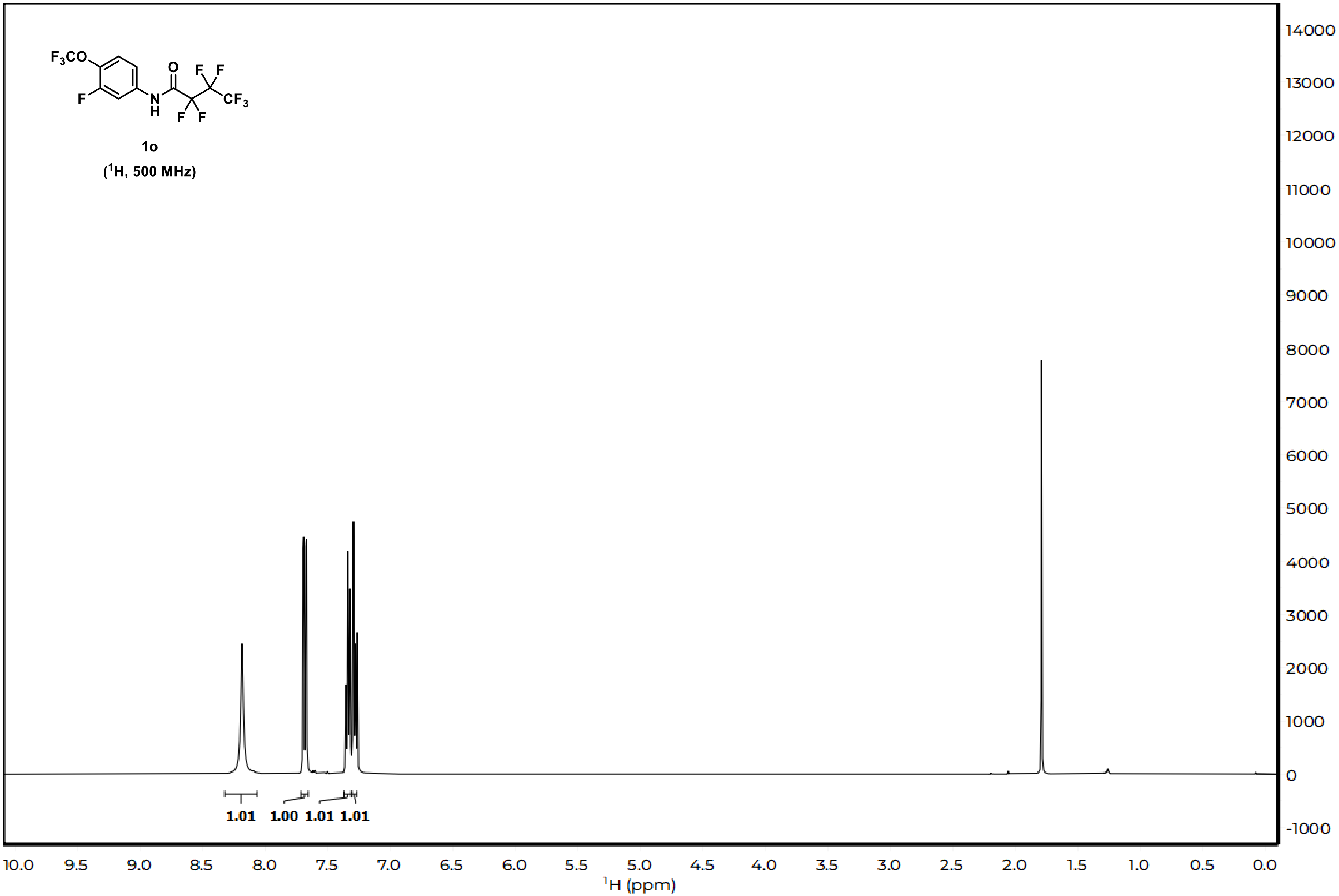
F3CO
F
N
H
O
F F
CF3
F F
1o
(1H, 500 MHz)
1.01
1.00
1.01
1.01
14000
13000
12000
11000
10000
9000
8000
7000
6000
5000
4000
3000
2000
1000
0
-1000
10.0
9.5
9.0
8.5
8.0
7.5
7.0
6.5
6.0
5.5
5.0
4.5
4.0
3.5
3.0
2.5
2.0
1.5
1.0
0.5
0.0
1H (ppm)

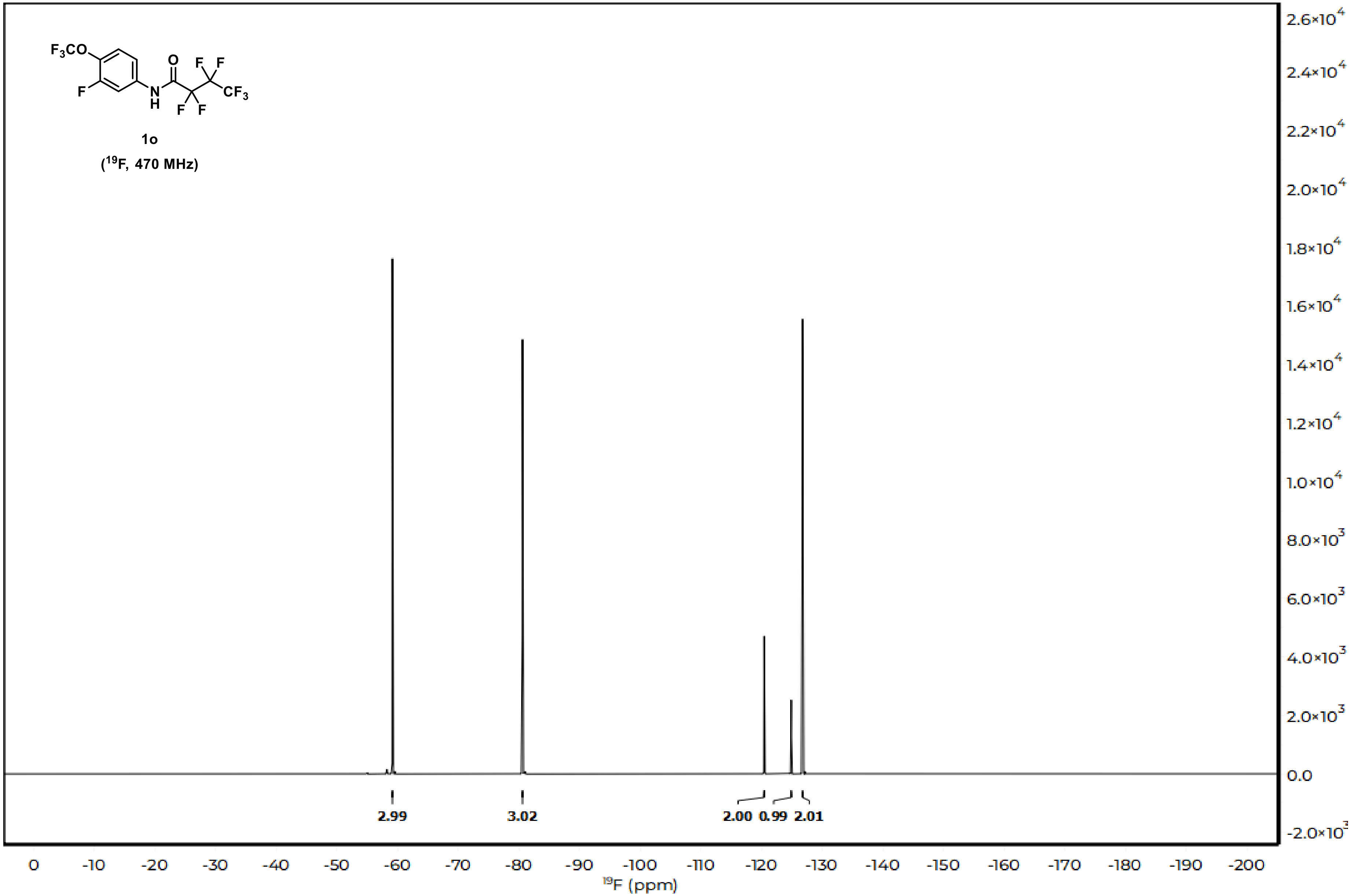

F3CO
F
N
H
O
F F
CF3
F F
1o
(19F, 470 MHz)
2.99
3.02
2.00 0.99 2.01
0
-10
-20
-30
-40
-50
-60
-70
-80
-90
-100
-110
-120
-130
-140
-150
-160
-170
-180
-190
-200
19F (ppm)
2.6×10^4
2.4×10^4
2.2×10^4
2.0×10^4
1.8×10^4
1.6×10^4
1.4×10^4
1.2×10^4
1.0×10^4
8.0×10^3
6.0×10^3
4.0×10^3
2.0×10^3
0.0
-2.0×10^3

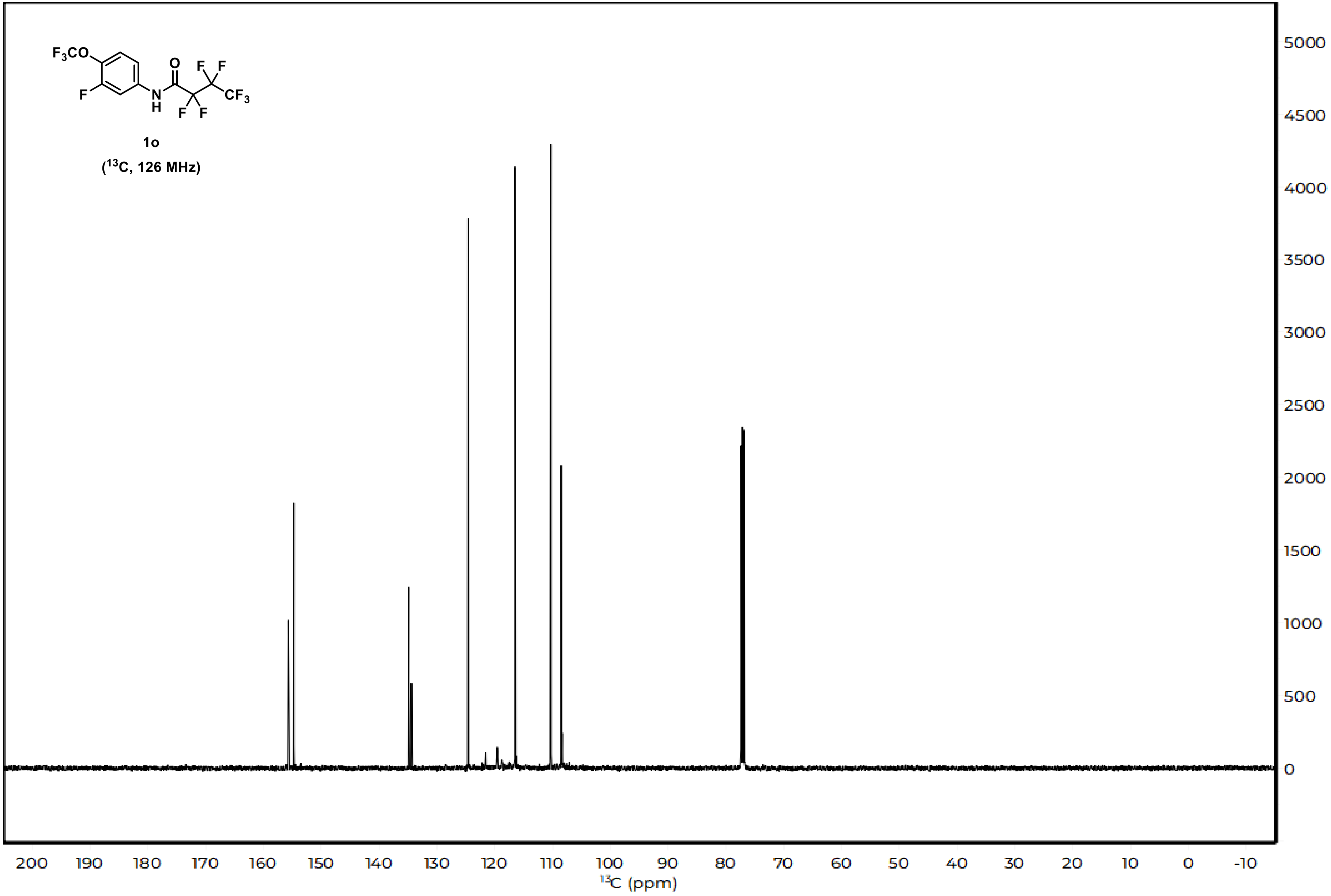

F3CO
F
N
H
O
F F
CF3
F F
1o
(13C, 126 MHz)
5000
4500
4000
3500
3000
2500
2000
1500
1000
500
0
200 190 180 170 160 150 140 130 120 110 100 90 80 70 60 50 40 30 20 10 0 -10
13C (ppm)

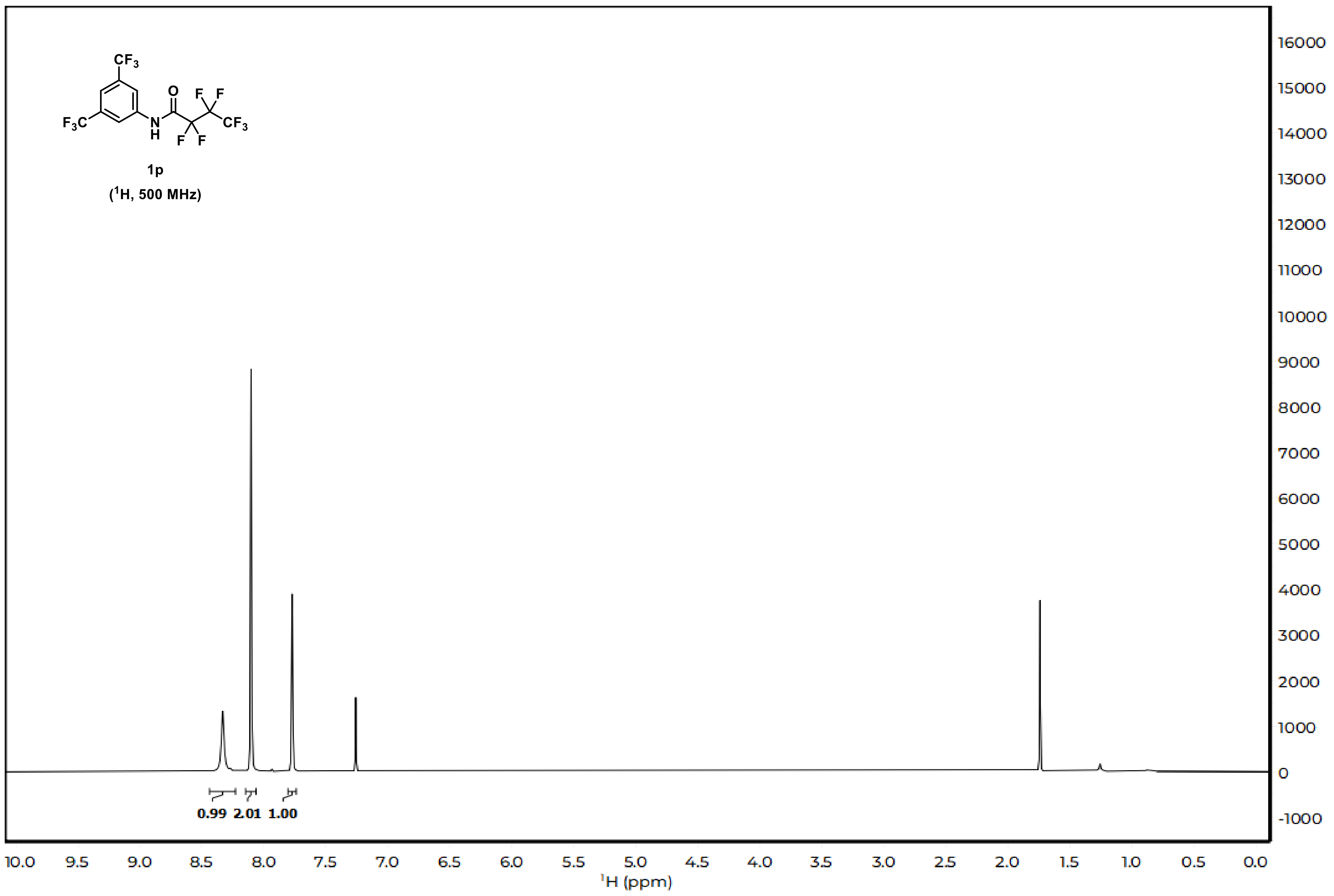

CF3
F3C
N
H
O
F F
CF3
F F
1p
(1H, 500 MHz)
0.99 2.01 1.00
1H (ppm)
10.0 9.5 9.0 8.5 8.0 7.5 7.0 6.5 6.0 5.5 5.0 4.5 4.0 3.5 3.0 2.5 2.0 1.5 1.0 0.5 0.0
16000 15000 14000 13000 12000 11000 10000 9000 8000 7000 6000 5000 4000 3000 2000 1000 0 -1000

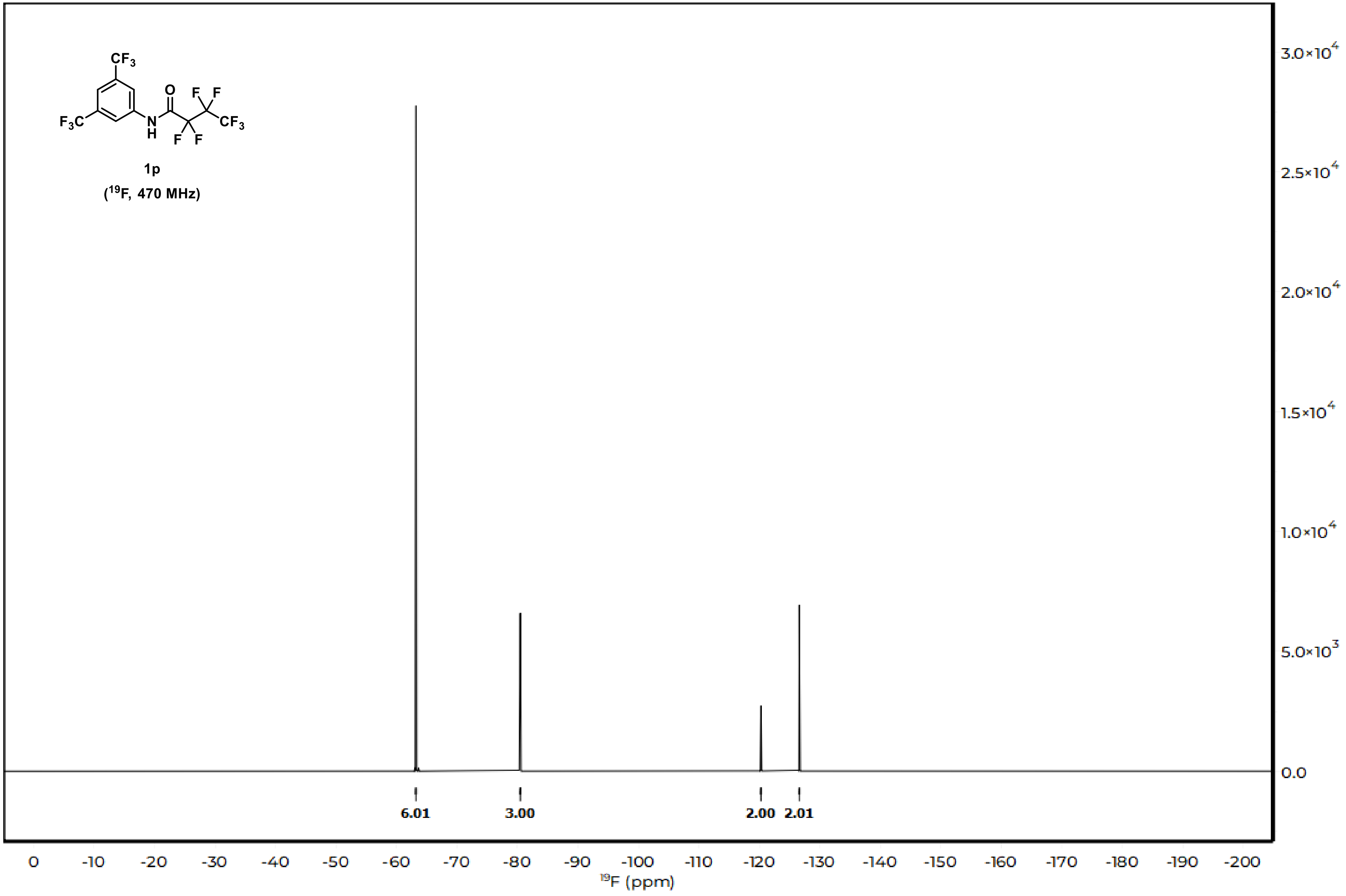
CF3
F3C
O
F F
N
H
CF3
F F
1p
(19F, 470 MHz)
3.0×10^4
2.5×10^4
2.0×10^4
1.5×10^4
1.0×10^4
5.0×10^3
0.0
6.01
3.00
2.00
2.01
0 -10 -20 -30 -40 -50 -60 -70 -80 -90 -100 -110 -120 -130 -140 -150 -160 -170 -180 -190 -200
19F (ppm)

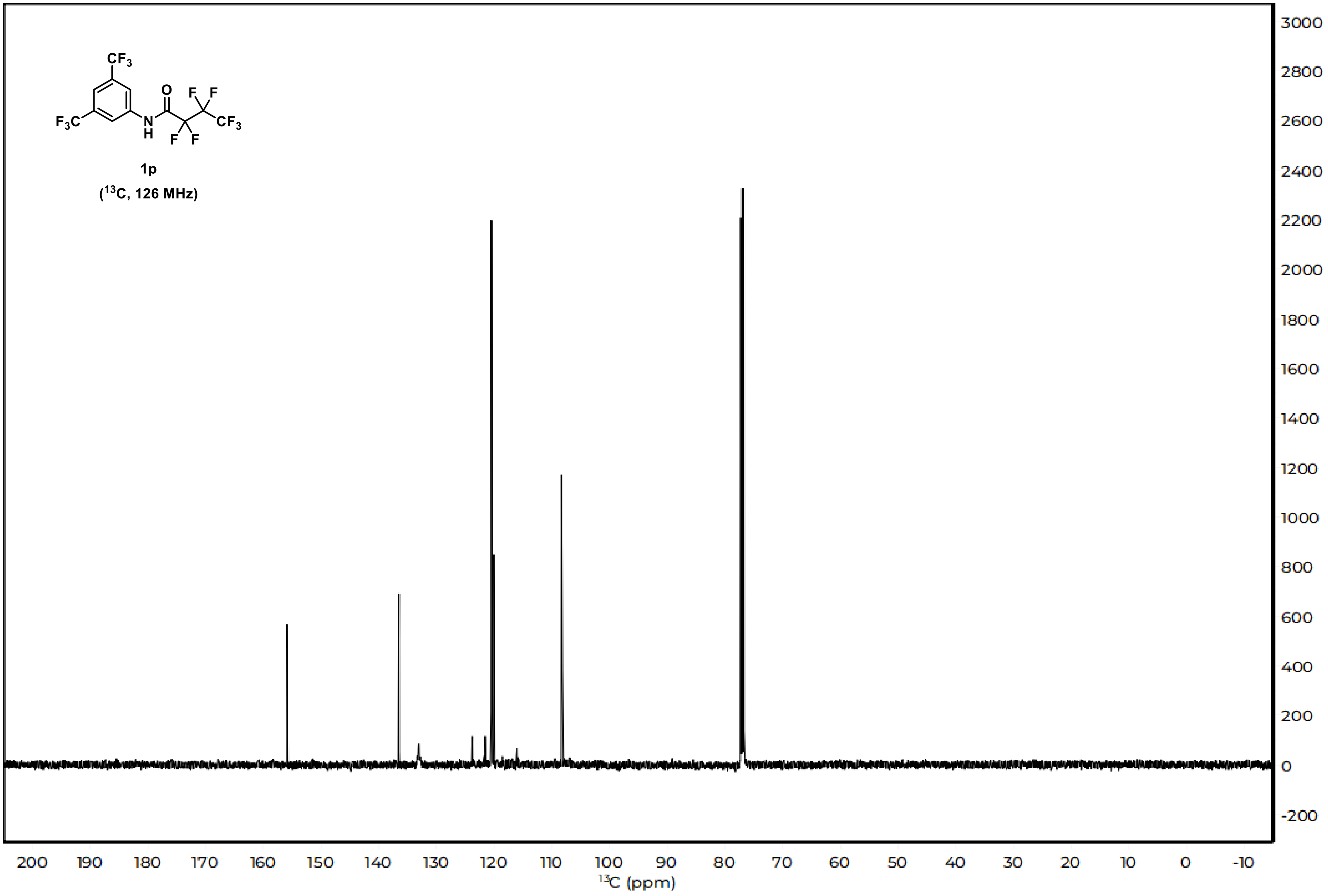
CF3
O
F F
F3C
N
H
CF3
F F
1p
(13C, 126 MHz)
3000
2800
2600
2400
2200
2000
1800
1600
1400
1200
1000
800
600
400
200
0
-200
200 190 180 170 160 150 140 130 120 110 100 90 80 70 60 50 40 30 20 10 0 -10
13C (ppm)

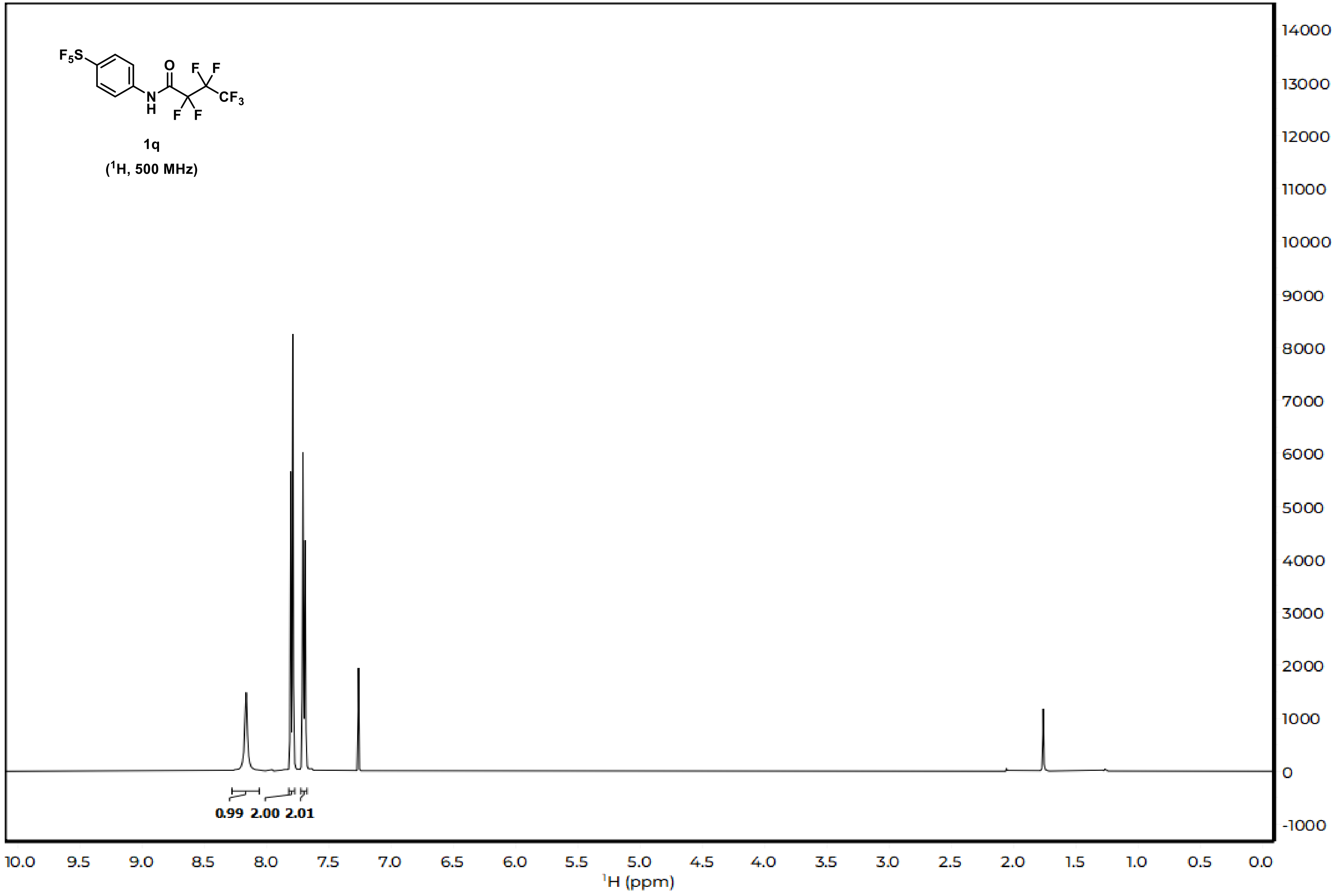

F5S
O
F F
N
H
CF3
F F
1q
(1H, 500 MHz)
14000
13000
12000
11000
10000
9000
8000
7000
6000
5000
4000
3000
2000
1000
0
-1000
0.99 2.00 2.01
10.0 9.5 9.0 8.5 8.0 7.5 7.0 6.5 6.0 5.5 5.0 4.5 4.0 3.5 3.0 2.5 2.0 1.5 1.0 0.5 0.0
1H (ppm)

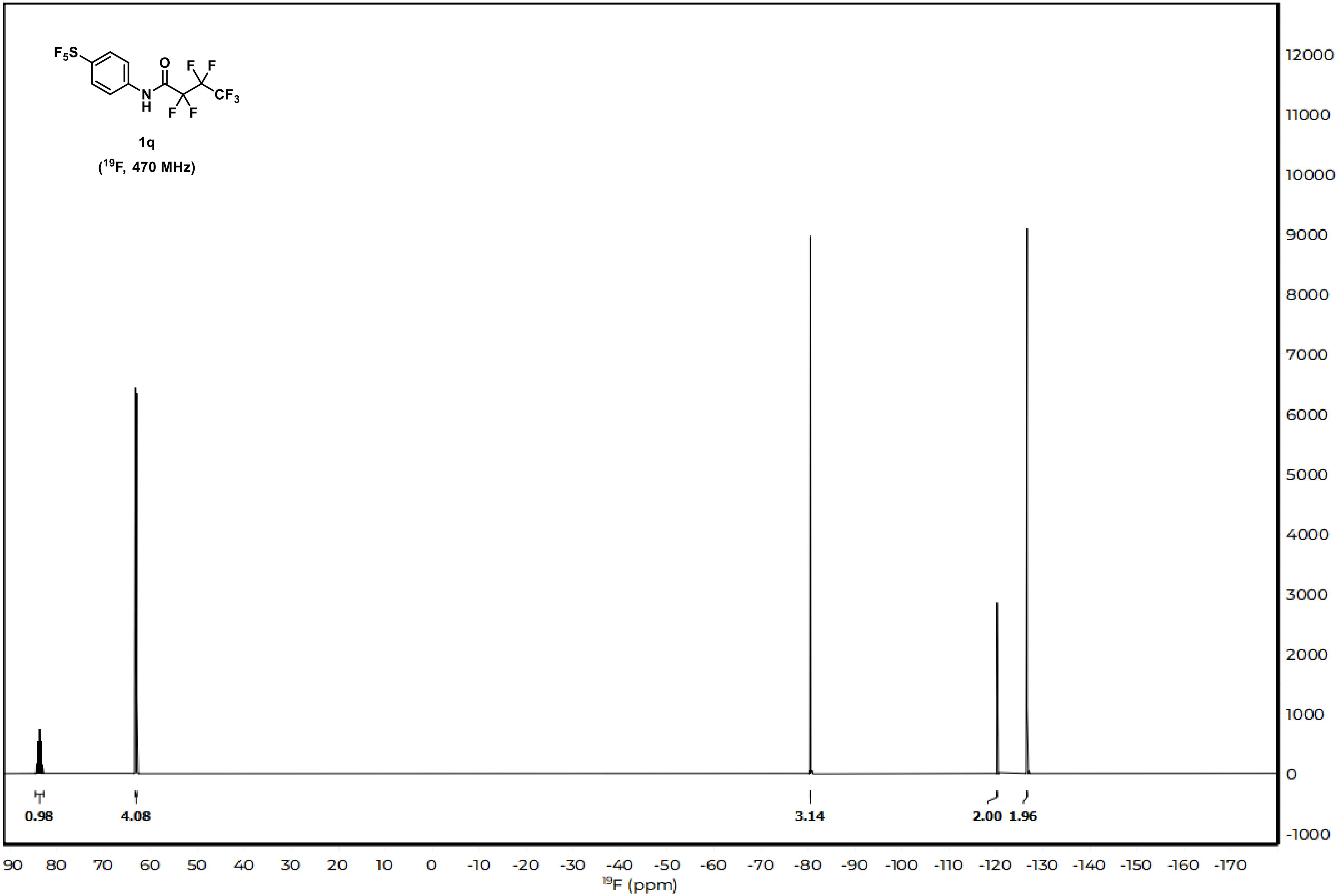
F5S
O
F F
N
H
F F
CF3
1q
(19F, 470 MHz)
12000
11000
10000
9000
8000
7000
6000
5000
4000
3000
2000
1000
0
-1000
0.98
4.08
3.14
2.00 1.96
90 80 70 60 50 40 30 20 10 0 -10 -20 -30 -40 -50 -60 -70 -80 -90 -100 -110 -120 -130 -140 -150 -160 -170
19F (ppm)

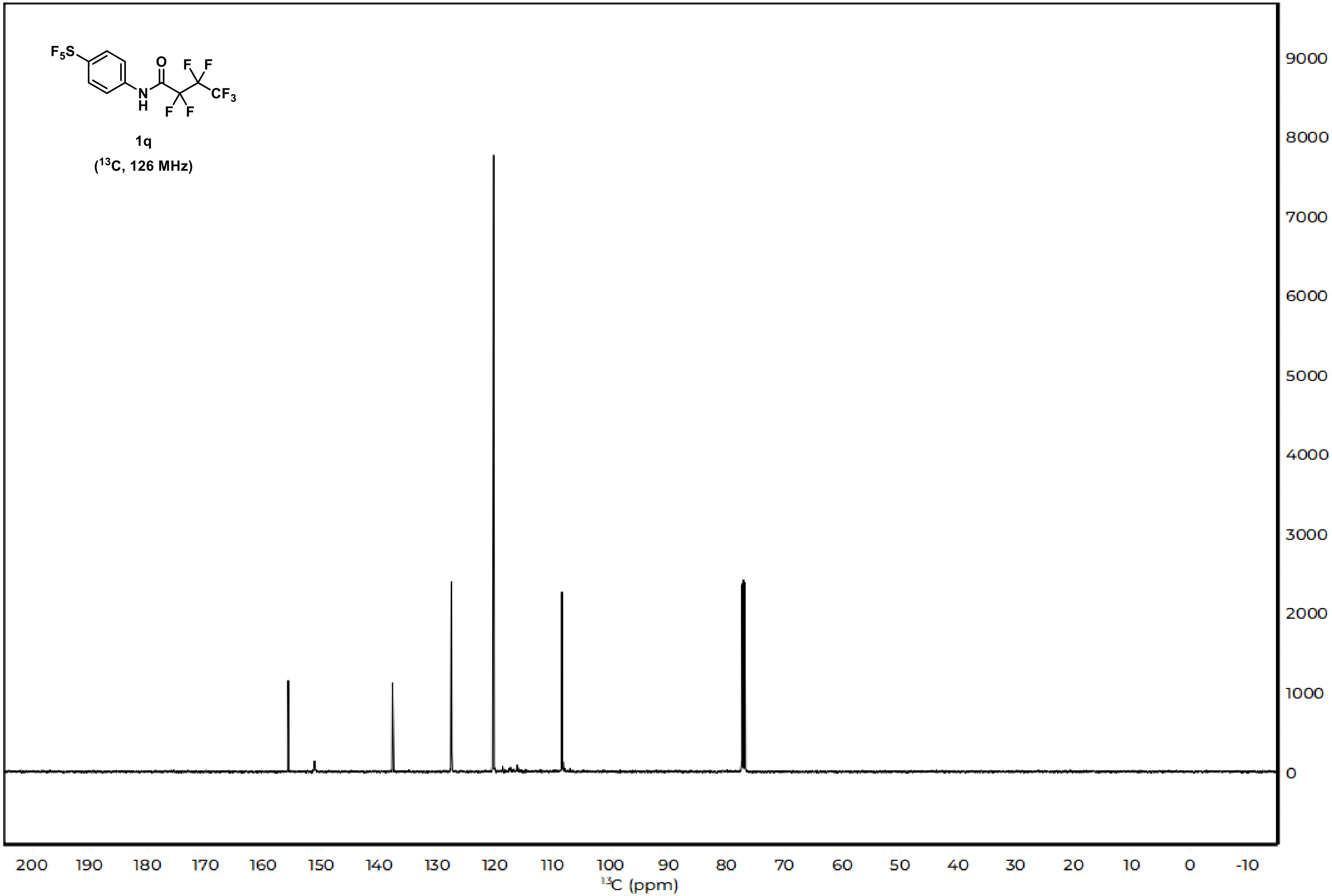

F5S
O
F F
N
H
CF3
F F
1q
(13C, 126 MHz)
9000
8000
7000
6000
5000
4000
3000
2000
1000
0
200 190 180 170 160 150 140 130 120 110 100 90 80 70 60 50 40 30 20 10 0 -10
13C (ppm)

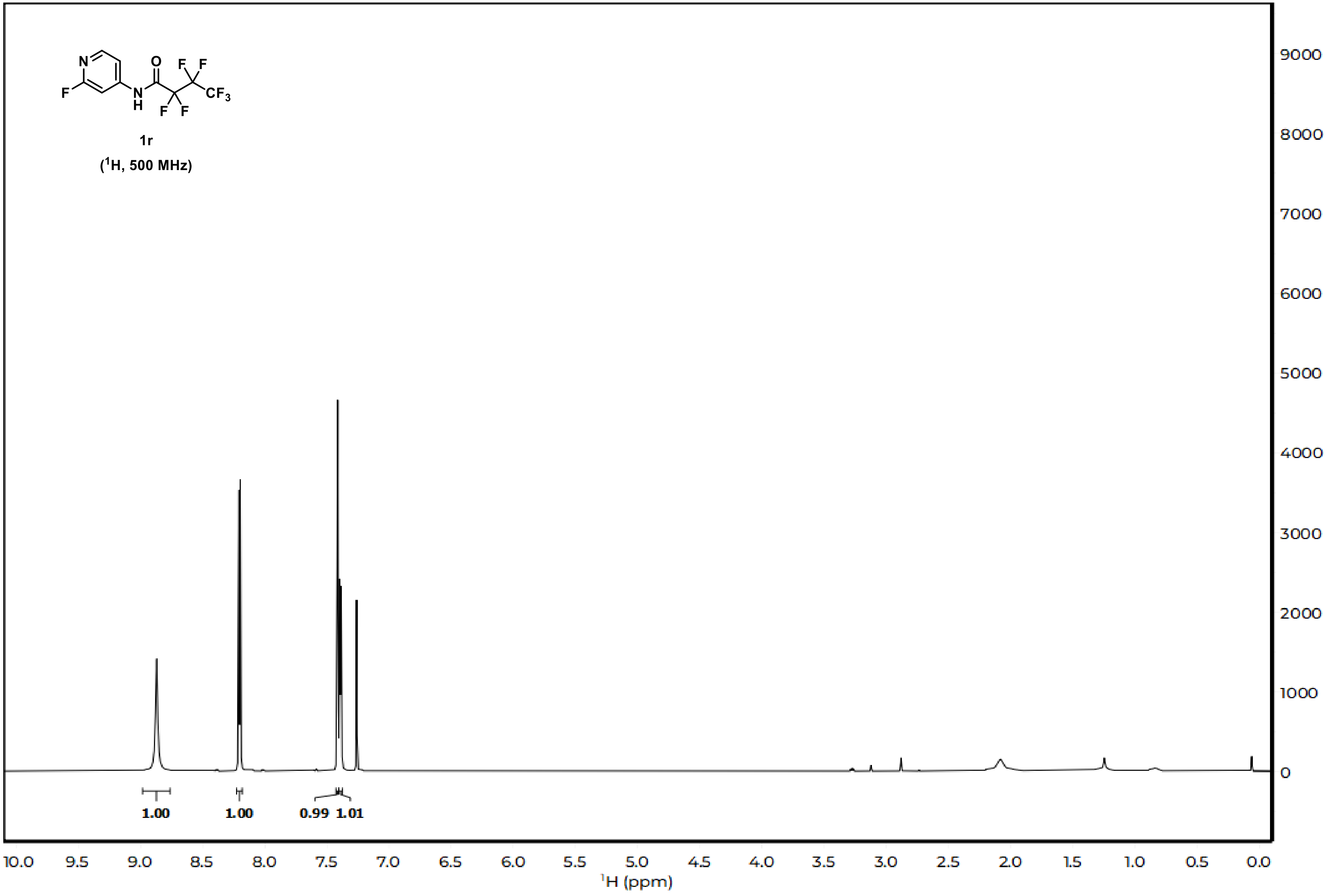
1r
(¹H, 500 MHz)
1.00
1.00
0.99
1.01
10.0 9.5 9.0 8.5 8.0 7.5 7.0 6.5 6.0 5.5 5.0 4.5 4.0 3.5 3.0 2.5 2.0 1.5 1.0 0.5 0.0
¹H (ppm)
9000 8000 7000 6000 5000 4000 3000 2000 1000 0

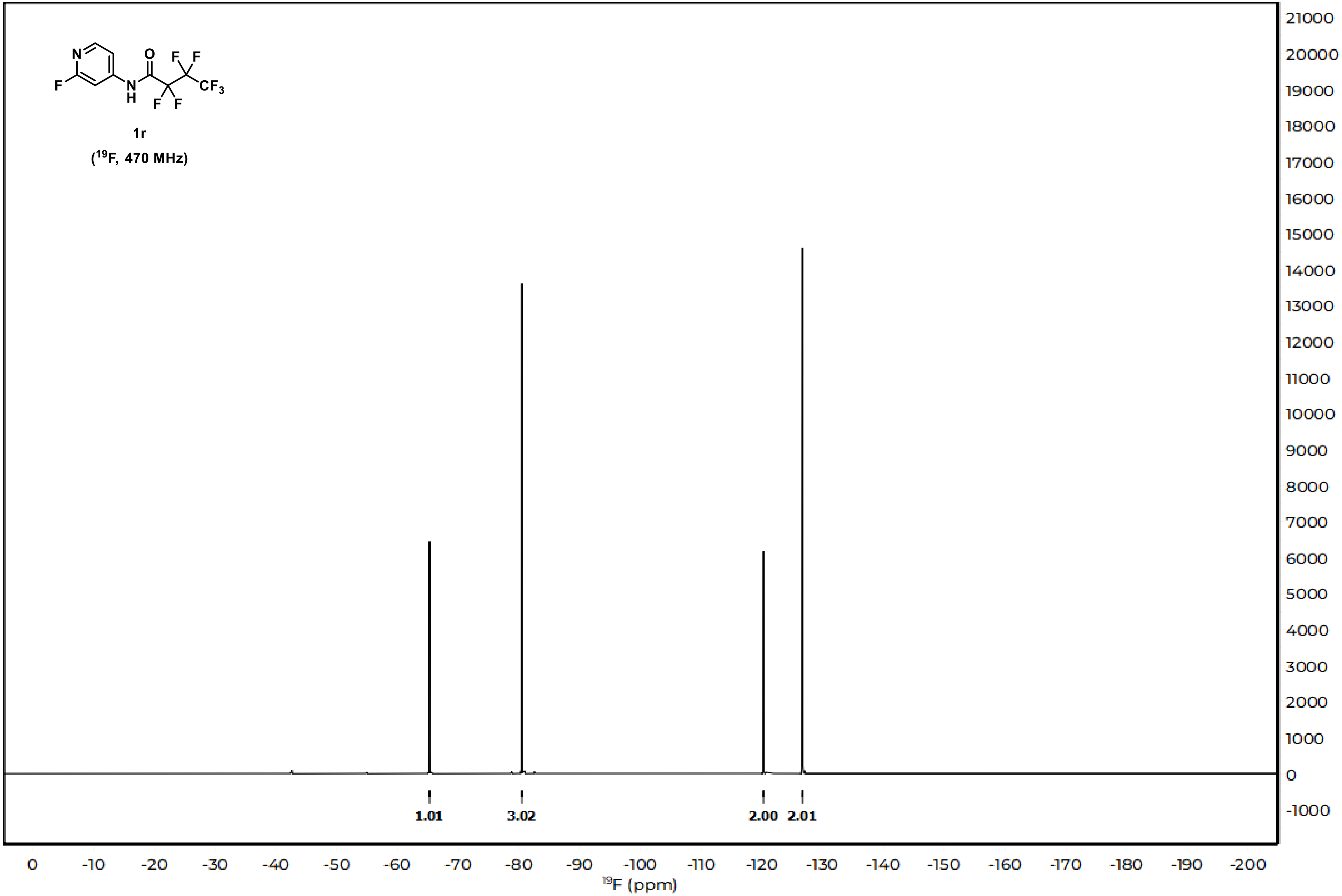
1r
(19F, 470 MHz)
1.01
3.02
2.00
2.01
19F (ppm)

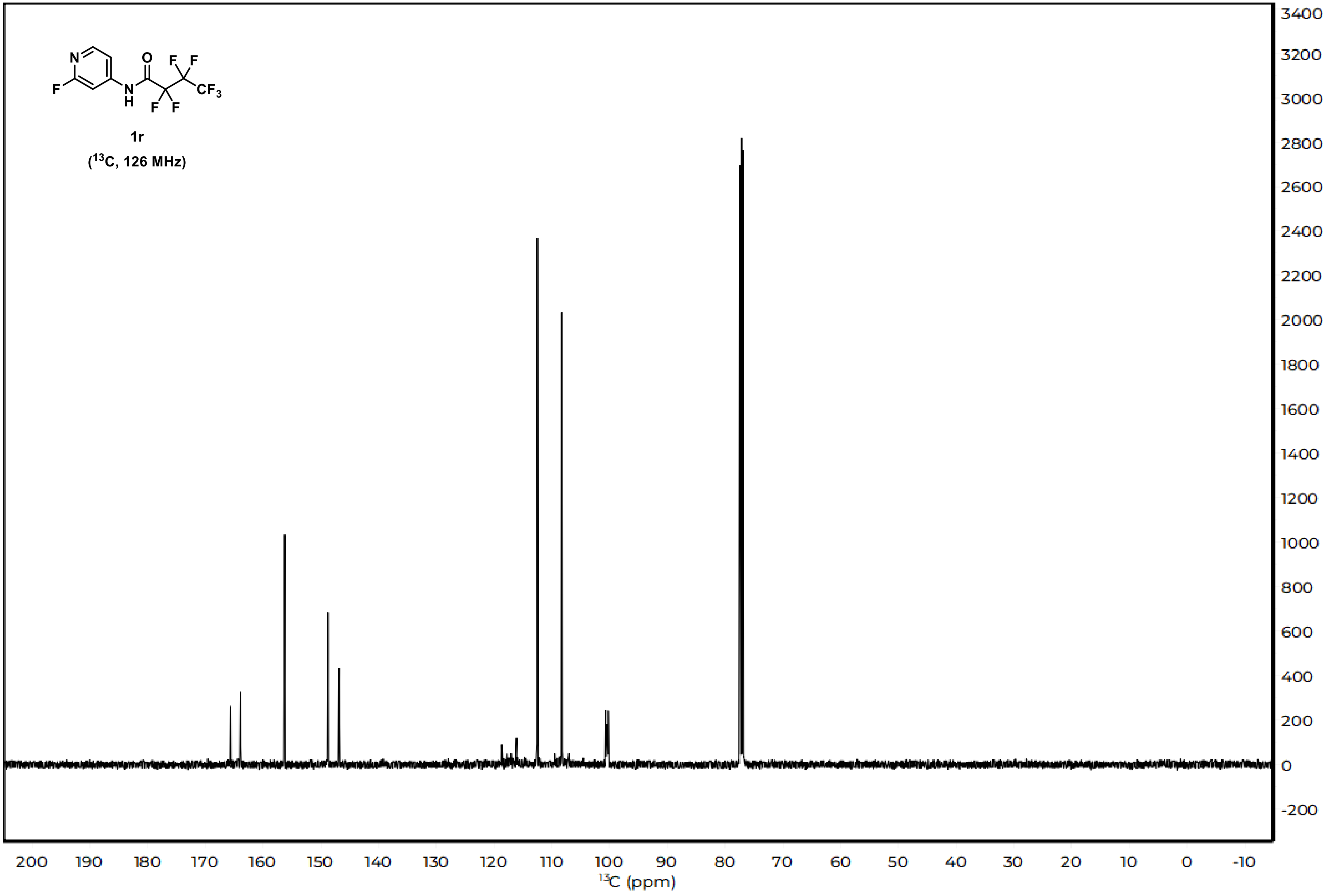

N
O
F
F
F
N
H
CF3
F
F
1r
(13C, 126 MHz)
3400
3200
3000
2800
2600
2400
2200
2000
1800
1600
1400
1200
1000
800
600
400
200
0
-200
200
190
180
170
160
150
140
130
120
110
100
90
80
70
60
50
40
30
20
10
0
-10
13C (ppm)

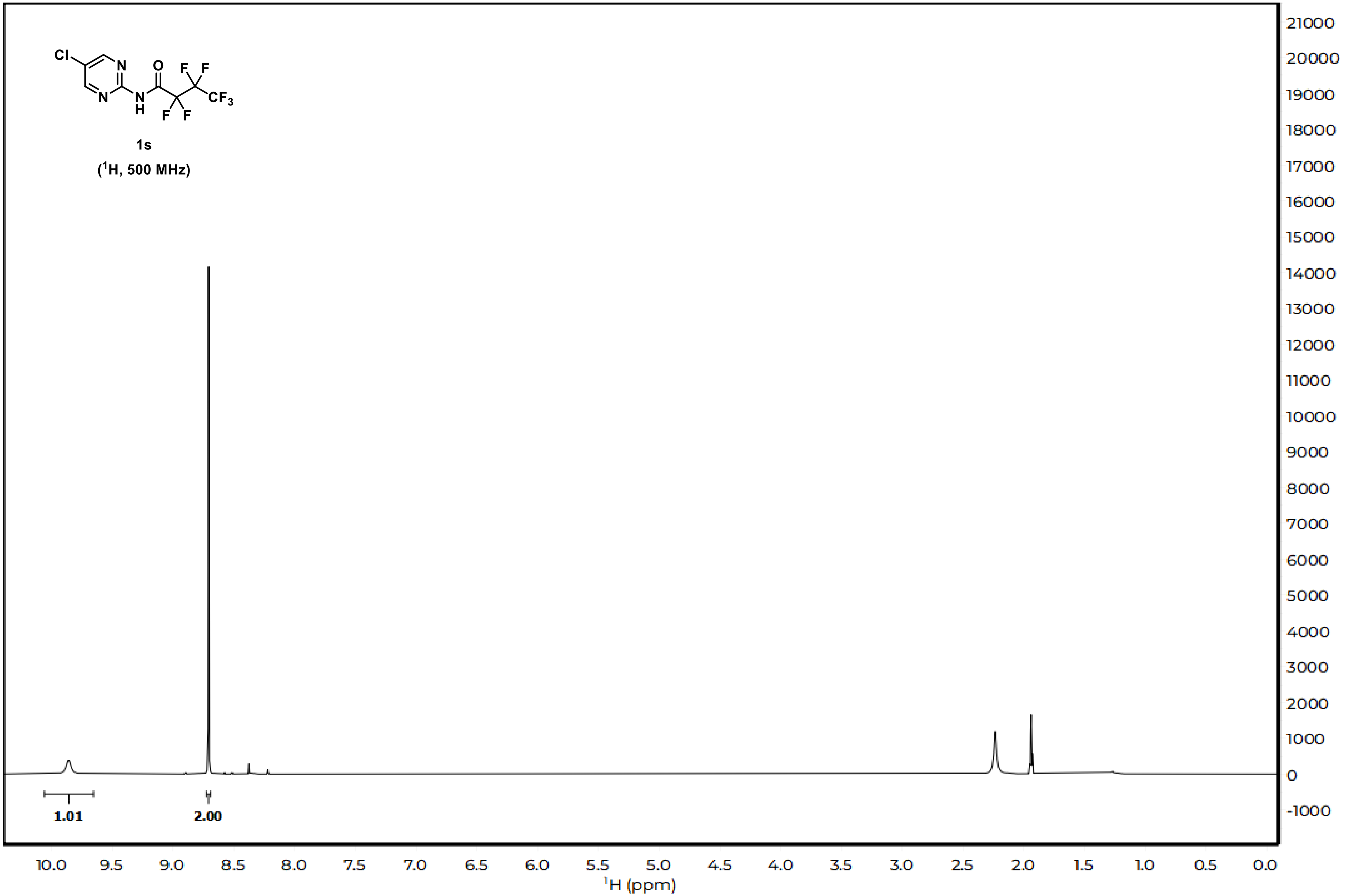

Cl
N
N
N
H
O
F
F
F
F
CF3
1s
(1H, 500 MHz)
1.01
2.00
21000
20000
19000
18000
17000
16000
15000
14000
13000
12000
11000
10000
9000
8000
7000
6000
5000
4000
3000
2000
1000
0
-1000
10.0
9.5
9.0
8.5
8.0
7.5
7.0
6.5
6.0
5.5
5.0
4.5
4.0
3.5
3.0
2.5
2.0
1.5
1.0
0.5
0.0
1H (ppm)

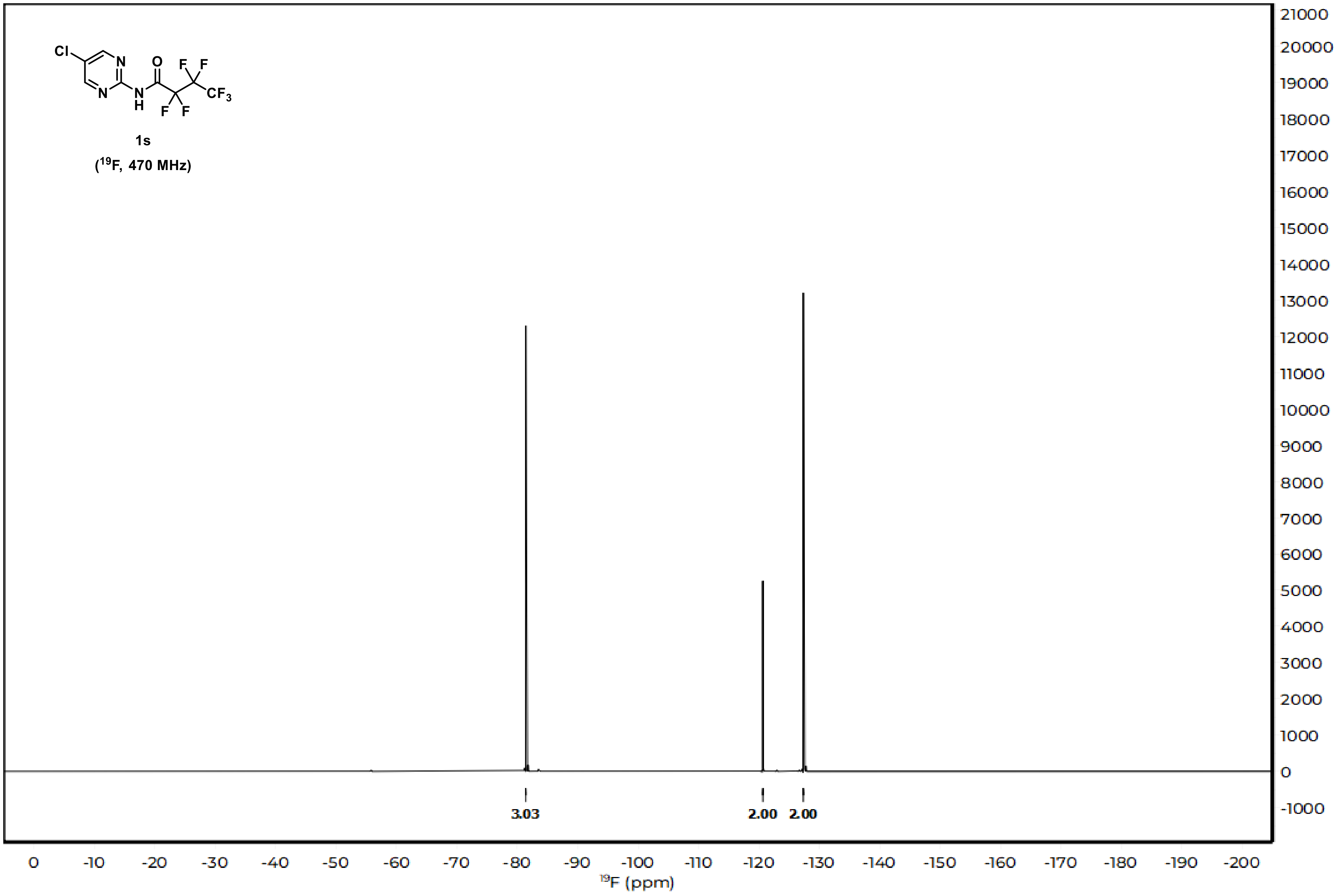
Cl
N
N
O
F F
N
H
CF3
F F
1s
(19F, 470 MHz)
3.03
2.00
2.00
0
-10
-20
-30
-40
-50
-60
-70
-80
-90
-100
-110
-120
-130
-140
-150
-160
-170
-180
-190
-200
19F (ppm)
21000
20000
19000
18000
17000
16000
15000
14000
13000
12000
11000
10000
9000
8000
7000
6000
5000
4000
3000
2000
1000
0
-1000

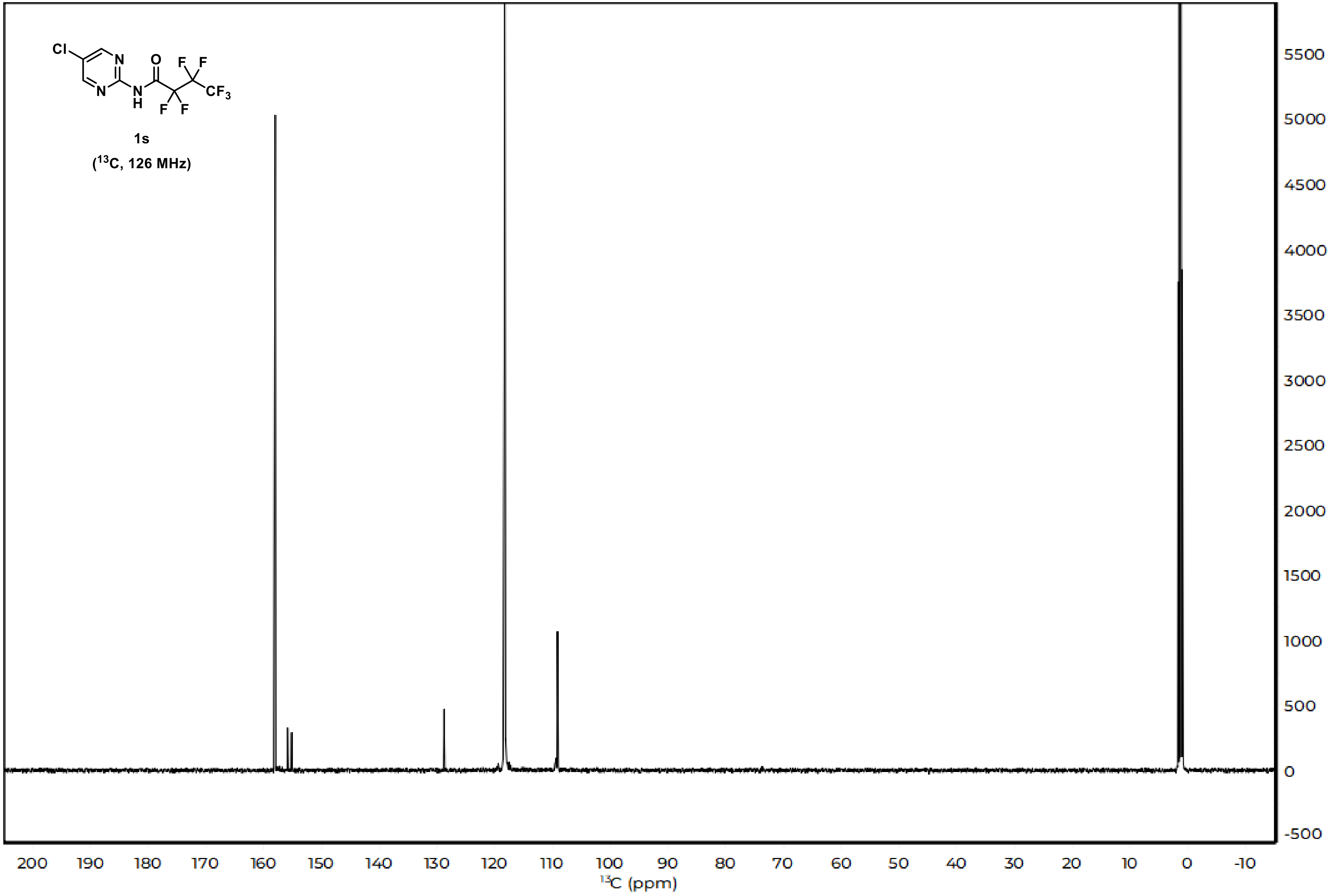
Cl
N
N
N
H
O
F
F
F
F
CF3
1s
(13C, 126 MHz)
5500
5000
4500
4000
3500
3000
2500
2000
1500
1000
500
0
-500
200
190
180
170
160
150
140
130
120
110
100
90
80
70
60
50
40
30
20
10
0
-10
13C (ppm)

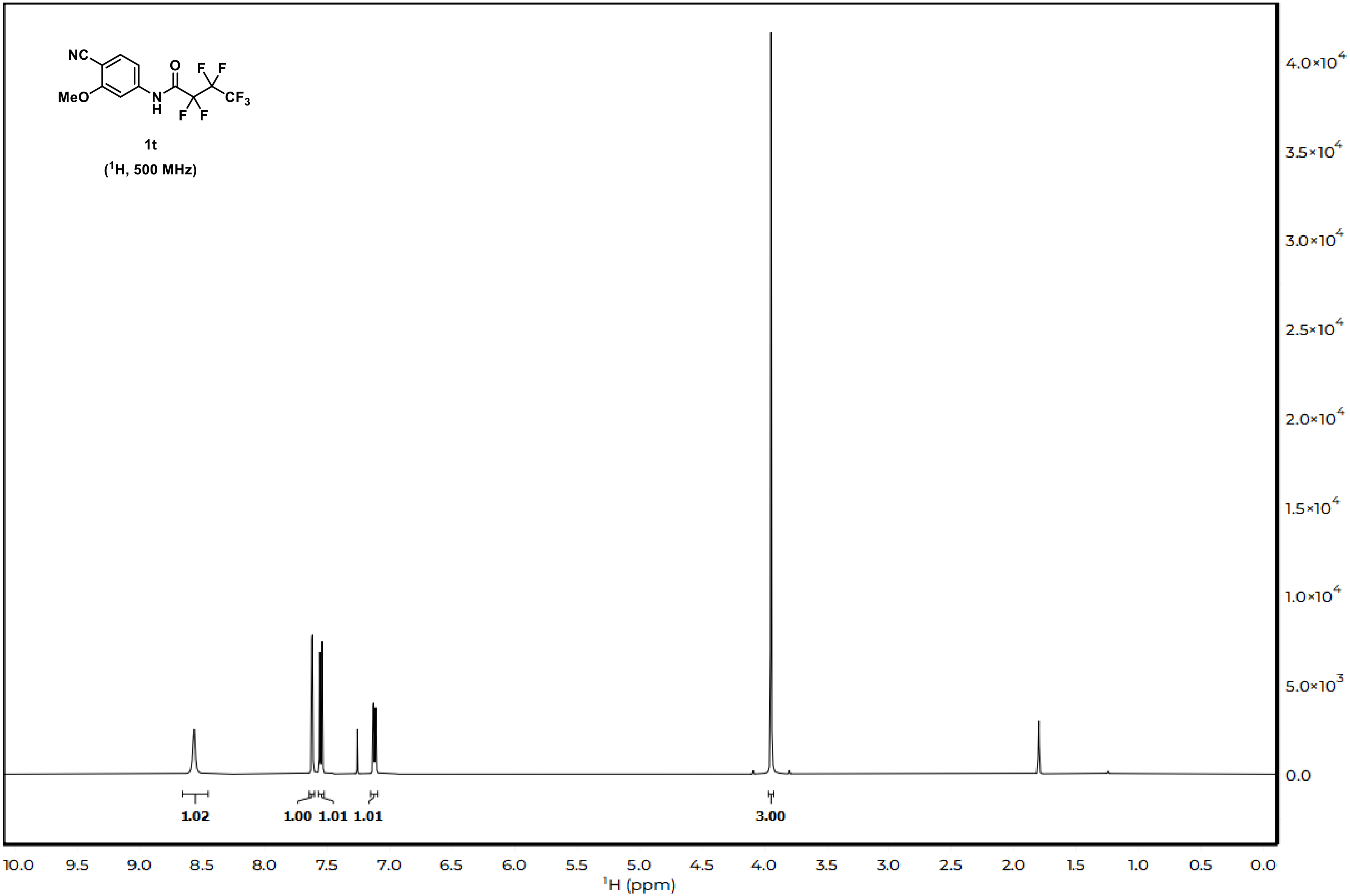
NC
MeO
O
F F
CF3
N
H
F F
1t
(1H, 500 MHz)
4.0×10^4
3.5×10^4
3.0×10^4
2.5×10^4
2.0×10^4
1.5×10^4
1.0×10^4
5.0×10^3
0.0
1.02
1.00 1.01 1.01
3.00
10.0 9.5 9.0 8.5 8.0 7.5 7.0 6.5 6.0 5.5 5.0 4.5 4.0 3.5 3.0 2.5 2.0 1.5 1.0 0.5 0.0
1H (ppm)

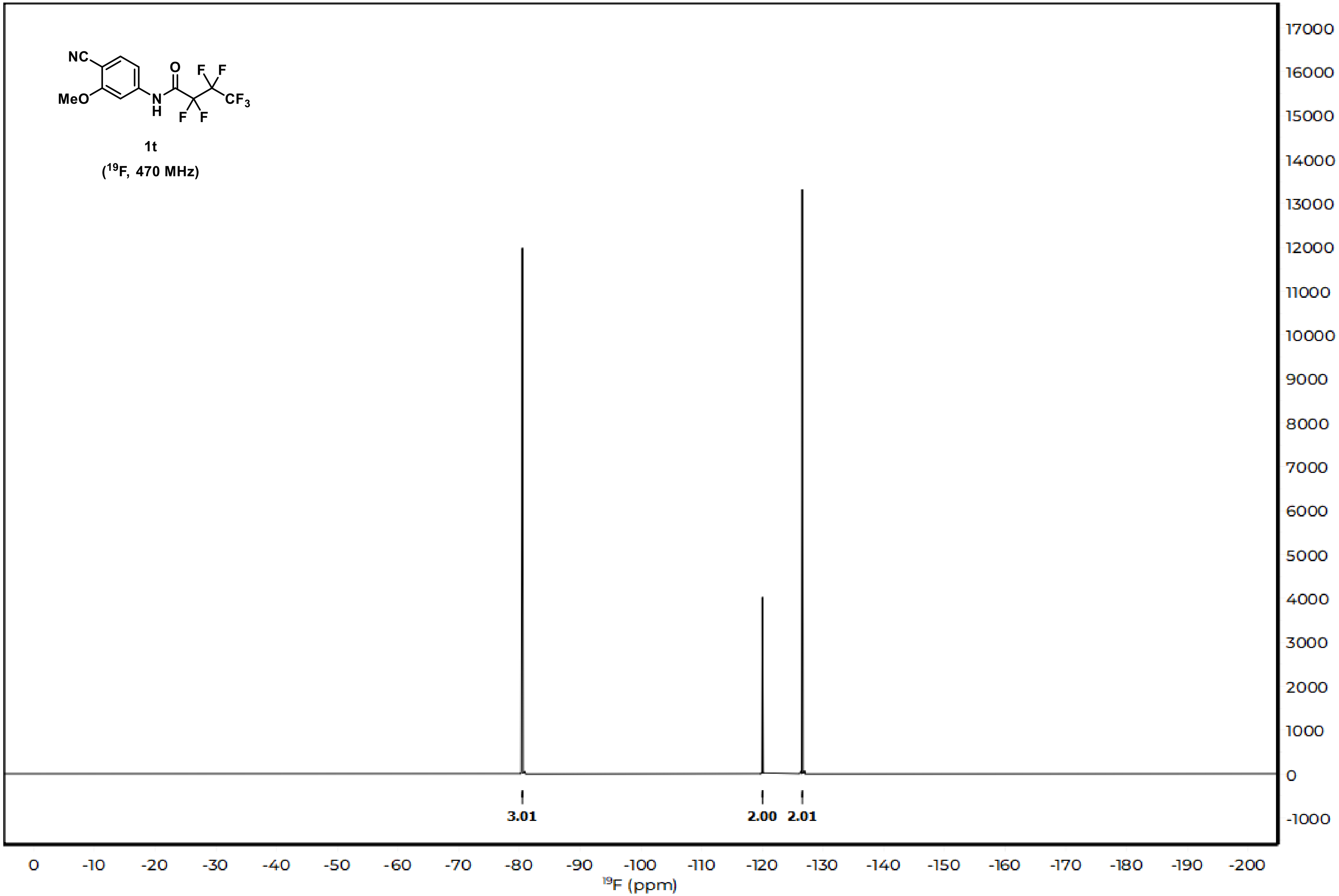

NC
MeO
O
F
F
CF3
N
H
1t
(19F, 470 MHz)
3.01
2.00
2.01
19F (ppm)

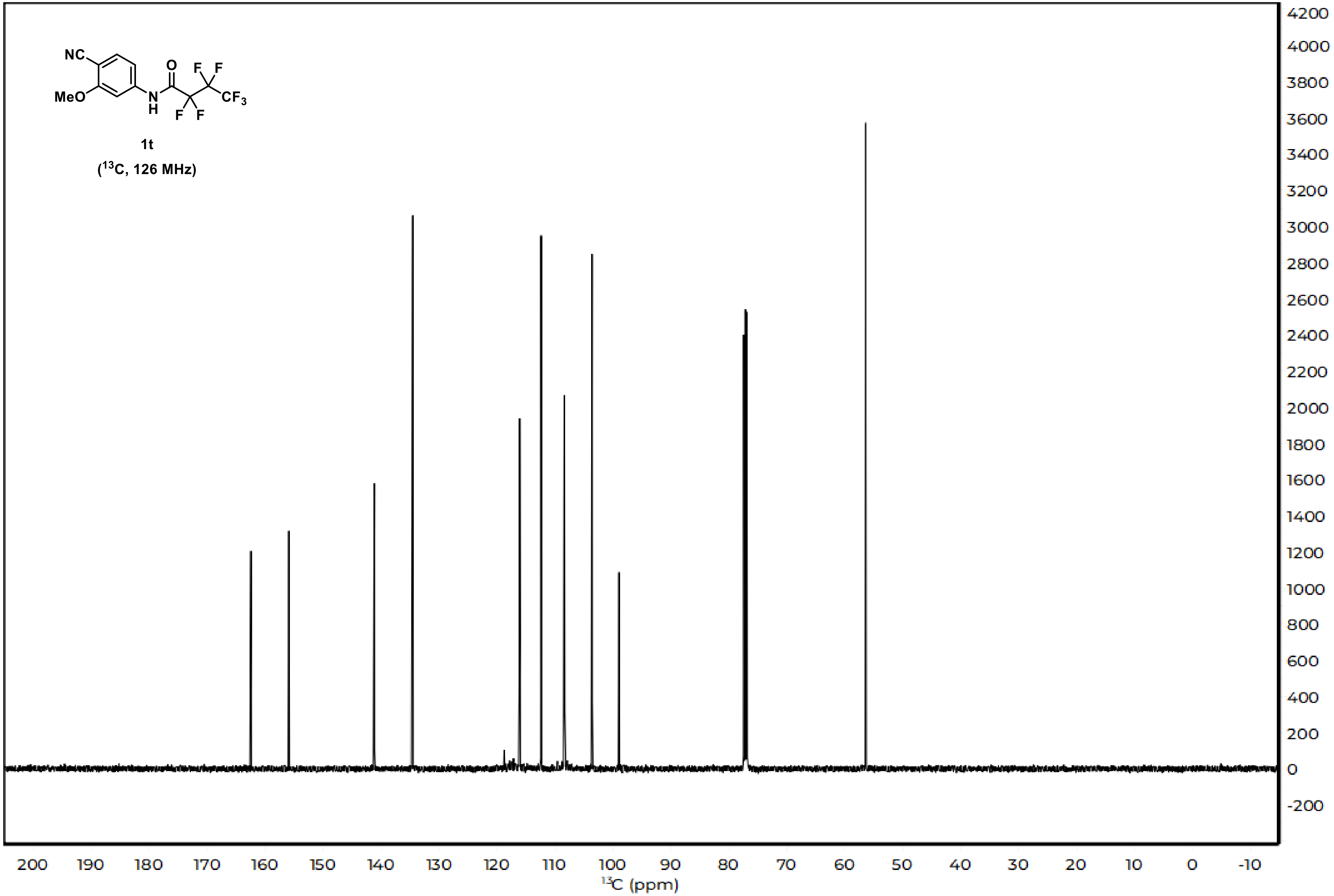

NC
MeO
O
F F
CF3
N
H
F F
1t
(13C, 126 MHz)
4200
4000
3800
3600
3400
3200
3000
2800
2600
2400
2200
2000
1800
1600
1400
1200
1000
800
600
400
200
0
-200
200 190 180 170 160 150 140 130 120 110 100 90 80 70 60 50 40 30 20 10 0 -10
13C (ppm)

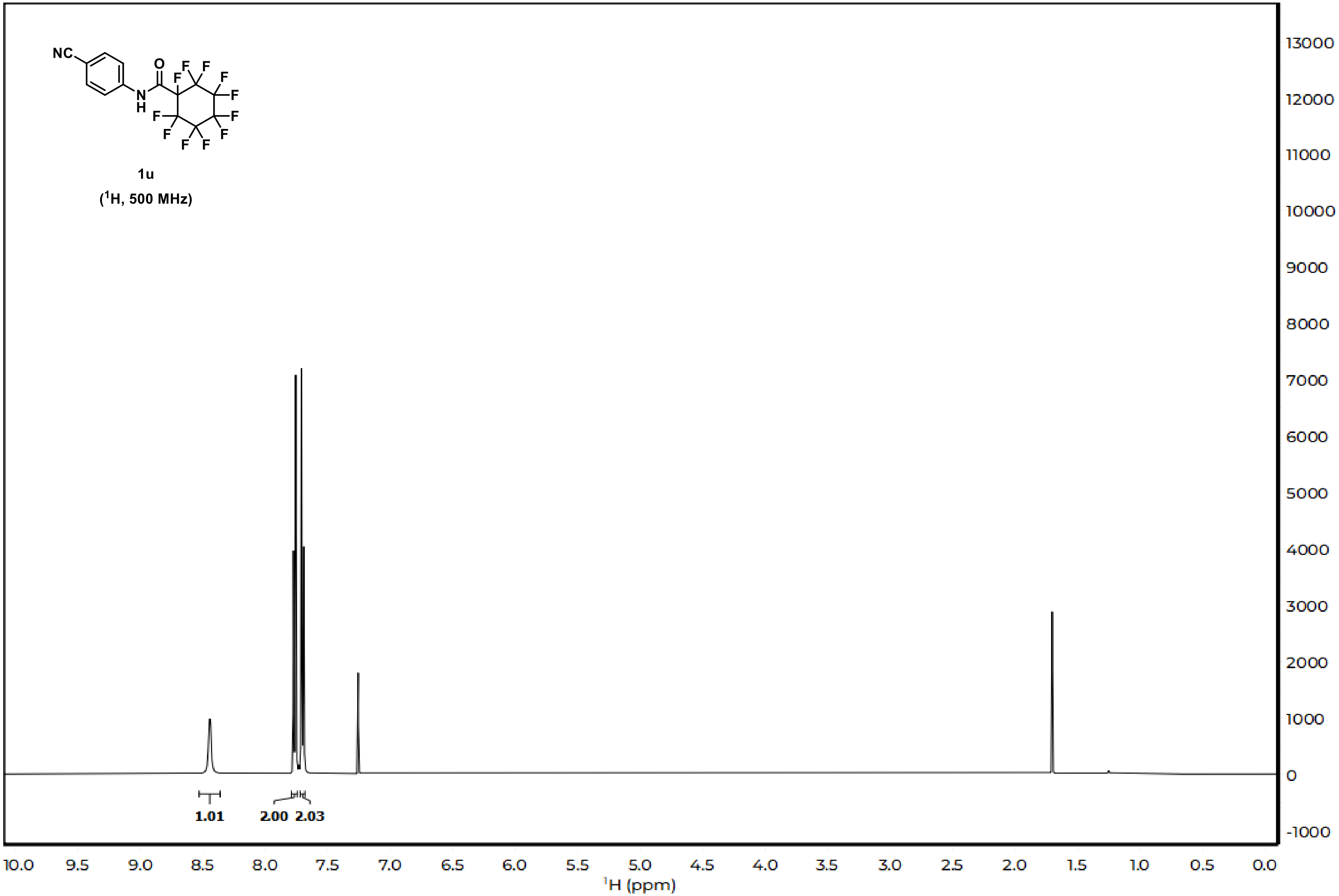
NC
O
N
H
F
1u
(1H, 500 MHz)
13000
12000
11000
10000
9000
8000
7000
6000
5000
4000
3000
2000
1000
0
-1000
1.01
2.00 2.03
10.0 9.5 9.0 8.5 8.0 7.5 7.0 6.5 6.0 5.5 5.0 4.5 4.0 3.5 3.0 2.5 2.0 1.5 1.0 0.5 0.0
1H (ppm)

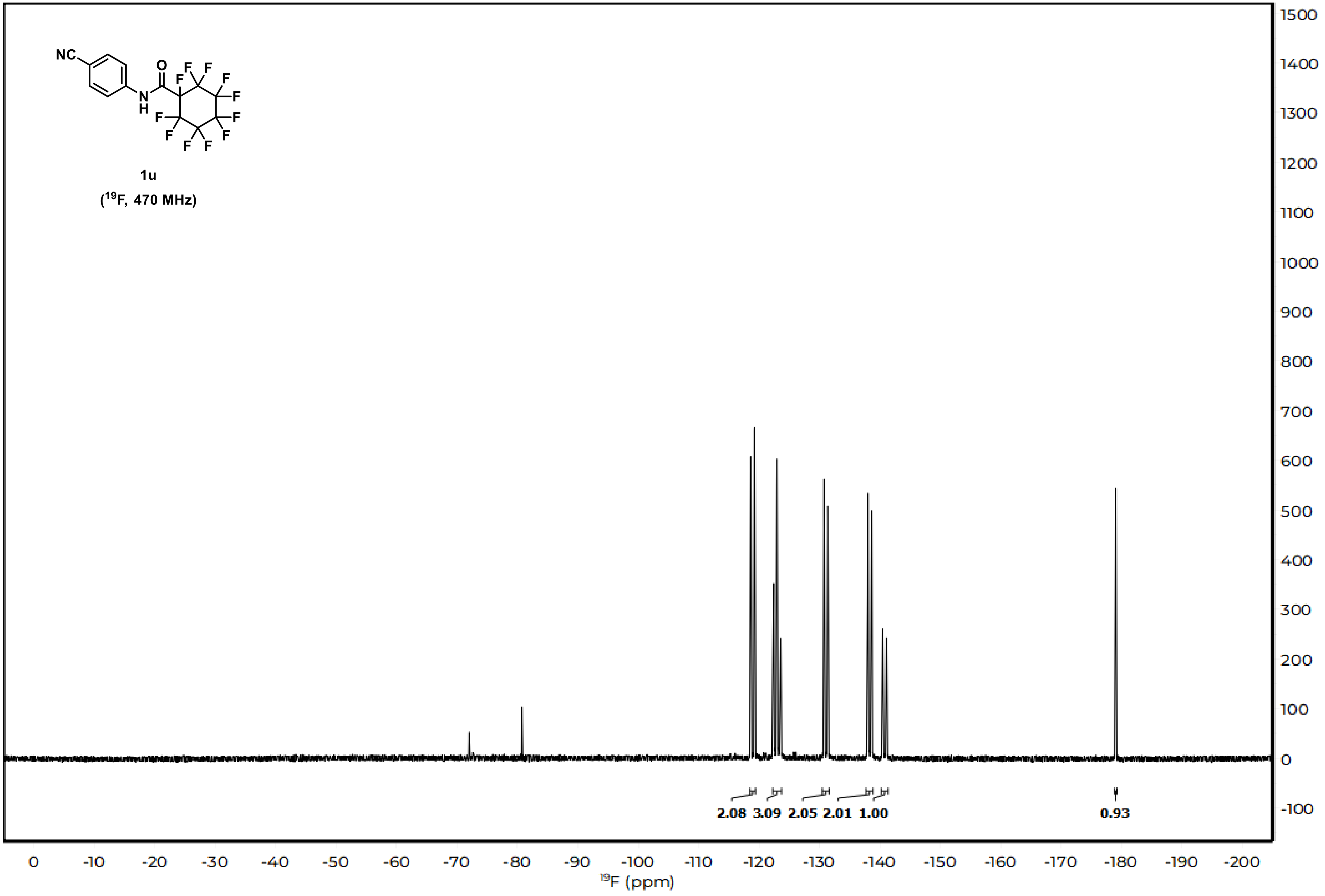
NC
O
N
H
F
1u
(^{19}F, 470 MHz)
2.08 3.09 2.05 2.01 1.00
0.93
^{19}F (ppm)

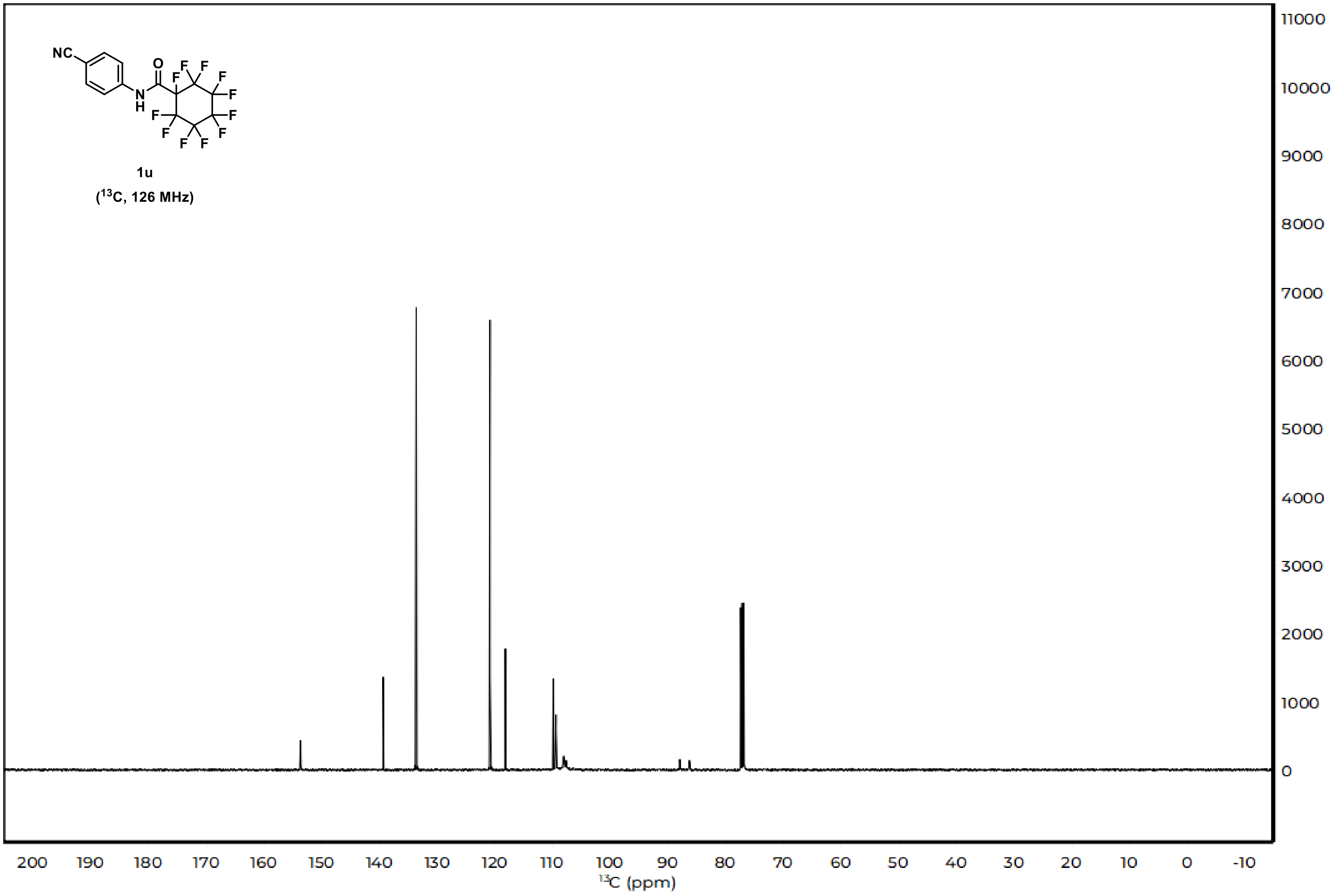
NC
O
N
H
F
1u
(13C, 126 MHz)
11000
10000
9000
8000
7000
6000
5000
4000
3000
2000
1000
0
200 190 180 170 160 150 140 130 120 110 100 90 80 70 60 50 40 30 20 10 0 -10
13C (ppm)

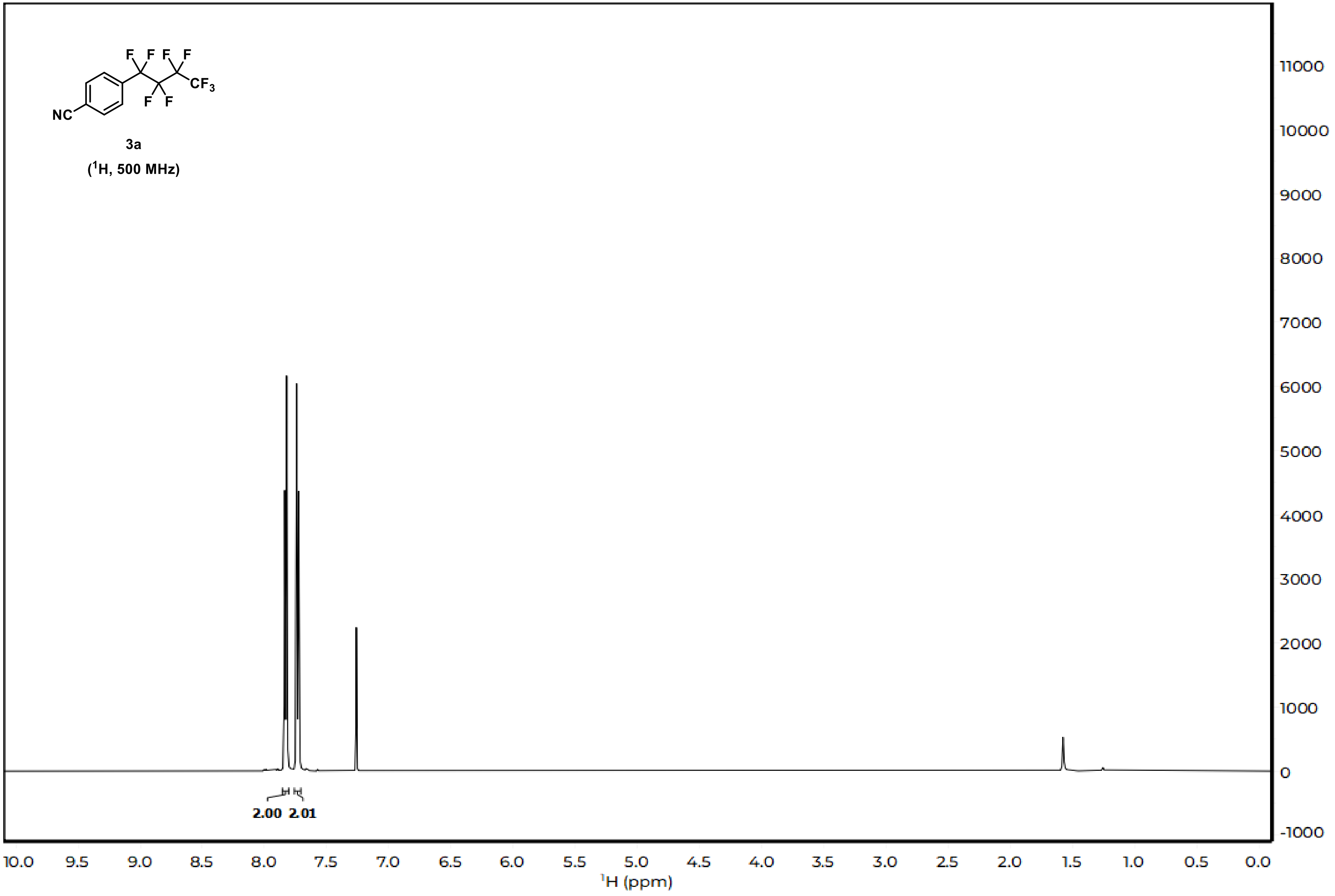

F F F F
CF3
F F
NC
3a
(1H, 500 MHz)
11000
10000
9000
8000
7000
6000
5000
4000
3000
2000
1000
0
-1000
2.00 2.01
10.0 9.5 9.0 8.5 8.0 7.5 7.0 6.5 6.0 5.5 5.0 4.5 4.0 3.5 3.0 2.5 2.0 1.5 1.0 0.5 0.0
1H (ppm)

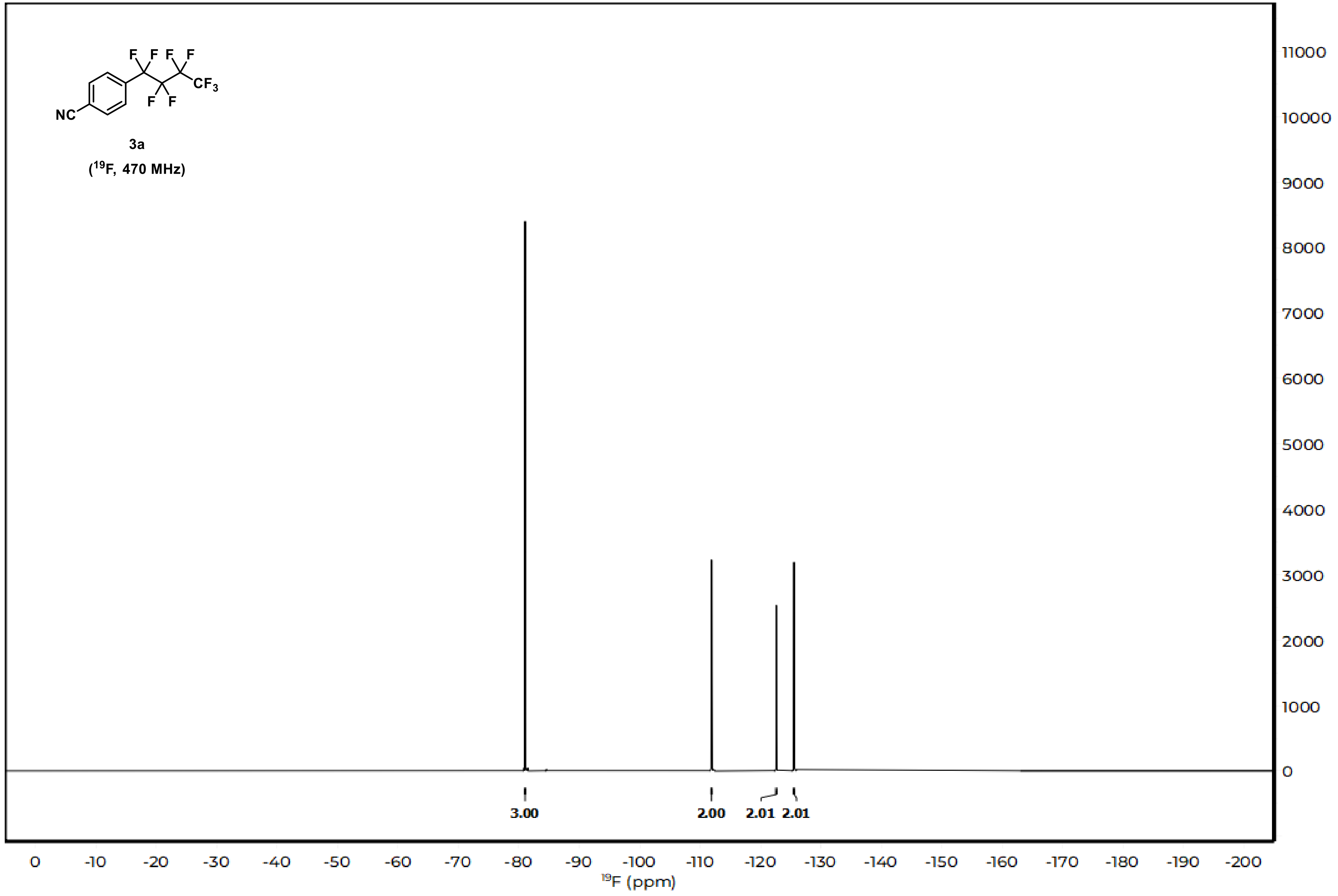

F F F F
CF3
NC
F F
3a
(19F, 470 MHz)
11000
10000
9000
8000
7000
6000
5000
4000
3000
2000
1000
0
3.00
2.00
2.01
2.01
0
-10
-20
-30
-40
-50
-60
-70
-80
-90
-100
-110
-120
-130
-140
-150
-160
-170
-180
-190
-200
19F (ppm)

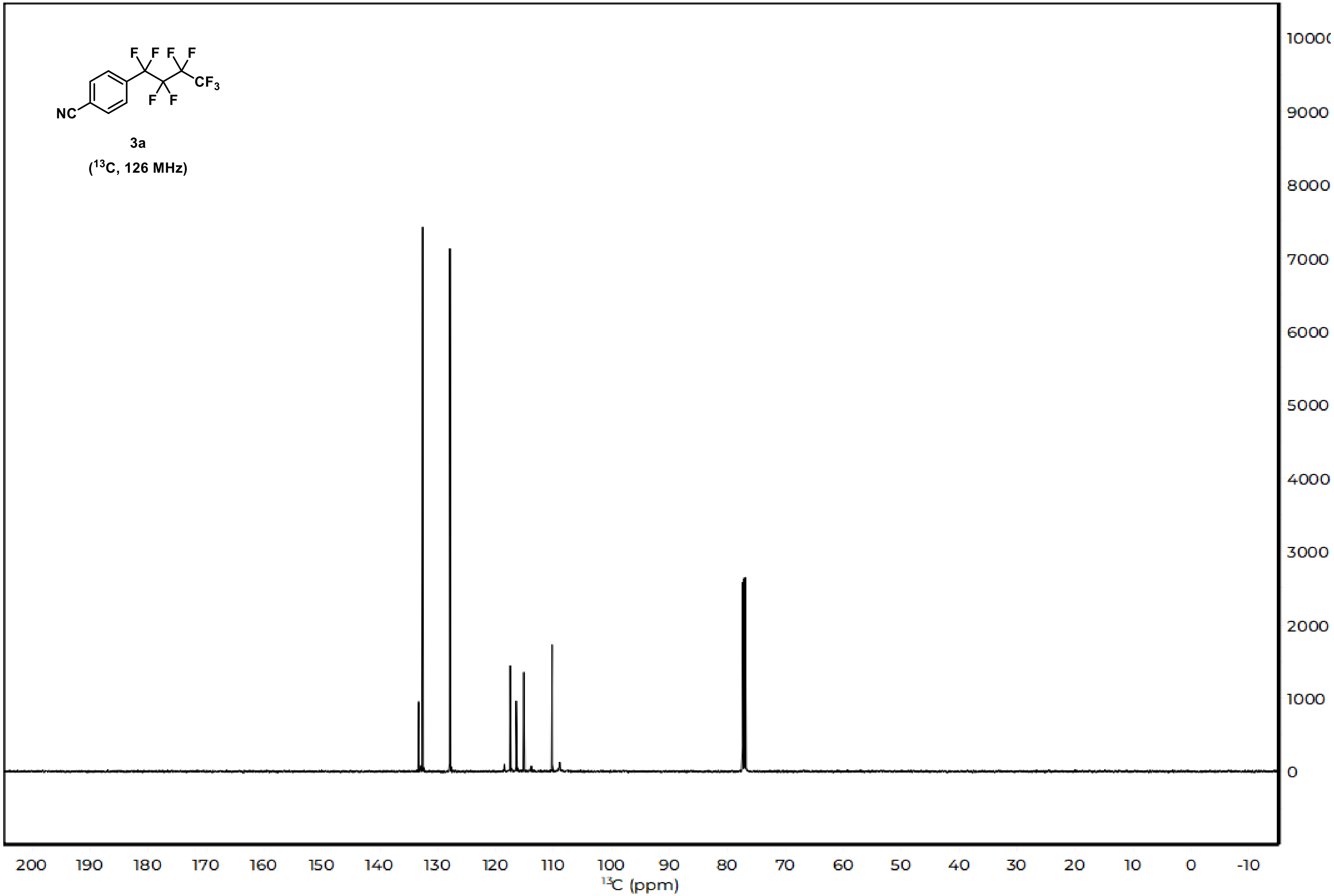

NC
F
CF3
3a
(13C, 126 MHz)
10000
9000
8000
7000
6000
5000
4000
3000
2000
1000
0
200
190
180
170
160
150
140
130
120
110
100
90
80
70
60
50
40
30
20
10
0
-10
13C (ppm)

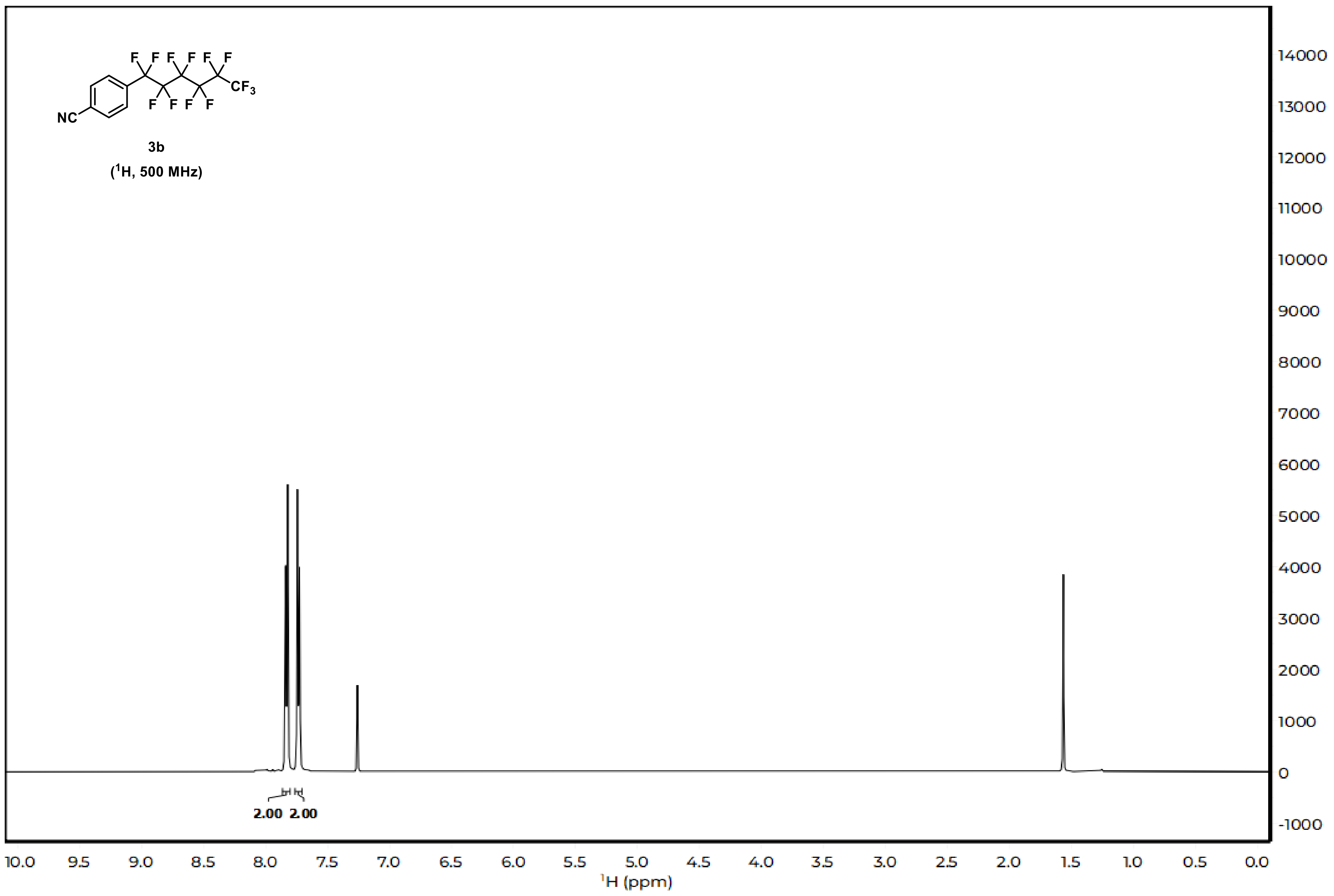

NC
CF3
3b
(1H, 500 MHz)
2.00 2.00
1H (ppm)

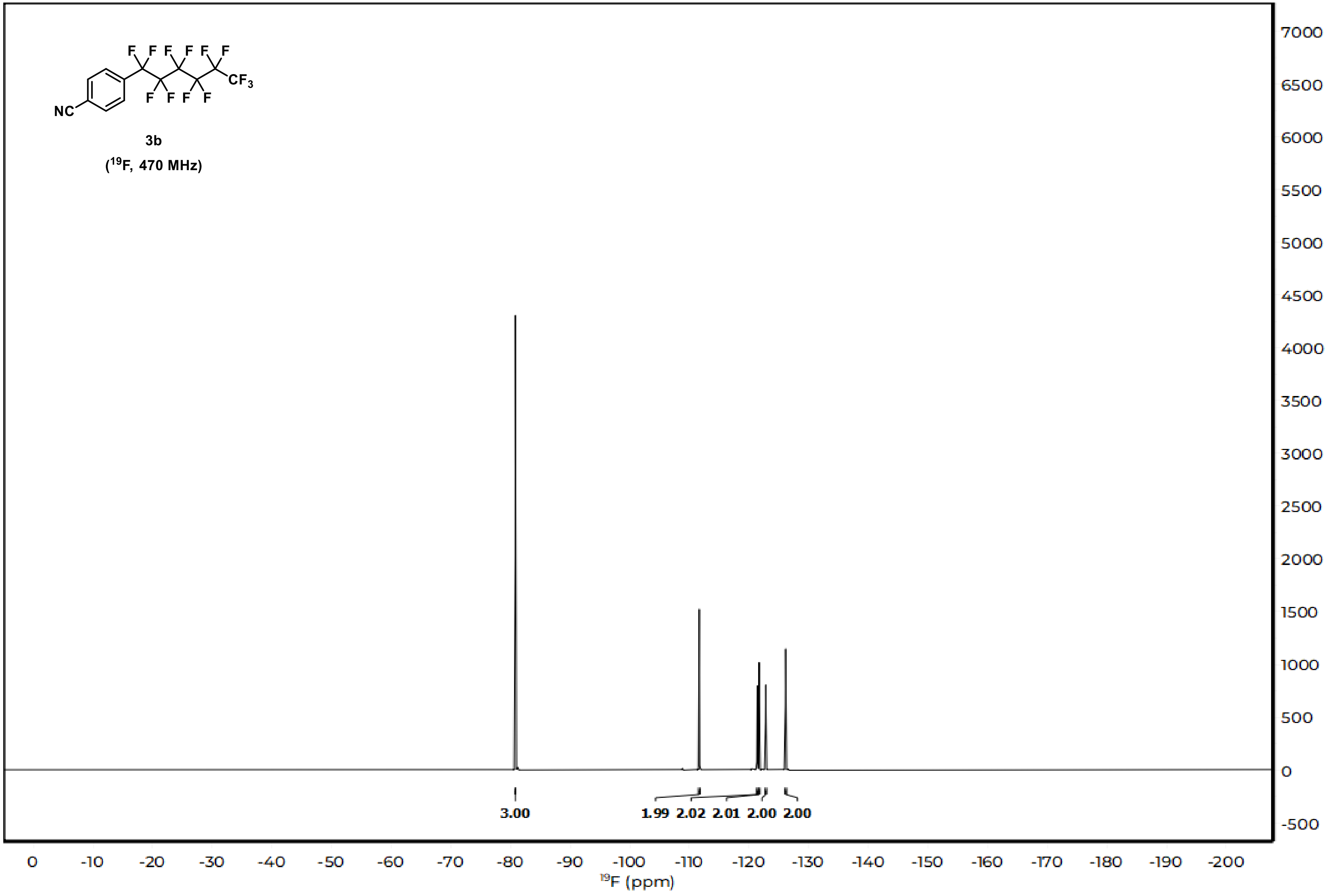
NC
CF3
3b
(19F, 470 MHz)
3.00
1.99 2.02 2.01 2.00 2.00
19F (ppm)

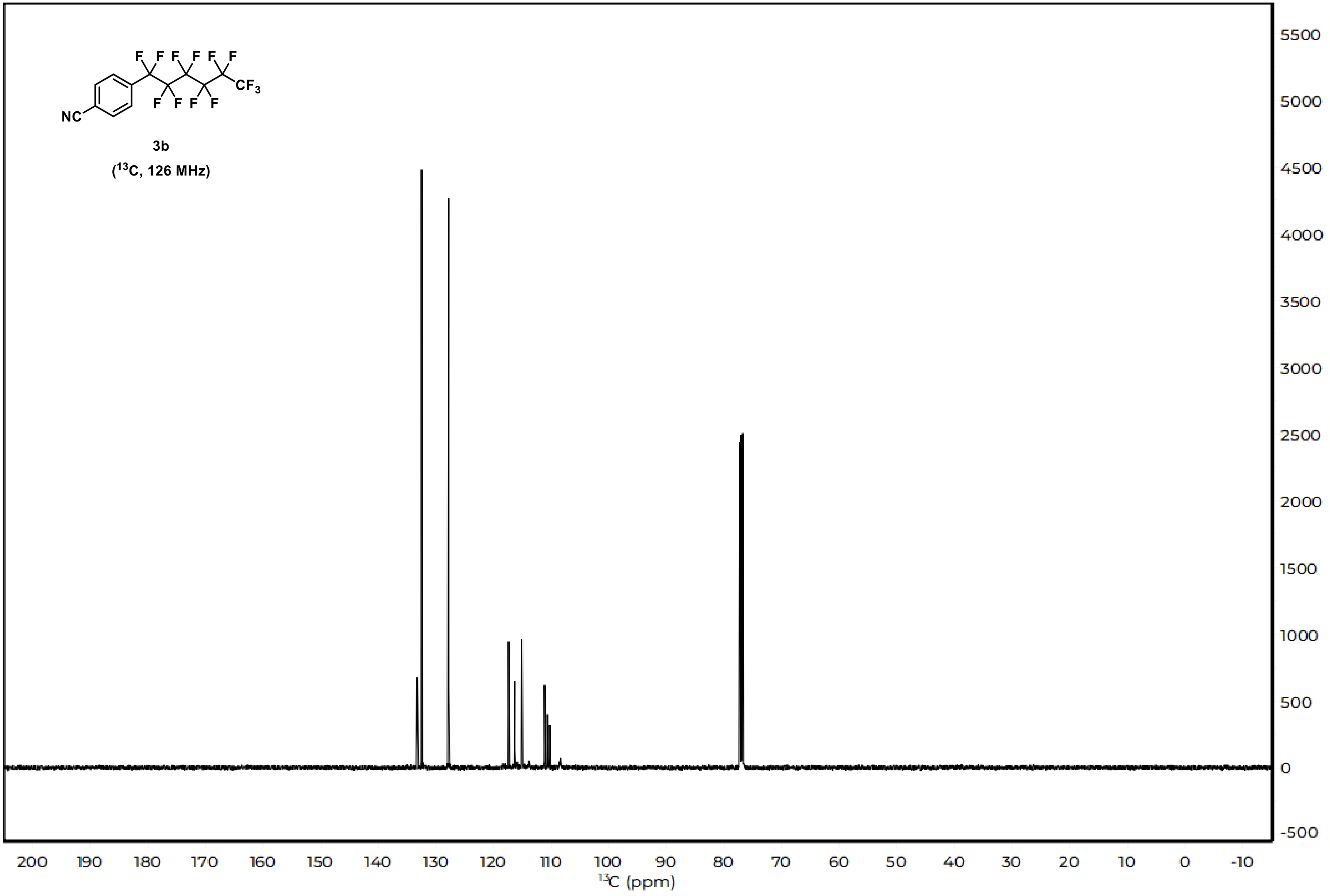
NC
F F F F F F
F F F F
CF3
3b
(13C, 126 MHz)
5500
5000
4500
4000
3500
3000
2500
2000
1500
1000
500
0
-500
200 190 180 170 160 150 140 130 120 110 100 90 80 70 60 50 40 30 20 10 0 -10
13C (ppm)

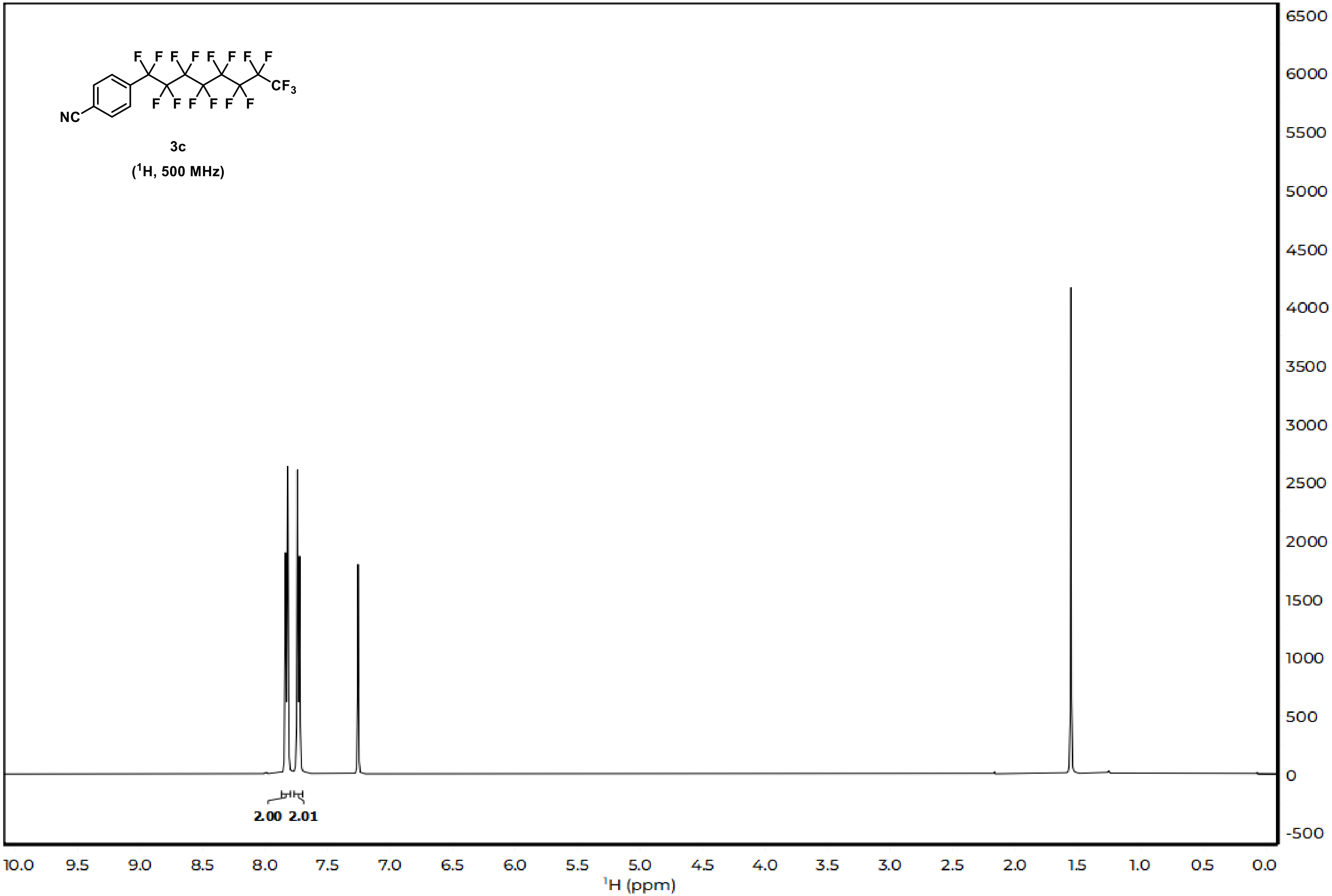

NC
CF3
3c
(1H, 500 MHz)
2.00 2.01
1H (ppm)

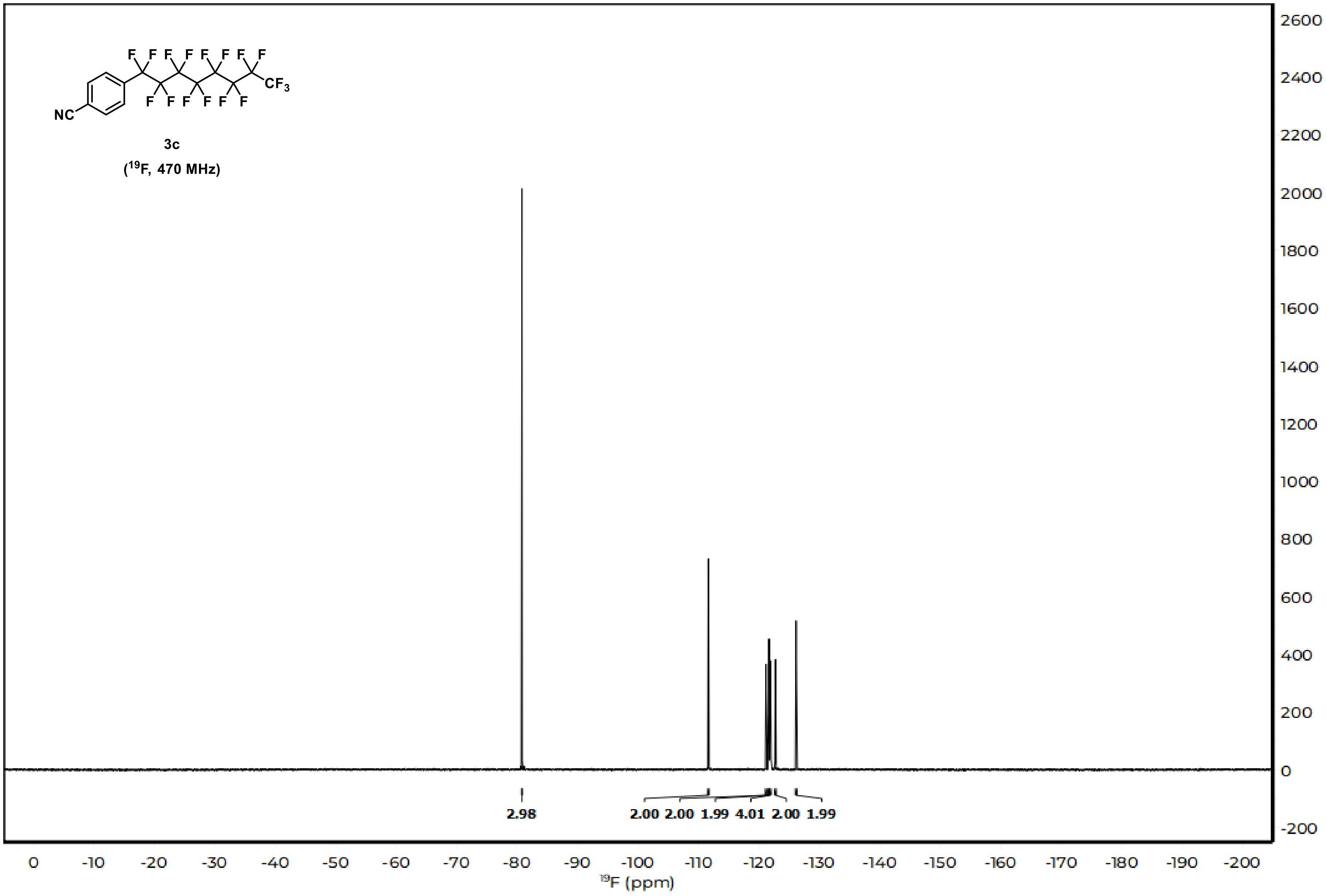

NC
CF3
3c
(19F, 470 MHz)
2.98
2.00 2.00 1.99 4.01 2.00 1.99
19F (ppm)

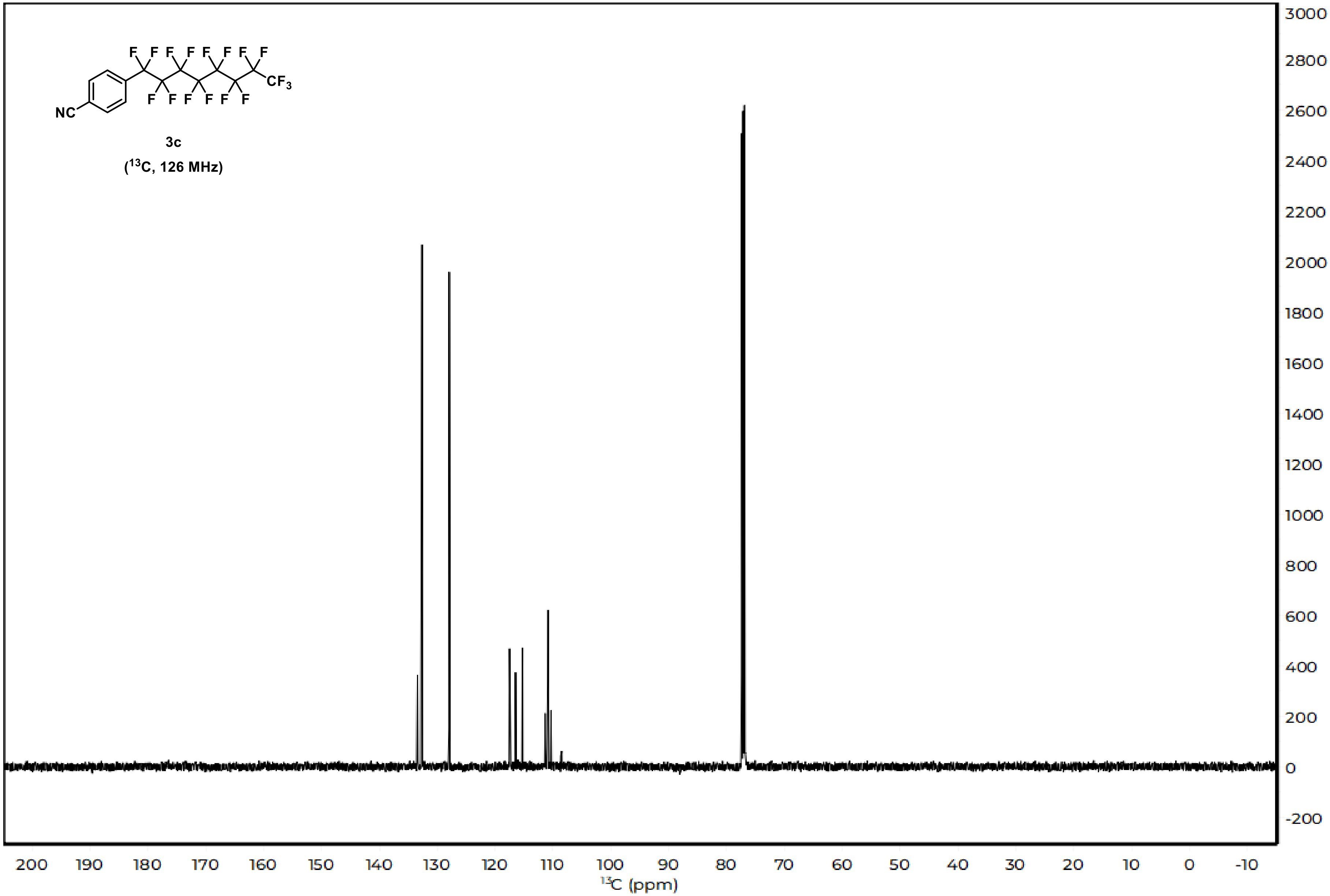

NC
CF3
3c
(13C, 126 MHz)
3000
2800
2600
2400
2200
2000
1800
1600
1400
1200
1000
800
600
400
200
0
-200
200 190 180 170 160 150 140 130 120 110 100 90 80 70 60 50 40 30 20 10 0 -10
13C (ppm)

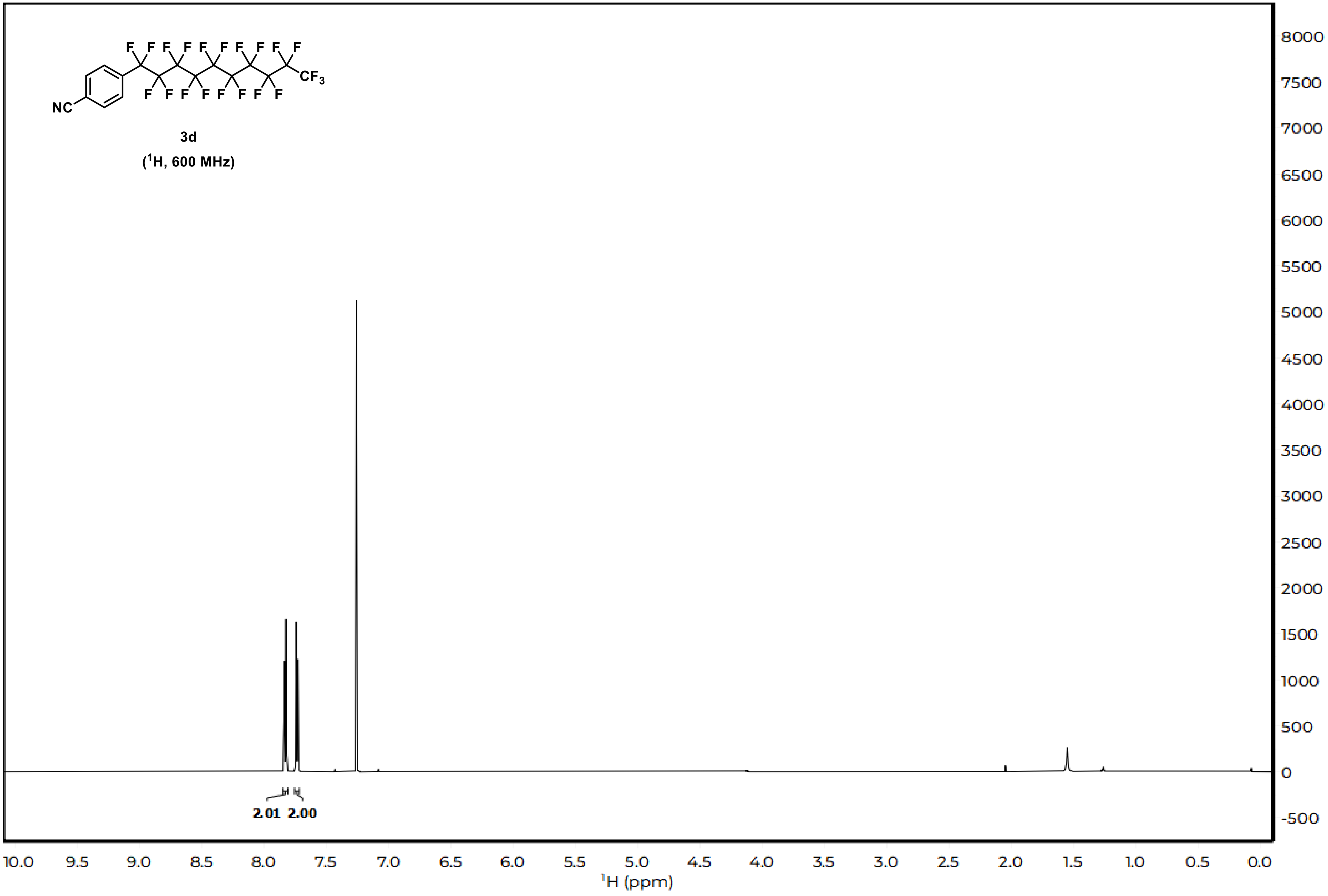

NC
CF3
3d
(1H, 600 MHz)
2.01 2.00
1H (ppm)

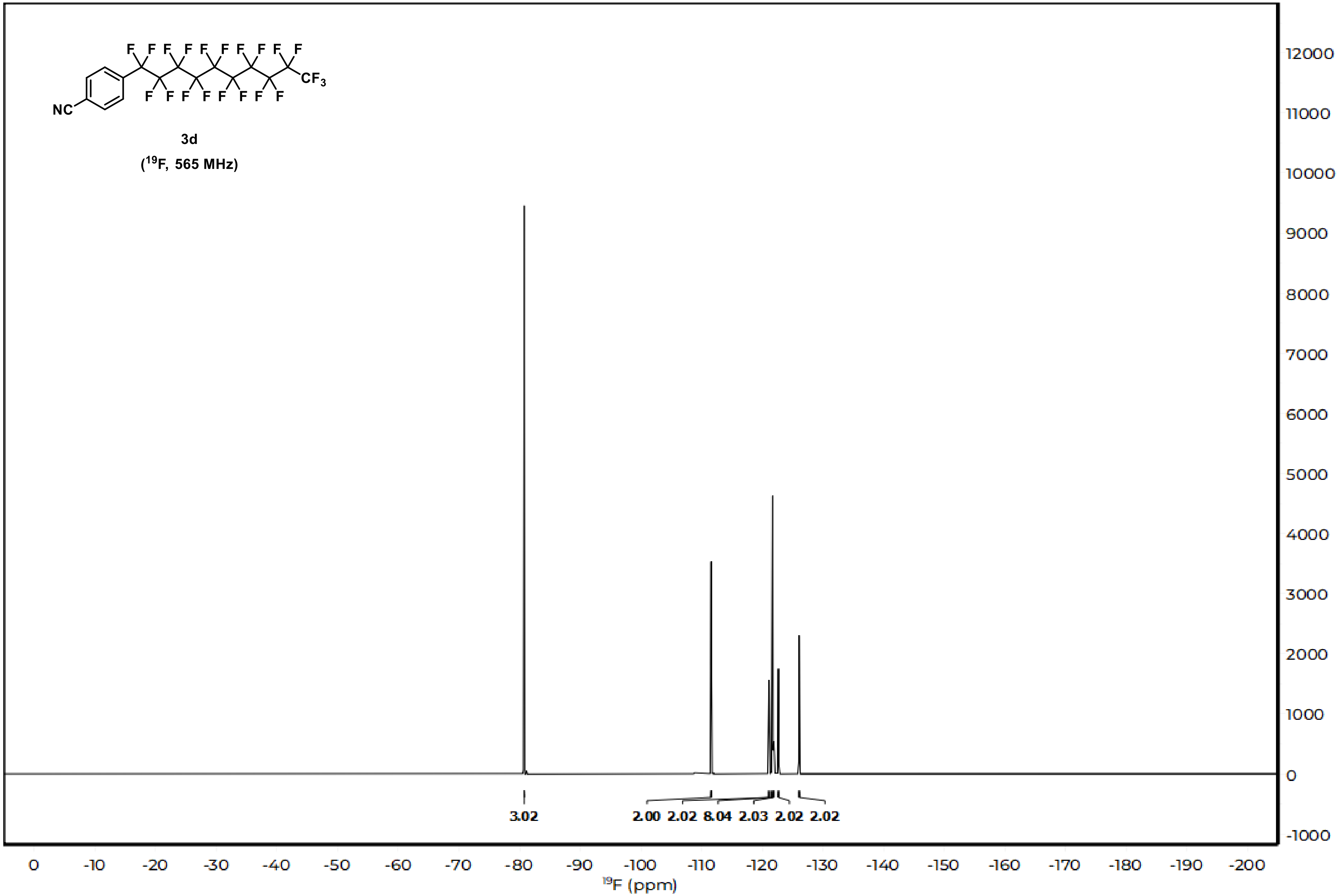

NC
CF3
3d
(19F, 565 MHz)
3.02
2.00 2.02 8.04 2.03 2.02 2.02
19F (ppm)

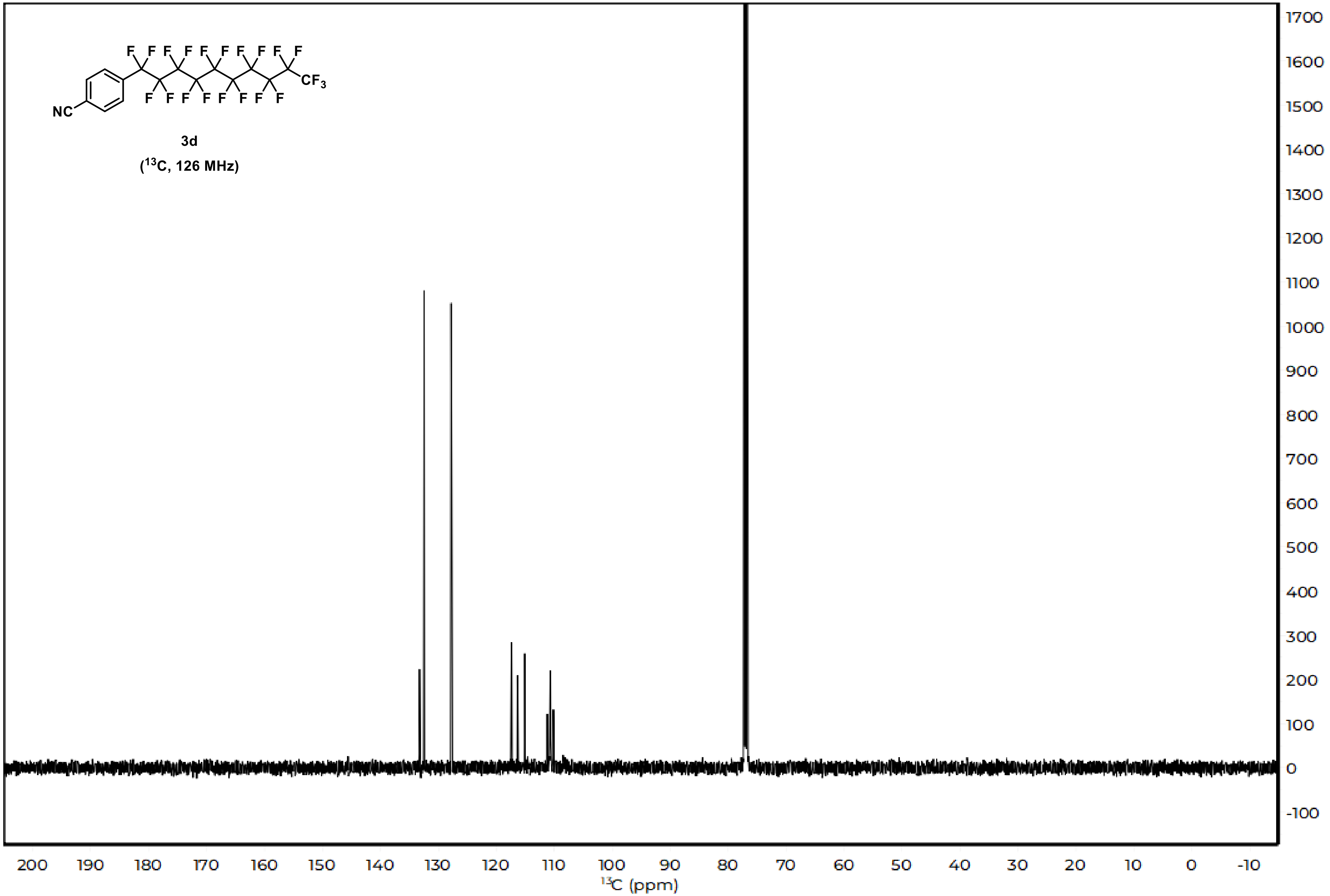

NC
CF3
3d
(13C, 126 MHz)
1700
1600
1500
1400
1300
1200
1100
1000
900
800
700
600
500
400
300
200
100
0
-100
200
190
180
170
160
150
140
130
120
110
100
90
80
70
60
50
40
30
20
10
0
-10
13C (ppm)

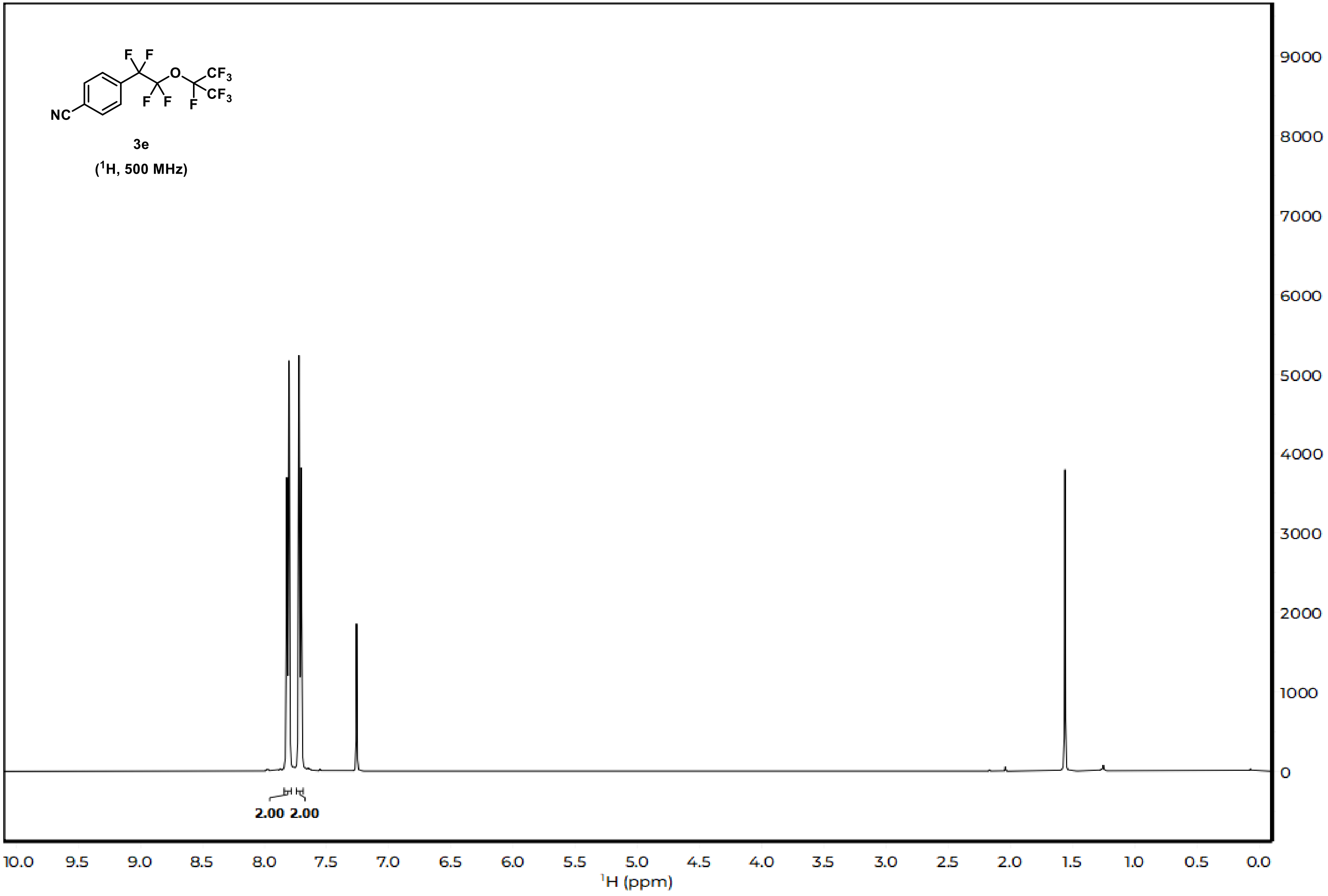
NC
F F
O
CF3
CF3
F F
F
3e
(1H, 500 MHz)
9000
8000
7000
6000
5000
4000
3000
2000
1000
0
2.00 2.00
10.0 9.5 9.0 8.5 8.0 7.5 7.0 6.5 6.0 5.5 5.0 4.5 4.0 3.5 3.0 2.5 2.0 1.5 1.0 0.5 0.0
1H (ppm)

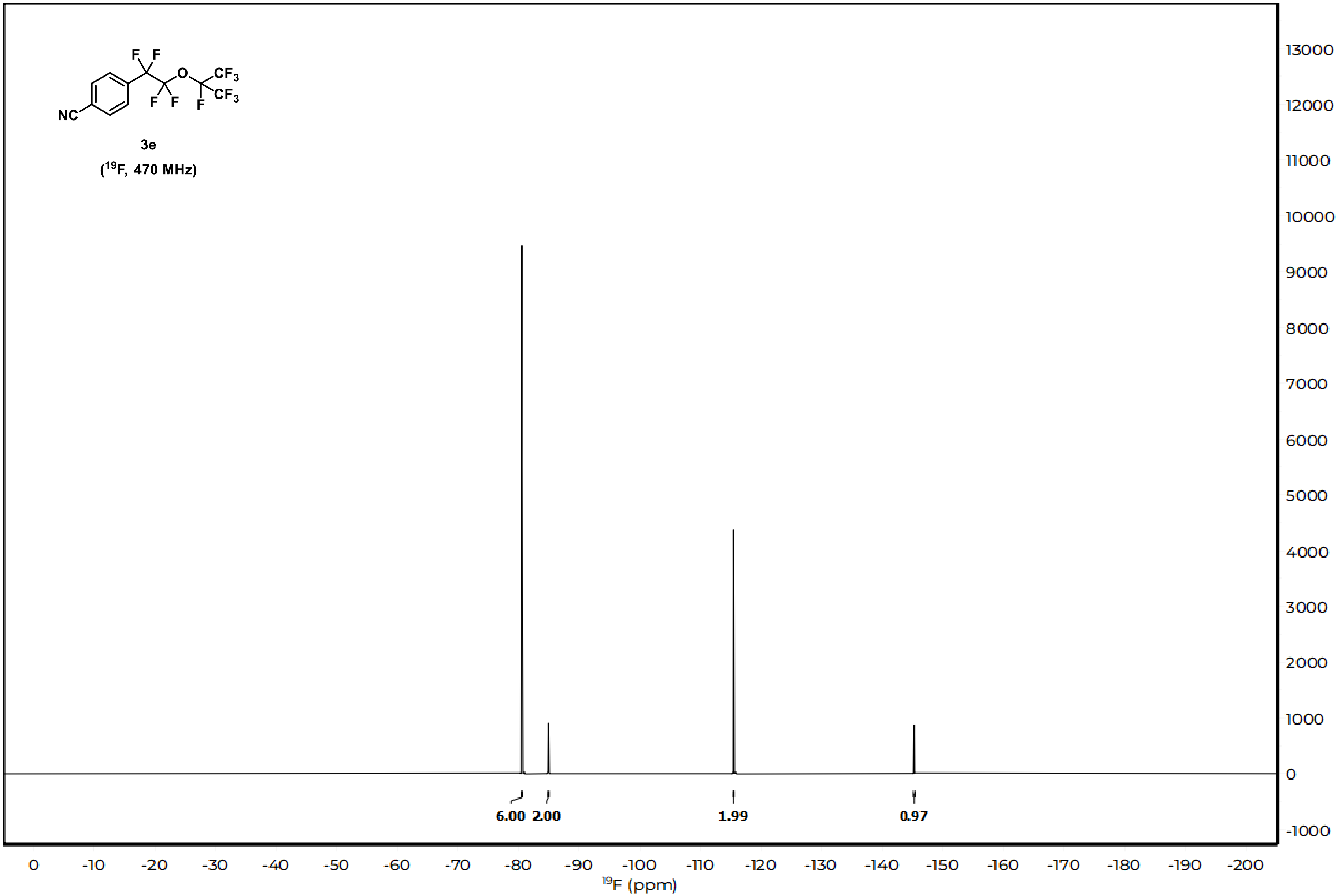
NC
F F
O
CF3
CF3
F F F
3e
(19F, 470 MHz)
13000
12000
11000
10000
9000
8000
7000
6000
5000
4000
3000
2000
1000
0
-1000
6.00 2.00
1.99
0.97
0
-10
-20
-30
-40
-50
-60
-70
-80
-90
-100
-110
-120
-130
-140
-150
-160
-170
-180
-190
-200
19F (ppm)

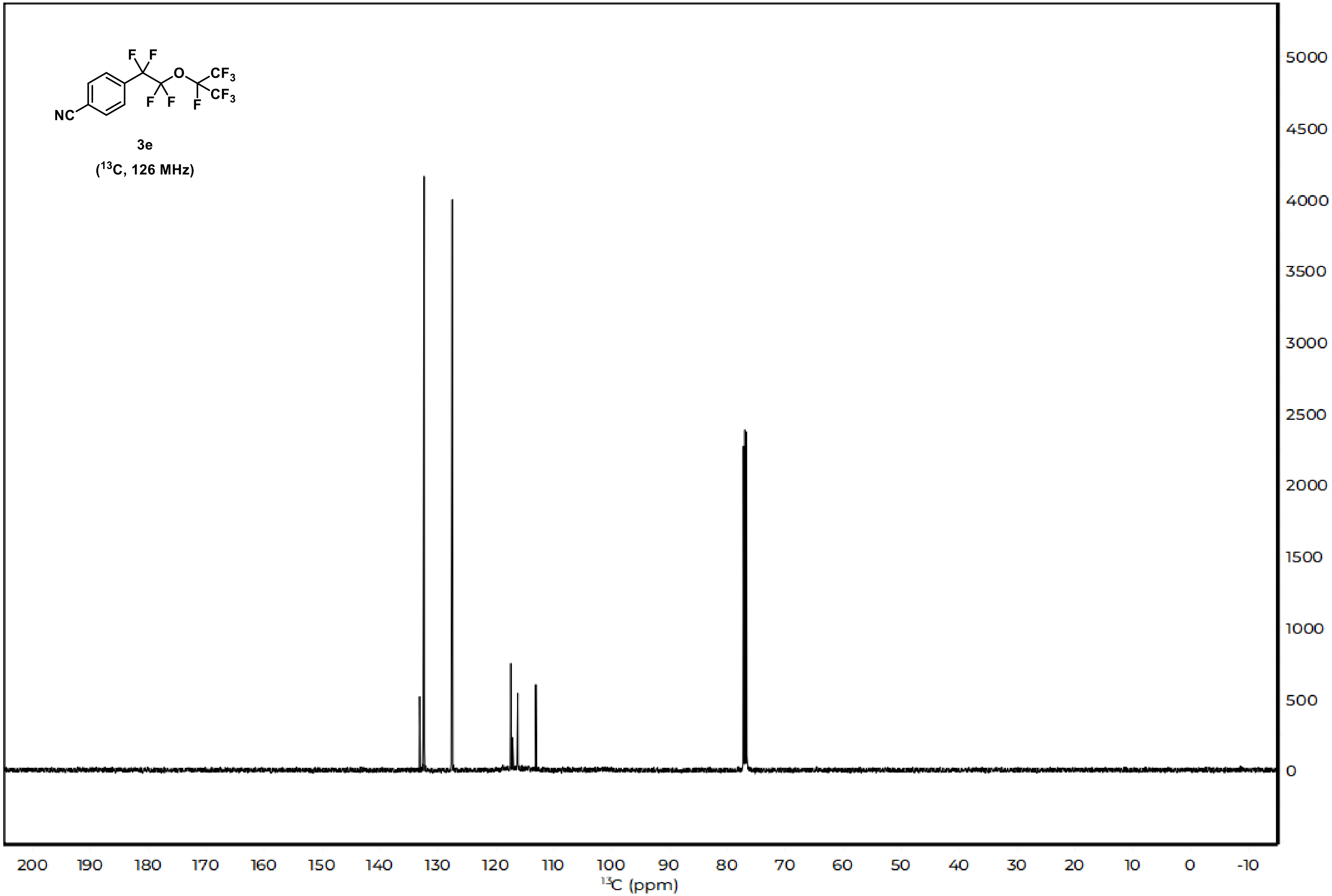

NC
F
F
O
CF_3
CF_3
3e
(^{13}C, 126 MHz)
5000
4500
4000
3500
3000
2500
2000
1500
1000
500
0
200
190
180
170
160
150
140
130
120
110
100
90
80
70
60
50
40
30
20
10
0
-10
^{13}C (ppm)

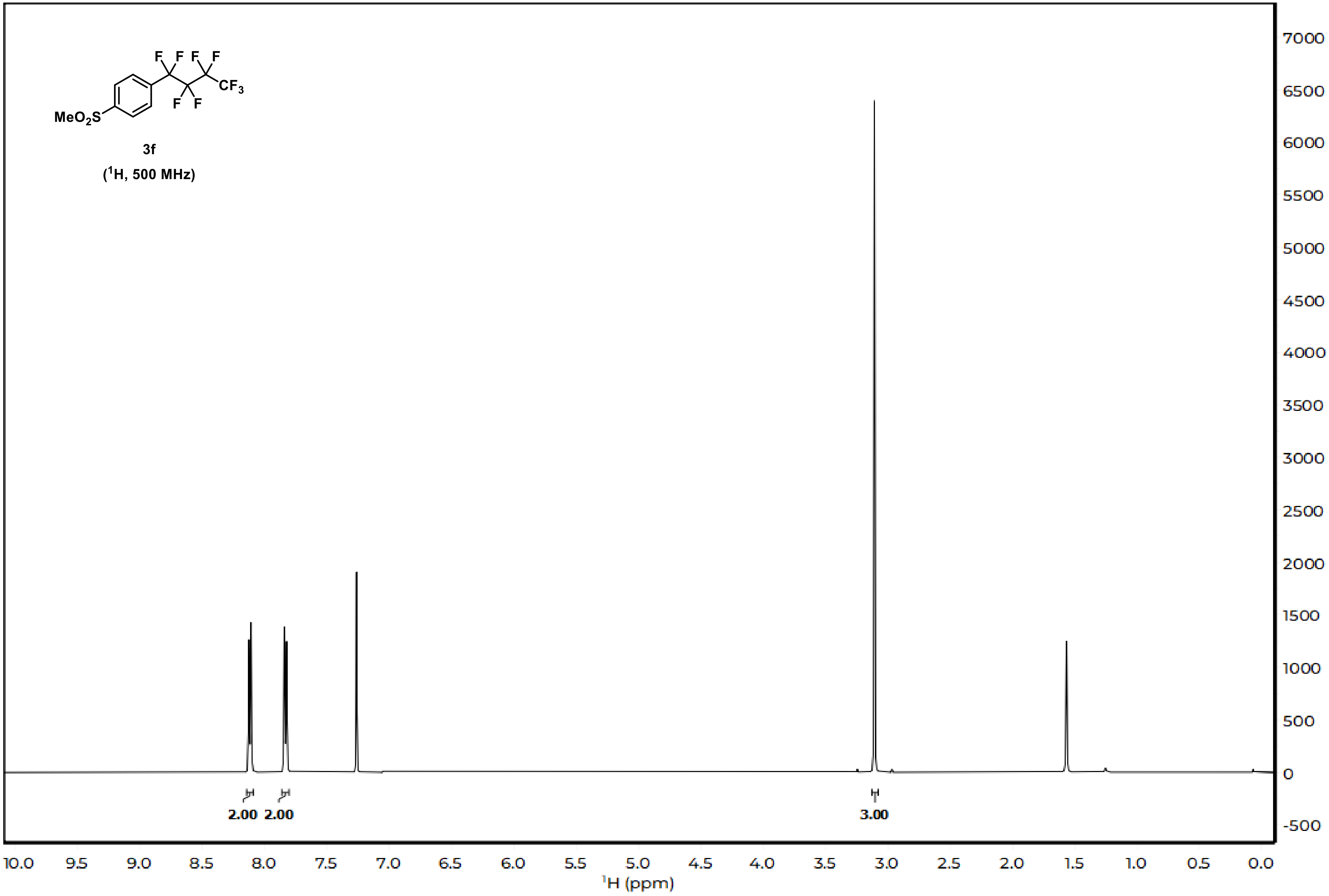
MeO2S
F F F F
CF3
F F
3f
(1H, 500 MHz)
7000
6500
6000
5500
5000
4500
4000
3500
3000
2500
2000
1500
1000
500
0
-500
2.00
2.00
3.00
10.0
9.5
9.0
8.5
8.0
7.5
7.0
6.5
6.0
5.5
5.0
4.5
4.0
3.5
3.0
2.5
2.0
1.5
1.0
0.5
0.0
1H (ppm)

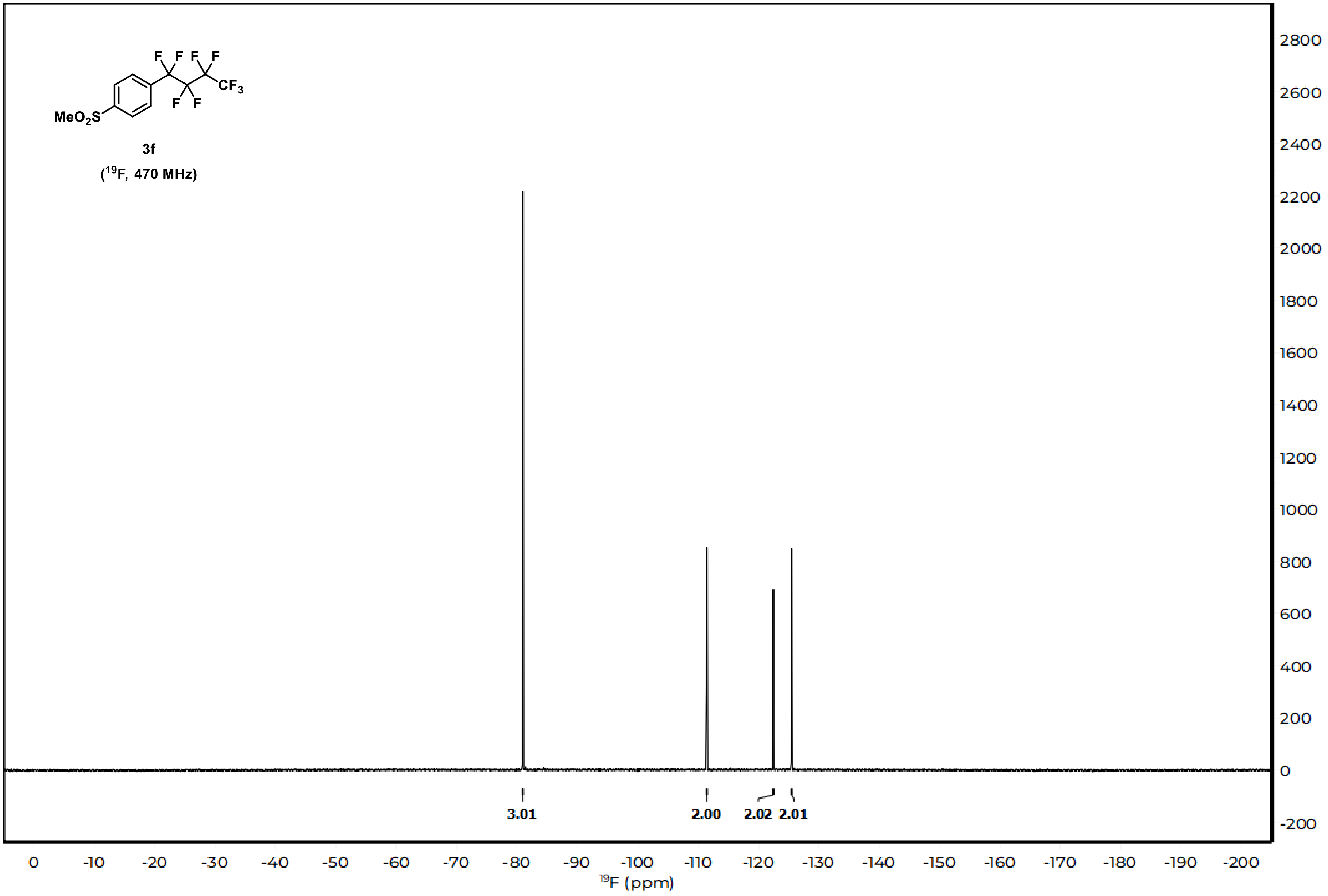

MeO2S
3f
(19F, 470 MHz)
3.01
2.00
2.02
2.01
19F (ppm)

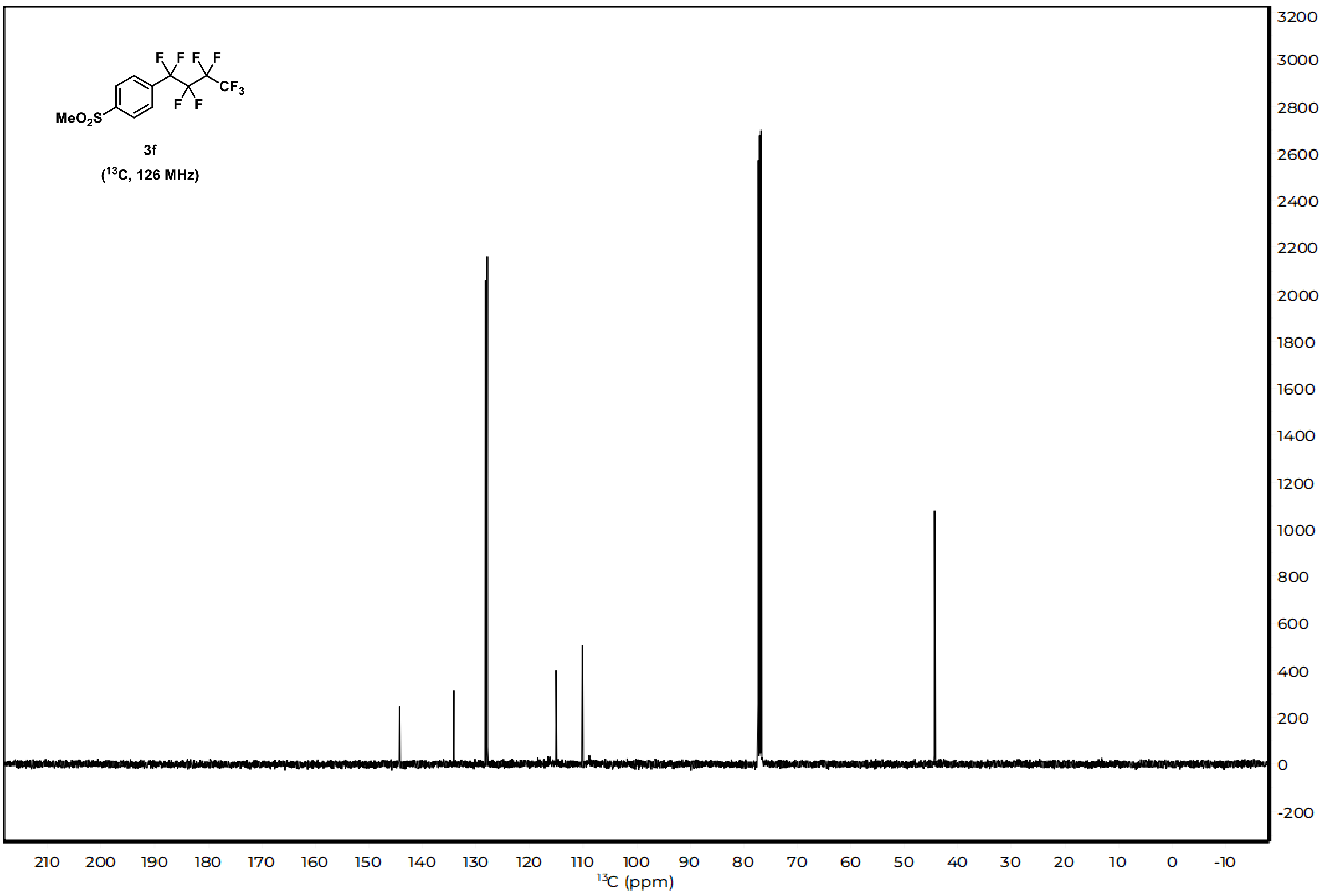
MeO2S
F F F F
CF3
F F
3f
(13C, 126 MHz)
3200
3000
2800
2600
2400
2200
2000
1800
1600
1400
1200
1000
800
600
400
200
0
-200
210 200 190 180 170 160 150 140 130 120 110 100 90 80 70 60 50 40 30 20 10 0 -10
13C (ppm)

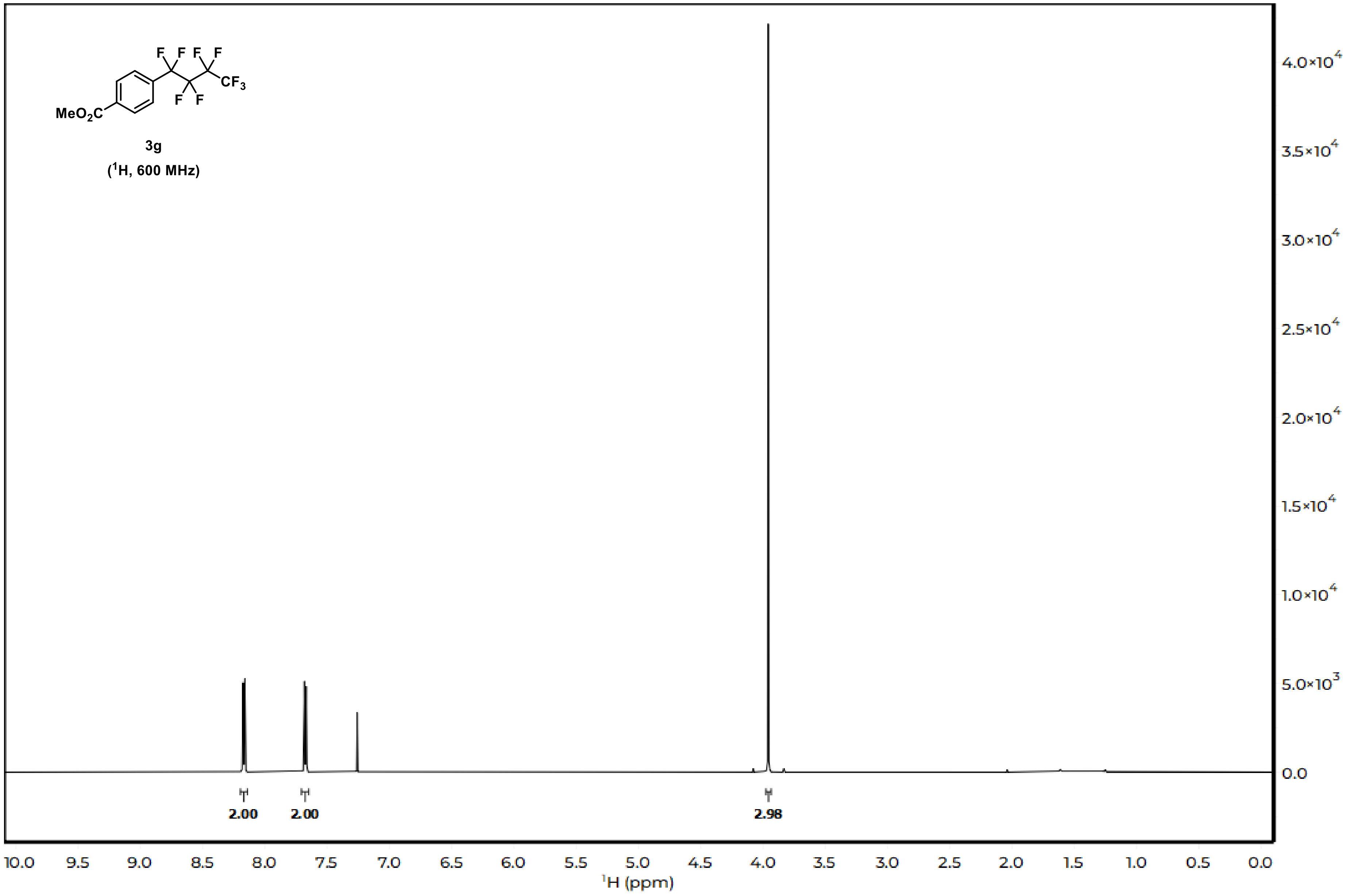
MeO2C
F F F F
CF3
F F
3g
(1H, 600 MHz)
4.0×10^4
3.5×10^4
3.0×10^4
2.5×10^4
2.0×10^4
1.5×10^4
1.0×10^4
5.0×10^3
0.0
2.00
2.00
2.98
10.0 9.5 9.0 8.5 8.0 7.5 7.0 6.5 6.0 5.5 5.0 4.5 4.0 3.5 3.0 2.5 2.0 1.5 1.0 0.5 0.0
1H (ppm)

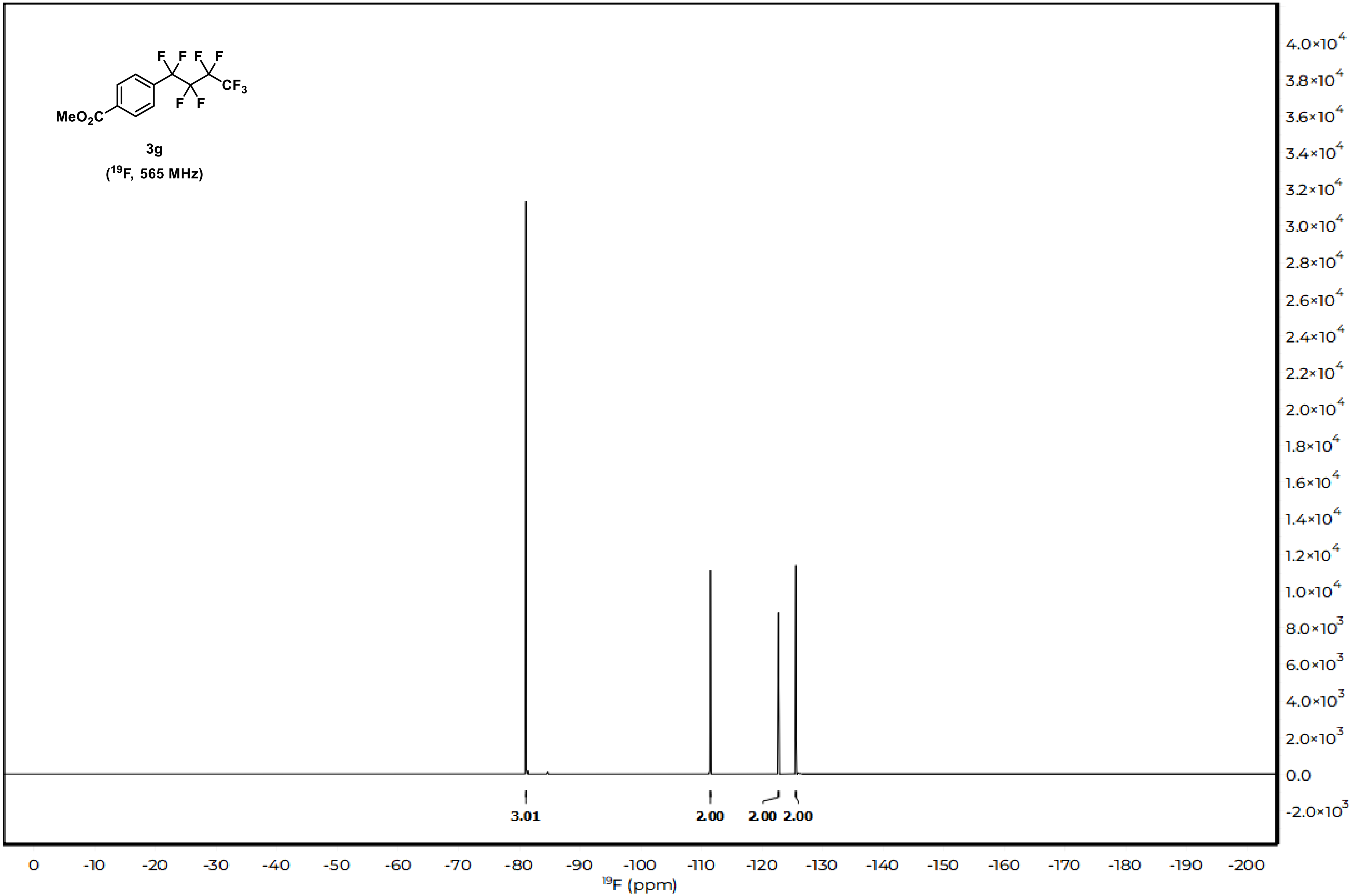
F F F F
CF3
F F
MeO2C
3g
(19F, 565 MHz)
3.01
2.00
2.00
2.00
19F (ppm)

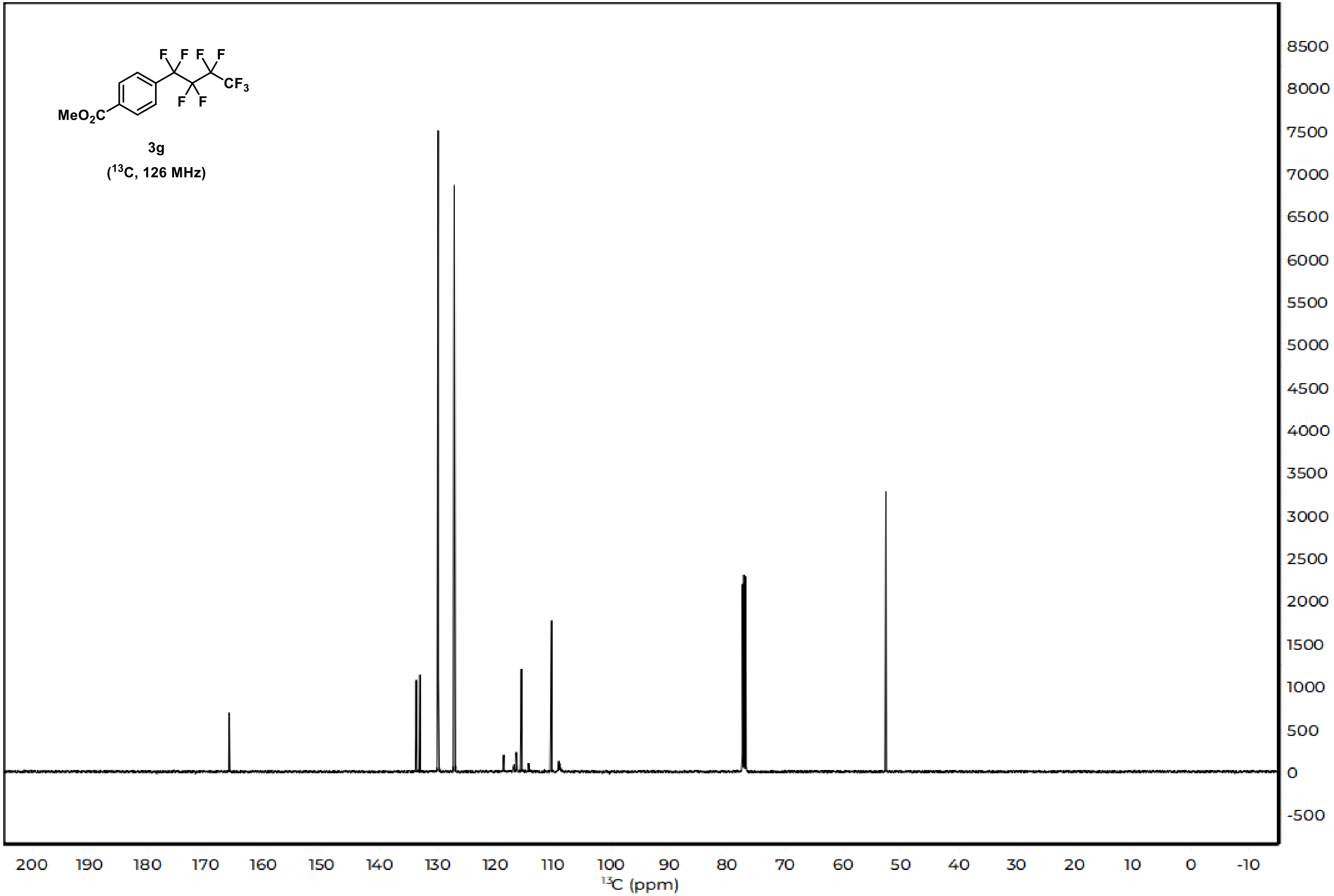

F F F F
CF3
F F
MeO2C
3g
(13C, 126 MHz)
8500
8000
7500
7000
6500
6000
5500
5000
4500
4000
3500
3000
2500
2000
1500
1000
500
0
-500
200 190 180 170 160 150 140 130 120 110 100 90 80 70 60 50 40 30 20 10 0 -10
13C (ppm)

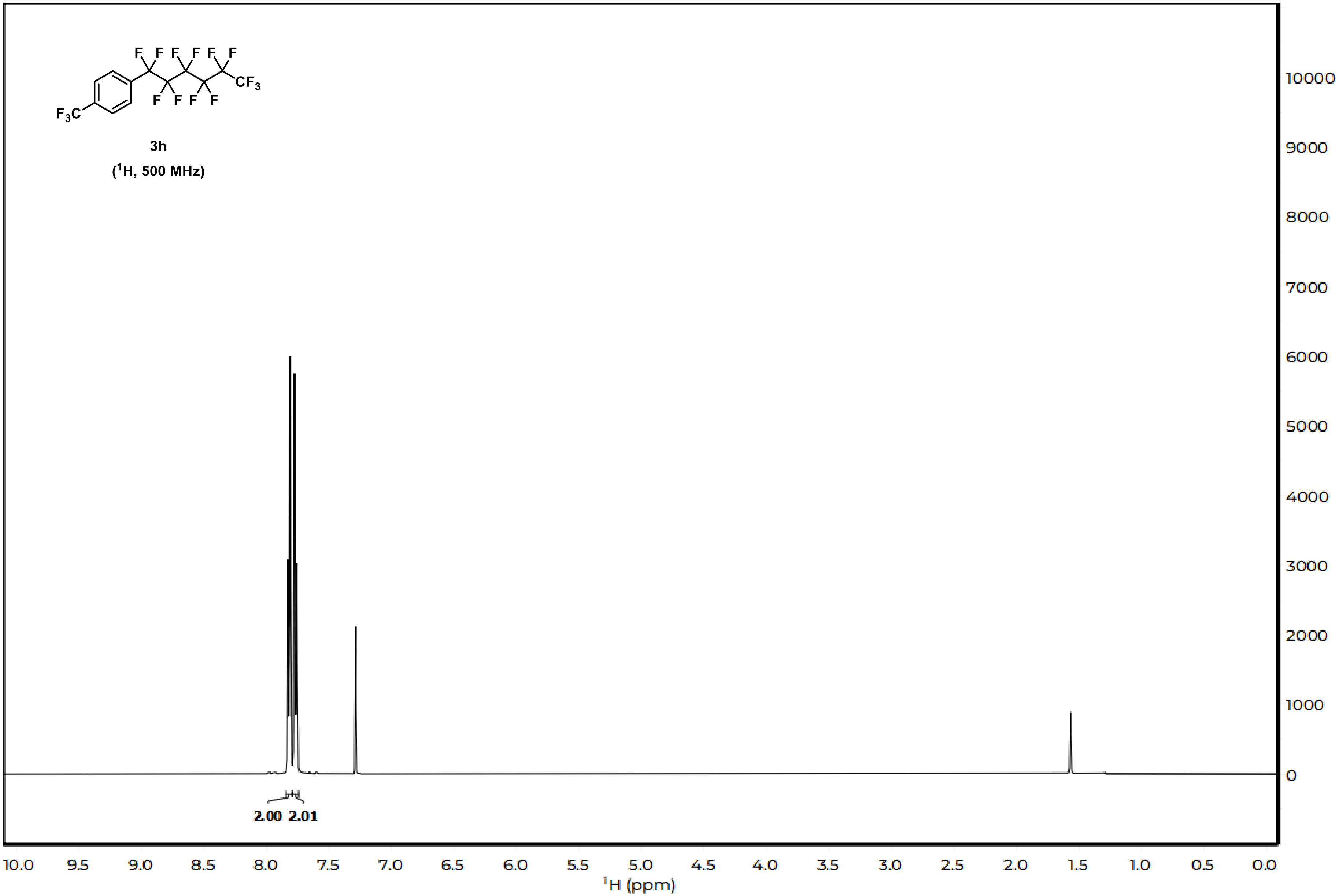

F F F F F F
CF3
F3C
F F F F
3h
(1H, 500 MHz)
10000
9000
8000
7000
6000
5000
4000
3000
2000
1000
0
2.00 2.01
10.0 9.5 9.0 8.5 8.0 7.5 7.0 6.5 6.0 5.5 5.0 4.5 4.0 3.5 3.0 2.5 2.0 1.5 1.0 0.5 0.0
1H (ppm)

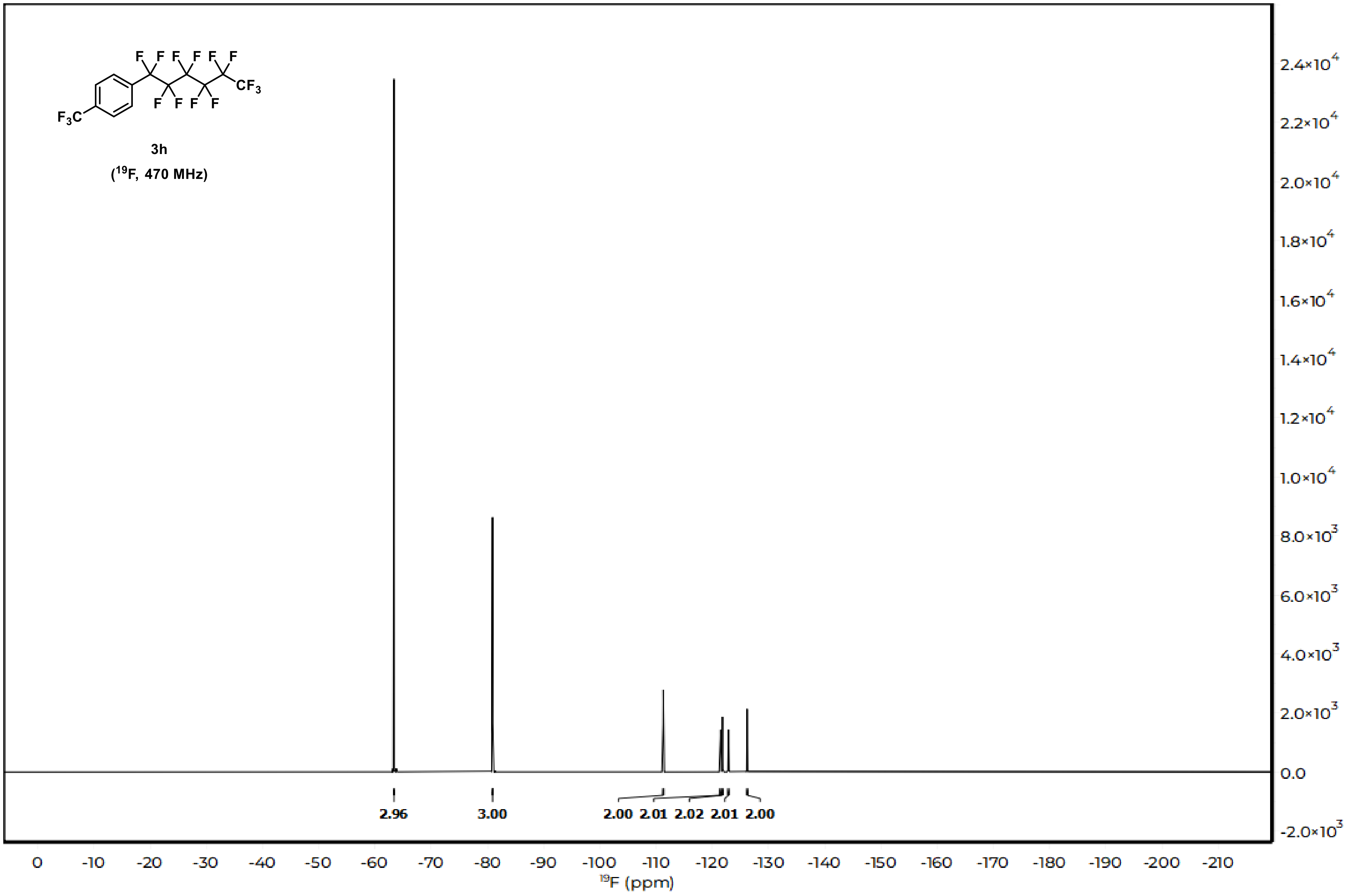
3h
(^{19}F, 470 MHz)
2.96
3.00
2.00 2.01 2.02 2.01 2.00
^{19}F (ppm)

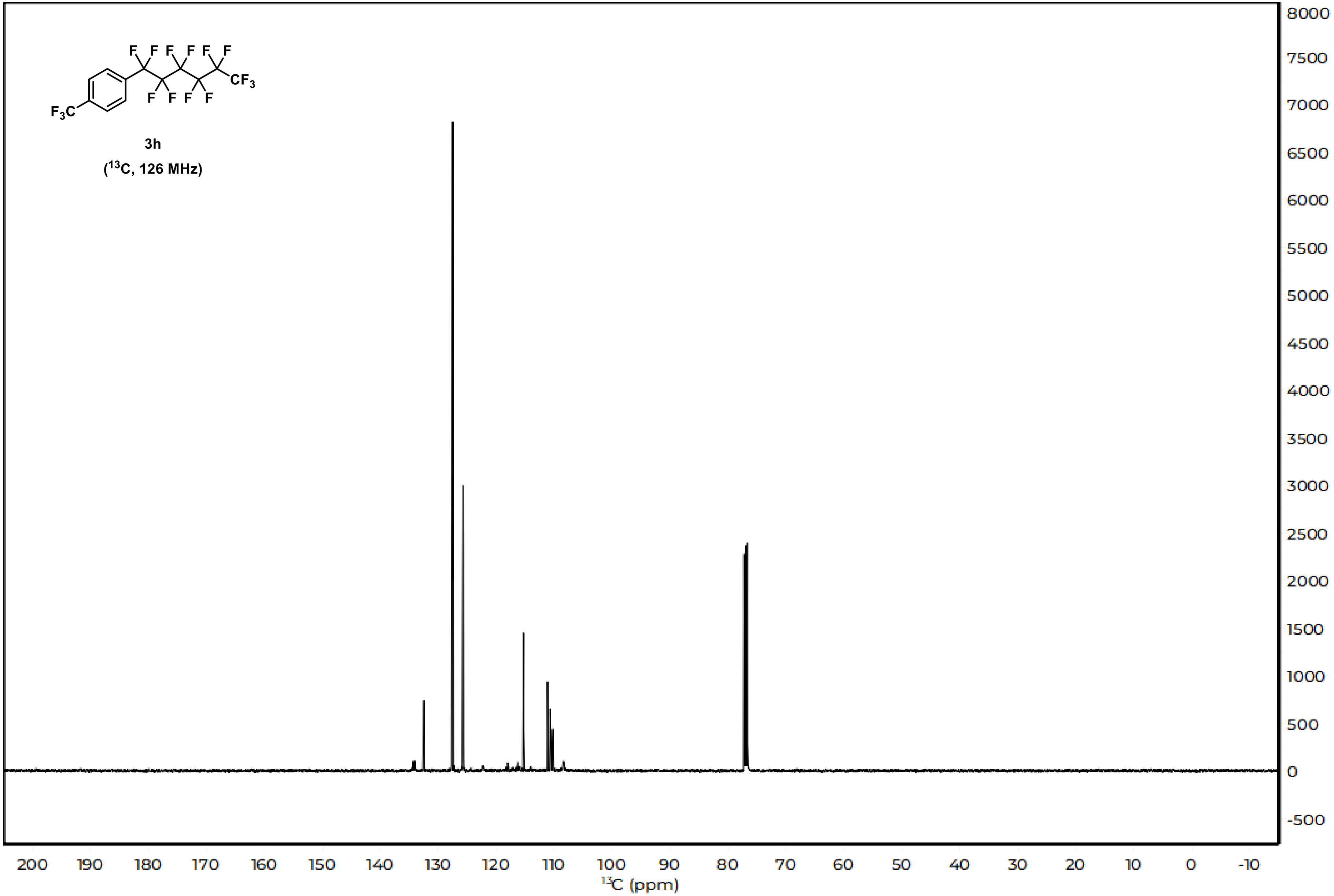
3h
(13C, 126 MHz)
8000
7500
7000
6500
6000
5500
5000
4500
4000
3500
3000
2500
2000
1500
1000
500
0
-500
200
190
180
170
160
150
140
130
120
110
100
90
80
70
60
50
40
30
20
10
0
-10
13C (ppm)

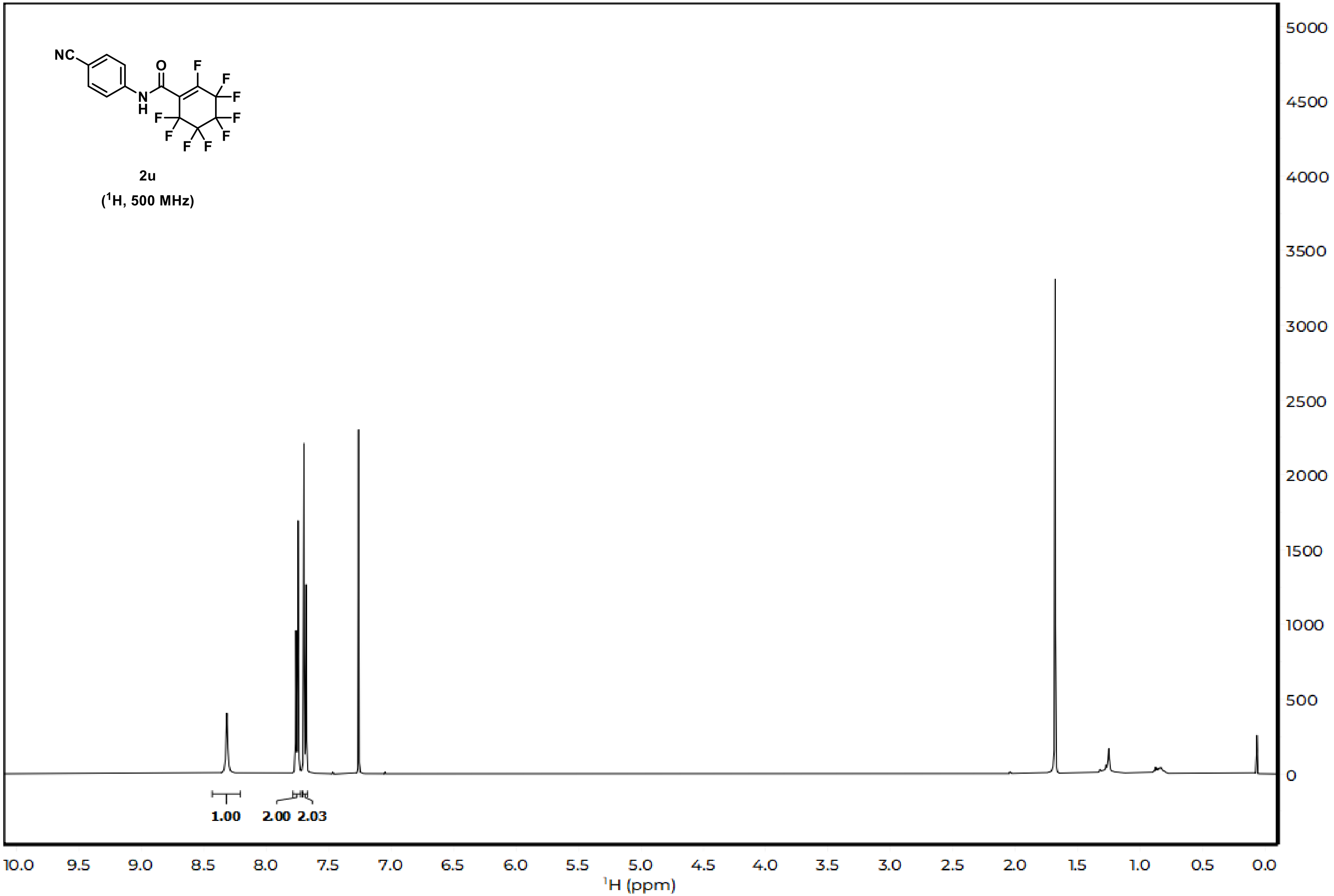
NC
O
F
N
H
F
2u
(¹H, 500 MHz)
5000
4500
4000
3500
3000
2500
2000
1500
1000
500
0
1.00
2.00
2.03
10.0
9.5
9.0
8.5
8.0
7.5
7.0
6.5
6.0
5.5
5.0
4.5
4.0
3.5
3.0
2.5
2.0
1.5
1.0
0.5
0.0
¹H (ppm)

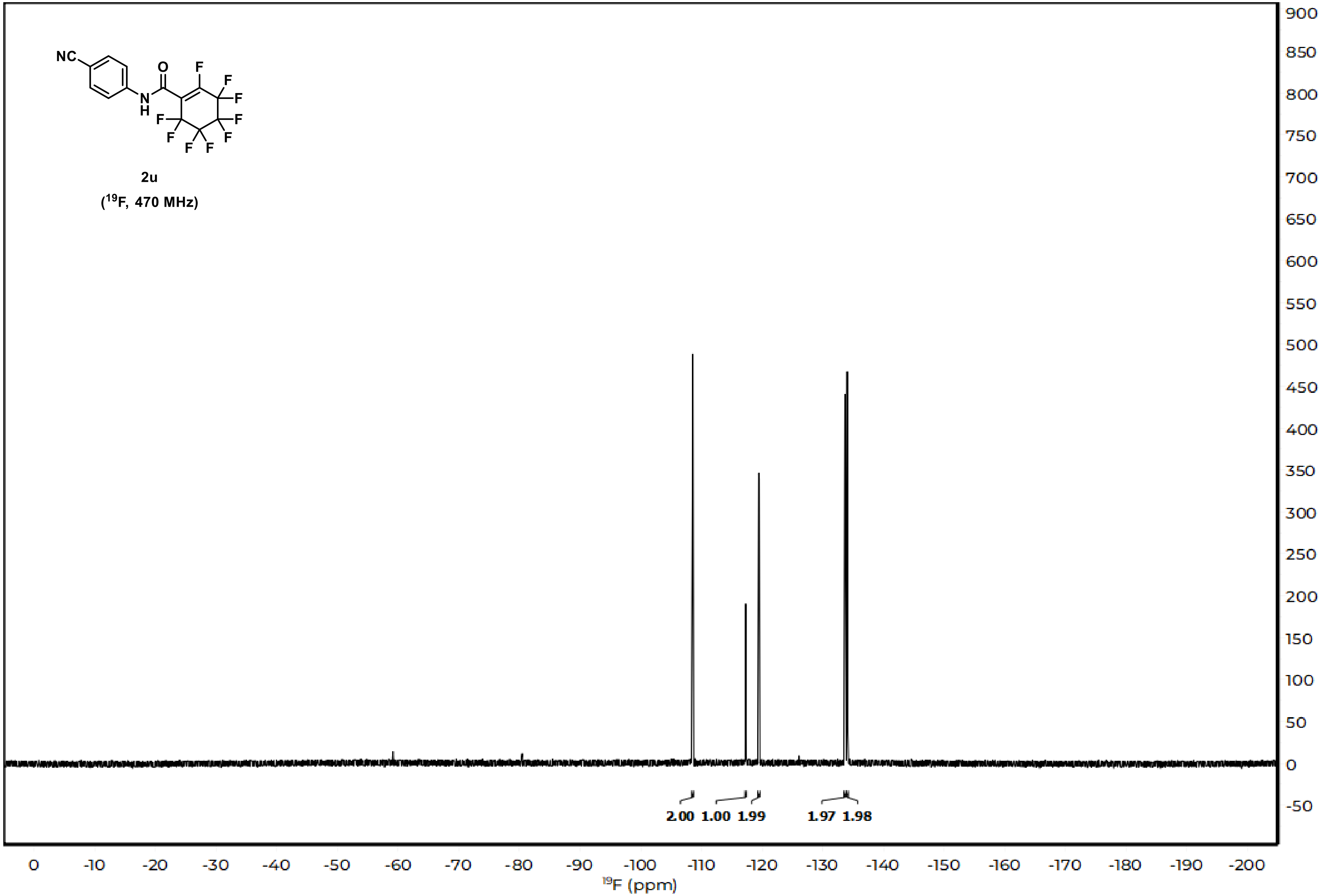

NC
O
F
N
H
2u
(19F, 470 MHz)
2.00 1.00 1.99 1.97 1.98
19F (ppm)

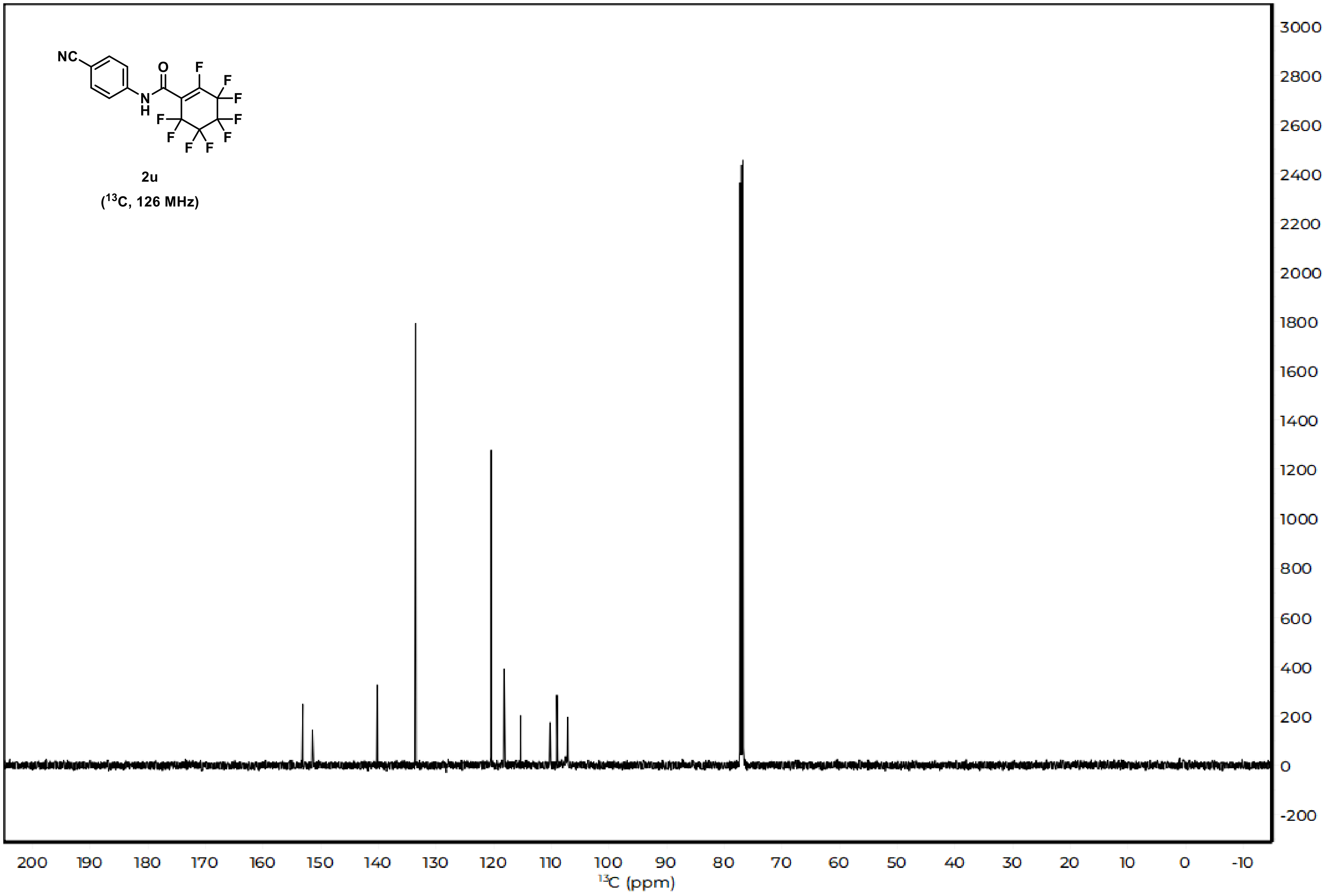
NC
O
F
N
H
2u
(^{13}C, 126 MHz)
^{13}C (ppm)
200 190 180 170 160 150 140 130 120 110 100 90 80 70 60 50 40 30 20 10 0 -10
3000 2800 2600 2400 2200 2000 1800 1600 1400 1200 1000 800 600 400 200 0 -200
